# PREP1 tumor suppressor protects the late-replicating DNA by controlling its replication timing and symmetry

**DOI:** 10.1038/s41598-018-21363-4

**Published:** 2018-02-16

**Authors:** Angela Palmigiano, Francesco Santaniello, Aurora Cerutti, Dmitry Penkov, Divya Purushothaman, Ekta Makhija, Lucilla Luzi, Fabrizio d’Adda di Fagagna, Pier Giuseppe Pelicci, Viveswara Shivashankar, Gaetano Ivan Dellino, Francesco Blasi

**Affiliations:** 1 IFOM (Foundation FIRC Institute of Molecular Oncology), via Adamello 16, 20139 Milan, Italy; 2 Department of Experimental Oncology, European Institute of Oncology, via Adamello 16, 20139 Milan, Italy; 3 Lomonosov Moscow State University, Leninskiye Gori 1, 119991 Moscow, Russia; 4 Mechano-Biology Institute, National University of Singapore, Singapore, Singapore; 5 Istituto di Genetica Molecolare, Consiglio Nazionale delle Ricerche (IGM-CNR), Via Abbiategrasso 207, 27100 Pavia, Italy; 6 Department of Oncology and Hemato-Oncology, University of Milan, Via Santa Sofia 9, 20142 Milan, Italy; 7 Present Address: Division of Genetics and Cell Biology, IRCCS San Raffaele Scientific Institute, via Olgettina 60, Milan, 20138 Italy; 8 Present Address: Oncogenomics Department, Netherland Cancer Institute (NKI), Plesmanlaan 121, 1066 CX Amsterdam, The Netherlands

## Abstract

The synthesis of middle-to-late-replicating DNA can be affected independently of the rest of the genome by down-regulating the tumor suppressor PREP1 (PKNOX1). Indeed, DNA combing shows that PREP1 down-regulation affects DNA replication rate, increases the number of simultaneously firing origins and the asymmetry of DNA replication, leading to DNA damage. Genome-wide analysis of replication timing by Repli-seq shows that, upon PREP1 down-regulation, 25% of the genome is replicated earlier in the S-phase. The targeted DNA sequences correspond to Lamin-Associated Domains (LADs), and include late-replicating (LRRs) and temporal transition regions (TTRs). Notably, the distribution of PREP1 DNA binding sites and of its target genes indicates that DNA replication defects are independent of the overall PREP1 transcriptional activity. Finally, PREP1 down-regulation causes a substantial decrease in Lamin B1 levels. This suggests that DNA is released from the nuclear lamina earlier than in the control cells and is available for replication, thus explaining timing defects and DNA damage.This is the first evidence that the replication timing of a specific fraction of the human genome is affected by PREP1 tumor suppressor. This previously unknown function might significantly contribute to the genomic instability observed in human tumors.

## Introduction

Many tumor suppressors prevent DNA damage which is the major cause of cancer^1^. These functions, although diverse, are directed to the entire genome and not to a specific subset of DNA sequences. DNA replication is regulated in time and space and is conserved through multiple cell cycles. Genes regulating the genome-wide replication timing have been identified^2^ and their absence induces global genomic alterations^3^. Therefore, fine regulation of replication timing is crucial for DNA protection. Moreover, DNA replication timing is also associated with the position of DNA in the nucleus as shown by the finding that peripheral, Lamins-bound, DNA is late replicating^4^.

PREP1 (PKNOX1) is a homeodomain transcription factor of the TALE (Three Amino Acid Loop Extension) super class of homeobox proteins, and is involved in both embryonic development and tumorigenesis^5,6^. Like other components (Pbx1, Meis1) of this super class^7,8^, *Prep1* is essential for embryonic development^9,10^. *Prep1*^*−/*−^ null embryos die around embryonic day 6 because the epiblast undergoes p53-dependent apoptosis; since the phenotype is exacerbated in an *Atm*^*−/−*^ background in which DNA repair is deficient, this phenotype likely depends on DNA damage^9^. Moreover, 75% of the homozygous hypomorphic *Prep1*^*i/i*^ mutant embryos that express only 2% of the normal Prep1 mRNA level, live longer dying at e17.5^10^. The 25% that survive, live an almost normal-length life but develop tumors at high frequency^11^. Importantly, depletion or lack of PREP1 in *in vitro* cultured fibroblasts induces accumulation of DNA damage foci^12^. These and other data^13^ indicate that *Prep1* is a tumor suppressor gene that acts by preventing DNA damage. However, the mechanism is unknown. The relevance of lack of PREP1 tumor suppressor in human cancer is demonstrated by the finding that about 50% of over one thousand human tumors lacks PREP1, which is instead expressed in the normal tissue^11^.

To better understand the molecular basis of PREP1 tumor suppression, we have analyzed the effect of PREP1 down-regulation on DNA replication. Our data show that, under these conditions, the timing, rate and symmetry of DNA replication are all affected, concurrently with the induction of DNA damage. Uniquely, PREP1 targets the fraction of the genome corresponding to the silenced, middle-to-late-replicating Lamin Associated DNA (LADs)^14^, which is consistent with the sizeable reduction of the Lamin B1 levels.

## Results

**Figure 1:**
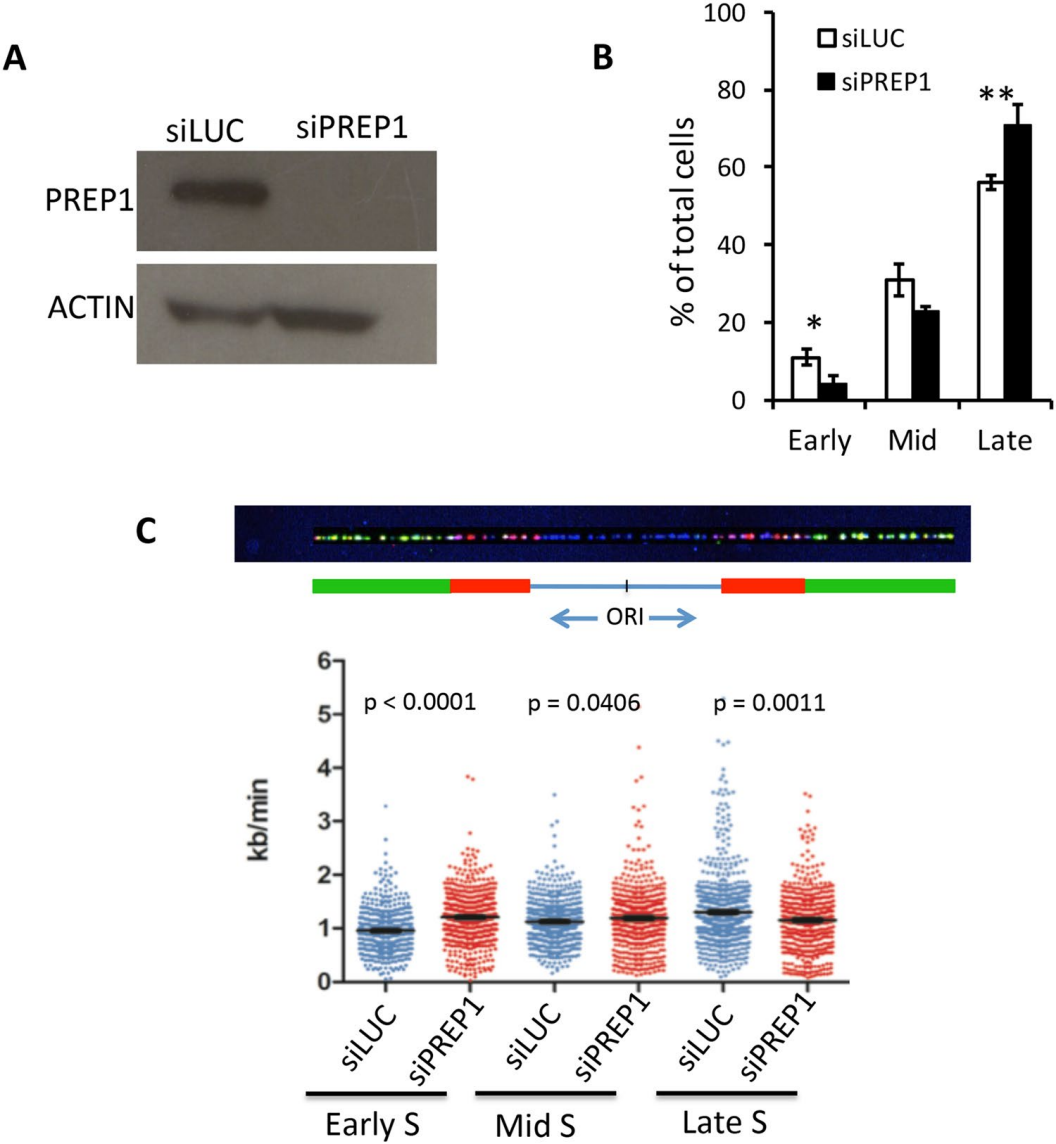
PREP1 down-regulation affects cell cycle and DNA replication rate of HeLa cells. (**A**) Immunoblot on a whole cell lysate showing the efficiency of PREP1 down-regulation 48 hrs after transfection with oligonucleotides 607 and 900. Luc siRNA was used as control. Actin was used as loading control. (**B**) Cell cycle analysis (see Methods). Percent of cells (normalized to the number of cells at the end of BrdU pulse, t = 0) in early-, mid- and late-S phase fractions, measured 4 hrs after the release from BrdU administration, in siLuc- (empty) and siPREP1-(black) transfected cells. The data are the average of three independent experiments. Asterisks indicate significant *p*-values (Student’s T test): 0.0203 (Early-S), and 0.0172 (Late-S). (**C)** (Top) Representative DNA fiber showing the incorporation of I-dU (red) and Cl-dU (green) DNA precursors. The mid-point between two red stretches is the replication origin. The length of the red stretches and the time of incubation allow the measurement of the replication rate. (Bottom) Measurement of DNA replication rates on individual DNA fibers (n = 511 and 565 in siLuc and siPREP1 cells, respectively). *p*-value (Student’s T-test), as indicated.

To study the effect of PREP1 down-regulation on DNA damage and DNA replication, we chose HeLa because of the wealth of relevant DNA replication information available from these cells. Three siRNA oligonucleotides, namely 607, 900 and 1406, were transfected and their individual effect observed after 24–72 hrs (Methods). Two of them, 607 and 900, drastically reduced the level of PREP1 in HeLa cells 48 hrs after transfection, with no effect on cell growth (Supplementary Figures S1A and S1B). Hence, they were used together and their efficiency on PREP1 level in HeLa cells 48 hrs after transfection is shown in Fig. 1A. Bacterial luciferase siRNA was used as control. Although not shown, in each of the following experiments the down-regulation of PREP1 has been verified by immunoblotting on whole cell lysates.

### PREP1 down- regulation accelerates the exit from early-S phase

We first tested the effect of PREP1 down-regulation on the cell cycle. HeLa cells were transfected with PREP1 or Luc siRNAs oligonucleotides, and 48 hrs later they were pulse-labeled (20 minutes) with BrdU. Cells were then incubated in BrdU-free medium (t = 0), and the number of BrdU-positive cells in the early-, mid- and late-S phase were counted at t = 2, 4, 6, 8 and 24 hrs (see Methods). Figure 1B shows that at t = 4 hrs, the percent of BrdU-containing cells in the early S-phase was slightly lower in PREP1 down-regulated than in control cells, whereas the opposite was observed for cells in late-S. This difference, although not always significant, was observed almost at all time points (Supplementary Figures S2A and S2B). Thus, PREP1 down-regulation appears to accelerate the exit from early-S phase without affecting the overall cell cycle, as also indicated by the lack of effect on growth rate (Supplementary Figure S1B) and total number of cells in S-phase (Supplementary Figure S2B). Similar results were obtained in normal human BJ fibroblasts (Supplementary Figure S2C) and suggest a complex effect of PREP1 down-regulation on DNA replication rate.

### PREP1 down-regulation impairs DNA replication dynamics, increases unidirectional forks and induces DNA damage

To better understand the impact of PREP1 down-regulation on DNA replication in early-, mid- and late-S phase, we used DNA molecular combing^15,16^, which shows the incorporation of fluorescent precursors in the newly replicated DNA fibers (see Methods). Figure 1C shows that the replication fork speed significantly increased in siPREP1 cells compared to control (siLuc cells), but only in the early-S phase (1.208 *vs*. 0.955 kb/min). Fork speed was instead almost identical to control during the mid-S phase (1.124 *vs*. 1.193 kb/min) and decreased during the late-S phase (1.152 kb/min *vs*. 1.304 kb/min). Remarkably, these data are in agreement with the cell cycle data of Fig. 1B.

**Figure 2:**
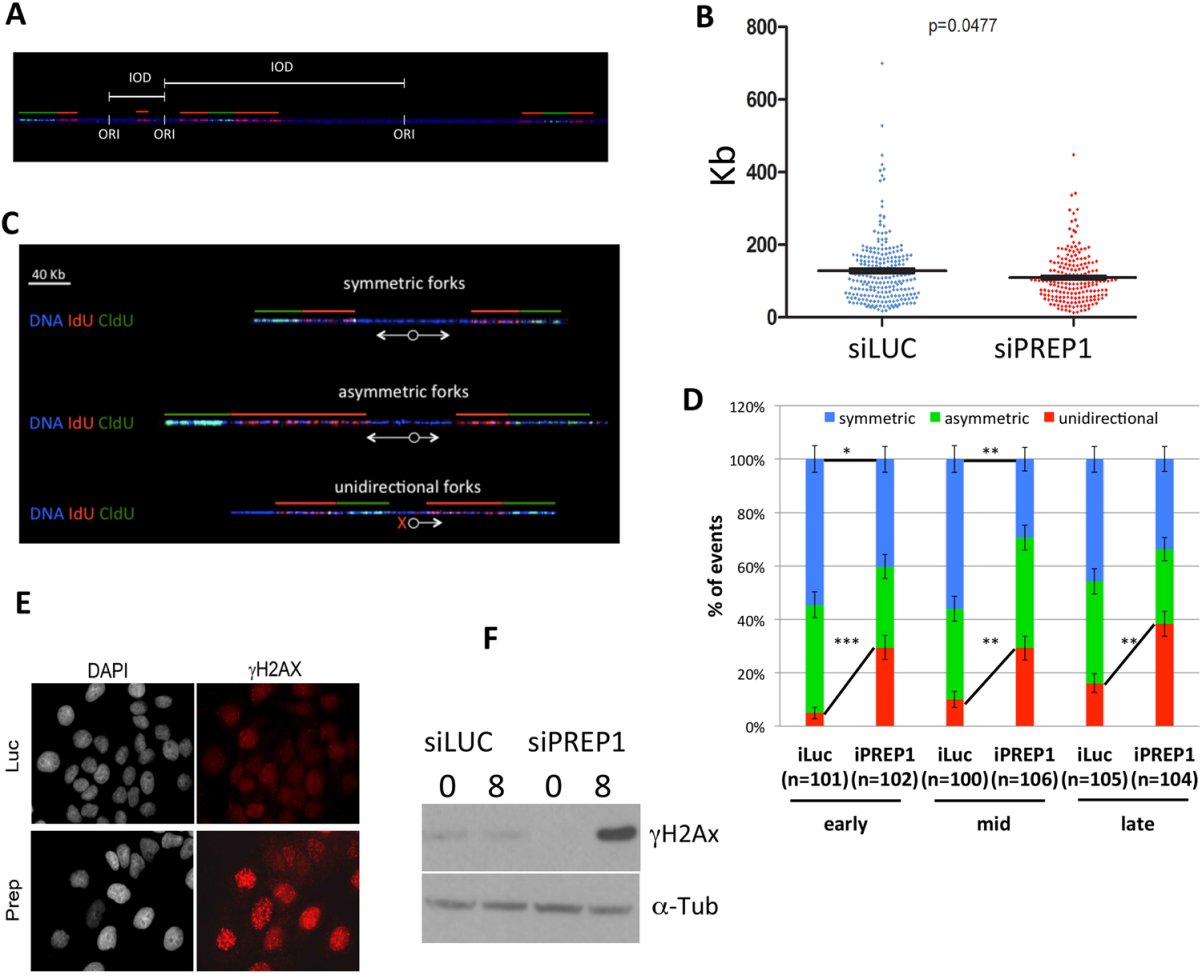
PREP1 down-regulation increases inter-origin distances, induces unidirectional forks and results in DNA damage. (**A**) A DNA fiber example with three origins of replication and the inter-origin distances (IOD) (see Methods). (**B**) Average IODs in control siLuc and siPREP1 HeLa cells. For this measurement, all data from early-, mid-, and late-S phase have been pooled together (total of 236 siLuc and 197 siPREP1 fibers). *p*-value, as indicated. (**C**) Example of fibers showing symmetric, asymmetric and unidirectional DNA replication forks (see Methods). (**D**) Proportion of the symmetric, asymmetric and unidirectional DNA replication forks in siLuc *vs*. siPREP1 cells in early-, mid- and late-S phase, as indicated. Numbers of fibers analyzed (n) and *p*-values (stars), as indicated (*0.0009; **0.0003 to 0.0005; ***4e^−6^). (**E**) Immuno-fluorescence showing DNA damage in HeLa cells using the γH2Ax marker (red fluorescence). DAPI (white) was used to identify the nuclei. (**F**) Immunoblotting analysis of siLuc and siPREP1-transfected HeLa cells arrested in G1 by a double thymidine block (0 h) or 8 hrs after release (8 h).

To further characterize the effect of PREP1 down-regulation on DNA replication, we measured the inter-origin distance (IOD). To avoid IOD underestimation, we used only fibers three times longer than the average IOD^17,18^. In PREP1 down-regulated cells IODs were 15% shorter than in control (Fig. 2A,B), indicating an equivalent increase of simultaneously activated, i.e., fired, origins.

DNA combing also allows to evaluate the impact of PREP1 on the symmetry of fork progression. We defined three symmetry groups based on the ratio between the replication rate of right *vs*. left forks: symmetric, asymmetric and uni-directional (Fig. 2C; for details see Methods). Figure 2D shows an overall reduction of symmetric forks in PREP1 down-regulated cells throughout the S phase, with a dramatic increase (about 30%) in unidirectional forks. Supplementary Figure S3 shows the actual complete data.

Since unidirectional forks represent the highest level of asymmetry which is associated with fork collapse and subsequent DNA damage, we assessed the impact of PREP1 down-regulation on DNA damage. Indeed, as shown in Fig. 2E, PREP1 down-regulation induced DNA damage in HeLa, as shown by the accumulation of γH2Ax foci, as previously found in other cell lines^12^. Finally, we measured γH2Ax in G1-arrested and S-phase cells by immunoblotting, and observed a dramatic increase in S-phase, only in the siPREP1 cells (Fig. 2F).

Taken together, this data show that PREP1 down-regulation impairs DNA replication dynamics by affecting rate, number of origins and fork symmetry, thus producing a high level of unidirectional replication forks that are likely the main cause of the observed DNA damage.

### PREP1 down-regulation induces a Late-to-Early (LtoE) shift of the replication timing of 25% of the HeLa genome

We then mapped the genomic regions whose replication was affected by PREP1 down-regulation, using Repli-seq that allows the genome-wide analysis of the temporal order of DNA replication (see Methods)^19–21^. Forty-eight hours after PREP1 or Luc (control) siRNA transfection, asynchronous HeLa cells were BrdU-pulsed for 45 min. Cells were then FACS-sorted in six consecutive sub-populations with increasing DNA content (S1–S6), during their progression through the S phase. The BrdU-containing DNA fragments were purified by immunoprecipitation, deep-sequenced, aligned and visualized on the UCSC Genome Browser (Supplementary Figure S4).

**Figure 3:**
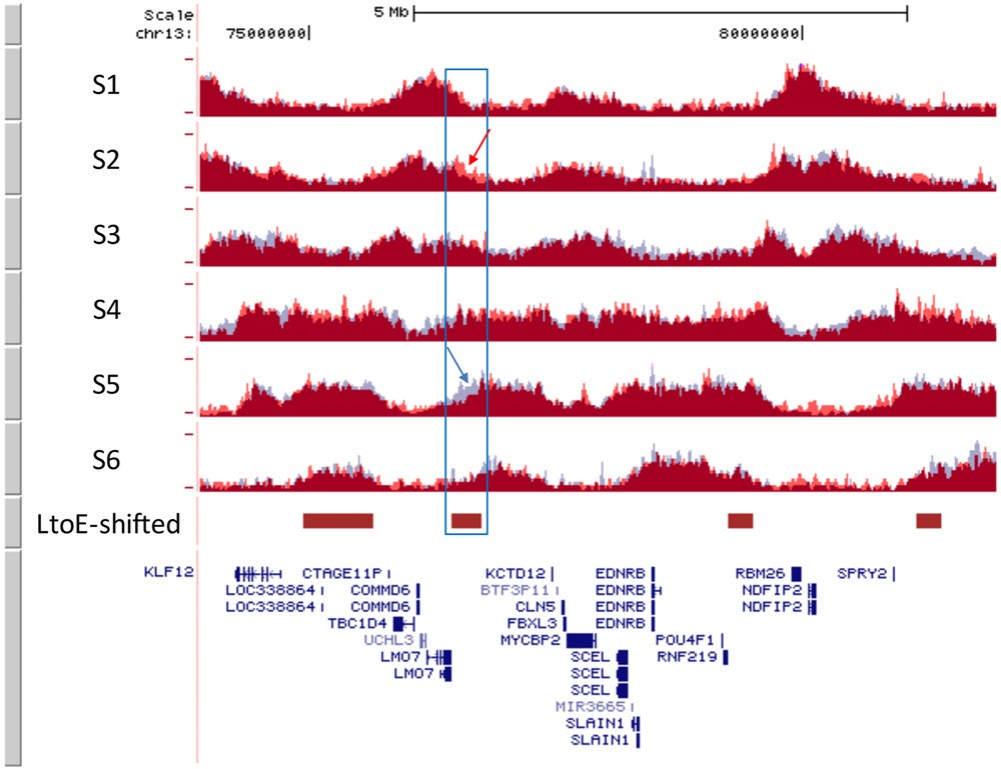
LtoE-shifted regions upon PREP1 down-regulation.Repliseq profiles (S1, early-, to S6, late-S phase) of siLuc cells (pale blue tracks) and siPREP1 (superimposed red tracks), showing a representative late-to-early (LtoE)-shifted region identified by visual inspection (empty blue box). Red and blue arrows indicate higher levels of BrdU incorporation within siPREP1 (in S2 window) or siLuc cells (in S5), respectively. Red boxes indicate late-to-early (LtoE)-shifted regions identified by bioinformatic analysis.

**Figure 4:**
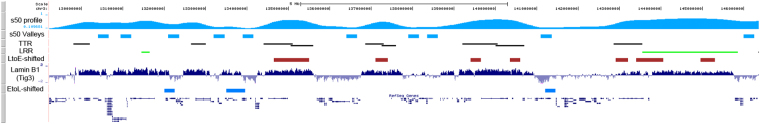
Mapping of temporally-shifted regions in siPREP1 cells. Representative genomic region (17 Mb in length) from the UCSC Genome Browser showing (top to bottom): the S50 profile and S50 valleys of siLuc cells (light blue track and boxes, respectively); Temporal Transition Regions (TTR, black) and Late-Replicating Regions (LRR, green horizontal lines); LtoE-shifted regions identified in siPREP1 cells (red boxes); Lamin B1-bound DNA (deep blue signals corresponding to positive log2-ratio scores^14^); EtoL-shifted regions identified in siPREP1 cells (dark blue boxes); position of RefSeq genes, as indicated.

Visual inspection of large genomic regions did not show gross alterations of the DNA replication profile (Supplementary Figure S4). However, when the Repli-seq profile of the siPREP1 was superimposed on that of the siLuc cells, we detected tens of genomic regions showing small differences that were compatible with early-to-late (EtoL) or late-to-early (LtoE) shifts, when comparing the two experimental conditions (Fig. 3 and Supplementary Figure S5). In order to obtain a genome-wide mapping of significant EtoL or LtoE shifts, we performed a differential analysis of replication timing (see Methods). We first measured the replication timing profile of both siLuc and siPREP1 cells. For this purpose, the HeLa genome was divided in non-overlapping 50 Kb-intervals and their S50 value (i.e. the fraction of the S phase at which 50% of each 50 Kb-interval is replicated) was calculated^21^. S50 values range from 0 (very early-) to 1 (very late-replicating). Figure 4 shows the S50 profile of a representative region from siLuc cells.

Finally, the S50 profiles of siLuc and siPREP1 cells were compared. The genome was divided into sliding windows of 250 Kb and their differential S50 value was calculated (example in Supplementary Table 1). 7,643 significantly LtoE-shifted and 1,072 EtoL-shifted windows were identified in siPREP1 compared to siLuc cells (Table 1 and Supplementary Tables 2–3**)**.

**Table 1:** PREP1 down-regulation induces Late-to-Early (LtoE) and Early-to-Late (EtoL) DNA replication shifts in HeLa cells*.

	250 Kb windows	Clustered contigs	Length of contigs (Mb)	Genome fraction
LtoE shifts	7,643	1,717	0.25–2.5	771 Mb (25.4%)
EtoL shifts	1,072	410	0.250–1.85	140 Mb (4.6%)

*LtoE and EtoL S50 shifts are calculated for each 250 Kb sliding window as:

Max S50 siPREP1 - Max S50 siLuc.

Coherently shifted 250 Kb windows often clustered together, generating 1,717 LtoE and 410 EtoL contigs (or regions) of 0.25–2.5 Mb and 0.25–1.85 Mb in length, respectively (Table 1, Figs 3 and 4, and Supplementary Figures S6 and S7A). In total, LtoE-shifted regions spanned 771 Mb, and EtoL-shifted regions 140 Mb, corresponding to 25.4% and 4.6% of the human genome, respectively (Table 1). Strikingly, almost all the regions showing differences in replication profiles initially identified by visual inspection, were detected by the genome-wide analysis (Fig. 3 and Supplementary Figure S5).

We then assessed the replication timing of the EtoL- and LtoE-shifted regions in control cells and found that they were not uniformly distributed throughout the S-phase (Fig. 4 and Supplementary Figure S6). In control cells LtoE-shifted regions were replicated in mid- and late-S phase, while EtoL-shifted ones during early-to-mid-S phase (Supplementary Figure S7B). Therefore, PREP1 down-regulation induces thousands of subtle, but significant, changes of replication timing in the HeLa genome.

To test the reproducibility of the observed shifts, we performed a biological replicate measuring the replication profiles of 19 LtoE-shifted and 18 control (i.e., showing no shift in replication timing) contigs, using specific primer pairs in Q-PCR assays. We validated 17/19 LtoE-shifted contigs and, consistently, confirmed the lack of effect in 17/18 control regions (Supplementary Table S4). A few examples are shown in Supplementary Figure S8.

Together, these data show that PREP1 down-regulation induces major changes in DNA replication timing of more than 25% of the HeLa genome. Because of the extremely low fraction of genome involved (<5%), we have not considered the EtoL shifts in detail in this study.

### LtoE-shifted contigs are enriched in Temporal Transition Regions and Late-Replicating Regions, and overlap with Lamin-Associated Domains

Repli-seq typically shows hundreds of symmetrical early-to-late transitions (inverted-Vs, Supplementary Figure S4) originating at replication initiation regions (the “inverted-V apexes”). Each inverted-V apex corresponds to a “valley” (in the S50 profile; Fig. 4 and Supplementary Figure S4) that is enriched in DNA replication origins^19–21^ (see Methods).

We then investigated the association of LtoE-shifted contigs of siPREP1 cells with S50 valleys. In siPREP1 and siLuc HeLa cells we found 1,859 and 1,868 valleys, respectively, which showed almost complete overlap (>93%; n = 1,741) (Supplementary Table S5). However, only a minority of LtoE-shifted contigs overlapped with S50 valleys (25%; n = 437/1,717); therefore, the differentially replicating regions upon PREP1 down-regulation are not those particularly enriched in DNA replication origins in control cells.

Thus, we asked whether LtoE-shifted contigs were associated with Temporal Transition Regions (TTRs) and/or late-replicating regions (LRRs). Temporal Transition Regions (TTRs)^22^ are defined as genomic regions, several hundreds of Kb in length, that separate early- from late-replicating regions (see Methods) with reduced replication origin activity^23^. Unlike valleys, the majority of TTRs overlapped with LtoE shifts (75%; n = 671/891; Fig. 4, Table 2 and Supplementary Table S6**)**. Likewise, 45% of the LtoE-shifted regions (n = 751/1,717) overlapped with a TTR.

**Table 2:** LtoE shifts are mostly present in temporal transition (TTR) and late replicating regions (LRR).

Total Nr. of TTR	891
Total Nr. of LtoE shifts	1,717
TTR/LtoE overlaps*	671/891 (75%); ^$^*p* value: 2.2e^−16^
LtoE/TTR Overlaps*	751/1,717 (45%); ^$^*p* value: 2.2e^−16^
Total nr. of EtoL shifts	410
LtoE overlapping with LRR	504/1717 (29%); p < 2.2e^−16^
LRRs overlapping with LtoE	249/348 (70%); p < 2.2e^−16^

^$^p-values refers to overlaps with random regions having the same size distribution as the LtoE (see Methods).

*The overlaps were measured allowing for a ±5 Kb tolerance.

The LtoE shifts also targeted late-replicating regions (LRRs), defined in this study as the genomic regions not overlapping with TTRs and having an S50 > 0.65 (see Methods). Overall, 29% (n = 504/1,717) of the LtoE-shifted contigs overlapped with a LRR (Table 2 and Fig. 4).

In conclusion, 74% of the LtoE shifts generated upon PREP1 down-regulation target TTRs or LRRs (45% and 29%, respectively). Together, these data suggest that the firing of origins shown by DNA combing and that were activated upon PREP1 down-regulation, originates from TTRs and LRRs.

LtoE-shifted regions show features similar to LADs^14^. About 40% of the human genome in Tig3 cells is bound to Lamin B1 (LADs, Lamin Associated Domains), and is enriched in gene-poor, late-replicating, and silenced DNA sequences. We then investigated the association of LtoE-shifted regions with LADs. Strikingly, we found a strong overlap between TIG3 LADs and the siPREP1-induced LtoE shifts in HeLa cells (Fig. 4); in particular, 72% (n = 1,237/1,717) of LtoE contigs were Lamin B1-bound (Table 3). Together, these data indicate that PREP1 down-regulation affects the replication of middle-to-late replicating genomic regions that are normally bound to Lamin B1.

**Table 3:** LtoE contigs are enriched in Lamin-Associated-Domains and depleted in PREP1 binding sites.

	Number	%	p-value*
LtoE contigs with LaminB1 binding sites	1,237/1,717	72	<2.2e-16 (enrichment of LaminB1-positive LtoE contigs)
PREP1 peaks within LtoE conting in siLuc cells	471/3,909	12	<2.2e-16 (depletion of PREP1 peaks within LtoE contigs)

*Fisher Test vs. random genomic contigs with 100 iterations (see Methods).

### LtoE shifts do not correspond to genomic regions associated with PREP1 transcriptional activity

**Figure 5:**
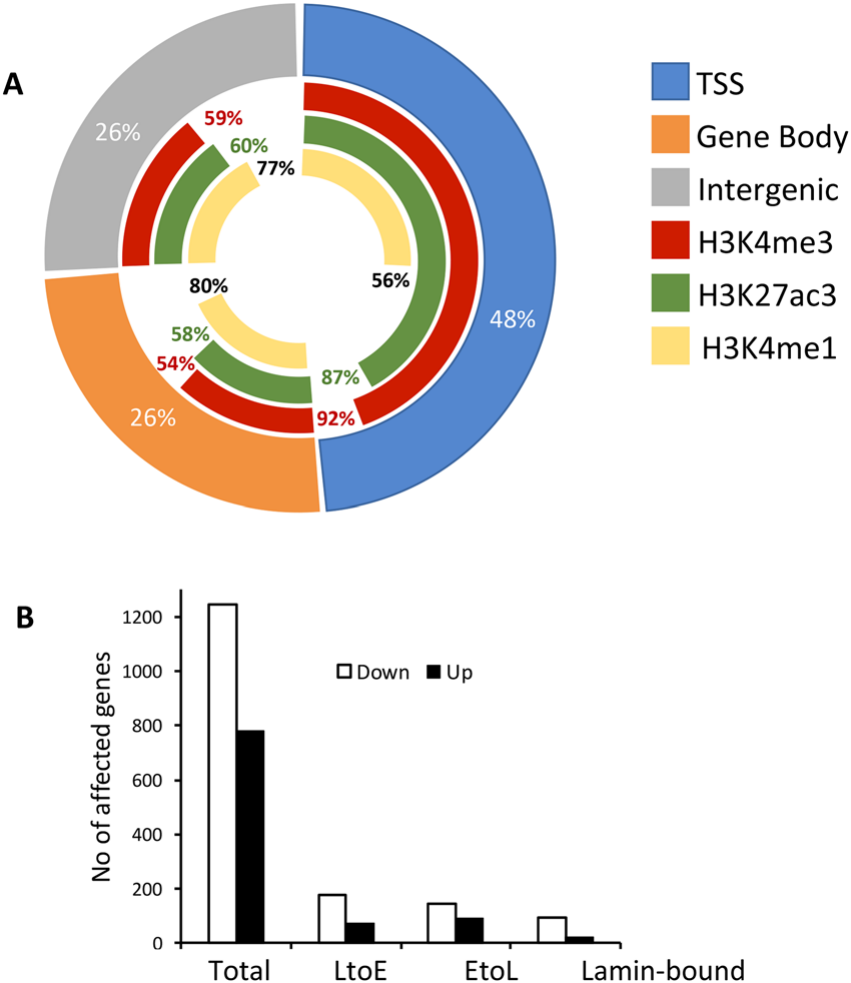
PREP1 DNA-binding sites landscape in HeLa cells. (**A**) A summary of the annotation of the 3,909 PREP1 peaks (*p* < 10^−5^) obtained by ChIP-seq: Promoter (±2.5 Kb from TSS), gene body, intergenic, and their overlaps with different histone marks. (**B**) Number of genes whose transcription is affected by PREP1 down-regulation: total, within LtoE contigs, within EtoL contigs, Lamin-bound, as indicated. The results are expressed as average of biological triplicate. Down and up indicate decrease (>40%), or increase (>2X) of the mRNA levels, respectively, in siPREP1- *vs*. siLuc-transfected cells.

We then asked whether the function of PREP1 as a DNA-binding transcription factor was associated with its effects on DNA replication. For this reason, we performed PREP1 ChIP-seq (see Methods) on control HeLa cells and measured the association of PREP1 binding sites to LtoE shifts. 3,909 PREP1-enriched regions, or peaks, were detected (Supplementary Table S7). About 70% of the PREP1 peaks were associated with chromatin modifications typical of active gene promoters (H3K4me3^+^ and H3K27Ac^+^) (Fig. 5A). These data are consistent with the previously observed PREP1 distribution in the mouse genome^24–26^.

PREP1 ChIP-seq in HeLa cells allowed the identification of its consensus sequence, 5′-TGAXTGACAG-3′ (Supplementary Figure S9), that confirmed previous data obtained in mouse cells^24–26^. Strikingly, the PREP1 binding sites were significantly under-represented in LtoE-shifted regions (Table 3), strongly arguing against a direct role of PREP1 binding to DNA in the observed shift of DNA replication from middle-to-late S towards earlier S-phase.

In a further attempt to correlate PREP1 transcription activity with LtoE shifts, we performed RNA-seq (see Methods), comparing transcription profiles of siLuc and siPREP1 cells (Fig. 5B). We reasoned that if PREP1-dependent transcription is associated to its effect on DNA replication, one would expect an enrichment in the LtoE shifts of the genes whose transcription is affected (i.e., either down-regulated by >40% or up-regulated >2-fold) by PREP1 down-regulation. PREP1 down-regulation significantly changed the expression of 1,815 genes, most of which showed reduced RNA levels (Fig. 5B). Differentially expressed genes (DEGs) were depleted within the LtoE-shifted regions (n = 249/1815, p < 2.2e^−16^) (Table 4, Supplementary Tables S8 and S9), and only a minority of them mapped within LADs (Fig. 5B). Likewise, LtoE contigs containing DEGs were under-represented (n = 215/1717, p < 2.2e^−16^).

**Table 4:** Differentially regulated genes are depleted in the LtoE shifted regions.

	Contigs	Number	Enrichment/depletion*	p-value
Contigs containing DEGs	LtoE	215/1717	Depleted	<2.2e^−16^
	EtoL	152/410	Enriched	<2.2e^−16^
DEGs within Contigs	LtoE	249/1815	Depleted	<2.2e^−16^
	EtoL	237/1815	Enriched	<2.2e^−16^

*The enrichment or depletion have been evaluated over random association (see Methods).

In conclusion, both ChIP-seq and RNAseq experiments argue against the association of the PREP1 transcriptional activity with its role in DNA replication of LtoE-shifted region. This conclusion does not exclude that PREP1 might have a specific direct effect on one or few genes that contribute to the observed DNA replication phenotype.

### PREP1 down-regulation affects the level of Lamin B1

**Figure 6:**
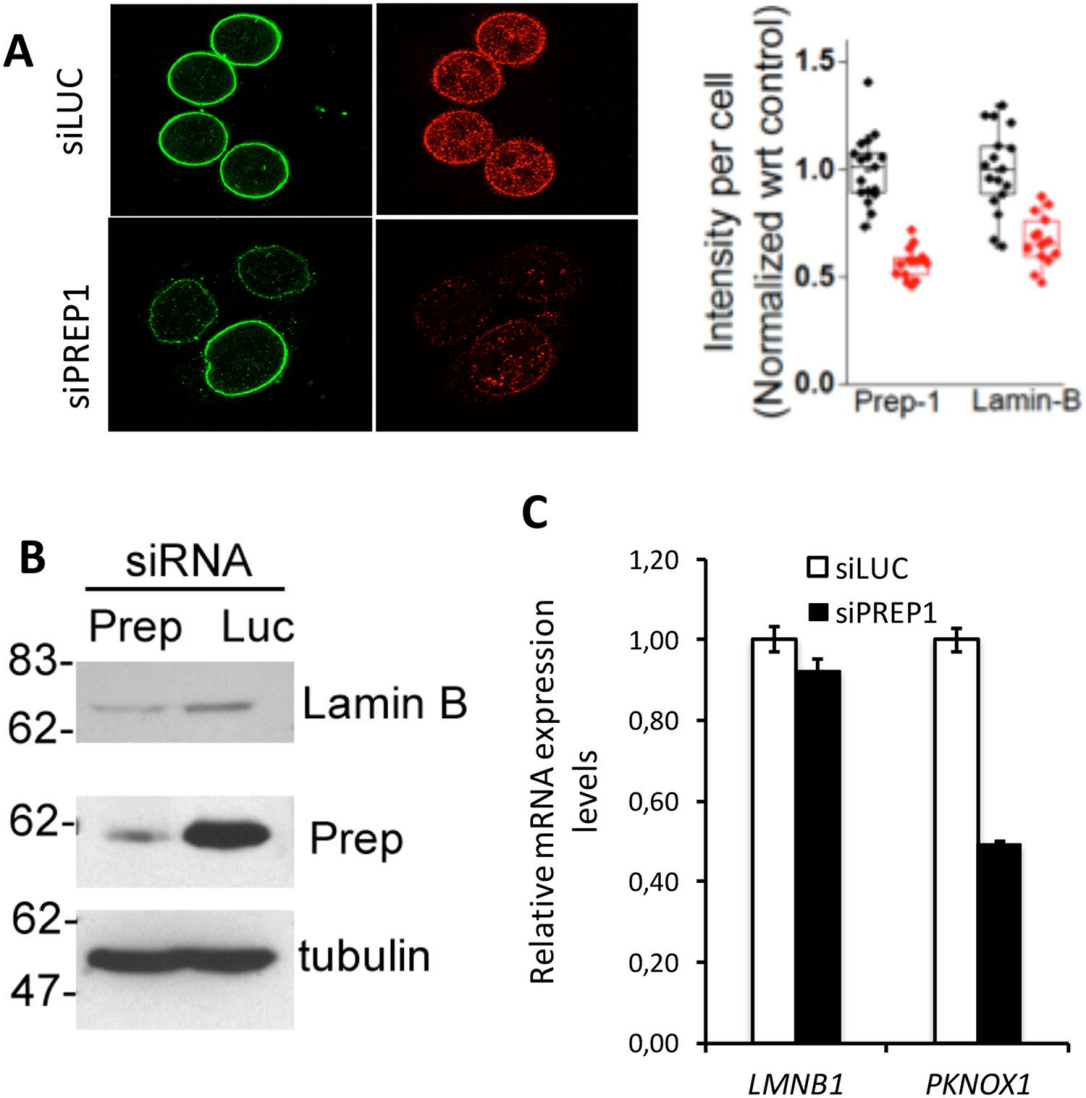
PREP1 down-regulation causes loss of Lamin B1. (**A**) Immunofluorescence staining of Lamin B1 (green) and PREP1 (red) in HeLa cells transfected with control siLuc (top) or siPREP1 oligonucleotides (bottom). The upper right panel shows the quantitation of PREP1 and Lamin B1 signal intensity per cell in control siLuc (black) and siPREP1-transfected cells (red). (**B**) Immunoblotting analysis on total cell lysates of HeLa cells treated with control (Luc) or PREP1 siRNA, as indicated. (**C**) qPCR analysis of PREP1 and Lamin B1 mRNA in siLuc and siPREP1 cells (average ± SD of three replicates).

We finally asked whether the observed LtoE shifts of the Lamin-associated DNA was linked to global changes in the Lamin levels. We measured a substantial (40%) decrease of Lamin B1 upon PREP1 down-regulation by both immunofluorescence and immunoblotting (Fig. 6A,B). However, the level of Lamin B1 mRNA was not affected (Fig. 6C), thus indicating that Lamin B1 decrease was due to its post-transcriptional regulation.

Lamins bind a consistent fraction of chromosomal DNA providing conditions for its peripheral localization, gene silencing and late replication^14,27^. Indeed, it has been shown that depletion of Lamin B1 induces alteration of DNA replication timing, gene expression, and loss or reduction of peripheral chromatin organization^28^. Moreover, a decrease of Lamin B1 has been associated with a release of chromatin from LADs and increased frequency of mutations in specific lymphocytes populations, with consequences in lymphomagenesis^29^. Our results, therefore, show that PREP1 down-regulation, through the alteration of Lamin B1 stability, contributes to the observed alterations of replication timing and genomic stability.

## Discussion

PREP1 is a transcription factor essential in mouse embryonic development, when it appears to prevent DNA damage accumulation^9,12^. The present study shows that the DNA damage accumulation observed in PREP1-missing cells can be due to alteration of the DNA replication timing. Moreover, we demonstrate that replication timing defects do not affect the whole genome, but mainly the late-replicating and gene-poor LADs, whose timing is modified independently of the rest of genome. Finally, we show that the observed changes in replication timing are associated with DNA damage, at least in part through alteration of DNA replication symmetry or unscheduled origin firing.

These results link defects in DNA replication to the developmental defects^9^ and the tumorigenicity^11^ of PREP1-missing or down-regulated cells, explaining its function as a tumor suppressor^6^. Hence *PREP1* is not, or not only, a developmentally essential gene, but it also controls basic cellular functions, like DNA replication timing.

PREP1 down-regulation causes shifts in DNA replication timing (Table 1). Indeed, a substantial amount (25%) of the genome, particularly late-replicating and gene-poor sequences (LRRs and TTRs) (Fig. 4, Tables 2 and 3), is replicated earlier in the S phase than in control. However, the S50 valleys, enriched in replication origins, are conserved in PREP1 down-regulated cells. Since PREP1 down-regulation induces a decrease in the inter-origin distance (Fig. 2C,D), and considering constant processivity of DNA polymerases in siPREP1 and siLuc cells, altogether our data strongly suggest an increase in simultaneously activated origins within LRRs and/or TTRs, which normally show reduced replication origin activity^23^.

A large fraction of the LAD sequences identified in fibroblasts^14^ overlaps with the LtoE replication shifts induced by PREP1 down-regulation in HeLa cells (Table 3), an extremely high overlap considering the differences in cell type. Most importantly, about three quarters of the LtoE shifts occur in LADs. Clearly, LADs appear as a major, if not the only, target of PREP1 down-regulation.

Gene promoters are enriched in origins of replication^21^. Since PREP1 mainly binds to promoters^24–26^, PREP1 might directly affect replication origin firing. However, LtoE-shifted regions are normally depleted both in PREP1 DNA-binding sites (Table 3) and in genes transcriptionally affected by PREP1 down-regulation (Fig. 5B and Table 4). Moreover, the genes coding for DNA replication-initiation factors (e.g., *ORC1–6, MCM2–7*, etc.) are not present among those whose transcription is affected by PREP1 down-regulation (Supplementary Tables S8, S9; see also raw data in GSE 101776).

Thus, if PREP1 does not directly bind to LADs/LRRs and TTRs, how are these regions targeted by PREP1 down-regulation? Levels of Lamin B1 have already been shown to affect DNA replication timing and DNA damage^14,27–29^. In particular, the level of Lamin B1 has been directly associated with the release of DNA from LADs, the frequency of DNA mutations and chromatin compaction^29,30^. Therefore, a decrease in Lamin levels (Fig. 6), induced by PREP1 down-regulation, might cause premature release of TTRs and LLRs DNA from the nuclear lamina, thus allowing earlier firing of replication origins therein. The unscheduled replication then leads to DNA damage, also through transcription-replication clashes^31^ caused by the absence of PREP1.

Since the decrease in Lamin B1 levels is not due to a direct transcriptional effect (i.e., PREP1 down-regulation does not directly affect *LMNB1* gene expression), it might affect, for example, the stability of the protein. Thus, the effect of PREP1 on replication timing and DNA damage is likely indirect, even though very substantial.

Unscheduled origin firing and the lack of replication symmetry appear as the major cause of DNA damage following PREP1 down-regulation. Indeed, unidirectional forks, which represent the extreme consequence of replication asymmetry, are mostly associated with fork collapse and DNA damage^32^.

In conclusion, the function of PREP1 tumor suppressor, even if not directly causative, is linked to the definition of DNA replication timing of a significant portion of the genome, DNA replication symmetry, and genomic stability. Therefore, PREP1 is a novel genome guardian that, through its effect on Lamin B1 protein levels, specifically protects late-replicating regions of the genome.

## Materials and Methods

### Cells

HeLa cells were cultured in DMEM and 10% heat-inactivated FBS. BJ human fibroblasts (ATCC) were cultured in MEM and 10% heat-inactivated FBS and used at 30–35 population doublings.

### PREP1 Down-Regulation

To down-regulate PREP1 in HeLa cells and BJ fibroblasts a mixture of two independent siRNA (Dharmacon, Lafayette, USA) were used (siRNA 607 = GATTTCTGCAGTCGATACA; siRNA 900 = CTCCCAGCTTCAGTTACAG). As control we used an siRNA targeting firely luciferase (CATCACGTACGCGGAATAC). 10 nM dsRNA were transfected using RNAi Max lipofectamine (Invitrogen). To assess PREP1 down-regulation, 30 µg protein extracts were subjected to SDS-PAGE gel and PREP1 was detected using a monoclonal CH12.2 antibody.

### Immunofluorescence

HeLa cells were grown on coverslips, transfected with siRNA for 24 h, fixed with 4% paraformaldehyde in phosphate-buffered saline (PBS) for 10 min, permeabilized with PBS 0.1% Triton X-100 and blocked with PBS-4% BSA (Sigma) for 1 h. Primary and secondary antibodies were incubated in blocking solution (PBS-4% BSA) for 90 min and 1 h, respectively. DNA damage foci were detected using phospho-H2AX (Ser139) antibody (Millipore; dilution 1:200) and Cy3 Donkey anti mouse secondary antibody; nuclei were visualized by DAPI staining (dilution 1:5000). For Lamin B1 cells were incubated in primary antibody (goat polyclonal SC 30264 from Santa Cruz Biotechnology) (1:400) and secondary antibody (Donkey anti goat Alexa 488 (1:400) in blocking solution for 1 hour at RT, counterstained with DAPI and coverslips mounted in glycerol mounting solution. Images were acquired using on Leica SP2 confocal microscope equipped with 63X/1.4NA objective. Mean fluorescence intensity of Lamin B1 was quantified using ImageJ software.

### Cell cycle flow cytometry analysis

Twenty-four hours after siRNA transfection, 3 × 10^6^ cells were pulse-labeled with 33 µM BrdU (Sigma-Aldrich) for 20 min at 37 °C, washed twice with PBS and released in BrdU-free medium for various times. At each time point, cells were resuspended in 750 µl PBS and fixed with 2250 µl of pure ethanol (Carlo Erba) for 30 min on ice. Cells were then washed with 1 ml (1%) BSA (Sigma-Aldrich) in PBS, incubated with 1 ml 2 N HCl (Carlo Erba) for 25 min at room temperature to denature DNA when 1.5 ml 0.1 M Sodium Borate (Sigma-Aldrich) was added for 2 min at room temperature. Cells were washed twice with 1 ml of PBS-BSA (1%) and incubated 1 h at room temperature with 100 µl of pure anti-BrdU antibody (Becton Dickinson) diluted 1:50 in PBS-BSA (1%). Washed cells were then resuspended in 100 µl anti-mouse FITC diluted 1:50 in PBS-BSA (1%) and incubated 1 h at room temperature. Cells were washed, resuspended in 2.5 µg/ml propidium iodide (Sigma-Aldrich), 250 µg/ml RNase and incubated overnight at 4 °C. The analysis of BrdU positive cell and DNA content was performed using a FACSCalibur with 488 nm laser (Becton Dickinson) and Cell Quest software (30,000 cells were analyzed for each sample).

### DNA Molecular Combing

Forty-eight hours after siRNA transfection, cells were pulse-labeled with the thymidine analogue IdU for 30 minutes, washed and labeled with CldU for further 30 minutes^15,16^. Cells were incubated with Propidium Iodide, sorted (flow cytometry) into early, mid and late S phase cells and embedded in agarose plugs (50,000 cells/plug). The DNA was extracted and aligned on silanized coverslips by DNA molecular combing. Immunofluorescence was performed to detect the thymidine analogues and the DNA fibres using specific primary and secondary antibodies (IdU: mouse anti-BrdU, Becton Dickinson and anti-mouse IgG2a Alexa 546 (Molecular Probes); CldU: rat anti-BrdU, Abcam, and goat anti-rat-Alexa Fluor 488 (Molecular Probes); DNA fibers: mouse anti-ssDNA antibody (Chemicon) and goat anti-mouse IgG1-Alexa Fluor 647 (Molecular Probes). Images were acquired automatically with a spinning disk confocal microscope, and the individually labelled DNA molecules were manually measured with ImageJ.

We followed published recommendations^17,18^ to determine the role of fiber length in the quantitative analyses. For replication symmetry measurements, we defined three replication forks symmetry groups: a. symmetric, in which left and right fork speed was comparable, with a <30% difference between left/right fork speed; b. asymmetric, in which the left/right fork speed differed by >30%; c. unidirectional fork progression, in which only one of the sister forks is proceeding from the replication origin, with left and right fork speed differing at 100%, thus representing the highest level of asymmetry.

### Repli-seq and genome-wide differential analysis of replication timing

Briefly, exponentially growing HeLa cells transfected with siLuc or siPREP1 were pulse labeled with 50 μM BrdU (Sigma-Aldrich, Milan, Italy) for 45 min, harvested and stained with PI. Cells were FACS-sorted in six sub-phases of the S phase (S1–S6) according to their increasing DNA content. Sorted cells were then lysed, DNA extracted by phenol-chloroform and immunoprecipitated using anti-BrdU antibody (Becton Dickinson Italia, Milan, Italy). Immunoprecipitated DNA was purified and quantified with PicoGreen staining (Invitrogen, CA, USA). Sequencing of the DNA from each S subpopulation was performed as previously described^24,25^ using Illumina single-end sequencing reactions (36 nt) performed on gel-excised DNA fragments (200 bp in length). After quality check, the reads were aligned to the reference human genome (hg19) using BWA^33^ (version 0.6.2-r126) with the BWA-backtrack algorithm and filtered to eliminate duplicate sequences. Numbers of sequences, from the initial raw reads throughout the various steps of analysis/processing, are shown in Supplementary Figure S10.

The differential analysis of replication timing between siPREP1 and siLUC cells was essentially divided in two phases: (1) normalization of Repli-seq data within each experimental condition and between the two conditions; (2) genome-wide comparison of the replication timing profile and estimation of statistical significance of the measured differences.

1. Normalizations: (i) the reference genome was binned in 100 bp long intervals, and the signal from the aligned data underlying these regions was calculated for each Repli-seq sample; (ii) for each condition (siPREP1 and siLUC), a trimmed mean of M-values normalization (TMM)^34^ was independently applied to the six S-phase windows, in order to account for the different “library size” (=depth of sequencing), thus allowing their direct comparison; (iii) the signal of the unsorted cells was subtracted from each S-phase window in order to reduce the signal background, or artifacts due to biases in DNA sequencing, ambiguous alignments, “signal spikes” (i.e., unspecific and very narrow high signals, invariably found in most of the sequenced samples), etc using the bigwigCompare function of the deepTools package^35^. After the subtraction, 100 bp bins occasionally resulting in negative values (corresponding to a negligible fraction of all bins) were converted to zero since they were most probably due to the absence of replication-associated signals during that particular temporal window; iv) within each experimental condition, the relative contribution of each S-phase window to the total BrdU signal, measured in genomic 100bp-long intervals, was calculated and expressed as the “S-phase window”-to-“total S-phase” signal ratio (ranging from zero to 1); v) a quantile normalization between corresponding S-phase windows of the two conditions (S1 siPREP1 vs. S1 siLuc, S2 siPREP1 vs. S2 siLuc, etc.) was applied to normalize the two experimental conditions; vi) for each condition, the signals of the six S-phase windows were finally aggregated by dividing the human genome in about 61,000 50 Kb intervals and measuring the S50 value for each of them as described in Dellino *et al*.^21^. S50 values is a replication timing estimator that measures the fraction of the S phase at which 50% of a given genomic region has been replicated^20,21^; it ranges from zero to 1 (indicating very-early to very-late replicating regions, respectively).

2. Genome-wide comparison of the replication timing profiles: once directly comparable data were obtained, we compared the S50 values of siPREP1 and siLuc control cells in order to measure changes of replication timing and to assess their statistical significance. The genome was divided into 250 Kb sliding windows, with 200 Kb overlap, which means that each 250 Kb window is made up of five adjacent 50 Kb intervals, and thus is characterized by five S50 values; for each window, the S50 values distributions for siPREP and siLuc were tested by a paired one-tail Student’s T-test for two alternative hypotheses: Late-to-Early (LtoE, when S50siPREP < S50siLUC,) or Early-to Late (EtoL, when S50siPREP > S50siLUC) shift in Replication Timing. Moreover, p-values were adjusted applying a Holm-Bonferroni correction and only windows with a corrected p-value ≤ 0.05 were used. This procedure allows the identification of genomic regions whose replication has been significantly shifted to earlier or later S-phase. Two or more adjacent 250 Kb windows that were coherently characterized by a statistically significant LtoE or EtoL shift were merged in LtoE or EtoL contigs.

The significance of the overlaps between ChIP-seq, RNA-seq or Lamin B1- associated DNA sequences was calculated with a Chi square test. The significance of overlaps between Lamin B1, ChIP-seq peaks or RNA-seq transcripts, a the LtoE or EtoL shifts was assessed by comparing the former with random genomic regions of the same size and distribution, iterated 100 times. Lamin B1 bound sequences^14^ were downloaded from the UCSC Genome Browser: NKI LaminB1 DamID Map, Tig3 cells, row counts 2,859,884, last updated 2010.

TTR^22^ were identified with an ad hoc developed script based on the presence, along the S50 profile, of i) an “inflection” point immediately following a “maximum” (or vice versa) showing absolute “ΔS50” value > 0.14, or ii) an “inflection” point immediately following another “inflection” point showing “ΔS50” value > 0.25. Late Replicating Regions (LRR) are instead those that have an S50 value > 0.65.

### ChIP-seq

Chromatin immunoprecipitations (IP) on HeLa cells were performed using standard methods^24,25^ with anti-Prep1 N15 antibody (sc-6245, Santa Cruz Biotechnology, Santa Cruz, USA). For each IP we used 20 μg antibody. IP with rabbit IgG was performed as negative control. Immunoprecipitated complexes were eluted from the beads by incubation for 30 min in EB (2% SDS in TE buffer) at 65 °C. The eluted material was reverse cross-linked at 65 °C overnight and incubated for 1 h at 55 °C with proteinase K. The DNA was purified with a PCR purification kit (Qiagen, Netherlands). About 10 ng of immunoprecipitated DNA were processed for sequencing. After library preparation DNA was sequenced using an Ion-Torrent Ion Proton P1 system.

After eliminating artifacts with FASTX-Toolkit v.0.0.13.2, reads were aligned to the hg19 genome using Bowtie v.0.12.8, allowing up to two mismatches per read and discarding reads with more than one mapping. Duplicate reads were removed using Samtool rmdup v 0.1.18. To identify enriched domains we used MACS version 2.0.10.20131028 with default parameters^35^, except for the p-value threshold that was set at p < 10^−5^.

### RNA-seq

For RNA-seq, three biological replicas of siLuc and siPREP1 HeLa cells were harvested 48 hours after transfection. Total RNA was purified^13^ and the library prepared. The sequencing was performed using Ion-Torrent Ion Proton P1 system. After eliminating artifacts with FASTX-Toolkit v.0.0.13.2, reads were aligned to the hg19 reference genome using Tophat v2.0.9. Then GenomicFeatures, Genomic Ranges and GenomicAligments R libraries were used to count the number of reads with respect to the annotation reference. Differentially expressed genes in siLuc v. siPREP1 treated cells were retrieved using DESeq2 R library^36^, filtering the results using a padj < 0.1 as a threshold. Gene ontology analysis was performed using Gorilla software^37^. Intersects between ChIPseq and RNAseq data were performed with the systems available in the Galaxy platform.

### Quantitative Real-Time PCR Analysis of replicated DNA

Essentially the same protocol for Repliseq was followed except that, after BrdU incorporation, the cells were fractionated into five sub-populations (S1 to S5). Flow-cytometry sorted cells were lysed and the BrdU-immunoprecipitated DNA amplified using specific primers (see Supplementary Table S4). Each reaction was performed in triplicate. Results were normalized on mitochondrial DNA^38^ with the specific primers MTR_F = CACACCCACCCAAGAACAG and MTR_R = AGTTTTAAGTTTTATGCGATTACCG. We used the ΔΔCT method to normalize the qPCR data to mitochondrial DNA Real-time PCRs were carried out on the Light Cycler480 (Hoffmann La Roche, Basel, Switzerland)^39^.

### Data accessibility

The raw Repli-seq, ChIP-seq and RNA-seq data are available from GEO, GSE101776.

## Electronic supplementary material

Supplementary Material

## References

[1] Jackson, S. P. & Bartek, J. The DNA-damage response in human biology and disease. *Nature.* **461**, 1071–1078 (2009). 10.1038/nature08467 PMC2906700 19847258

[2] Cornacchia D (2012). Mouse Rif1 is a key regulator of the replication-timing programme in mammalian cells. EMBO J..

[3] Foti R (2016). Nuclear Architecture Organized by Rif1 Underpins the Replication-Timing Program. Mol Cell..

[4] Gilbert, D. M. Temporal order of replication of Xenopus laevis 5S ribosomal RNA genes in somatic cells. *Proc. Natl. Acad. Sci. USA* **83**, 2924–2928 (1986). 10.1073/pnas.83.9.2924 PMC323419 3458252

[5] Berthelsen J, Zappavigna V, Mavilio F, Blasi F (1998). Prep1, a novel functional partner of Pbx proteins. EMBO J.

[6] Blasi, F., Bruckmann, C., Penkov, D. & Dardaei, L. A tale of TALE, PREP1, PBX1, and MEIS1: Interconnections and competition in cancer. Bioessays. **39**, 10.1002/bies.201600245 (2017). 10.1002/bies.201600245 28322463

[7] Selleri L (2001). Requirement for Pbx1 in skeletal patterning and programming chondrocyte proliferation and differentiation. Development..

[8] Azcoitia V, Aracil M, Martínez AC, Torres M (2005). The homeodomain protein Meis1 is essential for definitive hematopoiesis and vascular patterning in the mouse embryo. Dev Biol..

[9] Fernandez-Diaz LC (2010). The absence of *Prep1* causes p53-dependent apoptosis of pluripotent epiblast cells. Development.

[10] Ferretti E (2006). Hypomorphic mutation of the TALE gene *Prep1* (*pKnox1*) causes a major reduction of Pbx and Meis proteins and a pleiotropic embryonic phenotype. Mol. Cell. Biol..

[11] Longobardi E (2010). The homeodomain transcription factor *Prep1* gene *(pKnox1)* is a haploinsufficient tumor suppressor in man and mice. Molec Oncol.

[12] Iotti G (2011). The Homeodomain Transcription Factor Prep1 is required to maintain Genomic Stability. Proc Natl Acad Sci USA.

[13] Dardaei L, Longobardi E, Blasi F (2014). Prep1 and Meis1 competition for Pbx1 binding regulates protein stability and tumorigenesis. PNAS Plus..

[14] Guelen L (2008). Domain organization of human chromosomes revealed by mapping of nuclear lamina interactions. Nature..

[15] Michalet X (1997). Dynamic molecular combing: stretching the whole human genome for high-resolution studies. Science.

[16] Piunti A (2014). Polycomb proteins control proliferation and transformation independently of cell cycle checkpoints by regulating DNA replication. Nature Commun..

[17] Bianco JN (2012). Analysis of DNA replication profiles in budding yeast and mammalian cells using DNA combing. Methods..

[18] Técher H (2013). Replication dynamics: biases and robustness of DNA fiber analysis. J Mol Biol..

[19] Hansen RS (2010). Sequencing newly replicated DNA reveals widespread plasticity in human replication timing. Proc Natl Acad Sci USA.

[20] Chen CL (2010). Impact of replication timing on non-CpG and CpG substitution rates in mammalian genomes. Genome Res.

[21] Dellino GI (2013). Genome-wide mapping of human DNA-replication origins: levels of transcription at ORC1 sites regulate origin selection and replication timing. Genome Res..

[22] Pope BD (2014). Topologically associating domains are stable units of replication-timing regulation. Nature.

[23] Guan Z (2009). Decreased replication origin activity in temporal transition regions. J. Cell Biol..

[24] Penkov D (2013). Analysis of the *in vivo* DNA-binding profile and function of TALE homeoproteins reveals their specialization and differential interactions with Hox genes and proteins. Cell Reports.

[25] Laurent A (2015). ChIP-Seq and RNA-Seq analyses identify components of the Wnt and Fgf signaling pathways as Prep1 target genes in mouse embryonic stem cells. PLoS One..

[26] Dardaei L (2015). Meis1 overexpression causes a change of DNA target-sequence specificity which allows binding to the AP-1 element. Oncotarget..

[27] Dechat T (2008). Nuclear lamins: major factors in the structural organization and function of the nucleus and chromatin. Genes Dev.

[28] Shah PP (2013). Lamin B1 depletion in senescent cells triggers large-scale changes in gene expression and the chromatin landscape. Genes and Dev.

[29] Klymenko, T., *et al*. Lamin B1 regulates somatic mutations and progression of B-cell malignancies. *Leukemia*, 1‒12, 10.1038/leu.2017.255 (2017). 10.1038/leu.2017.255 PMC5808072 28804121

[30] Solovei I, Thanisch K, Feodorova Y (2016). How to rule the nucleus: divide et impera. Curr Opin Cell Biol.

[31] Bermejo R, Lai MS, Foiani M (2012). Preventing replication stress to maintain genome stability: resolving conflicts between replication and transcription. Mol Cell..

[32] Palumbo E, Matricardi L, Tosoni E, Bensimon A, Russo A (2010). Replication dynamics at common fragile site FRA6E. Chromosoma..

[33] Li H, Durbin R (2010). Fast and accurate long-read alignment with Burrows-Wheeler transform. Bioinformatics..

[34] Robinson MD, Oshlack A (2010). A scaling normalization method for differential expression analysis of RNA-seq data. Genome Biology.

[35] Ramírez F, Dündar F, Diehl S, Grüning BA, Manke T (2014). deepTools: a flexible platform for exploring deep-sequencing data. Nucleic Acids Res..

[36] Eden E, Navon R, Steinfeld I, Lipson D, Yakhini Z (2009). GOrilla: a tool for discovery and visualization of enriched GOterms in ranked gene lists. BMC Bioinformatics..

[37] Zhang Y (2008). Model-based Analysis of ChIP-seq (MACS). Genome Biol..

[38] Strehl S, LaSalle JM, Lalande M (1997). High-resolution analysis of DNA replication domain organization across an R/G-band boundary. Mol Cell Biol..

[39] Anders S, Huber W (2010). Differential expression analysis for sequence count data. Genome Biol.

