# Supplementary material for: PREP1 tumor suppressor protects the late-replicating DNA by controlling its replication timing and symmetry
Source: Sci Rep. 2018 Feb 16;8:3198. doi: 10.1038/s41598-018-21363-4 (PMC5816642; doi:10.1038/s41598-018-21363-4)
Supplement: Supplementary file 1 — Supplementary Material [file 41598_2018_21363_MOESM1_ESM.pdf]

PREP1 tumor suppressor protects the late-replicating DNA by controlling its replication timing and symmetry.

Angela Palmigiano[1&#], Francesco Santaniello[2#], Aurora Cerutti[1$], Dmitry Penkov[1,3], Divya Purushothaman[1], Ekta Makhija[4], Lucilla Luzi[2], Fabrizio d'Adda di Fagagna[1,5], Pier Giuseppe Pelicci[2,6], Viweswara Shivashankar[1,4], Gaetano Ivan Dellino[2,5*] and Francesco Blasi[1*]

[1]IFOM (Foundation FIRC Institute of Molecular Oncology) and [2]Department of Experimental Oncology, European Institute of Oncology, via Adamello 16, 20139 Milan, Italy; [3]Lomonosov Moscow State University, Leninskiye Gori 1, 119991 Moscow, Russia; [4]Mechano-Biology Institute, National University of Singapore, Singapore; [5]Istituto di Genetica Molecolare, Consiglio Nazionale delle Ricerche (IGM-CNR), Via Abbiategrasso 207, 27100 Pavia, Italy; [6]Department of Oncology and Hemato-Oncology, University of Milan, Via Santa Sofia 9, 20142 Milan, Italy.

SUPPLEMENTARY MATERIAL

Supplementary Figures.
- Figure S1
- Figure S2
- Figure S3
- Figure S4
- Figure S5
- Figure S6
- Figure S7

Supplementary Tables
- Table S1
- Table S2
- Table S3
- Table S4
- Table S5
- Table S6
- Table S7
- Table S8
- Table S9

Full length blots
- To Figure 1A
- To Figure 2F (two blots: anti-γH2Ax and anti-αTubulin)
- To Figure 6 (three blots: anti-PREP1, anti-Lamin B1 and anti-Tubulin).For each two siRNA transfection times are tested: 24 and 48 h. In Figure &, only the 48 h transfection time is reproduced.

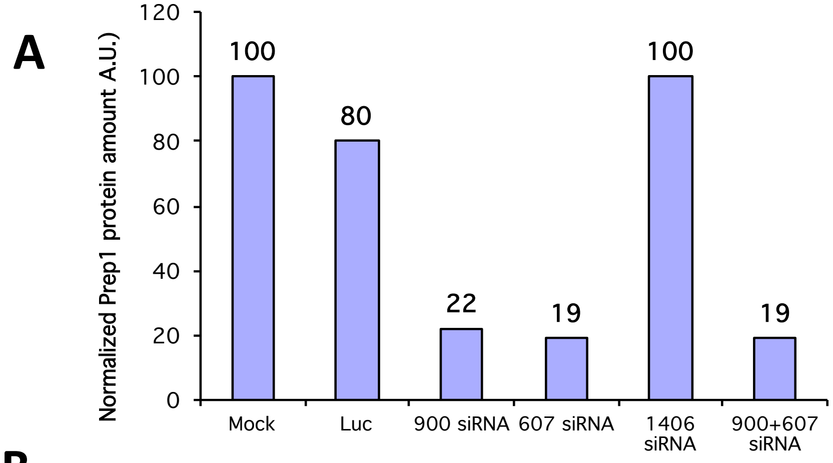

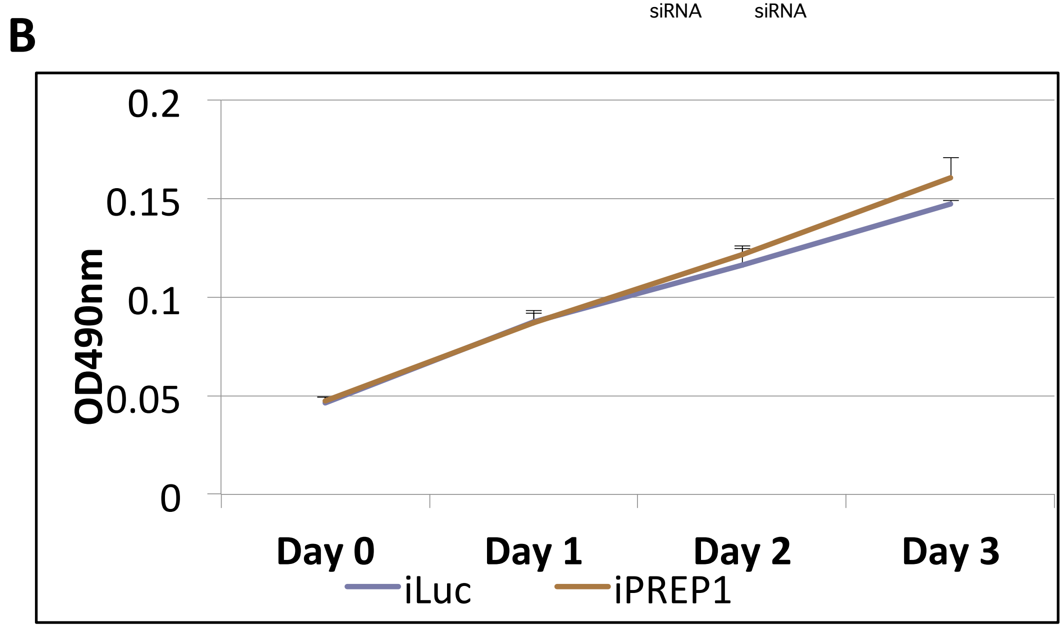

Supplemental Figure S1. Quantification (**A**) of the effect of three PREP1 siRNAs in HeLa cells by immunobloting with anti-PREP1 antibodies (not shown) of protein extracts prepared 48 hrs after transfection. Luciferase (Luc) siRNA was used as control. **B**. Down regulation of PREP1 has almost no effect on cells growth.

Note: Transfection of HeLa cells with the combination of “607” and “900” siRNAs did not induce apoptosis beyond that of the Mock transfection itself (18% at 48hrs, as measured by Annexin V positivity in flow cytometry) (data not shown).

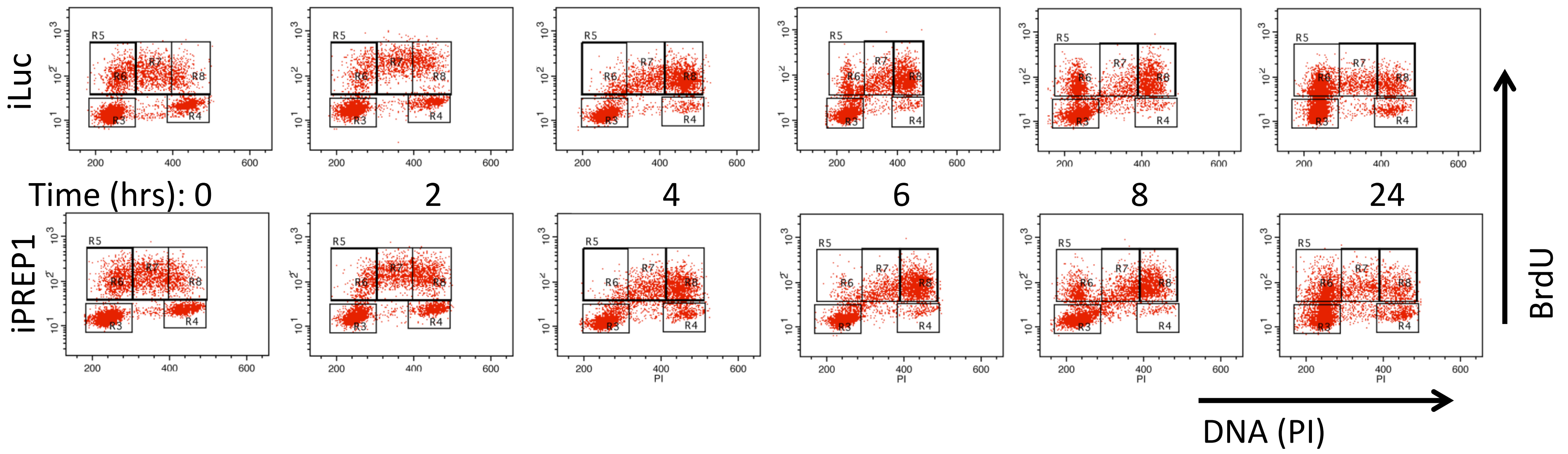

Supplementary Figure S2A. Example of cytofluorimetric analysis (BrdU vs. PI) of HeLa cells transfected with PREP1-specific 600+900 (bottom) or Luc (top) siRNA , at various hrs (indicated) after the BrdU pulse (see **Methods**). The rectangle R5 identifies the entire S phase. Rectangles R6, R7 and R8 identify the early, mid and late S phase, respectively.

B

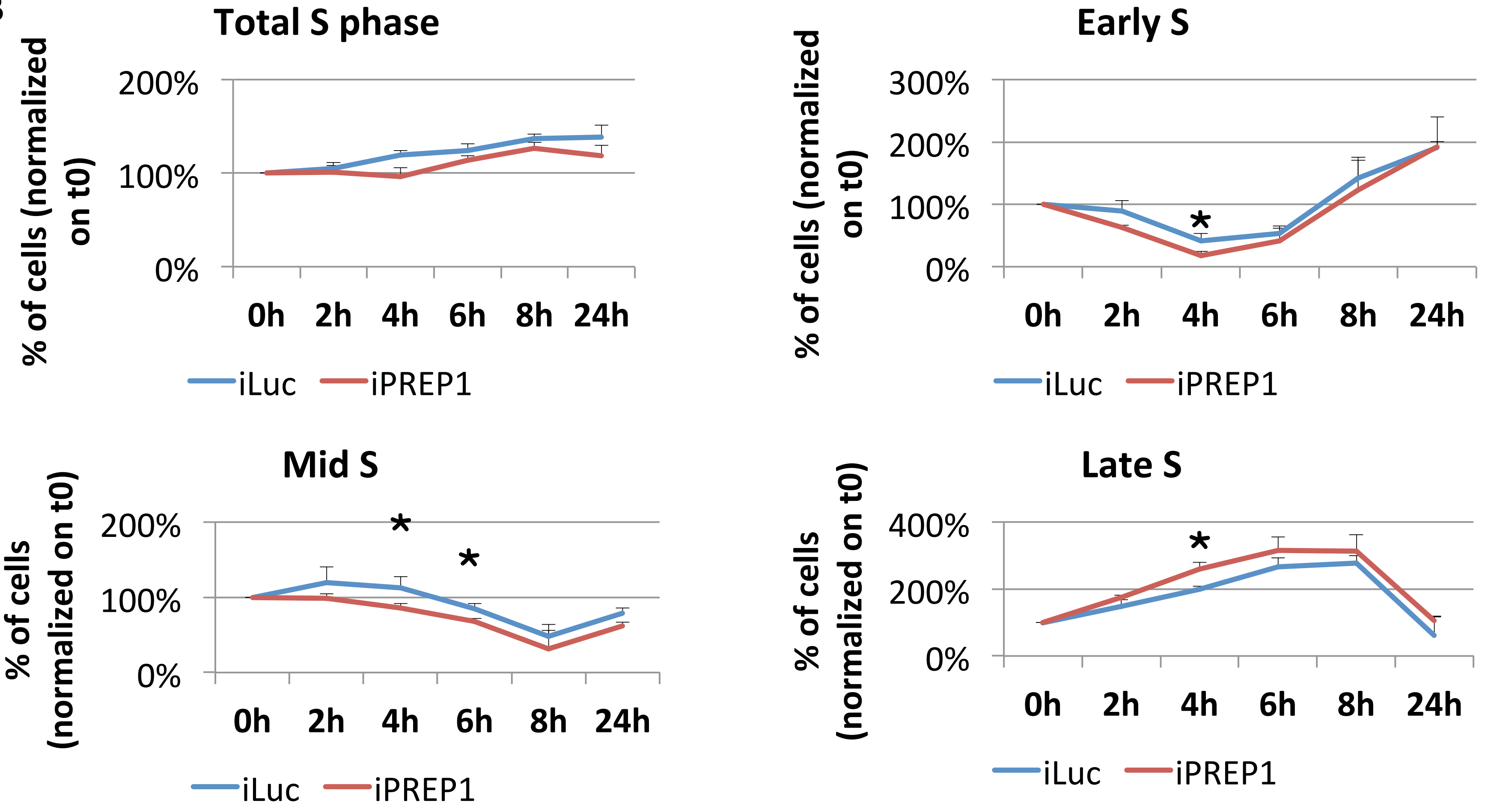

Supplementary Figure S2B. Cytofluorimetric analysis of the S phase of HeLa cells treated with PREP1-specific 600 or Luc siRNA. Cells were pulse-labeled with BrdU, imaged by flow-cytometry with BrdU antibodies and the number of cells in the various fractions of the S phase counted at various times thereafter. The data are the average of three independent experiments. The asterisk indicates the p-values (Student's T test): 0,0203 (4 hrs Early S), 0,0435 (4 hrs Mid S) 0,0324 (6 hrs Mid S), and 0,0172 (4 hrs Late S). The same number of cells was used in all cases.

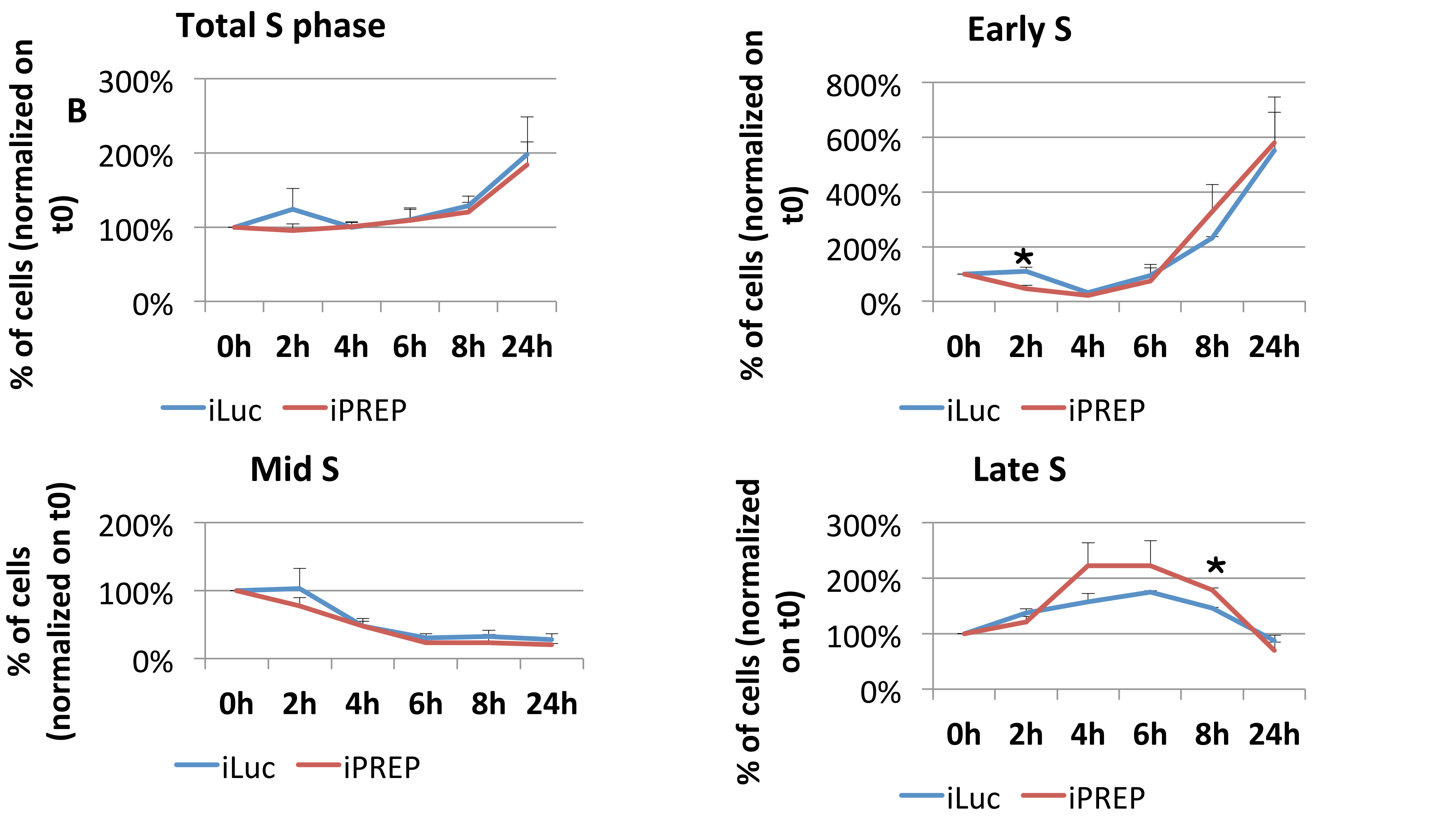

Supplementary Figure S2C. Cytofluori metric analysis of the S phase of human BJ fibroblasts treated with PREP1-specific 600 or Luc siRNA. Cells were pulse-labeled with BrdU, gated by cytofluorimetry and the number of cells in the various fractions of the S phase counted at various times thereafter. The data are the average of three independent experiments. P-values were calculated by the Student's T test and were P=0,0111 (2 hrs Early S) and 0,00716 (8 hrs Late S). The same number of cells was used in all cases.

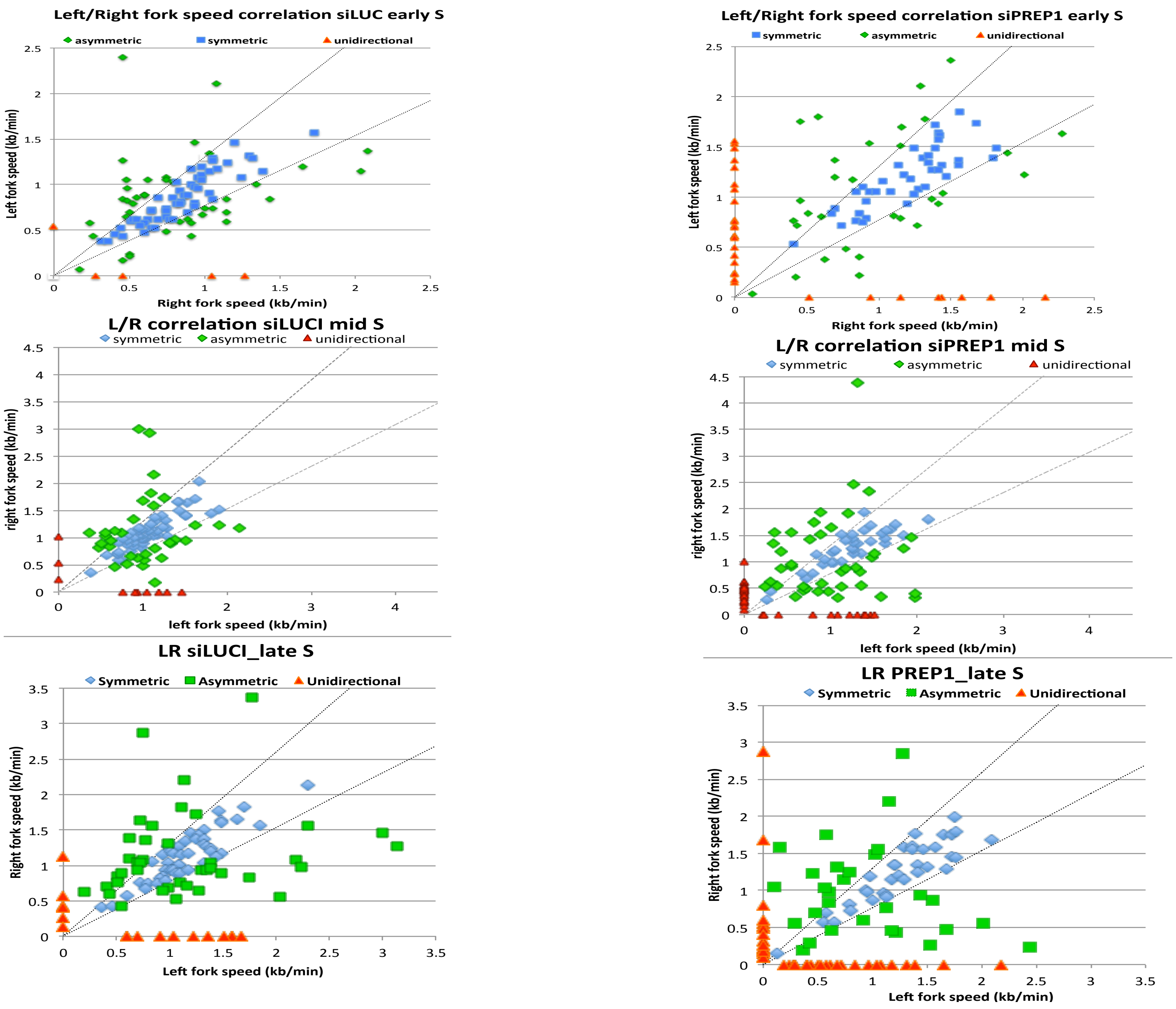

Supplemental Figure S3. Actual measure of the fork symmetry (see Methods) in the three Sub-populations (early-S, mid-S and late-S) of siLuc and siPREP1 cells.

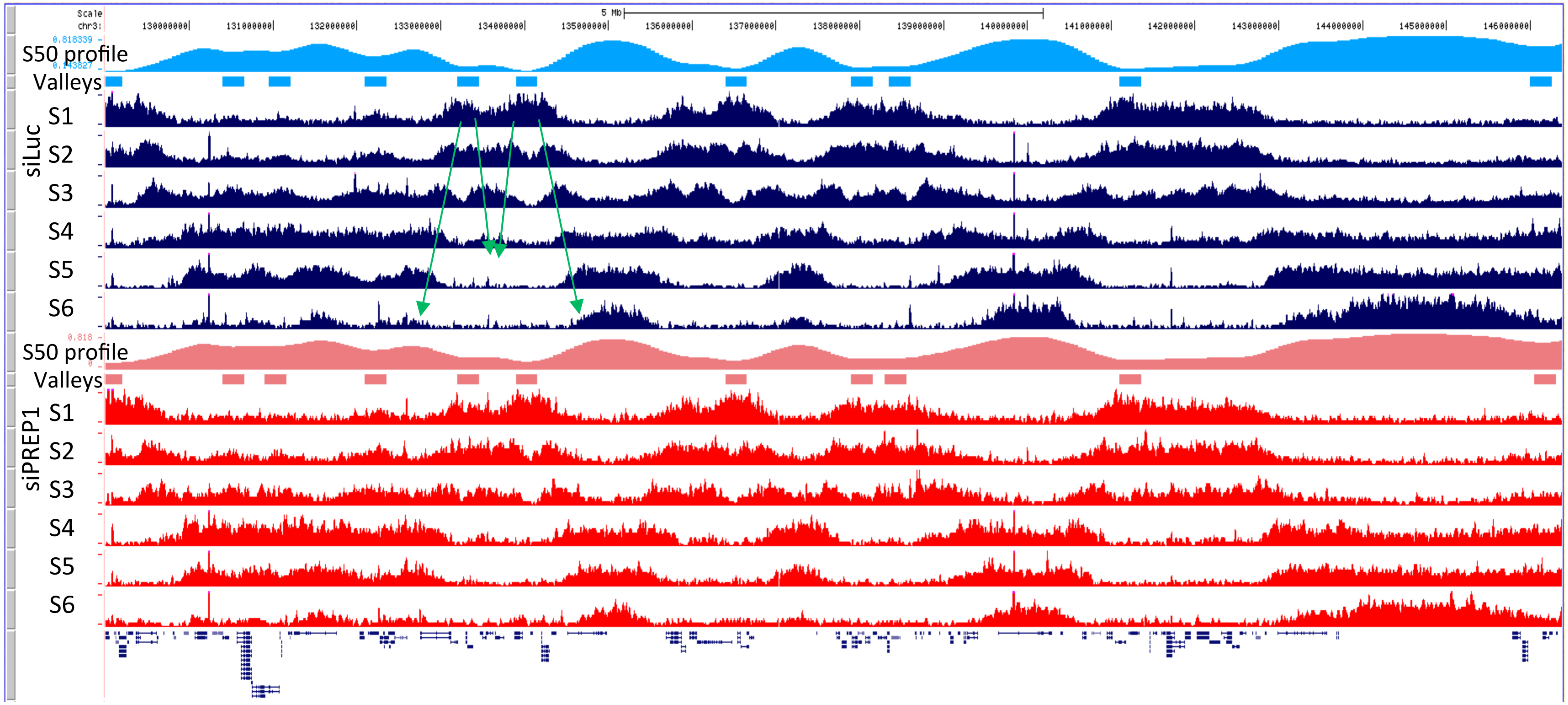

Supplementary Figure S4.

Repli-seq profiles of siLuc and siPREP1 cells. Representative genomic region (17 Mb in length) from UCSC Genome Browser showing replication profiles of siLuc and siPREP1 cells. BrdU-positive regions of siLuc subpopulations (S1 to S6, blue); BrdU-positive regions of siPREP1 subpopulations (S1 to S6, red); S50 profiles and S50 valleys (boxes), in light blue for siLuc and in light red for siPREP1 subpopulations; inverted-Vs (green arrows), as indicated.

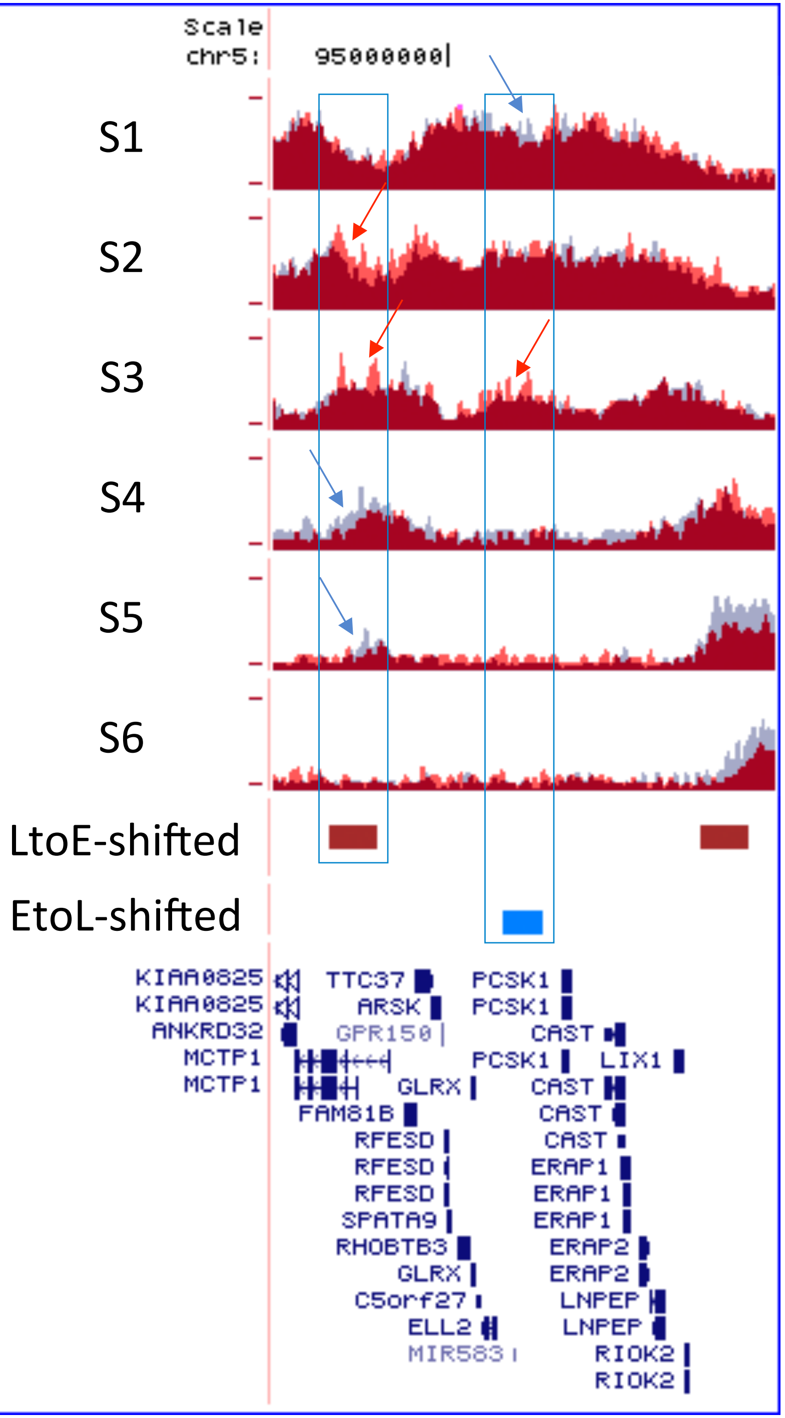

Supplementary Figure S5. Temporally-shifted regions upon Prep1 down-regulation.

Repliseq profiles (S1, early-, to S6, late-S phase) of siLuc cells (pale blue tracks) and siPrep1 (superimposed red tracks) showing representative late-to-early (LtoE)- and early-to-late (EtoL)-shifted regions identified by visual inspection (empty blue boxes).

Red and blue arrows within the empty blue boxes indicate genomic regions showing higher levels of BrdU incorporation within siPrep1 or siLuc cells, respectively, in different S-phase windows, as indicated. Red and blue boxes indicate LtoE- and EtoL-shifted regions, respectively, identified by bioinformatic analysis.

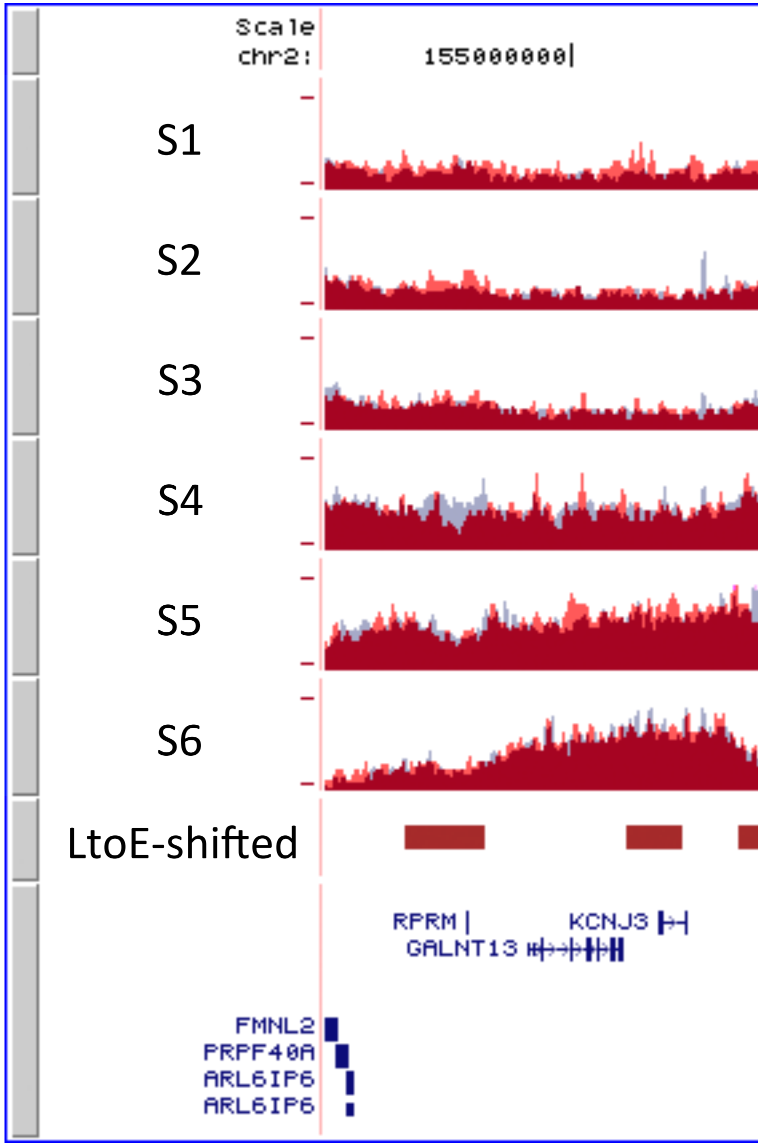

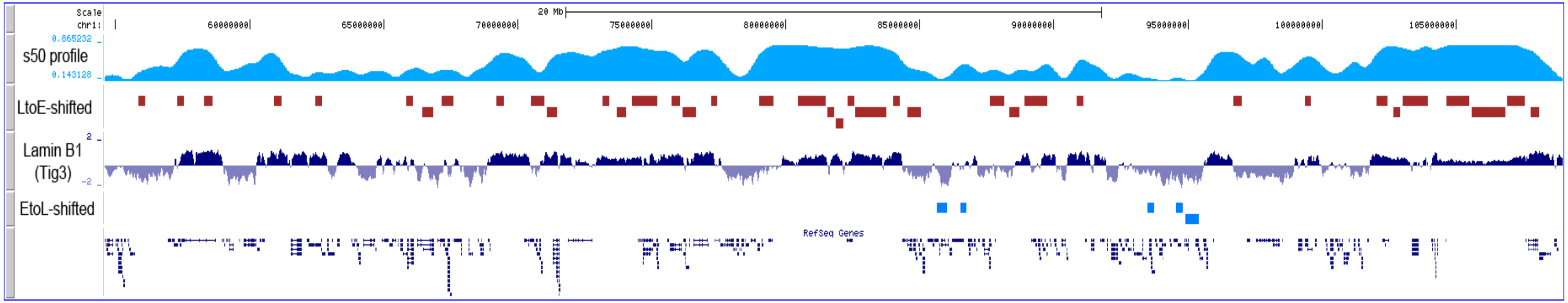

Supplementary Figure S6.

Mapping of temporally-shifted regions in siPrep1 cells. Genomic region (55 Mb in length) from human chromosome 1, showing (top to bottom): the S50 profile (light blue track); LtoE-shifted regions identified in siPrep1 cells (red boxes); Lamin B1-bound (deep blue signals corresponding to positive log2-ratio scores (14)); EtoL-shifted regions identified in siPREP1 cells (dark blue boxes); position of RefSeq genes, as indicated.

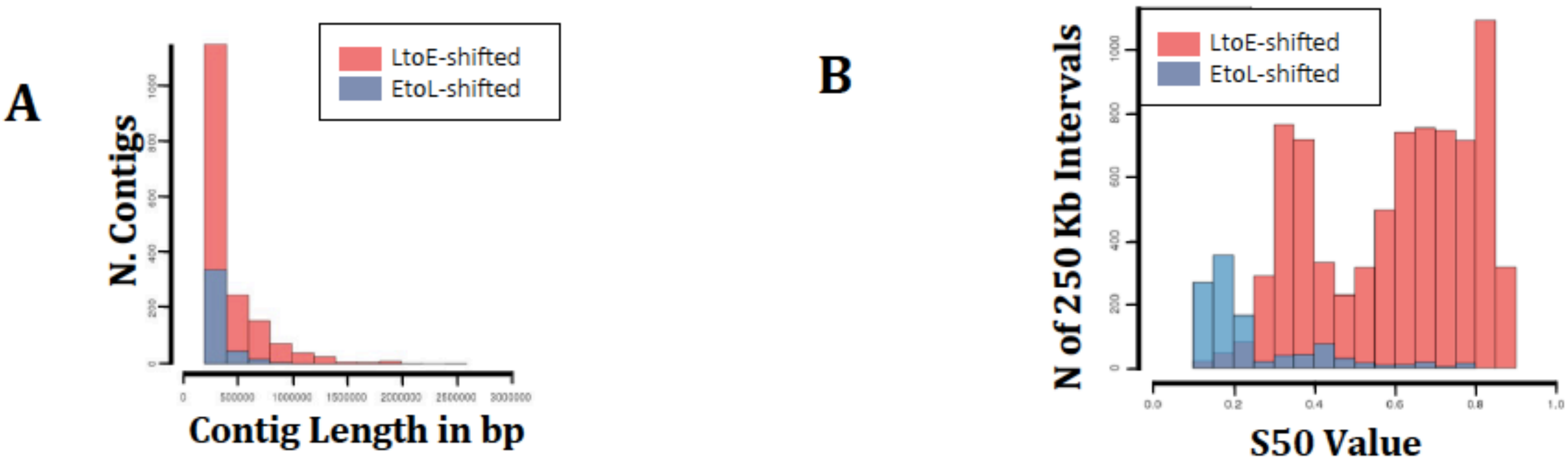

Supplementary Figure S7. Characterization of LtoE and EtoL shifted contigs.
A: Length distribution of LtoE (red) and EtoL (blue) contigs. LtoE and EtoL contigs consist mostly of 250Kb-long intervals, but can be as long as 2.5Mb.
B: In siLuc cellsLtoE shifted regions (red) derive from mid- and late-S phase (S50=0.3-0.4 and 0.6-0.8) . EtoL (blue) shifts derive early- mid-S phase (S50=0.15-0.4).

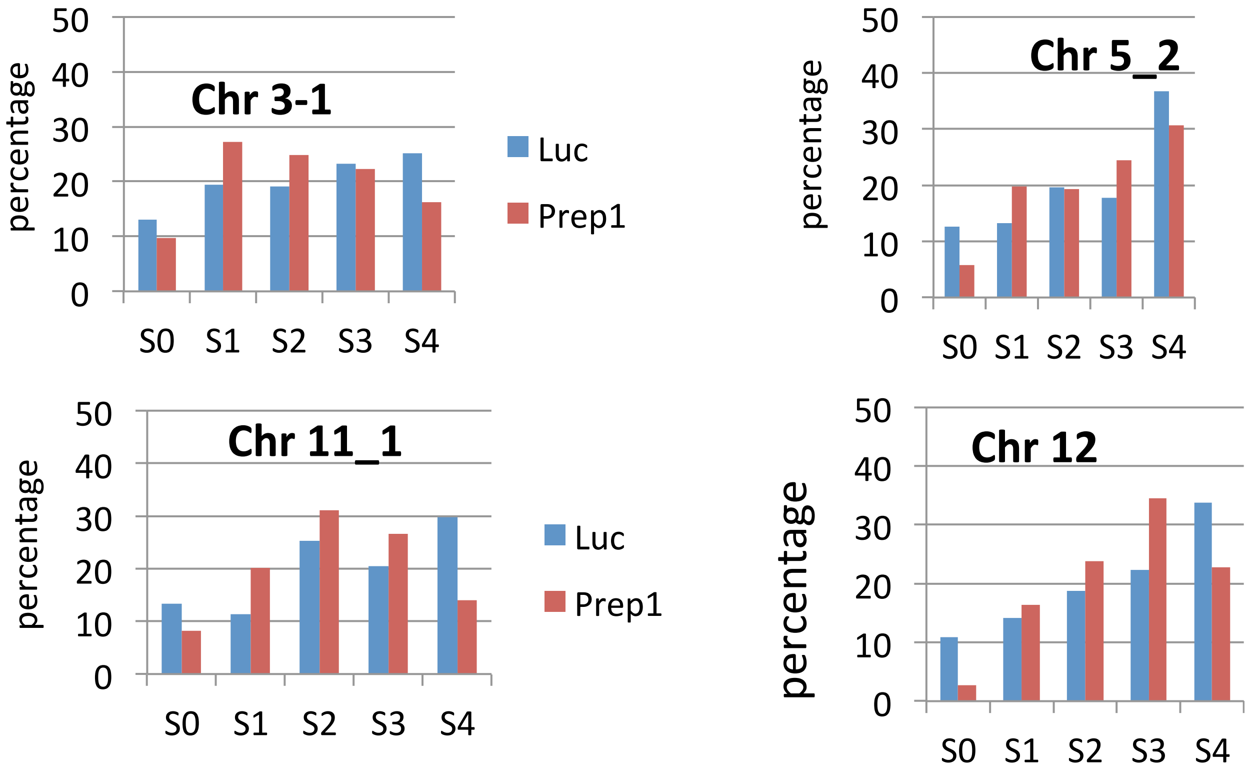

Supplementary Figure S8. Validation of LtoE regions by qPCR.

Supplementary Figure S8. Validation of LtoE-shifted regions by qPCR. HeLa cells were transfected with siLuc (control) or siPREP1, sorted by flow cytometry into five subpopulations of increasing DNA Content (S0 to S4). The immunoprecipitated DNA of each fraction was measured by qPCR with specific primer sets (listed in **Supplementary Table S4**). Data are normalized to mitochondrial DNA content and expressed as percent of total. S windows showing stronger signals in siPREP1 precede those with stronger signals in siLuc.

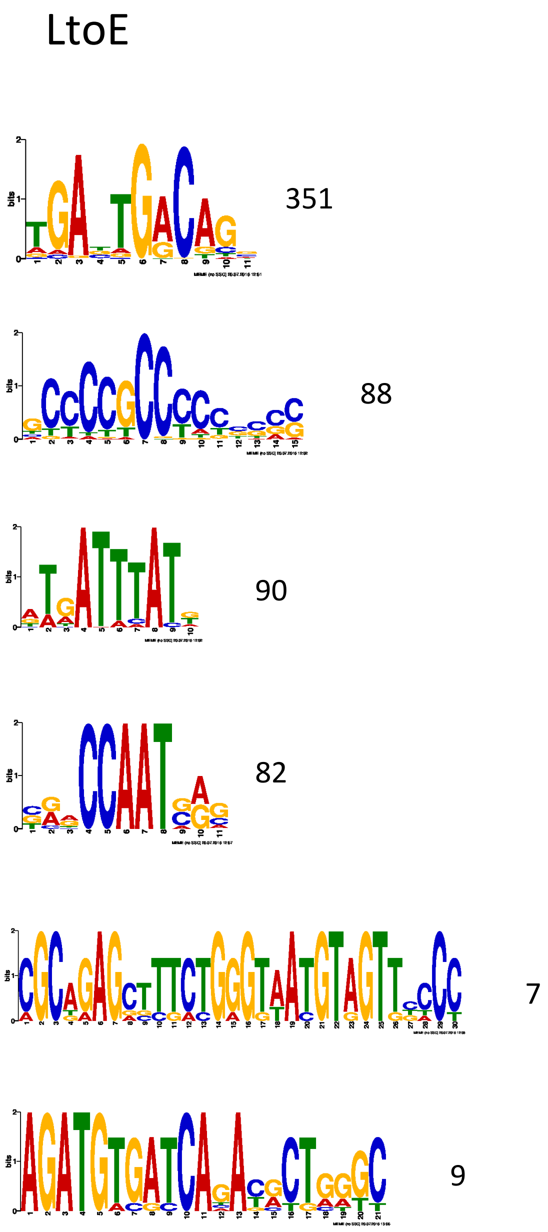

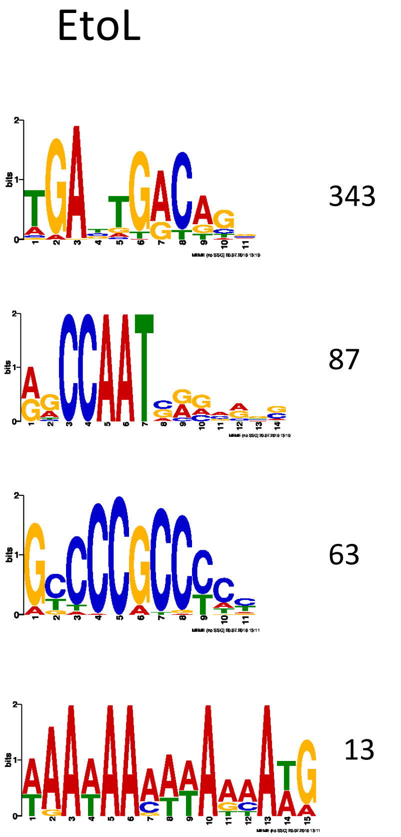

Supplementary Figure S9. Consensus sequences Bound by PREP1 in HeLa cells.

Supplementary Figure S10: Raw data: number of reads after pre-processing and alignment on Hg19

Control Condition

Hela_Blasi_iLuc_BrDUS1_120905_Hg19
tot_after_filter_illumina 32,160,807 79.08%
mapped 3,0371,675 74.68%
stdchr 27,737,863 68.20%
nodup 15,419,546 37.91%

Hela_Blasi_iLuc_BrDUS2_120905_Hg19
tot_after_filter_illumina 40,309,904 86.48%
mapped 38,708,476 83.04%
stdchr 35,305,302 75.74%
nodup 21,007,615 45.07%

Hela_Blasi_iLuc_BrDUS3_120905_Hg19
tot_after_filter_illumina 53,505,289 86.26%
mapped 51,658,321 83.28%
stdchr 46,891,306 75.60%
nodup 31,886,429 51.41%

Hela_Blasi_iLuc_BrDUS4_120905_Hg19
tot_after_filter_illumina 30,236,925 86.84%
mapped 28,613,066 82.17%
stdchr 25,604,856 73.53%
nodup 14,303,190 41.08%

Hela_Blasi_iLuc_BrDUS5_120905_Hg19
tot_after_filter_illumina 32,135,690 86.85%
mapped 30,090,459 81.32%
stdchr 26,278,144 71.02%
nodup 14,246,886 38.50%

Hela_Blasi_iLuc_BrDUS6_120905_Hg19
tot_after_filter_illumina 37,117,765 86.35%
mapped 33,351,964 77.59%
stdchr 28,880,082 67.19%
nodup 7,820,162 18.19%

Experimental Condition

Hela_Blasi_iPREP1_BrDUS1_120905_Hg19
tot_after_filter_illumina 33,870,008 78.87%
mapped 30,841,416 71.82%
stdchr 28,039,777 65.29%
nodup 10,526,141 24.51%

Hela_Blasi_iPREP1_BrDUS2_120905_Hg19
tot_after_filter_illumina 32,903,807 79.16%
mapped 30,875,801 74.28%
stdchr 28,068,857 67.53%
nodup 9,199,006 22.13%

Hela_Blasi_iPREP1_BrDUS3_120905_Hg19
tot_after_filter_illumina 30,228,235 79.74%
mapped 28,200,782 74.39%
stdchr 25,538,444 67.37%
nodup 7,543,725 19.90%

Hela_Blasi_iPREP1_BrDUS4_120905_Hg19
tot_after_filter_illumina 34,257,938 92.85%
mapped 30,863,575 83.65%
stdchr 27,373,228 74.19%
nodup 7,659,340 20.76%

Hela_Blasi_iPREP1_BrDUS5_120905_Hg19
tot_after_filter_illumina 4,4395,115 92.82%
mapped 39,961,617 83.55%
stdchr 35,140,338 73.47%
nodup 8,948,582 18.71%

Hela_Blasi_iPREP1_BrDUS6_120905_Hg19
tot_after_filter_illumina 42,835,055 92.85%
mapped 38,356,240 83.14%
stdchr 33,606,445 72.85%
nodup 6,874,330 14.90%

Supplementary Table S1

Example of determination of the statistical significance of the LtoE and EtoL shifts.

	LtoE shifted	EtoL shifted
Chromosome	13	5
Window Start	76500000	95350000
Window End	76750000	95600000
siPREP1 S50 values	0,419; 0,455; 0,526; 0,491; 0,384	0,218; 0,227; 0,227; 0,225; 0,224
siLuc S50 values	0,521; 0,557; 0,621; 0,591; 0,483	0,195; 0,202; 0,203; 0,201; 0,199
t_test_LtoE	5,77E-^{08}	0,999999869
t_test_EtoL	0,999999942	1,31E-^{07}
t_test_LtoE_padj	0,00318493	1
t-test_EtoL_padj	1	0,007533959
Max delta S50	-0,102	0,025

Genomic coordinates of the 250 kb windows shown in **Figure 3** (LtoE-shifted) and **Supplementary Figure S5** (EtoL-shifted); S50 values of the five 50-Kb windows therein; statistical significance, and highest delta S50 value (=siPREP1 S50 - siLuc S50) measured between corresponding 50 kb windows (Max delta S50).

Supplementary Table S2
List and coordinates of PREP1 DR induced late-to-early (LtoE) shifted genomic windows.

Chr	Start	End	Adj_Pvalue_LtoE	Adj_Pvalue_EtoL	Diff_S50
chr1	1450000	1700000	0,003829995	1	-0,010421404
chr1	1500000	1750000	0,015267012	1	-0,009817551
chr1	4300000	4550000	0,003703453	1	-0,049600917
chr1	4350000	4600000	0,008014744	1	-0,049600917
chr1	4400000	4650000	0,03986091	1	-0,049600917
chr1	4500000	4750000	0,043150173	1	-0,048633035
chr1	4550000	4800000	0,015725346	1	-0,046714691
chr1	4600000	4850000	0,003696235	1	-0,041958053
chr1	4650000	4900000	0,001361958	1	-0,041257152
chr1	4700000	4950000	0,002980184	1	-0,040080356
chr1	4750000	5000000	0,015226211	1	-0,038398576

chr1	5250000	5500000	0,014574973	1	-0,02684797
chr1	5300000	5550000	0,00378223	1	-0,025792626
chr1	5350000	5600000	0,004852082	1	-0,025078228
chr1	5400000	5650000	0,040118564	1	-0,024660586
chr1	7200000	7450000	0,035609915	1	-0,053543498
chr1	7250000	7500000	0,018897085	1	-0,057892051
chr1	14050000	14300000	0,02431762	1	-0,028824192
chr1	14100000	14350000	0,006005625	1	-0,027341222
chr1	14850000	15100000	0,01177731	1	-0,02695083
chr1	17050000	17300000	0,010429506	1	-0,007956807
chr1	26550000	26800000	0,029874066	1	-0,000764009
chr1	26600000	26850000	0,020420268	1	-0,000856728
chr1	30150000	30400000	0,034138751	1	-0,041464476
chr1	30200000	30450000	0,00564964	1	-0,041577626
chr1	30250000	30500000	0,000402348	1	-0,041518479

chr1	30300000	30550000	6,95E-06	1	-0,041518479
chr1	30350000	30600000	1,28E-08	1	-0,041518479
chr1	30400000	30650000	1,87E-07	1	-0,041518479
chr1	30450000	30700000	1,26E-06	1	-0,041518479
chr1	30500000	30750000	9,82E-07	1	-0,041470454
chr1	30550000	30800000	8,76E-05	1	-0,041544767
chr1	30600000	30850000	0,005014257	1	-0,041720799
chr1	34200000	34450000	0,008216622	1	-0,042278513
chr1	34250000	34500000	0,000492061	1	-0,041276201
chr1	34300000	34550000	0,003015596	1	-0,040197838
chr1	34350000	34600000	0,008793695	1	-0,039426132
chr1	34400000	34650000	0,006456305	1	-0,039426132
chr1	34450000	34700000	0,001079557	1	-0,039426132
chr1	34500000	34750000	0,000797808	1	-0,039426132

chr1	34550000	34800000	0,011103144	1	-0,039426132
chr1	34600000	34850000	0,038283224	1	-0,039263381
chr1	34650000	34900000	0,041159519	1	-0,03985288
chr1	34700000	34950000	0,013262942	1	-0,041124798
chr1	34750000	35000000	0,002038015	1	-0,042770679
chr1	34800000	35050000	0,027451224	1	-0,044291335
chr1	37350000	37600000	0,023147393	1	-0,023387015
chr1	38750000	39000000	0,047556248	1	-0,02064644
chr1	48100000	48350000	0,012537597	1	-0,019040469
chr1	48150000	48400000	0,038620068	1	-0,018178924
chr1	48250000	48500000	0,025738111	1	-0,018407406
chr1	48300000	48550000	0,021443863	1	-0,019890849
chr1	48700000	48950000	0,033235197	1	-0,033515323
chr1	48750000	49000000	0,004534547	1	-0,033694897
chr1	48800000	49050000	0,000389535	1	-0,03368928
chr1	48850000	49100000	1,45E-05	1	-

					0,033443744
chr1	48900000	49150000	6,56E-05	1	-0,032888576
chr1	48950000	49200000	0,001506432	1	-0,03199231
chr1	49000000	49250000	0,011091576	1	-0,030798312
chr1	49050000	49300000	0,041234365	1	-0,029432052
chr1	49250000	49500000	0,04450139	1	-0,02598273
chr1	49300000	49550000	0,007409592	1	-0,026118776
chr1	49350000	49600000	0,000305777	1	-0,026447922
chr1	49400000	49650000	8,77E-05	1	-0,02669178
chr1	49450000	49700000	0,00011909	1	-0,026578668
chr1	49500000	49750000	0,000144906	1	-0,026264242
chr1	49550000	49800000	0,00565358	1	-0,026023165
chr1	49850000	50100000	0,013109261	1	-0,021626949
chr1	50350000	50600000	0,003401271	1	-0,036450728
chr1	50400000	50650000	0,007810755	1	-0,038166744
chr1	50750000	51000000	0,038185025	1	-0,030094051

chr1	50800000	51050000	0,029726775	1	-0,028876637
chr1	50850000	51100000	0,043013812	1	-0,02798721
chr1	55850000	56100000	0,020503742	1	-0,021649575
chr1	57300000	57550000	0,009278629	1	-0,055473221
chr1	58300000	58550000	0,038537254	1	-0,032592131
chr1	58350000	58600000	0,023123711	1	-0,035362101
chr1	60900000	61150000	0,020278657	1	-0,042618471
chr1	60950000	61200000	0,008035183	1	-0,045241792
chr1	62450000	62700000	0,033223631	1	-0,021506524
chr1	65850000	66100000	0,04899806	1	-0,013387679
chr1	66450000	66700000	0,012343971	1	-0,033531594
chr1	66500000	66750000	0,000708836	1	-0,03554028
chr1	66550000	66800000	0,005783448	1	-0,036613858
chr1	66600000	66850000	0,026984415	1	-0,036804255
chr1	67150000	67400000	0,019361158	1	-0,021140124

chr1	67200000	67450000	0,003424789	1	- 0,021140124
chr1	67250000	67500000	0,000627798	1	- 0,021140124
chr1	67300000	67550000	0,001344731	1	- 0,020948109
chr1	67350000	67600000	0,024857136	1	- 0,020741632
chr1	69200000	69450000	0,007630088	1	- 0,026534524
chr1	69250000	69500000	0,01392445	1	- 0,025039683
chr1	70500000	70750000	0,030352729	1	- 0,034818414
chr1	70550000	70800000	0,00730433	1	- 0,036449711
chr1	70600000	70850000	0,001887232	1	-0,03775139
chr1	70650000	70900000	0,001936759	1	- 0,038642987
chr1	70700000	70950000	0,009121827	1	- 0,042681758
chr1	70750000	71000000	0,049766265	1	- 0,045101014
chr1	71100000	71350000	0,025165172	1	- 0,061670275
chr1	71150000	71400000	0,002288449	1	- 0,059346665
chr1	71200000	71450000	0,024775383	1	-

					0,055828963
chr1	73150000	73400000	0,035028671	1	- 0,015343029
chr1	73700000	73950000	0,030514295	1	- 0,008624382
chr1	73750000	74000000	0,002841695	1	- 0,008851833
chr1	73800000	74050000	0,006774317	1	- 0,009321005
chr1	74250000	74500000	0,024739854	1	- 0,013067031
chr1	74300000	74550000	0,008273973	1	- 0,013611098
chr1	74350000	74600000	0,002856834	1	- 0,014044659
chr1	74400000	74650000	0,001619179	1	- 0,014380778
chr1	74450000	74700000	0,002922122	1	- 0,014631782
chr1	74500000	74750000	0,011410089	1	- 0,014819582
chr1	74550000	74800000	0,04207943	1	- 0,014994111
chr1	74800000	75050000	0,038233252	1	- 0,017880067
chr1	74850000	75100000	0,015719168	1	- 0,018710204
chr1	74900000	75150000	0,011547555	1	-

					0,019392704
chr1	74950000	75200000	0,021174991	1	-0,01989953
chr1	75750000	76000000	0,012346477	1	-0,045357177
chr1	75800000	76050000	0,019564946	1	-0,048204472
chr1	76150000	76400000	0,037305161	1	-0,036078719
chr1	76350000	76600000	0,024944161	1	-0,038874933
chr1	76400000	76650000	0,031539089	1	-0,035809549
chr1	77200000	77450000	0,013312762	1	-0,033327313
chr1	79000000	79250000	0,020348472	1	-0,030112554
chr1	79050000	79300000	0,001831018	1	-0,030304138
chr1	79100000	79350000	0,000190998	1	-0,030411625
chr1	79150000	79400000	1,73E-05	1	-0,030288089
chr1	79200000	79450000	3,68E-05	1	-0,029754785
chr1	79250000	79500000	0,002226718	1	-0,028687705
chr1	79300000	79550000	0,031181659	1	-0,027079305

chr1	80450000	80700000	0,006897057	1	-0,015804285
chr1	80500000	80750000	0,002390271	1	-0,016742612
chr1	80550000	80800000	0,018751066	1	-0,017148052
chr1	80600000	80850000	0,04464032	1	-0,017080435
chr1	80650000	80900000	0,046639654	1	-0,016682352
chr1	80700000	80950000	0,024575805	1	-0,016116864
chr1	80750000	81000000	0,005554402	1	-0,015525623
chr1	80800000	81050000	0,000354337	1	-0,015011203
chr1	80850000	81100000	0,000232324	1	-0,014642193
chr1	80900000	81150000	0,001823407	1	-0,014461243
chr1	80950000	81200000	0,003599619	1	-0,014478817
chr1	81000000	81250000	0,002539348	1	-0,014667071
chr1	81050000	81300000	0,000797446	1	-0,01495519
chr1	81100000	81350000	0,000188541	1	-0,015250058
chr1	81150000	81400000	0,000335445	1	-

					0,015470253
chr1	81200000	81450000	0,003674023	1	-0,015589588
chr1	81250000	81500000	0,026952149	1	-0,015654932
chr1	81550000	81800000	0,044324089	1	-0,019331431
chr1	81850000	82100000	0,010874019	1	-0,024404797
chr1	81900000	82150000	0,01116351	1	-0,025660806
chr1	82300000	82550000	0,033508973	1	-0,01623186
chr1	82600000	82850000	0,012467828	1	-0,020471978
chr1	82650000	82900000	0,00047302	1	-0,020109311
chr1	82700000	82950000	0,000691584	1	-0,019696831
chr1	82750000	83000000	0,002588059	1	-0,019426899
chr1	82800000	83050000	0,001758807	1	-0,019460805
chr1	82850000	83100000	0,000309287	1	-0,019880727
chr1	82900000	83150000	0,002572359	1	-0,019880727
chr1	82950000	83200000	0,026263926	1	-0,019880727

chr1	83100000	83350000	0,045461836	1	-0,020642005
chr1	83150000	83400000	0,010211827	1	-0,021585779
chr1	83200000	83450000	0,000651703	1	-0,022491929
chr1	83250000	83500000	0,000107768	1	-0,023161233
chr1	83300000	83550000	0,000890599	1	-0,023489935
chr1	83350000	83600000	0,002160036	1	-0,023484957
chr1	83400000	83650000	0,001558256	1	-0,023236413
chr1	83450000	83700000	0,000271522	1	-0,02286283
chr1	83500000	83750000	0,001785935	1	-0,022467082
chr1	84000000	84250000	0,008174613	1	-0,042511526
chr1	84550000	84800000	0,009847607	1	-0,017978073
chr1	84600000	84850000	0,000138033	1	-0,016984098
chr1	84650000	84900000	0,000163168	1	-0,016571111
chr1	84700000	84950000	0,00010693	1	-0,016541485
chr1	84750000	85000000	0,000143956	1	-

					0,016711055
chr1	84800000	85050000	0,005387364	1	-0,016915498
chr1	87600000	87850000	0,013162466	1	-0,019848185
chr1	87650000	87900000	0,000198716	1	-0,019848185
chr1	87700000	87950000	2,25E-07	1	-0,019848185
chr1	87750000	88000000	2,09E-06	1	-0,019972646
chr1	87800000	88050000	0,000130681	1	-0,01993128
chr1	87850000	88100000	0,002093625	1	-0,019884567
chr1	87900000	88150000	0,015147311	1	-0,019924838
chr1	88350000	88600000	0,020518006	1	-0,033890411
chr1	88400000	88650000	0,001299716	1	-0,032957804
chr1	88450000	88700000	0,007563589	1	-0,031385808
chr1	88900000	89150000	0,018442969	1	-0,020478639
chr1	88950000	89200000	0,024849161	1	-0,019139916
chr1	89200000	89450000	0,041734247	1	-0,025041693

chr1	89250000	89500000	0,004253815	1	-0,024976163
chr1	89300000	89550000	0,000109515	1	-0,024776329
chr1	89350000	89600000	4,75E-05	1	-0,024463826
chr1	89400000	89650000	0,000673688	1	-0,023975083
chr1	89450000	89700000	0,004809751	1	-0,023201542
chr1	89500000	89750000	0,027937488	1	-0,022053373
chr1	90850000	91100000	0,029896971	1	-0,043743795
chr1	96700000	96950000	0,033392517	1	-0,03180939
chr1	96750000	97000000	0,010496071	1	-0,034108999
chr1	99350000	99600000	0,008395136	1	-0,023455367
chr1	102050000	102300000	0,047720826	1	-0,024606718
chr1	102100000	102350000	0,001684165	1	-0,024159384
chr1	102150000	102400000	0,001431889	1	-0,023367372
chr1	102200000	102450000	0,01991309	1	-0,022273867
chr1	102650000	102900000	0,005982472	1	-

					0,015536118
chr1	103000000	103250000	0,01207849	1	- 0,019237159
chr1	103050000	103300000	0,000439672	1	- 0,020227956
chr1	103100000	103350000	0,000221833	1	- 0,020855231
chr1	103150000	103400000	0,000406391	1	- 0,021070887
chr1	103200000	103450000	0,000143228	1	- 0,020938532
chr1	103250000	103500000	0,001529158	1	- 0,020625491
chr1	103300000	103550000	0,016621483	1	- 0,022348592
chr1	103350000	103600000	0,046441804	1	- 0,023142834
chr1	103400000	103650000	0,047552917	1	- 0,023634608
chr1	103450000	103700000	0,018553801	1	- 0,023708746
chr1	103500000	103750000	0,002058623	1	- 0,023384782
chr1	103550000	103800000	0,000454833	1	- 0,022792652
chr1	103600000	103850000	0,004907944	1	- 0,022088831
chr1	103650000	103900000	0,019668256	1	-

					0,021365721
chr1	103700000	103950000	0,039193281	1	-0,021365721
chr1	104650000	104900000	0,010948855	1	-0,032590548
chr1	104700000	104950000	0,000238765	1	-0,032266729
chr1	104750000	105000000	2,87E-05	1	-0,032159632
chr1	104800000	105050000	2,68E-05	1	-0,032490189
chr1	104850000	105100000	0,000243525	1	-0,03283683
chr1	104900000	105150000	0,004803831	1	-0,032934243
chr1	104950000	105200000	0,022891929	1	-0,032934243
chr1	105000000	105250000	0,040251332	1	-0,032934243
chr1	105050000	105300000	0,031461239	1	-0,033397823
chr1	105100000	105350000	0,009894894	1	-0,034463869
chr1	105150000	105400000	0,000903491	1	-0,035730527
chr1	105200000	105450000	0,001666775	1	-0,036808936
chr1	105250000	105500000	0,027118103	1	-0,036177646

chr1	105600000	105850000	0,032581476	1	- 0,024420656
chr1	105650000	105900000	0,001435096	1	- 0,024890036
chr1	105700000	105950000	0,000762614	1	- 0,025431115
chr1	105750000	106000000	0,002974827	1	- 0,025790276
chr1	105800000	106050000	0,002108218	1	- 0,025792636
chr1	105850000	106100000	0,000307866	1	- 0,025388624
chr1	105900000	106150000	0,000738303	1	-0,02466103
chr1	105950000	106200000	0,009101475	1	- 0,023772007
chr1	106000000	106250000	0,03360799	1	- 0,022883955
chr1	106150000	106400000	0,047779231	1	- 0,020787576
chr1	106200000	106450000	0,039081944	1	- 0,020123124
chr1	106250000	106500000	0,041788918	1	- 0,019380398
chr1	106450000	106700000	0,046608536	1	- 0,017077903
chr1	106500000	106750000	0,009254538	1	- 0,017297763
chr1	106550000	106800000	0,001624976	1	-

					0,017752606
chr1	106600000	106850000	0,02619511	1	- 0,018085899
chr1	106900000	107150000	0,038646896	1	- 0,021773163
chr1	106950000	107200000	0,002953721	1	- 0,023039913
chr1	107000000	107250000	0,000705017	1	- 0,023995085
chr1	107050000	107300000	0,008908148	1	- 0,024520572
chr1	107100000	107350000	0,0357916	1	- 0,024578028
chr1	107200000	107450000	0,031782428	1	- 0,023512726
chr1	107250000	107500000	0,004921307	1	- 0,022636286
chr1	107300000	107550000	0,019474696	1	- 0,021769393
chr1	107800000	108050000	0,026427681	1	- 0,048993598
chr1	107850000	108100000	0,006566137	1	- 0,052562687
chr1	116500000	116750000	0,01701608	1	- 0,028625873
chr1	116550000	116800000	0,000726909	1	- 0,030274094
chr1	116600000	116850000	9,06E-06	1	-

					0,031164877
chr1	116650000	116900000	1,05E-08	1	-0,031524588
chr1	116700000	116950000	1,68E-08	1	-0,031514766
chr1	116750000	117000000	4,69E-06	1	-0,031231463
chr1	116800000	117050000	0,000152502	1	-0,030705449
chr1	116850000	117100000	0,001583232	1	-0,029953342
chr1	116900000	117150000	0,007628523	1	-0,029038957
chr1	116950000	117200000	0,02147904	1	-0,028034283
chr1	117000000	117250000	0,041859649	1	-0,026980808
chr1	117500000	117750000	0,010490559	1	-0,019625283
chr1	117550000	117800000	0,000326547	1	-0,018726576
chr1	117600000	117850000	3,31E-06	1	-0,018075898
chr1	117650000	117900000	0,001043842	1	-0,017341295
chr1	118650000	118900000	0,011324701	1	-0,03506795
chr1	118950000	119200000	0,010119557	1	-0,028562301

chr1	142600000	142850000	0,038055413	1	-0,021893357
chr1	146850000	147100000	0,027655914	1	-0,018104337
chr1	148800000	149050000	0,00617364	1	-0,023891241
chr1	148850000	149100000	0,024536759	1	-0,023891241
chr1	152550000	152800000	0,02280081	1	-0,05165809
chr1	152600000	152850000	0,001415665	1	-0,05165809
chr1	152650000	152900000	4,67E-05	1	-0,05165809
chr1	152700000	152950000	0,000841017	1	-0,05165809
chr1	152750000	153000000	0,014012797	1	-0,05165809
chr1	158250000	158500000	0,025149907	1	-0,035010727
chr1	159200000	159450000	0,012988538	1	-0,024315301
chr1	159250000	159500000	0,003367267	1	-0,025516811
chr1	159700000	159950000	0,0498978	1	-0,013078076
chr1	159850000	160100000	0,030842162	1	-0,013212886
chr1	159900000	160150000	0,006199442	1	-0,014099159
chr1	163250000	163500000	0,005964029	1	-0,038520563
chr1	163300000	163550000	0,041342741	1	-

					0,036018282
chr1	163600000	163850000	0,010261663	1	-<br>0,028336394
chr1	163650000	163900000	0,025845089	1	-<br>0,028336394
chr1	164000000	164250000	0,009460406	1	-0,04002235
chr1	164050000	164300000	0,001824636	1	-0,04224339
chr1	164100000	164350000	0,016558279	1	-<br>0,043522764
chr1	164150000	164400000	0,045554556	1	-<br>0,043685194
chr1	164200000	164450000	0,036882269	1	-<br>0,042825329
chr1	164250000	164500000	0,007731716	1	-<br>0,041306396
chr1	164300000	164550000	0,001375791	1	-<br>0,039669074
chr1	164350000	164600000	0,015651301	1	-<br>0,038464095
chr1	164950000	165200000	0,008466473	1	-<br>0,069571166
chr1	165000000	165250000	0,009267639	1	-<br>0,069571166
chr1	165600000	165850000	0,048244754	1	-<br>0,019442203
chr1	166150000	166400000	0,005547861	1	-<br>0,041078475
chr1	166200000	166450000	0,000168539	1	-

					0,040494239
chr1	166250000	166500000	0,000384917	1	- 0,039764951
chr1	166300000	166550000	0,002222872	1	- 0,039764951
chr1	166350000	166600000	0,005459208	1	- 0,039764951
chr1	166400000	166650000	0,012026273	1	- 0,039764951
chr1	166450000	166700000	0,036055436	1	- 0,039764951
chr1	167150000	167400000	0,024738897	1	- 0,019410834
chr1	170200000	170450000	0,006311562	1	- 0,034420116
chr1	170250000	170500000	0,000970674	1	- 0,033337432
chr1	170300000	170550000	0,014808314	1	- 0,031815848
chr1	170600000	170850000	0,032603268	1	- 0,025072346
chr1	170650000	170900000	0,049055984	1	- 0,025072346
chr1	171000000	171250000	0,035091215	1	- 0,038862341
chr1	174600000	174850000	0,009642556	1	- 0,063987065
chr1	175150000	175400000	0,048835929	1	-

					0,022396572
chr1	175650000	175900000	0,045319433	1	-0,049984823
chr1	175700000	175950000	0,00328034	1	-0,04967247
chr1	175750000	176000000	0,000126894	1	-0,04882314
chr1	175800000	176050000	0,001282156	1	-0,047466121
chr1	175850000	176100000	0,011764117	1	-0,045654526
chr1	175900000	176150000	0,047072606	1	-0,0434998
chr1	184600000	184850000	0,030840623	1	-0,003786838
chr1	184650000	184900000	0,03094823	1	-0,003985335
chr1	184950000	185200000	0,01830059	1	-0,005221207
chr1	187400000	187650000	0,028680627	1	-0,013632767
chr1	187550000	187800000	0,013456546	1	-0,01344668
chr1	187600000	187850000	0,002697552	1	-0,01393623
chr1	187650000	187900000	0,014197556	1	-0,014340299
chr1	187700000	187950000	0,019022375	1	-0,014480135
chr1	187750000	188000000	0,005074208	1	-0,014312266
chr1	187800000	188050000	0,000646442	1	-0,013937895

chr1	187850000	188100000	0,003431121	1	-0,013548186
chr1	187900000	188150000	0,009581104	1	-0,013335852
chr1	187950000	188200000	0,005156562	1	-0,013414973
chr1	188000000	188250000	0,000800701	1	-0,01378396
chr1	188050000	188300000	0,005085111	1	-0,014346414
chr1	188100000	188350000	0,034592485	1	-0,014955137
chr1	188250000	188500000	0,016880843	1	-0,015803175
chr1	188300000	188550000	0,001568748	1	-0,015542794
chr1	188350000	188600000	0,00111053	1	-0,015231718
chr1	188400000	188650000	0,016750128	1	-0,014943799
chr1	188600000	188850000	0,036312066	1	-0,013985309
chr1	188650000	188900000	0,003587511	1	-0,013514034
chr1	188700000	188950000	0,020305418	1	-0,013065126
chr1	189050000	189300000	0,033203768	1	-0,017436196
chr1	189100000	189350000	0,0304993	1	-

					0,020165648
chr1	189450000	189700000	0,007380154	1	-0,026727435
chr1	189500000	189750000	0,004751602	1	-0,025807994
chr1	189550000	189800000	0,046601504	1	-0,024687355
chr1	189750000	190000000	0,042278987	1	-0,021323741
chr1	189800000	190050000	0,007615765	1	-0,021253064
chr1	189850000	190100000	0,000531508	1	-0,021316478
chr1	189900000	190150000	1,19E-05	1	-0,021485428
chr1	189950000	190200000	4,77E-05	1	-0,021632499
chr1	190000000	190250000	0,000299124	1	-0,021632499
chr1	190050000	190300000	0,00044555	1	-0,021632499
chr1	190100000	190350000	0,000142532	1	-0,021632499
chr1	190150000	190400000	0,000322233	1	-0,021737074
chr1	190200000	190450000	0,015166325	1	-0,022005688
chr1	190950000	191200000	0,028810693	1	-

					0,023880272
chr1	191000000	191250000	0,00915373	1	-0,02526812
chr1	193750000	194000000	0,016886227	1	-0,023830985
chr1	193800000	194050000	0,004381008	1	-0,022632706
chr1	194500000	194750000	0,047913682	1	-0,005035133
chr1	194950000	195200000	0,015972593	1	-0,008936612
chr1	195750000	196000000	0,040923026	1	-0,01825466
chr1	195800000	196050000	0,020980592	1	-0,019027445
chr1	195850000	196100000	0,010916064	1	-0,019694507
chr1	195900000	196150000	0,006029109	1	-0,020276551
chr1	195950000	196200000	0,004005175	1	-0,020779674
chr1	196000000	196250000	0,003638973	1	-0,021209495
chr1	196050000	196300000	0,004460602	1	-0,021586927
chr1	196100000	196350000	0,006266712	1	-0,021943596
chr1	196150000	196400000	0,008265782	1	-0,022316685
chr1	196200000	196450000	0,008539908	1	-

					0,022736538
chr1	196250000	196500000	0,005614067	1	- 0,023216918
chr1	196300000	196550000	0,001595684	1	- 0,023746524
chr1	196350000	196600000	0,000205372	1	- 0,024283701
chr1	196400000	196650000	0,003874488	1	- 0,024750554
chr1	196850000	197100000	0,006850884	1	- 0,013828604
chr1	196900000	197150000	0,012972488	1	- 0,013031814
chr1	196950000	197200000	0,008671904	1	- 0,012949046
chr1	197000000	197250000	0,007580606	1	- 0,013349795
chr1	208450000	208700000	0,003837458	1	- 0,061436666
chr1	208500000	208750000	0,014558142	1	- 0,058011359
chr1	210950000	211200000	0,034916298	1	- 0,040501212
chr1	213550000	213800000	0,046114333	1	- 0,048407934
chr1	213600000	213850000	0,003046529	1	- 0,048569517
chr1	213650000	213900000	0,000196914	1	-

					0,049091006
chr1	213700000	213950000	0,000463289	1	-0,049091006
chr1	213750000	214000000	0,003559445	1	-0,049091006
chr1	213800000	214050000	0,011835311	1	-0,049091006
chr1	213850000	214100000	0,014462024	1	-0,049091006
chr1	213900000	214150000	0,005101621	1	-0,050160318
chr1	213950000	214200000	0,00130193	1	-0,05164169
chr1	214000000	214250000	0,03383704	1	-0,05310355
chr1	215250000	215500000	0,004880091	1	-0,053178313
chr1	215300000	215550000	0,000175762	1	-0,052222551
chr1	215350000	215600000	0,001382885	1	-0,05202523
chr1	215400000	215650000	0,009279922	1	-0,05202523
chr1	215450000	215700000	0,030496343	1	-0,05202523
chr1	217400000	217650000	0,017084989	1	-0,029841591
chr1	217800000	218050000	0,003017692	1	-0,018224971
chr1	217850000	218100000	0,002311704	1	-0,017315375
chr1	219500000	219750000	0,016772742	1	-0,032229898

chr1	219550000	219800000	0,000896571	1	- 0,033870792
chr1	219600000	219850000	0,001169947	1	- 0,034950283
chr1	219650000	219900000	0,022000035	1	-0,03550004
chr1	220150000	220400000	0,02378287	1	- 0,018280751
chr1	220200000	220450000	0,003335489	1	-0,01706106
chr1	220250000	220500000	0,017048867	1	- 0,016454756
chr1	220300000	220550000	0,017924641	1	- 0,016439659
chr1	220350000	220600000	0,004030695	1	- 0,016877514
chr1	220400000	220650000	0,040230221	1	- 0,017513952
chr1	221150000	221400000	0,041187596	1	- 0,011194089
chr1	221200000	221450000	0,002471333	1	- 0,011325021
chr1	221250000	221500000	0,004902715	1	- 0,011887713
chr1	221800000	222050000	0,004111916	1	- 0,034973453
chr1	221850000	222100000	2,41E-05	1	- 0,034925928
chr1	221900000	222150000	4,49E-06	1	-0,03533787
chr1	221950000	222200000	0,000371182	1	-

					0,036258725
chr1	222000000	222250000	0,005751695	1	-0,036258725
chr1	222050000	222300000	0,021849112	1	-0,036258725
chr1	222100000	222350000	0,028142226	1	-0,036258725
chr1	222150000	222400000	0,011647481	1	-0,036258725
chr1	222200000	222450000	0,001556442	1	-0,037516391
chr1	222250000	222500000	0,012677761	1	-0,038760462
chr1	222800000	223050000	0,030862289	1	-0,01578689
chr1	222850000	223100000	0,017579374	1	-0,014674541
chr1	223250000	223500000	0,018653605	1	-0,023800597
chr1	223300000	223550000	0,002185711	1	-0,022858072
chr1	223350000	223600000	0,036275793	1	-0,022252817
chr1	226750000	227000000	0,009001034	1	-0,018527541
chr1	226800000	227050000	0,034744554	1	-0,017210947
chr1	226850000	227100000	0,030347037	1	-0,017202977

chr1	226900000	227150000	0,004657076	1	-<br>0,017586572
chr1	226950000	227200000	0,001401318	1	-<br>0,018105598
chr1	227000000	227250000	0,005911367	1	-<br>0,018429136
chr1	227050000	227300000	0,004280432	1	-<br>0,018263181
chr1	227100000	227350000	0,002189314	1	-<br>0,017442886
chr1	227800000	228050000	0,013894702	1	-<br>0,013982407
chr1	227850000	228100000	0,007009389	1	-<br>0,014919241
chr1	230150000	230400000	0,005879029	1	-0,03014848
chr1	230200000	230450000	0,005292043	1	-<br>0,028566516
chr1	233150000	233400000	0,037339832	1	-<br>0,020283023
chr1	233200000	233450000	0,002061982	1	-<br>0,021993487
chr1	233250000	233500000	0,01443073	1	-0,0228614
chr1	233300000	233550000	0,049826574	1	-<br>0,022979368
chr1	233450000	233700000	0,029123467	1	-0,01862549
chr1	233500000	233750000	0,010889088	1	-<br>0,018415956
chr1	233550000	233800000	0,002115038	1	-

					0,018464024
chr1	233600000	233850000	0,000297439	1	- 0,018957714
chr1	233650000	233900000	0,012360646	1	- 0,020122043
chr1	234100000	234350000	0,033068731	1	- 0,036433184
chr1	234150000	234400000	0,033701101	1	-0,03941224
chr1	234650000	234900000	0,013716542	1	- 0,014737443
chr1	234700000	234950000	0,004348651	1	-0,01291283
chr1	236300000	236550000	0,006576677	1	- 0,011175741
chr1	236350000	236600000	0,017920895	1	- 0,010648935
chr1	236500000	236750000	0,02276408	1	- 0,010292939
chr1	236550000	236800000	0,029242678	1	- 0,011256458
chr1	237050000	237300000	0,030046161	1	- 0,034676859
chr1	237100000	237350000	0,028375415	1	- 0,032248808
chr1	238350000	238600000	0,023482552	1	- 0,015735696
chr1	239350000	239600000	0,002013561	1	- 0,017088946
chr1	239400000	239650000	0,000647598	1	-

					0,017891042
chr1	239450000	239700000	0,001382386	1	-0,018154221
chr1	239500000	239750000	0,000419414	1	-0,018002473
chr1	239550000	239800000	0,0014829	1	-0,017654434
chr1	239600000	239850000	0,021143511	1	-0,017356341
chr1	239800000	240050000	0,01107763	1	-0,019133706
chr1	239850000	240100000	0,002238468	1	-0,0200178
chr1	239900000	240150000	0,03960915	1	-0,020695966
chr1	240200000	240450000	0,027644261	1	-0,014511943
chr1	240250000	240500000	0,030434752	1	-0,015483431
chr1	240500000	240750000	0,00378648	1	-0,017707129
chr1	240550000	240800000	0,000839983	1	-0,017707129
chr1	240600000	240850000	0,001503021	1	-0,017707129
chr1	240650000	240900000	0,00222463	1	-0,017641353
chr1	241200000	241450000	0,013612911	1	-0,044722311

chr1	242450000	242700000	0,025876283	1	-0,038627721
chr1	242500000	242750000	0,000686253	1	-0,038186593
chr1	242550000	242800000	8,77E-05	1	-0,037749077
chr1	242600000	242850000	0,000319078	1	-0,037476313
chr1	242650000	242900000	0,000241183	1	-0,037476313
chr1	242700000	242950000	4,75E-05	1	-0,037476313
chr1	242750000	243000000	2,67E-06	1	-0,037476313
chr1	242800000	243050000	3,03E-07	1	-0,037476313
chr1	242850000	243100000	9,91E-07	1	-0,037384994
chr1	242900000	243150000	7,03E-07	1	-0,037379629
chr1	242950000	243200000	2,66E-05	1	-0,037339298
chr1	243000000	243250000	0,00230684	1	-0,037211828
chr1	243050000	243300000	0,035376276	1	-0,037086665
chr1	243450000	243700000	0,003716249	1	-0,058368394

chr1	243500000	243750000	0,002608265	1	- 0,057108276
chr1	243550000	243800000	0,036984809	1	- 0,055195497
chr1	244450000	244700000	0,034074021	1	- 0,020922543
chr1	244500000	244750000	0,001842998	1	- 0,019597529
chr1	244550000	244800000	0,001538551	1	- 0,018934475
chr1	244600000	244850000	0,025668131	1	- 0,018728031
chr1	246050000	246300000	0,027754926	1	- 0,049460594
chr1	248250000	248500000	0,010080486	1	- 0,034503922
chr1	248300000	248550000	0,000523894	1	- 0,036042497
chr1	248350000	248600000	0,000103494	1	- 0,037062758
chr1	248400000	248650000	0,001384207	1	- 0,037544687
chr1	248450000	248700000	0,007097707	1	- 0,037551778
chr1	248500000	248750000	0,02247053	1	-0,03718766
chr2	400000	650000	0,004507295	1	- 0,036654961
chr2	450000	700000	0,030276145	1	-

					0,034579272
chr2	700000	950000	0,031837694	1	-0,029197755
chr2	750000	1000000	0,001762889	1	-0,029951207
chr2	800000	1050000	0,003580863	1	-0,031027595
chr2	850000	1100000	0,021693823	1	-0,032112811
chr2	900000	1150000	0,033747907	1	-0,032861591
chr2	950000	1200000	0,016812934	1	-0,032861591
chr2	1000000	1250000	0,002225458	1	-0,032861591
chr2	1050000	1300000	0,00389322	1	-0,032861591
chr2	1450000	1700000	0,032010343	1	-0,021451474
chr2	2050000	2300000	0,013483314	1	-0,057182969
chr2	2100000	2350000	0,00061562	1	-0,056223562
chr2	2150000	2400000	0,000756595	1	-0,054780749
chr2	2200000	2450000	0,00647218	1	-0,053188776
chr2	2250000	2500000	0,01671918	1	-0,05175033

chr2	2300000	2550000	0,019538781	1	-0,050665078
chr2	2350000	2600000	0,012072868	1	-0,050005664
chr2	2400000	2650000	0,003907177	1	-0,050005664
chr2	2450000	2700000	0,000579835	1	-0,050005664
chr2	2500000	2750000	3,08E-05	1	-0,050005664
chr2	2550000	2800000	1,24E-06	1	-0,050005664
chr2	2600000	2850000	5,22E-05	1	-0,049732404
chr2	2650000	2900000	0,002471345	1	-0,04971091
chr2	2700000	2950000	0,039431794	1	-0,049727252
chr2	4000000	4250000	0,022537162	1	-0,050135236
chr2	4050000	4300000	0,006971315	1	-0,047569068
chr2	5000000	5250000	0,00858822	1	-0,014393105
chr2	5050000	5300000	0,000122411	1	-0,014393105
chr2	5100000	5350000	8,51E-06	1	-0,014716469
chr2	5150000	5400000	0,000853239	1	-

					0,014761906
chr2	5200000	5450000	0,033926724	1	-0,014630992
chr2	5600000	5850000	0,012890981	1	-0,024263034
chr2	5650000	5900000	0,003512199	1	-0,024097327
chr2	5700000	5950000	0,042735596	1	-0,022749977
chr2	5950000	6200000	0,029640759	1	-0,019270053
chr2	6000000	6250000	0,001736904	1	-0,019270053
chr2	6050000	6300000	0,003828921	1	-0,019270053
chr2	6100000	6350000	0,036445398	1	-0,019173783
chr2	6900000	7150000	0,045151974	1	-0,060761698
chr2	6950000	7200000	0,00397039	1	-0,065037052
chr2	7000000	7250000	0,045766816	1	-0,062359883
chr2	7650000	7900000	0,017528394	1	-0,017344017
chr2	8150000	8400000	0,031378492	1	-0,033227411
chr2	8200000	8450000	0,003165983	1	-

					0,035443968
chr2	8250000	8500000	0,039229323	1	-0,036927785
chr2	12150000	12400000	0,015674425	1	-0,042447089
chr2	12200000	12450000	0,001143653	1	-0,041186425
chr2	12250000	12500000	0,00948622	1	-0,039227247
chr2	12950000	13200000	0,004650552	1	-0,018428624
chr2	13000000	13250000	0,000334764	1	-0,018839486
chr2	13050000	13300000	0,00294463	1	-0,01934438
chr2	13100000	13350000	0,010948938	1	-0,019885142
chr2	13150000	13400000	0,018162485	1	-0,020430524
chr2	13200000	13450000	0,02060957	1	-0,020977451
chr2	13250000	13500000	0,020423525	1	-0,021545919
chr2	13300000	13550000	0,021028922	1	-0,022171594
chr2	13350000	13600000	0,024836906	1	-0,022884604
chr2	13400000	13650000	0,032862059	1	-0,023598457

chr2	13450000	13700000	0,043009773	1	-0,023650692
chr2	13500000	13750000	0,047592409	1	-0,023650692
chr2	13550000	13800000	0,038141102	1	-0,023650692
chr2	13600000	13850000	0,018315819	1	-0,023739287
chr2	13650000	13900000	0,003752262	1	-0,024532426
chr2	13700000	13950000	0,000289447	1	-0,025324973
chr2	13750000	14000000	0,001489587	1	-0,025932285
chr2	13800000	14050000	0,011601696	1	-0,026225073
chr2	13850000	14100000	0,023373965	1	-0,026020101
chr2	13900000	14150000	0,013135404	1	-0,02536451
chr2	13950000	14200000	0,002118673	1	-0,024713585
chr2	14000000	14250000	0,016103559	1	-0,024016481
chr2	14300000	14550000	0,019013182	1	-0,029987806
chr2	14350000	14600000	0,00077486	1	-0,031767715
chr2	14400000	14650000	0,001353917	1	-

					0,032894277
chr2	14450000	14700000	0,004332205	1	- 0,033250018
chr2	14500000	14750000	0,002722824	1	- 0,032941628
chr2	14550000	14800000	0,000357795	1	- 0,032248653
chr2	14600000	14850000	0,00075173	1	- 0,031523173
chr2	14650000	14900000	0,00762494	1	- 0,031078387
chr2	14700000	14950000	0,021179686	1	- 0,031101511
chr2	14750000	15000000	0,024503681	1	- 0,031614794
chr2	14800000	15050000	0,014592909	1	- 0,032498695
chr2	14850000	15100000	0,004941518	1	- 0,033546499
chr2	14900000	15150000	0,001173866	1	- 0,034538049
chr2	14950000	15200000	0,000555847	1	- 0,035312122
chr2	15000000	15250000	0,002152538	1	- 0,035809722
chr2	15050000	15300000	0,016028286	1	- 0,036101462
chr2	15300000	15550000	0,041985393	1	-

					0,042076977
chr2	15350000	15600000	0,004564759	1	-0,044559143
chr2	15400000	15650000	0,042007025	1	-0,046642923
chr2	15950000	16200000	0,009736661	1	-0,019051157
chr2	16000000	16250000	0,000821683	1	-0,018287297
chr2	16050000	16300000	2,95E-05	1	-0,017838878
chr2	16100000	16350000	1,33E-06	1	-0,017596918
chr2	16150000	16400000	8,61E-05	1	-0,017503904
chr2	16200000	16450000	0,002312776	1	-0,017508135
chr2	16250000	16500000	0,014873454	1	-0,017520006
chr2	16300000	16550000	0,026149087	1	-0,01563678
chr2	16350000	16600000	0,009405193	1	-0,016096412
chr2	16400000	16650000	0,036981342	1	-0,017717781
chr2	16850000	17100000	0,017472953	1	-0,041605085
chr2	16900000	17150000	0,019091054	1	-0,039176996

chr2	17200000	17450000	0,0034662	1	-0,032104434
chr2	17250000	17500000	0,010171953	1	-0,032104434
chr2	17550000	17800000	0,015958329	1	-0,039363401
chr2	17600000	17850000	0,001134979	1	-0,041400262
chr2	17650000	17900000	0,005323957	1	-0,042863755
chr2	17700000	17950000	0,026760738	1	-0,043446448
chr2	17750000	18000000	0,035844883	1	-0,043043809
chr2	17800000	18050000	0,014286335	1	-0,041839271
chr2	17850000	18100000	0,001317559	1	-0,040264108
chr2	17900000	18150000	0,000574596	1	-0,039886232
chr2	17950000	18200000	0,002693689	1	-0,040507274
chr2	18000000	18250000	0,002471655	1	-0,040588874
chr2	18050000	18300000	0,000424213	1	-0,040080452
chr2	18100000	18350000	0,000298984	1	-0,039147321

chr2	18150000	18400000	0,003420805	1	- 0,038074121
chr2	18200000	18450000	0,010975315	1	- 0,037153759
chr2	18250000	18500000	0,012289727	1	- 0,036596446
chr2	18300000	18550000	0,005202824	1	- 0,036481189
chr2	18350000	18600000	0,000634585	1	- 0,036741861
chr2	18400000	18650000	4,14E-05	1	- 0,037192869
chr2	18450000	18700000	0,000162684	1	- 0,037580522
chr2	18500000	18750000	0,000252026	1	- 0,037659422
chr2	18550000	18800000	6,30E-05	1	- 0,037254556
chr2	18600000	18850000	0,000288435	1	- 0,036317686
chr2	18650000	18900000	0,006116882	1	- 0,034938681
chr2	18700000	18950000	0,036855612	1	- 0,033320973
chr2	18900000	19150000	0,034818466	1	-0,03046659
chr2	18950000	19200000	0,00318785	1	-0,03046659
chr2	19000000	19250000	0,007412123	1	-0,03046659
chr2	19400000	19650000	0,015475091	1	-

					0,042300431
chr2	19450000	19700000	0,001042941	1	-0,044440647
chr2	19500000	19750000	0,008840643	1	-0,045935957
chr2	22800000	23050000	0,029186455	1	-0,028806951
chr2	29400000	29650000	0,010731334	1	-0,023205417
chr2	29450000	29700000	0,001972059	1	-0,023854083
chr2	29500000	29750000	0,012183833	1	-0,024991069
chr2	29700000	29950000	0,017330473	1	-0,026805575
chr2	29750000	30000000	0,033158106	1	-0,026805575
chr2	34000000	34250000	0,008487845	1	-0,020738248
chr2	34050000	34300000	0,016958905	1	-0,019557268
chr2	34750000	35000000	0,049699923	1	-0,005696661
chr2	36900000	37150000	0,00722136	1	-0,00112445
chr2	36950000	37200000	0,016425851	1	-0,001190989
chr2	37000000	37250000	0,009932401	1	-0,001194895

chr2	41500000	41750000	0,025620958	1	-0,057109977
chr2	41550000	41800000	0,003771572	1	-0,060849771
chr2	44800000	45050000	0,019016412	1	-0,019695548
chr2	45400000	45650000	0,020955702	1	-0,005690864
chr2	47950000	48200000	0,007988101	1	-0,029456424
chr2	48000000	48250000	0,000369389	1	-0,028787683
chr2	48050000	48300000	0,003679731	1	-0,027885892
chr2	48100000	48350000	0,020427498	1	-0,026815033
chr2	48450000	48700000	0,037548632	1	-0,020628046
chr2	48500000	48750000	0,003930833	1	-0,021115075
chr2	48550000	48800000	0,006639577	1	-0,022317655
chr2	49050000	49300000	0,003905524	1	-0,042656054
chr2	49100000	49350000	0,011341722	1	-0,040441688
chr2	50550000	50800000	0,01273446	1	-0,007064606

chr2	50600000	50850000	0,005892896	1	-0,007647698
chr2	50650000	50900000	0,00476732	1	-0,007562867
chr2	51000000	51250000	0,002426411	1	-0,010424074
chr2	51050000	51300000	6,92E-05	1	-0,010424074
chr2	51100000	51350000	0,005512631	1	-0,010424074
chr2	51400000	51650000	0,005831032	1	-0,012953647
chr2	51450000	51700000	0,004481999	1	-0,013672991
chr2	51500000	51750000	0,018667808	1	-0,014011258
chr2	51550000	51800000	0,013182008	1	-0,013919756
chr2	51600000	51850000	0,00356281	1	-0,013511268
chr2	52000000	52250000	0,040960214	1	-0,018627868
chr2	52050000	52300000	0,008178156	1	-0,019679163
chr2	52100000	52350000	0,002399159	1	-0,02042086
chr2	52150000	52400000	0,002565853	1	-0,020888621
chr2	52200000	52450000	0,007163372	1	-

					0,021184317
					-
chr2	52250000	52500000	0,021518824	1	0,021439381
					-
chr2	52700000	52950000	0,035729065	1	0,030594207
chr2	52750000	53000000	0,004520141	1	-0,03227469
					-
chr2	53600000	53850000	0,022442219	1	0,036200788
					-
chr2	53650000	53900000	0,006083513	1	0,038775779
					-
chr2	54500000	54750000	0,045130524	1	0,026554297
					-
chr2	54550000	54800000	0,016661187	1	0,026554297
chr2	57250000	57500000	0,042032996	1	-0,01990926
chr2	57300000	57550000	0,023051368	1	-0,01990926
chr2	57350000	57600000	0,043379117	1	-0,01990926
					-
chr2	57500000	57750000	0,018125266	1	0,021211842
					-
chr2	57550000	57800000	0,013220135	1	0,022174237
					-
chr2	59200000	59450000	0,039777947	1	0,025879788
					-
chr2	60450000	60700000	0,027423771	1	0,031187392
					-
chr2	68550000	68800000	0,039665945	1	0,016809456
chr2	72300000	72550000	0,038451647	1	-

					0,042071678
chr2	72350000	72600000	0,001446532	1	- 0,040879371
chr2	72400000	72650000	0,009245007	1	- 0,038946601
chr2	76250000	76500000	0,015984278	1	- 0,034183013
chr2	76300000	76550000	0,000177997	1	- 0,034398296
chr2	76350000	76600000	0,000110572	1	- 0,035326261
chr2	76400000	76650000	0,000304812	1	- 0,035676561
chr2	76450000	76700000	0,010424974	1	- 0,037486233
chr2	76750000	77000000	0,013010983	1	- 0,043800735
chr2	79450000	79700000	0,031212644	1	- 0,030453549
chr2	79500000	79750000	0,012579502	1	- 0,031015367
chr2	79550000	79800000	0,006352748	1	- 0,031535056
chr2	79600000	79850000	0,003817619	1	- 0,031535056
chr2	79650000	79900000	0,002320616	1	- 0,031535056
chr2	79700000	79950000	0,001260611	1	-

					0,031535056
chr2	79750000	80000000	0,000658346	1	- 0,031535056
chr2	79800000	80050000	0,000497858	1	- 0,031995402
chr2	79850000	80100000	0,000917696	1	- 0,032375369
chr2	79900000	80150000	0,003181759	1	- 0,032684722
chr2	79950000	80200000	0,010803701	1	- 0,034324515
chr2	80000000	80250000	0,025873365	1	- 0,036986103
chr2	80050000	80300000	0,039569097	1	- 0,038559044
chr2	80100000	80350000	0,038024844	1	-0,03954774
chr2	80150000	80400000	0,022745301	1	- 0,040222166
chr2	80200000	80450000	0,00829413	1	-0,04060286
chr2	80250000	80500000	0,00179117	1	- 0,040789973
chr2	80300000	80550000	0,00022867	1	- 0,040893655
chr2	80350000	80600000	1,96E-05	1	- 0,040963329
chr2	80400000	80650000	1,29E-06	1	- 0,040958937
chr2	80450000	80700000	1,82E-07	1	-

					0,040766209
chr2	80500000	80750000	1,98E-05	1	-0,040252444
chr2	80550000	80800000	0,000571117	1	-0,039529234
chr2	80600000	80850000	0,005359032	1	-0,038893706
chr2	80650000	80900000	0,0242948	1	-0,038409211
chr2	80900000	81150000	0,016196054	1	-0,034878258
chr2	80950000	81200000	0,001233352	1	-0,033432463
chr2	81000000	81250000	0,00214186	1	-0,032350828
chr2	81050000	81300000	0,028086716	1	-0,031782259
chr2	81400000	81650000	0,038920573	1	-0,041606222
chr2	81450000	81700000	0,010968438	1	-0,043506863
chr2	81500000	81750000	0,001442847	1	-0,045119441
chr2	81550000	81800000	0,000119671	1	-0,046375975
chr2	81600000	81850000	0,002070911	1	-0,047216153
chr2	81650000	81900000	0,026573852	1	-

					0,042558754
chr2	82150000	82400000	0,008287678	1	- 0,024669565
chr2	82500000	82750000	0,031371663	1	- 0,034839857
chr2	82550000	82800000	0,003044853	1	- 0,033406072
chr2	82600000	82850000	0,033869482	1	- 0,031352699
chr2	82950000	83200000	0,048523295	1	- 0,021779238
chr2	83000000	83250000	0,00217162	1	- 0,021779238
chr2	83050000	83300000	0,00203459	1	- 0,021779238
chr2	83100000	83350000	0,008045835	1	- 0,021779238
chr2	83150000	83400000	0,006197521	1	- 0,021893448
chr2	83200000	83450000	0,001000094	1	- 0,022420171
chr2	83250000	83500000	0,001001149	1	- 0,023047271
chr2	83300000	83550000	0,011397217	1	- 0,023481488
chr2	83350000	83600000	0,033642387	1	- 0,022484793
chr2	83400000	83650000	0,030058375	1	-

					0,021623579
chr2	83450000	83700000	0,006819059	1	-0,021116597
chr2	83500000	83750000	0,002177355	1	-0,021077124
chr2	83550000	83800000	0,043442298	1	-0,021077124
chr2	84050000	84300000	0,029589483	1	-0,039989259
chr2	84100000	84350000	0,002150315	1	-0,042245282
chr2	84150000	84400000	0,000191035	1	-0,043806745
chr2	84200000	84450000	0,002909131	1	-0,044649555
chr2	84250000	84500000	0,01984222	1	-0,044827627
chr2	87750000	88000000	0,010082699	1	-0,030584959
chr2	95400000	95650000	0,013166246	1	-0,024337285
chr2	96200000	96450000	0,007547364	1	-0,021479648
chr2	98300000	98550000	0,016993011	1	-0,01932676
chr2	98350000	98600000	0,000445055	1	-0,018989496
chr2	98400000	98650000	0,000814222	1	-0,018609499

chr2	98450000	98700000	0,003458072	1	- 0,018257361
chr2	98500000	98750000	0,004977847	1	- 0,017954133
chr2	98550000	98800000	0,004146305	1	- 0,017690605
chr2	98600000	98850000	0,00281956	1	- 0,017444129
chr2	98650000	98900000	0,00205989	1	- 0,017444129
chr2	98700000	98950000	0,001974537	1	- 0,017444129
chr2	98750000	99000000	0,002543755	1	- 0,017444129
chr2	98800000	99050000	0,003827401	1	- 0,017444129
chr2	98850000	99100000	0,005441733	1	- 0,017193607
chr2	98900000	99150000	0,005856786	1	-0,01691685
chr2	98950000	99200000	0,003797904	1	- 0,016605268
chr2	99000000	99250000	0,001044753	1	- 0,016266655
chr2	99050000	99300000	8,56E-05	1	- 0,015935225
chr2	99100000	99350000	0,000188005	1	- 0,015667878
chr2	99150000	99400000	0,001491692	1	-

					0,015524978
chr2	99200000	99450000	0,002533014	1	-0,015547076
chr2	99250000	99500000	0,000908613	1	-0,015732089
chr2	99300000	99550000	0,000770361	1	-0,016027477
chr2	99350000	99600000	0,039060138	1	-0,01633122
chr2	100300000	100550000	0,009860375	1	-0,027549427
chr2	100350000	100600000	0,01184769	1	-0,026177164
chr2	100750000	101000000	0,03862996	1	-0,021094753
chr2	100800000	101050000	0,021734571	1	-0,020305468
chr2	100850000	101100000	0,009039573	1	-0,019646246
chr2	100900000	101150000	0,001661137	1	-0,019074572
chr2	100950000	101200000	0,000184987	1	-0,018589361
chr2	101000000	101250000	0,002115893	1	-0,018230611
chr2	101050000	101300000	0,012786341	1	-0,018070232
chr2	101100000	101350000	0,018585714	1	-0,018172217

chr2	101150000	101400000	0,006213205	1	-0,018556586
chr2	101200000	101450000	0,005423014	1	-0,019147061
chr2	102150000	102400000	0,012741756	1	-0,023870744
chr2	102400000	102650000	0,012015349	1	-0,022336191
chr2	102800000	103050000	0,003195881	1	-0,032759328
chr2	102850000	103100000	0,000215681	1	-0,032161265
chr2	102900000	103150000	0,001151091	1	-0,031641408
chr2	102950000	103200000	0,001985508	1	-0,031368935
chr2	103000000	103250000	0,000854303	1	-0,031355521
chr2	103050000	103300000	8,79E-05	1	-0,031453522
chr2	103100000	103350000	1,26E-06	1	-0,031414025
chr2	103150000	103400000	9,77E-06	1	-0,030977537
chr2	103200000	103450000	0,001097776	1	-0,029972034
chr2	103250000	103500000	0,021433704	1	-0,028346274

chr2	103800000	104050000	0,021130412	1	-0,011054034
chr2	103850000	104100000	0,001358164	1	-0,011299703
chr2	103900000	104150000	0,003572514	1	-0,011391857
chr2	103950000	104200000	0,001422794	1	-0,011259769
chr2	104000000	104250000	0,000733961	1	-0,0108917
chr2	104050000	104300000	0,016188012	1	-0,010319621
chr2	104400000	104650000	0,011922617	1	-0,008244276
chr2	104450000	104700000	0,008975644	1	-0,007780052
chr2	105200000	105450000	0,004219479	1	-0,030435105
chr2	105250000	105500000	0,005037503	1	-0,031928843
chr2	106550000	106800000	0,008601765	1	-0,028333692
chr2	106600000	106850000	0,026519694	1	-0,026415331
chr2	107400000	107650000	0,004382362	1	-0,016796202
chr2	107450000	107700000	0,001646865	1	-0,016187797
chr2	107500000	107750000	0,026024958	1	-

					0,016187797
chr2	107900000	108150000	0,028005985	1	-0,011028651
chr2	108400000	108650000	0,014311166	1	-0,019535353
chr2	109450000	109700000	0,027580372	1	-0,020544019
chr2	109500000	109750000	0,002304879	1	-0,019970971
chr2	109550000	109800000	0,009913113	1	-0,018843113
chr2	111750000	112000000	0,007897564	1	-0,015404698
chr2	111800000	112050000	7,66E-05	1	-0,016467376
chr2	111850000	112100000	0,000381424	1	-0,016750565
chr2	111900000	112150000	0,00179156	1	-0,016661429
chr2	111950000	112200000	0,019700032	1	-0,016486543
chr2	114300000	114550000	0,006051845	1	-0,014768978
chr2	114350000	114600000	0,00626047	1	-0,015345733
chr2	114950000	115200000	0,003659168	1	-0,046491666
chr2	115000000	115250000	0,020799426	1	-

					0,043962996
chr2	115400000	115650000	0,02723708	1	- 0,030398366
chr2	115450000	115700000	0,005442838	1	- 0,030169722
chr2	115500000	115750000	0,000836881	1	- 0,030008135
chr2	115550000	115800000	0,000122198	1	- 0,029905709
chr2	115600000	115850000	1,42E-05	1	- 0,029940574
chr2	115650000	115900000	3,45E-06	1	- 0,030228102
chr2	115700000	115950000	0,000188749	1	- 0,030816093
chr2	115750000	116000000	0,002793407	1	- 0,031198349
chr2	115800000	116050000	0,012496803	1	- 0,031568138
chr2	115850000	116100000	0,023099248	1	- 0,032041329
chr2	115900000	116150000	0,018392241	1	- 0,032681481
chr2	115950000	116200000	0,00508061	1	- 0,033925668
chr2	116000000	116250000	0,000566758	1	- 0,033098644
chr2	116050000	116300000	0,005327763	1	-

					0,031650702
chr2	116350000	116600000	0,036393128	1	-0,02506305
chr2	116400000	116650000	0,003381845	1	-0,02506305
chr2	116450000	116700000	0,029821933	1	-0,024996074
chr2	116700000	116950000	0,039001667	1	-0,029563411
chr2	116750000	117000000	0,003286269	1	-0,031231451
chr2	116800000	117050000	0,000938978	1	-0,032536602
chr2	116850000	117100000	0,013352772	1	-0,032849519
chr2	117300000	117550000	0,021747746	1	-0,023228167
chr2	117350000	117600000	0,001919391	1	-0,02210457
chr2	117400000	117650000	0,012380177	1	-0,02129048
chr2	117600000	117850000	0,01361342	1	-0,0229457
chr2	117650000	117900000	0,000785843	1	-0,024029915
chr2	117700000	117950000	0,000762399	1	-0,024838326
chr2	117750000	118000000	0,003493637	1	-0,025185751
chr2	117800000	118050000	0,00314589	1	-0,025056921
chr2	117850000	118100000	0,000553477	1	-0,024594441

chr2	117900000	118150000	0,001118379	1	-0,024038765
chr2	117950000	118200000	0,016485669	1	-0,023636675
chr2	118100000	118350000	0,033520044	1	-0,024794128
chr2	118150000	118400000	0,005076856	1	-0,025903081
chr2	122950000	123200000	0,004995959	1	-0,034727218
chr2	123000000	123250000	0,01322992	1	-0,033146111
chr2	123450000	123700000	0,020409246	1	-0,023041102
chr2	123500000	123750000	0,003241264	1	-0,024087242
chr2	123550000	123800000	0,040651464	1	-0,025584708
chr2	123800000	124050000	0,007631155	1	-0,028858791
chr2	123850000	124100000	0,005099535	1	-0,027448121
chr2	124200000	124450000	0,004758097	1	-0,02150405
chr2	124250000	124500000	0,014169113	1	-0,022602625
chr2	124700000	124950000	0,042820986	1	-0,028801461
chr2	124750000	125000000	0,028854271	1	-

					0,029536242
chr2	124800000	125050000	0,012358468	1	-0,030538243
chr2	124850000	125100000	0,002208749	1	-0,031550799
chr2	124900000	125150000	0,000221924	1	-0,03247366
chr2	124950000	125200000	0,00247918	1	-0,03158503
chr2	125000000	125250000	0,020076275	1	-0,030400206
chr2	125150000	125400000	0,026777404	1	-0,029407032
chr2	125200000	125450000	0,003132682	1	-0,029244861
chr2	125250000	125500000	0,006116912	1	-0,028901448
chr2	125550000	125800000	0,008380431	1	-0,034749121
chr2	125600000	125850000	0,011503981	1	-0,036627176
chr2	125950000	126200000	0,031918212	1	-0,026156107
chr2	126000000	126250000	0,001307587	1	-0,024341657
chr2	126050000	126300000	0,003846152	1	-0,023430931
chr2	126100000	126350000	0,004644998	1	-0,02333801
chr2	126150000	126400000	0,000918395	1	-0,023789007

chr2	126200000	126450000	0,000463065	1	- 0,024417677
chr2	126250000	126500000	0,005109152	1	- 0,024883052
chr2	126300000	126550000	0,015671028	1	- 0,024968462
chr2	126350000	126600000	0,01383964	1	- 0,024628091
chr2	126400000	126650000	0,003029396	1	- 0,023980913
chr2	126450000	126700000	0,001185757	1	- 0,023247476
chr2	126500000	126750000	0,023804181	1	- 0,022676665
chr2	127000000	127250000	0,039053888	1	- 0,035672997
chr2	127050000	127300000	0,018779876	1	-0,03710472
chr2	127100000	127350000	0,00592795	1	- 0,038404641
chr2	127150000	127400000	0,00070932	1	- 0,039559621
chr2	127200000	127450000	0,000559577	1	- 0,040519434
chr2	127250000	127500000	0,020711061	1	- 0,041187478
chr2	128250000	128500000	0,024224312	1	- 0,007051733
chr2	129400000	129650000	0,022266127	1	-

					0,056856645
chr2	129450000	129700000	0,001701026	1	-0,055931948
chr2	129500000	129750000	0,017409054	1	-0,055931948
chr2	129750000	130000000	0,032847497	1	-0,049124778
chr2	129800000	130050000	0,001841093	1	-0,044969058
chr2	129850000	130100000	0,000915024	1	-0,046056051
chr2	129900000	130150000	0,004626262	1	-0,046965536
chr2	129950000	130200000	0,005568128	1	-0,047484027
chr2	130000000	130250000	0,002086727	1	-0,047595483
chr2	130050000	130300000	0,000218872	1	-0,047595483
chr2	130100000	130350000	1,10E-05	1	-0,047595483
chr2	130150000	130400000	0,000125518	1	-0,047595483
chr2	130200000	130450000	0,001380224	1	-0,047595483
chr2	130250000	130500000	0,009388825	1	-0,0473932
chr2	131400000	131650000	0,01741844	1	-0,021601802

chr2	131800000	132050000	0,018946939	1	-0,012028109
chr2	131850000	132100000	0,002417153	1	-0,011659774
chr2	131900000	132150000	0,021611818	1	-0,010956335
chr2	132800000	133050000	0,015976127	1	-0,022767745
chr2	132850000	133100000	0,000318965	1	-0,024256224
chr2	132900000	133150000	0,00024885	1	-0,02499919
chr2	132950000	133200000	0,000183865	1	-0,025128754
chr2	133000000	133250000	0,00127045	1	-0,024865236
chr2	133050000	133300000	0,036110355	1	-0,024491182
chr2	133350000	133600000	0,019050115	1	-0,030491941
chr2	134700000	134950000	0,005377926	1	-0,050410608
chr2	134750000	135000000	0,000186054	1	-0,052531569
chr2	134800000	135050000	5,56E-06	1	-0,05370061
chr2	134850000	135100000	0,000113329	1	-0,054164081
chr2	134900000	135150000	0,00155925	1	-0,054105333

chr2	134950000	135200000	0,01390886	1	-0,053978277
chr2	136250000	136500000	0,026818463	1	-0,004832125
chr2	137000000	137250000	0,04482379	1	-0,047989678
chr2	137050000	137300000	0,01611136	1	-0,0485113
chr2	137100000	137350000	0,002767312	1	-0,0481173
chr2	137150000	137400000	0,000738318	1	-0,046473226
chr2	137200000	137450000	0,025540552	1	-0,043377258
chr2	138500000	138750000	0,010121424	1	-0,022347629
chr2	138550000	138800000	0,01060541	1	-0,023147892
chr2	138600000	138850000	0,027253959	1	-0,023584962
chr2	138800000	139050000	0,046715831	1	-0,029088239
chr2	138850000	139100000	0,01313449	1	-0,029088239
chr2	138900000	139150000	0,001189631	1	-0,029088239
chr2	138950000	139200000	0,001574145	1	-0,029578883
chr2	139000000	139250000	0,030657144	1	-0,030128148

chr2	139300000	139550000	0,009841083	1	-0,022025795
chr2	139350000	139600000	0,003187237	1	-0,022736747
chr2	139400000	139650000	0,010872878	1	-0,023101681
chr2	139450000	139700000	0,0056964	1	-0,022784255
chr2	139500000	139750000	0,003423774	1	-0,021640948
chr2	140550000	140800000	0,038755388	1	-0,024553622
chr2	140600000	140850000	0,003123528	1	-0,026404281
chr2	140650000	140900000	0,022457683	1	-0,027556165
chr2	140800000	141050000	0,014466877	1	-0,024659381
chr2	140850000	141100000	0,019919054	1	-0,026500839
chr2	141150000	141400000	0,03281373	1	-0,031828928
chr2	142950000	143200000	0,034650309	1	-0,012954841
chr2	143000000	143250000	0,009625853	1	-0,013393851
chr2	143050000	143300000	0,00344491	1	-0,013869496

chr2	145750000	146000000	0,001757257	1	-0,016615181
chr2	145800000	146050000	6,13E-06	1	-0,016648214
chr2	145850000	146100000	8,44E-07	1	-0,016761189
chr2	145900000	146150000	1,61E-05	1	-0,016897771
chr2	145950000	146200000	4,49E-05	1	-0,016959369
chr2	146000000	146250000	1,86E-05	1	-0,016837018
chr2	146050000	146300000	0,000156106	1	-0,016433245
chr2	146100000	146350000	0,006938217	1	-0,015683149
chr2	146900000	147150000	0,037990443	1	-0,006257757
chr2	148450000	148700000	0,032902259	1	-0,042528104
chr2	148500000	148750000	0,006126625	1	-0,044499118
chr2	148550000	148800000	0,000491429	1	-0,046102023
chr2	148600000	148850000	0,004491986	1	-0,047855922
chr2	152450000	152700000	0,042673013	1	-0,013486899

chr2	153950000	154200000	0,041764148	1	-0,082445197
chr2	154000000	154250000	0,005082218	1	-0,0871215
chr2	154050000	154300000	0,000191114	1	-0,090597431
chr2	154100000	154350000	0,000214291	1	-0,092740592
chr2	154150000	154400000	0,002853033	1	-0,087981224
chr2	154200000	154450000	0,015258223	1	-0,084593868
chr2	155350000	155600000	0,005959996	1	-0,022667188
chr2	155400000	155650000	0,002259518	1	-0,022906134
chr2	155450000	155700000	0,046397711	1	-0,022906134
chr2	156050000	156300000	0,005384613	1	-0,050616979
chr2	156100000	156350000	0,000649278	1	-0,052867273
chr2	156150000	156400000	0,009568525	1	-0,054229632
chr2	156500000	156750000	0,041300856	1	-0,042150825
chr2	156550000	156800000	0,005435253	1	-0,04005627
chr2	156600000	156850000	0,000196993	1	-0,038534237

chr2	156650000	156900000	0,000161602	1	-0,037624587
chr2	156700000	156950000	0,000916378	1	-0,037288968
chr2	156750000	157000000	0,001252332	1	-0,037422209
chr2	156800000	157050000	0,000505804	1	-0,037876266
chr2	156850000	157100000	5,27E-05	1	-0,038473585
chr2	156900000	157150000	0,000104875	1	-0,039025478
chr2	156950000	157200000	0,001404092	1	-0,039362899
chr2	157000000	157250000	0,005394648	1	-0,039364767
chr2	157050000	157300000	0,008936946	1	-0,038992603
chr2	157100000	157350000	0,007874878	1	-0,038302224
chr2	157150000	157400000	0,004147141	1	-0,034900854
chr2	157200000	157450000	0,001626824	1	-0,034551861
chr2	157250000	157500000	0,000940735	1	-0,034044669
chr2	157300000	157550000	0,002000904	1	-0,033206168

chr2	157350000	157600000	0,010067812	1	-0,031918092
chr2	158400000	158650000	0,022152345	1	-0,038333439
chr2	161350000	161600000	0,014147182	1	-0,014276523
chr2	161400000	161650000	0,004317729	1	-0,014495229
chr2	161450000	161700000	0,002524233	1	-0,014495229
chr2	161500000	161750000	0,002011845	1	-0,014495229
chr2	161550000	161800000	0,001522428	1	-0,014495229
chr2	161600000	161850000	0,000914235	1	-0,014495229
chr2	161650000	161900000	0,000419954	1	-0,014711932
chr2	161700000	161950000	0,000174908	1	-0,014912987
chr2	161750000	162000000	0,000118288	1	-0,015080709
chr2	161800000	162050000	0,000235055	1	-0,015208965
chr2	161850000	162100000	0,000933924	1	-0,016060399
chr2	161900000	162150000	0,003879966	1	-0,016496849

chr2	161950000	162200000	0,013415442	1	-0,01707945
chr2	162000000	162250000	0,037565179	1	-0,017825953
chr2	162200000	162450000	0,034587522	1	-0,019813103
chr2	162250000	162500000	0,026472393	1	-0,017996139
chr2	163550000	163800000	0,024105945	1	-0,037027758
chr2	165250000	165500000	0,027883493	1	-0,016483145
chr2	166150000	166400000	0,005748796	1	-0,046729837
chr2	166200000	166450000	0,002219435	1	-0,047838763
chr2	166250000	166500000	0,007074632	1	-0,049488085
chr2	166300000	166550000	0,024991387	1	-0,049488085
chr2	166450000	166700000	0,026375768	1	-0,049488085
chr2	166500000	166750000	0,004776303	1	-0,051451435
chr2	166550000	166800000	0,00046406	1	-0,053340156
chr2	166600000	166850000	0,005137186	1	-0,054735033
chr2	166650000	166900000	0,044151652	1	-

					0,055304676
chr2	167850000	168100000	0,022603924	1	-<br>0,011516082
chr2	170950000	171200000	0,003405447	1	-<br>0,014927134
chr2	171000000	171250000	5,44E-05	1	-<br>0,014927134
chr2	171050000	171300000	2,58E-06	1	-<br>0,014927134
chr2	171100000	171350000	3,33E-06	1	-<br>0,014927134
chr2	171150000	171400000	4,03E-06	1	-0,01493484
chr2	171200000	171450000	0,001603756	1	-<br>0,014971492
chr2	174200000	174450000	0,027690584	1	-<br>0,012292051
chr2	174350000	174600000	0,008802775	1	-<br>0,011945325
chr2	174400000	174650000	0,031583322	1	-<br>0,011945325
chr2	174600000	174850000	0,005421709	1	-<br>0,013434225
chr2	174650000	174900000	0,004852523	1	-0,01410862
chr2	175800000	176050000	0,026549039	1	-<br>0,013605797
chr2	175850000	176100000	0,000201757	1	-0,0138356
chr2	175900000	176150000	0,009884965	1	-<br>0,014825617

chr2	176500000	176750000	0,007315657	1	-0,044778139
chr2	176550000	176800000	0,013743392	1	-0,047282407
chr2	176900000	177150000	0,023853243	1	-0,034738119
chr2	177250000	177500000	0,005124938	1	-0,047128139
chr2	177300000	177550000	0,046748058	1	-0,047128139
chr2	178450000	178700000	0,023396936	1	-0,021479427
chr2	178500000	178750000	0,048897963	1	-0,0200749
chr2	178700000	178950000	0,01914563	1	-0,018441759
chr2	178750000	179000000	0,002556648	1	-0,018441759
chr2	178800000	179050000	0,010718139	1	-0,018189976
chr2	178850000	179100000	0,00996887	1	-0,01814636
chr2	178900000	179150000	0,002156413	1	-0,018602922
chr2	178950000	179200000	0,016791038	1	-0,019243619
chr2	179250000	179500000	0,00453209	1	-0,014353864
chr2	179300000	179550000	2,85E-05	1	-0,014173157

chr2	179350000	179600000	0,002271296	1	-0,013485908
chr2	179800000	180050000	0,037089177	1	-0,006905155
chr2	180050000	180300000	0,042264478	1	-0,007502747
chr2	183550000	183800000	0,026359695	1	-0,016356981
chr2	183600000	183850000	0,002591926	1	-0,017671692
chr2	183650000	183900000	0,013556239	1	-0,01830396
chr2	183700000	183950000	0,015862982	1	-0,018320339
chr2	183750000	184000000	0,00375114	1	-0,01738432
chr2	183800000	184050000	0,024535446	1	-0,018652072
chr2	184050000	184300000	0,043971341	1	-0,019682637
chr2	187900000	188150000	0,017366647	1	-0,023888771
chr2	187950000	188200000	0,006033133	1	-0,023888771
chr2	188300000	188550000	0,003519126	1	-0,017927948
chr2	188350000	188600000	0,00152066	1	-0,017669366
chr2	188400000	188650000	0,001334835	1	-0,017343188

chr2	188450000	188700000	0,004289171	1	-0,016416764
chr2	189250000	189500000	0,043027227	1	-0,024514494
chr2	189300000	189550000	0,003343084	1	-0,024514494
chr2	189350000	189600000	5,01E-05	1	-0,024514494
chr2	189400000	189650000	4,22E-06	1	-0,024514494
chr2	189450000	189700000	0,000896918	1	-0,024589604
chr2	189500000	189750000	0,021378848	1	-0,02443093
chr2	189750000	190000000	0,010807223	1	-0,028614709
chr2	189800000	190050000	0,04277209	1	-0,030198242
chr2	193850000	194100000	0,022046105	1	-0,025520352
chr2	194650000	194900000	0,016141846	1	-0,025226746
chr2	194700000	194950000	0,004085987	1	-0,026493658
chr2	194750000	195000000	0,005725203	1	-0,027194553
chr2	194800000	195050000	0,014411263	1	-0,027580639
chr2	194850000	195100000	0,022492786	1	-

					0,027955292
chr2	194900000	195150000	0,01495793	1	-0,028543292
chr2	194950000	195200000	0,002911479	1	-0,029390591
chr2	195000000	195250000	0,00102271	1	-0,030344473
chr2	195050000	195300000	0,015879015	1	-0,031106537
chr2	195250000	195500000	0,01857352	1	-0,028436869
chr2	195300000	195550000	0,001626824	1	-0,027028389
chr2	195350000	195600000	0,010211036	1	-0,026043545
chr2	195400000	195650000	0,046399204	1	-0,025721435
chr2	195650000	195900000	0,045997046	1	-0,03027686
chr2	195700000	195950000	0,033412622	1	-0,031319591
chr2	195750000	196000000	0,013062406	1	-0,032390535
chr2	195800000	196050000	0,00167008	1	-0,033509104
chr2	195850000	196100000	0,006310634	1	-0,034569639
chr2	197450000	197700000	0,031101434	1	-0,019022345

chr2	197500000	197750000	0,000321634	1	-0,020660743
chr2	197550000	197800000	0,000139405	1	-0,021347361
chr2	197600000	197850000	8,42E-05	1	-0,021377639
chr2	197650000	197900000	0,000460577	1	-0,02110543
chr2	197700000	197950000	0,00300612	1	-0,02085747
chr2	197750000	198000000	0,0031174	1	-0,020860444
chr2	197800000	198050000	0,001041065	1	-0,021178104
chr2	197850000	198100000	0,029262893	1	-0,021691715
chr2	198750000	199000000	0,047612334	1	-0,037124915
chr2	198800000	199050000	0,015357634	1	-0,034605057
chr2	200000000	200250000	0,003872658	1	-0,027708643
chr2	200050000	200300000	0,005484733	1	-0,029130361
chr2	200100000	200350000	0,049760565	1	-0,029726598
chr2	200400000	200650000	0,034420773	1	-0,023397905
chr2	200450000	200700000	0,008601765	1	-0,022362073

chr2	200500000	200750000	0,001451516	1	-0,021594494
chr2	200550000	200800000	0,000138254	1	-0,021074619
chr2	200600000	200850000	5,39E-06	1	-0,020753088
chr2	200650000	200900000	3,59E-05	1	-0,020571158
chr2	200700000	200950000	0,000613319	1	-0,020487852
chr2	200750000	201000000	0,00260587	1	-0,02049854
chr2	200800000	201050000	0,003224588	1	-0,020630006
chr2	200850000	201100000	0,000877082	1	-0,020916482
chr2	200900000	201150000	0,007758121	1	-0,021339827
chr2	204650000	204900000	0,025373762	1	-0,011595041
chr2	207300000	207550000	0,035824166	1	-0,014416428
chr2	209850000	210100000	0,008445114	1	-0,041147167
chr2	209900000	210150000	0,003060886	1	-0,043150286
chr2	210300000	210550000	0,015488038	1	-0,027812786
chr2	210350000	210600000	0,012061339	1	-

					0,029207586
chr2	210550000	210800000	0,044366566	1	-0,029207586
chr2	210600000	210850000	0,00933892	1	-0,030655944
chr2	210650000	210900000	0,000668966	1	-0,031891228
chr2	210700000	210950000	0,000311978	1	-0,032770931
chr2	210750000	211000000	0,009554083	1	-0,033255339
chr2	212800000	213050000	0,014017231	1	-0,031783679
chr2	212850000	213100000	0,000773589	1	-0,031783679
chr2	212900000	213150000	0,010858651	1	-0,031783679
chr2	213700000	213950000	0,041439958	1	-0,021253712
chr2	213750000	214000000	0,000674371	1	-0,02109006
chr2	213800000	214050000	4,34E-05	1	-0,021534008
chr2	213850000	214100000	0,007451695	1	-0,022881
chr2	214250000	214500000	0,006489844	1	-0,03523265
chr2	214300000	214550000	0,004187173	1	-0,033638247
chr2	215600000	215850000	0,007110392	1	-0,037862577

chr2	215650000	215900000	0,015603931	1	-0,04002143
chr2	218150000	218400000	0,04587998	1	- 0,036531481
chr2	218200000	218450000	0,005642793	1	- 0,038871342
chr2	220950000	221200000	0,018902964	1	- 0,013322263
chr2	221000000	221250000	0,004549962	1	-0,01261706
chr2	222900000	223150000	0,031081522	1	- 0,024100614
chr2	222950000	223200000	0,004289006	1	- 0,025198462
chr2	223000000	223250000	0,000435956	1	- 0,026181917
chr2	223050000	223300000	0,004917141	1	- 0,026845831
chr2	223100000	223350000	0,033900227	1	- 0,027050969
chr2	223550000	223800000	0,011410755	1	- 0,016755467
chr2	223900000	224150000	0,042437013	1	-0,02384923
chr2	223950000	224200000	0,030409653	1	- 0,024579525
chr2	224000000	224250000	0,026046666	1	- 0,024579525
chr2	224050000	224300000	0,015598652	1	- 0,024579525
chr2	224100000	224350000	0,003366808	1	-

					0,024579525
chr2	224150000	224400000	0,002248719	1	-<br>0,024579525
chr2	225400000	225650000	0,038079991	1	-<br>0,025420319
chr2	225450000	225700000	0,00749598	1	-<br>0,025707435
chr2	225500000	225750000	0,001656464	1	-0,02594012
chr2	225550000	225800000	0,00040861	1	-0,02594012
chr2	225600000	225850000	5,15E-05	1	-0,02594012
chr2	225650000	225900000	0,000267937	1	-0,02594012
chr2	225700000	225950000	0,013701086	1	-0,02594012
chr2	226700000	226950000	0,009211211	1	-<br>0,032098232
chr2	229100000	229350000	0,047614101	1	-<br>0,031386894
chr2	229150000	229400000	0,002488477	1	-<br>0,031356683
chr2	229200000	229450000	4,35E-05	1	-0,03153614
chr2	229250000	229500000	8,68E-05	1	-<br>0,032122751
chr2	229300000	229550000	0,00213573	1	-<br>0,032122751
chr2	229350000	229600000	0,015962814	1	-<br>0,032122751
chr2	229400000	229650000	0,046034707	1	-<br>0,032122751
chr2	229500000	229750000	0,036575909	1	-

					0,033151087
chr2	229550000	229800000	0,007893522	1	- 0,034483983
chr2	229600000	229850000	0,000809588	1	- 0,035853359
chr2	229650000	229900000	0,008005192	1	- 0,036931704
chr2	229950000	230200000	0,006548859	1	- 0,030519678
chr2	230300000	230550000	0,004907286	1	- 0,038241814
chr2	230350000	230600000	0,046323163	1	- 0,040134186
chr2	230750000	231000000	0,004912409	1	- 0,023309457
chr2	230800000	231050000	0,007607633	1	- 0,023995199
chr2	230850000	231100000	0,014015031	1	-0,02419947
chr2	230900000	231150000	0,003900736	1	-0,02365111
chr2	230950000	231200000	0,007419295	1	-0,02365111
chr2	234600000	234850000	0,003081553	1	- 0,004766565
chr2	234650000	234900000	0,000810464	1	- 0,004705856
chr2	234700000	234950000	0,002328628	1	- 0,004622674
chr2	234750000	235000000	0,005720411	1	- 0,004470797

chr2	234800000	235050000	0,028378598	1	-0,004239596
chr2	235050000	235300000	0,037337188	1	-0,004076046
chr2	237300000	237550000	0,032881763	1	-0,013671185
chr2	237350000	237600000	0,005509595	1	-0,013833725
chr2	237400000	237650000	0,006639013	1	-0,013032972
chr2	240300000	240550000	0,031953931	1	-0,050829497
chr2	240350000	240600000	0,004301363	1	-0,053278207
chr2	240400000	240650000	0,000312171	1	-0,055307674
chr2	240450000	240700000	0,003087615	1	-0,056662265
chr2	240500000	240750000	0,026468597	1	-0,052595784
chr3	0	250000	3,71E-05	1	-0,033967417
chr3	50000	300000	1,03E-05	1	-0,034136757
chr3	100000	350000	6,24E-07	1	-0,034385791
chr3	150000	400000	8,61E-08	1	-0,034589828

chr3	200000	450000	2,08E-06	1	-0,034658745
chr3	250000	500000	0,000222349	1	-0,034593086
chr3	300000	550000	0,003913869	1	-0,03681482
chr3	350000	600000	0,022760827	1	-0,03837389
chr3	600000	850000	0,019538781	1	-0,044479522
chr3	650000	900000	0,00296633	1	-0,044307883
chr3	700000	950000	0,000172427	1	-0,043558958
chr3	750000	1000000	0,001189721	1	-0,042256764
chr3	800000	1050000	0,014407911	1	-0,040469522
chr3	1200000	1450000	0,023295866	1	-0,028741319
chr3	1250000	1500000	0,001360017	1	-0,029444787
chr3	1300000	1550000	0,004248579	1	-0,030692205
chr3	1350000	1600000	0,046017279	1	-0,031908841
chr3	1600000	1850000	0,039431794	1	-0,03780607
chr3	1650000	1900000	0,003958142	1	-0,038316083
chr3	1700000	1950000	0,00044862	1	-

					0,037584452
chr3	1750000	2000000	0,005641602	1	-0,036847422
chr3	1800000	2050000	0,034074992	1	-0,036111814
chr3	2350000	2600000	0,012386732	1	-0,017711943
chr3	2400000	2650000	0,002015522	1	-0,017670068
chr3	2450000	2700000	0,015104672	1	-0,017171999
chr3	2500000	2750000	0,03535787	1	-0,017203509
chr3	2550000	2800000	0,041847944	1	-0,017599467
chr3	2600000	2850000	0,043068217	1	-0,018182133
chr3	3050000	3300000	0,012104503	1	-0,028556771
chr3	3100000	3350000	0,000777234	1	-0,030143254
chr3	3150000	3400000	0,002887609	1	-0,029447213
chr3	3200000	3450000	0,004154646	1	-0,02944788
chr3	3250000	3500000	0,001451461	1	-0,030546263
chr3	3300000	3550000	0,032541432	1	-0,031686678

chr3	3800000	4050000	0,007326551	1	-0,064215304
chr3	3850000	4100000	0,015211077	1	-0,067825928
chr3	4500000	4750000	0,027391986	1	-0,011652661
chr3	4550000	4800000	0,006299547	1	-0,010585443
chr3	4600000	4850000	0,009763578	1	-0,01050101
chr3	4650000	4900000	0,002863619	1	-0,010650603
chr3	4700000	4950000	0,002005618	1	-0,011005715
chr3	4750000	5000000	0,048500167	1	-0,011224088
chr3	5450000	5700000	0,006053492	1	-0,054914799
chr3	5500000	5750000	0,031964505	1	-0,051131985
chr3	5950000	6200000	0,024978826	1	-0,027297597
chr3	6000000	6250000	0,047829332	1	-0,026675662
chr3	6350000	6600000	0,030197416	1	-0,037714889
chr3	6400000	6650000	0,001926204	1	-0,040086097
chr3	6450000	6700000	0,012823031	1	-

					0,038530932
chr3	7950000	8200000	0,019775069	1	-0,028386545
chr3	8000000	8250000	0,00246028	1	-0,02977367
chr3	8050000	8300000	0,042179059	1	-0,030889007
chr3	9000000	9250000	0,012903869	1	-0,01842336
chr3	9050000	9300000	0,020305668	1	-0,019589475
chr3	14400000	14650000	0,008370302	1	-0,021833962
chr3	14450000	14700000	0,0034273	1	-0,020816922
chr3	16300000	16550000	0,004554976	1	-0,021388126
chr3	16350000	16600000	0,00083424	1	-0,02233284
chr3	16400000	16650000	0,009361633	1	-0,022894719
chr3	16450000	16700000	0,034434607	1	-0,022998479
chr3	16500000	16750000	0,043068217	1	-0,022652316
chr3	16550000	16800000	0,020128939	1	-0,02196712
chr3	16600000	16850000	0,002640189	1	-0,021143925
chr3	16650000	16900000	0,000209312	1	-0,020416913
chr3	16700000	16950000	0,001119112	1	-

					0,019973359
chr3	16750000	17000000	0,002749781	1	- 0,021143785
chr3	16800000	17050000	0,001957923	1	- 0,021285728
chr3	16850000	17100000	0,000546242	1	- 0,021352965
chr3	16900000	17150000	8,39E-05	1	- 0,021441416
chr3	16950000	17200000	3,55E-05	1	- 0,021629542
chr3	17000000	17250000	0,000152121	1	- 0,021939062
chr3	17050000	17300000	0,000762375	1	- 0,022326042
chr3	17100000	17350000	0,001965928	1	- 0,022701506
chr3	17150000	17400000	0,002335107	1	- 0,022962153
chr3	17200000	17450000	0,001087314	1	- 0,023008674
chr3	17250000	17500000	0,000138432	1	- 0,022753741
chr3	17300000	17550000	0,000428406	1	- 0,022107857
chr3	17350000	17600000	0,013451937	1	- 0,021517499
chr3	19600000	19850000	0,041716021	1	-

					0,035905044
chr3	20400000	20650000	0,003254203	1	- 0,040092219
chr3	20450000	20700000	0,001712793	1	- 0,038871173
chr3	20500000	20750000	0,013809713	1	- 0,037524627
chr3	20550000	20800000	0,038185025	1	- 0,036138171
chr3	21250000	21500000	0,004744754	1	- 0,013502905
chr3	21300000	21550000	0,000247929	1	- 0,013502905
chr3	21350000	21600000	0,003907523	1	- 0,013502905
chr3	21400000	21650000	0,026925794	1	- 0,013502905
chr3	22000000	22250000	0,005766304	1	- 0,023828717
chr3	22050000	22300000	0,005995564	1	- 0,024899905
chr3	26000000	26250000	0,019696446	1	- 0,039858969
chr3	26300000	26550000	0,035944422	1	- 0,030087602
chr3	26350000	26600000	0,014986898	1	- 0,030087602
chr3	26750000	27000000	0,012961185	1	-

					0,046686661
chr3	26800000	27050000	0,027509831	1	-0,049608079
chr3	28000000	28250000	0,008429529	1	-0,047394454
chr3	28050000	28300000	0,000485183	1	-0,046385691
chr3	28100000	28350000	0,002285375	1	-0,045576672
chr3	28150000	28400000	0,00327608	1	-0,04520821
chr3	28200000	28450000	0,001086486	1	-0,045294135
chr3	28250000	28500000	7,43E-05	1	-0,045653615
chr3	28300000	28550000	1,41E-05	1	-0,045976567
chr3	28350000	28600000	2,29E-05	1	-0,045896217
chr3	28400000	28650000	3,97E-05	1	-0,045044894
chr3	28450000	28700000	0,003174713	1	-0,044460222
chr3	31400000	31650000	0,019198869	1	-0,029475349
chr3	31450000	31700000	0,003026707	1	-0,028970846
chr3	34200000	34450000	0,01604223	1	-0,025497838

chr3	34250000	34500000	0,026082351	1	-<br>0,023966748
chr3	34600000	34850000	0,028336721	1	-<br>0,017528554
chr3	34650000	34900000	0,033401731	1	-<br>0,016749825
chr3	35350000	35600000	0,014025291	1	-<br>0,008291153
chr3	35400000	35650000	0,022796344	1	-<br>0,008291153
chr3	35650000	35900000	0,049090669	1	-<br>0,009811888
chr3	35700000	35950000	0,005040437	1	-<br>0,010439436
chr3	35750000	36000000	0,044754519	1	-<br>0,010941336
chr3	36450000	36700000	0,011291156	1	-<br>0,019569392
chr3	39700000	39950000	0,012903535	1	-0,03216247
chr3	40900000	41150000	0,011737094	1	-<br>0,038709072
chr3	41150000	41400000	0,020376996	1	-<br>0,033513118
chr3	41200000	41450000	0,001291366	1	-<br>0,031834756
chr3	41250000	41500000	0,000117726	1	-<br>0,030800707
chr3	41300000	41550000	0,002880522	1	-

					0,030392374
chr3	42950000	43200000	0,03710125	1	-0,018946964
chr3	44050000	44300000	0,004157291	1	-0,015155956
chr3	44100000	44350000	0,0464786	1	-0,015862248
chr3	46500000	46750000	0,007134452	1	-0,013741094
chr3	46550000	46800000	0,002617249	1	-0,01457953
chr3	53550000	53800000	0,04778627	1	-0,024842463
chr3	54350000	54600000	0,031004241	1	-0,061773508
chr3	54400000	54650000	0,006105117	1	-0,05952922
chr3	55700000	55950000	0,004358548	1	-0,020271386
chr3	55750000	56000000	0,047392893	1	-0,019080643
chr3	59550000	59800000	0,013381876	1	-0,033369119
chr3	59600000	59850000	0,000921285	1	-0,035039424
chr3	59650000	59900000	0,017728099	1	-0,036148031
chr3	62100000	62350000	0,025386775	1	-0,01344631
chr3	62350000	62600000	0,02699388	1	-0,012517836

chr3	62400000	62650000	0,021021719	1	-0,011733713
chr3	62650000	62900000	0,017916348	1	-0,013130923
chr3	62700000	62950000	0,003215158	1	-0,013698261
chr3	62750000	63000000	0,000245547	1	-0,014103243
chr3	62800000	63050000	0,001087527	1	-0,014383738
chr3	62850000	63100000	0,034703148	1	-0,014564201
chr3	63800000	64050000	0,040160488	1	-0,023399986
chr3	63850000	64100000	0,005693722	1	-0,023325891
chr3	63900000	64150000	0,00443484	1	-0,022084512
chr3	65500000	65750000	0,012589805	1	-0,031566457
chr3	65550000	65800000	0,023009137	1	-0,033391446
chr3	67200000	67450000	0,002329709	1	-0,033489923
chr3	67250000	67500000	0,001761198	1	-0,033489923
chr3	67300000	67550000	0,022951313	1	-0,033489923

chr3	68200000	68450000	0,021060493	1	-0,031947957
chr3	68250000	68500000	0,014984577	1	-0,031947957
chr3	68300000	68550000	0,019267859	1	-0,031947957
chr3	68350000	68600000	0,027674061	1	-0,031947957
chr3	68400000	68650000	0,030053625	1	-0,031947957
chr3	68450000	68700000	0,018343046	1	-0,032852413
chr3	68500000	68750000	0,004013455	1	-0,03390871
chr3	68550000	68800000	0,001203696	1	-0,035001522
chr3	68600000	68850000	0,038935707	1	-0,035914346
chr3	71100000	71350000	0,032482767	1	-0,011254621
chr3	71150000	71400000	0,010170543	1	-0,011254621
chr3	71350000	71600000	0,046134385	1	-0,010341946
chr3	71400000	71650000	0,011299537	1	-0,009830534
chr3	71450000	71700000	0,006876328	1	-0,009448112
chr3	71500000	71750000	0,042888512	1	-0,00922303

chr3	73550000	73800000	0,005008308	1	-0,0382295
chr3	73600000	73850000	0,002444462	1	-0,036714128
chr3	73650000	73900000	0,039118523	1	-0,034576537
chr3	74100000	74350000	0,030463911	1	-0,019406936
chr3	74150000	74400000	0,000667479	1	-0,019550727
chr3	74200000	74450000	9,51E-06	1	-0,019609031
chr3	74250000	74500000	1,38E-05	1	-0,019591206
chr3	74300000	74550000	2,74E-06	1	-0,019620188
chr3	74350000	74600000	1,05E-05	1	-0,019860508
chr3	74400000	74650000	0,000725083	1	-0,020433041
chr3	74450000	74700000	0,01155019	1	-0,020914293
chr3	74700000	74950000	0,029758245	1	-0,022501569
chr3	74750000	75000000	0,002513956	1	-0,023691217
chr3	74800000	75050000	0,00867421	1	-0,024666474
chr3	75050000	75300000	0,023970683	1	-

					0,021642401
chr3	75100000	75350000	0,017783656	1	-0,020308355
chr3	75400000	75650000	0,042164025	1	-0,025113139
chr3	75450000	75700000	0,005827463	1	-0,028066791
chr3	75500000	75750000	0,000318361	1	-0,02754138
chr3	75550000	75800000	0,002863619	1	-0,026382914
chr3	76750000	77000000	0,025474509	1	-0,026515167
chr3	76800000	77050000	0,002204443	1	-0,028002936
chr3	76850000	77100000	0,019318571	1	-0,029111928
chr3	77200000	77450000	0,04484273	1	-0,020884453
chr3	77250000	77500000	0,003062535	1	-0,019645442
chr3	77300000	77550000	6,44E-05	1	-0,018899235
chr3	77350000	77600000	0,000388093	1	-0,018547772
chr3	77400000	77650000	0,004360946	1	-0,019781365
chr3	77450000	77700000	0,021865478	1	-0,020627798

chr3	77950000	78200000	0,01870585	1	-0,032532845
chr3	78000000	78250000	0,001548949	1	-0,03210456
chr3	78050000	78300000	0,000695338	1	-0,031641643
chr3	78100000	78350000	0,016249553	1	-0,031370526
chr3	80450000	80700000	0,008812273	1	-0,031660043
chr3	81900000	82150000	0,023881268	1	-0,035200356
chr3	81950000	82200000	0,007321939	1	-0,033391694
chr3	83800000	84050000	0,01469661	1	-0,02029962
chr3	83850000	84100000	0,000847811	1	-0,020190703
chr3	83900000	84150000	0,00138319	1	-0,01956873
chr3	83950000	84200000	0,012023684	1	-0,018900224
chr3	84000000	84250000	0,028145515	1	-0,018388112
chr3	84050000	84300000	0,022042343	1	-0,018213136
chr3	84100000	84350000	0,00457488	1	-0,018495404
chr3	84150000	84400000	0,001812007	1	-0,01926517
chr3	84200000	84450000	0,034747552	1	-0,02030229
chr3	84500000	84750000	0,036340477	1	-

					0,023448183
chr3	84550000	84800000	0,002490802	1	-<br>0,024820601
chr3	84600000	84850000	0,002156693	1	-<br>0,025842921
chr3	84650000	84900000	0,034615865	1	-<br>0,026384265
chr3	85500000	85750000	0,035526557	1	-0,00624326
chr3	86300000	86550000	0,029274794	1	-<br>0,021204122
chr3	86350000	86600000	0,001162737	1	-<br>0,022748641
chr3	86400000	86650000	0,005671875	1	-<br>0,023607885
chr3	86450000	86700000	0,025631519	1	-<br>0,023824168
chr3	86500000	86750000	0,041537905	1	-<br>0,023519463
chr3	86550000	86800000	0,036663916	1	-<br>0,022872431
chr3	86600000	86850000	0,024084276	1	-<br>0,022085789
chr3	86650000	86900000	0,018876078	1	-<br>0,021334831
chr3	86700000	86950000	0,028939329	1	-<br>0,020713528
chr3	87050000	87300000	0,042157646	1	-<br>0,016079177

chr3	87450000	87700000	0,010385663	1	-0,02689724
chr3	87500000	87750000	0,000183511	1	-0,026688718
chr3	87550000	87800000	1,25E-05	1	-0,026611738
chr3	87600000	87850000	1,24E-05	1	-0,026742161
chr3	87650000	87900000	1,24E-05	1	-0,026742161
chr3	87700000	87950000	0,000181252	1	-0,026742161
chr3	87750000	88000000	0,000860542	1	-0,026742161
chr3	87800000	88050000	0,001582966	1	-0,026742161
chr3	87850000	88100000	0,001795088	1	-0,027047406
chr3	87900000	88150000	0,001880161	1	-0,029105614
chr3	87950000	88200000	0,00253434	1	-0,029676869
chr3	88000000	88250000	0,004655603	1	-0,030398875
chr3	88050000	88300000	0,009584664	1	-0,031272532
chr3	88100000	88350000	0,017944757	1	-0,032257304
chr3	88150000	88400000	0,027147724	1	-

					0,033279593
chr3	88200000	88450000	0,031390527	1	-0,034249925
chr3	88250000	88500000	0,02736015	1	-0,03509381
chr3	88300000	88550000	0,017943798	1	-0,035774331
chr3	88350000	88600000	0,009066593	1	-0,036298526
chr3	88400000	88650000	0,003714122	1	-0,036703046
chr3	88450000	88700000	0,001333132	1	-0,037016375
chr3	88500000	88750000	0,000436079	1	-0,037232419
chr3	88550000	88800000	0,000106628	1	-0,037293031
chr3	88600000	88850000	1,06E-05	1	-0,037103336
chr3	88650000	88900000	2,45E-05	1	-0,036569787
chr3	88700000	88950000	0,000838436	1	-0,035645108
chr3	88750000	89000000	0,007947014	1	-0,034366937
chr3	88800000	89050000	0,033699102	1	-0,032880106
chr3	89000000	89250000	0,022796579	1	-0,029794499

chr3	89050000	89300000	0,001934332	1	-0,030611936
chr3	89100000	89350000	0,005442905	1	-0,031452752
chr3	89150000	89400000	0,048513916	1	-0,031817109
chr3	89400000	89650000	0,037054654	1	-0,035285425
chr3	89450000	89700000	0,01368658	1	-0,03681366
chr3	89500000	89750000	0,003204388	1	-0,038116814
chr3	89550000	89800000	0,000279192	1	-0,039186079
chr3	89600000	89850000	0,000241608	1	-0,040008478
chr3	89650000	89900000	0,004859204	1	-0,040522964
chr3	89700000	89950000	0,028565558	1	-0,040618631
chr3	90000000	90250000	0,049029975	1	-0,033218969
chr3	94000000	94250000	0,011929432	1	-0,042005066
chr3	94050000	94300000	0,002649573	1	-0,040277516
chr3	94100000	94350000	0,038382326	1	-0,037943956
chr3	94500000	94750000	0,019327242	1	-

					0,025685425
chr3	94550000	94800000	0,003862073	1	- 0,025276512
chr3	94600000	94850000	0,003965332	1	- 0,024516633
chr3	94650000	94900000	0,022096721	1	- 0,023271989
chr3	95150000	95400000	0,041740856	1	- 0,012643034
chr3	95450000	95700000	0,008615677	1	- 0,015646401
chr3	95500000	95750000	0,009022659	1	- 0,014850987
chr3	95700000	95950000	0,016626942	1	- 0,013495437
chr3	96150000	96400000	0,040468114	1	- 0,025559177
chr3	96200000	96450000	0,007441242	1	- 0,026822959
chr3	96600000	96850000	0,022144554	1	- 0,017532536
chr3	97000000	97250000	0,007622971	1	- 0,025410824
chr3	97050000	97300000	0,03946306	1	- 0,026790336
chr3	97550000	97800000	0,019821101	1	- 0,007950246
chr3	97700000	97950000	0,027421807	1	-

					0,008375489
					-
chr3	97750000	98000000	0,009854722	1	0,007800257
					-
chr3	103750000	104000000	0,029202141	1	0,015608631
					-
chr3	103800000	104050000	0,002164611	1	0,016657186
					-
chr3	103850000	104100000	0,035560424	1	0,017306796
					-
chr3	107000000	107250000	0,004011036	1	0,032129162
					-
chr3	107050000	107300000	0,026181585	1	0,029886341
					-
chr3	110650000	110900000	0,02532364	1	0,039047344
					-
chr3	110700000	110950000	0,002042288	1	0,041675686
					-
chr3	110750000	111000000	0,026017862	1	0,043257228
					-
chr3	115850000	116100000	0,015230497	1	0,017463293
					-
chr3	115900000	116150000	0,042013008	1	0,016259641
					-
chr3	117350000	117600000	0,033353921	1	0,028184577
					-
chr3	117400000	117650000	0,00273861	1	0,029852663
chr3	117450000	117700000	5,92E-05	1	-

					0,030970121
chr3	117500000	117750000	6,24E-06	1	- 0,031563092
chr3	117550000	117800000	1,66E-05	1	- 0,031741887
chr3	117600000	117850000	5,77E-06	1	- 0,031658528
chr3	117650000	117900000	3,82E-06	1	- 0,031467611
chr3	117700000	117950000	0,000110336	1	- 0,031296909
chr3	117750000	118000000	0,00109672	1	- 0,031234781
chr3	117800000	118050000	0,005232166	1	- 0,031332674
chr3	117850000	118100000	0,017145541	1	- 0,031614675
chr3	117900000	118150000	0,044788285	1	- 0,032093056
chr3	118200000	118450000	0,039349053	1	- 0,040592778
chr3	118250000	118500000	0,003905293	1	- 0,042761544
chr3	118300000	118550000	0,018180977	1	- 0,044625593
chr3	118800000	119050000	0,038567035	1	- 0,016210639
chr3	118850000	119100000	0,000437045	1	-

					0,014868613
chr3	118900000	119150000	0,000925948	1	-0,014392393
chr3	118950000	119200000	0,001573103	1	-0,014436034
chr3	119000000	119250000	0,005012472	1	-0,015842411
chr3	119050000	119300000	0,04951444	1	-0,016950933
chr3	119400000	119650000	0,036336007	1	-0,023271892
chr3	120650000	120900000	0,040949263	1	-0,023140398
chr3	120700000	120950000	0,006906629	1	-0,023140398
chr3	120750000	121000000	0,005582082	1	-0,023140398
chr3	120800000	121050000	0,011970881	1	-0,023140398
chr3	120850000	121100000	0,019867726	1	-0,023465829
chr3	120900000	121150000	0,016320277	1	-0,0239278
chr3	120950000	121200000	0,004845639	1	-0,024591434
chr3	121000000	121250000	0,000489852	1	-0,02536082
chr3	121050000	121300000	0,003201967	1	-0,02603254
chr3	121100000	121350000	0,038204642	1	-0,026390792

chr3	121650000	121900000	0,016494158	1	-0,011041892
chr3	121700000	121950000	0,000213715	1	-0,011041892
chr3	121750000	122000000	0,000387866	1	-0,011041892
chr3	123250000	123500000	0,027571598	1	-0,027899644
chr3	123700000	123950000	0,009998725	1	-0,015031767
chr3	123750000	124000000	0,000597393	1	-0,015283795
chr3	123800000	124050000	0,001062839	1	-0,015264889
chr3	123850000	124100000	0,000609273	1	-0,015264889
chr3	123900000	124150000	0,025896196	1	-0,015264889
chr3	125750000	126000000	0,027296564	1	-0,025181452
chr3	125800000	126050000	0,00143916	1	-0,025454681
chr3	125850000	126100000	0,002256826	1	-0,026401228
chr3	125900000	126150000	0,029735191	1	-0,026861525
chr3	126450000	126700000	0,0135075	1	-0,013435458

chr3	126500000	126750000	0,000716456	1	-0,013716433
chr3	126550000	126800000	0,001157926	1	-0,013750405
chr3	126600000	126850000	0,000781026	1	-0,013319341
chr3	126650000	126900000	0,024014286	1	-0,012578057
chr3	134650000	134900000	0,021068725	1	-0,040251608
chr3	134700000	134950000	0,003798707	1	-0,038669731
chr3	134750000	135000000	0,028698021	1	-0,037093502
chr3	134850000	135100000	0,04532026	1	-0,035520451
chr3	134900000	135150000	0,009766155	1	-0,035520451
chr3	134950000	135200000	0,000897371	1	-0,035520451
chr3	135000000	135250000	0,00603403	1	-0,035520451
chr3	135050000	135300000	0,027471585	1	-0,035520451
chr3	135100000	135350000	0,032170742	1	-0,035885793
chr3	135150000	135400000	0,010145482	1	-0,036925095

chr3	135200000	135450000	0,002096577	1	-0,038322574
chr3	135250000	135500000	0,041513513	1	-0,039616051
chr3	137100000	137350000	0,010542191	1	-0,052346065
chr3	137150000	137400000	0,0382733	1	-0,052346065
chr3	139400000	139650000	0,005234379	1	-0,03174115
chr3	140350000	140600000	0,019642805	1	-0,037607463
chr3	142900000	143150000	0,034696132	1	-0,035962118
chr3	142950000	143200000	0,009795409	1	-0,033868123
chr3	143400000	143650000	0,021463827	1	-0,018895354
chr3	143450000	143700000	0,001843284	1	-0,019570426
chr3	143500000	143750000	0,01768017	1	-0,020429386
chr3	143700000	143950000	0,014135119	1	-0,02232906
chr3	143750000	144000000	0,001463063	1	-0,021624272
chr3	143800000	144050000	0,015501632	1	-0,020314677
chr3	144950000	145200000	0,045395827	1	-0,017759411

chr3	145000000	145250000	0,004519798	1	-0,018749101
chr3	145050000	145300000	0,00242993	1	-0,019553479
chr3	146400000	146650000	0,027040121	1	-0,021942589
chr3	146450000	146700000	0,038795609	1	-0,020246336
chr3	148050000	148300000	0,041357198	1	-0,016609906
chr3	148100000	148350000	0,01157743	1	-0,017706777
chr3	150600000	150850000	0,011008426	1	-0,012500001
chr3	150650000	150900000	0,009296421	1	-0,012008307
chr3	152450000	152700000	0,024561639	1	-0,014824124
chr3	152500000	152750000	0,000673916	1	-0,015842314
chr3	152550000	152800000	0,001649545	1	-0,016400321
chr3	152600000	152850000	0,011766089	1	-0,016552307
chr3	152650000	152900000	0,043293614	1	-0,016407253
chr3	153950000	154200000	0,013680521	1	-0,038948789

chr3	154450000	154700000	0,013125301	1	-0,019184057
chr3	154500000	154750000	0,001459161	1	-0,019184057
chr3	154550000	154800000	0,019505602	1	-0,019184057
chr3	154900000	155150000	0,035959361	1	-0,025634602
chr3	154950000	155200000	0,013489979	1	-0,027316539
chr3	157900000	158150000	0,033882789	1	-0,034775611
chr3	161450000	161700000	0,02486656	1	-0,035483307
chr3	161500000	161750000	0,001956562	1	-0,034906931
chr3	161550000	161800000	0,00105126	1	-0,03376594
chr3	161600000	161850000	0,016900861	1	-0,032190964
chr3	163200000	163450000	0,006857255	1	-0,03106202
chr3	163250000	163500000	0,006688317	1	-0,029620102
chr3	163500000	163750000	0,017871672	1	-0,026411342
chr3	163550000	163800000	0,000852918	1	-0,026251222
chr3	163600000	163850000	4,75E-05	1	-0,025823928

chr3	163650000	163900000	7,55E-05	1	-0,025476829
chr3	163700000	163950000	5,73E-05	1	-0,025539778
chr3	163750000	164000000	0,002315433	1	-0,024547063
chr3	163800000	164050000	0,040144535	1	-0,023046338
chr3	164450000	164700000	0,012513855	1	-0,025881121
chr3	164500000	164750000	0,017692589	1	-0,027713859
chr3	164700000	164950000	0,029351474	1	-0,025638848
chr3	164750000	165000000	0,001966426	1	-0,02428463
chr3	164800000	165050000	0,006996558	1	-0,02333032
chr3	164850000	165100000	0,039496947	1	-0,022942706
chr3	164950000	165200000	0,029527345	1	-0,023847148
chr3	165000000	165250000	0,003949998	1	-0,024851348
chr3	165050000	165300000	0,004422434	1	-0,025853999
chr3	165350000	165600000	0,042929742	1	-0,021458765
chr3	165400000	165650000	0,003271725	1	-0,02007214
chr3	165450000	165700000	0,027807076	1	-

					0,021182968
chr3	165700000	165950000	0,048742512	1	- 0,024059453
chr3	165750000	166000000	0,004523223	1	-0,02430922
chr3	165800000	166050000	0,001548562	1	- 0,024864338
chr3	165850000	166100000	0,019773266	1	-0,02550125
chr3	166100000	166350000	0,007551704	1	- 0,021999331
chr3	166150000	166400000	0,00059354	1	-0,02107536
chr3	166200000	166450000	0,003704996	1	- 0,020485725
chr3	166250000	166500000	0,011414604	1	- 0,020309533
chr3	166300000	166550000	0,007488659	1	- 0,020543305
chr3	166350000	166600000	0,001260468	1	- 0,021078035
chr3	166400000	166650000	0,008781038	1	- 0,021706183
chr3	168300000	168550000	0,034171496	1	-0,00579973
chr3	172850000	173100000	0,007959912	1	- 0,041164208
chr3	172900000	173150000	0,01545362	1	- 0,038745637
chr3	173300000	173550000	0,015255616	1	- 0,026049552
chr3	173350000	173600000	0,000569198	1	-

					0,025882435
chr3	173400000	173650000	0,002631321	1	-0,025094773
chr3	173450000	173700000	0,008035183	1	-0,024957312
chr3	173500000	173750000	0,0079351	1	-0,025282875
chr3	173550000	173800000	0,00329335	1	-0,02586231
chr3	173600000	173850000	0,000467175	1	-0,026506941
chr3	173650000	173900000	4,89E-05	1	-0,027070484
chr3	173700000	173950000	0,000690641	1	-0,027452366
chr3	173750000	174000000	0,004864679	1	-0,027590973
chr3	173800000	174050000	0,010553023	1	-0,027452381
chr3	173850000	174100000	0,007575988	1	-0,027035863
chr3	173900000	174150000	0,001356278	1	-0,02640163
chr3	173950000	174200000	0,003190695	1	-0,02569376
chr3	174000000	174250000	0,046940211	1	-0,025146923
chr3	174150000	174400000	0,043602331	1	-0,029802999
chr3	174200000	174450000	0,023266504	1	-0,027343997

chr3	175700000	175950000	0,038136045	1	-0,014736355
chr3	178450000	178700000	0,01100548	1	-0,032515183
chr3	178500000	178750000	0,015954016	1	-0,034975766
chr3	179800000	180050000	0,004034773	1	-0,030341059
chr3	179850000	180100000	0,005498174	1	-0,029152165
chr3	179900000	180150000	0,03199704	1	-0,028087828
chr3	179950000	180200000	0,049766426	1	-0,028087828
chr3	180000000	180250000	0,026139323	1	-0,028087828
chr3	180050000	180300000	0,003972417	1	-0,028087828
chr3	180100000	180350000	0,000149142	1	-0,028087828
chr3	180150000	180400000	0,000155809	1	-0,027429982
chr3	180200000	180450000	0,000241035	1	-0,027260985
chr3	180250000	180500000	5,44E-05	1	-0,027469074
chr3	180300000	180550000	0,000119669	1	-0,027827878

chr3	180350000	180600000	0,001381226	1	- 0,028098377
chr3	180400000	180650000	0,004296027	1	-0,02811472
chr3	180450000	180700000	0,005065025	1	- 0,027831487
chr3	180500000	180750000	0,002793382	1	- 0,025667767
chr3	180550000	180800000	0,000856945	1	- 0,025509295
chr3	180600000	180850000	0,00026268	1	- 0,025282069
chr3	180650000	180900000	0,000296176	1	- 0,024920518
chr3	180700000	180950000	0,000937572	1	- 0,024458423
chr3	180750000	181000000	0,002388463	1	- 0,024013511
chr3	180800000	181050000	0,002982083	1	- 0,023736289
chr3	180850000	181100000	0,001360783	1	- 0,023730976
chr3	180900000	181150000	0,000159888	1	- 0,023992681
chr3	180950000	181200000	0,000106794	1	- 0,023992681
chr3	181000000	181250000	0,00059218	1	- 0,023992681
chr3	181050000	181300000	0,000494396	1	-

					0,023992681
chr3	181100000	181350000	0,000268483	1	- 0,023992681
chr3	181150000	181400000	0,009496641	1	- 0,024385187
chr3	181450000	181700000	0,041209873	1	- 0,019391177
chr3	181950000	182200000	0,014260755	1	- 0,037375591
chr3	182000000	182250000	0,016443919	1	- 0,039647629
chr3	186750000	187000000	0,029359238	1	- 0,011886725
chr3	186950000	187200000	0,008069814	1	- 0,011071905
chr3	187000000	187250000	0,000370273	1	- 0,011191167
chr3	187050000	187300000	0,00243768	1	- 0,011191167
chr3	187100000	187350000	0,015095467	1	- 0,011191167
chr3	187150000	187400000	0,039463321	1	- 0,011191167
chr3	187300000	187550000	0,046353416	1	- 0,012065478
chr3	187350000	187600000	0,012517259	1	- 0,012550317
chr3	187400000	187650000	0,001465608	1	-

					0,013055808
chr3	187450000	187700000	0,012945036	1	- 0,012805585
chr3	187650000	187900000	0,045599036	1	- 0,012429972
chr3	188100000	188350000	0,023212228	1	- 0,031262819
chr3	188850000	189100000	0,001828937	1	- 0,021481862
chr3	188900000	189150000	0,004109122	1	- 0,021140346
chr3	188950000	189200000	0,004233534	1	- 0,021102842
chr3	189000000	189250000	0,000654323	1	- 0,021327425
chr3	189050000	189300000	3,15E-05	1	- 0,021451843
chr3	189100000	189350000	2,19E-05	1	- 0,021147509
chr3	189150000	189400000	0,002108286	1	- 0,021147509
chr3	192950000	193200000	0,02080928	1	- 0,024039591
chr3	193400000	193650000	0,043038219	1	- 0,014672359
chr3	193450000	193700000	0,006012189	1	- 0,013790309
chr3	193500000	193750000	0,007785169	1	-

					0,013304445
chr4	3800000	4050000	0,00882073	1	-<br>0,020511606
chr4	3850000	4100000	0,010703526	1	-0,01963775
chr4	5200000	5450000	0,010478335	1	-<br>0,030390855
chr4	5550000	5800000	0,0024777	1	-<br>0,023960084
chr4	5600000	5850000	0,000587768	1	-<br>0,022814057
chr4	5650000	5900000	0,001163212	1	-<br>0,022511189
chr4	10500000	10750000	0,005119634	1	-<br>0,020468643
chr4	10550000	10800000	4,50E-05	1	-<br>0,020749819
chr4	10600000	10850000	0,000699401	1	-0,02133426
chr4	10650000	10900000	0,008499941	1	-<br>0,022134791
chr4	10700000	10950000	0,034170281	1	-<br>0,023030453
chr4	11200000	11450000	0,016809646	1	-<br>0,030388639
chr4	11250000	11500000	0,00124059	1	-<br>0,031901593
chr4	11300000	11550000	0,007794874	1	-<br>0,031944414
chr4	11850000	12100000	0,036898538	1	-

					0,017866271
chr4	11900000	12150000	0,013823364	1	-0,017102405
chr4	11950000	12200000	0,003604634	1	-0,016549212
chr4	12000000	12250000	0,000401129	1	-0,01611839
chr4	12050000	12300000	0,00590607	1	-0,015773439
chr4	13100000	13350000	0,025362202	1	-0,074714817
chr4	13150000	13400000	0,002111013	1	-0,079764858
chr4	13200000	13450000	0,020224693	1	-0,08285245
chr4	15100000	15350000	0,002732882	1	-0,016702668
chr4	15150000	15400000	0,028780428	1	-0,017432965
chr4	15850000	16100000	0,028551672	1	-0,011105119
chr4	16600000	16850000	0,015773071	1	-0,025002945
chr4	16650000	16900000	0,001742102	1	-0,025417179
chr4	16700000	16950000	0,003716318	1	-0,026336977
chr4	16750000	17000000	0,034122352	1	-0,026336977
chr4	17050000	17300000	0,029096721	1	-

					0,032405413
chr4	17100000	17350000	0,015263802	1	- 0,034628896
chr4	18150000	18400000	0,01525262	1	- 0,036975982
chr4	18200000	18450000	0,024260818	1	- 0,034523676
chr4	18800000	19050000	0,032603264	1	- 0,014254776
chr4	18850000	19100000	0,002361604	1	- 0,014254776
chr4	18900000	19150000	0,008483718	1	- 0,014254776
chr4	19350000	19600000	0,008087302	1	- 0,024602759
chr4	19400000	19650000	0,00256106	1	- 0,023581459
chr4	19450000	19700000	0,039256751	1	- 0,022600509
chr4	19800000	20050000	0,009761911	1	- 0,017973623
chr4	19850000	20100000	0,00243717	1	- 0,017517443
chr4	19900000	20150000	0,036186157	1	- 0,018519776
chr4	20150000	20400000	0,010193714	1	-0,02093235
chr4	20200000	20450000	0,001115882	1	-0,02093235
chr4	20250000	20500000	0,007776449	1	-

					0,021318559
chr4	20300000	20550000	0,021600429	1	-<br>0,021480047
chr4	20350000	20600000	0,013676316	1	-<br>0,021149538
chr4	20400000	20650000	0,001969315	1	-<br>0,020499957
chr4	20450000	20700000	0,005105218	1	-<br>0,019810915
chr4	20500000	20750000	0,048684427	1	-<br>0,019374195
chr4	20650000	20900000	0,032013555	1	-0,02325538
chr4	20700000	20950000	0,003512633	1	-<br>0,022379463
chr4	20750000	21000000	0,029006079	1	-<br>0,020787715
chr4	23600000	23850000	0,040866126	1	-<br>0,016668963
chr4	23650000	23900000	0,001512414	1	-<br>0,017864752
chr4	23700000	23950000	0,001139457	1	-<br>0,018596339
chr4	23750000	24000000	0,007381106	1	-0,01888514
chr4	23800000	24050000	0,013090067	1	-0,01879204
chr4	23850000	24100000	0,008829158	1	-0,01842632
chr4	23900000	24150000	0,002321833	1	-<br>0,017934388
chr4	23950000	24200000	0,000173678	1	-

					0,017465224
chr4	24000000	24250000	7,49E-06	1	-0,017126073
chr4	24050000	24300000	5,86E-05	1	-0,016957572
chr4	24100000	24350000	0,00031146	1	-0,016939848
chr4	24150000	24400000	0,001037845	1	-0,017889947
chr4	24200000	24450000	0,003261984	1	-0,018333504
chr4	24250000	24500000	0,009430253	1	-0,018902114
chr4	24300000	24550000	0,022293064	1	-0,019574153
chr4	24350000	24600000	0,040068789	1	-0,02030219
chr4	24500000	24750000	0,033160827	1	-0,022083771
chr4	24550000	24800000	0,012770651	1	-0,022268895
chr4	24600000	24850000	0,002041007	1	-0,022150674
chr4	24650000	24900000	0,00016217	1	-0,021737824
chr4	24700000	24950000	0,001404954	1	-0,021112292
chr4	24750000	25000000	0,010378736	1	-0,021112292

chr4	24800000	25050000	0,023361398	1	-0,021112292
chr4	24850000	25100000	0,019144249	1	-0,021112292
chr4	24900000	25150000	0,004380875	1	-0,021112292
chr4	24950000	25200000	0,00128837	1	-0,020427809
chr4	25000000	25250000	0,020842647	1	-0,019881676
chr4	25200000	25450000	0,013258918	1	-0,023337767
chr4	25250000	25500000	0,012903422	1	-0,021809133
chr4	27400000	27650000	0,043899376	1	-0,030288716
chr4	27450000	27700000	0,003304765	1	-0,029904906
chr4	27500000	27750000	0,000330916	1	-0,029236721
chr4	27550000	27800000	0,002934756	1	-0,028474692
chr4	27600000	27850000	0,009629503	1	-0,02779668
chr4	27650000	27900000	0,01114772	1	-0,027354582
chr4	27700000	27950000	0,005109152	1	-0,027247224
chr4	27750000	28000000	0,000683545	1	-

					0,027494018
chr4	27800000	28050000	0,000116176	1	-0,028003968
chr4	27850000	28100000	0,001149753	1	-0,028606157
chr4	27900000	28150000	0,0038101	1	-0,029120681
chr4	27950000	28200000	0,004042995	1	-0,029451613
chr4	28000000	28250000	0,001856604	1	-0,02963954
chr4	28050000	28300000	0,000500076	1	-0,029834929
chr4	28100000	28350000	0,000241119	1	-0,030203548
chr4	28150000	28400000	0,000706114	1	-0,030813927
chr4	28200000	28450000	0,003019998	1	-0,031579828
chr4	28250000	28500000	0,007105829	1	-0,032290193
chr4	28300000	28550000	0,007361221	1	-0,032707498
chr4	28350000	28600000	0,002711479	1	-0,032673028
chr4	28400000	28650000	0,000277044	1	-0,032170569
chr4	28450000	28700000	0,000644934	1	-0,031308208

chr4	28500000	28750000	0,0075957	1	-0,030241911
chr4	28550000	28800000	0,029072324	1	-0,029099426
chr4	28900000	29150000	0,024097323	1	-0,023050322
chr4	28950000	29200000	0,003694018	1	-0,023111802
chr4	29000000	29250000	0,00013925	1	-0,023237413
chr4	29050000	29300000	1,40E-06	1	-0,023235982
chr4	29100000	29350000	4,18E-06	1	-0,022976999
chr4	29150000	29400000	0,000298309	1	-0,022485429
chr4	29200000	29450000	0,004727114	1	-0,021938378
chr4	29250000	29500000	0,023560455	1	-0,021393926
chr4	29450000	29700000	0,04670484	1	-0,01927552
chr4	29500000	29750000	0,020542819	1	-0,018907704
chr4	29550000	29800000	0,005434057	1	-0,018629813
chr4	29600000	29850000	0,000578272	1	-0,01841347
chr4	29650000	29900000	5,80E-05	1	-0,018228842

chr4	29700000	29950000	0,000904033	1	-0,01801678
chr4	29750000	30000000	0,006139622	1	-0,017933784
chr4	29800000	30050000	0,014778752	1	-0,018037851
chr4	29850000	30100000	0,016592363	1	-0,018330044
chr4	29900000	30150000	0,008762858	1	-0,018783188
chr4	29950000	30200000	0,001751122	1	-0,01991178
chr4	30000000	30250000	9,75E-05	1	-0,020127695
chr4	30050000	30300000	7,10E-05	1	-0,019901397
chr4	30100000	30350000	0,000198185	1	-0,019825152
chr4	30150000	30400000	7,78E-05	1	-0,019994877
chr4	30200000	30450000	0,000119912	1	-0,020411683
chr4	30250000	30500000	0,002057374	1	-0,020597079
chr4	30300000	30550000	0,008442673	1	-0,020597079
chr4	30350000	30600000	0,010389628	1	-0,020597079
chr4	30400000	30650000	0,00370781	1	-0,020597079

chr4	30450000	30700000	0,000581618	1	-0,020970542
chr4	30500000	30750000	0,009811428	1	-0,021500098
chr4	31350000	31600000	0,042617545	1	-0,027035837
chr4	31400000	31650000	0,001514099	1	-0,029096679
chr4	31450000	31700000	0,002360877	1	-0,030334488
chr4	31500000	31750000	0,007738281	1	-0,030671274
chr4	31550000	31800000	0,004776219	1	-0,030027691
chr4	31600000	31850000	0,000720005	1	-0,029424113
chr4	31650000	31900000	0,004227087	1	-0,028889104
chr4	31700000	31950000	0,04019047	1	-0,028410854
chr4	31850000	32100000	0,046930773	1	-0,030445531
chr4	31900000	32150000	0,006028022	1	-0,031968086
chr4	31950000	32200000	0,002360877	1	-0,033403321
chr4	32000000	32250000	0,033734356	1	-0,034372043

chr4	32200000	32450000	0,042434555	1	-0,030727493
chr4	32250000	32500000	0,003513646	1	-0,028966905
chr4	32300000	32550000	0,017376756	1	-0,027682778
chr4	32500000	32750000	0,007699373	1	-0,030029583
chr4	32550000	32800000	0,007057197	1	-0,031472711
chr4	33050000	33300000	0,003756989	1	-0,016439641
chr4	33100000	33350000	9,67E-05	1	-0,015809971
chr4	33150000	33400000	7,43E-07	1	-0,015527565
chr4	33200000	33450000	2,08E-06	1	-0,015427336
chr4	33250000	33500000	9,47E-06	1	-0,015406887
chr4	33300000	33550000	5,21E-06	1	-0,015426713
chr4	33350000	33600000	4,43E-05	1	-0,015482366
chr4	33400000	33650000	0,004533544	1	-0,015565348
chr4	34600000	34850000	0,021288312	1	-0,022351461

chr4	34650000	34900000	0,000655609	1	-0,022176228
chr4	34700000	34950000	3,02E-05	1	-0,021933318
chr4	34750000	35000000	0,000165015	1	-0,021727003
chr4	34800000	35050000	0,000212413	1	-0,021667155
chr4	34850000	35100000	4,79E-05	1	-0,0218572
chr4	34900000	35150000	0,000180943	1	-0,022375064
chr4	34950000	35200000	0,005178207	1	-0,022625284
chr4	35000000	35250000	0,03870214	1	-0,022625284
chr4	35400000	35650000	0,033029809	1	-0,030363152
chr4	35450000	35700000	0,005574227	1	-0,031700024
chr4	35500000	35750000	0,001489345	1	-0,032878424
chr4	35550000	35800000	0,047367409	1	-0,033784424
chr4	36600000	36850000	0,001678133	1	-0,027909683
chr4	36650000	36900000	1,36E-05	1	-0,028113803
chr4	36700000	36950000	0,000120586	1	-

					0,028113803
					-
chr4	36750000	37000000	0,002595975	1	0,028113803
					-
chr4	36800000	37050000	0,014814477	1	0,028113803
					-
chr4	36850000	37100000	0,030974891	1	0,028113803
					-
chr4	36900000	37150000	0,026239744	1	0,028689711
chr4	36950000	37200000	0,00737699	1	-0,02959659
					-
chr4	37000000	37250000	0,001070803	1	0,030649328
					-
chr4	37050000	37300000	0,018155743	1	0,031568405
					-
chr4	38200000	38450000	0,046260625	1	0,022506534
					-
chr4	38250000	38500000	0,006715172	1	0,022506534
					-
chr4	41600000	41850000	0,016483663	1	0,010377616
					-
chr4	41650000	41900000	0,017566982	1	0,010161396
chr4	41700000	41950000	0,013878436	1	-0,01047125
					-
chr4	41750000	42000000	0,02623901	1	0,011391825
					-
chr4	42600000	42850000	0,025641405	1	0,040976109
chr4	42650000	42900000	0,009454514	1	-

					0,038519832
chr4	43500000	43750000	0,027570725	1	-0,006258306
chr4	44100000	44350000	0,016322082	1	-0,020928826
chr4	44150000	44400000	0,003221629	1	-0,021841972
chr4	44200000	44450000	0,000621631	1	-0,022463793
chr4	44250000	44500000	0,000135206	1	-0,022883067
chr4	44300000	44550000	5,05E-05	1	-0,023161171
chr4	44350000	44600000	4,37E-05	1	-0,023342533
chr4	44400000	44650000	5,05E-05	1	-0,023468964
chr4	44450000	44700000	3,21E-05	1	-0,023585784
chr4	44500000	44750000	5,94E-06	1	-0,02372336
chr4	44550000	44800000	4,58E-05	1	-0,023879963
chr4	44600000	44850000	0,001370712	1	-0,024015565
chr4	44650000	44900000	0,011298191	1	-0,021991394
chr4	44700000	44950000	0,041943506	1	-0,021032047

chr4	44900000	45150000	0,023698246	1	-0,019418851
chr4	44950000	45200000	0,002523551	1	-0,019418851
chr4	45000000	45250000	5,33E-05	1	-0,019418851
chr4	45050000	45300000	8,32E-06	1	-0,019026392
chr4	45100000	45350000	0,003553548	1	-0,018959035
chr4	45900000	46150000	0,032414292	1	-0,011597297
chr4	46850000	47100000	0,016475759	1	-0,028015202
chr4	46900000	47150000	0,009833335	1	-0,029914346
chr4	47400000	47650000	0,013939559	1	-0,012071778
chr4	47450000	47700000	0,00060996	1	-0,011181776
chr4	47500000	47750000	0,001330427	1	-0,010949621
chr4	52950000	53200000	0,007750204	1	-0,028673325
chr4	55550000	55800000	0,014774302	1	-0,028967131
chr4	57300000	57550000	0,010571669	1	-0,021298808

chr4	57350000	57600000	0,000815699	1	-0,021427674
chr4	57400000	57650000	0,000809002	1	-0,022037897
chr4	57450000	57700000	0,013937655	1	-0,022405808
chr4	57650000	57900000	0,021761848	1	-0,020469311
chr4	57700000	57950000	0,014221198	1	-0,019460325
chr4	58250000	58500000	0,049519642	1	-0,059312385
chr4	58300000	58550000	0,002117468	1	-0,057721916
chr4	58350000	58600000	0,005078772	1	-0,055466403
chr4	58400000	58650000	0,038280514	1	-0,052928554
chr4	59100000	59350000	0,049235662	1	-0,026212663
chr4	59150000	59400000	0,030010935	1	-0,025693073
chr4	59200000	59450000	0,011204203	1	-0,025579065
chr4	59250000	59500000	0,001390388	1	-0,025990945
chr4	59300000	59550000	0,001298899	1	-0,026877786

chr4	59350000	59600000	0,02430139	1	-0,027658257
chr4	59650000	59900000	0,01313535	1	-0,03321401
chr4	59700000	59950000	0,000908633	1	-0,033836809
chr4	59750000	60000000	0,006067625	1	-0,032413306
chr4	59800000	60050000	0,048499159	1	-0,030773771
chr4	60050000	60300000	0,010387109	1	-0,026952597
chr4	60100000	60350000	0,000440101	1	-0,027296799
chr4	60150000	60400000	0,000127949	1	-0,027657543
chr4	60200000	60450000	0,000402207	1	-0,027824033
chr4	60250000	60500000	0,000188656	1	-0,02767935
chr4	60300000	60550000	4,60E-05	1	-0,027456559
chr4	60350000	60600000	0,000362347	1	-0,027456559
chr4	60400000	60650000	0,001022922	1	-0,027456559
chr4	60450000	60700000	0,000433382	1	-0,027456559
chr4	60500000	60750000	0,000835317	1	-0,027266308

chr4	60550000	60800000	0,019395051	1	-0,026797814
chr4	60800000	61050000	0,010877295	1	-0,032549679
chr4	60850000	61100000	0,000471716	1	-0,033187797
chr4	60900000	61150000	0,000537023	1	-0,032512689
chr4	60950000	61200000	0,002834907	1	-0,031910442
chr4	61000000	61250000	0,004198741	1	-0,031473788
chr4	61050000	61300000	0,002725321	1	-0,031170654
chr4	61100000	61350000	0,001168312	1	-0,030869539
chr4	61150000	61400000	0,000810322	1	-0,030386663
chr4	61200000	61450000	0,002399348	1	-0,029698157
chr4	61250000	61500000	0,014028591	1	-0,028722193
chr4	61600000	61850000	0,006210004	1	-0,021981971
chr4	61650000	61900000	0,00042042	1	-0,022415971
chr4	61700000	61950000	0,001162928	1	-0,02259772
chr4	61750000	62000000	0,000678966	1	-

					0,022450354
chr4	61800000	62050000	0,000119665	1	-0,02207061
chr4	61850000	62100000	0,000481577	1	-0,021696784
chr4	61900000	62150000	0,001097777	1	-0,021595198
chr4	61950000	62200000	0,000379752	1	-0,021943298
chr4	62000000	62250000	0,001324359	1	-0,022461743
chr4	62050000	62300000	0,024733433	1	-0,022729292
chr4	62400000	62650000	0,029210267	1	-0,028293505
chr4	62450000	62700000	0,005504153	1	-0,029553394
chr4	62500000	62750000	0,000358677	1	-0,030604442
chr4	62550000	62800000	0,00088966	1	-0,030302795
chr4	62600000	62850000	0,012031919	1	-0,029077734
chr4	62850000	63100000	0,040596742	1	-0,024556923
chr4	62900000	63150000	0,005984994	1	-0,024725655
chr4	62950000	63200000	0,000265426	1	-0,025133997

chr4	63000000	63250000	0,000455891	1	-0,025536193
chr4	63050000	63300000	0,001234566	1	-0,025767803
chr4	63100000	63350000	0,000501402	1	-0,025554938
chr4	63150000	63400000	0,000315125	1	-0,025087856
chr4	63200000	63450000	0,007750855	1	-0,024508677
chr4	63450000	63700000	0,006971889	1	-0,022190292
chr4	63500000	63750000	0,047707287	1	-0,021393623
chr4	63800000	64050000	0,021622565	1	-0,027784899
chr4	63850000	64100000	0,000805163	1	-0,029454885
chr4	63900000	64150000	0,000654767	1	-0,030509905
chr4	63950000	64200000	0,003336161	1	-0,030887132
chr4	64000000	64250000	0,004855679	1	-0,030695457
chr4	64050000	64300000	0,003347426	1	-0,030160767
chr4	64100000	64350000	0,001778325	1	-0,029527685

chr4	64150000	64400000	0,001385094	1	-0,028971042
chr4	64200000	64450000	0,002123019	1	-0,028550679
chr4	64250000	64500000	0,003950433	1	-0,028214887
chr4	64300000	64550000	0,005458406	1	-0,027862477
chr4	64350000	64600000	0,004301413	1	-0,027416847
chr4	64400000	64650000	0,001576227	1	-0,026874195
chr4	64450000	64700000	0,000200519	1	-0,026312911
chr4	64500000	64750000	5,15E-06	1	-0,025847425
chr4	64550000	64800000	2,44E-07	1	-0,02557204
chr4	64600000	64850000	6,51E-05	1	-0,025502683
chr4	64650000	64900000	0,001926581	1	-0,025561767
chr4	64700000	64950000	0,014485577	1	-0,025598196
chr4	64750000	65000000	0,041525501	1	-0,025443638
chr4	64800000	65050000	0,049133057	1	-0,02498179
chr4	64850000	65100000	0,01941816	1	-0,02421447
chr4	64900000	65150000	0,002541187	1	-

					0,023279264
chr4	64950000	65200000	0,019064963	1	-0,022425629
chr4	65200000	65450000	0,035613567	1	-0,026052673
chr4	65250000	65500000	0,010177304	1	-0,027788402
chr4	68350000	68600000	0,00747785	1	-0,019909725
chr4	68400000	68650000	0,014877567	1	-0,018739114
chr4	69650000	69900000	0,00903532	1	-0,021426923
chr4	69700000	69950000	0,027059231	1	-0,019936088
chr4	70250000	70500000	0,00121281	1	-0,004981873
chr4	70300000	70550000	0,000355926	1	-0,004981873
chr4	70350000	70600000	0,001936244	1	-0,004981873
chr4	70400000	70650000	0,002166494	1	-0,004981873
chr4	70450000	70700000	0,043218202	1	-0,004966966
chr4	71000000	71250000	0,004557567	1	-0,018899822
chr4	71050000	71300000	0,015420397	1	-

					0,019929726
chr4	72050000	72300000	0,020667522	1	- 0,022691044
chr4	72400000	72650000	0,010870571	1	- 0,016264539
chr4	72450000	72700000	0,021082003	1	- 0,016987722
chr4	72550000	72800000	0,042116746	1	- 0,017640547
chr4	72600000	72850000	0,006768976	1	-0,01730866
chr4	72650000	72900000	0,003500616	1	- 0,016479888
chr4	73650000	73900000	0,010279217	1	- 0,045288894
chr4	73700000	73950000	0,024907923	1	- 0,048361197
chr4	74000000	74250000	0,03660252	1	- 0,038797913
chr4	74050000	74300000	0,007762884	1	- 0,036898689
chr4	74100000	74350000	0,001688362	1	- 0,035631423
chr4	74150000	74400000	0,000447823	1	- 0,034848076
chr4	74200000	74450000	9,60E-05	1	- 0,034345994
chr4	74250000	74500000	8,59E-06	1	-0,03397183
chr4	74300000	74550000	2,15E-05	1	-

					0,033671652
chr4	74350000	74600000	0,000204776	1	-0,033486162
chr4	74400000	74650000	0,000419671	1	-0,033487782
chr4	74450000	74700000	0,000207368	1	-0,033711562
chr4	74500000	74750000	2,93E-05	1	-0,03410279
chr4	74550000	74800000	0,000156907	1	-0,034518712
chr4	74600000	74850000	0,001369427	1	-0,034775794
chr4	74650000	74900000	0,002950933	1	-0,034731328
chr4	74700000	74950000	0,0018387	1	-0,034349924
chr4	74750000	75000000	0,000269376	1	-0,033728178
chr4	74800000	75050000	0,000186524	1	-0,033073873
chr4	74850000	75100000	0,001589852	1	-0,032626226
chr4	74900000	75150000	0,00251926	1	-0,032568142
chr4	74950000	75200000	0,000712368	1	-0,033846603
chr4	75000000	75250000	0,004861565	1	-0,031997165

chr4	75900000	76150000	0,01978247	1	-0,036201757
chr4	75950000	76200000	0,019345953	1	-0,036201757
chr4	81200000	81450000	0,02085779	1	-0,025175931
chr4	81250000	81500000	0,004282697	1	-0,023973708
chr4	82750000	83000000	0,016941843	1	-0,045510656
chr4	85050000	85300000	0,005876683	1	-0,033957
chr4	85100000	85350000	0,019417781	1	-0,033957
chr4	85500000	85750000	0,010071216	1	-0,020026759
chr4	85550000	85800000	0,018503117	1	-0,021187158
chr4	86050000	86300000	0,013198468	1	-0,038789557
chr4	86100000	86350000	0,000483684	1	-0,038094077
chr4	86150000	86400000	0,001032985	1	-0,038094077
chr4	86200000	86450000	0,007657858	1	-0,038094077
chr4	86250000	86500000	0,019737101	1	-0,038094077
chr4	86300000	86550000	0,028742183	1	-0,038094077

chr4	86350000	86600000	0,031270062	1	-0,037111946
chr4	86400000	86650000	0,033822988	1	-0,036010306
chr4	87550000	87800000	0,004890553	1	-0,01619497
chr4	87600000	87850000	0,006888885	1	-0,017069207
chr4	88600000	88850000	0,036933032	1	-0,018346721
chr4	88650000	88900000	0,002476738	1	-0,018346721
chr4	88700000	88950000	0,001873711	1	-0,019093261
chr4	88750000	89000000	0,023475959	1	-0,019498075
chr4	90450000	90700000	0,013072699	1	-0,044830931
chr4	90500000	90750000	0,00100732	1	-0,047454359
chr4	90550000	90800000	0,000116636	1	-0,047852535
chr4	90600000	90850000	0,002013484	1	-0,046440016
chr4	90650000	90900000	0,013199042	1	-0,04480359
chr4	90700000	90950000	0,032257418	1	-0,043343068
chr4	90750000	91000000	0,033448974	1	-0,042561416

chr4	90800000	91050000	0,011254731	1	- 0,042909704
chr4	90850000	91100000	0,001966426	1	- 0,044638813
chr4	90900000	91150000	0,030102688	1	- 0,047668889
chr4	91200000	91450000	0,010028638	1	- 0,058142479
chr4	91250000	91500000	0,030842666	1	- 0,053860467
chr4	92050000	92300000	0,025485929	1	- 0,027076006
chr4	92100000	92350000	0,009499128	1	- 0,025646005
chr4	92400000	92650000	0,03265664	1	-0,02269964
chr4	92450000	92700000	0,014799082	1	- 0,021689878
chr4	92500000	92750000	0,012360158	1	-0,02102045
chr4	92550000	92800000	0,011759301	1	- 0,020545413
chr4	92600000	92850000	0,00666737	1	- 0,020101408
chr4	92650000	92900000	0,001309267	1	- 0,019610446
chr4	92700000	92950000	8,92E-05	1	- 0,019117931
chr4	92750000	93000000	0,000102881	1	-0,01875258
chr4	92800000	93050000	8,90E-05	1	-

					0,018631831
chr4	92850000	93100000	0,000409495	1	-0,018764904
chr4	92900000	93150000	0,009221958	1	-0,019011106
chr4	92950000	93200000	0,045115211	1	-0,019140327
chr4	93050000	93300000	0,019725315	1	-0,018379476
chr4	93100000	93350000	0,006590725	1	-0,017584928
chr4	93400000	93650000	0,008570053	1	-0,021757977
chr4	93450000	93700000	0,023423647	1	-0,022010671
chr4	96150000	96400000	0,008786946	1	-0,024483484
chr4	96200000	96450000	0,026743589	1	-0,022829219
chr4	96600000	96850000	0,026755523	1	-0,013000846
chr4	96650000	96900000	0,009112988	1	-0,013000846
chr4	97000000	97250000	0,008771617	1	-0,017585809
chr4	97050000	97300000	0,003256194	1	-0,016745458
chr4	97400000	97650000	0,022887918	1	-

					0,012578386
chr4	97850000	98100000	0,010616169	1	-0,020751498
chr4	97900000	98150000	0,000829639	1	-0,02014122
chr4	97950000	98200000	0,009343768	1	-0,019240942
chr4	98250000	98500000	0,004293837	1	-0,017201963
chr4	98300000	98550000	0,006996558	1	-0,016433972
chr4	98550000	98800000	0,014883072	1	-0,01834168
chr4	98600000	98850000	0,007307271	1	-0,019305079
chr4	101400000	101650000	0,036541835	1	-0,01541019
chr4	101450000	101700000	0,003944737	1	-0,01541019
chr4	101950000	102200000	0,009987687	1	-0,006251251
chr4	102000000	102250000	0,024965696	1	-0,005835399
chr4	104350000	104600000	0,014972047	1	-0,043639031
chr4	105600000	105850000	0,010958079	1	-0,035677386
chr4	105650000	105900000	0,002688375	1	-0,037694302
chr4	105700000	105950000	0,041070971	1	-0,03887425
chr4	106000000	106250000	0,048370278	1	-0,029808246

chr4	106050000	106300000	0,002797757	1	-0,028005421
chr4	106100000	106350000	2,68E-05	1	-0,026853818
chr4	106150000	106400000	8,84E-06	1	-0,026361617
chr4	106200000	106450000	0,001172087	1	-0,026369941
chr4	106250000	106500000	0,034782845	1	-0,026589742
chr4	107150000	107400000	0,027916881	1	-0,034086137
chr4	107200000	107450000	0,001997809	1	-0,033797643
chr4	107250000	107500000	0,000226786	1	-0,033005012
chr4	107300000	107550000	0,005235411	1	-0,031676541
chr4	107350000	107600000	0,048841952	1	-0,029871388
chr4	107700000	107950000	0,004906795	1	-0,02308545
chr4	107750000	108000000	0,006305727	1	-0,02308545
chr4	107800000	108050000	0,035688592	1	-0,02308545
chr4	107850000	108100000	0,049050151	1	-0,02308545
chr4	107900000	108150000	0,019696908	1	-0,02308545
chr4	107950000	108200000	0,002366953	1	-0,024038409
chr4	108000000	108250000	0,014264396	1	-

					0,024949765
chr4	108400000	108650000	0,020722598	1	-0,015289851
chr4	108450000	108700000	0,00022302	1	-0,014297159
chr4	108500000	108750000	2,98E-05	1	-0,013866595
chr4	108550000	108800000	3,26E-05	1	-0,013833479
chr4	108600000	108850000	0,000478956	1	-0,013974218
chr4	108650000	108900000	0,004414699	1	-0,014089685
chr4	108700000	108950000	0,013092177	1	-0,012799856
chr4	108750000	109000000	0,018989757	1	-0,012483964
chr4	108800000	109050000	0,017070577	1	-0,012223629
chr4	108850000	109100000	0,010152002	1	-0,012050624
chr4	108900000	109150000	0,003148973	1	-0,012037525
chr4	108950000	109200000	0,000355608	1	-0,012288523
chr4	109000000	109250000	0,004492156	1	-0,012903163
chr4	109800000	110050000	0,030700052	1	-0,03923014

chr4	109850000	110100000	0,003750766	1	-0,038835337
chr4	109900000	110150000	0,000538038	1	-0,037718122
chr4	109950000	110200000	0,009851218	1	-0,035960033
chr4	110500000	110750000	0,004923944	1	-0,01964808
chr4	110550000	110800000	0,006795126	1	-0,020448987
chr4	110600000	110850000	0,032117785	1	-0,021117013
chr4	110650000	110900000	0,032237379	1	-0,021342778
chr4	110700000	110950000	0,007183736	1	-0,020918843
chr4	110750000	111000000	0,005128095	1	-0,019790842
chr4	112300000	112550000	0,015931612	1	-0,021041942
chr4	112650000	112900000	0,015385287	1	-0,015766338
chr4	112700000	112950000	0,000365578	1	-0,014779683
chr4	112750000	113000000	0,000984942	1	-0,014357978
chr4	112800000	113050000	0,001423498	1	-0,014364019
chr4	112850000	113100000	0,001161474	1	-

					0,014583818
chr4	112900000	113150000	0,002850891	1	-0,014827316
chr4	112950000	113200000	0,016987338	1	-0,015005724
chr4	113200000	113450000	0,048294003	1	-0,019916664
chr4	113250000	113500000	0,004369002	1	-0,019577228
chr4	113300000	113550000	0,000809476	1	-0,01903701
chr4	113350000	113600000	0,005949272	1	-0,018516434
chr4	113400000	113650000	0,01280905	1	-0,01818631
chr4	113450000	113700000	0,007759672	1	-0,018107435
chr4	113500000	113750000	0,001239625	1	-0,018219949
chr4	113550000	113800000	4,51E-05	1	-0,018380489
chr4	113600000	113850000	2,06E-05	1	-0,018428008
chr4	113650000	113900000	1,78E-05	1	-0,018229397
chr4	113700000	113950000	0,000433108	1	-0,017701632
chr4	113750000	114000000	0,013243584	1	-0,01680637
chr4	115500000	115750000	0,008259579	1	-0,027802551

chr4	115550000	115800000	0,000991709	1	- 0,027943961
chr4	115600000	115850000	0,000139851	1	- 0,028081302
chr4	115650000	115900000	5,09E-05	1	- 0,028271257
chr4	115700000	115950000	9,25E-05	1	- 0,028580752
chr4	115750000	116000000	0,000425499	1	-0,02906905
chr4	115800000	116050000	0,002011305	1	- 0,029754098
chr4	115850000	116100000	0,006401154	1	- 0,030555399
chr4	115900000	116150000	0,011289333	1	- 0,031268213
chr4	115950000	116200000	0,008874655	1	- 0,031582729
chr4	116000000	116250000	0,002037113	1	- 0,031178036
chr4	116050000	116300000	0,001646632	1	- 0,029853522
chr4	116100000	116350000	0,049907347	1	- 0,027645634
chr4	116450000	116700000	0,022891929	1	- 0,019185796
chr4	116700000	116950000	0,021880478	1	- 0,022540068
chr4	116750000	117000000	0,001918983	1	-

					0,021730799
chr4	116800000	117050000	0,022918746	1	-0,020755486
chr4	117200000	117450000	0,026706519	1	-0,013514597
chr4	117250000	117500000	0,0068152	1	-0,012530374
chr4	117300000	117550000	0,044148897	1	-0,012665067
chr4	117400000	117650000	0,04047352	1	-0,01417319
chr4	117450000	117700000	0,006492259	1	-0,013981775
chr4	117500000	117750000	0,001887814	1	-0,013973857
chr4	117550000	117800000	0,039345954	1	-0,013973857
chr4	118400000	118650000	0,049179432	1	-0,024370905
chr4	118450000	118700000	0,029279761	1	-0,025533323
chr4	118500000	118750000	0,025059364	1	-0,026432057
chr4	118550000	118800000	0,023266526	1	-0,027204064
chr4	118600000	118850000	0,016870591	1	-0,027966692
chr4	118650000	118900000	0,006653522	1	-0,028777935

chr4	118700000	118950000	0,000846963	1	-0,029614265
chr4	118750000	119000000	0,001561548	1	-0,030384031
chr4	118800000	119050000	0,043910385	1	-0,030932368
chr4	119250000	119500000	0,018342297	1	-0,014694546
chr4	119450000	119700000	0,015031592	1	-0,01537139
chr4	119500000	119750000	0,004695579	1	-0,01621692
chr4	119850000	120100000	0,013439403	1	-0,011199248
chr4	121500000	121750000	0,046438251	1	-0,035222591
chr4	121750000	122000000	0,034487652	1	-0,041993125
chr4	121800000	122050000	0,008758112	1	-0,043739828
chr4	121850000	122100000	0,000710702	1	-0,045368032
chr4	121900000	122150000	0,00107109	1	-0,046656575
chr4	121950000	122200000	0,02121731	1	-0,047401772
chr4	125750000	126000000	0,008987208	1	-0,019783384
chr4	125800000	126050000	0,012015256	1	-0,018756267

chr4	127100000	127350000	0,012832354	1	-0,024591334
chr4	127150000	127400000	0,000928222	1	-0,023867156
chr4	127200000	127450000	0,008463423	1	-0,02337844
chr4	127250000	127500000	0,030955599	1	-0,023344321
chr4	127300000	127550000	0,037790096	1	-0,023344321
chr4	127350000	127600000	0,016018705	1	-0,023344321
chr4	127400000	127650000	0,001764946	1	-0,022970385
chr4	127450000	127700000	0,00376407	1	-0,022235913
chr4	127950000	128200000	0,004609402	1	-0,035361249
chr4	128000000	128250000	0,014015031	1	-0,037078428
chr4	129900000	130150000	0,011710092	1	-0,021959073
chr4	129950000	130200000	0,000133906	1	-0,021786411
chr4	130000000	130250000	8,26E-05	1	-0,022026012
chr4	130050000	130300000	0,000671207	1	-0,022799128
chr4	130100000	130350000	0,019472616	1	-

					0,024071207
chr4	130550000	130800000	0,027297352	1	- 0,037184548
chr4	130600000	130850000	0,002251262	1	- 0,036226202
chr4	130650000	130900000	0,007288374	1	- 0,034304635
chr4	131100000	131350000	0,031334632	1	- 0,019399386
chr4	131350000	131600000	0,042887497	1	- 0,023427329
chr4	131400000	131650000	0,009141444	1	- 0,022092018
chr4	131700000	131950000	0,048312811	1	- 0,017248477
chr4	131750000	132000000	0,001457738	1	- 0,016975989
chr4	131800000	132050000	0,000214235	1	- 0,016737721
chr4	131850000	132100000	0,001159527	1	- 0,016525072
chr4	131900000	132150000	0,00282864	1	- 0,016632937
chr4	131950000	132200000	0,008656835	1	- 0,016862099
chr4	132000000	132250000	0,034212306	1	- 0,017102859
chr4	132400000	132650000	0,013937714	1	-

					0,024371939
chr4	132450000	132700000	0,001029775	1	- 0,025443502
chr4	132500000	132750000	0,002713486	1	- 0,026251418
chr4	132550000	132800000	0,047003471	1	- 0,026707408
chr4	133000000	133250000	0,042793446	1	- 0,015794596
chr4	133050000	133300000	0,009934856	1	- 0,015054974
chr4	133100000	133350000	0,001315961	1	- 0,014514601
chr4	133150000	133400000	8,18E-05	1	- 0,014136344
chr4	133200000	133450000	1,38E-05	1	- 0,013912781
chr4	133250000	133500000	9,23E-05	1	- 0,013826092
chr4	133300000	133550000	0,000324658	1	- 0,013341511
chr4	133350000	133600000	0,000190874	1	- 0,013449855
chr4	133400000	133650000	0,000697352	1	- 0,013960883
chr4	133450000	133700000	0,032764225	1	- 0,014547975
chr4	133900000	134150000	0,008604107	1	-

					0,022748016
chr4	133950000	134200000	0,000403312	1	-0,023762039
chr4	134000000	134250000	6,27E-06	1	-0,024393213
chr4	134050000	134300000	9,94E-06	1	-0,024669216
chr4	134100000	134350000	1,39E-05	1	-0,024678539
chr4	134150000	134400000	1,74E-05	1	-0,024553212
chr4	134200000	134450000	0,000226498	1	-0,024417857
chr4	134250000	134500000	0,004419467	1	-0,024338396
chr4	134300000	134550000	0,042350668	1	-0,024274146
chr4	134600000	134850000	0,008869228	1	-0,017806321
chr4	134650000	134900000	0,020037371	1	-0,01834704
chr4	134850000	135100000	0,035563487	1	-0,018733473
chr4	134900000	135150000	0,021152952	1	-0,019604871
chr4	134950000	135200000	0,034399892	1	-0,020593059
chr4	135300000	135550000	0,013682022	1	-0,030130656

chr4	135350000	135600000	0,000797937	1	-0,029378761
chr4	135400000	135650000	0,003932681	1	-0,028334051
chr4	135450000	135700000	0,025610004	1	-0,027228032
chr4	135600000	135850000	0,03907629	1	-0,025017849
chr4	135650000	135900000	0,015744104	1	-0,024674339
chr4	135700000	135950000	0,004995653	1	-0,024366077
chr4	135750000	136000000	0,00221562	1	-0,023974809
chr4	135800000	136050000	0,002882584	1	-0,023410532
chr4	135850000	136100000	0,008677246	1	-0,022631079
chr4	135900000	136150000	0,029467637	1	-0,021655641
chr4	136150000	136400000	0,047290222	1	-0,018166124
chr4	136200000	136450000	0,005521027	1	-0,018322352
chr4	136250000	136500000	0,000140462	1	-0,018499282
chr4	136300000	136550000	2,23E-05	1	-0,018457111

chr4	136350000	136600000	8,68E-05	1	-0,018236327
chr4	136400000	136650000	0,004491498	1	-0,017998736
chr4	136750000	137000000	0,006222041	1	-0,014058646
chr4	136800000	137050000	0,02581489	1	-0,013357386
chr4	137500000	137750000	0,00704855	1	-0,035893684
chr4	137550000	137800000	0,0023048	1	-0,037536981
chr4	137600000	137850000	0,046317619	1	-0,038642815
chr4	139350000	139600000	0,014652504	1	-0,018131432
chr4	139400000	139650000	0,004953037	1	-0,018131432
chr4	140950000	141200000	0,033193241	1	-0,019914737
chr4	141000000	141250000	0,011119415	1	-0,019914737
chr4	141050000	141300000	0,001315031	1	-0,020603628
chr4	141100000	141350000	0,007242212	1	-0,02123321
chr4	142150000	142400000	0,004689168	1	-0,018359479
chr4	142200000	142450000	0,00014918	1	-

					0,018335003
chr4	142250000	142500000	9,43E-07	1	-0,018386456
chr4	142300000	142550000	3,83E-06	1	-0,018556424
chr4	142350000	142600000	9,10E-05	1	-0,018807872
chr4	142400000	142650000	0,000434892	1	-0,019039166
chr4	142450000	142700000	0,000519454	1	-0,01912254
chr4	142500000	142750000	0,000133214	1	-0,018959905
chr4	142550000	142800000	0,000156212	1	-0,01853289
chr4	142600000	142850000	0,003240852	1	-0,017929628
chr4	142650000	142900000	0,017103426	1	-0,017325063
chr4	142700000	142950000	0,029080714	1	-0,016927451
chr4	142750000	143000000	0,016057738	1	-0,016905568
chr4	142800000	143050000	0,002226811	1	-0,017327651
chr4	142850000	143100000	0,004291681	1	-0,018122684
chr4	142900000	143150000	0,047108522	1	-0,019098296
chr4	143050000	143300000	0,041751747	1	-

					0,019098296
chr4	143100000	143350000	0,004648827	1	-0,019098296
chr4	143150000	143400000	0,032370619	1	-0,019982249
chr4	144250000	144500000	0,002866121	1	-0,017408717
chr4	144300000	144550000	1,85E-05	1	-0,017562251
chr4	144350000	144600000	0,000199756	1	-0,017978963
chr4	144400000	144650000	0,003339607	1	-0,018536538
chr4	144450000	144700000	0,011474352	1	-0,018985686
chr4	144500000	144750000	0,009757287	1	-0,019061113
chr4	144550000	144800000	0,001928737	1	-0,018596346
chr4	144600000	144850000	0,006502774	1	-0,017588326
chr4	144950000	145200000	0,025697356	1	-0,013735957
chr4	145650000	145900000	0,04144685	1	-0,03343969
chr4	145700000	145950000	0,001976816	1	-0,035766285
chr4	145750000	146000000	0,003932452	1	-0,037321606

chr4	145800000	146050000	0,026442048	1	-0,037931359
chr4	149200000	149450000	0,011878213	1	-0,024388744
chr4	149550000	149800000	0,005715852	1	-0,018268196
chr4	149600000	149850000	0,000364875	1	-0,018638695
chr4	149650000	149900000	0,002041191	1	-0,018988632
chr4	149700000	149950000	0,003977828	1	-0,018988632
chr4	149750000	150000000	0,003426906	1	-0,018988632
chr4	149800000	150050000	0,002895379	1	-0,018988632
chr4	149850000	150100000	0,005459701	1	-0,018988632
chr4	149900000	150150000	0,020907375	1	-0,019278053
chr4	150200000	150450000	0,011243801	1	-0,024135191
chr4	153650000	153900000	0,034459206	1	-0,014881372
chr4	154800000	155050000	0,016452383	1	-0,024039073
chr4	155200000	155450000	0,020278657	1	-0,016004534

chr4	155250000	155500000	0,003609388	1	- 0,014559772
chr4	155300000	155550000	0,006920303	1	- 0,014044549
chr4	155350000	155600000	0,028050659	1	- 0,013436231
chr4	155500000	155750000	0,022364158	1	- 0,012808531
chr4	155550000	155800000	0,004804884	1	- 0,013485911
chr4	156000000	156250000	0,003785662	1	-0,02217228
chr4	156050000	156300000	0,000103382	1	-0,02217228
chr4	156100000	156350000	0,00063336	1	-0,02217228
chr4	156150000	156400000	0,005899768	1	-0,02217228
chr4	156200000	156450000	0,034512398	1	-0,02217228
chr4	158450000	158700000	0,009626228	1	- 0,014809297
chr4	158500000	158750000	0,006348515	1	- 0,015676389
chr4	160700000	160950000	0,022796884	1	- 0,027967881
chr4	160750000	161000000	0,029711847	1	- 0,026358592
chr4	161550000	161800000	0,033266991	1	- 0,012530786
chr4	161600000	161850000	0,003373373	1	- 0,012530786
chr4	161650000	161900000	0,005922962	1	-

					0,012292157
chr4	163250000	163500000	0,031562513	1	-0,017281426
chr4	163300000	163550000	0,038071985	1	-0,016536998
chr4	163400000	163650000	0,027035682	1	-0,016556409
chr4	163450000	163700000	0,004780266	1	-0,016556409
chr4	163500000	163750000	0,047528558	1	-0,016556409
chr4	163850000	164100000	0,044903903	1	-0,022120802
chr4	163900000	164150000	0,009093163	1	-0,023183938
chr4	163950000	164200000	0,000650338	1	-0,024110825
chr4	164000000	164250000	0,001639127	1	-0,0248183
chr4	164050000	164300000	0,014536675	1	-0,025189085
chr4	164100000	164350000	0,03382664	1	-0,025127399
chr4	164150000	164400000	0,025953367	1	-0,0246282
chr4	164200000	164450000	0,005535037	1	-0,023822906
chr4	164250000	164500000	0,000479688	1	-0,022883088
chr4	164300000	164550000	0,001514493	1	-

					0,023335515
chr4	164350000	164600000	0,002560344	1	-0,023404712
chr4	164400000	164650000	0,000732473	1	-0,022881941
chr4	164450000	164700000	0,004924577	1	-0,02172493
chr4	165500000	165750000	0,020797233	1	-0,024211607
chr4	165850000	166100000	0,005451188	1	-0,019333833
chr4	165900000	166150000	0,037549782	1	-0,018372569
chr4	166200000	166450000	0,010900607	1	-0,022766866
chr4	166250000	166500000	0,000794614	1	-0,02372836
chr4	166300000	166550000	3,58E-05	1	-0,024419245
chr4	166350000	166600000	8,06E-05	1	-0,024788268
chr4	166400000	166650000	5,19E-05	1	-0,024838972
chr4	166450000	166700000	0,000810183	1	-0,024650924
chr4	166500000	166750000	0,03104606	1	-0,025736786
chr4	167050000	167300000	0,008991955	1	-0,05733547
chr4	167100000	167350000	0,001828344	1	-0,055121431

chr4	167150000	167400000	0,031723821	1	-0,05187865
chr4	168550000	168800000	0,008041274	1	-0,030217309
chr4	168600000	168850000	0,032019422	1	-0,031902364
chr4	170450000	170700000	0,03428759	1	-0,010035946
chr4	171250000	171500000	0,002290533	1	-0,026828822
chr4	171300000	171550000	0,015066367	1	-0,025528849
chr4	171700000	171950000	0,037956136	1	-0,017522207
chr4	171750000	172000000	0,005561301	1	-0,017505537
chr4	171800000	172050000	0,000277005	1	-0,017612652
chr4	171850000	172100000	1,96E-05	1	-0,017612652
chr4	171900000	172150000	0,000239802	1	-0,017612652
chr4	171950000	172200000	0,001262971	1	-0,017612652
chr4	172000000	172250000	0,003608984	1	-0,017612652
chr4	172050000	172300000	0,008164166	1	-0,017810557
chr4	172100000	172350000	0,016538039	1	-

					0,018081779
chr4	172150000	172400000	0,030126944	1	- 0,018425496
chr4	172200000	172450000	0,04754835	1	- 0,018850843
chr4	172400000	172650000	0,035363387	1	- 0,021531026
chr4	172450000	172700000	0,01381098	1	- 0,022357673
chr4	172500000	172750000	0,00229286	1	- 0,023150942
chr4	172550000	172800000	0,000272895	1	- 0,023842411
chr4	172600000	172850000	0,005005453	1	- 0,024349972
chr4	172950000	173200000	0,044803489	1	- 0,018514828
chr4	173000000	173250000	0,002874356	1	- 0,017333179
chr4	173050000	173300000	0,015573161	1	- 0,016583878
chr4	173200000	173450000	0,028263875	1	- 0,017184159
chr4	173250000	173500000	0,005889116	1	- 0,017997478
chr4	176200000	176450000	0,008809279	1	- 0,030943373
chr4	176250000	176500000	0,042315427	1	-

					0,032879821
chr4	178250000	178500000	0,004809117	1	-0,021647716
chr4	178300000	178550000	0,000353259	1	-0,021066181
chr4	178350000	178600000	0,013451838	1	-0,019761798
chr4	179850000	180100000	0,033819292	1	-0,02585281
chr4	180200000	180450000	0,013993525	1	-0,018293811
chr4	180250000	180500000	0,000600405	1	-0,018684291
chr4	180300000	180550000	0,002015059	1	-0,018948321
chr4	180350000	180600000	0,001867698	1	-0,018955121
chr4	180400000	180650000	0,000299153	1	-0,018662598
chr4	180450000	180700000	0,000786385	1	-0,018107614
chr4	180500000	180750000	0,011302328	1	-0,017379387
chr4	181150000	181400000	0,006740705	1	-0,010745206
chr4	181200000	181450000	0,047858871	1	-0,010204048
chr4	181500000	181750000	0,0353446	1	-0,013169442

chr4	181550000	181800000	0,002173792	1	-0,013963102
chr4	181600000	181850000	0,000336393	1	-0,014529293
chr4	181650000	181900000	0,002293896	1	-0,014810424
chr4	181700000	181950000	0,004431544	1	-0,014811091
chr4	181750000	182000000	0,002811854	1	-0,014599792
chr4	181800000	182050000	0,000918395	1	-0,014290103
chr4	181850000	182100000	0,000984825	1	-0,014003293
chr4	181900000	182150000	0,014650619	1	-0,013816823
chr4	185800000	186050000	0,007810755	1	-0,021522669
chr4	186850000	187100000	0,041069649	1	-0,029551324
chr4	186900000	187150000	0,010414662	1	-0,029780594
chr4	186950000	187200000	0,001504682	1	-0,029749632
chr4	187000000	187250000	7,12E-05	1	-0,029576412
chr4	187050000	187300000	3,27E-06	1	-0,029455537

chr4	187100000	187350000	2,93E-06	1	- 0,029586653
chr4	187150000	187400000	3,03E-05	1	-0,03007902
chr4	187200000	187450000	0,001043608	1	- 0,030888583
chr4	187250000	187500000	0,007118464	1	- 0,031414372
chr4	187300000	187550000	0,014475596	1	- 0,031414372
chr4	187350000	187600000	0,009679749	1	- 0,031414372
chr4	187400000	187650000	0,001658119	1	- 0,031414372
chr4	187450000	187700000	0,002506252	1	- 0,031806235
chr4	188650000	188900000	0,009425686	1	- 0,033675124
chr4	188700000	188950000	0,00061423	1	- 0,035092065
chr4	188750000	189000000	0,003065005	1	-0,03610873
chr4	188800000	189050000	0,045971462	1	- 0,036624283
chr4	189200000	189450000	0,005955444	1	-0,02277144
chr4	189250000	189500000	0,046714868	1	- 0,024165814
chr4	189700000	189950000	0,021916041	1	- 0,037324837
chr4	189750000	190000000	0,001600318	1	-

					0,036877845
chr4	189800000	190050000	0,000315195	1	-0,036004191
chr4	189850000	190100000	0,004550343	1	-0,034820567
chr4	189900000	190150000	0,02402015	1	-0,033435549
chr4	190200000	190450000	0,04431181	1	-0,026880474
chr4	190250000	190500000	0,007359864	1	-0,027094559
chr4	190300000	190550000	0,000486238	1	-0,027717509
chr4	190350000	190600000	0,002090181	1	-0,027717509
chr4	190400000	190650000	0,007555272	1	-0,027717509
chr4	190450000	190700000	0,004706157	1	-0,027717509
chr4	190500000	190750000	0,001773075	1	-0,027717509
chr5	1200000	1450000	0,01275187	1	-0,026011188
chr5	1250000	1500000	0,002048377	1	-0,024769419
chr5	1300000	1550000	0,018854041	1	-0,023766557
chr5	1350000	1600000	0,04748087	1	-

					0,023073605
chr5	1400000	1650000	0,029871387	1	-0,023117156
chr5	1450000	1700000	0,005917063	1	-0,024274492
chr5	1900000	2150000	0,040217063	1	-0,05034833
chr5	1950000	2200000	0,009264134	1	-0,047537511
chr5	2600000	2850000	0,043707844	1	-0,02207052
chr5	2650000	2900000	0,019682434	1	-0,021939953
chr5	2700000	2950000	0,002842099	1	-0,021939953
chr5	2750000	3000000	0,000697266	1	-0,021939953
chr5	2800000	3050000	0,007795618	1	-0,021939953
chr5	2850000	3100000	0,026208096	1	-0,021939953
chr5	2900000	3150000	0,02776541	1	-0,022252224
chr5	2950000	3200000	0,010816349	1	-0,022920171
chr5	3000000	3250000	0,001285062	1	-0,023741711
chr5	3050000	3300000	3,29E-05	1	-0,02446968
chr5	3100000	3350000	3,03E-06	1	-0,024907455

chr5	3150000	3400000	0,000102654	1	-0,024992559
chr5	3200000	3450000	0,003974486	1	-0,024835599
chr5	3250000	3500000	0,027194869	1	-0,027494834
chr5	3350000	3600000	0,033193241	1	-0,028480445
chr5	3400000	3650000	0,005358583	1	-0,027721362
chr5	3450000	3700000	0,0095011	1	-0,026055953
chr5	3900000	4150000	0,023147099	1	-0,013814638
chr5	4550000	4800000	0,019025169	1	-0,038418684
chr5	4600000	4850000	0,002565078	1	-0,039998497
chr5	4650000	4900000	0,001321774	1	-0,041261988
chr5	5650000	5900000	0,038361221	1	-0,043168393
chr5	5700000	5950000	0,043598193	1	-0,040258824
chr5	7150000	7400000	0,032550053	1	-0,027845789
chr5	7200000	7450000	0,003211978	1	-0,026730314

chr5	7250000	7500000	0,030213861	1	- 0,025453179
chr5	7400000	7650000	0,040135651	1	- 0,025453179
chr5	7450000	7700000	0,004085745	1	- 0,025453179
chr5	7500000	7750000	0,010241976	1	-0,02435865
chr5	7850000	8100000	0,0290605	1	-0,03449738
chr5	7900000	8150000	0,006346629	1	- 0,034547867
chr5	7950000	8200000	0,000494769	1	- 0,034092602
chr5	8000000	8250000	0,000484832	1	- 0,033162777
chr5	8050000	8300000	0,007916998	1	- 0,031901715
chr5	8100000	8350000	0,038819715	1	- 0,030508469
chr5	8600000	8850000	0,013723167	1	-0,02236226
chr5	9000000	9250000	0,035839765	1	- 0,033864433
chr5	9050000	9300000	0,001486454	1	- 0,036128811
chr5	9100000	9350000	0,000269336	1	- 0,037559221
chr5	9150000	9400000	0,001491128	1	- 0,038158819
chr5	9200000	9450000	0,001648058	1	-

					0,038072774
chr5	9250000	9500000	0,000338837	1	-0,037535479
chr5	9300000	9550000	0,000967457	1	-0,036833227
chr5	9350000	9600000	0,017828516	1	-0,036273203
chr5	9550000	9800000	0,021155202	1	-0,039801739
chr5	9600000	9850000	0,003495277	1	-0,041837148
chr5	11050000	11300000	0,013310383	1	-0,037104146
chr5	11550000	11800000	0,047911357	1	-0,015872918
chr5	12050000	12300000	0,006822255	1	-0,027811495
chr5	12100000	12350000	0,00029888	1	-0,028565163
chr5	12150000	12400000	0,000625558	1	-0,027855751
chr5	12200000	12450000	0,006390239	1	-0,026975629
chr5	12250000	12500000	0,022524242	1	-0,026020975
chr5	12300000	12550000	0,042848634	1	-0,025556546
chr5	12400000	12650000	0,036808074	1	-

					0,025556546
chr5	12450000	12700000	0,011949646	1	- 0,025556546
chr5	12500000	12750000	0,001161082	1	- 0,025098865
chr5	12550000	12800000	0,002713607	1	- 0,024337878
chr5	12600000	12850000	0,030101142	1	- 0,023889701
chr5	12750000	13000000	0,027754915	1	- 0,025400703
chr5	12800000	13050000	0,00332488	1	-0,02662107
chr5	12850000	13100000	0,026537955	1	- 0,027740589
chr5	13250000	13500000	0,032012473	1	- 0,017155644
chr5	15400000	15650000	0,034629672	1	- 0,027525886
chr5	15450000	15700000	0,007074098	1	- 0,026226171
chr5	15700000	15950000	0,008102312	1	- 0,023618488
chr5	15750000	16000000	0,003994872	1	- 0,023618488
chr5	15800000	16050000	0,033402228	1	-0,02291486
chr5	15900000	16150000	0,028924359	1	- 0,023453169
chr5	15950000	16200000	0,004955511	1	-

					0,024478644
chr5	16350000	16600000	0,008266912	1	-0,015577704
chr5	16400000	16650000	0,001623741	1	-0,014511049
chr5	16450000	16700000	0,008722977	1	-0,014337069
chr5	17950000	18200000	0,002238514	1	-0,047021019
chr5	18000000	18250000	0,002509897	1	-0,045427602
chr5	18050000	18300000	0,022638063	1	-0,0435073
chr5	18600000	18850000	0,030466863	1	-0,025982501
chr5	18650000	18900000	0,00412896	1	-0,026075426
chr5	18700000	18950000	0,000162734	1	-0,026394431
chr5	18750000	19000000	0,000150496	1	-0,026791821
chr5	18800000	19050000	0,000675537	1	-0,027087925
chr5	18850000	19100000	0,00054092	1	-0,027102637
chr5	18900000	19150000	0,000100989	1	-0,026696909
chr5	18950000	19200000	0,001027893	1	-0,025810112

chr5	19000000	19250000	0,02018953	1	-0,024481831
chr5	19400000	19650000	0,00789581	1	-0,016626651
chr5	19450000	19700000	0,002458054	1	-0,016626651
chr5	19500000	19750000	0,018125998	1	-0,016626651
chr5	19550000	19800000	0,035614664	1	-0,016652823
chr5	19600000	19850000	0,02577871	1	-0,017053594
chr5	19650000	19900000	0,00790618	1	-0,017660359
chr5	19700000	19950000	0,000843572	1	-0,018285381
chr5	19750000	20000000	3,46E-05	1	-0,018779661
chr5	19800000	20050000	0,000375853	1	-0,01906346
chr5	19850000	20100000	0,005700087	1	-0,01845576
chr5	19900000	20150000	0,045055373	1	-0,017692354
chr5	21450000	21700000	0,021081312	1	-0,038715851
chr5	21500000	21750000	0,00116082	1	-0,042403342
chr5	21550000	21800000	0,000122836	1	-0,041731074

chr5	21600000	21850000	0,000758719	1	-0,041123717
chr5	21650000	21900000	0,001434161	1	-0,04069605
chr5	21700000	21950000	0,001021416	1	-0,040374174
chr5	21750000	22000000	0,000581113	1	-0,039913274
chr5	21800000	22050000	0,001145355	1	-0,038992426
chr5	21850000	22100000	0,00859844	1	-0,037359554
chr5	22300000	22550000	0,048636572	1	-0,020642796
chr5	22350000	22600000	0,002656145	1	-0,021376802
chr5	22400000	22650000	0,014976216	1	-0,022166051
chr5	22450000	22700000	0,038366101	1	-0,022829798
chr5	22500000	22750000	0,033422491	1	-0,023272427
chr5	22550000	22800000	0,013152001	1	-0,0234716
chr5	22600000	22850000	0,002175714	1	-0,023445645
chr5	22650000	22900000	0,00010445	1	-0,023204119
chr5	22700000	22950000	0,000195911	1	-0,022722323

chr5	22750000	23000000	0,003990509	1	-0,022039613
chr5	22800000	23050000	0,031090395	1	-0,021188654
chr5	23250000	23500000	0,029942276	1	-0,012841642
chr5	23600000	23850000	0,018402968	1	-0,017286994
chr5	23650000	23900000	0,00081713	1	-0,01822843
chr5	23700000	23950000	0,000285636	1	-0,018841139
chr5	23750000	24000000	0,001357625	1	-0,019107897
chr5	23800000	24050000	0,000979267	1	-0,019061412
chr5	23850000	24100000	0,000476496	1	-0,018786645
chr5	23900000	24150000	0,014643434	1	-0,018431366
chr5	24200000	24450000	0,049076395	1	-0,02307864
chr5	24250000	24500000	0,004744783	1	-0,024523343
chr5	24300000	24550000	0,000110047	1	-0,025573397
chr5	24350000	24600000	1,25E-05	1	-0,026148577
chr5	24400000	24650000	9,21E-05	1	-0,026318495

chr5	24450000	24700000	0,000377649	1	-0,025234378
chr5	24500000	24750000	0,001989198	1	-0,024570709
chr5	24550000	24800000	0,011856356	1	-0,023584137
chr5	25050000	25300000	0,0098416	1	-0,014414516
chr5	25100000	25350000	0,020651012	1	-0,013566806
chr5	25350000	25600000	0,013882913	1	-0,015569546
chr5	25400000	25650000	0,001125985	1	-0,016367216
chr5	25450000	25700000	0,010133488	1	-0,015675604
chr5	25700000	25950000	0,026162087	1	-0,013680431
chr5	25750000	26000000	0,002198138	1	-0,013680431
chr5	25800000	26050000	0,021468223	1	-0,013506102
chr5	26300000	26550000	0,003630443	1	-0,025053858
chr5	26350000	26600000	0,000440821	1	-0,026241183
chr5	26400000	26650000	0,003086896	1	-0,026803511

chr5	26450000	26700000	0,006937865	1	-0,026790334
chr5	26500000	26750000	0,006188757	1	-0,026379344
chr5	26550000	26800000	0,003092369	1	-0,025792808
chr5	26600000	26850000	0,001118201	1	-0,025218184
chr5	26650000	26900000	0,000400628	1	-0,02475224
chr5	26700000	26950000	0,000208859	1	-0,024413297
chr5	26750000	27000000	0,000203109	1	-0,024165988
chr5	26800000	27050000	0,000327467	1	-0,023970879
chr5	26850000	27100000	0,000551599	1	-0,023791871
chr5	26900000	27150000	0,000565906	1	-0,023599758
chr5	26950000	27200000	0,000184272	1	-0,023365879
chr5	27000000	27250000	0,000111557	1	-0,02308598
chr5	27050000	27300000	0,004347054	1	-0,022807041
chr5	27100000	27350000	0,043550947	1	-0,022642107
chr5	27350000	27600000	0,009066569	1	-0,028787685

chr5	27400000	27650000	0,000730265	1	-0,028346055
chr5	27450000	27700000	0,005634926	1	-0,027329609
chr5	27500000	27750000	0,028200239	1	-0,026328745
chr5	27550000	27800000	0,049699923	1	-0,0255156
chr5	27600000	27850000	0,042649251	1	-0,024962405
chr5	27650000	27900000	0,019934885	1	-0,024640123
chr5	27700000	27950000	0,005464059	1	-0,024438942
chr5	27750000	28000000	0,00121541	1	-0,024201086
chr5	27800000	28050000	0,000815408	1	-0,023758391
chr5	27850000	28100000	0,003808884	1	-0,023758391
chr5	27900000	28150000	0,026807859	1	-0,023758391
chr5	28200000	28450000	0,028327323	1	-0,018774298
chr5	28600000	28850000	0,016683104	1	-0,030354418
chr5	28650000	28900000	0,001524524	1	-0,030354418
chr5	28700000	28950000	0,000493027	1	-

					0,030354418
chr5	28750000	29000000	0,001448726	1	-0,030354418
chr5	28800000	29050000	0,005104113	1	-0,030354418
chr5	28850000	29100000	0,010602741	1	-0,030621753
chr5	28900000	29150000	0,012391591	1	-0,031106356
chr5	28950000	29200000	0,008488502	1	-0,031814387
chr5	29000000	29250000	0,003484247	1	-0,03264493
chr5	29050000	29300000	0,000823209	1	-0,033458483
chr5	29100000	29350000	9,54E-05	1	-0,034136948
chr5	29150000	29400000	3,55E-06	1	-0,034939174
chr5	29200000	29450000	2,21E-07	1	-0,034828994
chr5	29250000	29500000	1,36E-06	1	-0,034724129
chr5	29300000	29550000	3,31E-06	1	-0,034614811
chr5	29350000	29600000	8,49E-06	1	-0,034404788
chr5	29400000	29650000	9,99E-05	1	-0,034404788

chr5	29450000	29700000	0,00250142	1	-0,034404788
chr5	29500000	29750000	0,047485067	1	-0,034404788
chr5	30550000	30800000	0,012490186	1	-0,026957448
chr5	30600000	30850000	0,000181456	1	-0,026690961
chr5	30650000	30900000	0,000204173	1	-0,026856498
chr5	30700000	30950000	0,000243127	1	-0,026856498
chr5	30750000	31000000	0,006616428	1	-0,026856498
chr5	30950000	31200000	0,03555222	1	-0,028815293
chr5	31000000	31250000	0,006151763	1	-0,030321615
chr5	33100000	33350000	0,001238461	1	-0,020097101
chr5	38000000	38250000	0,029396206	1	-0,022476964
chr5	38050000	38300000	0,0031476	1	-0,023618212
chr5	38100000	38350000	0,026480725	1	-0,024604493
chr5	38550000	38800000	0,010269368	1	-0,011185341

chr5	38600000	38850000	0,015839437	1	-0,011598445
chr5	38650000	38900000	0,041804394	1	-0,011914099
chr5	38700000	38950000	0,033927319	1	-0,012157824
chr5	38750000	39000000	0,018024262	1	-0,012441457
chr5	38800000	39050000	0,017116538	1	-0,012891714
chr5	38850000	39100000	0,043766044	1	-0,013565498
chr5	39050000	39300000	0,033143579	1	-0,014885269
chr5	39100000	39350000	0,037703982	1	-0,013504478
chr5	41200000	41450000	0,005769137	1	-0,042038396
chr5	42250000	42500000	0,014762627	1	-0,044569687
chr5	42300000	42550000	0,003952073	1	-0,044569687
chr5	42350000	42600000	0,000451685	1	-0,045474919
chr5	42400000	42650000	0,001701318	1	-0,046480255
chr5	42450000	42700000	0,039829955	1	-0,047213367

chr5	44000000	44250000	0,036996211	1	-<br>0,038645454
chr5	44050000	44300000	0,001417109	1	-<br>0,037598998
chr5	44100000	44350000	0,006050994	1	-<br>0,036086669
chr5	44150000	44400000	0,045234137	1	-<br>0,034314399
chr5	44800000	45050000	0,032019419	1	-<br>0,020836668
chr5	44850000	45100000	0,00542016	1	-<br>0,021943848
chr5	45400000	45650000	0,023111911	1	-0,04436306
chr5	45450000	45700000	0,001266936	1	-<br>0,045226298
chr5	45500000	45750000	0,001193367	1	-<br>0,045208124
chr5	45550000	45800000	0,015757252	1	-<br>0,044755313
chr5	45950000	46200000	0,037601971	1	-<br>0,030172351
chr5	49450000	49700000	0,046622573	1	-<br>0,019097169
chr5	49500000	49750000	0,002435708	1	-0,02085207
chr5	49550000	49800000	0,015858942	1	-<br>0,021715367
chr5	49850000	50100000	0,04036241	1	-<br>0,016170935

chr5	49900000	50150000	0,018144691	1	-0,0159779
chr5	49950000	50200000	0,004840991	1	-0,015877282
chr5	50000000	50250000	0,001081696	1	-0,015690255
chr5	50050000	50300000	0,002238313	1	-0,015173618
chr5	50100000	50350000	0,037625306	1	-0,014112728
chr5	51550000	51800000	0,024503681	1	-0,036619602
chr5	52350000	52600000	0,031048702	1	-0,011092769
chr5	52400000	52650000	0,027738133	1	-0,010345327
chr5	52600000	52850000	0,031730019	1	-0,010042953
chr5	53100000	53350000	0,046753823	1	-0,022094564
chr5	53150000	53400000	0,006305534	1	-0,022094564
chr5	56850000	57100000	0,012661877	1	-0,013103091
chr5	56900000	57150000	0,034833485	1	-0,01410459
chr5	57100000	57350000	0,043906826	1	-0,0126689
chr5	57150000	57400000	0,034973981	1	-0,012132194
chr5	57200000	57450000	0,045950513	1	-0,01174106

chr5	57300000	57550000	0,019666212	1	-0,010154082
chr5	57350000	57600000	0,009945082	1	-0,010885842
chr5	57800000	58050000	0,038493744	1	-0,021376198
chr5	59400000	59650000	0,021140486	1	-0,037950561
chr5	59450000	59700000	0,002421486	1	-0,037508053
chr5	59500000	59750000	0,000673463	1	-0,037508053
chr5	59550000	59800000	0,01439186	1	-0,037508053
chr5	59850000	60100000	0,043204143	1	-0,030065135
chr5	59900000	60150000	0,003042152	1	-0,028195706
chr5	59950000	60200000	0,018262914	1	-0,026976033
chr5	60150000	60400000	0,024878679	1	-0,02937406
chr5	60200000	60450000	0,001952771	1	-0,030845463
chr5	60250000	60500000	0,001964369	1	-0,032041243
chr5	60300000	60550000	0,016968557	1	-0,03268851
chr5	60350000	60600000	0,036562843	1	-0,032643063

chr5	60400000	60650000	0,022154344	1	-0,031948684
chr5	60450000	60700000	0,003207451	1	-0,030842677
chr5	60500000	60750000	0,007282736	1	-0,03130838
chr5	60800000	61050000	0,028619507	1	-0,039754606
chr5	60850000	61100000	0,001814871	1	-0,038835849
chr5	60900000	61150000	0,003671704	1	-0,037211497
chr5	60950000	61200000	0,047091767	1	-0,035075328
chr5	64050000	64300000	0,023966146	1	-0,029920685
chr5	64100000	64350000	0,006000187	1	-0,032254144
chr5	67300000	67550000	0,042254981	1	-0,01961643
chr5	67350000	67600000	0,009376835	1	-0,020754188
chr5	67400000	67650000	0,007774546	1	-0,021552114
chr5	67450000	67700000	0,040440206	1	-0,022027293
chr5	67900000	68150000	0,013789423	1	-0,039943378
chr5	73750000	74000000	0,022944715	1	-0,012060134

chr5	73800000	74050000	0,010688058	1	-0,013023057
chr5	73950000	74200000	0,014492118	1	-0,012936169
chr5	74000000	74250000	0,012016803	1	-0,012313201
chr5	74450000	74700000	0,018254365	1	-0,020351936
chr5	74500000	74750000	0,00136644	1	-0,019960592
chr5	74550000	74800000	0,00218379	1	-0,019174652
chr5	74600000	74850000	0,035163726	1	-0,018095034
chr5	74850000	75100000	0,039622198	1	-0,017709299
chr5	75250000	75500000	0,018839866	1	-0,031751176
chr5	76800000	77050000	0,037613165	1	-0,025364016
chr5	76850000	77100000	0,002459229	1	-0,025663895
chr5	76900000	77150000	0,000982055	1	-0,026165954
chr5	76950000	77200000	0,002832071	1	-0,026811092
chr5	77000000	77250000	0,006885963	1	-0,026904602

chr5	77050000	77300000	0,007777171	1	-0,026904602
chr5	77100000	77350000	0,003033127	1	-0,026904602
chr5	77150000	77400000	0,000405373	1	-0,026904602
chr5	77200000	77450000	0,004188555	1	-0,027435712
chr5	77900000	78150000	0,048686232	1	-0,010841521
chr5	77950000	78200000	0,020536462	1	-0,010568572
chr5	78600000	78850000	0,005403552	1	-0,026773655
chr5	78650000	78900000	0,000335676	1	-0,026920903
chr5	78700000	78950000	7,21E-05	1	-0,027180136
chr5	78750000	79000000	0,0001675	1	-0,027510516
chr5	78800000	79050000	0,000400255	1	-0,027808537
chr5	78850000	79100000	0,000421609	1	-0,027969987
chr5	78900000	79150000	0,000146315	1	-0,027941576
chr5	78950000	79200000	1,34E-05	1	-0,027741582

chr5	79000000	79250000	2,33E-05	1	-0,027468199
chr5	79050000	79300000	0,000194426	1	-0,027468199
chr5	79100000	79350000	0,000470786	1	-0,027468199
chr5	79150000	79400000	0,000525858	1	-0,027468199
chr5	79200000	79450000	0,000446559	1	-0,027441288
chr5	79250000	79500000	0,000621709	1	-0,027124804
chr5	79300000	79550000	0,002426896	1	-0,026847264
chr5	79350000	79600000	0,015783731	1	-0,026608459
chr5	80200000	80450000	0,046155095	1	-0,019101245
chr5	80250000	80500000	0,004841769	1	-0,020210235
chr5	80300000	80550000	0,000230819	1	-0,021047736
chr5	80350000	80600000	0,001120476	1	-0,021560332
chr5	80400000	80650000	0,005854226	1	-0,020167011
chr5	80450000	80700000	0,009639587	1	-0,019793431

chr5	80500000	80750000	0,007022943	1	-0,019562701
chr5	80550000	80800000	0,002641623	1	-0,019422242
chr5	80600000	80850000	0,000655325	1	-0,019285855
chr5	80650000	80900000	0,00023745	1	-0,019096958
chr5	80700000	80950000	0,000270344	1	-0,018872373
chr5	80750000	81000000	0,000373134	1	-0,018872373
chr5	80800000	81050000	0,000190879	1	-0,018872373
chr5	80850000	81100000	7,32E-05	1	-0,018872373
chr5	80900000	81150000	0,002981203	1	-0,018872373
chr5	80950000	81200000	0,043188134	1	-0,02110389
chr5	81250000	81500000	0,010324657	1	-0,025121356
chr5	81650000	81900000	0,025291737	1	-0,015563476
chr5	81700000	81950000	0,049894749	1	-0,015794717
chr5	81750000	82000000	0,026842065	1	-0,014891126
chr5	85800000	86050000	0,034261994	1	-

					0,024689223
chr5	85850000	86100000	0,010962501	1	-0,02702323
chr5	86050000	86300000	0,036692609	1	- 0,025506766
chr5	86100000	86350000	0,003874643	1	- 0,024265364
chr5	86150000	86400000	0,000198076	1	- 0,023331006
chr5	86200000	86450000	0,001465203	1	- 0,023912371
chr5	86250000	86500000	0,007468469	1	-0,02457791
chr5	86300000	86550000	0,015724287	1	- 0,025280449
chr5	86350000	86600000	0,020592581	1	- 0,025963741
chr5	86400000	86650000	0,018782517	1	- 0,026540272
chr5	86450000	86700000	0,011137077	1	- 0,026898294
chr5	86500000	86750000	0,003272599	1	- 0,026919504
chr5	86550000	86800000	0,000310697	1	- 0,026507326
chr5	86600000	86850000	0,001027822	1	- 0,025628196
chr5	86650000	86900000	0,01984635	1	- 0,024325312
chr5	87050000	87300000	0,006042098	1	-

					0,016730069
chr5	87100000	87350000	0,005296411	1	- 0,016730069
chr5	87150000	87400000	0,04603412	1	- 0,016730069
chr5	87300000	87550000	0,046292903	1	- 0,018018322
chr5	87350000	87600000	0,005922115	1	- 0,018909181
chr5	87400000	87650000	0,007773354	1	- 0,019746033
chr5	89800000	90050000	0,026842065	1	-0,02311371
chr5	92750000	93000000	0,010927087	1	-0,02226532
chr5	92800000	93050000	0,009166658	1	- 0,020933521
chr5	94250000	94500000	0,013202955	1	- 0,031617061
chr5	94300000	94550000	0,029815478	1	- 0,029325048
chr5	96600000	96850000	0,029128189	1	- 0,025225208
chr5	96650000	96900000	0,016852178	1	- 0,023560833
chr5	97750000	98000000	0,011274485	1	- 0,034137953
chr5	97800000	98050000	0,000872289	1	- 0,035564232
chr5	97850000	98100000	0,000121548	1	-

					0,036596253
chr5	97900000	98150000	0,001329484	1	- 0,037180549
chr5	97950000	98200000	0,004557816	1	- 0,037288572
chr5	98000000	98250000	0,004263612	1	- 0,036944678
chr5	98050000	98300000	0,00092146	1	- 0,036260216
chr5	98100000	98350000	0,001200304	1	- 0,035441041
chr5	98150000	98400000	0,029603626	1	- 0,034766606
chr5	98450000	98700000	0,019044059	1	- 0,048225297
chr5	98500000	98750000	0,008599781	1	- 0,045397638
chr5	99050000	99300000	0,010602741	1	- 0,019434291
chr5	99100000	99350000	0,049784529	1	- 0,019480967
chr5	99550000	99800000	0,039987005	1	- 0,032779839
chr5	99600000	99850000	0,007942088	1	- 0,034529774
chr5	99650000	99900000	0,001056561	1	- 0,035823823
chr5	99700000	99950000	7,52E-05	1	-

					0,036702264
chr5	99750000	100000000	1,76E-06	1	-0,037239365
chr5	99800000	100050000	9,69E-09	1	-0,037517394
chr5	99850000	100100000	5,05E-09	1	-0,037608491
chr5	99900000	100150000	3,48E-09	1	-0,037590106
chr5	99950000	100200000	7,36E-09	1	-0,037540146
chr5	100000000	100250000	6,89E-06	1	-0,037598112
chr5	100050000	100300000	0,000417132	1	-0,037598112
chr5	100100000	100350000	0,006062629	1	-0,037598112
chr5	100150000	100400000	0,035499777	1	-0,037497374
chr5	100350000	100600000	0,021789068	1	-0,033196372
chr5	100400000	100650000	0,004631794	1	-0,031535068
chr5	100700000	100950000	0,009801871	1	-0,039107784
chr5	100750000	101000000	0,002814513	1	-0,037489247
chr5	100800000	101050000	0,036981342	1	-

					0,035411297
chr5	101200000	101450000	0,015518148	1	-0,027451373
chr5	101250000	101500000	0,001095761	1	-0,026138098
chr5	101300000	101550000	0,005383811	1	-0,025271283
chr5	101350000	101600000	0,017639813	1	-0,024952427
chr5	101400000	101650000	0,010009048	1	-0,025219232
chr5	101450000	101700000	0,004411905	1	-0,02595992
chr5	103050000	103300000	0,020888056	1	-0,044111276
chr5	103100000	103350000	0,000421047	1	-0,04351916
chr5	103150000	103400000	0,000325057	1	-0,042746506
chr5	103200000	103450000	0,002797864	1	-0,042746506
chr5	103250000	103500000	0,011444729	1	-0,042746506
chr5	103300000	103550000	0,043353157	1	-0,042746506
chr5	104150000	104400000	0,027704329	1	-0,040297723
chr5	104200000	104450000	0,001825132	1	-0,038866041
chr5	104250000	104500000	0,020998886	1	-

					0,036687925
chr5	104600000	104850000	0,029426838	1	-0,026040906
chr5	104650000	104900000	0,000743123	1	-0,026304495
chr5	104700000	104950000	2,09E-05	1	-0,026317852
chr5	104750000	105000000	3,52E-05	1	-0,025943614
chr5	104800000	105050000	0,001209855	1	-0,02570685
chr5	104850000	105100000	0,019206276	1	-0,025629018
chr5	105250000	105500000	0,015836802	1	-0,019226322
chr5	105300000	105550000	0,001826146	1	-0,018509696
chr5	105350000	105600000	0,000931285	1	-0,017954007
chr5	105400000	105650000	0,019327962	1	-0,017587295
chr5	105750000	106000000	0,035193701	1	-0,022795094
chr5	105800000	106050000	0,032438657	1	-0,023710551
chr5	106050000	106300000	0,040584072	1	-0,028023747
chr5	106100000	106350000	0,004875071	1	-0,029392197

chr5	106150000	106400000	0,018245899	1	-0,030682652
chr5	106650000	106900000	0,045602948	1	-0,009987881
chr5	107350000	107600000	0,032877425	1	-0,058162532
chr5	107850000	108100000	0,04117976	1	-0,019945846
chr5	108150000	108400000	0,003298434	1	-0,022802609
chr5	108200000	108450000	0,004948057	1	-0,02185691
chr5	108250000	108500000	0,039675744	1	-0,02185691
chr5	108400000	108650000	0,040844158	1	-0,02185691
chr5	108450000	108700000	0,00661322	1	-0,02086147
chr5	108500000	108750000	0,000270842	1	-0,020043993
chr5	108550000	108800000	0,000678289	1	-0,020225118
chr5	108600000	108850000	0,010756942	1	-0,021090862
chr5	109150000	109400000	0,006485871	1	-0,044631275
chr5	109650000	109900000	0,011346685	1	-0,023551045
chr5	110050000	110300000	0,010121591	1	-0,033413219
chr5	110100000	110350000	0,000921619	1	-0,034822572

chr5	110150000	110400000	3,22E-05	1	-0,035771911
chr5	110200000	110450000	4,09E-06	1	-0,036281084
chr5	110250000	110500000	9,77E-05	1	-0,036449188
chr5	110300000	110550000	0,001050903	1	-0,036448463
chr5	110350000	110600000	0,003084215	1	-0,036492756
chr5	110400000	110650000	0,002129942	1	-0,036777106
chr5	110450000	110700000	0,000828688	1	-0,037384178
chr5	110500000	110750000	0,04113476	1	-0,038194776
chr5	114000000	114250000	0,018053754	1	-0,027499752
chr5	115150000	115400000	0,014909833	1	-0,031836134
chr5	115200000	115450000	0,005383531	1	-0,032884684
chr5	115250000	115500000	0,027038817	1	-0,034710372
chr5	115600000	115850000	0,046597657	1	-0,049083657
chr5	115650000	115900000	0,003636672	1	-0,048322578

chr5	115700000	115950000	0,001006805	1	-0,046743952
chr5	115750000	116000000	0,016959418	1	-0,044493475
chr5	116300000	116550000	0,014447093	1	-0,022253681
chr5	116350000	116600000	0,000312493	1	-0,022253681
chr5	116400000	116650000	0,000198214	1	-0,022253681
chr5	116450000	116700000	0,000160335	1	-0,022253681
chr5	116500000	116750000	0,000282154	1	-0,022480412
chr5	116550000	116800000	0,007182606	1	-0,022801113
chr5	116600000	116850000	0,048463699	1	-0,02295657
chr5	116750000	117000000	0,039916849	1	-0,018911908
chr5	116800000	117050000	0,003695452	1	-0,019472978
chr5	116850000	117100000	0,006439893	1	-0,020364406
chr5	117100000	117350000	0,016382486	1	-0,021369721
chr5	117150000	117400000	0,00303332	1	-0,022283412
chr5	117200000	117450000	0,000816246	1	-

					0,022970026
chr5	117250000	117500000	0,002261214	1	-<br>0,023392285
chr5	117300000	117550000	0,02062514	1	-0,02361603
chr5	117800000	118050000	0,010754499	1	-<br>0,046038264
chr5	117850000	118100000	0,045532603	1	-<br>0,048896322
chr5	119000000	119250000	0,043946171	1	-<br>0,044561977
chr5	119050000	119300000	0,003533405	1	-0,04271154
chr5	119100000	119350000	0,036853288	1	-<br>0,039844525
chr5	119900000	120150000	0,021190315	1	-<br>0,012467645
chr5	119950000	120200000	0,00310412	1	-<br>0,012995592
chr5	120000000	120250000	0,031079689	1	-<br>0,013657651
chr5	120200000	120450000	0,015034888	1	-<br>0,014963471
chr5	120250000	120500000	0,000918873	1	-<br>0,014963471
chr5	120300000	120550000	0,00036554	1	-<br>0,014963471
chr5	120350000	120600000	0,000566319	1	-<br>0,014963471
chr5	120400000	120650000	0,002318227	1	-

					0,014963471
chr5	120900000	121150000	0,02463915	1	-0,038005722
chr5	121400000	121650000	0,004954074	1	-0,018151234
chr5	121450000	121700000	0,027791575	1	-0,017373377
chr5	121500000	121750000	0,048951552	1	-0,017400957
chr5	121550000	121800000	0,032144157	1	-0,017915975
chr5	121600000	121850000	0,010067887	1	-0,018624525
chr5	121650000	121900000	0,00137206	1	-0,019310534
chr5	121700000	121950000	5,44E-05	1	-0,019845347
chr5	121750000	122000000	5,26E-05	1	-0,020181145
chr5	121800000	122050000	0,000856934	1	-0,020321155
chr5	121850000	122100000	0,005705985	1	-0,020295989
chr5	121900000	122150000	0,025795518	1	-0,02013522
chr5	122400000	122650000	0,010603022	1	-0,011238907
chr5	122750000	123000000	0,043918092	1	-0,016185569

chr5	124650000	124900000	0,0366932	1	- 0,025191962
chr5	124700000	124950000	0,017507801	1	- 0,025191962
chr5	124750000	125000000	0,032584592	1	- 0,025191962
chr5	124950000	125200000	0,029023598	1	-0,02718856
chr5	125000000	125250000	0,003167766	1	- 0,028442168
chr5	125050000	125300000	0,005315929	1	- 0,029570584
chr5	128700000	128950000	0,028019358	1	- 0,016109308
chr5	128750000	129000000	0,019220557	1	- 0,016109308
chr5	128950000	129200000	0,010817582	1	- 0,017033559
chr5	129000000	129250000	0,000602345	1	- 0,017915237
chr5	129050000	129300000	0,00121639	1	- 0,018491855
chr5	129100000	129350000	0,00127886	1	- 0,018636669
chr5	129150000	129400000	0,001293693	1	- 0,018640032
chr5	129200000	129450000	0,044348293	1	- 0,020065104
chr5	129850000	130100000	0,006130791	1	-

					0,043875085
chr5	129900000	130150000	0,013679415	1	-0,046016028
chr5	130850000	131100000	0,041230125	1	-0,01428813
chr5	132850000	133100000	0,03220231	1	-0,006267106
chr5	135800000	136050000	0,049093366	1	-0,013437777
chr5	141850000	142100000	0,028903696	1	-0,009456071
chr5	141900000	142150000	0,029666467	1	-0,010274056
chr5	143900000	144150000	0,007290319	1	-0,027801002
chr5	143950000	144200000	0,001543531	1	-0,027216418
chr5	144000000	144250000	0,003564469	1	-0,027216418
chr5	144050000	144300000	0,001103653	1	-0,027216418
chr5	144100000	144350000	0,001754584	1	-0,027216418
chr5	144150000	144400000	0,028888176	1	-0,027216418
chr5	144450000	144700000	0,016904873	1	-0,033578168
chr5	144500000	144750000	0,004568549	1	-0,034936776

chr5	144550000	144800000	0,001063809	1	- 0,035951026
chr5	144600000	144850000	0,000130319	1	- 0,036672574
chr5	144650000	144900000	7,44E-05	1	- 0,037200504
chr5	144700000	144950000	0,003780766	1	- 0,037586342
chr5	146000000	146250000	0,005339365	1	- 0,019824452
chr5	146050000	146300000	9,37E-05	1	- 0,019663121
chr5	146100000	146350000	4,69E-05	1	- 0,019960707
chr5	146150000	146400000	0,003589595	1	- 0,021053173
chr5	146700000	146950000	0,004416078	1	- 0,051267407
chr5	146750000	147000000	0,007794874	1	- 0,051267407
chr5	147450000	147700000	0,004386176	1	-0,01664263
chr5	147500000	147750000	0,008616512	1	- 0,017397241
chr5	147700000	147950000	0,041395224	1	- 0,019658093
chr5	147750000	148000000	0,00476961	1	- 0,019658093
chr5	147800000	148050000	0,012069797	1	-

					0,019658093
chr5	149050000	149300000	0,024201632	1	-0,005589701
chr5	149100000	149350000	0,018817487	1	-0,005946923
chr5	151400000	151650000	0,04555576	1	-0,042778028
chr5	151450000	151700000	0,011323742	1	-0,040188197
chr5	151950000	152200000	0,033689526	1	-0,016865643
chr5	152000000	152250000	0,002687571	1	-0,017395531
chr5	152050000	152300000	0,012853244	1	-0,017845644
chr5	152100000	152350000	0,017834841	1	-0,01818215
chr5	152150000	152400000	0,010935907	1	-0,01818215
chr5	152200000	152450000	0,006911781	1	-0,01818215
chr5	152250000	152500000	0,011496814	1	-0,01818215
chr5	152300000	152550000	0,04092546	1	-0,01818215
chr5	152900000	153150000	0,009076071	1	-0,036377299
chr5	153500000	153750000	0,048973574	1	-0,004061978
chr5	154550000	154800000	0,010177794	1	-0,041519469
chr5	154600000	154850000	0,023834924	1	-0,038917598

chr5	155550000	155800000	0,01063227	1	-0,018308609
chr5	155600000	155850000	0,000502278	1	-0,018633093
chr5	155650000	155900000	7,25E-06	1	-0,019145378
chr5	155700000	155950000	2,40E-06	1	-0,019375408
chr5	155750000	156000000	4,86E-05	1	-0,019397804
chr5	155800000	156050000	0,004557744	1	-0,019298899
chr5	156250000	156500000	0,009635371	1	-0,031846426
chr5	156300000	156550000	0,028085292	1	-0,033753214
chr5	157650000	157900000	0,019240331	1	-0,044955028
chr5	157700000	157950000	0,028162546	1	-0,044955028
chr5	157950000	158200000	0,044312818	1	-0,049305017
chr5	158000000	158250000	0,004673775	1	-0,051935356
chr5	158050000	158300000	0,017456685	1	-0,054282525
chr5	158950000	159200000	0,017963791	1	-0,036975128

chr5	160100000	160350000	0,017742316	1	-0,048357854
chr5	160150000	160400000	0,00040313	1	-0,047675629
chr5	160200000	160450000	0,000360699	1	-0,046831905
chr5	160250000	160500000	0,002469146	1	-0,045795336
chr5	160300000	160550000	0,008682897	1	-0,044333146
chr5	160350000	160600000	0,033015136	1	-0,042144819
chr5	160900000	161150000	0,015329596	1	-0,015636371
chr5	160950000	161200000	0,03725376	1	-0,016517427
chr5	161600000	161850000	0,024420196	1	-0,03479648
chr5	161650000	161900000	0,001431685	1	-0,036703472
chr5	161700000	161950000	0,000583823	1	-0,038047101
chr5	161750000	162000000	0,004902395	1	-0,038718168
chr5	161800000	162050000	0,010247573	1	-0,038693367
chr5	161850000	162100000	0,005845122	1	-0,038077745
chr5	161900000	162150000	0,000793316	1	-

					0,037113895
					-
chr5	161950000	162200000	0,002092416	1	0,036139453
					-
chr5	162000000	162250000	0,030762641	1	0,035503204
					-
chr5	162350000	162600000	0,008575371	1	0,047218415
					-
chr5	162400000	162650000	0,004307613	1	0,049487759
chr5	163250000	163500000	0,022973807	1	-0,03237221
					-
chr5	163300000	163550000	0,003343389	1	0,030958854
					-
chr5	163350000	163600000	0,049388758	1	0,028956323
					-
chr5	163950000	164200000	0,035171461	1	0,010733333
					-
chr5	164400000	164650000	0,004488834	1	0,019199469
					-
chr5	164450000	164700000	0,022237879	1	0,018070083
					-
chr5	164750000	165000000	0,029614732	1	0,014537139
					-
chr5	164800000	165050000	0,001011511	1	0,014384853
					-
chr5	164850000	165100000	0,000190588	1	0,014150924
chr5	164900000	165150000	0,000821949	1	-0,0139731
chr5	164950000	165200000	0,000896366	1	-

					0,014047122
chr5	165000000	165250000	0,000393629	1	- 0,014239915
chr5	165050000	165300000	7,69E-05	1	- 0,014443608
chr5	165100000	165350000	6,88E-06	1	- 0,014667369
chr5	165150000	165400000	0,000179808	1	- 0,014348776
chr5	165200000	165450000	0,004641338	1	- 0,014096794
chr5	165250000	165500000	0,039171728	1	- 0,014096794
chr5	165650000	165900000	0,003395019	1	- 0,009711032
chr5	165700000	165950000	0,003445533	1	- 0,009263128
chr5	165750000	166000000	0,017174122	1	- 0,009080239
chr5	165800000	166050000	0,019833468	1	- 0,009146023
chr5	165850000	166100000	0,006634543	1	- 0,009391962
chr5	165900000	166150000	0,000535178	1	- 0,009713459
chr5	165950000	166200000	9,92E-06	1	- 0,009989555
chr5	166000000	166250000	0,000144881	1	-

					0,010129113
chr5	166050000	166300000	0,022900611	1	-0,010113723
chr5	166500000	166750000	0,037699435	1	-0,021034309
chr5	168450000	168700000	0,029790678	1	-0,03030735
chr5	172950000	173200000	0,010944034	1	-0,018158045
chr5	173000000	173250000	0,043567711	1	-0,018834969
chr5	173400000	173650000	0,040419307	1	-0,034818946
chr5	174200000	174450000	0,012103777	1	-0,026795765
chr5	174250000	174500000	0,001427846	1	-0,025940304
chr5	174300000	174550000	0,016166061	1	-0,024844152
chr5	174700000	174950000	0,043892998	1	-0,018886062
chr6	1950000	2200000	0,025504672	1	-0,032008672
chr6	2000000	2250000	0,001706472	1	-0,033460569
chr6	2050000	2300000	0,00031261	1	-0,034630029
chr6	2100000	2350000	0,007424387	1	-0,03526445
chr6	5350000	5600000	0,017930128	1	-

					0,050624683
chr6	5400000	5650000	0,030531053	1	-0,050624683
chr6	6200000	6450000	0,024563005	1	-0,031414316
chr6	6250000	6500000	0,002079605	1	-0,033056233
chr6	6300000	6550000	0,000148957	1	-0,034197241
chr6	6350000	6600000	0,002451529	1	-0,034855136
chr6	6400000	6650000	0,024373091	1	-0,035051588
chr6	10050000	10300000	0,007780712	1	-0,019176361
chr6	10100000	10350000	0,013428015	1	-0,020354997
chr6	13950000	14200000	0,016683104	1	-0,010760012
chr6	16250000	16500000	0,013428664	1	-0,024317351
chr6	16300000	16550000	0,026073747	1	-0,026026487
chr6	16700000	16950000	0,003987513	1	-0,016754797
chr6	16750000	17000000	0,000216377	1	-0,016162367
chr6	16800000	17050000	0,000437318	1	-

					0,016184328
chr6	16850000	17100000	0,000171213	1	-0,015897086
chr6	16900000	17150000	0,002015181	1	-0,015793226
chr6	16950000	17200000	0,04157912	1	-0,015793226
chr6	18650000	18900000	0,004481209	1	-0,040964185
chr6	18700000	18950000	0,020393132	1	-0,038625161
chr6	19050000	19300000	0,049857119	1	-0,028454093
chr6	19100000	19350000	0,006350909	1	-0,028454093
chr6	19150000	19400000	0,000249704	1	-0,028454093
chr6	19200000	19450000	0,000348741	1	-0,027762465
chr6	19250000	19500000	0,002257485	1	-0,027486501
chr6	19300000	19550000	0,003359975	1	-0,027578704
chr6	19350000	19600000	0,001284893	1	-0,027970762
chr6	19400000	19650000	0,000203975	1	-0,02853991
chr6	19450000	19700000	0,002522545	1	-0,029095359

chr6	19500000	19750000	0,025062125	1	-0,029415241
chr6	19700000	19950000	0,012200894	1	-0,026401606
chr6	19750000	20000000	0,000953357	1	-0,025238896
chr6	19800000	20050000	0,006671685	1	-0,024430728
chr6	19850000	20100000	0,042337348	1	-0,024137372
chr6	20150000	20400000	0,017776811	1	-0,029908609
chr6	20200000	20450000	0,002241691	1	-0,031136941
chr6	20250000	20500000	0,000173072	1	-0,032161134
chr6	20300000	20550000	0,000922145	1	-0,031528722
chr6	20350000	20600000	0,002198959	1	-0,031282371
chr6	20400000	20650000	0,000784904	1	-0,031890088
chr6	20450000	20700000	0,004538006	1	-0,033644231
chr6	20800000	21050000	0,018166407	1	-0,047333819
chr6	23950000	24200000	0,01268859	1	-0,02321923
chr6	24000000	24250000	0,001182548	1	-

					0,024521393
chr6	24050000	24300000	0,029309715	1	-0,025268455
chr6	27300000	27550000	0,011267099	1	-0,020556895
chr6	27350000	27600000	0,001151613	1	-0,020563837
chr6	27400000	27650000	0,008548048	1	-0,01994642
chr6	27450000	27700000	0,021574122	1	-0,019394439
chr6	27500000	27750000	0,024514156	1	-0,018932779
chr6	27550000	27800000	0,017750084	1	-0,018556926
chr6	27600000	27850000	0,009625853	1	-0,018276939
chr6	27650000	27900000	0,003795617	1	-0,018276939
chr6	27700000	27950000	0,000791128	1	-0,018276939
chr6	27750000	28000000	5,90E-05	1	-0,018276939
chr6	27800000	28050000	0,000221525	1	-0,018276939
chr6	27850000	28100000	0,002390172	1	-0,018125325
chr6	27900000	28150000	0,007313607	1	-0,018145927

chr6	27950000	28200000	0,009804737	1	-0,018358067
chr6	28000000	28250000	0,007183174	1	-0,018732983
chr6	28050000	28300000	0,003312661	1	-0,019194315
chr6	28100000	28350000	0,001104999	1	-0,019650708
chr6	28150000	28400000	0,000297215	1	-0,020031909
chr6	28200000	28450000	5,18E-05	1	-0,020315278
chr6	28250000	28500000	5,78E-06	1	-0,020520911
chr6	28300000	28550000	0,000179348	1	-0,020136746
chr6	28350000	28600000	0,00404008	1	-0,019424258
chr6	28400000	28650000	0,030559891	1	-0,018899678
chr6	38150000	38400000	0,005667472	1	-0,018868844
chr6	38200000	38450000	0,047723383	1	-0,018868844
chr6	38500000	38750000	0,040144535	1	-0,012712077
chr6	38550000	38800000	0,004826681	1	-0,012542941

chr6	38600000	38850000	0,005608379	1	- 0,011991825
chr6	39350000	39600000	0,02956252	1	-0,02506361
chr6	39550000	39800000	0,020289591	1	- 0,024995463
chr6	40300000	40550000	0,011080455	1	- 0,050963987
chr6	40350000	40600000	0,001591195	1	- 0,053388166
chr6	40400000	40650000	0,02767935	1	- 0,055132988
chr6	40900000	41150000	0,015333991	1	- 0,024280881
chr6	40950000	41200000	0,008473276	1	-0,02339034
chr6	44900000	45150000	0,031416751	1	- 0,043495869
chr6	45950000	46200000	0,012471236	1	- 0,036749574
chr6	46000000	46250000	0,000720905	1	- 0,036749574
chr6	46050000	46300000	0,012674799	1	- 0,036749574
chr6	50600000	50850000	0,007283192	1	-0,02832411
chr6	50650000	50900000	0,001419581	1	- 0,027299694
chr6	50700000	50950000	0,020752004	1	- 0,025894912
chr6	51000000	51250000	0,00506335	1	-

					0,021409501
chr6	51050000	51300000	0,005906354	1	-0,021409501
chr6	51550000	51800000	0,009532429	1	-0,039287605
chr6	51600000	51850000	0,014317084	1	-0,041546527
chr6	52100000	52350000	0,014032767	1	-0,018644563
chr6	52350000	52600000	0,029690632	1	-0,021620579
chr6	52400000	52650000	0,016752408	1	-0,020100519
chr6	53800000	54050000	0,004990809	1	-0,009471876
chr6	53850000	54100000	0,000145166	1	-0,009387853
chr6	53900000	54150000	0,000340571	1	-0,009350854
chr6	53950000	54200000	0,000183562	1	-0,009293183
chr6	54000000	54250000	0,000199046	1	-0,009145354
chr6	54050000	54300000	0,001418532	1	-0,008903741
chr6	54100000	54350000	0,006480008	1	-0,008661581
chr6	54150000	54400000	0,008439207	1	-

					0,008592772
chr6	54200000	54450000	0,002507083	1	-0,008592772
chr6	54250000	54500000	0,028111901	1	-0,008592772
chr6	54650000	54900000	0,007216377	1	-0,01481731
chr6	57550000	57800000	0,017329056	1	-0,036014001
chr6	57600000	57850000	0,000551756	1	-0,036014001
chr6	57650000	57900000	0,005346796	1	-0,036014001
chr6	57700000	57950000	0,039111563	1	-0,036014001
chr6	58450000	58700000	0,040548744	1	-0,015632012
chr6	62150000	62400000	0,031735482	1	-0,03305524
chr6	62200000	62450000	0,005866895	1	-0,034641403
chr6	62250000	62500000	0,000410652	1	-0,036470238
chr6	62300000	62550000	5,48E-05	1	-0,036470844
chr6	62350000	62600000	0,000658558	1	-0,035765365
chr6	62400000	62650000	0,002540954	1	-0,035174179
chr6	62450000	62700000	0,003029208	1	-

					0,035051373
chr6	62500000	62750000	0,001008747	1	-0,035051373
chr6	62550000	62800000	0,000203795	1	-0,035051373
chr6	62600000	62850000	0,003695446	1	-0,034997818
chr6	62650000	62900000	0,031397084	1	-0,034777862
chr6	62850000	63100000	0,015906139	1	-0,038904815
chr6	62900000	63150000	0,001233698	1	-0,040638897
chr6	62950000	63200000	0,000342407	1	-0,042007824
chr6	63000000	63250000	0,003153523	1	-0,042749004
chr6	63050000	63300000	0,008009285	1	-0,042779167
chr6	63100000	63350000	0,006104672	1	-0,042196852
chr6	63150000	63400000	0,001153014	1	-0,04123987
chr6	63200000	63450000	0,000857862	1	-0,040215876
chr6	63250000	63500000	0,021443977	1	-0,039438928
chr6	63550000	63800000	0,041657768	1	-0,049875516

chr6	63600000	63850000	0,00453833	1	-0,053134748
chr6	64550000	64800000	0,006355106	1	-0,031506575
chr6	64600000	64850000	0,017554087	1	-0,02946553
chr6	65050000	65300000	0,034786263	1	-0,014235661
chr6	65100000	65350000	0,041092829	1	-0,015032298
chr6	65250000	65500000	0,018637874	1	-0,015514009
chr6	65300000	65550000	0,003884746	1	-0,014795225
chr6	65600000	65850000	0,018833182	1	-0,013141363
chr6	66000000	66250000	0,019275159	1	-0,02092816
chr6	66050000	66300000	0,01123017	1	-0,021584684
chr6	66100000	66350000	0,025190828	1	-0,022544213
chr6	66350000	66600000	0,04555576	1	-0,02697753
chr6	66400000	66650000	0,005762244	1	-0,026664476
chr6	66450000	66700000	0,000677651	1	-0,025891454
chr6	66500000	66750000	0,008780717	1	-0,024793836
chr6	66750000	67000000	0,041095837	1	-

					0,020950446
chr6	66800000	67050000	0,003593219	1	- 0,021113232
chr6	66850000	67100000	0,004073306	1	- 0,021514604
chr6	66900000	67150000	0,043769904	1	- 0,022192432
chr6	67150000	67400000	0,040259527	1	- 0,028236379
chr6	67200000	67450000	0,015577357	1	- 0,028706475
chr6	67250000	67500000	0,006049799	1	-0,02912068
chr6	67300000	67550000	0,002878037	1	- 0,029433471
chr6	67350000	67600000	0,00192738	1	- 0,029648044
chr6	67400000	67650000	0,001658555	1	- 0,029793301
chr6	67450000	67700000	0,001270959	1	- 0,029865103
chr6	67500000	67750000	0,000489748	1	- 0,029961981
chr6	67550000	67800000	7,28E-05	1	- 0,030174225
chr6	67600000	67850000	0,001317965	1	-0,02988822
chr6	67650000	67900000	0,030605542	1	- 0,028934728
chr6	68050000	68300000	0,029756274	1	-

					0,017661169
chr6	68300000	68550000	0,014370275	1	-0,02077594
chr6	68350000	68600000	0,019245855	1	-0,020674391
chr6	68650000	68900000	0,022637762	1	-0,016142543
chr6	68700000	68950000	0,010308384	1	-0,015082191
chr6	69100000	69350000	0,028565257	1	-0,020964368
chr6	69150000	69400000	0,002268135	1	-0,021941922
chr6	69200000	69450000	0,000215276	1	-0,022769487
chr6	69250000	69500000	0,002018188	1	-0,023228562
chr6	69300000	69550000	0,007898138	1	-0,023303161
chr6	69350000	69600000	0,012726553	1	-0,023050894
chr6	69400000	69650000	0,012429582	1	-0,022580634
chr6	69450000	69700000	0,009532583	1	-0,022021511
chr6	69500000	69750000	0,006982951	1	-0,021473258
chr6	69550000	69800000	0,004969084	1	-0,020986213

chr6	69600000	69850000	0,002471882	1	-0,020557391
chr6	69650000	69900000	0,000429155	1	-0,020161977
chr6	69700000	69950000	0,00072329	1	-0,01979019
chr6	69750000	70000000	0,023316557	1	-0,019488037
chr6	70100000	70350000	0,004211792	1	-0,026640519
chr6	70150000	70400000	0,001370268	1	-0,027907248
chr6	70200000	70450000	0,017203784	1	-0,028564691
chr6	75250000	75500000	0,012631866	1	-0,01813221
chr6	75300000	75550000	0,001438077	1	-0,019319627
chr6	75350000	75600000	0,012535344	1	-0,019890143
chr6	75400000	75650000	0,04212914	1	-0,019261033
chr6	76750000	77000000	0,031217759	1	-0,025506255
chr6	79450000	79700000	0,013201861	1	-0,027406566
chr6	79500000	79750000	0,000861008	1	-0,029163488
chr6	79550000	79800000	0,007678522	1	-0,03003061
chr6	79600000	79850000	0,032119297	1	-

					0,030127614
chr6	79900000	80150000	0,04165197	1	-0,024508297
chr6	79950000	80200000	0,005273679	1	-0,023410382
chr6	80000000	80250000	0,003235486	1	-0,022466241
chr6	80050000	80300000	0,042191181	1	-0,024509564
chr6	80250000	80500000	0,00639219	1	-0,025401799
chr6	80300000	80550000	0,027940964	1	-0,023579739
chr6	81600000	81850000	0,002448584	1	-0,019697856
chr6	81650000	81900000	0,002445042	1	-0,020630875
chr6	81700000	81950000	0,013358353	1	-0,020976519
chr6	81750000	82000000	0,018879297	1	-0,020817082
chr6	81800000	82050000	0,008178128	1	-0,020322613
chr6	81850000	82100000	0,000870842	1	-0,019698322
chr6	81900000	82150000	0,001081738	1	-0,01914358
chr6	81950000	82200000	0,012130199	1	-0,018815143

chr6	82000000	82250000	0,035609915	1	-0,018809397
chr6	82050000	82300000	0,039045739	1	-0,019142902
chr6	82100000	82350000	0,016558279	1	-0,019749868
chr6	82150000	82400000	0,001994046	1	-0,020494993
chr6	82200000	82450000	0,003031284	1	-0,021196985
chr6	83900000	84150000	0,005985844	1	-0,034503574
chr6	85450000	85700000	0,016732283	1	-0,035333971
chr6	86650000	86900000	0,041573504	1	-0,047228099
chr6	86700000	86950000	0,002371725	1	-0,045561838
chr6	86750000	87000000	0,018535594	1	-0,043072919
chr6	87250000	87500000	0,004088502	1	-0,026462559
chr6	87300000	87550000	0,013500509	1	-0,025191953
chr6	87350000	87600000	0,042755848	1	-0,024754233
chr6	87400000	87650000	0,029218712	1	-0,025131455

chr6	87450000	87700000	0,004474125	1	-0,02608742
chr6	87500000	87750000	0,003250357	1	-0,027191287
chr6	87550000	87800000	0,031068461	1	-0,027958228
chr6	87700000	87950000	0,011333473	1	-0,02624271
chr6	87750000	88000000	0,001443228	1	-0,025044972
chr6	87800000	88050000	0,009968678	1	-0,024216474
chr6	87850000	88100000	0,027886264	1	-0,02400165
chr6	87900000	88150000	0,020116554	1	-0,024404665
chr6	87950000	88200000	0,00362117	1	-0,025217883
chr6	88000000	88250000	0,001255073	1	-0,026125858
chr6	88050000	88300000	0,022517515	1	-0,026806972
chr6	88350000	88600000	0,017831188	1	-0,019575818
chr6	88400000	88650000	0,004098996	1	-0,020539683
chr6	88800000	89050000	0,008773414	1	-0,030401213
chr6	88850000	89100000	0,023945873	1	-0,030401213
chr6	91500000	91750000	0,018870418	1	-

					0,020854082
chr6	91550000	91800000	0,024546493	1	-0,019869977
chr6	91700000	91950000	0,018744836	1	-0,019044001
chr6	91750000	92000000	0,00114949	1	-0,019190802
chr6	91800000	92050000	0,001994514	1	-0,019190802
chr6	91850000	92100000	0,008418037	1	-0,019190802
chr6	91900000	92150000	0,008522093	1	-0,019190802
chr6	91950000	92200000	0,002579175	1	-0,019490098
chr6	92000000	92250000	0,00018933	1	-0,020026787
chr6	92050000	92300000	7,00E-06	1	-0,020462918
chr6	92100000	92350000	5,01E-06	1	-0,020676957
chr6	92150000	92400000	6,31E-05	1	-0,020661871
chr6	92200000	92450000	0,001911542	1	-0,020519893
chr6	92250000	92500000	0,010847953	1	-0,020421167
chr6	92300000	92550000	0,016100935	1	-

					0,020535425
chr6	92350000	92600000	0,005666251	1	-0,020942935
chr6	92400000	92650000	0,003701453	1	-0,021583084
chr6	93550000	93800000	0,034610556	1	-0,022416057
chr6	93600000	93850000	0,024329824	1	-0,020940549
chr6	94200000	94450000	0,007408858	1	-0,011185471
chr6	94250000	94500000	0,040353247	1	-0,012146792
chr6	94550000	94800000	0,04420674	1	-0,016425001
chr6	94600000	94850000	0,033976585	1	-0,01517482
chr6	96300000	96550000	0,037626269	1	-0,020636891
chr6	96350000	96600000	0,006468741	1	-0,020636891
chr6	96400000	96650000	0,000356598	1	-0,020636891
chr6	96450000	96700000	7,26E-05	1	-0,020636891
chr6	96500000	96750000	0,000359638	1	-0,020887218
chr6	96550000	96800000	0,000301586	1	-0,020871771

chr6	96600000	96850000	0,000100955	1	-0,020658141
chr6	96650000	96900000	0,002967762	1	-0,020373724
chr6	96700000	96950000	0,042330696	1	-0,020177112
chr6	97150000	97400000	0,005100735	1	-0,032498591
chr6	97200000	97450000	0,002551394	1	-0,033978426
chr6	97950000	98200000	0,036614848	1	-0,03137098
chr6	98000000	98250000	0,00906353	1	-0,031123263
chr6	98300000	98550000	0,038890162	1	-0,022887736
chr6	98350000	98600000	0,001588072	1	-0,02349038
chr6	98400000	98650000	0,0029983	1	-0,024044706
chr6	98450000	98700000	0,006337935	1	-0,024296907
chr6	98500000	98750000	0,002346797	1	-0,024133968
chr6	98550000	98800000	0,000302566	1	-0,023613965
chr6	98600000	98850000	0,001804573	1	-0,022948576
chr6	98650000	98900000	0,007943912	1	-0,022453505

chr6	98700000	98950000	0,006688701	1	-0,022453505
chr6	98750000	99000000	0,001552274	1	-0,022453505
chr6	98800000	99050000	0,022987892	1	-0,022453505
chr6	99200000	99450000	0,017215066	1	-0,036511162
chr6	99250000	99500000	0,005728765	1	-0,038813113
chr6	102450000	102700000	0,006854077	1	-0,016072205
chr6	102500000	102750000	0,000303857	1	-0,015783055
chr6	102550000	102800000	0,001344731	1	-0,015303711
chr6	102600000	102850000	0,013646212	1	-0,014708443
chr6	102650000	102900000	0,045801518	1	-0,01410764
chr6	102800000	103050000	0,018821212	1	-0,013366756
chr6	102850000	103100000	0,001660399	1	-0,013622519
chr6	102900000	103150000	0,001122056	1	-0,014034876
chr6	102950000	103200000	0,009193855	1	-0,014466341
chr6	103000000	103250000	0,018198179	1	-

					0,014772503
chr6	103050000	103300000	0,010754023	1	-0,014847655
chr6	103100000	103350000	0,001661137	1	-0,01465871
chr6	103150000	103400000	0,000496825	1	-0,014256337
chr6	103200000	103450000	0,006432804	1	-0,013750588
chr6	103250000	103500000	0,028180446	1	-0,013257634
chr6	103300000	103550000	0,048302062	1	-0,012843889
chr6	103350000	103600000	0,047073249	1	-0,012487896
chr6	103400000	103650000	0,038397409	1	-0,012096199
chr6	103450000	103700000	0,046638747	1	-0,011560301
chr6	104150000	104400000	0,010462402	1	-0,017754986
chr6	104400000	104650000	0,016581408	1	-0,015959837
chr6	104450000	104700000	0,018231189	1	-0,014988076
chr6	104650000	104900000	0,008960008	1	-0,0159986
chr6	104700000	104950000	0,020182365	1	-0,016882015
chr6	105200000	105450000	0,014064988	1	-0,00890827

chr6	105250000	105500000	0,001949823	1	-0,00839781
chr6	105300000	105550000	0,005433055	1	-0,008127085
chr6	105350000	105600000	0,003196205	1	-0,00811495
chr6	109150000	109400000	0,042140284	1	-0,018100217
chr6	109200000	109450000	0,006176383	1	-0,017953797
chr6	109250000	109500000	0,000613346	1	-0,017444049
chr6	109300000	109550000	0,009503915	1	-0,016977439
chr6	114550000	114800000	0,047695716	1	-0,014581096
chr6	114800000	115050000	0,045178585	1	-0,012579386
chr6	115350000	115600000	0,018986236	1	-0,025294783
chr6	115400000	115650000	0,002119505	1	-0,025451608
chr6	115450000	115700000	6,14E-05	1	-0,025600991
chr6	115500000	115750000	4,91E-07	1	-0,025822314
chr6	115550000	115800000	0,00012412	1	-0,025976417
chr6	115600000	115850000	0,007624028	1	-0,025898949

chr6	116000000	116250000	0,011364222	1	-<br>0,038565276
chr6	117350000	117600000	0,049250737	1	-<br>0,021598495
chr6	118200000	118450000	0,006002096	1	-<br>0,045191116
chr6	118250000	118500000	0,002309067	1	-0,04329491
chr6	119500000	119750000	0,030827664	1	-<br>0,029253374
chr6	120050000	120300000	0,019458212	1	-<br>0,010272637
chr6	120100000	120350000	0,000681052	1	-0,01043338
chr6	120150000	120400000	0,00213162	1	-0,01043338
chr6	120550000	120800000	0,037567787	1	-<br>0,015503849
chr6	120600000	120850000	0,006650971	1	-<br>0,016502498
chr6	120650000	120900000	0,014548043	1	-<br>0,017109201
chr6	121200000	121450000	0,009102216	1	-<br>0,039910289
chr6	121250000	121500000	0,015925701	1	-<br>0,042285388
chr6	121700000	121950000	0,033794292	1	-<br>0,021621184
chr6	121750000	122000000	0,002158586	1	-<br>0,022451412
chr6	121800000	122050000	0,028810693	1	-

					0,023897592
chr6	122250000	122500000	0,019189572	1	-0,038728454
chr6	122300000	122550000	0,001982656	1	-0,038728454
chr6	122350000	122600000	6,33E-05	1	-0,038728454
chr6	122400000	122650000	1,54E-05	1	-0,039430857
chr6	122450000	122700000	6,17E-05	1	-0,039701461
chr6	122500000	122750000	3,89E-05	1	-0,039631542
chr6	122550000	122800000	8,35E-06	1	-0,03934718
chr6	122600000	122850000	7,53E-05	1	-0,039811753
chr6	122650000	122900000	0,000412617	1	-0,040270774
chr6	122700000	122950000	0,000439474	1	-0,040367402
chr6	122750000	123000000	9,68E-05	1	-0,039878879
chr6	122800000	123050000	0,000517219	1	-0,038705131
chr6	122850000	123100000	0,012128448	1	-0,036901842
chr6	123250000	123500000	0,021952124	1	-0,025200206

chr6	123300000	123550000	0,001926667	1	-0,02507055
chr6	123350000	123600000	0,000230247	1	-0,024784083
chr6	123400000	123650000	0,000493848	1	-0,024304161
chr6	123450000	123700000	0,002558687	1	-0,023704614
chr6	123500000	123750000	0,007640682	1	-0,023704614
chr6	123550000	123800000	0,012018116	1	-0,023704614
chr6	123600000	123850000	0,011282768	1	-0,023704614
chr6	123650000	123900000	0,008762857	1	-0,023704614
chr6	123700000	123950000	0,011591753	1	-0,023108681
chr6	123750000	124000000	0,04295103	1	-0,022606161
chr6	124850000	125100000	0,017813174	1	-0,020669622
chr6	125700000	125950000	0,041315732	1	-0,028026331
chr6	126650000	126900000	0,013216679	1	-0,043944772
chr6	126700000	126950000	0,001726188	1	-0,042305876
chr6	126750000	127000000	0,027550801	1	-

					0,039905913
chr6	128400000	128650000	0,039995414	1	- 0,028876281
chr6	129600000	129850000	0,046786231	1	- 0,027806271
chr6	129650000	129900000	0,002801806	1	- 0,029673782
chr6	129700000	129950000	0,004950192	1	- 0,031029305
chr6	129750000	130000000	0,029305024	1	- 0,031644419
chr6	129800000	130050000	0,039479979	1	- 0,031442042
chr6	129850000	130100000	0,011731499	1	- 0,030563224
chr6	129900000	130150000	0,00541417	1	- 0,030036798
chr6	130350000	130600000	0,019005735	1	- 0,050126547
chr6	130400000	130650000	0,001160547	1	- 0,048830691
chr6	130450000	130700000	0,006473897	1	- 0,046507973
chr6	133000000	133250000	0,042090542	1	- 0,020624026
chr6	133050000	133300000	0,009542466	1	- 0,022240986
chr6	134850000	135100000	0,00272431	1	-

					0,011756516
chr6	134900000	135150000	0,024281088	1	- 0,011103164
chr6	136950000	137200000	0,034577017	1	- 0,009932875
chr6	137000000	137250000	0,000698074	1	- 0,009892257
chr6	137050000	137300000	3,52E-05	1	- 0,009731848
chr6	137100000	137350000	0,002320009	1	- 0,009369098
chr6	137150000	137400000	0,037833967	1	- 0,008788969
chr6	139100000	139350000	0,017805646	1	- 0,018802591
chr6	139150000	139400000	0,036438168	1	- 0,017158921
chr6	143600000	143850000	0,01222853	1	- 0,008055412
chr6	143650000	143900000	0,02497434	1	- 0,008595301
chr6	145800000	146050000	0,003597721	1	- 0,026162164
chr6	145850000	146100000	0,000836132	1	- 0,027642023
chr6	145900000	146150000	0,00224677	1	- 0,028062863
chr6	145950000	146200000	0,000828202	1	-

					0,027807005
chr6	146000000	146250000	0,000536384	1	- 0,027665482
chr6	146050000	146300000	0,01323278	1	- 0,029058123
chr6	146400000	146650000	0,025450114	1	- 0,039000419
chr6	146450000	146700000	0,001767187	1	- 0,038133037
chr6	146500000	146750000	0,003879935	1	- 0,038108642
chr6	148200000	148450000	0,04134453	1	- 0,008134521
chr6	148800000	149050000	0,039770102	1	- 0,029221814
chr6	151950000	152200000	0,010442159	1	- 0,028659742
chr6	152000000	152250000	0,009235081	1	- 0,029533792
chr6	152050000	152300000	0,016804013	1	- 0,030441202
chr6	152100000	152350000	0,019570627	1	- 0,031036256
chr6	152150000	152400000	0,00814893	1	- 0,030932728
chr6	152200000	152450000	0,001543354	1	- 0,029817997
chr6	152250000	152500000	0,03217886	1	-

					0,029068914
chr6	153600000	153850000	0,008256692	1	-0,032769459
chr6	153650000	153900000	0,047175867	1	-0,030989477
chr6	153800000	154050000	0,045069065	1	-0,029274727
chr6	153850000	154100000	0,003271899	1	-0,029274727
chr6	153900000	154150000	0,001841254	1	-0,029274727
chr6	153950000	154200000	0,006639867	1	-0,028994656
chr6	154000000	154250000	0,003672516	1	-0,029157871
chr6	154050000	154300000	0,00162775	1	-0,029873706
chr6	154100000	154350000	0,042531997	1	-0,030711952
chr6	157050000	157300000	0,0151842	1	-0,025472469
chr6	159700000	159950000	0,039692344	1	-0,006542666
chr6	159750000	160000000	0,005955795	1	-0,006542666
chr6	160550000	160800000	0,009818879	1	-0,018725421
chr6	160600000	160850000	0,017163761	1	-

					0,018777801
chr6	160650000	160900000	0,012709781	1	- 0,020169609
chr6	161150000	161400000	0,045802253	1	- 0,056944342
chr6	161200000	161450000	0,002979894	1	- 0,054698152
chr6	161250000	161500000	0,032248352	1	-0,05120827
chr6	164200000	164450000	0,027584786	1	- 0,020246309
chr6	164400000	164650000	0,015616828	1	- 0,019726339
chr6	165200000	165450000	0,008210361	1	- 0,042917799
chr6	165250000	165500000	0,000498268	1	- 0,044820249
chr6	165300000	165550000	0,003323595	1	- 0,046099524
chr6	165350000	165600000	0,028863444	1	- 0,046614559
chr6	165650000	165900000	0,017632275	1	- 0,037743166
chr6	165700000	165950000	0,002933971	1	-0,03626187
chr6	165750000	166000000	0,000164332	1	- 0,035190605
chr6	165800000	166050000	0,00012916	1	- 0,034504707
chr6	165850000	166100000	0,002593558	1	-

					0,034156543
chr6	165900000	166150000	0,014534985	1	- 0,034127114
chr6	165950000	166200000	0,034946639	1	- 0,034439212
chr6	166000000	166250000	0,042309226	1	- 0,035128714
chr6	166050000	166300000	0,024516195	1	- 0,036187085
chr6	166100000	166350000	0,00501288	1	- 0,037503296
chr6	166150000	166400000	0,000614652	1	- 0,038843796
chr6	166200000	166450000	0,007558315	1	- 0,039894732
chr6	167000000	167250000	0,014118186	1	- 0,014474383
chr6	167700000	167950000	0,021484841	1	-0,03114321
chr6	167750000	168000000	0,031657128	1	-0,03114321
chr6	170000000	170250000	0,014135119	1	- 0,025720164
chr6	170500000	170750000	0,001358195	1	- 0,012092352
chr6	170550000	170800000	0,031996887	1	- 0,012991849
chr7	3100000	3350000	0,030774626	1	- 0,038309184
chr7	3150000	3400000	0,003879966	1	-

					0,036564181
chr7	3600000	3850000	0,03004224	1	-0,020566806
chr7	4050000	4300000	0,014608949	1	-0,038943841
chr7	4100000	4350000	0,008089723	1	-0,041459702
chr7	4650000	4900000	0,005588011	1	-0,01531633
chr7	4700000	4950000	0,010121424	1	-0,015640109
chr7	4750000	5000000	0,004701006	1	-0,015640109
chr7	4800000	5050000	0,024441515	1	-0,015640109
chr7	7150000	7400000	0,032195307	1	-0,009920523
chr7	7200000	7450000	0,010107177	1	-0,009920523
chr7	8500000	8750000	0,021344965	1	-0,03104592
chr7	8850000	9100000	0,035188009	1	-0,020905023
chr7	8900000	9150000	0,001133855	1	-0,020998033
chr7	8950000	9200000	9,16E-06	1	-0,021152523
chr7	9000000	9250000	7,07E-05	1	-0,021407586
chr7	9050000	9300000	0,000531289	1	-

					0,021786867
chr7	9100000	9350000	0,001974689	1	-<br>0,022238154
chr7	9150000	9400000	0,003802822	1	-<br>0,022633381
chr7	9200000	9450000	0,003271725	1	-<br>0,022831394
chr7	9250000	9500000	0,000942127	1	-<br>0,022748628
chr7	9300000	9550000	9,90E-05	1	-<br>0,022396419
chr7	9350000	9600000	0,000610134	1	-<br>0,021991054
chr7	9400000	9650000	0,005161296	1	-<br>0,021573506
chr7	9450000	9700000	0,015624886	1	-<br>0,021138187
chr7	9500000	9750000	0,028941964	1	-<br>0,020675583
chr7	9550000	9800000	0,049262997	1	-<br>0,020064159
chr7	10000000	10250000	0,008518745	1	-<br>0,012765142
chr7	10600000	10850000	0,030480818	1	-<br>0,023703752
chr7	11150000	11400000	0,030584518	1	-<br>0,013438412
chr7	11200000	11450000	0,003789102	1	-

					0,014463074
chr7	11250000	11500000	0,02346453	1	-0,015073437
chr7	16200000	16450000	0,039073757	1	-0,036304375
chr7	16250000	16500000	0,012228301	1	-0,03930981
chr7	16900000	17150000	0,046593427	1	-0,006195048
chr7	17050000	17300000	0,013741996	1	-0,00635147
chr7	17100000	17350000	0,005776829	1	-0,006005762
chr7	19750000	20000000	0,036596702	1	-0,023150826
chr7	19800000	20050000	0,004750416	1	-0,024922401
chr7	20700000	20950000	0,043319059	1	-0,02285682
chr7	21050000	21300000	0,048126216	1	-0,014398632
chr7	21550000	21800000	0,038202291	1	-0,027076175
chr7	21600000	21850000	0,010290662	1	-0,028283677
chr7	21650000	21900000	0,001039861	1	-0,02931469
chr7	21700000	21950000	0,00111592	1	-0,030152355
chr7	21750000	22000000	0,025691639	1	-0,030705777
chr7	25500000	25750000	0,00282082	1	-

					0,019030415
chr7	25550000	25800000	2,21E-05	1	-0,019861821
chr7	25600000	25850000	3,11E-07	1	-0,020173795
chr7	25650000	25900000	5,35E-05	1	-0,020225907
chr7	25700000	25950000	0,002358914	1	-0,020206898
chr7	25750000	26000000	0,039126967	1	-0,020182922
chr7	28100000	28350000	0,044047329	1	-0,02505823
chr7	31150000	31400000	0,040588208	1	-0,034279908
chr7	31200000	31450000	0,003839926	1	-0,032787984
chr7	31250000	31500000	0,043853771	1	-0,030786074
chr7	31600000	31850000	0,005130259	1	-0,023376885
chr7	32100000	32350000	0,012245287	1	-0,040916914
chr7	32150000	32400000	0,00890636	1	-0,043344701
chr7	34600000	34850000	0,026623213	1	-0,036516964
chr7	34650000	34900000	0,030102688	1	-0,036516964

chr7	35000000	35250000	0,023249385	1	-0,023436624
chr7	35200000	35450000	0,014827707	1	-0,023112956
chr7	35950000	36200000	0,02292752	1	-0,012771336
chr7	36850000	37100000	0,048537179	1	-0,061533762
chr7	36900000	37150000	0,005625097	1	-0,061264448
chr7	36950000	37200000	0,000241309	1	-0,060226909
chr7	37000000	37250000	0,001162097	1	-0,058465588
chr7	37050000	37300000	0,014285831	1	-0,055975898
chr7	37900000	38150000	0,043417184	1	-0,01850397
chr7	38550000	38800000	0,02880107	1	-0,06352259
chr7	38600000	38850000	0,001413826	1	-0,06352259
chr7	38650000	38900000	0,009433596	1	-0,06352259
chr7	38700000	38950000	0,045029924	1	-0,06352259
chr7	38900000	39150000	0,041700272	1	-0,055868666
chr7	42400000	42650000	0,04603476	1	-0,01660675
chr7	47150000	47400000	0,048295573	1	-0,018958764
chr7	48400000	48650000	0,005981231	1	-0,027856016

chr7	48450000	48700000	0,03530892	1	- 0,025973832
chr7	48800000	49050000	0,00628212	1	- 0,018840028
chr7	48850000	49100000	5,20E-05	1	- 0,018920669
chr7	48900000	49150000	7,01E-06	1	- 0,018749035
chr7	48950000	49200000	0,000302362	1	- 0,018226668
chr7	49000000	49250000	0,011579393	1	- 0,017299984
chr7	51800000	52050000	0,012043254	1	- 0,053387012
chr7	54250000	54500000	0,034437377	1	- 0,030311949
chr7	54300000	54550000	0,039551328	1	- 0,032556595
chr7	55600000	55850000	0,026574204	1	- 0,014849492
chr7	56500000	56750000	0,010816491	1	- 0,033550584
chr7	56800000	57050000	0,011342254	1	- 0,027819548
chr7	56850000	57100000	0,032927424	1	- 0,029690844
chr7	57100000	57350000	0,024528705	1	-0,03507933
chr7	57150000	57400000	0,002406569	1	-

					0,033739558
chr7	57200000	57450000	0,022066956	1	-0,031945764
chr7	57400000	57650000	0,030281015	1	-0,02896363
chr7	57450000	57700000	0,002937463	1	-0,030097868
chr7	57500000	57750000	0,017764627	1	-0,031497486
chr7	57650000	57900000	0,014634404	1	-0,032730321
chr7	57700000	57950000	0,003302677	1	-0,031200874
chr7	61800000	62050000	0,011429639	1	-0,045763641
chr7	61850000	62100000	0,043009773	1	-0,048312063
chr7	62100000	62350000	0,033408529	1	-0,036443628
chr7	62150000	62400000	0,001725609	1	-0,036927577
chr7	62200000	62450000	0,000105102	1	-0,037340122
chr7	62250000	62500000	0,000151398	1	-0,037220115
chr7	62300000	62550000	0,000201398	1	-0,036230463
chr7	62350000	62600000	0,009557883	1	-0,034683502

chr7	63000000	63250000	0,031841993	1	-0,027293777
chr7	63050000	63300000	0,004787484	1	-0,028900002
chr7	63100000	63350000	0,003071968	1	-0,029928279
chr7	63150000	63400000	0,014441349	1	-0,030456761
chr7	63450000	63700000	0,019649938	1	-0,037818869
chr7	63500000	63750000	0,021357831	1	-0,040049115
chr7	67550000	67800000	0,024506533	1	-0,048033834
chr7	67600000	67850000	0,009014517	1	-0,048623237
chr7	67650000	67900000	0,002393163	1	-0,048871245
chr7	67700000	67950000	0,000306025	1	-0,048709669
chr7	67750000	68000000	2,55E-05	1	-0,048097182
chr7	67800000	68050000	0,00037472	1	-0,047055183
chr7	67850000	68100000	0,003967744	1	-0,045675243
chr7	67900000	68150000	0,016187405	1	-0,044086618

chr7	67950000	68200000	0,038268572	1	-<br>0,042408462
chr7	68400000	68650000	0,037911526	1	-<br>0,029937089
chr7	68450000	68700000	0,007074038	1	-<br>0,029921848
chr7	68500000	68750000	0,000421497	1	-<br>0,029921848
chr7	68550000	68800000	4,13E-06	1	-<br>0,029921848
chr7	68600000	68850000	1,25E-06	1	-<br>0,029921848
chr7	68650000	68900000	0,000257927	1	-<br>0,029921848
chr7	68700000	68950000	0,007273275	1	-0,0300586
chr7	69000000	69250000	0,007368337	1	-<br>0,024643172
chr7	69050000	69300000	0,042191181	1	-<br>0,023380709
chr7	69300000	69550000	0,026544618	1	-<br>0,030696628
chr7	69350000	69600000	0,002112209	1	-<br>0,029607267
chr7	69400000	69650000	0,017577516	1	-<br>0,028042741
chr7	69650000	69900000	0,012506777	1	-<br>0,024284511
chr7	70050000	70300000	0,026546472	1	-

					0,038536767
chr7	70100000	70350000	0,000759559	1	-0,040632807
chr7	70150000	70400000	0,000376775	1	-0,040146272
chr7	70200000	70450000	0,000545086	1	-0,040247076
chr7	70250000	70500000	0,000170494	1	-0,040758249
chr7	70300000	70550000	0,001960365	1	-0,040975824
chr7	70350000	70600000	0,019696446	1	-0,040975824
chr7	70650000	70900000	0,028704274	1	-0,048151764
chr7	70700000	70950000	0,009887659	1	-0,04995994
chr7	70750000	71000000	0,001274524	1	-0,051629909
chr7	70800000	71050000	0,000751417	1	-0,053074865
chr7	70850000	71100000	0,021282849	1	-0,054102641
chr7	71300000	71550000	0,043530198	1	-0,027540515
chr7	71600000	71850000	0,019462298	1	-0,03240897
chr7	76400000	76650000	0,015636751	1	-0,016165443
chr7	76450000	76700000	0,012321988	1	-

					0,015372704
chr7	77250000	77500000	0,046872469	1	-0,017075378
chr7	77900000	78150000	0,045561825	1	-0,032007006
chr7	77950000	78200000	0,007681466	1	-0,030413455
chr7	88250000	88500000	0,028158356	1	-0,029068544
chr7	88600000	88850000	0,004684931	1	-0,022941184
chr7	88650000	88900000	0,025223652	1	-0,0239317
chr7	88750000	89000000	0,036857492	1	-0,024621423
chr7	88800000	89050000	0,00560915	1	-0,024621423
chr7	88850000	89100000	0,001650762	1	-0,024621423
chr7	88900000	89150000	0,019880742	1	-0,024621423
chr7	89050000	89300000	0,031392219	1	-0,023448585
chr7	89100000	89350000	0,005431541	1	-0,022355452
chr7	89450000	89700000	0,020626494	1	-0,029135897
chr7	90700000	90950000	0,013045239	1	-0,010126166

chr7	91550000	91800000	0,008599522	1	-0,00601382
chr7	92900000	93150000	0,013828803	1	- 0,017346435
chr7	93350000	93600000	0,007815259	1	-0,01116438
chr7	93400000	93650000	0,042873682	1	-0,01056547
chr7	93800000	94050000	0,03803449	1	- 0,017921235
chr7	93850000	94100000	0,01060332	1	- 0,018137162
chr7	93900000	94150000	0,002810352	1	- 0,018320701
chr7	93950000	94200000	0,001086236	1	- 0,018554542
chr7	94000000	94250000	0,001330725	1	-0,01892556
chr7	94050000	94300000	0,004589963	1	- 0,019498076
chr7	94100000	94350000	0,018559716	1	-0,02029292
chr7	94700000	94950000	0,004748335	1	- 0,032769965
chr7	94750000	95000000	0,016516365	1	- 0,034323634
chr7	96250000	96500000	0,04960507	1	- 0,040979209
chr7	96300000	96550000	0,006484659	1	- 0,040979209
chr7	96350000	96600000	0,000271838	1	- 0,040979209
chr7	96400000	96650000	0,000254873	1	-

					0,040979209
chr7	96450000	96700000	0,000576469	1	-0,041373006
chr7	96500000	96750000	0,00020632	1	-0,041144409
chr7	96550000	96800000	0,002299694	1	-0,040533544
chr7	96600000	96850000	0,041683319	1	-0,044870686
chr7	96900000	97150000	0,011748092	1	-0,053561624
chr7	96950000	97200000	0,001144723	1	-0,053561624
chr7	97000000	97250000	0,017828516	1	-0,055275016
chr7	103500000	103750000	0,005050059	1	-0,048269562
chr7	103550000	103800000	0,000207372	1	-0,048269562
chr7	103600000	103850000	0,000811635	1	-0,048322206
chr7	103650000	103900000	0,0027171	1	-0,048631247
chr7	103700000	103950000	0,002219861	1	-0,048230086
chr7	103750000	104000000	0,00046206	1	-0,047381217
chr7	103800000	104050000	2,16E-05	1	-

					0,046443021
chr7	103850000	104100000	2,60E-06	1	-0,045768282
chr7	103900000	104150000	9,23E-05	1	-0,045562666
chr7	103950000	104200000	0,009783456	1	-0,045778725
chr7	105500000	105750000	0,039610706	1	-0,007928105
chr7	108000000	108250000	0,0082422	1	-0,021581434
chr7	108050000	108300000	0,003333434	1	-0,022412728
chr7	108100000	108350000	0,043520005	1	-0,023994173
chr7	108400000	108650000	0,026962499	1	-0,031250406
chr7	108450000	108700000	0,007109666	1	-0,029607125
chr7	108900000	109150000	0,022763025	1	-0,017426713
chr7	108950000	109200000	0,017345119	1	-0,017426713
chr7	109300000	109550000	0,00955647	1	-0,022836046
chr7	109350000	109600000	0,000679857	1	-0,023757711
chr7	109400000	109650000	0,00382995	1	-

					0,024410641
chr7	110600000	110850000	0,003286005	1	-0,043162372
chr7	110650000	110900000	0,025772341	1	-0,045227131
chr7	111800000	112050000	0,023320394	1	-0,019701157
chr7	111850000	112100000	0,011058315	1	-0,020460011
chr7	111900000	112150000	0,008435502	1	-0,02302207
chr7	111950000	112200000	0,016408148	1	-0,023935084
chr7	112400000	112650000	0,0352938	1	-0,039789138
chr7	112450000	112700000	0,002336019	1	-0,038411861
chr7	112500000	112750000	0,014357179	1	-0,036543044
chr7	112700000	112950000	0,043506221	1	-0,032459454
chr7	112750000	113000000	0,003636672	1	-0,033209894
chr7	112800000	113050000	0,002636687	1	-0,034304512
chr7	112850000	113100000	0,026557287	1	-0,034304512
chr7	113050000	113300000	0,037530997	1	-0,036027038

chr7	113100000	113350000	0,007236375	1	-0,037632469
chr7	113150000	113400000	0,000792416	1	-0,039091397
chr7	113200000	113450000	0,012981277	1	-0,040205156
chr7	113900000	114150000	0,022582968	1	-0,042387808
chr7	116700000	116950000	0,006994579	1	-0,012049609
chr7	116750000	117000000	0,00064985	1	-0,012049609
chr7	116800000	117050000	0,017357106	1	-0,012361827
chr7	117550000	117800000	0,019322049	1	-0,010128934
chr7	118950000	119200000	0,033194082	1	-0,022802006
chr7	119000000	119250000	0,002323141	1	-0,021996551
chr7	119050000	119300000	0,014105236	1	-0,021277412
chr7	119100000	119350000	0,038681124	1	-0,021277412
chr7	119150000	119400000	0,026724627	1	-0,021277412
chr7	119200000	119450000	0,004154756	1	-0,021277412

chr7	119250000	119500000	0,002618608	1	-0,021156231
chr7	119300000	119550000	0,030765509	1	-0,020594517
chr7	119550000	119800000	0,037747496	1	-0,024017864
chr7	119600000	119850000	0,020241995	1	-0,025016346
chr7	119650000	119900000	0,011016177	1	-0,02587075
chr7	119700000	119950000	0,005077921	1	-0,026610422
chr7	119750000	120000000	0,001328164	1	-0,02727225
chr7	119800000	120050000	0,000124506	1	-0,027864858
chr7	119850000	120100000	0,000946762	1	-0,028350433
chr7	119900000	120150000	0,024525995	1	-0,028652275
chr7	121300000	121550000	0,020584014	1	-0,021339415
chr7	121350000	121600000	0,003165865	1	-0,020554447
chr7	121400000	121650000	0,024645965	1	-0,019735993
chr7	121600000	121850000	0,032411252	1	-0,017484019
chr7	121650000	121900000	0,021410908	1	-0,017034656

chr7	121700000	121950000	0,01849928	1	-0,016571656
chr7	121750000	122000000	0,018832417	1	-0,016141665
chr7	121800000	122050000	0,015821856	1	-0,015845876
chr7	121850000	122100000	0,00674076	1	-0,01581221
chr7	121900000	122150000	0,000883609	1	-0,016161577
chr7	121950000	122200000	0,00449016	1	-0,016966374
chr7	122650000	122900000	0,017653171	1	-0,037655057
chr7	122700000	122950000	0,002660748	1	-0,039695996
chr7	123550000	123800000	0,004684148	1	-0,030065967
chr7	123600000	123850000	0,029445968	1	-0,030065967
chr7	123800000	124050000	0,040761027	1	-0,027129849
chr7	123850000	124100000	0,003488053	1	-0,025628372
chr7	123900000	124150000	0,025354485	1	-0,024525348
chr7	124500000	124750000	0,032815165	1	-0,054321226
chr7	124550000	124800000	0,015288415	1	-

							0,055306966
chr7	124600000	124850000		0,00672947		1	-0,056047095
chr7	124650000	124900000		0,002387827		1	-0,056455655
chr7	124700000	124950000		0,00046804		1	-0,056413648
chr7	124750000	125000000		3,59E-05		1	-0,055789743
chr7	124800000	125050000		0,000309881		1	-0,054460441
chr7	124850000	125100000		0,006516478		1	-0,054460441
chr7	125850000	126100000		0,011182477		1	-0,040259181
chr7	125900000	126150000		0,047525168		1	-0,037765658
chr7	126200000	126450000		0,009076071		1	-0,030809429
chr7	126250000	126500000		0,000394214		1	-0,031104684
chr7	126300000	126550000		9,63E-05		1	-0,031630839
chr7	126350000	126600000		0,001193608		1	-0,032311756
chr7	126400000	126650000		0,004183117		1	-0,032311756
chr7	126450000	126700000		0,005601213		1	-

					0,032311756
chr7	126500000	126750000	0,002690726	1	-0,032311756
chr7	126550000	126800000	0,000409376	1	-0,032311756
chr7	126600000	126850000	0,005439643	1	-0,033012521
chr7	133250000	133500000	0,022279774	1	-0,062032659
chr7	133300000	133550000	0,009326115	1	-0,062032659
chr7	134800000	135050000	0,036255764	1	-0,01577738
chr7	135200000	135450000	0,029954044	1	-0,007945662
chr7	135250000	135500000	0,002998752	1	-0,00749186
chr7	136150000	136400000	0,009824274	1	-0,038892703
chr7	136200000	136450000	0,008777501	1	-0,037154641
chr7	136500000	136750000	0,014446585	1	-0,030781155
chr7	136550000	136800000	0,001735267	1	-0,030781155
chr7	136600000	136850000	6,16E-05	1	-0,030781155
chr7	136650000	136900000	7,04E-05	1	-0,030781155
chr7	136700000	136950000	0,000479159	1	-

					0,030643377
chr7	136750000	137000000	0,000631835	1	-0,030709678
chr7	136800000	137050000	0,000162413	1	-0,030998142
chr7	136850000	137100000	0,001366298	1	-0,031426137
chr7	137400000	137650000	0,012857267	1	-0,008532334
chr7	137450000	137700000	0,02206208	1	-0,007985362
chr7	137500000	137750000	0,007109666	1	-0,008107754
chr7	137550000	137800000	0,000490962	1	-0,008475148
chr7	137600000	137850000	0,000272919	1	-0,008557226
chr7	137650000	137900000	0,000920979	1	-0,008906434
chr7	137700000	137950000	0,027939314	1	-0,00949292
chr7	137900000	138150000	0,030006744	1	-0,010695558
chr7	137950000	138200000	0,006052024	1	-0,010695558
chr7	140500000	140750000	0,00669245	1	-0,021138667
chr7	140550000	140800000	2,65E-05	1	-0,021237383

chr7	140600000	140850000	0,00010898	1	-0,021753331
chr7	140650000	140900000	0,005125362	1	-0,022684158
chr7	140700000	140950000	0,04037608	1	-0,023810587
chr7	140850000	141100000	0,014246163	1	-0,024394833
chr7	140900000	141150000	0,014575531	1	-0,024394833
chr7	141650000	141900000	0,049981904	1	-0,056606461
chr7	141700000	141950000	0,010276531	1	-0,056606461
chr7	144800000	145050000	0,028399905	1	-0,029842922
chr7	144850000	145100000	0,010750615	1	-0,030213724
chr7	144900000	145150000	0,003196153	1	-0,030495568
chr7	144950000	145200000	0,001031823	1	-0,030831662
chr7	145000000	145250000	0,000887846	1	-0,031360906
chr7	145050000	145300000	0,002557992	1	-0,032164988
chr7	145100000	145350000	0,009594999	1	-0,032583867

chr7	145150000	145400000	0,027414087	1	-0,032583867
chr7	145400000	145650000	0,025044445	1	-0,035928887
chr7	145450000	145700000	0,006175111	1	-0,037902376
chr7	145500000	145750000	0,000527057	1	-0,039411218
chr7	145550000	145800000	0,002441708	1	-0,037802734
chr7	145600000	145850000	0,046527204	1	-0,035386055
chr7	146050000	146300000	0,045778791	1	-0,018552848
chr7	146100000	146350000	0,0022361	1	-0,019194941
chr7	146150000	146400000	0,013356791	1	-0,019957949
chr7	146200000	146450000	0,043780979	1	-0,020660151
chr7	146250000	146500000	0,047524708	1	-0,02114976
chr7	146300000	146550000	0,021455948	1	-0,02131813
chr7	146350000	146600000	0,003220091	1	-0,021118414
chr7	146400000	146650000	0,000413607	1	-0,020567691
chr7	146450000	146700000	0,005919445	1	-0,019841799

chr7	146500000	146750000	0,041711389	1	-0,019204217
chr7	146700000	146950000	0,040944928	1	-0,018391109
chr7	146750000	147000000	0,004381989	1	-0,017867395
chr7	146800000	147050000	0,00041388	1	-0,017164328
chr7	146850000	147100000	0,003530248	1	-0,016753018
chr7	146900000	147150000	0,01300377	1	-0,016680941
chr7	146950000	147200000	0,020189558	1	-0,016898461
chr7	147000000	147250000	0,020539583	1	-0,017302571
chr7	147050000	147300000	0,017100199	1	-0,017791055
chr7	147100000	147350000	0,012499921	1	-0,018294699
chr7	147150000	147400000	0,007824242	1	-0,018788689
chr7	147200000	147450000	0,004276391	1	-0,019266337
chr7	147250000	147500000	0,002732882	1	-0,019714821
chr7	147300000	147550000	0,004197511	1	-0,020106668

chr7	147350000	147600000	0,01786318	1	-0,020425091
chr7	147750000	148000000	0,024092984	1	-0,031082686
chr7	147800000	148050000	0,014838178	1	-0,033201775
chr7	149300000	149550000	0,003198204	1	-0,016452463
chr7	149350000	149600000	0,028626559	1	-0,01559823
chr7	152750000	153000000	0,022210786	1	-0,036021208
chr7	153750000	154000000	0,010653683	1	-0,02261727
chr7	153800000	154050000	0,008573437	1	-0,022365081
chr7	153850000	154100000	0,028950497	1	-0,022365081
chr7	153900000	154150000	0,016639219	1	-0,022365081
chr7	153950000	154200000	0,004165037	1	-0,022365081
chr7	154350000	154600000	0,029078276	1	-0,033934257
chr7	155100000	155350000	0,041734247	1	-0,016705142
chr7	155150000	155400000	0,046399695	1	-0,017319529
chr7	155850000	156100000	0,042084676	1	-0,039609138

chr7	155900000	156150000	0,00737763	1	- 0,039609138
chr7	157650000	157900000	0,007555267	1	- 0,046074398
chr7	157700000	157950000	0,026211386	1	- 0,046074398
chr7	158850000	159100000	0,005515493	1	- 0,023764744
chr7	158900000	159138663	0,004642434	1	- 0,023234748
chrX	2750000	3000000	0,046390618	1	- 0,015687026
chrX	2800000	3050000	0,008582329	1	- 0,017127599
chrX	3100000	3350000	0,015615758	1	- 0,013044095
chrX	3150000	3400000	0,047369399	1	- 0,013044095
chrX	3900000	4150000	0,023496657	1	- 0,037906126
chrX	3950000	4200000	0,003448332	1	- 0,036208743
chrX	4000000	4250000	0,049326982	1	- 0,033753154
chrX	4350000	4600000	0,03208234	1	- 0,023562121
chrX	4400000	4650000	0,007296762	1	-0,02483052
chrX	4700000	4950000	0,04532841	1	-

					0,032225193
chrX	4750000	5000000	0,00297224	1	- 0,031532037
chrX	4800000	5050000	0,001810943	1	- 0,030500155
chrX	4850000	5100000	0,014343974	1	- 0,029464344
chrX	4900000	5150000	0,031488675	1	- 0,028681585
chrX	4950000	5200000	0,025774766	1	- 0,028529142
chrX	5000000	5250000	0,00879024	1	- 0,028529142
chrX	5050000	5300000	0,001168349	1	- 0,028529142
chrX	5100000	5350000	5,47E-05	1	- 0,028529142
chrX	5150000	5400000	4,26E-06	1	- 0,028250273
chrX	5200000	5450000	1,59E-05	1	- 0,028104395
chrX	5250000	5500000	4,87E-05	1	- 0,028082255
chrX	5300000	5550000	3,51E-05	1	- 0,028025173
chrX	5350000	5600000	1,02E-05	1	- 0,027863976
chrX	5400000	5650000	0,000175652	1	-0,02764094

chrX	5450000	5700000	0,001914824	1	-0,027480345
chrX	5500000	5750000	0,006372151	1	-0,027513383
chrX	5550000	5800000	0,01009846	1	-0,027808461
chrX	5600000	5850000	0,010045263	1	-0,028341221
chrX	5650000	5900000	0,007959869	1	-0,029013643
chrX	5700000	5950000	0,006333731	1	-0,029714401
chrX	5750000	6000000	0,005809736	1	-0,030373516
chrX	5800000	6050000	0,005966006	1	-0,030981876
chrX	5850000	6100000	0,00607813	1	-0,031574398
chrX	5900000	6150000	0,005652853	1	-0,03219053
chrX	5950000	6200000	0,004782588	1	-0,032838785
chrX	6000000	6250000	0,003865355	1	-0,035829302
chrX	6050000	6300000	0,003113321	1	-0,036490248
chrX	6100000	6350000	0,002401785	1	-0,0369834
chrX	6150000	6400000	0,00151369	1	-0,037336387

chrX	6200000	6450000	0,000573336	1	-0,037449192
chrX	6250000	6500000	7,52E-05	1	-0,03721087
chrX	6300000	6550000	6,96E-05	1	-0,036767212
chrX	6350000	6600000	0,002540736	1	-0,036767212
chrX	6400000	6650000	0,028920777	1	-0,036767212
chrX	7650000	7900000	0,031252462	1	-0,02623536
chrX	8900000	9150000	0,013350929	1	-0,023452366
chrX	8950000	9200000	0,000492017	1	-0,02461071
chrX	9000000	9250000	0,00278674	1	-0,025354371
chrX	9050000	9300000	0,015371845	1	-0,025546624
chrX	9100000	9350000	0,031121308	1	-0,022713253
chrX	9150000	9400000	0,031348126	1	-0,021994192
chrX	9200000	9450000	0,014865405	1	-0,021845149
chrX	9250000	9500000	0,00216622	1	-0,02215494
chrX	9300000	9550000	0,001423091	1	-0,023049626
chrX	9350000	9600000	0,042186494	1	-0,024654956

chrX	9800000	10050000	0,021403008	1	-0,042507443
chrX	11500000	11750000	0,012658708	1	-0,038964008
chrX	11550000	11800000	0,001526476	1	-0,037597953
chrX	11600000	11850000	0,022102211	1	-0,035209812
chrX	12350000	12600000	0,027142549	1	-0,037845689
chrX	12400000	12650000	0,022498122	1	-0,040668714
chrX	14200000	14450000	0,00721624	1	-0,046023964
chrX	14250000	14500000	0,000165759	1	-0,045441155
chrX	14300000	14550000	0,000155564	1	-0,044975504
chrX	14350000	14600000	0,000265934	1	-0,044910593
chrX	14400000	14650000	6,51E-05	1	-0,044910593
chrX	14450000	14700000	0,000286289	1	-0,044910593
chrX	14500000	14750000	0,005341138	1	-0,044910593
chrX	14550000	14800000	0,027606803	1	-0,044910593

chrX	14750000	15000000	0,015315818	1	- 0,050197123
chrX	14800000	15050000	0,00146892	1	- 0,052270628
chrX	14850000	15100000	0,006052092	1	- 0,054043849
chrX	16100000	16350000	0,03642203	1	-0,02879735
chrX	17250000	17500000	0,013143791	1	- 0,036542597
chrX	17300000	17550000	0,006195121	1	- 0,034726196
chrX	17650000	17900000	0,006094754	1	- 0,027827776
chrX	17700000	17950000	0,000497739	1	- 0,026361649
chrX	17750000	18000000	0,000581683	1	- 0,025747006
chrX	17800000	18050000	0,001884914	1	- 0,025894356
chrX	21350000	21600000	0,007938798	1	- 0,040592473
chrX	22350000	22600000	0,012650421	1	- 0,041726204
chrX	22400000	22650000	0,000731561	1	- 0,040606357
chrX	22450000	22700000	0,005868854	1	- 0,039130871
chrX	22500000	22750000	0,030769848	1	-

					0,037596299
chrX	22650000	22900000	0,019134088	1	-0,035543144
chrX	22700000	22950000	0,001964841	1	-0,035543144
chrX	22750000	23000000	0,005997199	1	-0,035543144
chrX	23050000	23300000	0,015393093	1	-0,04376058
chrX	23100000	23350000	0,022343705	1	-0,046401721
chrX	24650000	24900000	0,006644302	1	-0,026128101
chrX	24700000	24950000	0,000789299	1	-0,025410292
chrX	24750000	25000000	0,005478258	1	-0,024734435
chrX	24800000	25050000	0,009929578	1	-0,024447165
chrX	24850000	25100000	0,003323809	1	-0,024824298
chrX	24900000	25150000	0,00236166	1	-0,025976887
chrX	25200000	25450000	0,022062875	1	-0,032344849
chrX	25250000	25500000	0,020991106	1	-0,029985051
chrX	26200000	26450000	0,019705239	1	-0,024693324

chrX	27250000	27500000	0,040652781	1	- 0,037736745
chrX	27300000	27550000	0,021239646	1	- 0,036261926
chrX	27650000	27900000	0,002889457	1	- 0,025925236
chrX	27700000	27950000	0,001946268	1	- 0,025925236
chrX	27750000	28000000	0,002801573	1	- 0,025925236
chrX	27800000	28050000	0,001869105	1	- 0,026012738
chrX	28700000	28950000	0,013417841	1	- 0,029119802
chrX	28750000	29000000	0,0004809	1	- 0,028427026
chrX	28800000	29050000	0,005021841	1	- 0,027385348
chrX	28850000	29100000	0,040266937	1	- 0,025944028
chrX	30200000	30450000	0,03209316	1	-0,02688993
chrX	30250000	30500000	0,019089129	1	- 0,028918399
chrX	31350000	31600000	0,042764215	1	- 0,068486734
chrX	31400000	31650000	0,001653719	1	- 0,068486734
chrX	31450000	31700000	0,003628638	1	-

					0,068486734
chrX	31500000	31750000	0,03522577	1	-0,068486734
chrX	32850000	33100000	0,027435499	1	-0,011465567
chrX	32900000	33150000	0,032466977	1	-0,010771542
chrX	33250000	33500000	0,04919833	1	-0,008086286
chrX	33300000	33550000	0,035609915	1	-0,007944755
chrX	33350000	33600000	0,009623543	1	-0,008064975
chrX	33400000	33650000	0,005777984	1	-0,008563621
chrX	33900000	34150000	0,030521049	1	-0,016217387
chrX	33950000	34200000	0,046700763	1	-0,016888668
chrX	34350000	34600000	0,007745094	1	-0,026275118
chrX	34400000	34650000	0,026122968	1	-0,027749249
chrX	35150000	35400000	0,042793326	1	-0,034803515
chrX	35200000	35450000	0,003252804	1	-0,037436443
chrX	35250000	35500000	0,037089177	1	-

					0,039080984
chrX	35700000	35950000	0,028039181	1	-0,019743086
chrX	36550000	36800000	0,049460702	1	-0,028698502
chrX	36600000	36850000	0,004350543	1	-0,030411983
chrX	36650000	36900000	0,004883958	1	-0,031717776
chrX	38300000	38550000	0,01148374	1	-0,011687896
chrX	38350000	38600000	0,020115515	1	-0,011687896
chrX	38400000	38650000	0,014375452	1	-0,011687896
chrX	38450000	38700000	0,017326084	1	-0,011310053
chrX	38900000	39150000	0,033407024	1	-0,022296719
chrX	38950000	39200000	0,006067625	1	-0,022296719
chrX	39000000	39250000	0,004061678	1	-0,023064958
chrX	39050000	39300000	0,009774637	1	-0,0234897
chrX	39100000	39350000	0,021880282	1	-0,023791299
chrX	39150000	39400000	0,024440971	1	-0,024190087

chrX	39200000	39450000	0,009138468	1	-0,024813329
chrX	39250000	39500000	0,001806701	1	-0,025634164
chrX	39300000	39550000	0,035651253	1	-0,026457688
chrX	39700000	39950000	0,04397175	1	-0,013674849
chrX	40000000	40250000	0,043606587	1	-0,018100928
chrX	40050000	40300000	0,049054174	1	-0,016354223
chrX	41700000	41950000	0,006200551	1	-0,036205125
chrX	41750000	42000000	0,00047223	1	-0,035250646
chrX	41800000	42050000	0,007985114	1	-0,033568452
chrX	43300000	43550000	0,004227044	1	-0,029303841
chrX	43350000	43600000	0,01661271	1	-0,030772006
chrX	43550000	43800000	0,047594001	1	-0,028365447
chrX	43600000	43850000	0,003478927	1	-0,026712705
chrX	43650000	43900000	0,008408865	1	-0,02549174
chrX	43700000	43950000	0,032067456	1	-

					0,025001796
chrX	43750000	44000000	0,026635488	1	-0,025292082
chrX	43800000	44050000	0,004967672	1	-0,026152091
chrX	43850000	44100000	0,002280771	1	-0,02719165
chrX	43900000	44150000	0,03535787	1	-0,027980496
chrX	44200000	44450000	0,009583332	1	-0,020673456
chrX	44250000	44500000	0,000396309	1	-0,020660553
chrX	44300000	44550000	0,002079596	1	-0,020357012
chrX	44350000	44600000	0,011426611	1	-0,020197665
chrX	44400000	44650000	0,022520127	1	-0,020379516
chrX	44450000	44700000	0,022859993	1	-0,020809888
chrX	44500000	44750000	0,012028476	1	-0,021400602
chrX	44550000	44800000	0,002375976	1	-0,022064547
chrX	44600000	44850000	0,000378592	1	-0,022704694
chrX	44650000	44900000	0,008743892	1	-0,023271105

chrX	47750000	48000000	0,022788991	1	-0,016278003
chrX	47800000	48050000	0,001343571	1	-0,016278003
chrX	47850000	48100000	0,002222529	1	-0,016278003
chrX	50000000	50250000	0,030883626	1	-0,031452835
chrX	50050000	50300000	0,001182127	1	-0,030790803
chrX	50100000	50350000	0,002485844	1	-0,029683133
chrX	50150000	50400000	0,030323702	1	-0,028167756
chrX	54250000	54500000	0,029131627	1	-0,027934572
chrX	54600000	54850000	0,015897776	1	-0,019588429
chrX	55000000	55250000	0,024532079	1	-0,028703673
chrX	55050000	55300000	0,001607547	1	-0,028208406
chrX	55100000	55350000	0,000918395	1	-0,027382036
chrX	55150000	55400000	0,009067796	1	-0,026474671
chrX	55200000	55450000	0,023646237	1	-0,025771651

chrX	55250000	55500000	0,019820774	1	-0,025510139
chrX	55300000	55550000	0,004592259	1	-0,025794313
chrX	55350000	55600000	0,000673464	1	-0,026543545
chrX	55400000	55650000	0,005847399	1	-0,027490886
chrX	55450000	55700000	0,021055887	1	-0,028235642
chrX	55500000	55750000	0,016777235	1	-0,028354117
chrX	55550000	55800000	0,003136333	1	-0,027523472
chrX	55600000	55850000	0,015556539	1	-0,025636492
chrX	56500000	56750000	0,007987959	1	-0,047214449
chrX	56550000	56800000	0,000967052	1	-0,049852192
chrX	56600000	56850000	0,007368099	1	-0,05120465
chrX	56650000	56900000	0,016382486	1	-0,051311306
chrX	56700000	56950000	0,009027632	1	-0,050418039
chrX	56750000	57000000	0,001210769	1	-0,048960408
chrX	56800000	57050000	0,004515211	1	-

					0,047496511
chrX	57150000	57400000	0,009459292	1	- 0,058897434
chrX	57200000	57450000	0,000608008	1	- 0,061424726
chrX	57250000	57500000	0,002893017	1	- 0,063232486
chrX	57300000	57550000	0,030992195	1	- 0,064096462
chrX	57550000	57800000	0,00486529	1	- 0,054217835
chrX	57600000	57850000	0,003316428	1	- 0,053859921
chrX	57650000	57900000	0,039526431	1	- 0,056723655
chrX	57900000	58150000	0,013286161	1	- 0,065333349
chrX	57950000	58200000	0,000823168	1	-0,06403501
chrX	58000000	58250000	0,001859708	1	- 0,061849634
chrX	58050000	58300000	0,021694387	1	- 0,059043436
chrX	61750000	62000000	0,048591567	1	- 0,020848251
chrX	61800000	62050000	0,003605226	1	- 0,022257339
chrX	61850000	62100000	0,000118001	1	- 0,023150468

chrX	61900000	62150000	0,00078471	1	-0,023631602
chrX	61950000	62200000	0,007654733	1	-0,023792132
chrX	62000000	62250000	0,028095317	1	-0,023679925
chrX	62150000	62400000	0,03370093	1	-0,021952284
chrX	62200000	62450000	0,005816483	1	-0,0211041
chrX	62250000	62500000	0,002278536	1	-0,020316795
chrX	62700000	62950000	0,009289025	1	-0,033168132
chrX	62750000	63000000	0,000318194	1	-0,0326547
chrX	62800000	63050000	0,000565783	1	-0,031937839
chrX	62850000	63100000	0,004322816	1	-0,031108356
chrX	62900000	63150000	0,012260228	1	-0,03021223
chrX	62950000	63200000	0,021991151	1	-0,029269211
chrX	63000000	63250000	0,031989673	1	-0,028300567
chrX	63050000	63300000	0,041283078	1	-0,02733927
chrX	63100000	63350000	0,046985482	1	-0,026444592
chrX	63150000	63400000	0,044333635	1	-0,025689923

chrX	63200000	63450000	0,0314347	1	-0,02514788
chrX	63250000	63500000	0,014215697	1	-0,024878985
chrX	63300000	63550000	0,003061965	1	-0,024902738
chrX	63350000	63600000	0,000195112	1	-0,025197715
chrX	63400000	63650000	0,000211286	1	-0,025706439
chrX	63450000	63700000	0,002443445	1	-0,026355088
chrX	63500000	63750000	0,008664166	1	-0,027075351
chrX	63550000	63800000	0,015407466	1	-0,027808324
chrX	63600000	63850000	0,017730186	1	-0,028489903
chrX	63650000	63900000	0,014185265	1	-0,029038226
chrX	63700000	63950000	0,007354796	1	-0,029314804
chrX	63750000	64000000	0,001927667	1	-0,029314804
chrX	63800000	64050000	0,000160944	1	-0,029314804
chrX	63850000	64100000	9,37E-05	1	-0,029314804
chrX	63900000	64150000	0,001016506	1	-

					0,029358542
chrX	63950000	64200000	0,002940168	1	- 0,029380343
chrX	64000000	64250000	0,002979288	1	- 0,029100101
chrX	64050000	64300000	0,001251177	1	- 0,028603981
chrX	64100000	64350000	0,000256047	1	- 0,028046806
chrX	64150000	64400000	0,000103991	1	- 0,027589375
chrX	64200000	64450000	0,000907897	1	- 0,027318919
chrX	64250000	64500000	0,013064142	1	- 0,027202667
chrX	65250000	65500000	0,006388812	1	- 0,025378974
chrX	65300000	65550000	0,000680851	1	- 0,025572347
chrX	65350000	65600000	0,000200357	1	- 0,025800805
chrX	65400000	65650000	0,000187498	1	- 0,026029168
chrX	65450000	65700000	0,000187424	1	- 0,026198369
chrX	65500000	65750000	9,58E-05	1	- 0,026245711
chrX	65550000	65800000	1,42E-05	1	-

					0,026115862
chrX	65600000	65850000	2,00E-05	1	-0,025761447
chrX	65650000	65900000	0,000669048	1	-0,025551731
chrX	65700000	65950000	0,006831472	1	-0,025551731
chrX	65750000	66000000	0,034182276	1	-0,025551731
chrX	66100000	66350000	0,015677878	1	-0,019087915
chrX	66150000	66400000	0,008793695	1	-0,018027729
chrX	66950000	67200000	0,00729772	1	-0,045716439
chrX	67000000	67250000	0,009240588	1	-0,048158825
chrX	68250000	68500000	0,033547084	1	-0,013555069
chrX	68900000	69150000	0,015827268	1	-0,037245805
chrX	68950000	69200000	0,033324001	1	-0,038955818
chrX	69450000	69700000	0,017083483	1	-0,015260677
chrX	69650000	69900000	0,008305802	1	-0,017078163
chrX	69700000	69950000	0,002411522	1	-

					0,017078163
chrX	69750000	70000000	0,047528558	1	-0,017078163
chrX	72350000	72600000	0,02820295	1	-0,034905941
chrX	72400000	72650000	0,003167766	1	-0,034905941
chrX	72450000	72700000	0,003544231	1	-0,034905941
chrX	73000000	73250000	0,0107737	1	-0,013410765
chrX	73250000	73500000	0,04851304	1	-0,014690816
chrX	73300000	73550000	0,010052242	1	-0,016787561
chrX	73350000	73600000	0,000691667	1	-0,016598908
chrX	73400000	73650000	0,000105288	1	-0,016344712
chrX	73450000	73700000	0,000324583	1	-0,016255389
chrX	73500000	73750000	0,00021347	1	-0,016640386
chrX	73550000	73800000	0,012169442	1	-0,017801716
chrX	74000000	74250000	0,037625726	1	-0,038191033
chrX	74050000	74300000	0,001393297	1	-

					0,037151105
chrX	74100000	74350000	0,005933282	1	- 0,035778318
chrX	74150000	74400000	0,036640494	1	- 0,034203384
chrX	74700000	74950000	0,013147776	1	- 0,018389717
chrX	74750000	75000000	0,005771971	1	- 0,019275121
chrX	75200000	75450000	0,007851632	1	- 0,027001591
chrX	75250000	75500000	0,000323546	1	- 0,028199221
chrX	75300000	75550000	6,77E-06	1	- 0,028876189
chrX	75350000	75600000	2,31E-05	1	- 0,028727242
chrX	75400000	75650000	4,52E-05	1	- 0,028564118
chrX	75450000	75700000	8,29E-05	1	- 0,028271252
chrX	75500000	75750000	0,000459014	1	- 0,027716474
chrX	75550000	75800000	0,00396372	1	-0,02680116
chrX	75600000	75850000	0,0261928	1	-0,02550389
chrX	75950000	76200000	0,026976727	1	- 0,020493331
chrX	76000000	76250000	0,038408635	1	-

					0,019162759
chrX	76450000	76700000	0,005635944	1	-0,03455074
chrX	76500000	76750000	0,002295358	1	-0,036319329
chrX	76550000	76800000	0,039142798	1	-0,037231061
chrX	77450000	77700000	0,01700247	1	-0,049074819
chrX	77500000	77750000	0,009708154	1	-0,046376291
chrX	77950000	78200000	0,029871387	1	-0,028952287
chrX	78000000	78250000	0,021009652	1	-0,028161525
chrX	78050000	78300000	0,022033379	1	-0,027309284
chrX	78100000	78350000	0,029740782	1	-0,026372505
chrX	78150000	78400000	0,045879244	1	-0,025331113
chrX	78500000	78750000	0,041009759	1	-0,019322592
chrX	78550000	78800000	0,003856217	1	-0,019631891
chrX	78600000	78850000	0,000616244	1	-0,020147442
chrX	78650000	78900000	0,004454779	1	-0,020661128

chrX	78700000	78950000	0,008767726	1	- 0,020824754
chrX	78750000	79000000	0,004289406	1	- 0,020824754
chrX	78800000	79050000	0,000489984	1	- 0,020824754
chrX	78850000	79100000	0,000386904	1	- 0,020824754
chrX	78900000	79150000	0,003260757	1	- 0,020975121
chrX	78950000	79200000	0,006749731	1	- 0,020977907
chrX	79000000	79250000	0,003883553	1	- 0,020677003
chrX	79050000	79300000	0,000523183	1	- 0,020196665
chrX	79100000	79350000	0,000107778	1	- 0,019722314
chrX	79150000	79400000	0,000764728	1	- 0,019422727
chrX	79200000	79450000	0,001646865	1	- 0,019385075
chrX	79250000	79500000	0,001141824	1	- 0,019586907
chrX	79300000	79550000	0,000430833	1	- 0,019916285
chrX	79350000	79600000	0,000383829	1	-0,02023571
chrX	79400000	79650000	0,002778449	1	-

					0,020458546
chrX	79450000	79700000	0,027407629	1	-0,020591827
chrX	79950000	80200000	0,021539095	1	-0,041015323
chrX	80000000	80250000	0,001450713	1	-0,040041336
chrX	80050000	80300000	0,0049809	1	-0,038159063
chrX	80500000	80750000	0,023757348	1	-0,022597436
chrX	80550000	80800000	0,034314795	1	-0,024311906
chrX	80900000	81150000	0,007238613	1	-0,033370934
chrX	80950000	81200000	0,000848874	1	-0,032443594
chrX	81000000	81250000	0,006794375	1	-0,031459111
chrX	81050000	81300000	0,019113514	1	-0,030634192
chrX	81100000	81350000	0,020353218	1	-0,030049551
chrX	81150000	81400000	0,011030992	1	-0,02966437
chrX	81200000	81450000	0,003801792	1	-0,029363067
chrX	81250000	81500000	0,001296508	1	-0,029024924

chrX	81300000	81550000	0,000965203	1	-0,028569827
chrX	81350000	81600000	0,001889392	1	-0,02797411
chrX	81400000	81650000	0,004955042	1	-0,027565168
chrX	81450000	81700000	0,011249114	1	-0,027466685
chrX	81500000	81750000	0,019722564	1	-0,027466685
chrX	81550000	81800000	0,026964616	1	-0,027466685
chrX	81600000	81850000	0,030338093	1	-0,026916007
chrX	81650000	81900000	0,030896419	1	-0,025625867
chrX	81700000	81950000	0,0323606	1	-0,023767903
chrX	81750000	82000000	0,03929799	1	-0,021922242
chrX	82050000	82300000	0,018663509	1	-0,018344671
chrX	82100000	82350000	0,002918681	1	-0,019106567
chrX	82150000	82400000	0,046767116	1	-0,019888515
chrX	82500000	82750000	0,034289118	1	-0,027642844
chrX	82550000	82800000	0,002340301	1	-

					0,028568029
chrX	82600000	82850000	4,48E-05	1	-0,029195782
chrX	82650000	82900000	5,70E-05	1	-0,029526512
chrX	82700000	82950000	0,000130032	1	-0,029612693
chrX	82750000	83000000	0,000125546	1	-0,02942314
chrX	82800000	83050000	0,00031521	1	-0,029153508
chrX	82850000	83100000	0,00310578	1	-0,028936781
chrX	82900000	83150000	0,02967468	1	-0,027933287
chrX	83300000	83550000	0,009201043	1	-0,017530161
chrX	83350000	83600000	0,012558267	1	-0,016643589
chrX	83550000	83800000	0,033623226	1	-0,017842204
chrX	83600000	83850000	0,007984902	1	-0,018643418
chrX	83650000	83900000	0,00073143	1	-0,019328211
chrX	83700000	83950000	9,69E-05	1	-0,019834406
chrX	83750000	84000000	0,001743445	1	-0,020137492

chrX	83800000	84050000	0,012041312	1	-0,02022285
chrX	83850000	84100000	0,032075541	1	-0,020078104
chrX	83900000	84150000	0,041301383	1	-0,019703617
chrX	83950000	84200000	0,024424707	1	-0,019135922
chrX	84000000	84250000	0,004782339	1	-0,018466795
chrX	84050000	84300000	0,000495446	1	-0,017830728
chrX	84100000	84350000	0,004759442	1	-0,017371518
chrX	84150000	84400000	0,028841974	1	-0,017200502
chrX	84350000	84600000	0,041107861	1	-0,021872059
chrX	84400000	84650000	0,021463827	1	-0,022278206
chrX	84450000	84700000	0,008434234	1	-0,022522477
chrX	84500000	84750000	0,002101765	1	-0,022522477
chrX	84550000	84800000	0,000250864	1	-0,022522477
chrX	84600000	84850000	1,04E-05	1	-0,022522477
chrX	84650000	84900000	4,95E-06	1	-

					0,022522477
chrX	84700000	84950000	0,000314367	1	- 0,022605436
chrX	84750000	85000000	0,005281014	1	- 0,022590347
chrX	84800000	85050000	0,027365416	1	- 0,022610249
chrX	84900000	85150000	0,043037376	1	- 0,023372274
chrX	84950000	85200000	0,009995394	1	- 0,024232162
chrX	85000000	85250000	0,003840195	1	- 0,025245325
chrX	86050000	86300000	0,023568729	1	- 0,030670581
chrX	86100000	86350000	0,000820472	1	- 0,029975719
chrX	86150000	86400000	0,002506077	1	- 0,029096796
chrX	86200000	86450000	0,01357158	1	- 0,028202474
chrX	86250000	86500000	0,02630271	1	- 0,027387398
chrX	86300000	86550000	0,028956935	1	- 0,026672824
chrX	86350000	86600000	0,023622921	1	- 0,026033407
chrX	86400000	86650000	0,017351789	1	-

					0,025439148
chrX	86450000	86700000	0,013083452	1	-0,024883226
chrX	86500000	86750000	0,010093198	1	-0,024396429
chrX	86550000	86800000	0,006936859	1	-0,024026208
chrX	86600000	86850000	0,003497161	1	-0,023803072
chrX	86650000	86900000	0,001071705	1	-0,023705189
chrX	86700000	86950000	0,000192561	1	-0,023627201
chrX	86750000	87000000	6,01E-05	1	-0,023402433
chrX	86800000	87050000	0,000470709	1	-0,022849776
chrX	86850000	87100000	0,008037343	1	-0,021842141
chrX	87300000	87550000	0,002403841	1	-0,013786705
chrX	87350000	87600000	0,001596257	1	-0,013119045
chrX	87400000	87650000	0,002212996	1	-0,012973828
chrX	87450000	87700000	0,000420437	1	-0,013518669
chrX	87500000	87750000	0,000177245	1	-

					0,013282862
chrX	87550000	87800000	0,000831249	1	-0,013130728
chrX	87600000	87850000	0,000776121	1	-0,013148359
chrX	87650000	87900000	0,00013219	1	-0,013315957
chrX	87700000	87950000	0,000134366	1	-0,013520363
chrX	87750000	88000000	0,000457444	1	-0,013606726
chrX	87800000	88050000	0,000222766	1	-0,013449862
chrX	87850000	88100000	0,000709495	1	-0,013442894
chrX	87900000	88150000	0,020444935	1	-0,013442894
chrX	88300000	88550000	0,027106419	1	-0,008611143
chrX	88700000	88950000	0,010826062	1	-0,014148371
chrX	88750000	89000000	0,038442796	1	-0,013346772
chrX	88900000	89150000	0,018386906	1	-0,01361499
chrX	89250000	89500000	0,030989642	1	-0,018838858
chrX	89950000	90200000	0,026080513	1	-0,022352852

chrX	90000000	90250000	0,000726464	1	-0,023865444
chrX	90050000	90300000	0,000777872	1	-0,024716778
chrX	90100000	90350000	0,004162495	1	-0,024988552
chrX	90150000	90400000	0,006332254	1	-0,024822577
chrX	90200000	90450000	0,003848529	1	-0,024385878
chrX	90250000	90500000	0,00088684	1	-0,023845782
chrX	90300000	90550000	5,36E-05	1	-0,023341869
chrX	90350000	90600000	2,47E-05	1	-0,022976709
chrX	90400000	90650000	0,000148882	1	-0,022802988
chrX	90450000	90700000	0,000155031	1	-0,022824415
chrX	90500000	90750000	3,63E-05	1	-0,02299961
chrX	90550000	90800000	0,000639242	1	-0,023246188
chrX	90600000	90850000	0,0153396	1	-0,023452074
chrX	92450000	92700000	0,032444377	1	-0,035255407
chrX	92500000	92750000	0,002719184	1	-

					0,033857308
chrX	92550000	92800000	0,027885605	1	-0,031914803
chrX	92800000	93050000	0,021500942	1	-0,027359357
chrX	92850000	93100000	0,001271783	1	-0,028127773
chrX	92900000	93150000	0,003971425	1	-0,028768246
chrX	92950000	93200000	0,005795344	1	-0,028869864
chrX	93000000	93250000	0,001414756	1	-0,028173433
chrX	93050000	93300000	0,00649628	1	-0,026644211
chrX	93600000	93850000	0,03081827	1	-0,011692399
chrX	94050000	94300000	0,031199619	1	-0,023491382
chrX	94100000	94350000	0,007986936	1	-0,023537521
chrX	94500000	94750000	0,004042995	1	-0,017020259
chrX	94550000	94800000	0,001290619	1	-0,016282105
chrX	94600000	94850000	0,021966191	1	-0,015924006
chrX	94900000	95150000	0,009235671	1	-0,01991662

chrX	94950000	95200000	0,022394288	1	-0,02101508
chrX	95650000	95900000	0,002412246	1	-0,027859488
chrX	95700000	95950000	0,007021914	1	-0,029128721
chrX	95750000	96000000	0,010167533	1	-0,029359187
chrX	95800000	96050000	0,002376961	1	-0,0277897
chrX	95850000	96100000	0,004696181	1	-0,02911048
chrX	96100000	96350000	0,016719877	1	-0,033883393
chrX	96150000	96400000	0,001191962	1	-0,033883393
chrX	96200000	96450000	0,009389119	1	-0,033883393
chrX	97050000	97300000	0,016684272	1	-0,017219279
chrX	97100000	97350000	0,003158464	1	-0,016704629
chrX	97150000	97400000	0,010706388	1	-0,016445148
chrX	97200000	97450000	0,005960765	1	-0,016593589
chrX	97250000	97500000	0,001324489	1	-0,017192953
chrX	97300000	97550000	0,019346909	1	-0,01820209
chrX	97650000	97900000	0,014223573	1	-0,024436716

chrX	97700000	97950000	0,000967618	1	-0,023741813
chrX	97750000	98000000	0,007075666	1	-0,022796525
chrX	97800000	98050000	0,044884934	1	-0,021733903
chrX	98700000	98950000	0,014970705	1	-0,023307311
chrX	98750000	99000000	0,015567459	1	-0,024190709
chrX	98800000	99050000	0,035799744	1	-0,024720137
chrX	99000000	99250000	0,034127258	1	-0,027931347
chrX	99050000	99300000	0,006174328	1	-0,029169638
chrX	99100000	99350000	0,000453149	1	-0,030293721
chrX	99150000	99400000	0,002928598	1	-0,031116489
chrX	99200000	99450000	0,024654903	1	-0,031484761
chrX	99350000	99600000	0,03887431	1	-0,029512804
chrX	99400000	99650000	0,006083513	1	-0,028258893
chrX	99450000	99700000	0,000528929	1	-0,027138721

chrX	99500000	99750000	0,003526168	1	-0,026405135
chrX	99550000	99800000	0,011912914	1	-0,026197262
chrX	99600000	99850000	0,010333848	1	-0,026503708
chrX	99650000	99900000	0,002209808	1	-0,027174558
chrX	99700000	99950000	0,001261275	1	-0,027972082
chrX	99750000	100000000	0,037463501	1	-0,028625567
chrX	100700000	100950000	0,029900423	1	-0,02122997
chrX	101050000	101300000	0,014669067	1	-0,016052448
chrX	102100000	102350000	0,045950513	1	-0,016651199
chrX	105450000	105700000	0,006881175	1	-0,038737823
chrX	105500000	105750000	0,000269631	1	-0,040857305
chrX	105550000	105800000	0,001543579	1	-0,041846894
chrX	105600000	105850000	0,004126366	1	-0,041939006
chrX	105650000	105900000	0,003853588	1	-0,041430448
chrX	105700000	105950000	0,00173894	1	-

					0,040632246
chrX	105750000	106000000	0,000472235	1	- 0,039810647
chrX	105800000	106050000	0,000142751	1	- 0,039145469
chrX	105850000	106100000	0,000177272	1	- 0,038701373
chrX	105900000	106150000	0,000771748	1	- 0,038434857
chrX	105950000	106200000	0,003563632	1	- 0,038226995
chrX	106000000	106250000	0,011714529	1	- 0,037934934
chrX	106050000	106300000	0,026745056	1	- 0,037443708
chrX	106100000	106350000	0,044609946	1	- 0,036701336
chrX	106700000	106950000	0,046313218	1	- 0,019933461
chrX	107000000	107250000	0,016329778	1	- 0,027091503
chrX	107050000	107300000	0,0294928	1	- 0,025395969
chrX	107250000	107500000	0,017529475	1	- 0,024798001
chrX	107600000	107850000	0,047038125	1	- 0,037315831
chrX	107650000	107900000	0,002350108	1	-

					0,036194412
chrX	107700000	107950000	0,009600847	1	- 0,034450595
chrX	108200000	108450000	0,005228003	1	- 0,019184817
chrX	108250000	108500000	0,00408753	1	- 0,019184817
chrX	108300000	108550000	0,020446145	1	- 0,019184817
chrX	108350000	108600000	0,026250895	1	- 0,019350087
chrX	108400000	108650000	0,012306145	1	- 0,019898172
chrX	108450000	108700000	0,002068996	1	- 0,020590098
chrX	108500000	108750000	9,23E-05	1	- 0,021213955
chrX	108550000	108800000	0,000176901	1	- 0,021630693
chrX	108600000	108850000	0,002507998	1	- 0,021798022
chrX	108650000	108900000	0,015063715	1	- 0,021742847
chrX	109500000	109750000	0,01201072	1	- 0,008187388
chrX	110000000	110250000	0,018854041	1	- 0,016525773
chrX	110050000	110300000	0,006827822	1	-

					0,015646761
chrX	110350000	110600000	0,047221177	1	-0,012274699
chrX	110400000	110650000	0,031230821	1	-0,012274699
chrX	110750000	111000000	0,04498117	1	-0,01647722
chrX	110800000	111050000	0,007788748	1	-0,017601701
chrX	110850000	111100000	0,000508984	1	-0,018451743
chrX	110900000	111150000	0,001636901	1	-0,017778087
chrX	110950000	111200000	0,0285317	1	-0,016806776
chrX	111350000	111600000	0,038244474	1	-0,011105761
chrX	111400000	111650000	0,030300913	1	-0,010636817
chrX	114050000	114300000	0,009055556	1	-0,029663979
chrX	114100000	114350000	0,016952629	1	-0,031462241
chrX	115300000	115550000	0,043030613	1	-0,015290424
chrX	115350000	115600000	0,02474507	1	-0,016176233
chrX	115500000	115750000	0,020169389	1	-0,016261769

chrX	118050000	118300000	0,032748429	1	-0,026595757
chrX	119350000	119600000	0,042883696	1	-0,03013734
chrX	119400000	119650000	0,006954542	1	-0,029824462
chrX	119700000	119950000	0,009937724	1	-0,024800637
chrX	119950000	120200000	0,034472848	1	-0,029601425
chrX	120000000	120250000	0,003578193	1	-0,028339008
chrX	120050000	120300000	0,040249386	1	-0,026437176
chrX	121900000	122150000	0,008366501	1	-0,029773688
chrX	121950000	122200000	0,001127379	1	-0,031387584
chrX	122000000	122250000	0,013849378	1	-0,032269524
chrX	123450000	123700000	0,033909753	1	-0,039741786
chrX	123500000	123750000	0,002983519	1	-0,038258253
chrX	123550000	123800000	0,026732452	1	-0,035679064
chrX	123950000	124200000	0,040095881	1	-0,020855844
chrX	124000000	124250000	0,025993939	1	-

					0,022060515
chrX	124150000	124400000	0,0352938	1	-0,023288454
chrX	124200000	124450000	0,003389425	1	-0,022459792
chrX	124250000	124500000	0,015643598	1	-0,021284447
chrX	124400000	124650000	0,047476044	1	-0,020066977
chrX	124450000	124700000	0,009678292	1	-0,021306349
chrX	124800000	125050000	0,029070777	1	-0,030960339
chrX	124850000	125100000	0,022157721	1	-0,029050775
chrX	125250000	125500000	0,015531751	1	-0,017797529
chrX	125400000	125650000	0,047468449	1	-0,019111994
chrX	125450000	125700000	0,004660415	1	-0,018704156
chrX	125500000	125750000	0,001266387	1	-0,018185303
chrX	125550000	125800000	0,006234046	1	-0,017820616
chrX	125600000	125850000	0,007443333	1	-0,017695665
chrX	125650000	125900000	0,00207575	1	-

					0,017733483
chrX	125700000	125950000	0,000130767	1	-0,017682418
chrX	125750000	126000000	0,00013597	1	-0,01745799
chrX	125800000	126050000	0,00826658	1	-0,017154774
chrX	126600000	126850000	0,007374433	1	-0,011342061
chrX	126650000	126900000	0,009299679	1	-0,011683719
chrX	126950000	127200000	0,016530304	1	-0,014839594
chrX	127000000	127250000	0,016091459	1	-0,015792898
chrX	128200000	128450000	0,028447643	1	-0,03101917
chrX	129600000	129850000	0,016285901	1	-0,017602527
chrX	130500000	130750000	0,007700231	1	-0,014057707
chrX	130550000	130800000	0,000842583	1	-0,015024593
chrX	130600000	130850000	0,001802806	1	-0,015316818
chrX	130650000	130900000	0,000528061	1	-0,01515717
chrX	130700000	130950000	4,00E-05	1	-0,014835408
chrX	130750000	131000000	2,10E-05	1	-0,014593387

chrX	130800000	131050000	0,000344817	1	-0,014549545
chrX	130850000	131100000	0,032054602	1	-0,014673334
chrX	133000000	133250000	0,008708699	1	-0,030643361
chrX	134350000	134600000	0,026958503	1	-0,029962266
chrX	136200000	136450000	0,00786456	1	-0,036549068
chrX	136250000	136500000	0,006840801	1	-0,034878131
chrX	136750000	137000000	0,018304238	1	-0,022910152
chrX	136800000	137050000	0,002407043	1	-0,023022346
chrX	136850000	137100000	0,000111356	1	-0,023364437
chrX	136900000	137150000	0,000603921	1	-0,023908742
chrX	136950000	137200000	0,005851572	1	-0,024672301
chrX	137000000	137250000	0,02473979	1	-0,025710261
chrX	137400000	137650000	0,009787241	1	-0,033454025
chrX	137450000	137700000	0,007335509	1	-0,03447845
chrX	137950000	138200000	0,040771955	1	-

					0,017793797
chrX	138000000	138250000	0,002286026	1	- 0,016762665
chrX	138050000	138300000	0,007599341	1	- 0,016112101
chrX	138750000	139000000	0,038614997	1	- 0,040726515
chrX	138800000	139050000	0,002997546	1	- 0,043644237
chrX	138850000	139100000	0,029536639	1	- 0,045549725
chrX	141850000	142100000	0,033108208	1	- 0,037774903
chrX	141900000	142150000	0,001713042	1	- 0,040162668
chrX	141950000	142200000	2,17E-05	1	- 0,041721348
chrX	142000000	142250000	1,16E-05	1	-0,04240659
chrX	142050000	142300000	6,89E-06	1	- 0,042240256
chrX	142100000	142350000	3,91E-05	1	- 0,042763281
chrX	142150000	142400000	0,000255481	1	- 0,043223824
chrX	142200000	142450000	0,000344427	1	-0,04340205
chrX	142250000	142500000	9,37E-05	1	- 0,043159627
chrX	142300000	142550000	4,61E-05	1	-

					0,042442056
chrX	142350000	142600000	0,001245909	1	-0,041253332
chrX	142400000	142650000	0,012130199	1	-0,039619443
chrX	143500000	143750000	0,016390955	1	-0,016318924
chrX	143550000	143800000	0,002543772	1	-0,016856809
chrX	143600000	143850000	0,025343461	1	-0,017573262
chrX	144100000	144350000	0,004913902	1	-0,031962745
chrX	144150000	144400000	0,00479398	1	-0,030915612
chrX	145200000	145450000	0,031689406	1	-0,019513164
chrX	145250000	145500000	0,012302927	1	-0,019099111
chrX	145900000	146150000	0,010215194	1	-0,02322863
chrX	145950000	146200000	0,000173619	1	-0,024533294
chrX	146000000	146250000	1,36E-05	1	-0,025208668
chrX	146050000	146300000	1,56E-05	1	-0,025343177
chrX	146100000	146350000	0,000198166	1	-0,025164988

chrX	146150000	146400000	0,001751211	1	- 0,024959576
chrX	146200000	146450000	0,003452541	1	- 0,024956838
chrX	146250000	146500000	0,001795033	1	- 0,025249571
chrX	146300000	146550000	0,000216391	1	- 0,025760142
chrX	146350000	146600000	0,00013367	1	- 0,026289485
chrX	146400000	146650000	0,000889898	1	- 0,026618542
chrX	146450000	146700000	0,001285483	1	- 0,026618634
chrX	146500000	146750000	0,000368283	1	- 0,026313595
chrX	146550000	146800000	7,64E-05	1	- 0,025861692
chrX	146600000	146850000	0,000869474	1	- 0,025479443
chrX	146650000	146900000	0,004195512	1	- 0,025341422
chrX	146700000	146950000	0,006564516	1	- 0,025514803
chrX	146750000	147000000	0,004276391	1	-0,02595231
chrX	146800000	147050000	0,001022457	1	- 0,026535446
chrX	146850000	147100000	8,47E-05	1	-

					0,027123294
chrX	146900000	147150000	0,000611523	1	-0,027587659
chrX	146950000	147200000	0,00799084	1	-0,025675721
chrX	147000000	147250000	0,036295313	1	-0,024550484
chrX	147300000	147550000	0,034726316	1	-0,019770862
chrX	147350000	147600000	0,013028807	1	-0,019478402
chrX	147400000	147650000	0,004603271	1	-0,019234333
chrX	147450000	147700000	0,001878614	1	-0,019234333
chrX	147500000	147750000	0,001007268	1	-0,019234333
chrX	147550000	147800000	0,000680372	1	-0,019234333
chrX	147600000	147850000	0,000518193	1	-0,019234333
chrX	147650000	147900000	0,000442681	1	-0,019013147
chrX	147700000	147950000	0,000443902	1	-0,018809373
chrX	147750000	148000000	0,0004685	1	-0,018621122
chrX	147800000	148050000	0,000349569	1	-

					0,018442117
chrX	147850000	148100000	9,65E-05	1	-0,018256321
chrX	147900000	148150000	3,13E-05	1	-0,018060137
chrX	147950000	148200000	0,000681869	1	-0,017877271
chrX	148000000	148250000	0,004145359	1	-0,017776028
chrX	148050000	148300000	0,005152729	1	-0,017840127
chrX	148100000	148350000	0,001360091	1	-0,018117626
chrX	148150000	148400000	0,011020017	1	-0,018560209
chrX	149350000	149600000	0,010643461	1	-0,013756719
chrX	149400000	149650000	0,046155099	1	-0,014634238
chrX	149650000	149900000	0,032568552	1	-0,01204887
chrX	150000000	150250000	0,038835922	1	-0,017785464
chrX	150050000	150300000	0,007308285	1	-0,016843543
chrX	150300000	150550000	0,036395228	1	-0,014684605
chrX	150700000	150950000	0,015990776	1	-0,022278201

chrX	151450000	151700000	0,013074895	1	-0,02890583
chrX	152200000	152450000	0,02824838	1	-0,056876097
chrX	154300000	154550000	0,039111436	1	-0,033636947
chrX	154350000	154600000	0,006554081	1	-0,031850514
chr8	150000	400000	0,014699213	1	-0,029532787
chr8	200000	450000	0,001520014	1	-0,029443235
chr8	250000	500000	3,62E-05	1	-0,029291006
chr8	300000	550000	1,94E-06	1	-0,029230286
chr8	350000	600000	1,97E-06	1	-0,02934733
chr8	400000	650000	6,34E-06	1	-0,029644211
chr8	450000	700000	0,000114544	1	-0,030038307
chr8	500000	750000	0,00047936	1	-0,030393372
chr8	550000	800000	0,000557309	1	-0,030567746
chr8	600000	850000	0,000175228	1	-0,030482388
chr8	650000	900000	1,93E-05	1	-0,030169116

chr8	700000	950000	8,45E-05	1	-0,029772695
chr8	750000	1000000	0,000349846	1	-0,029513561
chr8	800000	1050000	0,000235095	1	-0,029605755
chr8	850000	1100000	7,93E-05	1	-0,029605755
chr8	900000	1150000	0,001700633	1	-0,029605755
chr8	950000	1200000	0,015611077	1	-0,029605755
chr8	1000000	1250000	0,041532483	1	-0,029605755
chr8	1050000	1300000	0,042963654	1	-0,030169195
chr8	1100000	1350000	0,016494158	1	-0,031165244
chr8	1150000	1400000	0,001787435	1	-0,032391427
chr8	1200000	1450000	0,003377032	1	-0,033531293
chr8	1250000	1500000	0,047741413	1	-0,034243001
chr8	1550000	1800000	0,027139879	1	-0,026386487
chr8	1600000	1850000	0,00141151	1	-0,025002999

chr8	1650000	1900000	0,000642871	1	- 0,024119595
chr8	1700000	1950000	0,008661179	1	- 0,023725469
chr8	1750000	2000000	0,038777317	1	- 0,026863397
chr8	2000000	2250000	0,049640985	1	- 0,032143766
chr8	2050000	2300000	0,00595591	1	- 0,031357526
chr8	2100000	2350000	0,007893594	1	- 0,029529865
chr8	2650000	2900000	0,010420527	1	-0,01046265
chr8	2700000	2950000	0,040406857	1	- 0,009800901
chr8	2850000	3100000	0,010729335	1	- 0,011213029
chr8	2900000	3150000	0,003290939	1	- 0,010696379
chr8	3200000	3450000	0,013070617	1	- 0,009164593
chr8	3250000	3500000	0,04807534	1	- 0,009642785
chr8	3350000	3600000	0,031311242	1	- 0,008991074
chr8	5400000	5650000	0,035287729	1	- 0,015789068
chr8	5450000	5700000	0,006648628	1	-

					0,017194683
chr8	5500000	5750000	0,026002378	1	- 0,017878189
chr8	5550000	5800000	0,017666604	1	- 0,017823933
chr8	5600000	5850000	0,008354287	1	-0,01724064
chr8	5900000	6150000	0,018191136	1	- 0,020823242
chr8	9900000	10150000	0,03660252	1	- 0,039794977
chr8	9950000	10200000	0,005287482	1	- 0,039794977
chr8	10000000	10250000	0,004265158	1	- 0,041366698
chr8	10750000	11000000	0,021504064	1	- 0,037284982
chr8	13600000	13850000	0,012035017	1	- 0,033455789
chr8	15800000	16050000	0,041410012	1	-0,03811512
chr8	15850000	16100000	0,007759672	1	- 0,041190866
chr8	16350000	16600000	0,012803099	1	- 0,020535667
chr8	16600000	16850000	0,006586919	1	- 0,024530466
chr8	16650000	16900000	0,009520725	1	- 0,024530466
chr8	17950000	18200000	0,03149867	1	-

					0,014063423
chr8	18250000	18500000	0,004875558	1	-0,011811825
chr8	18300000	18550000	0,006128526	1	-0,011811825
chr8	18350000	18600000	0,03888494	1	-0,011811825
chr8	18400000	18650000	0,044781569	1	-0,011811825
chr8	18450000	18700000	0,011618518	1	-0,011811825
chr8	19450000	19700000	0,019012207	1	-0,021138556
chr8	21200000	21450000	0,006310058	1	-0,037798818
chr8	25450000	25700000	0,030118282	1	-0,02237737
chr8	26700000	26950000	0,014668049	1	-0,030093946
chr8	28750000	29000000	0,035630876	1	-0,020299781
chr8	31150000	31400000	0,020508185	1	-0,03032094
chr8	31200000	31450000	0,00271742	1	-0,030810221
chr8	31250000	31500000	0,002975822	1	-0,03171637
chr8	31300000	31550000	0,013074895	1	-0,032956529
chr8	31350000	31600000	0,035481434	1	-0,032956529

chr8	31400000	31650000	0,037284045	1	-0,032956529
chr8	31450000	31700000	0,010470237	1	-0,032956529
chr8	31500000	31750000	0,010206011	1	-0,032956529
chr8	32950000	33200000	0,035014955	1	-0,010336888
chr8	33000000	33250000	0,013942181	1	-0,010796729
chr8	33050000	33300000	0,015607076	1	-0,011192364
chr8	33650000	33900000	0,042065096	1	-0,04687125
chr8	33700000	33950000	0,001299302	1	-0,0459012
chr8	33750000	34000000	0,001475413	1	-0,044717931
chr8	33800000	34050000	0,007792372	1	-0,043606166
chr8	33850000	34100000	0,012491859	1	-0,042725499
chr8	33900000	34150000	0,009300818	1	-0,042129215
chr8	33950000	34200000	0,003802767	1	-0,041789078
chr8	34000000	34250000	0,000896601	1	-0,041617654
chr8	34050000	34300000	0,000137139	1	-0,041475633

chr8	34100000	34350000	3,55E-05	1	- 0,041183329
chr8	34150000	34400000	0,000130054	1	- 0,040552803
chr8	34200000	34450000	0,00153327	1	- 0,039431489
chr8	34250000	34500000	0,013160177	1	- 0,037747456
chr8	34700000	34950000	0,020452835	1	- 0,023356413
chr8	34750000	35000000	0,000814222	1	- 0,023915988
chr8	34800000	35050000	0,002433132	1	-0,02438543
chr8	34850000	35100000	0,003716658	1	- 0,024523998
chr8	34900000	35150000	0,000912198	1	- 0,024185038
chr8	34950000	35200000	0,000983136	1	- 0,023333928
chr8	35000000	35250000	0,022515704	1	- 0,022040836
chr8	35350000	35600000	0,012401193	1	- 0,016816281
chr8	36200000	36450000	0,00720741	1	- 0,045787468
chr8	36250000	36500000	0,002004069	1	- 0,048037302
chr8	36300000	36550000	0,03516771	1	-

					0,049407984
chr8	36650000	36900000	0,00681281	1	-0,034886017
chr8	36700000	36950000	0,030178651	1	-0,033038356
chr8	37050000	37300000	0,004851442	1	-0,047672211
chr8	37100000	37350000	0,01050631	1	-0,047672211
chr8	39000000	39250000	0,005742227	1	-0,017584986
chr8	39050000	39300000	0,010874034	1	-0,01707756
chr8	39100000	39350000	0,017197889	1	-0,017011839
chr8	39150000	39400000	0,004207517	1	-0,017466416
chr8	39200000	39450000	0,005048163	1	-0,018207133
chr8	39250000	39500000	0,049144659	1	-0,018207133
chr8	39400000	39650000	0,021375178	1	-0,018306543
chr8	39450000	39700000	0,002355867	1	-0,019259057
chr8	39500000	39750000	0,024239655	1	-0,020022568
chr8	39800000	40050000	0,028216307	1	-0,015429109

chr8	40100000	40350000	0,038615256	1	-0,018224088
chr8	41100000	41350000	0,00535321	1	-0,014277631
chr8	41150000	41400000	0,024751158	1	-0,015034497
chr8	41400000	41650000	0,043548992	1	-0,0127204
chr8	41450000	41700000	0,001432476	1	-0,011879734
chr8	41500000	41750000	0,001251835	1	-0,011384528
chr8	43300000	43550000	0,007372681	1	-0,043470352
chr8	43350000	43600000	0,002035944	1	-0,04170456
chr8	43400000	43650000	0,044476881	1	-0,038917486
chr8	46850000	47100000	0,006278574	1	-0,038345107
chr8	46900000	47150000	0,000342935	1	-0,040512834
chr8	46950000	47200000	0,002868118	1	-0,041424049
chr8	47000000	47250000	0,010102018	1	-0,041413779
chr8	47050000	47300000	0,019250421	1	-0,040845768
chr8	47100000	47350000	0,028134176	1	-0,039992468

chr8	47150000	47400000	0,033050645	1	-0,038971368
chr8	47200000	47450000	0,028205395	1	-0,037566931
chr8	47250000	47500000	0,01411701	1	-0,03665136
chr8	47300000	47550000	0,002831661	1	-0,035588899
chr8	47350000	47600000	0,00020004	1	-0,034551499
chr8	47400000	47650000	0,000749854	1	-0,033796823
chr8	47450000	47700000	0,003587963	1	-0,03347161
chr8	47500000	47750000	0,003511021	1	-0,033639999
chr8	47550000	47800000	0,000724765	1	-0,034229178
chr8	47600000	47850000	0,001889952	1	-0,035021726
chr8	47650000	47900000	0,042229219	1	-0,035679902
chr8	48050000	48300000	0,003230199	1	-0,02236193
chr8	48100000	48350000	0,008039003	1	-0,021313248
chr8	48150000	48400000	0,033203768	1	-0,020960695
chr8	48200000	48450000	0,032310229	1	-0,021215615
chr8	48250000	48500000	0,007737288	1	-

					0,021902356
chr8	48300000	48550000	0,003137844	1	- 0,022768116
chr8	49000000	49250000	0,022140392	1	- 0,014428457
chr8	50450000	50700000	0,026478746	1	- 0,008864305
chr8	50900000	51150000	0,01657434	1	- 0,016772679
chr8	51250000	51500000	0,010703425	1	- 0,011975733
chr8	51300000	51550000	0,021779732	1	- 0,011208385
chr8	51350000	51600000	0,03294804	1	- 0,011008553
chr8	51400000	51650000	0,008310822	1	- 0,011288199
chr8	51450000	51700000	0,027479827	1	- 0,011801705
chr8	53050000	53300000	0,035478959	1	- 0,045688617
chr8	54100000	54350000	0,028976995	1	- 0,036876764
chr8	55750000	56000000	0,016748959	1	- 0,024443381
chr8	55800000	56050000	0,006530864	1	- 0,025326912
chr8	55850000	56100000	0,038905067	1	-

					0,025326912
chr8	56150000	56400000	0,02052553	1	-0,03162976
chr8	59000000	59250000	0,041660659	1	-0,023039282
chr8	59050000	59300000	0,004662373	1	-0,024971002
chr8	59100000	59350000	0,047696272	1	-0,026046075
chr8	61000000	61250000	0,016483999	1	-0,028091631
chr8	61500000	61750000	0,004532634	1	-0,015796034
chr8	61550000	61800000	0,027935453	1	-0,016508935
chr8	62150000	62400000	0,020497197	1	-0,029338771
chr8	62200000	62450000	0,009158856	1	-0,031308021
chr8	62550000	62800000	0,010946307	1	-0,021677165
chr8	62600000	62850000	0,003273103	1	-0,022352723
chr8	62650000	62900000	0,013231681	1	-0,022841579
chr8	62700000	62950000	0,012302701	1	-0,023075025
chr8	62750000	63000000	0,003700446	1	-0,023184022

chr8	62800000	63050000	0,000661409	1	-0,023422956
chr8	62850000	63100000	0,001012461	1	-0,024044141
chr8	62900000	63150000	0,011506852	1	-0,025190602
chr8	63200000	63450000	0,042312609	1	-0,032309987
chr8	63250000	63500000	0,003938756	1	-0,030966289
chr8	63300000	63550000	0,041117698	1	-0,028684983
chr8	66000000	66250000	0,012934369	1	-0,025640927
chr8	66700000	66950000	0,031991066	1	-0,017030323
chr8	68100000	68350000	0,047025937	1	-0,009614879
chr8	68150000	68400000	0,046656375	1	-0,009956422
chr8	69750000	70000000	0,048523222	1	-0,013540604
chr8	73550000	73800000	0,005602678	1	-0,018611908
chr8	73600000	73850000	0,002819281	1	-0,019499236
chr8	75400000	75650000	0,031346107	1	-0,051159031

chr8	75450000	75700000	0,001791376	1	-0,053345736
chr8	75500000	75750000	5,24E-05	1	-0,055234312
chr8	75550000	75800000	0,000535655	1	-0,054112117
chr8	75600000	75850000	0,00535321	1	-0,052369411
chr8	75650000	75900000	0,024558399	1	-0,050168982
chr8	76300000	76550000	0,024617456	1	-0,023165151
chr8	76350000	76600000	0,010091253	1	-0,02444666
chr8	76600000	76850000	0,009497825	1	-0,027044634
chr8	78300000	78550000	0,032030548	1	-0,017290125
chr8	79500000	79750000	0,029810964	1	-0,039201211
chr8	79550000	79800000	0,003395019	1	-0,038942873
chr8	79600000	79850000	0,000178854	1	-0,038258465
chr8	79650000	79900000	0,000938426	1	-0,037357002
chr8	79700000	79950000	0,005125362	1	-0,036474734
chr8	79750000	80000000	0,00873582	1	-

					0,035807357
chr8	79800000	80050000	0,00615919	1	-0,035462581
chr8	79850000	80100000	0,001730687	1	-0,035436928
chr8	79900000	80150000	0,000138443	1	-0,035436928
chr8	79950000	80200000	4,23E-06	1	-0,035436928
chr8	80000000	80250000	2,79E-06	1	-0,035436928
chr8	80050000	80300000	1,94E-05	1	-0,035436928
chr8	80100000	80350000	0,001690824	1	-0,035616655
chr8	80150000	80400000	0,03043937	1	-0,035799293
chr8	82350000	82600000	0,009078992	1	-0,01833744
chr8	82400000	82650000	0,036156985	1	-0,019445579
chr8	82500000	82750000	0,013486769	1	-0,018166719
chr8	82900000	83150000	0,030028627	1	-0,025885076
chr8	83500000	83750000	0,014017003	1	-0,010303733
chr8	83750000	84000000	0,045129552	1	-0,011076487

chr8	83800000	84050000	0,003185982	1	-0,011770495
chr8	83850000	84100000	0,000361906	1	-0,012278685
chr8	83900000	84150000	0,003152222	1	-0,012542129
chr8	83950000	84200000	0,0078208	1	-0,012438698
chr8	84000000	84250000	0,004232088	1	-0,012019167
chr8	84050000	84300000	0,001187492	1	-0,011984439
chr8	84100000	84350000	0,031865092	1	-0,012509959
chr8	84450000	84700000	0,047716509	1	-0,018202472
chr8	84500000	84750000	0,003944057	1	-0,01877711
chr8	84550000	84800000	9,18E-05	1	-0,019176756
chr8	84600000	84850000	1,23E-05	1	-0,019458742
chr8	84650000	84900000	0,000373654	1	-0,01958577
chr8	84700000	84950000	0,003960879	1	-0,019574651
chr8	84750000	85000000	0,019944463	1	-0,018802894
chr8	84800000	85050000	0,048591314	1	-0,018005585

chr8	84850000	85100000	0,048713916	1	-0,017644637
chr8	84900000	85150000	0,013272615	1	-0,017380899
chr8	84950000	85200000	0,009581375	1	-0,017172001
chr8	85350000	85600000	0,032603268	1	-0,026813213
chr8	85400000	85650000	0,005180448	1	-0,028402372
chr8	85450000	85700000	0,003056783	1	-0,029396657
chr8	85500000	85750000	0,009423203	1	-0,029926905
chr8	85550000	85800000	0,030666048	1	-0,03026192
chr8	85650000	85900000	0,049614951	1	-0,031481852
chr8	85700000	85950000	0,017788894	1	-0,032625534
chr8	85750000	86000000	0,001812485	1	-0,033967847
chr8	85800000	86050000	0,000949613	1	-0,035182523
chr8	85850000	86100000	0,007794471	1	-0,035917458
chr8	85900000	86150000	0,015822005	1	-0,035952363
chr8	85950000	86200000	0,008419424	1	-

					0,035312226
chr8	86000000	86250000	0,001003725	1	-0,033114096
chr8	86050000	86300000	9,84E-05	1	-0,033497078
chr8	86100000	86350000	8,60E-05	1	-0,033168983
chr8	86150000	86400000	0,002207111	1	-0,031480485
chr8	89000000	89250000	0,017253207	1	-0,034882539
chr8	89050000	89300000	0,004535916	1	-0,036861079
chr8	90100000	90350000	0,004141384	1	-0,009277794
chr8	90150000	90400000	0,001286935	1	-0,009094932
chr8	90200000	90450000	0,003069413	1	-0,009094932
chr8	90250000	90500000	0,00109225	1	-0,009094932
chr8	90300000	90550000	0,000125232	1	-0,009094932
chr8	90350000	90600000	0,000208608	1	-0,009094932
chr8	90400000	90650000	0,000167623	1	-0,009033839
chr8	90450000	90700000	0,00256086	1	-0,00911101

chr8	91200000	91450000	0,02808585	1	-0,031177488
chr8	91750000	92000000	0,005439643	1	-0,007950121
chr8	91800000	92050000	0,000214219	1	-0,007497389
chr8	91850000	92100000	0,000359801	1	-0,007222679
chr8	91900000	92150000	0,001336245	1	-0,007597954
chr8	92400000	92650000	0,029662573	1	-0,022709225
chr8	93350000	93600000	0,002934419	1	-0,005433555
chr8	93400000	93650000	0,021442078	1	-0,005609264
chr8	96650000	96900000	0,017972629	1	-0,052549161
chr8	96700000	96950000	0,006892072	1	-0,052549161
chr8	96750000	97000000	0,006277401	1	-0,052549161
chr8	96800000	97050000	0,007793567	1	-0,052549161
chr8	96850000	97100000	0,007878151	1	-0,052549161
chr8	96900000	97150000	0,005014822	1	-0,053638042

chr8	96950000	97200000	0,001608531	1	-0,054865269
chr8	97000000	97250000	0,000170678	1	-0,056081869
chr8	97050000	97300000	3,83E-05	1	-0,057088636
chr8	97100000	97350000	0,000864604	1	-0,05771834
chr8	97150000	97400000	0,007878104	1	-0,053312137
chr8	97200000	97450000	0,037897607	1	-0,050721089
chr8	98100000	98350000	0,020659798	1	-0,031257089
chr8	100500000	100750000	0,020849887	1	-0,036969119
chr8	103100000	103350000	0,034614857	1	-0,018773268
chr8	103150000	103400000	0,038460298	1	-0,020228421
chr8	104650000	104900000	0,029717995	1	-0,018730996
chr8	105400000	105650000	0,028186314	1	-0,022410644
chr8	106750000	107000000	0,042509113	1	-0,026608898
chr8	106800000	107050000	0,038673861	1	-0,02792257
chr8	107150000	107400000	0,006544598	1	-0,040705241

chr8	108950000	109200000	0,0354579	1	-<br>0,032202725
chr8	109000000	109250000	0,006530864	1	-<br>0,033973524
chr8	109050000	109300000	0,001569206	1	-<br>0,035199847
chr8	109100000	109350000	0,001156269	1	-0,03597024
chr8	109150000	109400000	0,002376978	1	-<br>0,036443166
chr8	109200000	109450000	0,005765077	1	-<br>0,038487173
chr8	109250000	109500000	0,011322976	1	-<br>0,040833346
chr8	109300000	109550000	0,017280713	1	-<br>0,041995863
chr8	109350000	109600000	0,021258395	1	-<br>0,042101145
chr8	109400000	109650000	0,021111103	1	-<br>0,042101145
chr8	109450000	109700000	0,015282617	1	-<br>0,042101145
chr8	109500000	109750000	0,006235608	1	-<br>0,042101145
chr8	109550000	109800000	0,000872069	1	-<br>0,043176351
chr8	109600000	109850000	0,000493928	1	-<br>0,044263591
chr8	109650000	109900000	0,011108849	1	-

					0,045056526
chr8	111000000	111250000	0,007134829	1	- 0,032019257
chr8	111050000	111300000	0,004418724	1	- 0,030617829
chr8	111650000	111900000	0,004696252	1	- 0,014966501
chr8	111700000	111950000	0,008061265	1	- 0,015484467
chr8	111750000	112000000	0,021553465	1	- 0,015770981
chr8	111800000	112050000	0,010437733	1	- 0,015670532
chr8	111850000	112100000	0,001568968	1	- 0,015166566
chr8	111900000	112150000	0,011896862	1	- 0,014368626
chr8	112200000	112450000	0,004154756	1	- 0,012317744
chr8	112250000	112500000	0,015486538	1	- 0,013001846
chr8	112550000	112800000	0,00724737	1	- 0,015528714
chr8	112600000	112850000	0,009816001	1	- 0,015169119
chr8	113250000	113500000	0,04315365	1	- 0,011707035
chr8	113300000	113550000	0,005129863	1	-

					0,011745322
chr8	113750000	114000000	0,003765612	1	-0,006823329
chr8	113800000	114050000	0,013546561	1	-0,006491653
chr8	113850000	114100000	0,032405874	1	-0,006475837
chr8	113900000	114150000	0,02699388	1	-0,006634367
chr8	113950000	114200000	0,022438989	1	-0,006808394
chr8	114500000	114750000	0,017914544	1	-0,02085685
chr8	114550000	114800000	0,000305265	1	-0,022274499
chr8	114600000	114850000	0,000269053	1	-0,022967812
chr8	114650000	114900000	0,000302787	1	-0,023067149
chr8	114700000	114950000	6,88E-05	1	-0,022826279
chr8	114750000	115000000	0,001233375	1	-0,022506573
chr8	114800000	115050000	0,021401952	1	-0,022304715
chr8	115200000	115450000	0,030554824	1	-0,03324385
chr8	115250000	115500000	0,001863294	1	-0,035188317
chr8	115300000	115550000	0,000310109	1	-

					0,036532743
chr8	115350000	115600000	0,00506916	1	-0,037200697
chr8	115400000	115650000	0,033293488	1	-0,037253619
chr8	115800000	116050000	0,009120203	1	-0,02248385
chr8	115850000	116100000	0,013245281	1	-0,02248385
chr8	116000000	116250000	0,019701792	1	-0,023373992
chr8	116050000	116300000	0,004424696	1	-0,024520288
chr8	116500000	116750000	0,018924948	1	-0,013837547
chr8	117050000	117300000	0,036925549	1	-0,026356012
chr8	117100000	117350000	0,003355527	1	-0,028150911
chr8	117150000	117400000	0,035936816	1	-0,029381892
chr8	117600000	117850000	0,026876915	1	-0,017532299
chr8	117650000	117900000	0,002346103	1	-0,016620886
chr8	117700000	117950000	0,0252331	1	-0,016000057
chr8	117950000	118200000	0,03206511	1	-0,018675671
chr8	121250000	121500000	0,014929992	1	-0,03973453

chr8	121300000	121550000	0,029397026	1	-0,037286251
chr8	122300000	122550000	0,010070507	1	-0,042394014
chr8	122350000	122600000	0,001381108	1	-0,044153648
chr8	122400000	122650000	0,000211352	1	-0,045289991
chr8	122450000	122700000	7,92E-05	1	-0,04594873
chr8	122500000	122750000	7,43E-05	1	-0,046320045
chr8	122550000	122800000	6,99E-05	1	-0,046585387
chr8	122600000	122850000	3,49E-05	1	-0,046867983
chr8	122650000	122900000	5,96E-06	1	-0,04775798
chr8	122700000	122950000	2,86E-07	1	-0,047628208
chr8	122750000	123000000	2,67E-07	1	-0,047528979
chr8	122800000	123050000	6,40E-07	1	-0,047477872
chr8	122850000	123100000	6,53E-07	1	-0,047368885
chr8	122900000	123150000	6,42E-06	1	-0,046954659
chr8	122950000	123200000	0,000292303	1	-0,04590627
chr8	123000000	123250000	0,006845217	1	-

					0,043910591
chr8	125350000	125600000	0,037603628	1	- 0,013728232
chr8	125400000	125650000	0,007608067	1	- 0,014545475
chr8	125450000	125700000	0,000707642	1	- 0,014333047
chr8	125500000	125750000	0,001139457	1	- 0,013814169
chr8	125550000	125800000	0,026557287	1	- 0,013021583
chr8	127100000	127350000	0,02582743	1	- 0,025255196
chr8	131250000	131500000	0,002185749	1	- 0,007555335
chr8	131300000	131550000	0,006321384	1	- 0,008137896
chr8	131900000	132150000	0,021544189	1	- 0,028282877
chr8	132550000	132800000	0,01546275	1	- 0,018144062
chr8	132600000	132850000	0,003609388	1	- 0,019146711
chr8	132650000	132900000	0,02589453	1	- 0,019683345
chr8	133000000	133250000	0,027026982	1	- 0,032301736
chr8	133050000	133300000	0,003172117	1	-

					0,031000097
chr8	133100000	133350000	0,029749387	1	-0,029457706
chr8	135300000	135550000	0,007545169	1	-0,047390164
chr8	135350000	135600000	0,032698083	1	-0,050026151
chr8	136050000	136300000	0,014385348	1	-0,034283758
chr8	136100000	136350000	0,027469066	1	-0,032056977
chr8	136400000	136650000	0,007685345	1	-0,025988457
chr8	136450000	136700000	0,001340331	1	-0,026724677
chr8	136500000	136750000	0,006967739	1	-0,027322415
chr8	136550000	136800000	0,007976353	1	-0,02753581
chr8	136600000	136850000	0,001883406	1	-0,027245714
chr8	136650000	136900000	0,000489544	1	-0,026473752
chr8	136700000	136950000	0,008066732	1	-0,025963585
chr8	136950000	137200000	0,017852615	1	-0,024094923
chr8	137000000	137250000	0,001610761	1	-0,022940463

chr8	137050000	137300000	0,010023649	1	- 0,022122974
chr8	137100000	137350000	0,040888424	1	- 0,021819424
chr8	137150000	137400000	0,044156923	1	- 0,022088289
chr8	137200000	137450000	0,014644575	1	- 0,022830544
chr8	137250000	137500000	0,001217191	1	- 0,023788312
chr8	137300000	137550000	0,000488119	1	- 0,024624885
chr8	137350000	137600000	0,00184637	1	- 0,025060882
chr8	137400000	137650000	0,001134362	1	- 0,023982681
chr8	137450000	137700000	0,000362305	1	- 0,024619707
chr8	137500000	137750000	0,004562497	1	-0,02553009
chr8	137550000	137800000	0,024260818	1	- 0,026434735
chr8	137600000	137850000	0,036210279	1	- 0,027049785
chr8	137650000	137900000	0,017992198	1	- 0,027158609
chr8	137700000	137950000	0,002365978	1	- 0,026654012
chr8	137750000	138000000	0,002191149	1	-

					0,026654012
chr8	137800000	138050000	0,038654714	1	- 0,026654012
chr8	138100000	138350000	0,013287627	1	- 0,019408551
chr8	138150000	138400000	0,003397922	1	- 0,020224902
chr8	138200000	138450000	0,035543851	1	- 0,021240608
chr8	138400000	138650000	0,049256548	1	- 0,024257244
chr8	138450000	138700000	0,010301767	1	- 0,024271741
chr8	138500000	138750000	0,000693101	1	- 0,024064049
chr8	138550000	138800000	3,26E-05	1	- 0,023828871
chr8	138600000	138850000	5,43E-05	1	- 0,023796364
chr8	138650000	138900000	3,68E-05	1	- 0,024149422
chr8	138700000	138950000	0,000958699	1	- 0,024952799
chr8	138750000	139000000	0,016300496	1	- 0,026123148
chr8	138950000	139200000	0,046656375	1	- 0,029722584
chr8	139000000	139250000	0,00708268	1	-

					0,029498353
chr8	139050000	139300000	0,000386896	1	-0,02931478
chr8	139100000	139350000	0,001426771	1	-0,02931478
chr8	139150000	139400000	0,007854578	1	-0,02931478
chr8	139200000	139450000	0,014637641	1	-0,02931478
chr8	139250000	139500000	0,014779141	1	-<br>0,028911424
chr8	139300000	139550000	0,011513464	1	-<br>0,028161818
chr8	139350000	139600000	0,010082389	1	-<br>0,027420566
chr8	139400000	139650000	0,012960542	1	-<br>0,026774201
chr8	139450000	139700000	0,022617293	1	-<br>0,026211544
chr8	139500000	139750000	0,04104871	1	-<br>0,025661003
chr8	139700000	139950000	0,023379025	1	-<br>0,022557496
chr8	139750000	140000000	0,003156943	1	-<br>0,021686116
chr8	139800000	140050000	0,0018199	1	-<br>0,020950096
chr8	140300000	140550000	0,009972262	1	-<br>0,038769917
chr8	140350000	140600000	0,030778027	1	-<br>0,041200415
chr8	140700000	140950000	0,00508242	1	-

					0,028651539
chr8	140750000	141000000	0,000576777	1	- 0,027110465
chr8	140800000	141050000	0,000636124	1	- 0,026574931
chr8	140850000	141100000	0,003301938	1	- 0,026815947
chr8	143350000	143600000	0,009886531	1	- 0,012218389
chr8	143850000	144100000	0,02098168	1	- 0,022157539
chr9	1100000	1350000	0,012412742	1	- 0,024702607
chr9	1150000	1400000	0,03373425	1	- 0,023232695
chr9	1300000	1550000	0,043405136	1	- 0,022502104
chr9	1350000	1600000	0,031538349	1	- 0,023498258
chr9	1700000	1950000	0,028850785	1	-0,03394028
chr9	1750000	2000000	0,036035533	1	- 0,036558448
chr9	6300000	6550000	0,036653509	1	- 0,015484377
chr9	7150000	7400000	0,023134793	1	- 0,055186385
chr9	7200000	7450000	0,001510278	1	-0,05331676
chr9	7250000	7500000	0,017928184	1	-

					0,050529277
chr9	8150000	8400000	0,013437825	1	-0,013339805
chr9	8200000	8450000	0,000255297	1	-0,01321268
chr9	8250000	8500000	4,62E-05	1	-0,013130166
chr9	8300000	8550000	9,50E-05	1	-0,013089765
chr9	8350000	8600000	4,76E-05	1	-0,013031008
chr9	8400000	8650000	5,70E-05	1	-0,012879562
chr9	8450000	8700000	0,00055636	1	-0,012591587
chr9	8500000	8750000	0,004996389	1	-0,012173492
chr9	8550000	8800000	0,023575955	1	-0,011678478
chr9	8750000	9000000	0,031974313	1	-0,010407748
chr9	8800000	9050000	0,006145586	1	-0,010404541
chr9	8850000	9100000	0,000355404	1	-0,010440923
chr9	8900000	9150000	2,71E-06	1	-0,010450116
chr9	8950000	9200000	1,15E-07	1	-0,01041111
chr9	9000000	9250000	7,95E-07	1	-

					0,010363797
					-
chr9	9050000	9300000	8,64E-07	1	0,010363797
					-
chr9	9100000	9350000	4,40E-05	1	0,010363797
					-
chr9	9150000	9400000	0,003133351	1	0,010363797
					-
chr9	9200000	9450000	0,036837309	1	0,010363797
					-
chr9	9450000	9700000	0,011870996	1	0,012337279
					-
chr9	9500000	9750000	0,00152094	1	0,012972272
					-
chr9	9550000	9800000	0,018057428	1	0,013404232
					-
chr9	9800000	10050000	0,007449624	1	0,011546518
					-
chr9	9850000	10100000	0,014972034	1	0,010985985
					-
chr9	10200000	10450000	0,016544911	1	0,015175271
					-
chr9	10250000	10500000	0,000528685	1	0,015653733
					-
chr9	10300000	10550000	4,95E-05	1	0,016174985
					-
chr9	10350000	10600000	5,21E-05	1	0,016345717
chr9	10400000	10650000	0,001620243	1	-0,01624714

chr9	10450000	10700000	0,042233206	1	-0,016051534
chr9	10900000	11150000	0,039580147	1	-0,026487674
chr9	10950000	11200000	0,013072472	1	-0,029964424
chr9	11000000	11250000	0,00322575	1	-0,030127513
chr9	11050000	11300000	0,000390427	1	-0,030013465
chr9	11100000	11350000	4,28E-05	1	-0,029548659
chr9	11150000	11400000	0,00109134	1	-0,028676121
chr9	11200000	11450000	0,014729837	1	-0,027388464
chr9	11650000	11900000	0,014470453	1	-0,018656351
chr9	11700000	11950000	0,005022408	1	-0,018656351
chr9	11750000	12000000	0,002195569	1	-0,018241684
chr9	11800000	12050000	0,000424196	1	-0,017927854
chr9	11850000	12100000	0,007349229	1	-0,017622839
chr9	13000000	13250000	0,04895241	1	-0,022902957

chr9	13050000	13300000	0,02943914	1	-0,024119919
chr9	13500000	13750000	0,006111058	1	-0,041340919
chr9	14600000	14850000	0,030768619	1	-0,054219849
chr9	14650000	14900000	0,0067972	1	-0,054219849
chr9	16550000	16800000	0,029049505	1	-0,018618279
chr9	16850000	17100000	0,02268405	1	-0,013921388
chr9	17250000	17500000	0,034270035	1	-0,023995381
chr9	17300000	17550000	0,001401306	1	-0,023995381
chr9	17350000	17600000	0,001480249	1	-0,023995381
chr9	17400000	17650000	0,011582634	1	-0,023995381
chr9	17450000	17700000	0,030584518	1	-0,023868218
chr9	17500000	17750000	0,046102457	1	-0,023386909
chr9	19750000	20000000	0,014163184	1	-0,041503299
chr9	20500000	20750000	0,006227461	1	-0,018283692

chr9	20550000	20800000	0,000217484	1	-0,019193798
chr9	20600000	20850000	0,000864655	1	-0,018886185
chr9	20650000	20900000	0,00169903	1	-0,018719863
chr9	20700000	20950000	0,000684016	1	-0,018773987
chr9	20750000	21000000	9,39E-05	1	-0,019096187
chr9	20800000	21050000	0,001234183	1	-0,019096187
chr9	20850000	21100000	0,014011319	1	-0,019096187
chr9	21050000	21300000	0,026806664	1	-0,020499154
chr9	21100000	21350000	0,00303705	1	-0,021408628
chr9	21150000	21400000	0,004506984	1	-0,022239495
chr9	21600000	21850000	0,017954119	1	-0,013258365
chr9	21650000	21900000	0,001833063	1	-0,011770615
chr9	21700000	21950000	0,008182716	1	-0,011196558
chr9	22700000	22950000	0,004722177	1	-0,040696434

chr9	22750000	23000000	0,02785916	1	-0,038424644
chr9	23100000	23350000	0,017557787	1	-0,028950767
chr9	23150000	23400000	0,003848277	1	-0,028647901
chr9	23200000	23450000	0,001208728	1	-0,028296751
chr9	23250000	23500000	0,001014386	1	-0,028296751
chr9	23300000	23550000	0,001759416	1	-0,028296751
chr9	23350000	23600000	0,003207451	1	-0,028296751
chr9	23400000	23650000	0,004292518	1	-0,028296751
chr9	23450000	23700000	0,003760056	1	-0,027854298
chr9	23500000	23750000	0,0022293	1	-0,026753378
chr9	23550000	23800000	0,001218354	1	-0,025430643
chr9	23600000	23850000	0,001430099	1	-0,024919025
chr9	23650000	23900000	0,005408193	1	-0,024088615
chr9	23700000	23950000	0,02792089	1	-0,022927256

chr9	24000000	24250000	0,006234046	1	-0,018900709
chr9	24050000	24300000	0,003395367	1	-0,018900709
chr9	24100000	24350000	0,01838978	1	-0,018900709
chr9	24150000	24400000	0,02048305	1	-0,018900709
chr9	24200000	24450000	0,004701618	1	-0,01931956
chr9	24250000	24500000	0,003155363	1	-0,020024622
chr9	25100000	25350000	0,040426594	1	-0,021104501
chr9	25150000	25400000	0,003623745	1	-0,022585779
chr9	25200000	25450000	0,001130977	1	-0,023395676
chr9	25250000	25500000	0,002622307	1	-0,023764434
chr9	25300000	25550000	0,008287122	1	-0,023973658
chr9	25350000	25600000	0,018486153	1	-0,024243262
chr9	25400000	25650000	0,027166753	1	-0,02468192
chr9	25450000	25700000	0,024898474	1	-0,025297901
chr9	25500000	25750000	0,010693088	1	-0,026048065

chr9	25550000	25800000	0,001643833	1	-0,026869664
chr9	25600000	25850000	0,037420639	1	-0,027671653
chr9	27700000	27950000	0,008733405	1	-0,012876899
chr9	27750000	28000000	0,038368594	1	-0,012044624
chr9	28750000	29000000	0,00469969	1	-0,04398256
chr9	28800000	29050000	0,008062645	1	-0,046003854
chr9	29200000	29450000	0,021479428	1	-0,026777873
chr9	29250000	29500000	0,000304405	1	-0,027064641
chr9	29300000	29550000	0,000108985	1	-0,026806376
chr9	29350000	29600000	0,0009186	1	-0,025757467
chr9	29400000	29650000	0,039060138	1	-0,023888518
chr9	31700000	31950000	0,035687882	1	-0,03567869
chr9	31750000	32000000	0,006429171	1	-0,037289553
chr9	31800000	32050000	0,001215682	1	-0,038680486
chr9	31850000	32100000	0,035728303	1	-0,039763604

chr9	32550000	32800000	0,016573764	1	-0,019995344
chr9	72000000	72250000	0,035771222	1	-0,017316174
chr9	72600000	72850000	0,017455258	1	-0,015010231
chr9	73900000	74150000	0,014277759	1	-0,02586277
chr9	76100000	76350000	0,044714131	1	-0,03241286
chr9	76150000	76400000	0,005559515	1	-0,032939504
chr9	76200000	76450000	0,004777579	1	-0,03405076
chr9	76250000	76500000	0,030197416	1	-0,03405076
chr9	76700000	76950000	0,010709628	1	-0,052358695
chr9	76750000	77000000	0,001015015	1	-0,054772831
chr9	76800000	77050000	0,023988791	1	-0,056430085
chr9	77900000	78150000	0,037206625	1	-0,034343732
chr9	77950000	78200000	0,000808006	1	-0,033708545
chr9	78000000	78250000	0,000534778	1	-0,033595731
chr9	78050000	78300000	0,000426473	1	-0,034501032
chr9	78100000	78350000	0,010455724	1	-0,034501032

chr9	78450000	78700000	0,021682409	1	- 0,048169417
chr9	78500000	78750000	0,03002326	1	- 0,051605917
chr9	79000000	79250000	0,004707394	1	- 0,022030534
chr9	79050000	79300000	0,037494484	1	- 0,023239259
chr9	79250000	79500000	0,008857598	1	-0,02497339
chr9	85500000	85750000	0,028086329	1	- 0,017024114
chr9	85550000	85800000	0,040478153	1	- 0,018411003
chr9	85950000	86200000	0,010912917	1	- 0,010707161
chr9	86100000	86350000	0,036681747	1	-0,01065512
chr9	86150000	86400000	0,003792833	1	-0,01118714
chr9	86200000	86450000	0,002168605	1	- 0,011678777
chr9	86250000	86500000	0,017365224	1	- 0,011971439
chr9	86300000	86550000	0,032912641	1	- 0,011980746
chr9	86350000	86600000	0,014499732	1	- 0,011717159
chr9	86400000	86650000	0,001968441	1	- 0,011135186
chr9	86450000	86700000	0,010255708	1	-

					0,011659452
chr9	87100000	87350000	0,016987338	1	-0,027842914
chr9	87150000	87400000	0,00034509	1	-0,027430079
chr9	87200000	87450000	0,000285944	1	-0,027241576
chr9	87250000	87500000	0,000222399	1	-0,027470205
chr9	87300000	87550000	0,000232081	1	-0,028178533
chr9	87350000	87600000	0,005750286	1	-0,028178533
chr9	87400000	87650000	0,04121663	1	-0,028178533
chr9	87650000	87900000	0,010486126	1	-0,032346743
chr9	87700000	87950000	0,001455507	1	-0,033813721
chr9	87750000	88000000	0,024280401	1	-0,034906007
chr9	89400000	89650000	0,024351342	1	-0,017333172
chr9	90350000	90600000	0,005168524	1	-0,017625895
chr9	90400000	90650000	0,042782518	1	-0,017625895
chr9	92350000	92600000	0,048116218	1	-0,03382253

chr9	92400000	92650000	0,022006877	1	-0,034496753
chr9	92450000	92700000	0,010304081	1	-0,034496753
chr9	92500000	92750000	0,005618435	1	-0,034496753
chr9	92550000	92800000	0,003269724	1	-0,034496753
chr9	92600000	92850000	0,00143211	1	-0,034496753
chr9	92650000	92900000	0,000269937	1	-0,035122222
chr9	92700000	92950000	3,68E-05	1	-0,035700107
chr9	92750000	93000000	0,000546612	1	-0,03615354
chr9	92800000	93050000	0,004551575	1	-0,036362146
chr9	92850000	93100000	0,012843795	1	-0,036221165
chr9	92900000	93150000	0,017265358	1	-0,035701248
chr9	92950000	93200000	0,012944262	1	-0,034877445
chr9	93000000	93250000	0,006642372	1	-0,033911076
chr9	93050000	93300000	0,004422878	1	-0,03299676
chr9	93100000	93350000	0,010749912	1	-0,032275863

chr9	94200000	94450000	0,00954834	1	- 0,022277954
chr9	101450000	101700000	0,013543893	1	- 0,039131767
chr9	101500000	101750000	0,00111228	1	- 0,040943569
chr9	101550000	101800000	3,18E-05	1	- 0,042491699
chr9	101600000	101850000	0,000107975	1	- 0,041806983
chr9	101650000	101900000	0,001341219	1	- 0,040845563
chr9	101700000	101950000	0,006261994	1	- 0,039670699
chr9	101750000	102000000	0,016717292	1	-0,0384052
chr9	101800000	102050000	0,029330843	1	- 0,037186898
chr9	101850000	102100000	0,036026214	1	- 0,036095843
chr9	101900000	102150000	0,034259509	1	- 0,035094122
chr9	101950000	102200000	0,031276989	1	- 0,034021929
chr9	102000000	102250000	0,038707005	1	- 0,032669947
chr9	102600000	102850000	0,003244427	1	- 0,015480358
chr9	102650000	102900000	1,80E-05	1	-

					0,014771379
chr9	102700000	102950000	0,000122528	1	- 0,014512901
chr9	102750000	103000000	0,010933616	1	- 0,014560473
chr9	103500000	103750000	0,014356834	1	- 0,040930262
chr9	106300000	106550000	0,010729546	1	- 0,014686319
chr9	107200000	107450000	0,005263116	1	- 0,048293307
chr9	107250000	107500000	0,018481355	1	- 0,051109767
chr9	107400000	107650000	0,027107155	1	- 0,048792616
chr9	107450000	107700000	0,002918499	1	- 0,046678948
chr9	107500000	107750000	0,000238367	1	- 0,044958427
chr9	107550000	107800000	0,001373925	1	- 0,043984966
chr9	107600000	107850000	0,002851623	1	- 0,043841496
chr9	107650000	107900000	0,001372967	1	- 0,044368341
chr9	107700000	107950000	0,000154364	1	- 0,045243674
chr9	107750000	108000000	0,000370697	1	-

					0,046100797
chr9	107800000	108050000	0,005606695	1	- 0,046622356
chr9	107850000	108100000	0,027435499	1	- 0,042935781
chr9	108450000	108700000	0,013663494	1	- 0,024911747
chr9	108850000	109100000	0,011362266	1	- 0,037243113
chr9	108900000	109150000	0,000555692	1	- 0,036562691
chr9	108950000	109200000	0,005164884	1	- 0,036562691
chr9	110000000	110250000	0,007315365	1	- 0,021959497
chr9	110050000	110300000	0,006700828	1	- 0,022000375
chr9	111450000	111700000	0,038017334	1	- 0,018417625
chr9	112950000	113200000	0,01794972	1	- 0,013595348
chr9	113650000	113900000	0,010931127	1	-0,02027989
chr9	113700000	113950000	0,016292209	1	-0,02027989
chr9	116550000	116800000	0,034697285	1	- 0,023566465
chr9	116600000	116850000	0,002870704	1	- 0,023566465
chr9	116650000	116900000	0,018270385	1	-

					0,023566465
chr9	118800000	119050000	0,011028919	1	-0,02520253
chr9	118850000	119100000	0,037072109	1	-0,02333933
chr9	119550000	119800000	0,004663802	1	- 0,040383033
chr9	119600000	119850000	0,017211291	1	- 0,038484857
chr9	120100000	120350000	0,018586471	1	- 0,026479913
chr9	120150000	120400000	0,003203217	1	- 0,026529319
chr9	120200000	120450000	0,00016528	1	- 0,026867433
chr9	120250000	120500000	0,000303595	1	-0,02745052
chr9	120300000	120550000	0,003241953	1	-0,02818182
chr9	120350000	120600000	0,009409878	1	- 0,028881087
chr9	120400000	120650000	0,010036435	1	- 0,029287484
chr9	120450000	120700000	0,003281497	1	- 0,029106398
chr9	120500000	120750000	0,000846433	1	- 0,028103576
chr9	120550000	120800000	0,024737155	1	- 0,027057669
chr9	121550000	121800000	0,046649749	1	- 0,027594186
chr9	121600000	121850000	0,002571797	1	-

					0,028661642
chr9	121650000	121900000	9,87E-05	1	-0,029606388
chr9	121700000	121950000	0,000477437	1	-0,030171581
chr9	121750000	122000000	0,001036167	1	-0,030230309
chr9	121800000	122050000	0,000612146	1	-0,029943707
chr9	121850000	122100000	0,000108156	1	-0,029503041
chr9	121900000	122150000	4,02E-06	1	-0,029086534
chr9	121950000	122200000	1,96E-08	1	-0,028817395
chr9	122000000	122250000	1,40E-07	1	-0,02872838
chr9	122050000	122300000	2,07E-06	1	-0,028763755
chr9	122100000	122350000	2,34E-06	1	-0,028815068
chr9	122150000	122400000	4,03E-05	1	-0,028785768
chr9	122200000	122450000	0,003994643	1	-0,028656772
chr9	122650000	122900000	0,012710068	1	-0,047046689
chr9	122700000	122950000	0,00937971	1	-0,049947562

chr9	124600000	124850000	0,006241439	1	-0,020317984
chr9	124650000	124900000	0,002255457	1	-0,019429865
chr9	124700000	124950000	0,042890923	1	-0,018190005
chr9	125450000	125700000	0,023207155	1	-0,009305241
chr9	125500000	125750000	0,000740913	1	-0,008786181
chr9	125550000	125800000	0,000155872	1	-0,008466875
chr9	125600000	125850000	0,034725267	1	-0,008385854
chr9	128450000	128700000	0,02436871	1	-0,01877573
chr9	128850000	129100000	0,003772495	1	-0,012367454
chr9	128900000	129150000	0,009558875	1	-0,011781306
chr9	134800000	135050000	0,029522031	1	-0,008783445
chr9	135450000	135700000	0,04490403	1	-0,013688921
chr9	140250000	140500000	0,007564529	1	-0,003697758
chr10	350000	600000	0,048863945	1	-0,012341829
chr10	1500000	1750000	0,01895013	1	-

					0,031415942
chr10	1550000	1800000	0,001606989	1	-0,031415942
chr10	1600000	1850000	0,011261508	1	-0,031415942
chr10	1650000	1900000	0,0314515	1	-0,031534901
chr10	1700000	1950000	0,031723984	1	-0,030928435
chr10	1750000	2000000	0,015855151	1	-0,029934705
chr10	1800000	2050000	0,005306208	1	-0,028902217
chr10	1850000	2100000	0,00257465	1	-0,028067538
chr10	1900000	2150000	0,00517499	1	-0,025461577
chr10	1950000	2200000	0,025840756	1	-0,024202815
chr10	2500000	2750000	0,004318259	1	-0,010829639
chr10	2550000	2800000	0,043981564	1	-0,010829639
chr10	3100000	3350000	0,011012326	1	-0,020568506
chr10	3150000	3400000	0,017107828	1	-0,021675778
chr10	4250000	4500000	0,007402156	1	-

					0,029346958
chr10	4300000	4550000	0,000268126	1	-<br>0,029346958
chr10	4350000	4600000	3,01E-06	1	-<br>0,029346958
chr10	4400000	4650000	0,000492343	1	-<br>0,029346958
chr10	4450000	4700000	0,025073129	1	-<br>0,029291337
chr10	4900000	5150000	0,022058215	1	-<br>0,060359046
chr10	4950000	5200000	0,016179578	1	-<br>0,056427669
chr10	6450000	6700000	0,04645654	1	-<br>0,010307012
chr10	6500000	6750000	0,001726544	1	-<br>0,010307012
chr10	6950000	7200000	0,014138744	1	-<br>0,024585357
chr10	7000000	7250000	0,00469969	1	-<br>0,024585357
chr10	7050000	7300000	0,014135119	1	-<br>0,025254783
chr10	7100000	7350000	0,006536136	1	-<br>0,024995274
chr10	7150000	7400000	0,002380163	1	-<br>0,024215179
chr10	7200000	7450000	0,028529962	1	-

					0,023437124
chr10	7350000	7600000	0,036199225	1	-0,02735928
chr10	7400000	7650000	0,005649368	1	-0,026010388
chr10	19100000	19350000	0,021188505	1	-0,027082826
chr10	20800000	21050000	0,006424635	1	-0,031344466
chr10	20850000	21100000	0,013619235	1	-0,033185348
chr10	22100000	22350000	0,033430905	1	-0,023571307
chr10	22850000	23100000	0,017394823	1	-0,013624158
chr10	24350000	24600000	0,012053263	1	-0,040389384
chr10	24400000	24650000	0,0024698	1	-0,042963642
chr10	24450000	24700000	0,022657854	1	-0,044262139
chr10	24600000	24850000	0,041551796	1	-0,041709692
chr10	24650000	24900000	0,011440736	1	-0,039952201
chr10	24700000	24950000	0,001485492	1	-0,0384405
chr10	24750000	25000000	8,79E-05	1	-0,037390895
chr10	24800000	25050000	3,04E-06	1	-

					0,036816118
chr10	24850000	25100000	8,72E-08	1	-0,036576421
chr10	24900000	25150000	1,51E-06	1	-0,0364918
chr10	24950000	25200000	0,000106378	1	-0,036435682
chr10	25000000	25250000	0,00145502	1	-0,038307753
chr10	25050000	25300000	0,007061698	1	-0,039430594
chr10	25100000	25350000	0,015185417	1	-0,040481738
chr10	25150000	25400000	0,014944713	1	-0,041144281
chr10	25200000	25450000	0,005542107	1	-0,041115367
chr10	25250000	25500000	0,00065396	1	-0,040182538
chr10	25300000	25550000	0,004747448	1	-0,038273247
chr10	25850000	26100000	0,002399186	1	-0,0167473
chr10	25900000	26150000	0,002529976	1	-0,016783077
chr10	25950000	26200000	0,001876027	1	-0,016783077
chr10	30150000	30400000	0,045090504	1	-0,011735324
chr10	30200000	30450000	0,04536309	1	-

					0,011526096
chr10	30250000	30500000	0,023526829	1	-0,011303604
chr10	31000000	31250000	0,014856933	1	-0,022942092
chr10	31050000	31300000	0,049045531	1	-0,021259127
chr10	31500000	31750000	0,012192147	1	-0,011377701
chr10	31550000	31800000	0,008351296	1	-0,011377701
chr10	31700000	31950000	0,037184383	1	-0,011750743
chr10	31750000	32000000	0,006332609	1	-0,012300947
chr10	36000000	36250000	0,009127114	1	-0,041179391
chr10	38000000	38250000	0,048302062	1	-0,035528428
chr10	38050000	38300000	0,005008136	1	-0,037640443
chr10	38100000	38350000	0,000248798	1	-0,039239795
chr10	38150000	38400000	0,001965713	1	-0,04020066
chr10	38200000	38450000	0,016280317	1	-0,036690244
chr10	38650000	38900000	0,032550053	1	-0,023734636

chr10	38700000	38950000	0,014607038	1	-0,022963637
chr10	38750000	39000000	0,043971341	1	-0,02154454
chr10	44300000	44550000	0,010064878	1	-0,033865738
chr10	44800000	45050000	0,010607973	1	-0,016836497
chr10	44850000	45100000	0,04943073	1	-0,015818915
chr10	45000000	45250000	0,019397779	1	-0,016676733
chr10	49500000	49750000	0,031582663	1	-0,017096332
chr10	49550000	49800000	0,000306343	1	-0,017336752
chr10	49600000	49850000	0,006563162	1	-0,018536031
chr10	50050000	50300000	0,004777253	1	-0,034637738
chr10	50100000	50350000	0,037527454	1	-0,034637738
chr10	52650000	52900000	0,038787749	1	-0,02161929
chr10	53600000	53850000	0,029936734	1	-0,036897736
chr10	54950000	55200000	0,046846493	1	-0,039273116
chr10	55000000	55250000	0,003158237	1	-0,038287449

chr10	55050000	55300000	0,005379602	1	-0,036477472
chr10	55450000	55700000	0,022248099	1	-0,024283022
chr10	55500000	55750000	0,007442309	1	-0,02549608
chr10	55750000	56000000	0,021509157	1	-0,029830369
chr10	55800000	56050000	0,001617086	1	-0,029374958
chr10	55850000	56100000	0,000730824	1	-0,028517925
chr10	55900000	56150000	0,00999025	1	-0,027416537
chr10	55950000	56200000	0,042455457	1	-0,026262055
chr10	56100000	56350000	0,040050634	1	-0,024268314
chr10	56150000	56400000	0,007751685	1	-0,024407717
chr10	56200000	56450000	0,000473089	1	-0,024913984
chr10	56250000	56500000	0,001796514	1	-0,025676564
chr10	56300000	56550000	0,01326582	1	-0,026565086
chr10	56350000	56600000	0,032037025	1	-0,02746289
chr10	56400000	56650000	0,040297139	1	-0,028287087

chr10	56450000	56700000	0,033191749	1	-0,028991768
chr10	56500000	56750000	0,020174442	1	-0,029562358
chr10	56550000	56800000	0,009624481	1	-0,029995515
chr10	56600000	56850000	0,003608726	1	-0,030276977
chr10	56650000	56900000	0,000964637	1	-0,030365173
chr10	56700000	56950000	0,000132466	1	-0,030365173
chr10	56750000	57000000	7,58E-06	1	-0,030365173
chr10	56800000	57050000	6,32E-05	1	-0,030366695
chr10	56850000	57100000	0,000948192	1	-0,030327251
chr10	56900000	57150000	0,005211795	1	-0,030255821
chr10	56950000	57200000	0,015746267	1	-0,030040284
chr10	57000000	57250000	0,030463418	1	-0,029638397
chr10	57050000	57300000	0,039230885	1	-0,029022082
chr10	57100000	57350000	0,033612309	1	-0,028201967

chr10	57150000	57400000	0,01931142	1	-0,027281082
chr10	57200000	57450000	0,008261968	1	-0,026375115
chr10	57250000	57500000	0,004100275	1	-0,025593769
chr10	57300000	57550000	0,005382598	1	-0,02500417
chr10	57350000	57600000	0,018315819	1	-0,024587929
chr10	57850000	58100000	0,02413081	1	-0,015330329
chr10	57900000	58150000	0,004022341	1	-0,014747045
chr10	57950000	58200000	0,000905947	1	-0,014269844
chr10	58000000	58250000	0,018898424	1	-0,013917117
chr10	58500000	58750000	0,012533077	1	-0,024285241
chr10	59500000	59750000	0,03256758	1	-0,031424724
chr10	62850000	63100000	0,00598527	1	-0,012866129
chr10	64950000	65200000	0,043738003	1	-0,015335557
chr10	65750000	66000000	0,042781019	1	-0,04158019
chr10	65800000	66050000	0,002253414	1	-0,040145491

chr10	65850000	66100000	0,016919557	1	-0,038070593
chr10	68950000	69200000	0,013006912	1	-0,036419502
chr10	69600000	69850000	0,001166637	1	-0,006435339
chr10	71400000	71650000	0,015429854	1	-0,022316421
chr10	71450000	71700000	0,044189368	1	-0,022316421
chr10	72700000	72950000	0,00219881	1	-0,025513734
chr10	72750000	73000000	0,005543438	1	-0,025513734
chr10	76000000	76250000	0,006397429	1	-0,023693465
chr10	76050000	76300000	0,042812395	1	-0,022133447
chr10	77400000	77650000	0,027934085	1	-0,043146742
chr10	77450000	77700000	0,00866945	1	-0,040647048
chr10	79000000	79250000	0,006226755	1	-0,038265879
chr10	80700000	80950000	0,025514279	1	-0,020878402
chr10	81100000	81350000	0,011748571	1	-0,012764582

chr10	81150000	81400000	0,000316037	1	-0,012647266
chr10	81200000	81450000	0,000147565	1	-0,012464574
chr10	81250000	81500000	0,000410574	1	-0,012335398
chr10	81300000	81550000	0,000302051	1	-0,01238336
chr10	81350000	81600000	0,000131046	1	-0,012650742
chr10	81400000	81650000	0,001981011	1	-0,013057384
chr10	81450000	81700000	0,011597423	1	-0,013057384
chr10	81500000	81750000	0,01579421	1	-0,013057384
chr10	81550000	81800000	0,005364289	1	-0,013057384
chr10	81600000	81850000	0,000660867	1	-0,013057384
chr10	81650000	81900000	0,003100769	1	-0,013437054
chr10	81700000	81950000	0,013115367	1	-0,0136182
chr10	81750000	82000000	0,010861769	1	-0,013515429
chr10	81800000	82050000	0,002104812	1	-0,013174132
chr10	81850000	82100000	0,014751963	1	-0,013680241

chr10	82450000	82700000	0,009303929	1	-0,03447153
chr10	82500000	82750000	0,008462033	1	-0,032803862
chr10	82750000	83000000	0,011607116	1	-0,028767347
chr10	82800000	83050000	0,000933835	1	-0,029613968
chr10	82850000	83100000	0,006795419	1	-0,030659555
chr10	82900000	83150000	0,024342632	1	-0,031582859
chr10	82950000	83200000	0,024239655	1	-0,032045448
chr10	83000000	83250000	0,006694147	1	-0,031742227
chr10	83050000	83300000	0,001590526	1	-0,030482691
chr10	83100000	83350000	0,039298144	1	-0,029390677
chr10	84200000	84450000	0,046483011	1	-0,039722279
chr10	84250000	84500000	0,014520362	1	-0,041529994
chr10	84300000	84550000	0,002274028	1	-0,043157945
chr10	84350000	84600000	0,000110245	1	-0,044465719
chr10	84400000	84650000	3,88E-06	1	-

					0,045333958
chr10	84450000	84700000	4,65E-06	1	-0,045719175
chr10	84500000	84750000	6,56E-06	1	-0,045692059
chr10	84550000	84800000	0,000254063	1	-0,045437465
chr10	84600000	84850000	0,00282321	1	-0,045214312
chr10	84650000	84900000	0,009202337	1	-0,045279114
chr10	84700000	84950000	0,01203854	1	-0,045791784
chr10	84750000	85000000	0,006443854	1	-0,046758199
chr10	84800000	85050000	0,001057327	1	-0,048009854
chr10	84850000	85100000	0,000208831	1	-0,049254823
chr10	84900000	85150000	0,003402012	1	-0,050162146
chr10	84950000	85200000	0,022466909	1	-0,050463932
chr10	85150000	85400000	0,026689988	1	-0,045350252
chr10	85200000	85450000	0,00426922	1	-0,043512517
chr10	85250000	85500000	0,000226571	1	-

					0,042004226
chr10	85300000	85550000	0,000888798	1	-0,041019437
chr10	85350000	85600000	0,005595858	1	-0,040649165
chr10	85400000	85650000	0,010486036	1	-0,044636217
chr10	85450000	85700000	0,007848429	1	-0,045113898
chr10	85500000	85750000	0,001977352	1	-0,044887399
chr10	85550000	85800000	0,000317511	1	-0,043744904
chr10	85600000	85850000	0,007405486	1	-0,041594532
chr10	86000000	86250000	0,044519462	1	-0,026058469
chr10	86300000	86550000	0,008879518	1	-0,032260094
chr10	86350000	86600000	0,000784837	1	-0,031358766
chr10	86400000	86650000	0,007459649	1	-0,030234173
chr10	86450000	86700000	0,034797313	1	-0,029003705
chr10	87550000	87800000	0,025570734	1	-0,056614987
chr10	92100000	92350000	0,012869238	1	-

					0,032982545
chr10	96550000	96800000	0,038859203	1	-0,023053116
chr10	96600000	96850000	0,013061208	1	-0,023053116
chr10	97000000	97250000	0,014384015	1	-0,013352153
chr10	97050000	97300000	0,001098851	1	-0,013638938
chr10	97100000	97350000	0,002983328	1	-0,013751129
chr10	97150000	97400000	0,001281788	1	-0,013621828
chr10	97200000	97450000	0,00033465	1	-0,013282363
chr10	97250000	97500000	0,004091574	1	-0,012827119
chr10	97300000	97550000	0,022378251	1	-0,012367984
chr10	97350000	97600000	0,043247214	1	-0,011985024
chr10	97400000	97650000	0,041949085	1	-0,011694166
chr10	97450000	97700000	0,026291973	1	-0,01144588
chr10	97500000	97750000	0,015983945	1	-0,011162282
chr10	97550000	97800000	0,017024641	1	-0,011162282

chr10	97600000	97850000	0,032592728	1	-0,011162282
chr10	97750000	98000000	0,026738313	1	-0,010790703
chr10	97800000	98050000	0,003524406	1	-0,010349676
chr10	97850000	98100000	0,017445451	1	-0,009937414
chr10	98000000	98250000	0,040213961	1	-0,01012326
chr10	100450000	100700000	0,014251789	1	-0,029787851
chr10	100500000	100750000	0,002880432	1	-0,029787851
chr10	100550000	100800000	0,030527579	1	-0,029787851
chr10	100950000	101200000	0,046097494	1	-0,021855549
chr10	106350000	106600000	0,021512238	1	-0,048323977
chr10	106850000	107100000	0,020388379	1	-0,023991914
chr10	106900000	107150000	0,001244336	1	-0,024093157
chr10	106950000	107200000	4,25E-05	1	-0,024423514
chr10	107000000	107250000	0,000681764	1	-0,02505018
chr10	107050000	107300000	0,008808094	1	-0,026038986

chr10	107100000	107350000	0,046902534	1	-0,027403503
chr10	107400000	107650000	0,010953822	1	-0,033304326
chr10	107450000	107700000	0,001007538	1	-0,032280883
chr10	107500000	107750000	0,010402756	1	-0,030819411
chr10	107800000	108050000	0,016449675	1	-0,025168812
chr10	107850000	108100000	0,00091246	1	-0,025168812
chr10	107900000	108150000	0,000349617	1	-0,025168812
chr10	107950000	108200000	0,004610036	1	-0,025168812
chr10	108000000	108250000	0,021024133	1	-0,025186576
chr10	108300000	108550000	0,035099482	1	-0,030682442
chr10	108350000	108600000	0,007286623	1	-0,032097458
chr10	108400000	108650000	0,000534861	1	-0,033320996
chr10	108450000	108700000	1,26E-05	1	-0,034196708
chr10	108500000	108750000	1,42E-05	1	-0,034478909

chr10	108550000	108800000	1,14E-05	1	-0,034293255
chr10	108600000	108850000	7,89E-06	1	-0,034629078
chr10	108650000	108900000	0,000143179	1	-0,034989653
chr10	108700000	108950000	0,000805522	1	-0,035284378
chr10	108750000	109000000	0,001401833	1	-0,035468471
chr10	108800000	109050000	0,000994149	1	-0,035597363
chr10	108850000	109100000	0,0003158	1	-0,035849273
chr10	108900000	109150000	5,01E-05	1	-0,036257556
chr10	108950000	109200000	9,27E-06	1	-0,03673015
chr10	109000000	109250000	1,83E-05	1	-0,037042319
chr10	109050000	109300000	9,36E-05	1	-0,037445698
chr10	109100000	109350000	0,000249624	1	-0,03770543
chr10	109150000	109400000	0,000232974	1	-0,03770543
chr10	109200000	109450000	5,18E-05	1	-0,03770543
chr10	109250000	109500000	0,000159951	1	-0,03770543
chr10	109300000	109550000	0,005993928	1	-0,037806504
chr10	109850000	110100000	0,007414601	1	-

					0,016084471
chr10	109900000	110150000	0,01712763	1	-0,016888311
chr10	111100000	111350000	0,008314983	1	-0,045675858
chr10	111150000	111400000	0,000799006	1	-0,047617536
chr10	111200000	111450000	0,009732939	1	-0,049031651
chr10	112900000	113150000	0,044597593	1	-0,051369895
chr10	112950000	113200000	0,012162071	1	-0,051696562
chr10	113000000	113250000	0,001459718	1	-0,05138228
chr10	113050000	113300000	0,000109414	1	-0,050489102
chr10	113100000	113350000	0,001119514	1	-0,049172346
chr10	113150000	113400000	0,008089411	1	-0,047644199
chr10	113200000	113450000	0,022020876	1	-0,046123392
chr10	113250000	113500000	0,032484357	1	-0,044786536
chr10	113300000	113550000	0,030393313	1	-0,043715836
chr10	113350000	113600000	0,020036942	1	-0,043715836

chr10	113400000	113650000	0,01146341	1	-0,043715836
chr10	113450000	113700000	0,009574876	1	-0,043715836
chr10	113500000	113750000	0,021564581	1	-0,043715836
chr10	118200000	118450000	0,002292969	1	-0,021837088
chr10	118250000	118500000	1,29E-05	1	-0,022827488
chr10	118300000	118550000	1,28E-05	1	-0,023164236
chr10	118350000	118600000	7,97E-06	1	-0,023143793
chr10	118400000	118650000	5,85E-07	1	-0,023002515
chr10	118450000	118700000	8,26E-09	1	-0,022880723
chr10	118500000	118750000	2,29E-08	1	-0,022829299
chr10	118550000	118800000	6,03E-06	1	-0,022838902
chr10	118600000	118850000	0,000242901	1	-0,022871447
chr10	118650000	118900000	0,003104564	1	-0,021459242
chr10	118700000	118950000	0,018676501	1	-0,020564609

chr10	118900000	119150000	0,039247794	1	-0,018398679
chr10	118950000	119200000	0,005495286	1	-0,019295083
chr10	119350000	119600000	0,013872077	1	-0,029978933
chr10	119400000	119650000	0,010341383	1	-0,028321643
chr10	123350000	123600000	0,026557287	1	-0,014174745
chr10	123650000	123900000	0,007742271	1	-0,012530289
chr10	123700000	123950000	0,003091215	1	-0,012023657
chr10	123750000	124000000	0,014606051	1	-0,011781858
chr10	124050000	124300000	0,03319507	1	-0,008418726
chr10	124100000	124350000	0,001502759	1	-0,008635511
chr10	124150000	124400000	0,001872027	1	-0,008747114
chr10	124200000	124450000	0,001443554	1	-0,008580795
chr10	124250000	124500000	0,007388043	1	-0,008034746
chr10	126900000	127150000	0,013428184	1	-0,031665709

chr10	128150000	128400000	0,008603565	1	-0,038024709
chr10	129000000	129250000	0,023647138	1	-0,048251471
chr10	129050000	129300000	0,005446436	1	-0,050390698
chr10	129100000	129350000	0,000733771	1	-0,05199108
chr10	129150000	129400000	4,13E-05	1	-0,05316156
chr10	129200000	129450000	0,000339005	1	-0,05394125
chr10	129250000	129500000	0,00661316	1	-0,054312455
chr10	129300000	129550000	0,049946687	1	-0,054227561
chr10	129700000	129950000	0,034665812	1	-0,03191134
chr10	130050000	130300000	0,026879496	1	-0,043698589
chr10	130100000	130350000	0,005713527	1	-0,041481338
chr10	130700000	130950000	0,037952585	1	-0,022696916
chr10	130750000	131000000	0,010997107	1	-0,021809422
chr10	130800000	131050000	0,001100499	1	-0,021051998
chr10	130850000	131100000	0,005690004	1	-0,020441858
chr10	131400000	131650000	0,020062705	1	-0,040255337

chr10	131450000	131700000	0,019437037	1	-0,042827919
chr10	132300000	132550000	0,025270775	1	-0,014658188
chr10	132350000	132600000	0,002083007	1	-0,015582981
chr10	132400000	132650000	0,023549261	1	-0,014693236
chr10	133300000	133550000	0,005812748	1	-0,025744399
chr10	133350000	133600000	0,02981284	1	-0,02715356
chr10	134750000	135000000	0,004245234	1	-0,016947229
chr10	134800000	135050000	0,004531914	1	-0,017900233
chr10	134850000	135100000	0,022624422	1	-0,018218536
chr10	134900000	135150000	0,037734643	1	-0,018019948
chr10	134950000	135200000	0,043027164	1	-0,017516075
chr11	400000	650000	0,004909598	1	-0,004204548
chr11	450000	700000	0,00604975	1	-0,004380852
chr11	2000000	2250000	0,014671781	1	-0,040597691
chr11	2050000	2300000	0,00091377	1	-

					0,039505331
chr11	2100000	2350000	0,006489844	1	-0,038975695
chr11	3150000	3400000	0,037675185	1	-0,011782672
chr11	4450000	4700000	0,032312649	1	-0,02921339
chr11	4500000	4750000	0,009673327	1	-0,027537153
chr11	11350000	11600000	0,018580658	1	-0,017044717
chr11	15250000	15500000	0,021683258	1	-0,032544042
chr11	15300000	15550000	0,003021311	1	-0,032544042
chr11	15350000	15600000	0,004711492	1	-0,032629414
chr11	16400000	16650000	0,005384613	1	-0,024366045
chr11	16450000	16700000	0,004440271	1	-0,025582738
chr11	18800000	19050000	0,020189136	1	-0,005899828
chr11	18850000	19100000	0,045423763	1	-0,005833358
chr11	18900000	19150000	0,021364722	1	-0,005833358
chr11	18950000	19200000	0,002350567	1	-0,005833358

chr11	19000000	19250000	0,000938281	1	- 0,005833358
chr11	20050000	20300000	0,007388945	1	-0,02635726
chr11	20700000	20950000	0,009492089	1	- 0,036727952
chr11	20750000	21000000	0,011987091	1	- 0,034889951
chr11	21950000	22200000	0,029514977	1	- 0,031274945
chr11	23250000	23500000	0,006046912	1	- 0,023688852
chr11	23300000	23550000	0,000953372	1	- 0,023688852
chr11	23350000	23600000	0,005740812	1	- 0,023688852
chr11	23400000	23650000	0,009875766	1	- 0,023688852
chr11	23450000	23700000	0,006957873	1	- 0,023479207
chr11	23500000	23750000	0,003647469	1	- 0,022878708
chr11	23550000	23800000	0,004096326	1	- 0,022345893
chr11	23600000	23850000	0,01504644	1	- 0,021843328
chr11	24000000	24250000	0,005833263	1	- 0,014630857
chr11	24050000	24300000	0,048342418	1	-

					0,014893476
chr11	24700000	24950000	0,0048336	1	-0,032639337
chr11	24750000	25000000	0,003361674	1	-0,03115225
chr11	25300000	25550000	0,04427344	1	-0,01345091
chr11	25600000	25850000	0,046401973	1	-0,019274142
chr11	25650000	25900000	0,003978634	1	-0,019274142
chr11	25700000	25950000	0,000388587	1	-0,019274142
chr11	25750000	26000000	0,002889018	1	-0,019274142
chr11	25800000	26050000	0,007355934	1	-0,019274142
chr11	25850000	26100000	0,004206283	1	-0,019030663
chr11	25900000	26150000	0,00061423	1	-0,018584009
chr11	25950000	26200000	0,003711939	1	-0,01811654
chr11	26000000	26250000	0,043558585	1	-0,017824145
chr11	26550000	26800000	0,04633853	1	-0,031714582
chr11	26600000	26850000	0,004224319	1	-0,03357919
chr11	26650000	26900000	0,000536474	1	-0,035028226
chr11	26700000	26950000	0,006405843	1	-

					0,035878004
chr11	26750000	27000000	0,030930184	1	-0,036048065
chr11	26900000	27150000	0,047032382	1	-0,033386743
chr11	26950000	27200000	0,019259226	1	-0,032074239
chr11	27000000	27250000	0,004552464	1	-0,03087917
chr11	27050000	27300000	0,00067588	1	-0,029934032
chr11	27100000	27350000	0,000202009	1	-0,029299378
chr11	27150000	27400000	0,001111388	1	-0,028952112
chr11	27200000	27450000	0,015203034	1	-0,028787148
chr11	31500000	31750000	0,006174031	1	-0,028122754
chr11	31550000	31800000	0,006432528	1	-0,029694059
chr11	34050000	34300000	0,048926063	1	-0,018143238
chr11	37000000	37250000	0,014627354	1	-0,018899782
chr11	38450000	38700000	0,03209316	1	-0,011980453
chr11	38500000	38750000	0,011380308	1	-0,012582944

chr11	38550000	38800000	0,008959699	1	- 0,013002124
chr11	38600000	38850000	0,01199104	1	-0,01329007
chr11	38650000	38900000	0,014016937	1	- 0,013541624
chr11	38700000	38950000	0,010203338	1	- 0,013837342
chr11	38750000	39000000	0,004102802	1	- 0,014199955
chr11	38800000	39050000	0,001006248	1	- 0,014581842
chr11	38850000	39100000	0,000380295	1	- 0,014901236
chr11	38900000	39150000	0,001026045	1	- 0,015109875
chr11	38950000	39200000	0,005404883	1	- 0,015222615
chr11	39000000	39250000	0,017435676	1	- 0,015326568
chr11	39050000	39300000	0,029336791	1	- 0,015526145
chr11	39100000	39350000	0,025976339	1	- 0,015884412
chr11	39150000	39400000	0,012227438	1	- 0,017966239
chr11	39200000	39450000	0,002954546	1	- 0,018039206
chr11	39250000	39500000	0,000329387	1	-

					0,018057391
chr11	39300000	39550000	1,69E-05	1	-0,018088444
chr11	39350000	39600000	2,88E-06	1	-0,01819017
chr11	39400000	39650000	3,88E-05	1	-0,01842039
chr11	39450000	39700000	0,000619896	1	-0,018826104
chr11	39500000	39750000	0,00482912	1	-0,019421369
chr11	39550000	39800000	0,019016292	1	-0,020147316
chr11	39600000	39850000	0,038732015	1	-0,020846589
chr11	39650000	39900000	0,038116199	1	-0,021285405
chr11	39700000	39950000	0,013816825	1	-0,021215357
chr11	39750000	40000000	0,00199087	1	-0,020470909
chr11	39800000	40050000	0,024537541	1	-0,019036663
chr11	40750000	41000000	0,004419396	1	-0,027991423
chr11	40800000	41050000	0,03914925	1	-0,029365153
chr11	41250000	41500000	0,042760776	1	-0,015537724
chr11	41650000	41900000	0,021433704	1	-

					0,031344437
chr11	42050000	42300000	0,048115534	1	-0,020723071
chr11	42100000	42350000	0,012161201	1	-0,019765097
chr11	42150000	42400000	0,008819288	1	-0,019093585
chr11	42200000	42450000	0,00918006	1	-0,018702904
chr11	42250000	42500000	0,004305765	1	-0,01836009
chr11	42300000	42550000	0,001065932	1	-0,017944847
chr11	42350000	42600000	0,023630659	1	-0,017492873
chr11	42700000	42950000	0,006386855	1	-0,024461317
chr11	44500000	44750000	0,029483102	1	-0,019327039
chr11	44550000	44800000	0,006553171	1	-0,019358179
chr11	44600000	44850000	0,000537999	1	-0,019100563
chr11	44650000	44900000	0,000477318	1	-0,01858474
chr11	44700000	44950000	0,007475035	1	-0,017896156
chr11	44750000	45000000	0,035628368	1	-0,017141744
chr11	48400000	48650000	0,021795887	1	-

					0,040479367
chr11	48450000	48700000	0,009412962	1	-0,038230311
chr11	48800000	49050000	0,007509019	1	-0,028328587
chr11	48850000	49100000	0,000225841	1	-0,028753044
chr11	48900000	49150000	0,000421874	1	-0,028894328
chr11	48950000	49200000	0,000208173	1	-0,028520648
chr11	49000000	49250000	0,000939665	1	-0,027497389
chr11	49050000	49300000	0,02865861	1	-0,025791767
chr11	50100000	50350000	0,004488484	1	-0,033344179
chr11	50150000	50400000	0,001200812	1	-0,034885114
chr11	50200000	50450000	0,015578428	1	-0,035740082
chr11	50700000	50950000	0,007354166	1	-0,023572053
chr11	50750000	51000000	0,028195542	1	-0,023572053
chr11	51050000	51300000	0,014355853	1	-0,02954134
chr11	51100000	51350000	0,000869483	1	-0,031219715

chr11	51150000	51400000	0,010457079	1	-0,032231467
chr11	56300000	56550000	0,007773758	1	-0,042223778
chr11	56350000	56600000	0,000410105	1	-0,043981528
chr11	56400000	56650000	8,50E-05	1	-0,045125465
chr11	56450000	56700000	0,001732303	1	-0,045678657
chr11	56500000	56750000	0,01466234	1	-0,045719904
chr11	59950000	60200000	0,003257111	1	-0,034786425
chr11	60000000	60250000	0,011966057	1	-0,034786425
chr11	60500000	60750000	0,00624078	1	-0,016389727
chr11	60550000	60800000	0,041114437	1	-0,015679268
chr11	60700000	60950000	0,012707326	1	-0,016857984
chr11	60750000	61000000	0,000960571	1	-0,01760373
chr11	60800000	61050000	0,005740447	1	-0,018180787
chr11	62800000	63050000	0,01726611	1	-0,019829879
chr11	76500000	76750000	0,020656384	1	-

					0,007489002
chr11	76550000	76800000	0,017774057	1	- 0,007798843
chr11	78400000	78650000	0,012823031	1	- 0,044071169
chr11	78450000	78700000	0,001120796	1	- 0,042779982
chr11	78500000	78750000	0,005934336	1	- 0,041601881
chr11	78550000	78800000	0,011815293	1	- 0,041092514
chr11	78600000	78850000	0,00461779	1	- 0,041453799
chr11	78650000	78900000	0,000550119	1	- 0,042434017
chr11	78700000	78950000	0,001675472	1	- 0,043435767
chr11	78750000	79000000	0,003933638	1	- 0,043777964
chr11	78800000	79050000	0,001390955	1	- 0,042977779
chr11	78850000	79100000	0,003067517	1	- 0,040934627
chr11	79250000	79500000	0,006688701	1	- 0,026717467
chr11	79300000	79550000	0,001475959	1	- 0,026717467
chr11	79350000	79600000	0,00991079	1	-

					0,026717467
chr11	79400000	79650000	0,018751604	1	-0,026822117
chr11	79450000	79700000	0,015443636	1	-0,027403899
chr11	79500000	79750000	0,007182167	1	-0,028203963
chr11	79550000	79800000	0,001934875	1	-0,029019936
chr11	79600000	79850000	0,000231427	1	-0,029721834
chr11	79650000	79900000	1,82E-05	1	-0,030249493
chr11	79700000	79950000	0,000584682	1	-0,030583586
chr11	79750000	80000000	0,013843705	1	-0,030722536
chr11	80650000	80900000	0,009597761	1	-0,034049745
chr11	80950000	81200000	0,048345757	1	-0,026282302
chr11	81000000	81250000	0,003037794	1	-0,026510908
chr11	81050000	81300000	4,06E-05	1	-0,026684644
chr11	81100000	81350000	1,27E-05	1	-0,026548481
chr11	81150000	81400000	9,69E-05	1	-

					0,025950851
chr11	81200000	81450000	0,004341502	1	-0,024881187
chr11	81250000	81500000	0,046081591	1	-0,023472779
chr11	81500000	81750000	0,014139245	1	-0,020843156
chr11	81550000	81800000	0,031502216	1	-0,020731507
chr11	81950000	82200000	0,003942512	1	-0,031140904
chr11	82000000	82250000	0,015388335	1	-0,032604063
chr11	83800000	84050000	0,026562926	1	-0,031658869
chr11	83850000	84100000	0,037397214	1	-0,029502784
chr11	84400000	84650000	0,00986817	1	-0,013043814
chr11	84450000	84700000	0,028674628	1	-0,013043814
chr11	84800000	85050000	0,014151704	1	-0,018898092
chr11	87000000	87250000	0,004063707	1	-0,050062674
chr11	87050000	87300000	0,000850984	1	-0,04850177
chr11	87100000	87350000	0,013769053	1	-0,046298648

chr11	87500000	87750000	0,010617529	1	-0,036161949
chr11	87550000	87800000	0,001180775	1	-0,03463133
chr11	87600000	87850000	0,000692999	1	-0,033747774
chr11	87650000	87900000	0,004624757	1	-0,033353528
chr11	87700000	87950000	0,030527579	1	-0,033162136
chr11	88000000	88250000	0,004423405	1	-0,025083109
chr11	88050000	88300000	0,003099726	1	-0,026036947
chr11	88100000	88350000	0,023630659	1	-0,027038556
chr11	88150000	88400000	0,04451629	1	-0,027777034
chr11	88200000	88450000	0,026316617	1	-0,027973122
chr11	88250000	88500000	0,004115145	1	-0,027433422
chr11	88300000	88550000	0,003699105	1	-0,026088545
chr11	92850000	93100000	0,023897104	1	-0,038186822
chr11	92900000	93150000	0,002060942	1	-0,040063621
chr11	92950000	93200000	0,001186727	1	-

					0,041485427
chr11	93000000	93250000	0,030195025	1	-0,042346402
chr11	96500000	96750000	0,036740597	1	-0,030120796
chr11	96950000	97200000	0,018680777	1	-0,015388525
chr11	97400000	97650000	0,006204141	1	-0,024075327
chr11	97450000	97700000	0,000321765	1	-0,023666228
chr11	97500000	97750000	0,001936474	1	-0,02349035
chr11	97550000	97800000	0,008918517	1	-0,023466644
chr11	97600000	97850000	0,020512461	1	-0,023466644
chr11	97650000	97900000	0,047039749	1	-0,023378827
chr11	98200000	98450000	0,006202737	1	-0,008181098
chr11	98250000	98500000	2,26E-05	1	-0,007754269
chr11	98300000	98550000	0,000212273	1	-0,007629366
chr11	98350000	98600000	0,015198742	1	-0,007616595
chr11	98750000	99000000	0,017807972	1	-0,012961093

chr11	99800000	100050000	0,02591513	1	-0,012111011
chr11	99850000	100100000	0,020077259	1	-0,013118371
chr11	100050000	100300000	0,029029308	1	-0,012203364
chr11	100800000	101050000	0,034389815	1	-0,048336702
chr11	100850000	101100000	0,005548071	1	-0,04588244
chr11	103450000	103700000	0,012230617	1	-0,027102399
chr11	103500000	103750000	0,000910121	1	-0,026476455
chr11	103550000	103800000	0,004941518	1	-0,025224083
chr11	104050000	104300000	0,038119879	1	-0,012887918
chr11	104450000	104700000	0,032148349	1	-0,0225453
chr11	105350000	105600000	0,028622714	1	-0,027766005
chr11	105800000	106050000	0,0480062	1	-0,014267145
chr11	105850000	106100000	0,008721296	1	-0,013817701
chr11	105900000	106150000	0,032167912	1	-0,012958883
chr11	106800000	107050000	0,043740342	1	-0,022686252

chr11	106850000	107100000	0,005476213	1	-0,023920335
chr11	106900000	107150000	0,001205547	1	-0,024866666
chr11	106950000	107200000	0,037853122	1	-0,025524808
chr11	107500000	107750000	0,005726326	1	-0,006616811
chr11	108100000	108350000	0,025340096	1	-0,020060802
chr11	109000000	109250000	0,014520347	1	-0,042583888
chr11	109050000	109300000	0,006526267	1	-0,042583888
chr11	110550000	110800000	0,003229869	1	-0,029963274
chr11	110600000	110850000	0,021381985	1	-0,028379572
chr11	112900000	113150000	0,004849917	1	-0,052290003
chr11	112950000	113200000	0,007951346	1	-0,054806542
chr11	113350000	113600000	0,013045045	1	-0,035435741
chr11	113400000	113650000	0,000223118	1	-0,034875328
chr11	113450000	113700000	0,000103697	1	-0,033902684

chr11	113500000	113750000	0,000248997	1	-0,033718735
chr11	113550000	113800000	0,000175789	1	-0,03397562
chr11	113600000	113850000	0,000133815	1	-0,035204812
chr11	113650000	113900000	0,000311438	1	-0,035269337
chr11	113700000	113950000	0,001104007	1	-0,035269337
chr11	113750000	114000000	0,002723245	1	-0,035269337
chr11	113800000	114050000	0,003823766	1	-0,035434217
chr11	113850000	114100000	0,003025938	1	-0,035967766
chr11	113900000	114150000	0,001459264	1	-0,036646906
chr11	113950000	114200000	0,000600331	1	-0,037347256
chr11	114000000	114250000	0,000566813	1	-0,037942309
chr11	114050000	114300000	0,002287805	1	-0,04077674
chr11	114100000	114350000	0,014082266	1	-0,042521286
chr11	114450000	114700000	0,032435599	1	-0,057025929
chr11	114500000	114750000	0,003039356	1	-0,054799564

chr11	114550000	114800000	0,033045371	1	-0,050931354
chr11	117550000	117800000	0,00907901	1	-0,023075007
chr11	117600000	117850000	0,002146352	1	-0,023075007
chr11	119600000	119850000	0,007813023	1	-0,02279519
chr11	121100000	121350000	0,03201403	1	-0,016846182
chr11	123750000	124000000	0,012290233	1	-0,047506607
chr11	123800000	124050000	0,00052614	1	-0,046513516
chr11	123850000	124100000	0,002853212	1	-0,046513516
chr11	123900000	124150000	0,036596702	1	-0,046513516
chr11	124850000	125100000	0,008959818	1	-0,013796833
chr11	124900000	125150000	0,008320013	1	-0,013361843
chr11	125250000	125500000	0,020734199	1	-0,010541167
chr11	125600000	125850000	0,003640221	1	-0,014449684
chr11	125650000	125900000	0,004818129	1	-0,015208793
chr11	125700000	125950000	0,010227777	1	-

					0,015470322
chr11	125750000	126000000	0,003376521	1	-0,015267521
chr11	125800000	126050000	0,018543831	1	-0,014798435
chr11	126550000	126800000	0,01295414	1	-0,052929402
chr11	126600000	126850000	0,022324871	1	-0,049415171
chr11	131200000	131450000	0,030518796	1	-0,078497629
chr11	131250000	131500000	0,012225332	1	-0,079239582
chr11	131300000	131550000	0,002190467	1	-0,078753951
chr11	131350000	131600000	0,000260649	1	-0,076948828
chr11	131400000	131650000	0,003741153	1	-0,074006911
chr11	131450000	131700000	0,03526952	1	-0,070300795
chr11	132250000	132500000	0,023117621	1	-0,017397492
chr11	132750000	133000000	0,012336286	1	-0,028496752
chr11	132800000	133050000	0,001616366	1	-0,029650324
chr11	132850000	133100000	7,48E-05	1	-

					0,030497941
chr11	132900000	133150000	7,31E-07	1	-0,031023399
chr11	132950000	133200000	1,27E-07	1	-0,031251259
chr11	133000000	133250000	3,83E-06	1	-0,031258102
chr11	133050000	133300000	0,000168177	1	-0,031163616
chr11	133100000	133350000	0,001827153	1	-0,031102831
chr11	133150000	133400000	0,008463286	1	-0,03118831
chr11	133200000	133450000	0,022870467	1	-0,031493337
chr11	133250000	133500000	0,04138045	1	-0,032048016
chr11	133350000	133600000	0,042896757	1	-0,033888982
chr11	133400000	133650000	0,019272028	1	-0,035105584
chr11	133450000	133700000	0,003004812	1	-0,036406036
chr11	133500000	133750000	0,001455507	1	-0,037629132
chr11	133550000	133800000	0,042555308	1	-0,038544584
chr11	134300000	134550000	0,012305835	1	-0,033868271

chr11	134350000	134600000	0,010163961	1	-0,032032151
chr12	1950000	2200000	0,021834198	1	-0,01411826
chr12	3700000	3950000	0,024151713	1	-0,045177689
chr12	3750000	4000000	0,009017452	1	-0,045177689
chr12	7750000	8000000	0,036086605	1	-0,023309983
chr12	11800000	12050000	0,037046	1	-0,013570096
chr12	11850000	12100000	0,00501288	1	-0,014459543
chr12	13750000	14000000	0,007276417	1	-0,031180876
chr12	13800000	14050000	0,042842137	1	-0,029033332
chr12	15800000	16050000	0,040502405	1	-0,014336914
chr12	15850000	16100000	0,007084744	1	-0,014669531
chr12	15900000	16150000	0,009011789	1	-0,01517754
chr12	15950000	16200000	0,026180781	1	-0,015794455
chr12	16100000	16350000	0,023478919	1	-0,016892308
chr12	16150000	16400000	0,003787593	1	-0,016806394

chr12	16200000	16450000	0,000137927	1	-0,016688075
chr12	16250000	16500000	2,59E-06	1	-0,01677649
chr12	16300000	16550000	0,000228294	1	-0,017274398
chr12	16350000	16600000	0,013290355	1	-0,01825385
chr12	16650000	16900000	0,02329657	1	-0,023115222
chr12	16700000	16950000	0,006768976	1	-0,021837776
chr12	18750000	19000000	0,011132708	1	-0,033466353
chr12	20600000	20850000	0,029572385	1	-0,026840563
chr12	20650000	20900000	0,001207356	1	-0,026097506
chr12	20700000	20950000	0,005484597	1	-0,025289588
chr12	20750000	21000000	0,017923003	1	-0,024650401
chr12	20800000	21050000	0,017271639	1	-0,024321917
chr12	20850000	21100000	0,005548538	1	-0,02433457
chr12	20900000	21150000	0,000441868	1	-0,024593972
chr12	20950000	21200000	6,56E-05	1	-0,02490439
chr12	21000000	21250000	0,000184863	1	-0,025003964

chr12	21050000	21300000	9,26E-05	1	- 0,024634433
chr12	21100000	21350000	0,001705749	1	- 0,023613953
chr12	21950000	22200000	0,01766444	1	- 0,031254344
chr12	22000000	22250000	0,000359905	1	- 0,031254344
chr12	22050000	22300000	6,11E-05	1	- 0,031254344
chr12	22100000	22350000	0,000239305	1	- 0,031254344
chr12	22150000	22400000	0,000267142	1	- 0,031254344
chr12	22200000	22450000	0,000194224	1	- 0,030939795
chr12	22250000	22500000	0,000161294	1	- 0,030636137
chr12	22300000	22550000	0,000124524	1	- 0,030391013
chr12	22350000	22600000	4,15E-05	1	- 0,030168701
chr12	22400000	22650000	1,11E-05	1	- 0,029927685
chr12	22450000	22700000	0,00030463	1	-0,02968675
chr12	22500000	22750000	0,003669746	1	- 0,029536384
chr12	22550000	22800000	0,015356352	1	-

					0,029594433
chr12	22600000	22850000	0,033956699	1	-<br>0,033779261
chr12	22650000	22900000	0,048335859	1	-<br>0,034957485
chr12	22700000	22950000	0,047374762	1	-<br>0,035978536
chr12	22750000	23000000	0,029944716	1	-<br>0,036632783
chr12	22800000	23050000	0,009357439	1	-<br>0,036687839
chr12	22850000	23100000	0,000953931	1	-<br>0,035958719
chr12	22900000	23150000	0,003062284	1	-<br>0,034368083
chr12	28400000	28650000	0,03088605	1	-<br>0,022297702
chr12	31000000	31250000	0,03273908	1	-<br>0,013742403
chr12	32050000	32300000	0,046407095	1	-<br>0,018614582
chr12	33250000	33500000	0,005234172	1	-<br>0,032373476
chr12	34250000	34500000	0,019786359	1	-<br>0,040930316
chr12	34300000	34550000	0,000705017	1	-0,04016394
chr12	34350000	34600000	0,001451135	1	-0,03895995
chr12	34400000	34650000	0,01566794	1	-

					0,037340702
chr12	38000000	38250000	0,033622684	1	-0,028696697
chr12	38050000	38300000	0,026281059	1	-0,030975402
chr12	40150000	40400000	0,013601534	1	-0,024301341
chr12	40750000	41000000	0,036338572	1	-0,024467027
chr12	40800000	41050000	0,006008232	1	-0,023370926
chr12	40850000	41100000	0,041996253	1	-0,022322009
chr12	41000000	41250000	0,011284876	1	-0,02161483
chr12	41050000	41300000	0,000573197	1	-0,02205763
chr12	41100000	41350000	0,001224809	1	-0,022539672
chr12	41150000	41400000	0,004265406	1	-0,022920237
chr12	41200000	41450000	0,004039268	1	-0,023157706
chr12	41250000	41500000	0,001612498	1	-0,023308474
chr12	41300000	41550000	0,000437541	1	-0,023485697
chr12	41350000	41600000	0,000306172	1	-0,023819273
chr12	41400000	41650000	0,00138319	1	-

					0,024424796
chr12	41450000	41700000	0,009698229	1	-0,024845058
chr12	42100000	42350000	0,00402261	1	-0,05338869
chr12	42150000	42400000	0,02151846	1	-0,055972774
chr12	44400000	44650000	0,016305204	1	-0,033585016
chr12	46000000	46250000	0,009848357	1	-0,017243281
chr12	46050000	46300000	0,014249576	1	-0,018248871
chr12	55250000	55500000	0,0242948	1	-0,031344004
chr12	61000000	61250000	0,005184393	1	-0,018634299
chr12	61050000	61300000	0,02766958	1	-0,017809398
chr12	61300000	61550000	0,042975405	1	-0,014969015
chr12	61350000	61600000	0,03400303	1	-0,014541946
chr12	61400000	61650000	0,026040845	1	-0,014213312
chr12	61450000	61700000	0,013321121	1	-0,014077025
chr12	61500000	61750000	0,002549497	1	-0,01422325
chr12	61550000	61800000	0,000758531	1	-

					0,014710178
chr12	61600000	61850000	0,018715031	1	- 0,015397668
chr12	62250000	62500000	0,006266798	1	- 0,035830858
chr12	62300000	62550000	0,004331527	1	- 0,037775659
chr12	62700000	62950000	0,014339466	1	- 0,027776859
chr12	62750000	63000000	0,007336116	1	- 0,026250423
chr12	63000000	63250000	0,007474291	1	- 0,030500638
chr12	63050000	63300000	0,015218161	1	- 0,028554353
chr12	64000000	64250000	0,007435612	1	- 0,004237271
chr12	67700000	67950000	0,01967889	1	- 0,040685683
chr12	67750000	68000000	0,002601402	1	- 0,040624667
chr12	67800000	68050000	0,000120344	1	-0,04031011
chr12	67850000	68100000	0,000251294	1	-0,04031011
chr12	67900000	68150000	0,003787574	1	-0,04031011
chr12	67950000	68200000	0,021227283	1	-0,04031011
chr12	70450000	70700000	0,016761903	1	- 0,012469724
chr12	70500000	70750000	0,010602836	1	-

					0,012469724
chr12	70550000	70800000	0,018605043	1	- 0,012469724
chr12	70600000	70850000	0,037756546	1	- 0,012469724
chr12	70650000	70900000	0,049103564	1	- 0,012733674
chr12	70700000	70950000	0,029051915	1	- 0,013100923
chr12	70750000	71000000	0,005061727	1	- 0,013583893
chr12	70800000	71050000	0,005560242	1	- 0,014110086
chr12	72550000	72800000	0,012979643	1	-0,03343699
chr12	73400000	73650000	0,032705338	1	- 0,007838593
chr12	73950000	74200000	0,04863078	1	- 0,025075188
chr12	77650000	77900000	0,021586598	1	- 0,019231254
chr12	80550000	80800000	0,004949706	1	- 0,012344407
chr12	80600000	80850000	0,000190672	1	- 0,012189285
chr12	80650000	80900000	0,000925389	1	-0,01176433
chr12	80700000	80950000	0,028881543	1	- 0,011029201
chr12	81700000	81950000	0,006363556	1	-

					0,019596618
chr12	81750000	82000000	0,017424565	1	-0,018430201
chr12	82500000	82750000	0,007051643	1	-0,025224968
chr12	82550000	82800000	0,00013818	1	-0,026502779
chr12	82600000	82850000	4,91E-06	1	-0,02711879
chr12	82650000	82900000	9,10E-05	1	-0,027261622
chr12	82700000	82950000	0,000521167	1	-0,028289493
chr12	82750000	83000000	0,000581683	1	-0,028332102
chr12	82800000	83050000	0,000167196	1	-0,027790421
chr12	82850000	83100000	0,003072889	1	-0,026547323
chr12	83250000	83500000	0,007281016	1	-0,017827721
chr12	83300000	83550000	0,00589967	1	-0,018348881
chr12	83350000	83600000	0,013780047	1	-0,018578742
chr12	83400000	83650000	0,005561502	1	-0,018466973
chr12	83450000	83700000	0,000474915	1	-0,018139793

chr12	83500000	83750000	0,000545442	1	-0,017799953
chr12	83550000	83800000	0,001922349	1	-0,01759981
chr12	83600000	83850000	0,001490318	1	-0,017544675
chr12	83650000	83900000	0,000333694	1	-0,017496598
chr12	83700000	83950000	0,000114038	1	-0,017247647
chr12	83750000	84000000	0,001816402	1	-0,016641685
chr12	83800000	84050000	0,029153563	1	-0,015661825
chr12	84100000	84350000	0,0151842	1	-0,012830084
chr12	84150000	84400000	0,021224358	1	-0,012420277
chr12	84250000	84500000	0,048103042	1	-0,012200238
chr12	84300000	84550000	0,010022281	1	-0,012768316
chr12	85300000	85550000	0,018904505	1	-0,028201168
chr12	87800000	88050000	0,010609389	1	-0,015083207
chr12	93300000	93550000	0,019819807	1	-0,013253437
chr12	96850000	97100000	0,027509566	1	-

					0,038613551
chr12	96900000	97150000	0,007596664	1	-0,038865731
chr12	96950000	97200000	0,001016234	1	-0,038729097
chr12	97000000	97250000	5,50E-05	1	-0,038240443
chr12	97050000	97300000	0,000307209	1	-0,037475348
chr12	97100000	97350000	0,002901091	1	-0,03651469
chr12	97150000	97400000	0,010427906	1	-0,035422804
chr12	97200000	97450000	0,023263244	1	-0,034225308
chr12	97250000	97500000	0,04164318	1	-0,032912007
chr12	97700000	97950000	0,008174613	1	-0,023555776
chr12	97750000	98000000	0,011869039	1	-0,023555776
chr12	98150000	98400000	0,035068496	1	-0,035174954
chr12	98200000	98450000	0,01628365	1	-0,037657398
chr12	101000000	101250000	0,020301887	1	-0,012078848
chr12	101050000	101300000	0,007578554	1	-0,011383329

chr12	103600000	103850000	0,038766323	1	- 0,021086998
chr12	103650000	103900000	0,007317482	1	- 0,022163763
chr12	107000000	107250000	0,003544033	1	- 0,016856429
chr12	107050000	107300000	6,21E-05	1	- 0,017591065
chr12	107100000	107350000	0,000150085	1	- 0,017940023
chr12	107150000	107400000	0,000807565	1	- 0,017203844
chr12	107200000	107450000	0,001678169	1	- 0,016935396
chr12	107250000	107500000	0,001878685	1	- 0,016736145
chr12	107300000	107550000	0,000937194	1	-0,01669955
chr12	107350000	107600000	0,000135897	1	- 0,016911206
chr12	107400000	107650000	0,000472258	1	- 0,017389626
chr12	107450000	107700000	0,007680086	1	- 0,018041652
chr12	107500000	107750000	0,027830615	1	- 0,018651904
chr12	107550000	107800000	0,029750534	1	- 0,018927895
chr12	107600000	107850000	0,007997212	1	-

					0,018588021
chr12	107650000	107900000	0,006736202	1	- 0,017458531
chr12	114650000	114900000	0,049112149	1	- 0,040336812
chr12	114700000	114950000	0,004234799	1	- 0,041148393
chr12	114750000	115000000	0,003949135	1	- 0,042955717
chr12	115200000	115450000	0,002367254	1	- 0,026213207
chr12	115250000	115500000	0,006964314	1	- 0,027041653
chr12	115300000	115550000	0,017711953	1	- 0,027573839
chr12	115350000	115600000	0,009603713	1	- 0,027571269
chr12	115400000	115650000	0,001234491	1	- 0,026946051
chr12	115450000	115700000	0,003495592	1	- 0,025791343
chr12	115500000	115750000	0,043877701	1	- 0,024365712
chr12	115700000	115950000	0,023780344	1	- 0,022115055
chr12	115750000	116000000	0,003307692	1	- 0,022115055
chr12	115800000	116050000	0,024704865	1	-

					0,022115055
chr12	115900000	116150000	0,042606435	1	- 0,022319624
chr12	115950000	116200000	0,006570746	1	- 0,023265679
chr12	116000000	116250000	0,010642498	1	- 0,024337517
chr12	116350000	116600000	0,01865056	1	- 0,017570292
chr12	116400000	116650000	0,002363971	1	- 0,016561412
chr12	116450000	116700000	0,019479777	1	- 0,016117546
chr12	118000000	118250000	0,036393128	1	- 0,011672723
chr12	118050000	118300000	0,004398841	1	- 0,012269739
chr12	119100000	119350000	0,004435338	1	- 0,053256532
chr12	119150000	119400000	8,56E-05	1	- 0,053013902
chr12	119200000	119450000	1,64E-06	1	- 0,052836635
chr12	119250000	119500000	3,05E-06	1	- 0,052836635
chr12	119300000	119550000	8,12E-07	1	- 0,052836635
chr12	119350000	119600000	1,01E-06	1	-

					0,052836635
chr12	119400000	119650000	6,73E-06	1	-0,052836635
chr12	119450000	119700000	5,77E-06	1	-0,052827775
chr12	119500000	119750000	1,03E-05	1	-0,053000976
chr12	119550000	119800000	0,000984882	1	-0,05328188
chr12	119600000	119850000	0,021622565	1	-0,053506909
chr12	124550000	124800000	0,046041608	1	-0,019672626
chr12	124600000	124850000	0,002949913	1	-0,019672626
chr12	124650000	124900000	0,025666524	1	-0,02052643
chr12	125950000	126200000	0,009365395	1	-0,046116094
chr12	127950000	128200000	0,002859063	1	-0,025316115
chr12	128000000	128250000	0,000360703	1	-0,024939257
chr12	128050000	128300000	0,00091772	1	-0,0248211
chr12	128100000	128350000	0,000323138	1	-0,025025589
chr12	128150000	128400000	0,000100595	1	-0,025388991
chr12	128200000	128450000	0,001096689	1	-0,025458853

chr12	128250000	128500000	0,003867877	1	- 0,025458853
chr12	128300000	128550000	0,003705734	1	- 0,025458853
chr12	128350000	128600000	0,000934802	1	- 0,025558508
chr12	128400000	128650000	0,000175522	1	-0,02605203
chr12	128450000	128700000	0,003377275	1	- 0,026524566
chr12	128500000	128750000	0,032623261	1	- 0,026734544
chr12	128800000	129050000	0,005274527	1	- 0,021105163
chr12	129550000	129800000	0,047059649	1	- 0,055687521
chr12	129600000	129850000	0,041757283	1	- 0,055687521
chr12	129650000	129900000	0,040643287	1	- 0,055687521
chr12	129700000	129950000	0,036951168	1	- 0,055687521
chr12	129750000	130000000	0,028860727	1	- 0,057520716
chr12	129800000	130050000	0,019267595	1	- 0,059435943
chr12	129850000	130100000	0,01161838	1	- 0,061335683
chr12	129900000	130150000	0,006571249	1	-

					0,063104801
chr12	129950000	130200000	0,003152269	1	-0,06468399
chr12	130000000	130250000	0,000873706	1	- 0,066080564
chr12	130050000	130300000	9,15E-05	1	- 0,067324344
chr12	130100000	130350000	0,000795398	1	- 0,068377014
chr12	130150000	130400000	0,017592435	1	- 0,069068918
chr12	130600000	130850000	0,006329805	1	- 0,038292444
chr12	130650000	130900000	0,000117423	1	- 0,038680531
chr12	130700000	130950000	0,000101294	1	-0,03855239
chr12	130750000	131000000	0,000115382	1	- 0,037709722
chr12	130800000	131050000	0,003329294	1	- 0,036211197
chr12	130850000	131100000	0,036856974	1	- 0,036211197
chr12	131100000	131350000	0,047055417	1	- 0,032353358
chr12	131150000	131400000	0,004442438	1	- 0,030645724
chr12	131200000	131450000	0,000685064	1	- 0,029398234
chr12	131250000	131500000	0,010687587	1	-

					0,028703665
chr12	131550000	131800000	0,017714145	1	-0,037909885
chr12	131600000	131850000	0,001341219	1	-0,037671873
chr12	131650000	131900000	0,006005625	1	-0,037671873
chr12	132650000	132900000	0,003488101	1	-0,025790852
chr12	132700000	132950000	0,034243533	1	-0,025790852
chr12	133100000	133350000	0,011969623	1	-0,015248617
chr12	133150000	133400000	0,02192991	1	-0,015963688
chr12	133300000	133550000	0,010297765	1	-0,016493021
chr12	133350000	133600000	0,000658553	1	-0,01611702
chr12	133400000	133650000	0,002207497	1	-0,015680908
chr12	133450000	133700000	0,009558693	1	-0,015304338
chr12	133500000	133750000	0,012516534	1	-0,015042708
chr12	133550000	133800000	0,007020502	1	-0,014891246
chr12	133600000	133850000	0,001944829	1	-0,014816713

chr12	133650000	133851895	0,000274888	1	-0,014788353
chr13	19000000	19250000	0,016283313	1	-0,005628549
chr13	19050000	19300000	0,003382344	1	-0,006078712
chr13	19100000	19350000	0,004932481	1	-0,006245627
chr13	19150000	19400000	0,008947373	1	-0,006176419
chr13	19850000	20100000	0,012294562	1	-0,040288478
chr13	22300000	22550000	0,011378032	1	-0,029774721
chr13	24650000	24900000	0,003874978	1	-0,020240923
chr13	24700000	24950000	0,002357396	1	-0,021068164
chr13	24750000	25000000	0,017922977	1	-0,021457657
chr13	25000000	25250000	0,017408811	1	-0,025979397
chr13	25050000	25300000	0,001565981	1	-0,025117876
chr13	25100000	25350000	0,016581506	1	-0,023679568
chr13	25600000	25850000	0,03097475	1	-0,011269363

chr13	25650000	25900000	0,000996057	1	-0,011396673
chr13	25700000	25950000	0,000403916	1	-0,011705206
chr13	25750000	26000000	0,01198858	1	-0,012283208
chr13	26150000	26400000	0,007293124	1	-0,018105376
chr13	26200000	26450000	0,000301596	1	-0,018105376
chr13	26250000	26500000	0,001254786	1	-0,018105376
chr13	26300000	26550000	0,003078029	1	-0,018105376
chr13	26350000	26600000	0,003261925	1	-0,018105376
chr13	26400000	26650000	0,003636893	1	-0,017768685
chr13	26450000	26700000	0,007368099	1	-0,017457742
chr13	26500000	26750000	0,017852615	1	-0,017197998
chr13	26550000	26800000	0,026822127	1	-0,016926929
chr13	26600000	26850000	0,013929143	1	-0,01655632
chr13	26650000	26900000	0,003669746	1	-0,016057316
chr13	27100000	27350000	0,039596925	1	-

					0,030009104
chr13	27150000	27400000	0,038432779	1	-0,032566431
chr13	28450000	28700000	0,035078523	1	-0,025678763
chr13	28500000	28750000	0,006969817	1	-0,026768274
chr13	28550000	28800000	0,000710128	1	-0,027976365
chr13	28600000	28850000	0,007902173	1	-0,026656053
chr13	28900000	29150000	0,045036513	1	-0,020789781
chr13	28950000	29200000	0,002463831	1	-0,021212162
chr13	29000000	29250000	0,001288134	1	-0,02182551
chr13	29050000	29300000	0,009514641	1	-0,022481116
chr13	29100000	29350000	0,019013182	1	-0,023034992
chr13	29150000	29400000	0,014169671	1	-0,023320782
chr13	29200000	29450000	0,003375536	1	-0,023134394
chr13	29250000	29500000	0,0010694	1	-0,022251118
chr13	29300000	29550000	0,04360699	1	-0,020498454

chr13	31350000	31600000	0,018519637	1	-0,021039187
chr13	31400000	31650000	0,04057932	1	-0,019730691
chr13	31650000	31900000	0,010597209	1	-0,016984217
chr13	31700000	31950000	0,001618363	1	-0,016672636
chr13	31750000	32000000	0,003858733	1	-0,016094904
chr13	31800000	32050000	0,024985164	1	-0,015323646
chr13	33550000	33800000	0,032830158	1	-0,012509097
chr13	35700000	35950000	0,020195179	1	-0,031170322
chr13	37150000	37400000	0,003439022	1	-0,020483343
chr13	37200000	37450000	0,001911512	1	-0,021582558
chr13	37250000	37500000	0,019208406	1	-0,021945938
chr13	40500000	40750000	0,005763467	1	-0,057098427
chr13	40550000	40800000	0,030508983	1	-0,060212281
chr13	44750000	45000000	0,003209771	1	-0,017390309

chr13	44800000	45050000	0,036919804	1	-0,016166138
chr13	47250000	47500000	0,022438989	1	-0,012552847
chr13	47500000	47750000	0,044937744	1	-0,010486046
chr13	47550000	47800000	0,005216776	1	-0,010960654
chr13	47600000	47850000	0,038283224	1	-0,010960654
chr13	48150000	48400000	0,036814816	1	-0,019487858
chr13	48200000	48450000	0,027253017	1	-0,02101281
chr13	50750000	51000000	0,02301676	1	-0,02964109
chr13	50800000	51050000	0,001927833	1	-0,02956273
chr13	50850000	51100000	8,51E-05	1	-0,029114569
chr13	50900000	51150000	0,001080373	1	-0,028208933
chr13	50950000	51200000	0,018786355	1	-0,02678878
chr13	51450000	51700000	0,016774913	1	-0,013990913
chr13	51700000	51950000	0,008068054	1	-0,015359843
chr13	51750000	52000000	0,003127753	1	-0,016173491
chr13	51800000	52050000	0,033209069	1	-0,016658108

chr13	51950000	52200000	0,034104169	1	-0,015652185
chr13	52000000	52250000	0,003309239	1	-0,014883718
chr13	52050000	52300000	0,016080987	1	-0,015390851
chr13	52350000	52600000	0,017760122	1	-0,019143303
chr13	52400000	52650000	0,00103214	1	-0,018794162
chr13	52450000	52700000	0,001294393	1	-0,018794162
chr13	52500000	52750000	0,016011741	1	-0,018794162
chr13	52750000	53000000	0,042151601	1	-0,014868086
chr13	52800000	53050000	0,010602769	1	-0,015870231
chr13	53250000	53500000	0,011781481	1	-0,028153561
chr13	54500000	54750000	0,017511531	1	-0,016666066
chr13	56600000	56850000	0,010372387	1	-0,010697827
chr13	56650000	56900000	0,028628677	1	-0,01101067
chr13	56700000	56950000	0,031055478	1	-0,011069201
chr13	56750000	57000000	0,005914663	1	-

					0,010909321
chr13	56800000	57050000	0,000491118	1	-0,010678992
chr13	56850000	57100000	0,001244184	1	-0,010540327
chr13	56900000	57150000	0,001269359	1	-0,010540327
chr13	56950000	57200000	0,000301337	1	-0,010540327
chr13	57000000	57250000	0,001780978	1	-0,010540327
chr13	57050000	57300000	0,010817582	1	-0,010540327
chr13	57100000	57350000	0,019464497	1	-0,010591476
chr13	57150000	57400000	0,016542796	1	-0,010823252
chr13	57200000	57450000	0,009947629	1	-0,011149382
chr13	57250000	57500000	0,006010134	1	-0,011473288
chr13	57300000	57550000	0,00353797	1	-0,011745764
chr13	57350000	57600000	0,001067375	1	-0,011976826
chr13	57400000	57650000	0,000291396	1	-0,012200311
chr13	57450000	57700000	0,010317479	1	-0,01241755

chr13	57800000	58050000	0,016757298	1	-0,008238784
chr13	57850000	58100000	0,019286743	1	-0,008632532
chr13	58000000	58250000	0,025048461	1	-0,009190471
chr13	58050000	58300000	0,002930178	1	-0,009157993
chr13	58100000	58350000	7,96E-05	1	-0,009070552
chr13	58150000	58400000	0,000104753	1	-0,008935085
chr13	58200000	58450000	0,001360425	1	-0,008722464
chr13	58250000	58500000	0,009217008	1	-0,008392988
chr13	59800000	60050000	0,011073828	1	-0,032466185
chr13	60100000	60350000	0,019902959	1	-0,027643214
chr13	60150000	60400000	0,002652344	1	-0,025731679
chr13	60200000	60450000	0,004105662	1	-0,024883821
chr13	60250000	60500000	0,003451648	1	-0,025074979
chr13	61100000	61350000	0,029287624	1	-0,037930449

chr13	61150000	61400000	0,013293877	1	-0,035612104
chr13	62750000	63000000	0,025064748	1	-0,02068084
chr13	62800000	63050000	0,005812627	1	-0,021730669
chr13	62850000	63100000	0,002840036	1	-0,022411917
chr13	62900000	63150000	0,003359426	1	-0,022848152
chr13	62950000	63200000	0,005654265	1	-0,023182035
chr13	63000000	63250000	0,009083512	1	-0,02352762
chr13	63050000	63300000	0,012376568	1	-0,023945492
chr13	63100000	63350000	0,014160833	1	-0,024450566
chr13	63150000	63400000	0,013451331	1	-0,025027547
chr13	63200000	63450000	0,009809963	1	-0,025652043
chr13	63250000	63500000	0,004406408	1	-0,026302521
chr13	63300000	63550000	0,000753373	1	-0,026952023
chr13	63350000	63600000	0,000218419	1	-0,027550506
chr13	63400000	63650000	0,006382936	1	-0,028012714

chr13	63900000	64150000	0,007672686	1	-0,015560013
chr13	64150000	64400000	0,010458397	1	-0,016793439
chr13	64200000	64450000	0,0208508	1	-0,017691063
chr13	65200000	65450000	0,001881431	1	-0,010691313
chr13	65250000	65500000	3,43E-06	1	-0,010765902
chr13	65300000	65550000	2,61E-05	1	-0,0108901
chr13	65350000	65600000	0,000305443	1	-0,011058579
chr13	65400000	65650000	0,001866833	1	-0,011320341
chr13	65450000	65700000	0,010534699	1	-0,011772658
chr13	65950000	66200000	0,021281534	1	-0,020082012
chr13	66000000	66250000	0,013387184	1	-0,020082012
chr13	66050000	66300000	0,027333376	1	-0,020356094
chr13	66350000	66600000	0,042394086	1	-0,025521191
chr13	66400000	66650000	0,004445647	1	-0,026902825
chr13	66450000	66700000	0,001384006	1	-

					0,028039954
chr13	66500000	66750000	0,031077823	1	-0,028753882
chr13	67950000	68200000	0,007289496	1	-0,044714667
chr13	68000000	68250000	0,02808585	1	-0,041953076
chr13	68500000	68750000	0,039972298	1	-0,022749829
chr13	68550000	68800000	0,003144512	1	-0,022946028
chr13	68600000	68850000	7,75E-05	1	-0,023192268
chr13	68650000	68900000	5,10E-05	1	-0,023221496
chr13	68700000	68950000	5,42E-05	1	-0,02280708
chr13	68750000	69000000	0,002503249	1	-0,021839056
chr13	69250000	69500000	0,003580309	1	-0,011974293
chr13	69300000	69550000	6,18E-05	1	-0,011890136
chr13	69350000	69600000	0,000303041	1	-0,011572469
chr13	69400000	69650000	0,012305835	1	-0,010944601
chr13	72950000	73200000	0,025021977	1	-0,037375728

chr13	73000000	73250000	0,029283979	1	-0,040196907
chr13	74950000	75200000	0,020909906	1	-0,031133951
chr13	75000000	75250000	0,000719776	1	-0,030600642
chr13	75050000	75300000	0,000590222	1	-0,029987893
chr13	75100000	75350000	0,002630723	1	-0,029509203
chr13	75150000	75400000	0,002868353	1	-0,029509203
chr13	75200000	75450000	0,001040287	1	-0,029509203
chr13	75250000	75500000	0,000159583	1	-0,029509203
chr13	75300000	75550000	9,28E-05	1	-0,029509203
chr13	75350000	75600000	0,001807778	1	-0,029262028
chr13	75400000	75650000	0,028850785	1	-0,029185538
chr13	76450000	76700000	0,007915377	1	-0,100098499
chr13	76500000	76750000	0,00318493	1	-0,095530283
chr13	79250000	79500000	0,007836677	1	-0,03960632
chr13	81150000	81400000	0,014313134	1	-

					0,019662145
chr13	82750000	83000000	0,021476483	1	-0,022844712
chr13	82800000	83050000	0,004090813	1	-0,02379086
chr13	82850000	83100000	0,00029113	1	-0,024591234
chr13	82900000	83150000	5,03E-06	1	-0,025146155
chr13	82950000	83200000	1,34E-05	1	-0,025414737
chr13	83000000	83250000	0,000628864	1	-0,02545149
chr13	83050000	83300000	0,006170826	1	-0,02540389
chr13	83100000	83350000	0,019310906	1	-0,025468982
chr13	83150000	83400000	0,022798663	1	-0,025808622
chr13	83200000	83450000	0,009113756	1	-0,027659911
chr13	83250000	83500000	0,00107937	1	-0,028714496
chr13	83300000	83550000	0,004770185	1	-0,028714496
chr13	86600000	86850000	0,014320875	1	-0,020821722
chr13	86650000	86900000	0,010182737	1	-0,021789143
chr13	87000000	87250000	0,013549486	1	-0,026570196

chr13	87050000	87300000	0,002717535	1	-0,027795172
chr13	87100000	87350000	0,002277019	1	-0,028552686
chr13	87150000	87400000	0,005603931	1	-0,028960603
chr13	87200000	87450000	0,010327733	1	-0,029267816
chr13	87250000	87500000	0,008289756	1	-0,02971261
chr13	87300000	87550000	0,001930758	1	-0,030397415
chr13	87350000	87600000	0,000633926	1	-0,031224377
chr13	87400000	87650000	0,011376534	1	-0,031933721
chr13	87650000	87900000	0,013422652	1	-0,02786706
chr13	87700000	87950000	0,000640477	1	-0,026237999
chr13	87750000	88000000	0,001606989	1	-0,026862185
chr13	87800000	88050000	0,00548169	1	-0,027338437
chr13	87850000	88100000	0,004406837	1	-0,027511055
chr13	87900000	88150000	0,000862181	1	-0,027283602
chr13	87950000	88200000	0,000249457	1	-0,026602284

chr13	88000000	88250000	0,00646107	1	-0,026602284
chr13	88350000	88600000	0,036276399	1	-0,019158797
chr13	88700000	88950000	0,005365472	1	-0,025194636
chr13	88750000	89000000	0,036635485	1	-0,02372459
chr13	89100000	89350000	0,038072846	1	-0,020054115
chr13	89150000	89400000	0,023041137	1	-0,018908475
chr13	89200000	89450000	0,015369045	1	-0,01689635
chr13	89250000	89500000	0,012664715	1	-0,016476732
chr13	89300000	89550000	0,016732066	1	-0,0159303
chr13	89350000	89600000	0,038925974	1	-0,015182991
chr13	91850000	92100000	0,031511619	1	-0,018360649
chr13	93300000	93550000	0,034667618	1	-0,020173401
chr13	93350000	93600000	0,015925076	1	-0,022002724
chr13	96000000	96250000	0,020495357	1	-0,019313153
chr13	96900000	97150000	0,026460809	1	-0,026106994
chr13	96950000	97200000	0,002302442	1	-

					0,026106994
chr13	97000000	97250000	0,010759051	1	-0,026106994
chr13	101000000	101250000	0,02967468	1	-0,01720781
chr13	102800000	103050000	0,042227871	1	-0,0263253
chr13	105350000	105600000	0,007985653	1	-0,024795458
chr13	105400000	105650000	0,00116082	1	-0,025842562
chr13	105450000	105700000	0,001371098	1	-0,026464178
chr13	105500000	105750000	0,009992089	1	-0,026764932
chr13	105850000	106100000	0,004958193	1	-0,034818466
chr13	105900000	106150000	0,003067517	1	-0,036449944
chr13	105950000	106200000	0,047021523	1	-0,037407267
chr13	107300000	107550000	0,021344764	1	-0,030774065
chr13	107350000	107600000	0,010289864	1	-0,029411996
chr13	107550000	107800000	0,025759064	1	-0,026346993
chr13	107600000	107850000	0,004794206	1	-0,026281473
chr13	107650000	107900000	0,000312754	1	-0,0264589

chr13	107700000	107950000	6,59E-05	1	-0,026929893
chr13	107750000	108000000	0,001868943	1	-0,027453485
chr13	107800000	108050000	0,017243169	1	-0,027453485
chr13	108300000	108550000	0,005964519	1	-0,042279764
chr13	108350000	108600000	0,002870867	1	-0,044198498
chr13	109100000	109350000	0,039065398	1	-0,018297377
chr13	112650000	112900000	0,00991518	1	-0,044562673
chr13	112700000	112950000	0,012027992	1	-0,047169535
chr14	27650000	27900000	0,024856566	1	-0,020588269
chr14	27700000	27950000	0,014705599	1	-0,020588269
chr14	27850000	28100000	0,033055983	1	-0,0197936
chr14	27900000	28150000	0,00337947	1	-0,018881574
chr14	27950000	28200000	0,000591657	1	-0,018147378
chr14	28000000	28250000	0,008940378	1	-0,017742097
chr14	28400000	28650000	0,03383704	1	-

					0,024090702
chr14	28450000	28700000	0,003014032	1	- 0,025362492
chr14	28500000	28750000	0,003476188	1	- 0,026375557
chr14	29200000	29450000	0,032893397	1	- 0,029329674
chr14	29250000	29500000	0,005066358	1	- 0,027918762
chr14	29950000	30200000	0,024744709	1	- 0,012672796
chr14	30000000	30250000	0,018005753	1	- 0,012833447
chr14	30500000	30750000	0,009739115	1	- 0,025685415
chr14	30550000	30800000	0,000761686	1	- 0,027189499
chr14	30600000	30850000	0,004471441	1	- 0,027968127
chr14	30650000	30900000	0,009476377	1	- 0,028031967
chr14	30700000	30950000	0,006260975	1	- 0,027563516
chr14	30750000	31000000	0,001848509	1	- 0,026855117
chr14	30800000	31050000	0,000836053	1	- 0,026196209
chr14	30850000	31100000	0,006223086	1	-

					0,025762362
chr14	33350000	33600000	0,006546702	1	-0,02806702
chr14	33600000	33850000	0,035733978	1	-0,023569748
chr14	33650000	33900000	0,002217529	1	-0,023569748
chr14	33700000	33950000	0,00350605	1	-0,023569748
chr14	33750000	34000000	0,018832417	1	-0,023569748
chr14	33800000	34050000	0,021618153	1	-0,023718833
chr14	33850000	34100000	0,005484597	1	-0,024344869
chr14	33900000	34150000	0,004212677	1	-0,025210969
chr14	34500000	34750000	0,036171472	1	-0,016587897
chr14	36550000	36800000	0,015702429	1	-0,028373349
chr14	36600000	36850000	0,024553146	1	-0,026538895
chr14	37000000	37250000	0,024283578	1	-0,017205943
chr14	37050000	37300000	0,023029732	1	-0,016693048
chr14	37500000	37750000	0,048533044	1	-0,008976183

chr14	37550000	37800000	0,007367773	1	-0,008521931
chr14	37600000	37850000	0,008658935	1	-0,008369296
chr14	37650000	37900000	0,010016245	1	-0,007813394
chr14	40150000	40400000	0,015418964	1	-0,034615227
chr14	41100000	41350000	0,010331148	1	-0,004972007
chr14	41150000	41400000	0,01620051	1	-0,005291162
chr14	41350000	41600000	0,040566113	1	-0,004458648
chr14	41400000	41650000	0,038646896	1	-0,004906373
chr14	41900000	42150000	0,036889081	1	-0,015540648
chr14	44150000	44400000	0,029646872	1	-0,005659097
chr14	44200000	44450000	0,000333017	1	-0,005219109
chr14	44250000	44500000	0,011579393	1	-0,005069811
chr14	44900000	45150000	0,018270385	1	-0,034920142
chr14	45400000	45650000	0,04891363	1	-0,015507398

chr14	45900000	46150000	0,031738122	1	-0,036045901
chr14	46350000	46600000	0,001688319	1	-0,020553514
chr14	46400000	46650000	0,000818899	1	-0,020820514
chr14	46450000	46700000	0,001347222	1	-0,020913371
chr14	46500000	46750000	0,000375168	1	-0,0209043
chr14	46550000	46800000	1,97E-05	1	-0,020895752
chr14	46600000	46850000	9,20E-08	1	-0,020917335
chr14	46650000	46900000	4,62E-09	1	-0,020869825
chr14	46700000	46950000	1,96E-05	1	-0,020559272
chr14	46750000	47000000	0,001946567	1	-0,019790996
chr14	46800000	47050000	0,040772345	1	-0,018476253
chr14	47200000	47450000	0,03593318	1	-0,009985386
chr14	47250000	47500000	0,018432001	1	-0,009985386
chr14	47700000	47950000	0,010510824	1	-0,016130088
chr14	47750000	48000000	0,001407231	1	-

					0,015556608
chr14	47800000	48050000	0,0224281	1	-0,014824895
chr14	48850000	49100000	0,030307582	1	-0,013992786
chr14	56550000	56800000	0,027471585	1	-0,026736384
chr14	56600000	56850000	0,009033826	1	-0,025359018
chr14	56850000	57100000	0,030561166	1	-0,021552506
chr14	56900000	57150000	0,002359446	1	-0,022339709
chr14	56950000	57200000	0,01638637	1	-0,02345556
chr14	57150000	57400000	0,03444655	1	-0,026051649
chr14	57200000	57450000	0,003685451	1	-0,026051649
chr14	57250000	57500000	0,032984848	1	-0,026051649
chr14	60550000	60800000	0,049952619	1	-0,016858752
chr14	60600000	60850000	0,004089778	1	-0,018581803
chr14	61500000	61750000	0,040443744	1	-0,012070114
chr14	63150000	63400000	0,032414565	1	-0,010613727

chr14	63200000	63450000	0,030602929	1	- 0,010578488
chr14	63250000	63500000	0,044913539	1	- 0,010578488
chr14	63300000	63550000	0,010969748	1	- 0,010578488
chr14	63350000	63600000	0,049646897	1	- 0,010578488
chr14	63600000	63850000	0,047023134	1	- 0,012736495
chr14	63650000	63900000	0,035347797	1	-0,01376567
chr14	65850000	66100000	0,032402325	1	- 0,021731371
chr14	66250000	66500000	0,008763664	1	-0,01322837
chr14	66300000	66550000	0,012336586	1	-0,01322837
chr14	66350000	66600000	0,010154762	1	-0,01322837
chr14	71800000	72050000	0,030263352	1	- 0,043085214
chr14	71850000	72100000	0,0023219	1	- 0,041478269
chr14	71900000	72150000	0,022657596	1	-0,03939145
chr14	72250000	72500000	0,014120823	1	- 0,030287843
chr14	72300000	72550000	0,002657388	1	-0,02999174
chr14	72350000	72600000	0,001209855	1	-0,02999174
chr14	72400000	72650000	0,005404883	1	-0,02999174
chr14	76550000	76800000	0,039932228	1	-0,01359445
chr14	78650000	78900000	0,041176904	1	-

					0,041311901
chr14	78700000	78950000	0,001249388	1	- 0,041311901
chr14	78750000	79000000	0,003137767	1	- 0,041311901
chr14	78800000	79050000	0,037851604	1	- 0,041311901
chr14	79950000	80200000	0,043379117	1	- 0,044080947
chr14	81700000	81950000	0,045691562	1	- 0,033999836
chr14	81750000	82000000	0,030789444	1	- 0,035209102
chr14	81800000	82050000	0,047423946	1	- 0,036858656
chr14	82050000	82300000	0,017967585	1	-0,04245625
chr14	82100000	82350000	0,003304994	1	- 0,040517521
chr14	86650000	86900000	0,030717318	1	- 0,013011011
chr14	86700000	86950000	0,001343767	1	- 0,012644768
chr14	86750000	87000000	0,006232756	1	- 0,012158543
chr14	86800000	87050000	0,034972327	1	- 0,011672941
chr14	86950000	87200000	0,0273864	1	- 0,010861949

chr14	87000000	87250000	0,008221026	1	-0,010732622
chr14	87050000	87300000	0,002280004	1	-0,010605519
chr14	87100000	87350000	0,000757564	1	-0,010535927
chr14	87150000	87400000	0,000127141	1	-0,010681105
chr14	87200000	87450000	0,003105221	1	-0,011245988
chr14	87700000	87950000	0,019946459	1	-0,022374559
chr14	87750000	88000000	0,002730422	1	-0,023517
chr14	87800000	88050000	0,000381638	1	-0,024218846
chr14	87850000	88100000	4,53E-05	1	-0,024630802
chr14	87900000	88150000	2,57E-06	1	-0,024891792
chr14	87950000	88200000	1,78E-06	1	-0,025070788
chr14	88000000	88250000	3,85E-06	1	-0,025165939
chr14	88050000	88300000	5,26E-06	1	-0,025152997
chr14	88100000	88350000	0,000430781	1	-0,025043657
chr14	88150000	88400000	0,007669267	1	-

					0,024916517
chr14	88200000	88450000	0,036583994	1	-0,024916547
chr14	88300000	88550000	0,041598947	1	-0,025873596
chr14	88350000	88600000	0,007270562	1	-0,026898781
chr14	88400000	88650000	0,02281097	1	-0,028066776
chr14	89300000	89550000	0,003283101	1	-0,020988947
chr14	89350000	89600000	0,02730885	1	-0,019925974
chr14	90550000	90800000	0,002978889	1	-0,023052794
chr14	90600000	90850000	0,011185272	1	-0,024123878
chr14	90650000	90900000	0,017304549	1	-0,024248098
chr14	90700000	90950000	0,004599178	1	-0,023706199
chr14	90750000	91000000	0,002128956	1	-0,022900267
chr14	90800000	91050000	0,025560329	1	-0,024548498
chr14	90950000	91200000	0,017990605	1	-0,025497376
chr14	91000000	91250000	0,006101469	1	-

					0,024066656
chr14	92050000	92300000	0,020042717	1	-0,021609579
chr14	92700000	92950000	0,028386707	1	-0,022215471
chr14	92750000	93000000	0,000551222	1	-0,021921931
chr14	92800000	93050000	0,000149392	1	-0,021921931
chr14	92850000	93100000	0,000187104	1	-0,021921931
chr14	92900000	93150000	2,74E-05	1	-0,021921931
chr14	92950000	93200000	6,78E-07	1	-0,021921931
chr14	93000000	93250000	0,00010379	1	-0,021768927
chr14	93050000	93300000	0,010286984	1	-0,02181279
chr14	93850000	94100000	0,0335942	1	-0,013281463
chr14	93900000	94150000	0,010562358	1	-0,013373686
chr14	93950000	94200000	0,001495265	1	-0,013109776
chr14	94000000	94250000	0,004861325	1	-0,013109776
chr14	95200000	95450000	0,010730851	1	-0,032910337

chr14	95250000	95500000	0,027515434	1	-0,034720387
chr14	96800000	97050000	0,011791957	1	-0,014179996
chr14	96850000	97100000	0,011513464	1	-0,01345917
chr14	97000000	97250000	0,026368237	1	-0,014294773
chr14	97450000	97700000	0,046017279	1	-0,03708074
chr14	97500000	97750000	0,003565038	1	-0,035575922
chr14	97550000	97800000	0,03197503	1	-0,033734358
chr14	98400000	98650000	0,025618372	1	-0,013194724
chr14	98450000	98700000	0,001372952	1	-0,013267067
chr14	98500000	98750000	8,77E-06	1	-0,013267067
chr14	98550000	98800000	9,83E-05	1	-0,013267067
chr14	98600000	98850000	0,005500349	1	-0,013267067
chr14	99400000	99650000	0,030770895	1	-0,046208852
chr14	101300000	101550000	0,012047154	1	-0,025060172
chr14	101350000	101600000	0,000947597	1	-0,025116315

chr14	101400000	101650000	5,70E-05	1	-<br>0,025259126
chr14	101450000	101700000	5,64E-05	1	-<br>0,025259126
chr14	101500000	101750000	0,000427662	1	-<br>0,025259126
chr14	101550000	101800000	0,001677299	1	-<br>0,025259126
chr14	101600000	101850000	0,002169202	1	-<br>0,025259126
chr14	101650000	101900000	0,000632354	1	-<br>0,025570401
chr14	101700000	101950000	0,002059038	1	-<br>0,026034522
chr14	104650000	104900000	0,04306802	1	-<br>0,017139204
chr14	105950000	106200000	0,023161385	1	-0,01981899
chr14	106000000	106250000	0,00490381	1	-<br>0,018858959
chr14	106250000	106500000	0,049485656	1	-<br>0,016062469
chr14	106300000	106550000	0,03037914	1	-<br>0,017347497
chr14	106600000	106850000	0,006171174	1	-<br>0,020525659
chr15	22500000	22750000	0,031437569	1	-<br>0,035823007
chr15	25250000	25500000	0,04959305	1	-

					0,037894539
chr15	25300000	25550000	0,004641456	1	-0,040197273
chr15	25350000	25600000	0,000329762	1	-0,041928225
chr15	25400000	25650000	0,003184778	1	-0,040499042
chr15	25450000	25700000	0,017916348	1	-0,039055486
chr15	25500000	25750000	0,0426051	1	-0,037594475
chr15	25950000	26200000	0,037696667	1	-0,027052958
chr15	26000000	26250000	0,02927321	1	-0,026261448
chr15	26050000	26300000	0,029865422	1	-0,025403038
chr15	26100000	26350000	0,039239819	1	-0,024470001
chr15	26400000	26650000	0,013036436	1	-0,02067324
chr15	26450000	26700000	0,001103225	1	-0,021202203
chr15	26500000	26750000	0,005000524	1	-0,021633495
chr15	26800000	27050000	0,047420827	1	-0,024780427
chr15	26850000	27100000	0,007391718	1	-0,026094418

chr15	26900000	27150000	0,000408005	1	-0,027182763
chr15	26950000	27200000	0,001806424	1	-0,027926633
chr15	27000000	27250000	0,020705178	1	-0,028254562
chr15	27250000	27500000	0,025378885	1	-0,024132154
chr15	27300000	27550000	0,00648529	1	-0,022868132
chr15	27850000	28100000	0,025805426	1	-0,039171779
chr15	27900000	28150000	0,00344491	1	-0,040886563
chr15	27950000	28200000	0,001063049	1	-0,04235212
chr15	28000000	28250000	0,02723819	1	-0,04338934
chr15	29250000	29500000	0,023372774	1	-0,024338929
chr15	29300000	29550000	0,004523107	1	-0,024338929
chr15	30450000	30700000	0,025767061	1	-0,008918123
chr15	30550000	30800000	0,018662569	1	-0,008918123
chr15	30600000	30850000	0,033300126	1	-0,008463261
chr15	30800000	31050000	0,031863974	1	-0,009067688

chr15	31600000	31850000	0,003039108	1	-0,035466476
chr15	31650000	31900000	0,0154725	1	-0,033817275
chr15	32350000	32600000	0,023089121	1	-0,013596354
chr15	32600000	32850000	0,021835314	1	-0,015306785
chr15	33700000	33950000	0,026156259	1	-0,028413785
chr15	37750000	38000000	0,011391212	1	-0,015412755
chr15	37800000	38050000	0,003232785	1	-0,015412755
chr15	45200000	45450000	0,037600083	1	-0,011161002
chr15	45350000	45600000	0,035252263	1	-0,010507243
chr15	45900000	46150000	0,049958163	1	-0,027662137
chr15	45950000	46200000	0,033421188	1	-0,02853131
chr15	46000000	46250000	0,030485915	1	-0,029382285
chr15	46050000	46300000	0,025228493	1	-0,030041855
chr15	46100000	46350000	0,012650435	1	-0,03031926
chr15	46150000	46400000	0,002388226	1	-0,030083308

chr15	46200000	46450000	0,000366469	1	- 0,029304471
chr15	46250000	46500000	0,006170746	1	- 0,028057565
chr15	46700000	46950000	0,038934292	1	- 0,017740179
chr15	46750000	47000000	0,003741212	1	- 0,017740179
chr15	47200000	47450000	0,01273618	1	- 0,030076233
chr15	47950000	48200000	0,013771259	1	- 0,048046194
chr15	48000000	48250000	0,001673821	1	-0,05078659
chr15	48050000	48300000	0,029899826	1	- 0,047105588
chr15	49650000	49900000	0,008711998	1	- 0,015282431
chr15	49700000	49950000	0,000437966	1	- 0,015427482
chr15	49750000	50000000	0,000390552	1	- 0,015427482
chr15	49800000	50050000	0,002513956	1	- 0,015427482
chr15	49850000	50100000	0,008153112	1	- 0,015427482
chr15	49900000	50150000	0,008260687	1	- 0,015427482
chr15	49950000	50200000	0,002003068	1	-

					0,015729385
chr15	50000000	50250000	0,018741122	1	- 0,016159645
chr15	52550000	52800000	0,002043864	1	-0,00588892
chr15	52600000	52850000	0,027433267	1	- 0,006421997
chr15	53250000	53500000	0,041108328	1	- 0,028346153
chr15	55100000	55350000	0,036285197	1	- 0,036868734
chr15	56500000	56750000	0,021290826	1	- 0,016367092
chr15	56550000	56800000	0,000358268	1	- 0,016104552
chr15	56600000	56850000	0,000583105	1	- 0,015860013
chr15	56650000	56900000	0,001459748	1	- 0,015860013
chr15	56700000	56950000	0,000935842	1	- 0,015860013
chr15	56750000	57000000	0,0001885	1	- 0,015860013
chr15	56800000	57050000	9,19E-06	1	- 0,015860013
chr15	56850000	57100000	8,25E-07	1	- 0,015692549
chr15	56900000	57150000	9,69E-07	1	- 0,015621762

chr15	56950000	57200000	1,23E-05	1	-0,015635471
chr15	57000000	57250000	0,001246911	1	-0,015697152
chr15	57050000	57300000	0,027165749	1	-0,014087151
chr15	61200000	61450000	0,002051941	1	-0,040762013
chr15	61250000	61500000	0,002368134	1	-0,039377693
chr15	61300000	61550000	0,021115282	1	-0,037768584
chr15	62250000	62500000	0,019932767	1	-0,007716908
chr15	62300000	62550000	0,002802856	1	-0,007051204
chr15	62350000	62600000	0,002507998	1	-0,006942657
chr15	68750000	69000000	0,00348041	1	-0,018954301
chr15	68800000	69050000	0,024742215	1	-0,018141607
chr15	69000000	69250000	0,044681303	1	-0,018141607
chr15	69050000	69300000	0,024189038	1	-0,017403559
chr15	69100000	69350000	0,016431669	1	-0,016795874

chr15	69150000	69400000	0,018389527	1	- 0,016312803
chr15	69200000	69450000	0,031117863	1	- 0,015917791
chr15	69350000	69600000	0,020928033	1	- 0,014667565
chr15	69400000	69650000	0,006064502	1	- 0,014106703
chr15	69900000	70150000	0,035078523	1	- 0,032915917
chr15	69950000	70200000	0,007567542	1	- 0,032745246
chr15	80350000	80600000	0,045845726	1	- 0,011666245
chr15	80400000	80650000	0,009617277	1	-0,01099425
chr15	82100000	82350000	0,009210694	1	- 0,032833761
chr15	84050000	84300000	0,035444368	1	- 0,031283871
chr15	84100000	84350000	0,00379805	1	- 0,031283871
chr15	84150000	84400000	0,001396693	1	- 0,031283871
chr15	84200000	84450000	0,000642518	1	- 0,031283871
chr15	84250000	84500000	0,000304904	1	- 0,031283871
chr15	84300000	84550000	0,023111911	1	-

					0,031743335
chr15	86550000	86800000	0,017822288	1	-0,04013605
chr15	86600000	86850000	0,005669316	1	-0,038040047
chr15	87050000	87300000	0,046991064	1	-0,020642514
chr15	87100000	87350000	0,001633307	1	-0,020642514
chr15	87150000	87400000	0,004878048	1	-0,020642514
chr15	87200000	87450000	0,040352042	1	-0,020358843
chr15	87450000	87700000	0,021499668	1	-0,024257689
chr15	87500000	87750000	0,01729454	1	-0,025550906
chr15	91100000	91350000	0,047161246	1	-0,007360107
chr15	91250000	91500000	0,049387638	1	-0,007752775
chr15	92000000	92250000	0,01020418	1	-0,060919315
chr15	92050000	92300000	0,001064087	1	-0,059528406
chr15	92100000	92350000	0,005878707	1	-0,058387792
chr15	92150000	92400000	0,008722187	1	-0,057313554

chr15	92200000	92450000	0,002512349	1	- 0,057951839
chr15	92250000	92500000	0,000443032	1	-0,05944207
chr15	92300000	92550000	0,003322203	1	- 0,061140372
chr15	92350000	92600000	0,00981969	1	- 0,061140372
chr15	92400000	92650000	0,006277146	1	- 0,061140372
chr15	92450000	92700000	0,000941994	1	- 0,061140372
chr15	92500000	92750000	0,003663679	1	- 0,061140372
chr15	93850000	94100000	0,009491466	1	- 0,056392745
chr15	93900000	94150000	0,00293268	1	- 0,054069933
chr15	93950000	94200000	0,045500079	1	- 0,052680939
chr15	95200000	95450000	0,019529053	1	- 0,040476926
chr15	96000000	96250000	0,003901817	1	- 0,005933847
chr15	96050000	96300000	0,000527737	1	- 0,005621318
chr15	96700000	96950000	0,036566663	1	- 0,039842781
chr15	96750000	97000000	0,006415308	1	-

					0,043227059
chr15	98500000	98750000	0,027191302	1	-0,038579632
chr15	98550000	98800000	0,031185191	1	-0,041558438
chr15	100550000	100800000	0,049670238	1	-0,024709741
chr16	5500000	5750000	0,00717524	1	-0,035392546
chr16	5550000	5800000	0,029344927	1	-0,033167435
chr16	6300000	6550000	0,034976831	1	-0,01270846
chr16	8050000	8300000	0,039276883	1	-0,041905597
chr16	8100000	8350000	0,016513961	1	-0,043427049
chr16	8150000	8400000	0,002419899	1	-0,044997902
chr16	8200000	8450000	0,001282203	1	-0,046455214
chr16	8250000	8500000	0,032563826	1	-0,047530661
chr16	9500000	9750000	0,028174749	1	-0,041931552
chr16	9550000	9800000	0,005272037	1	-0,040064886
chr16	9600000	9850000	0,047495374	1	-0,038008537

chr16	9850000	10100000	0,039416947	1	-0,035596331
chr16	9900000	10150000	0,027709931	1	-0,03465502
chr16	9950000	10200000	0,038941269	1	-0,033538546
chr16	10600000	10850000	0,007404935	1	-0,014274483
chr16	10650000	10900000	0,047089047	1	-0,015056029
chr16	10850000	11100000	0,018387174	1	-0,015494282
chr16	12500000	12750000	0,01251131	1	-0,044443362
chr16	12550000	12800000	0,001143873	1	-0,043091375
chr16	12600000	12850000	0,01091773	1	-0,041491545
chr16	12650000	12900000	0,040135587	1	-0,039893457
chr16	12850000	13100000	0,044698777	1	-0,034939116
chr16	12900000	13150000	0,041872142	1	-0,033726471
chr16	13100000	13350000	0,034021584	1	-0,030052557
chr16	13150000	13400000	0,005668628	1	-0,030052557
chr16	13200000	13450000	0,003592538	1	-

					0,030052557
chr16	13500000	13750000	0,036814816	1	- 0,037143032
chr16	13550000	13800000	0,010795899	1	- 0,039697391
chr16	17000000	17250000	0,007468469	1	-0,02048026
chr16	17050000	17300000	0,004366871	1	- 0,019741979
chr16	17100000	17350000	0,028917642	1	- 0,018971077
chr16	17300000	17550000	0,019503579	1	- 0,017708054
chr16	17350000	17600000	0,002457545	1	- 0,017708054
chr16	17400000	17650000	0,000955129	1	- 0,017660066
chr16	17450000	17700000	0,020128265	1	- 0,017271392
chr16	17850000	18100000	0,046017279	1	- 0,025221329
chr16	21200000	21450000	0,014030667	1	- 0,020134513
chr16	23400000	23650000	0,047618588	1	- 0,009368186
chr16	23450000	23700000	0,011578058	1	- 0,009992032
chr16	23500000	23750000	0,012580947	1	- 0,011388905

chr16	23550000	23800000	0,02302796	1	-0,011752009
chr16	23600000	23850000	0,021580821	1	-0,011905204
chr16	23650000	23900000	0,005567657	1	-0,011655497
chr16	23700000	23950000	0,008716733	1	-0,010903512
chr16	25500000	25750000	0,017275227	1	-0,033684445
chr16	26800000	27050000	0,013856566	1	-0,031546017
chr16	27100000	27350000	0,043779627	1	-0,027062131
chr16	27150000	27400000	0,002827117	1	-0,025235086
chr16	27200000	27450000	0,018139996	1	-0,024159445
chr16	27400000	27650000	0,010280719	1	-0,028594601
chr16	27450000	27700000	0,000965418	1	-0,028594601
chr16	27500000	27750000	0,010438141	1	-0,028594601
chr16	33600000	33850000	0,043253982	1	-0,025497824
chr16	33750000	34000000	0,030944424	1	-0,027046927

chr16	33800000	34050000	0,002727762	1	-0,027106064
chr16	33850000	34100000	0,000304826	1	-0,027616728
chr16	33900000	34150000	0,004488834	1	-0,028865452
chr16	34250000	34500000	0,003677012	1	-0,035516163
chr16	34300000	34550000	0,00178968	1	-0,037230648
chr16	34350000	34600000	0,012940329	1	-0,038054615
chr16	34400000	34650000	0,024978859	1	-0,037947527
chr16	34450000	34700000	0,01597025	1	-0,037118908
chr16	34500000	34750000	0,003083054	1	-0,035938275
chr16	34550000	34800000	0,000176867	1	-0,034803232
chr16	34600000	34850000	0,000490001	1	-0,034022727
chr16	34650000	34900000	0,001812901	1	-0,033991151
chr16	34700000	34950000	0,001306118	1	-0,035649865
chr16	34750000	35000000	0,000226396	1	-0,035005456

chr16	34800000	35050000	0,002497653	1	-0,033526183
chr16	47300000	47550000	0,032264399	1	-0,05652726
chr16	48150000	48400000	0,048321591	1	-0,012982673
chr16	48200000	48450000	0,001859243	1	-0,013996228
chr16	48250000	48500000	2,68E-05	1	-0,014555918
chr16	48300000	48550000	6,75E-05	1	-0,01475951
chr16	48350000	48600000	0,000714883	1	-0,015341769
chr16	48400000	48650000	0,001959949	1	-0,015611662
chr16	48450000	48700000	0,001284547	1	-0,015593638
chr16	48500000	48750000	0,000243446	1	-0,015289984
chr16	48550000	48800000	0,001601278	1	-0,014778396
chr16	48600000	48850000	0,013351644	1	-0,014275307
chr16	48650000	48900000	0,023051576	1	-0,014064347
chr16	48700000	48950000	0,007835188	1	-0,014395807
chr16	48750000	49000000	0,01055774	1	-0,015392791

chr16	49350000	49600000	0,049784529	1	-0,035304679
chr16	49400000	49650000	0,008337882	1	-0,037863856
chr16	51000000	51250000	0,031887115	1	-0,029005225
chr16	52700000	52950000	0,024174634	1	-0,044883995
chr16	52750000	53000000	0,012933517	1	-0,048354756
chr16	53350000	53600000	0,010256156	1	-0,024115919
chr16	53750000	54000000	0,030115753	1	-0,038668009
chr16	54950000	55200000	0,030107958	1	-0,044257403
chr16	55000000	55250000	0,01291227	1	-0,044257403
chr16	55050000	55300000	0,003774367	1	-0,044257403
chr16	55100000	55350000	0,000451647	1	-0,044257403
chr16	55150000	55400000	0,000181819	1	-0,045197033
chr16	55200000	55450000	0,005865716	1	-0,04584529
chr16	55500000	55750000	0,049179432	1	-0,037397643
chr16	55550000	55800000	0,005029877	1	-

					0,034055443
chr16	55600000	55850000	0,032613159	1	-0,034055443
chr16	55750000	56000000	0,015599527	1	-0,035100366
chr16	55800000	56050000	0,003567983	1	-0,036872417
chr16	56650000	56900000	0,001158718	1	-0,003945619
chr16	56700000	56950000	0,000334667	1	-0,003754919
chr16	56750000	57000000	0,022807383	1	-0,003770539
chr16	59100000	59350000	0,009197138	1	-0,030877628
chr16	63500000	63750000	0,01188715	1	-0,010089291
chr16	63550000	63800000	0,017930747	1	-0,009470628
chr16	64050000	64300000	0,047742077	1	-0,005482208
chr16	64100000	64350000	0,004847873	1	-0,005452113
chr16	64150000	64400000	0,036100429	1	-0,00560341
chr16	65300000	65550000	0,046187241	1	-0,023594351
chr16	65800000	66050000	0,007735142	1	-0,041424669

chr16	65850000	66100000	0,022980096	1	-0,043669226
chr16	72650000	72900000	0,042808177	1	-0,028920907
chr16	72700000	72950000	0,005740934	1	-0,030456611
chr16	72750000	73000000	0,000444825	1	-0,031691434
chr16	72800000	73050000	0,005321727	1	-0,032520759
chr16	76000000	76250000	0,044251135	1	-0,030910329
chr16	76550000	76800000	0,047334678	1	-0,011223576
chr16	76600000	76850000	0,009798673	1	-0,011223576
chr16	76800000	77050000	0,013327793	1	-0,012175019
chr16	77950000	78200000	0,012035818	1	-0,029624515
chr16	79200000	79450000	0,022847158	1	-0,027553571
chr16	79250000	79500000	0,00207942	1	-0,027527647
chr16	79300000	79550000	6,00E-05	1	-0,027360692
chr16	79350000	79600000	1,00E-05	1	-0,027130542

chr16	79400000	79650000	5,96E-05	1	-0,026941201
chr16	79450000	79700000	5,70E-05	1	-0,026943757
chr16	79500000	79750000	2,23E-05	1	-0,026988274
chr16	79550000	79800000	0,001460917	1	-0,026988274
chr16	79600000	79850000	0,035553165	1	-0,026988274
chr16	80050000	80300000	0,04519116	1	-0,049778139
chr16	80100000	80350000	0,003356966	1	-0,053441316
chr16	80150000	80400000	0,029461045	1	-0,055874416
chr16	83450000	83700000	0,01304864	1	-0,035107888
chr16	86700000	86950000	0,005876953	1	-0,011427679
chr16	86750000	87000000	0,007019289	1	-0,011427679
chr16	86800000	87050000	0,033300126	1	-0,011427679
chr16	86900000	87150000	0,035488975	1	-0,011681434
chr16	86950000	87200000	0,009385972	1	-0,012153497

chr17	2900000	3150000	0,01822088	1	-0,013307337
chr17	2950000	3200000	0,000466766	1	-0,013061648
chr17	3000000	3250000	0,001410882	1	-0,013061648
chr17	3050000	3300000	0,011704138	1	-0,013061648
chr17	8750000	9000000	0,003704666	1	-0,012108571
chr17	8800000	9050000	0,010838649	1	-0,012108571
chr17	9250000	9500000	0,029466719	1	-0,006613206
chr17	10000000	10250000	0,016719877	1	-0,031807476
chr17	11250000	11500000	0,014721092	1	-0,025006846
chr17	11300000	11550000	0,001035407	1	-0,026182722
chr17	11350000	11600000	0,006271501	1	-0,027052495
chr17	13150000	13400000	0,012860776	1	-0,0175648
chr17	13200000	13450000	0,003152025	1	-0,018076312
chr17	13250000	13500000	0,017109238	1	-0,0184427
chr17	13900000	14150000	0,020698401	1	-0,036842107

chr17	13950000	14200000	0,0018094	1	-0,038584938
chr17	14000000	14250000	0,000470173	1	-0,039909757
chr17	14050000	14300000	0,008510281	1	-0,037628849
chr17	20300000	20550000	0,004168281	1	-0,035511366
chr17	20350000	20600000	0,007422176	1	-0,035489269
chr17	21800000	22050000	0,003456316	1	-0,034970486
chr17	21850000	22100000	0,000708474	1	-0,033953759
chr17	21900000	22150000	0,013040995	1	-0,032398416
chr17	25350000	25600000	0,003185371	1	-0,021313621
chr17	25400000	25650000	2,69E-05	1	-0,022263531
chr17	25450000	25700000	6,32E-07	1	-0,022662644
chr17	25500000	25750000	0,000104001	1	-0,022712482
chr17	25550000	25800000	0,002192044	1	-0,022628321
chr17	25600000	25850000	0,011776252	1	-0,022602321

chr17	25650000	25900000	0,023990066	1	-0,022778158
chr17	25700000	25950000	0,020794487	1	-0,025786773
chr17	25750000	26000000	0,006700035	1	-0,025751294
chr17	25800000	26050000	0,00062315	1	-0,025270951
chr17	25850000	26100000	0,001379682	1	-0,024452641
chr17	25900000	26150000	0,015159309	1	-0,023479121
chr17	31150000	31400000	0,029262893	1	-0,033976322
chr17	31200000	31450000	0,007136517	1	-0,032217924
chr17	32800000	33050000	0,015618588	1	-0,041771715
chr17	33250000	33500000	0,01605228	1	-0,022671995
chr17	33300000	33550000	0,001578799	1	-0,021383904
chr17	33350000	33600000	0,013410397	1	-0,020852966
chr17	34500000	34750000	0,020186099	1	-0,017341578
chr17	34800000	35050000	0,015625563	1	-0,015124399

chr17	34850000	35100000	0,045519642	1	-0,014462402
chr17	49550000	49800000	0,040044178	1	-0,043062809
chr17	49600000	49850000	0,031084832	1	-0,039888728
chr17	50000000	50250000	0,007405933	1	-0,025030562
chr17	50050000	50300000	0,00879024	1	-0,026089937
chr17	50100000	50350000	0,0462708	1	-0,027147383
chr17	50250000	50500000	0,02264234	1	-0,029114618
chr17	50300000	50550000	0,006742215	1	-0,029423148
chr17	50350000	50600000	0,001666639	1	-0,029666691
chr17	50400000	50650000	0,000424784	1	-0,029847125
chr17	50450000	50700000	0,000101694	1	-0,029906541
chr17	50500000	50750000	1,18E-05	1	-0,029783742
chr17	50550000	50800000	7,83E-06	1	-0,029470483
chr17	50600000	50850000	0,000136733	1	-0,029061043

chr17	50650000	50900000	0,000519893	1	-0,028742489
chr17	50700000	50950000	0,000432495	1	-0,028726979
chr17	50750000	51000000	8,95E-05	1	-0,029152986
chr17	50800000	51050000	0,000692449	1	-0,030006302
chr17	50850000	51100000	0,008707947	1	-0,031087938
chr17	50900000	51150000	0,026441014	1	-0,031754673
chr17	50950000	51200000	0,026465153	1	-0,031754673
chr17	51000000	51250000	0,007943326	1	-0,031754673
chr17	51050000	51300000	0,001117991	1	-0,031754673
chr17	51100000	51350000	0,012680578	1	-0,032057995
chr17	51450000	51700000	0,019471806	1	-0,024127875
chr17	51500000	51750000	0,002689493	1	-0,023004068
chr17	51550000	51800000	0,000648134	1	-0,022332167
chr17	51600000	51850000	0,000693032	1	-0,021980578

chr17	51650000	51900000	0,001367236	1	-0,021778736
chr17	51700000	51950000	0,001978723	1	-0,021584763
chr17	51750000	52000000	0,001394338	1	-0,021325633
chr17	51800000	52050000	0,000295505	1	-0,020997565
chr17	51850000	52100000	0,000150594	1	-0,020652735
chr17	51900000	52150000	0,005149848	1	-0,020374434
chr17	52350000	52600000	0,020005301	1	-0,029696084
chr17	52400000	52650000	0,005720446	1	-0,030974809
chr17	52450000	52700000	0,00267544	1	-0,031918263
chr17	52500000	52750000	0,00345545	1	-0,032572833
chr17	52550000	52800000	0,008544846	1	-0,033052197
chr17	52600000	52850000	0,020360996	1	-0,033502996
chr17	52650000	52900000	0,032016236	1	-0,034079126
chr17	52700000	52950000	0,026090575	1	-0,034895945

chr17	52750000	53000000	0,007484291	1	-0,035980678
chr17	52800000	53050000	0,001695948	1	-0,037223709
chr17	52850000	53100000	0,045243883	1	-0,038359905
chr17	59100000	59350000	0,047765677	1	-0,006699875
chr17	59150000	59400000	0,001459006	1	-0,006699875
chr17	59200000	59450000	0,000492554	1	-0,006745476
chr17	59250000	59500000	0,017668744	1	-0,006847383
chr17	64650000	64900000	0,0474539	1	-0,012315587
chr17	66500000	66750000	0,029250506	1	-0,011154336
chr17	66550000	66800000	0,001737694	1	-0,010826069
chr17	66600000	66850000	0,004401557	1	-0,010650783
chr17	66650000	66900000	0,003381205	1	-0,01083737
chr17	66700000	66950000	0,004618665	1	-0,01145155
chr17	67000000	67250000	0,006971315	1	-0,013386458
chr17	67050000	67300000	0,005877869	1	-0,0141738
chr17	67100000	67350000	0,021273678	1	-

					0,014534623
chr17	67150000	67400000	0,012329695	1	-0,014417079
chr17	67200000	67450000	0,004644998	1	-0,013957662
chr17	67450000	67700000	0,017910039	1	-0,015925966
chr17	67950000	68200000	0,046846493	1	-0,006873394
chr17	68000000	68250000	0,002451422	1	-0,006979502
chr17	68050000	68300000	0,002258057	1	-0,00729869
chr17	68950000	69200000	0,020227064	1	-0,036864887
chr17	69000000	69250000	0,002263529	1	-0,038465448
chr17	69050000	69300000	0,000366925	1	-0,039788515
chr17	69100000	69350000	0,00485259	1	-0,040642824
chr17	69150000	69400000	0,024687462	1	-0,040869386
chr17	69200000	69450000	0,047902964	1	-0,040419834
chr17	69250000	69500000	0,046758101	1	-0,03939851
chr17	69300000	69550000	0,024638997	1	-0,034103477
chr17	69350000	69600000	0,006812431	1	-

					0,033902782
chr17	69400000	69650000	0,000913732	1	-0,033839271
chr17	69450000	69700000	5,54E-05	1	-0,033797119
chr17	69500000	69750000	1,96E-06	1	-0,033744544
chr17	69550000	69800000	9,72E-08	1	-0,033753122
chr17	69600000	69850000	4,55E-07	1	-0,03398141
chr17	69650000	69900000	9,09E-05	1	-0,0346048
chr17	69700000	69950000	0,002333908	1	-0,0346048
chr17	69750000	70000000	0,018015833	1	-0,0346048
chr17	69950000	70200000	0,021693322	1	-0,037255064
chr17	70000000	70250000	0,002002713	1	-0,038956726
chr17	70050000	70300000	0,003952287	1	-0,040431968
chr17	70100000	70350000	0,045022336	1	-0,041283251
chr17	70350000	70600000	0,007326562	1	-0,034454819
chr17	70400000	70650000	0,000254522	1	-0,032932072
chr17	70450000	70700000	0,00045747	1	-0,032065771
chr17	70500000	70750000	0,000806427	1	-

					0,031858011
chr17	70550000	70800000	0,000211883	1	- 0,032148136
chr17	70600000	70850000	0,001354637	1	- 0,032674931
chr17	70650000	70900000	0,038422815	1	- 0,033138457
chr17	71750000	72000000	0,02950671	1	- 0,034715818
chr17	71800000	72050000	0,00181057	1	- 0,034715818
chr17	71850000	72100000	0,00317003	1	- 0,036036291
chr17	72350000	72600000	0,008549656	1	- 0,019588087
chr17	72400000	72650000	0,008988633	1	- 0,018679518
chr17	75500000	75750000	0,035350286	1	-0,01812018
chr17	80800000	81050000	0,014299684	1	-0,00465427
chr18	200000	450000	0,003124077	1	- 0,018943066
chr18	250000	500000	0,000192969	1	- 0,018943066
chr18	300000	550000	0,000192284	1	- 0,018609244
chr18	350000	600000	0,002172458	1	- 0,018749121
chr18	1050000	1300000	0,0033486	1	-

					0,019858737
chr18	1100000	1350000	0,01537087	1	-0,018830834
chr18	1350000	1600000	0,015586807	1	-0,016355557
chr18	1400000	1650000	0,00083091	1	-0,016740583
chr18	1450000	1700000	0,001496063	1	-0,016948171
chr18	1500000	1750000	0,001140367	1	-0,016696344
chr18	1550000	1800000	0,003327716	1	-0,016696344
chr18	4100000	4350000	0,041689122	1	-0,059965649
chr18	4150000	4400000	0,006717725	1	-0,056721912
chr18	4900000	5150000	0,049092041	1	-0,022391625
chr18	4950000	5200000	0,017472655	1	-0,021488477
chr18	5000000	5250000	0,003090885	1	-0,02066372
chr18	5050000	5300000	0,000335633	1	-0,020025399
chr18	5100000	5350000	0,000349252	1	-0,019637253
chr18	5150000	5400000	0,005242953	1	-0,019471548

chr18	7850000	8100000	0,011641613	1	-0,106727982
chr18	10650000	10900000	0,027994173	1	-0,011077365
chr18	14800000	15050000	0,019248855	1	-0,015770666
chr18	22450000	22700000	0,009410998	1	-0,025728316
chr18	23250000	23500000	0,021381857	1	-0,031231574
chr18	23650000	23900000	0,030275912	1	-0,019868866
chr18	23700000	23950000	0,000869014	1	-0,018620899
chr18	23750000	24000000	3,03E-06	1	-0,018019059
chr18	23800000	24050000	1,59E-08	1	-0,017825281
chr18	23850000	24100000	1,38E-08	1	-0,017818751
chr18	23900000	24150000	1,59E-06	1	-0,01801829
chr18	23950000	24200000	0,000274273	1	-0,018454578
chr18	24000000	24250000	0,009407051	1	-0,019368402
chr18	24400000	24650000	0,038068517	1	-0,030801097
chr18	26250000	26500000	0,043249815	1	-

					0,012038916
chr18	26300000	26550000	0,044699936	1	-0,012038916
chr18	28150000	28400000	0,041693388	1	-0,037747922
chr18	28200000	28450000	0,002551856	1	-0,04044194
chr18	28250000	28500000	0,019041746	1	-0,042205369
chr18	28750000	29000000	0,04728447	1	-0,024348223
chr18	28800000	29050000	0,036635677	1	-0,023339651
chr18	28850000	29100000	0,044870817	1	-0,022578063
chr18	29500000	29750000	0,038247038	1	-0,011764039
chr18	30200000	30450000	0,035151115	1	-0,033581688
chr18	32100000	32350000	0,045061681	1	-0,019622871
chr18	32150000	32400000	0,002051958	1	-0,02099603
chr18	32200000	32450000	0,001308604	1	-0,02190773
chr18	32250000	32500000	0,009433186	1	-0,022294279
chr18	32300000	32550000	0,022085332	1	-0,022193898
chr18	32350000	32600000	0,031433447	1	-0,021744643

chr18	34350000	34600000	0,027012646	1	-0,048387377
chr18	34400000	34650000	0,008916152	1	-0,045556352
chr18	35100000	35350000	0,002109846	1	-0,052548083
chr18	35150000	35400000	0,005308339	1	-0,05032164
chr18	35750000	36000000	0,00308659	1	-0,019890888
chr18	35800000	36050000	1,03E-05	1	-0,019890888
chr18	35850000	36100000	9,38E-06	1	-0,019890888
chr18	35900000	36150000	1,61E-05	1	-0,019890888
chr18	35950000	36200000	0,000134812	1	-0,020009605
chr18	36000000	36250000	0,001997024	1	-0,020079824
chr18	36050000	36300000	0,014934907	1	-0,020128523
chr18	36350000	36600000	0,023042128	1	-0,024298024
chr18	36400000	36650000	0,008503665	1	-0,025311966
chr18	36450000	36700000	0,006400812	1	-0,026122661
chr18	36500000	36750000	0,016555518	1	-

					0,026707717
chr18	36900000	37150000	0,005596183	1	- 0,041470374
chr18	36950000	37200000	0,009610901	1	- 0,041470374
chr18	38950000	39200000	0,044238264	1	- 0,021332538
chr18	39000000	39250000	0,031882061	1	- 0,022223456
chr18	39050000	39300000	0,031250417	1	- 0,022994132
chr18	39100000	39350000	0,036954697	1	- 0,023684614
chr18	39150000	39400000	0,041892312	1	- 0,024367181
chr18	39200000	39450000	0,037404793	1	- 0,025111172
chr18	39250000	39500000	0,022469817	1	- 0,025945786
chr18	39300000	39550000	0,007455272	1	- 0,026842962
chr18	39350000	39600000	0,000938582	1	- 0,027715476
chr18	39400000	39650000	6,57E-05	1	- 0,028445461
chr18	39450000	39700000	0,000514191	1	- 0,028920421
chr18	39500000	39750000	0,003008997	1	-

					0,027288272
chr18	39550000	39800000	0,00667608	1	-0,026693092
chr18	39600000	39850000	0,008859148	1	-0,026121733
chr18	39650000	39900000	0,009480683	1	-0,025541656
chr18	39700000	39950000	0,010398387	1	-0,024916213
chr18	39750000	40000000	0,013134299	1	-0,024239516
chr18	39800000	40050000	0,017628798	1	-0,023555495
chr18	39850000	40100000	0,020822477	1	-0,022947051
chr18	39900000	40150000	0,017361317	1	-0,022527257
chr18	39950000	40200000	0,007745926	1	-0,022398343
chr18	40000000	40250000	0,001144164	1	-0,022626087
chr18	40050000	40300000	0,000290628	1	-0,023196879
chr18	40100000	40350000	0,004503728	1	-0,023997232
chr18	40150000	40400000	0,020634608	1	-0,024808053
chr18	40200000	40450000	0,02903407	1	-

					0,025346567
chr18	40250000	40500000	0,013099546	1	- 0,025338676
chr18	40300000	40550000	0,001769799	1	- 0,024610462
chr18	40350000	40600000	0,011045896	1	- 0,023162871
chr18	41700000	41950000	0,01490051	1	- 0,028511201
chr18	42400000	42650000	0,033022177	1	- 0,026260486
chr18	44850000	45100000	0,017548439	1	-0,02290483
chr18	44900000	45150000	0,001858219	1	-0,02290483
chr18	44950000	45200000	0,024338526	1	- 0,023655075
chr18	45350000	45600000	0,004554036	1	- 0,016104681
chr18	45400000	45650000	0,000384841	1	- 0,015381635
chr18	45450000	45700000	0,02765903	1	- 0,013521662
chr18	47250000	47500000	0,047659119	1	- 0,038471573
chr18	47300000	47550000	0,020248936	1	- 0,035805559
chr18	48800000	49050000	0,032651881	1	- 0,026706728
chr18	51300000	51550000	0,042391889	1	-

					0,041286747
chr18	51350000	51600000	0,002190376	1	-0,044480408
chr18	51400000	51650000	0,013986202	1	-0,046392446
chr18	52050000	52300000	0,011281486	1	-0,024770792
chr18	52100000	52350000	0,001089414	1	-0,024770792
chr18	52150000	52400000	0,008567983	1	-0,024770792
chr18	52200000	52450000	0,027975127	1	-0,024620738
chr18	52250000	52500000	0,033045371	1	-0,025025872
chr18	52300000	52550000	0,019811079	1	-0,025793235
chr18	52350000	52600000	0,006560482	1	-0,026694596
chr18	52400000	52650000	0,00100702	1	-0,028832521
chr18	52450000	52700000	5,71E-05	1	-0,028474316
chr18	52500000	52750000	0,000558252	1	-0,027688939
chr18	52550000	52800000	0,012855675	1	-0,026353941
chr18	56700000	56950000	0,044172915	1	-

					0,013463846
chr18	56950000	57200000	0,028712706	1	-0,011205572
chr18	57000000	57250000	0,000572321	1	-0,011205572
chr18	57050000	57300000	7,67E-05	1	-0,011205572
chr18	57100000	57350000	0,01411088	1	-0,011205572
chr18	59400000	59650000	0,040937733	1	-0,032480789
chr18	61150000	61400000	0,018217698	1	-0,032010033
chr18	61200000	61450000	0,003718103	1	-0,030498011
chr18	61550000	61800000	0,015128278	1	-0,022451557
chr18	61600000	61850000	0,016513254	1	-0,023484587
chr18	61700000	61950000	0,027661049	1	-0,02381772
chr18	61750000	62000000	0,00844213	1	-0,022383173
chr18	64700000	64950000	0,028474457	1	-0,017576457
chr18	64750000	65000000	0,005423553	1	-0,018682633
chr18	64800000	65050000	0,011933079	1	-0,019263569

chr18	65250000	65500000	0,030767236	1	- 0,034711389
chr18	65300000	65550000	0,006432446	1	- 0,032812596
chr18	65850000	66100000	0,008837096	1	-0,01045646
chr18	65900000	66150000	0,000315763	1	- 0,010298065
chr18	65950000	66200000	0,006956277	1	- 0,009783266
chr18	67350000	67600000	0,034405267	1	- 0,016463084
chr18	67400000	67650000	0,031644052	1	- 0,017935041
chr18	72450000	72700000	0,04774785	1	- 0,028285644
chr18	72500000	72750000	0,035903473	1	- 0,028285644
chr18	73950000	74200000	0,029626637	1	- 0,015891978
chr18	74000000	74250000	0,004684768	1	- 0,016778776
chr18	74050000	74300000	0,002012862	1	- 0,017363553
chr18	74100000	74350000	0,005939426	1	-0,01768649
chr18	74150000	74400000	0,025801336	1	- 0,017872868
chr18	74450000	74700000	0,034037315	1	- 0,021971422

chr18	74500000	74750000	0,009072204	1	-0,022934367
chr18	74550000	74800000	0,001013908	1	-0,023764369
chr18	74600000	74850000	9,00E-05	1	-0,024369479
chr18	74650000	74900000	0,001345728	1	-0,02368369
chr18	74700000	74950000	0,011946232	1	-0,022788291
chr18	74750000	75000000	0,046438251	1	-0,02173035
chr18	76400000	76650000	0,03657095	1	-0,025558191
chr18	76450000	76700000	0,007522643	1	-0,027510049
chr18	77850000	78077248	0,008780575	1	-0,008878858
chr20	0	250000	0,034489529	1	-0,013368265
chr20	50000	300000	0,020622933	1	-0,01311285
chr20	100000	350000	0,00418667	1	-0,012676653
chr20	550000	800000	0,005180137	1	-0,021558547
chr20	600000	850000	0,006340818	1	-0,020569944
chr20	4300000	4550000	0,013628526	1	-0,012575288
chr20	5250000	5500000	0,002407816	1	-0,01586762

chr20	5300000	5550000	0,000833739	1	-0,01586762
chr20	5350000	5600000	0,007010991	1	-0,01586762
chr20	5400000	5650000	0,029659074	1	-0,015751047
chr20	9600000	9850000	0,03693621	1	-0,047880821
chr20	9650000	9900000	0,00251895	1	-0,051070796
chr20	9700000	9950000	0,020115515	1	-0,053261957
chr20	10200000	10450000	0,031200444	1	-0,023396733
chr20	10450000	10700000	0,012368155	1	-0,024855012
chr20	11500000	11750000	0,004744953	1	-0,036701837
chr20	11550000	11800000	7,76E-05	1	-0,038760554
chr20	11600000	11850000	7,64E-05	1	-0,039501236
chr20	11650000	11900000	0,000108191	1	-0,039357978
chr20	11700000	11950000	0,0024698	1	-0,040936277
chr20	11750000	12000000	0,015874592	1	-0,042361992
chr20	11800000	12050000	0,029192383	1	-0,043431525

chr20	11850000	12100000	0,018167747	1	-0,043738777
chr20	11900000	12150000	0,003072589	1	-0,043061956
chr20	11950000	12200000	0,001409879	1	-0,041451341
chr20	12000000	12250000	0,024338526	1	-0,039215951
chr20	12250000	12500000	0,021861305	1	-0,03360497
chr20	12300000	12550000	0,001012028	1	-0,034396347
chr20	12350000	12600000	0,001639673	1	-0,035079081
chr20	12400000	12650000	0,00268815	1	-0,035137226
chr20	12450000	12700000	0,000826841	1	-0,034165812
chr20	12500000	12750000	0,013106061	1	-0,03364663
chr20	16250000	16500000	0,006645134	1	-0,024513028
chr20	16300000	16550000	8,73E-05	1	-0,025773924
chr20	16350000	16600000	1,17E-06	1	-0,026381022
chr20	16400000	16650000	0,000144127	1	-0,026489672
chr20	16450000	16700000	0,005948412	1	-0,026343801

chr20	16800000	17050000	0,028763659	1	-0,033955832
chr20	16850000	17100000	0,002136014	1	-0,035624923
chr20	16900000	17150000	0,007307102	1	-0,037027876
chr20	17450000	17700000	0,039728379	1	-0,013845743
chr20	17500000	17750000	0,001880199	1	-0,012043223
chr20	17550000	17800000	0,012053748	1	-0,011332227
chr20	19300000	19550000	0,006693667	1	-0,039649458
chr20	19350000	19600000	0,020463792	1	-0,041841189
chr20	19850000	20100000	0,020858357	1	-0,021495606
chr20	20400000	20650000	0,012697277	1	-0,047025439
chr20	20450000	20700000	0,004206054	1	-0,049950334
chr20	24550000	24800000	0,006252678	1	-0,038744162
chr20	37950000	38200000	0,03468281	1	-0,030285682
chr20	38800000	39050000	0,022965189	1	-0,030402445

chr20	40500000	40750000	0,047110444	1	-0,035587231
chr20	40550000	40800000	0,001801686	1	-0,034580008
chr20	40600000	40850000	0,007141591	1	-0,033151224
chr20	40950000	41200000	0,012299574	1	-0,026072631
chr20	41000000	41250000	0,003986703	1	-0,026996483
chr20	41050000	41300000	0,04777061	1	-0,026996483
chr20	41400000	41650000	0,029336791	1	-0,033588026
chr20	41450000	41700000	0,006643822	1	-0,034933239
chr20	41500000	41750000	0,000801335	1	-0,036154665
chr20	41550000	41800000	0,014110538	1	-0,037156984
chr20	49800000	50050000	0,029888162	1	-0,019739975
chr20	49850000	50100000	0,009766155	1	-0,019739975
chr20	50950000	51200000	0,007352087	1	-0,024187658
chr20	51550000	51800000	0,002131558	1	-0,008789406

chr20	51600000	51850000	0,001901753	1	-0,008371202
chr20	51650000	51900000	0,001563363	1	-0,008302627
chr20	51700000	51950000	0,00599393	1	-0,008443159
chr20	53100000	53350000	0,010542191	1	-0,051963602
chr20	53150000	53400000	0,006282339	1	-0,049317126
chr20	53900000	54150000	0,048526871	1	-0,022247439
chr20	53950000	54200000	0,00986181	1	-0,022144549
chr20	54000000	54250000	0,000903152	1	-0,021300531
chr20	54050000	54300000	8,71E-05	1	-0,020761377
chr20	54100000	54350000	0,00434376	1	-0,020459714
chr20	54550000	54800000	0,026085994	1	-0,035713698
chr20	55000000	55250000	0,000670618	1	-0,017907255
chr20	55050000	55300000	0,000946845	1	-0,017228292
chr20	56150000	56400000	0,012173359	1	-0,025142104

chr20	56600000	56850000	0,009041233	1	-0,015051426
chr20	56650000	56900000	0,006003092	1	-0,014212657
chr20	57100000	57350000	0,013421337	1	-0,022433767
chr20	57150000	57400000	0,000829922	1	-0,022433767
chr20	57200000	57450000	0,005815772	1	-0,022828275
chr20	58200000	58450000	0,016005526	1	-0,03417485
chr20	58400000	58650000	0,011433745	1	-0,029858206
chr20	58450000	58700000	0,010598386	1	-0,031265244
chr20	58500000	58750000	0,047879076	1	-0,032310022
chr20	58550000	58800000	0,042033423	1	-0,032442922
chr20	58600000	58850000	0,007602506	1	-0,031423388
chr20	58650000	58900000	0,015458881	1	-0,029343653
chr20	59200000	59450000	0,004026612	1	-0,01283985
chr20	59250000	59500000	0,047286633	1	-0,01283985
chr20	59950000	60200000	0,024805031	1	-0,041971708
chr20	60000000	60250000	0,006042788	1	-

					0,044791665
chr20	62700000	62950000	0,0301367	1	-0,008261222
chr19	600000	850000	0,038918315	1	-0,003029901
chr19	650000	900000	0,042374421	1	-0,003111602
chr19	1100000	1350000	0,041485751	1	-0,009199946
chr19	3300000	3550000	0,001808041	1	-0,002908355
chr19	3350000	3600000	0,001561112	1	-0,002921031
chr19	3400000	3650000	0,000550366	1	-0,002976471
chr19	3450000	3700000	0,000363313	1	-0,003020496
chr19	3500000	3750000	0,000522909	1	-0,003002534
chr19	3550000	3800000	0,000551041	1	-0,002906138
chr19	3600000	3850000	0,011489723	1	-0,00275674
chr19	15000000	15250000	0,008435723	1	-0,018461513
chr19	15050000	15300000	0,001887268	1	-0,018461513
chr19	15100000	15350000	0,039307692	1	-0,019003488

chr19	20200000	20450000	0,007680587	1	-0,023619416
chr19	20250000	20500000	0,042769349	1	-0,022118828
chr19	20500000	20750000	0,002486051	1	-0,019802445
chr19	20550000	20800000	4,72E-05	1	-0,019802445
chr19	20600000	20850000	0,012400206	1	-0,01948436
chr19	21900000	22150000	0,0304993	1	-0,023584026
chr19	21950000	22200000	0,037222203	1	-0,022056995
chr19	22350000	22600000	0,024985164	1	-0,015318281
chr19	22400000	22650000	0,002664199	1	-0,015607688
chr19	22450000	22700000	0,002053393	1	-0,016245593
chr19	22500000	22750000	0,031524151	1	-0,016657585
chr19	23450000	23700000	0,008418494	1	-0,048371816
chr19	23500000	23750000	0,00044719	1	-0,050727678
chr19	23550000	23800000	0,002402287	1	-0,052175782
chr19	23600000	23850000	0,011007645	1	-

					0,052609533
chr19	23650000	23900000	0,015910199	1	-0,052107307
chr19	23700000	23950000	0,009521236	1	-0,050939999
chr19	23750000	24000000	0,002352522	1	-0,04838334
chr19	23800000	24050000	0,000197385	1	-0,046767909
chr19	23850000	24100000	4,80E-06	1	-0,046634487
chr19	23900000	24150000	1,13E-05	1	-0,046087033
chr19	23950000	24200000	0,000472443	1	-0,044991764
chr19	24000000	24250000	0,006407279	1	-0,043316626
chr19	24050000	24300000	0,040228056	1	-0,041087831
chr19	27900000	28150000	0,027673338	1	-0,040872063
chr19	27950000	28200000	0,007199079	1	-0,04266692
chr19	28000000	28250000	0,000943881	1	-0,045999235
chr19	28050000	28300000	9,87E-05	1	-0,045153787
chr19	28100000	28350000	0,001710204	1	-0,043724279
chr19	28150000	28400000	0,01700238	1	-

					0,041879528
chr19	28400000	28650000	0,039227528	1	-<br>0,035305624
chr19	28450000	28700000	0,007724932	1	-<br>0,035165944
chr19	28500000	28750000	0,000746476	1	-<br>0,035029766
chr19	28550000	28800000	0,000124686	1	-<br>0,034608772
chr19	28600000	28850000	0,00085128	1	-<br>0,033684573
chr19	28650000	28900000	0,011155604	1	-<br>0,032167987
chr19	29150000	29400000	0,016153125	1	-<br>0,018242095
chr19	29800000	30050000	0,002427238	1	-<br>0,045780013
chr19	29850000	30100000	0,000620493	1	-<br>0,047875595
chr19	29900000	30150000	0,003396862	1	-<br>0,048739998
chr19	29950000	30200000	0,004789216	1	-<br>0,048541338
chr19	30000000	30250000	0,002286099	1	-<br>0,047683161
chr19	30050000	30300000	0,000395493	1	-<br>0,046631081
chr19	30100000	30350000	1,79E-05	1	-

					0,045750955
chr19	30150000	30400000	4,63E-06	1	-0,045526396
chr19	30200000	30450000	0,000169643	1	-0,046313362
chr19	30250000	30500000	0,002179643	1	-0,047643107
chr19	30300000	30550000	0,01387248	1	-0,049560091
chr19	30350000	30600000	0,049403592	1	-0,051913652
chr19	30550000	30800000	0,018053655	1	-0,057042409
chr19	30600000	30850000	0,001899012	1	-0,055091044
chr19	30650000	30900000	0,020346395	1	-0,051577816
chr19	31400000	31650000	0,010535418	1	-0,010660839
chr19	31700000	31950000	0,00503956	1	-0,012417785
chr19	31750000	32000000	0,013138751	1	-0,013085427
chr19	31800000	32050000	0,032982559	1	-0,01334579
chr19	31850000	32100000	0,012703057	1	-0,013155946
chr19	33850000	34100000	0,009960216	1	-0,011948716

chr19	34050000	34300000	0,015607899	1	-0,010909091
chr19	34100000	34350000	0,002596185	1	-0,010909091
chr19	34150000	34400000	0,012996615	1	-0,010909091
chr19	34200000	34450000	0,015338179	1	-0,010844752
chr19	34250000	34500000	0,00381895	1	-0,01109842
chr19	34300000	34550000	0,033603564	1	-0,011510605
chr19	36500000	36750000	0,017324228	1	-0,002410443
chr19	37100000	37350000	0,035930552	1	-0,014753771
chr19	40250000	40500000	0,03804477	1	-0,020285158
chr19	44450000	44700000	0,042869778	1	-0,015629817
chr19	44500000	44750000	0,010589397	1	-0,014808008
chr19	51250000	51500000	0,02343562	1	-0,013472454
chr19	51300000	51550000	0,004412951	1	-0,012974485
chr19	51350000	51600000	0,019057932	1	-0,01266242
chr19	51400000	51650000	0,014930697	1	-0,012752532

chr19	51450000	51700000	0,003610699	1	-0,013334544
chr19	51500000	51750000	0,04285656	1	-0,013651335
chr19	51750000	52000000	0,049134765	1	-0,017492931
chr19	51800000	52050000	0,005118381	1	-0,016683781
chr19	51850000	52100000	0,046456123	1	-0,015610007
chr19	52100000	52350000	0,011134639	1	-0,013535982
chr19	52500000	52750000	0,016119575	1	-0,020173035
chr19	52550000	52800000	0,000948432	1	-0,021172224
chr19	52600000	52850000	0,000219753	1	-0,021840128
chr19	52650000	52900000	0,004044824	1	-0,021806014
chr19	52700000	52950000	0,027312099	1	-0,021547028
chr19	56350000	56600000	0,010593417	1	-0,021246975
chr19	56400000	56650000	0,038255455	1	-0,019828398
chr19	58150000	58400000	0,008261503	1	-0,013955989

chr19	58200000	58450000	0,016383084	1	-0,013955989
chr22	17000000	17250000	0,010141975	1	-0,018399488
chr22	17050000	17300000	0,035334167	1	-0,019634786
chr22	17300000	17550000	0,012850151	1	-0,016512657
chr22	17600000	17850000	0,024902538	1	-0,019553031
chr22	22450000	22700000	0,036682215	1	-0,010183265
chr22	22750000	23000000	0,03037726	1	-0,008079453
chr22	23250000	23500000	0,012198114	1	-0,018120759
chr22	23300000	23550000	0,000677716	1	-0,019056721
chr22	23350000	23600000	2,43E-05	1	-0,019568563
chr22	23400000	23650000	1,12E-06	1	-0,01977879
chr22	23450000	23700000	0,000263219	1	-0,019855843
chr22	23500000	23750000	0,016283313	1	-0,019916614
chr22	28600000	28850000	0,00731484	1	-0,011658568
chr22	28650000	28900000	0,005515431	1	-

					0,011658568
chr22	32650000	32900000	0,039311746	1	- 0,019706686
chr22	32700000	32950000	0,0170773	1	- 0,018890954
chr22	33550000	33800000	0,018503711	1	-0,0323204
chr22	33600000	33850000	0,005476214	1	- 0,030795754
chr22	34050000	34300000	0,047847398	1	- 0,018510438
chr22	34100000	34350000	0,002542361	1	- 0,018901851
chr22	34150000	34400000	0,001986146	1	- 0,019561915
chr22	34200000	34450000	0,021344965	1	- 0,019677782
chr22	35050000	35300000	0,046621808	1	- 0,051761948
chr22	35100000	35350000	0,008974239	1	- 0,055706448
chr22	43950000	44200000	0,001733375	1	- 0,013041883
chr22	44000000	44250000	0,004587644	1	- 0,013620767
chr22	44050000	44300000	0,013577808	1	-0,01374981
chr22	44100000	44350000	0,011253409	1	- 0,013549603
chr22	44150000	44400000	0,004106823	1	-

					0,013178116
					-
chr22	44200000	44450000	0,002632285	1	0,012801403
					-
chr22	44250000	44500000	0,02597741	1	0,012539002
					-
chr22	47550000	47800000	0,02421256	1	0,017292157
					-
chr22	47600000	47850000	0,035291747	1	0,018044156
					-
chr22	48250000	48500000	0,01719153	1	0,036247518
					-
chr22	48300000	48550000	0,001487923	1	0,038198988
					-
chr22	48350000	48600000	0,018984972	1	0,039535046
					-
chr22	48650000	48900000	0,04147138	1	0,032048681
					-
chr22	49000000	49250000	0,011475949	1	0,046744084
					-
chr22	49050000	49300000	0,000222183	1	0,046346205
					-
chr22	49100000	49350000	2,06E-05	1	0,046129963
					-
chr22	49150000	49400000	2,90E-05	1	0,046129963
					-
chr22	49200000	49450000	5,03E-06	1	0,046129963
chr22	49250000	49500000	1,69E-06	1	-

					0,046129963
chr22	49300000	49550000	1,74E-06	1	-0,046129963
chr22	49350000	49600000	0,000100187	1	-0,046202636
chr22	49400000	49650000	0,004276693	1	-0,046446939
chr22	49450000	49700000	0,046877159	1	-0,046584089
chr22	51100000	51304566	0,017701403	1	-0,006348451
chr21	10700000	10950000	0,037514499	1	-0,043078158
chr21	15250000	15500000	0,011547555	1	-0,020969009
chr21	17400000	17650000	0,009147966	1	-0,05006523
chr21	17450000	17700000	0,000299475	1	-0,049089333
chr21	17500000	17750000	0,001962113	1	-0,047569809
chr21	17550000	17800000	0,017241399	1	-0,045618637
chr21	19300000	19550000	0,011412024	1	-0,030479019
chr21	19600000	19850000	0,016187806	1	-0,024391238
chr21	19650000	19900000	0,001793742	1	-0,025293467

chr21	19700000	19950000	0,021739086	1	-0,026564504
chr21	20050000	20300000	0,022252529	1	-0,034819092
chr21	20100000	20350000	0,003374415	1	-0,034741356
chr21	20150000	20400000	0,000183005	1	-0,034212141
chr21	20200000	20450000	0,000840256	1	-0,033224761
chr21	20250000	20500000	0,012109358	1	-0,031832933
chr21	20550000	20800000	0,024902682	1	-0,025749947
chr21	20600000	20850000	0,00140813	1	-0,026115906
chr21	20650000	20900000	0,000221334	1	-0,026559604
chr21	20700000	20950000	0,000999672	1	-0,026853794
chr21	20750000	21000000	0,000925592	1	-0,026940274
chr21	20800000	21050000	0,000159601	1	-0,026717371
chr21	20850000	21100000	8,33E-05	1	-0,026242259
chr21	20900000	21150000	0,001420409	1	-0,025581159

chr21	20950000	21200000	0,00820181	1	-0,024797793
chr21	21000000	21250000	0,023426903	1	-0,023915847
chr21	21050000	21300000	0,048983565	1	-0,022914814
chr21	21550000	21800000	0,002116474	1	-0,014490339
chr21	21600000	21850000	0,005719437	1	-0,015012093
chr21	21650000	21900000	0,03189178	1	-0,015624665
chr21	22000000	22250000	0,040694072	1	-0,018180488
chr21	22050000	22300000	0,004235713	1	-0,019117533
chr21	22100000	22350000	0,001203013	1	-0,019511666
chr21	22150000	22400000	0,015605043	1	-0,018652286
chr21	22400000	22650000	0,022475017	1	-0,015959019
chr21	22450000	22700000	0,008523818	1	-0,015685519
chr21	22500000	22750000	0,003952613	1	-0,015475663
chr21	22550000	22800000	0,001295172	1	-0,015486336

chr21	22600000	22850000	0,00031794	1	-0,015652262
chr21	22650000	22900000	0,013514808	1	-0,015697221
chr21	23000000	23250000	0,027207668	1	-0,022332863
chr21	23800000	24050000	0,017555579	1	-0,019236058
chr21	23850000	24100000	0,003640144	1	-0,019977969
chr21	23900000	24150000	0,000269644	1	-0,020584481
chr21	23950000	24200000	0,000515374	1	-0,020455923
chr21	24000000	24250000	0,010524047	1	-0,019566778
chr21	24400000	24650000	0,024204972	1	-0,013389193
chr21	24450000	24700000	0,011007645	1	-0,014137315
chr21	25200000	25450000	0,02768868	1	-0,023869072
chr21	25250000	25500000	0,021519325	1	-0,024800945
chr21	25300000	25550000	0,028848284	1	-0,025542896
chr21	25350000	25600000	0,046853874	1	-0,026186494

chr21	25550000	25800000	0,03063986	1	-0,02988643
chr21	25600000	25850000	0,014638092	1	-0,031041223
chr21	25650000	25900000	0,006398395	1	-0,032086866
chr21	25700000	25950000	0,002625527	1	-0,032962715
chr21	25750000	26000000	0,000843525	1	-0,033676371
chr21	25800000	26050000	0,00013625	1	-0,03426731
chr21	25850000	26100000	8,31E-06	1	-0,034755265
chr21	25900000	26150000	1,51E-05	1	-0,035109294
chr21	25950000	26200000	4,27E-05	1	-0,035268264
chr21	26000000	26250000	1,70E-05	1	-0,035197322
chr21	26050000	26300000	0,000107986	1	-0,034946014
chr21	26100000	26350000	0,002510866	1	-0,03466656
chr21	26150000	26400000	0,014326022	1	-0,034571321
chr21	26200000	26450000	0,032209537	1	-0,034847447
chr21	26250000	26500000	0,036984809	1	-0,035567821
chr21	26300000	26550000	0,023977177	1	-

					0,036662202
chr21	26350000	26600000	0,008379516	1	-0,037949676
chr21	26400000	26650000	0,001158508	1	-0,039206159
chr21	26450000	26700000	0,000133079	1	-0,040246093
chr21	26500000	26750000	0,003476382	1	-0,040946908
chr21	26550000	26800000	0,049063699	1	-0,041229281
chr21	27300000	27550000	0,016555518	1	-0,039734759
chr21	27350000	27600000	0,02803944	1	-0,037590499
chr21	28700000	28950000	0,014026908	1	-0,009684072
chr21	28750000	29000000	0,002806062	1	-0,009684072
chr21	28800000	29050000	0,048569189	1	-0,009413105
chr21	29550000	29800000	0,021519937	1	-0,043751583
chr21	29600000	29850000	0,004091687	1	-0,046684597
chr21	31000000	31250000	0,01488446	1	-0,042294415
chr21	31050000	31300000	0,002168959	1	-0,04222016

chr21	31100000	31350000	0,000136393	1	-0,041562527
chr21	31150000	31400000	0,001096233	1	-0,04023862
chr21	31200000	31450000	0,019290989	1	-0,038219521
chr21	32650000	32900000	0,009555851	1	-0,036528589
chr21	32700000	32950000	0,045740508	1	-0,038769612
chr21	35600000	35850000	0,00878749	1	-0,025736156
chr21	35650000	35900000	6,55E-05	1	-0,025663893
chr21	35700000	35950000	7,77E-07	1	-0,025620149
chr21	35750000	36000000	6,30E-06	1	-0,02545462
chr21	35800000	36050000	0,000167087	1	-0,024945328
chr21	35850000	36100000	0,004787626	1	-0,023907785
chr21	36350000	36600000	0,011610422	1	-0,011278473
chr21	36400000	36650000	4,50E-05	1	-0,01120984
chr21	36450000	36700000	9,53E-05	1	-0,010960106
chr21	36500000	36750000	0,002257985	1	-0,010632114
chr21	36550000	36800000	0,008286631	1	-

					0,010426367
chr21	36600000	36850000	0,005196597	1	- 0,010553262
chr21	36650000	36900000	0,00396321	1	- 0,011146322
chr21	39250000	39500000	0,019389855	1	- 0,041780939
chr21	39300000	39550000	0,001441284	1	- 0,041780939
chr21	39350000	39600000	0,016602096	1	- 0,041780939
chr21	41150000	41400000	0,030262667	1	- 0,047913359
chr21	41200000	41450000	0,000884575	1	- 0,046837212
chr21	41250000	41500000	0,002474295	1	- 0,045536164
chr21	41300000	41550000	0,012440669	1	- 0,044180286
chr21	41350000	41600000	0,025304665	1	- 0,042788562
chr21	41400000	41650000	0,036353938	1	- 0,041316034
chr21	41450000	41700000	0,048837228	1	- 0,039757321
chr21	41650000	41900000	0,039573347	1	- 0,035110267
chr21	41700000	41950000	0,014062853	1	-

					0,034776487
chr21	41750000	42000000	0,00286156	1	-0,03458357
chr21	41800000	42050000	0,000475451	1	-0,03458357
chr21	41850000	42100000	0,000441245	1	-0,03458357
chr21	41900000	42150000	0,003652052	1	-0,03458357
chr21	41950000	42200000	0,034868537	1	-0,03458357
chr21	47200000	47450000	0,031726291	1	- 0,014175194
chr21	47250000	47500000	0,003449751	1	- 0,015118271
chr21	47300000	47550000	0,040216794	1	- 0,015759124
chr21	47650000	47900000	0,0343038	1	- 0,010030769
chr21	47700000	47950000	0,007369427	1	- 0,010030769

Supplementary Table S3
List and Coordinates of PREP1 down-regulation induced early-to-late (EtoL) shifted genomic windows.

Chr	Start	End	Adj_Pvalue_LtoE	Adj_Pvalue_EtoL	Diff_S50
chr1	8100000	8350000	1	0,010046729	0,016668876
chr1	9450000	9700000	1	0,010668936	0,014763006
chr1	9800000	10050000	1	0,044046198	0,011653145
chr1	9850000	10100000	1	0,003207613	0,010992879
chr1	9900000	10150000	1	0,000108585	0,010579138
chr1	9950000	10200000	1	0,000327069	0,010014094
chr1	10000000	10250000	1	0,009027634	0,009543768
chr1	10850000	11100000	1	0,028269587	0,01293364
chr1	10900000	11150000	1	0,000985331	0,014332189
chr1	10950000	11200000	1	0,000138512	0,014018114
chr1	11000000	11250000	1	0,008116364	0,013326893
chr1	12100000	12350000	1	0,01686947	0,013277876
chr1	12150000	12400000	1	0,005639785	0,013058139
chr1	16100000	16350000	1	0,011598529	0,002972254
chr1	16150000	16400000	1	0,008526088	0,002807093
chr1	16200000	16450000	1	0,042771219	0,00271946
chr1	16250000	16500000	1	0,035218588	0,002903549
chr1	16300000	16550000	1	0,008779055	0,00287663
chr1	19450000	19700000	1	0,047325326	0,031490029
chr1	19500000	19750000	1	0,038638319	0,034497433
chr1	21000000	21250000	1	0,03729389	0,008389796
chr1	22050000	22300000	1	0,013236967	0,006117388
chr1	22100000	22350000	1	0,013654811	0,006123313
chr1	22150000	22400000	1	0,012812132	0,005717688
chr1	24600000	24850000	1	0,017094641	0,023845095
chr1	27250000	27500000	1	0,043594151	0,003931502
chr1	27300000	27550000	1	0,001173095	0,003931502
chr1	27350000	27600000	1	1,19E-05	0,003931502
chr1	27400000	27650000	1	0,002026305	0,003931502
chr1	27950000	28200000	1	0,019388358	0,013079842
chr1	28600000	28850000	1	0,014350039	0,003930439
chr1	32050000	32300000	1	0,021204414	0,003341968
chr1	32500000	32750000	1	0,007796403	0,005475981
chr1	32550000	32800000	1	0,000549082	0,005333545
chr1	32600000	32850000	1	0,008439846	0,005068764
chr1	35800000	36050000	1	0,002039133	0,004949127
chr1	35850000	36100000	1	0,014016497	0,005051808
chr1	36150000	36400000	1	0,036942673	0,006274001
chr1	38100000	38350000	1	0,020578054	0,011191839
chr1	40450000	40700000	1	0,019327041	0,027695418
chr1	40500000	40750000	1	0,012510837	0,02596203

chr1	41600000	41850000	1	0,003609384	0,014436388
chr1	41650000	41900000	1	0,005978473	0,013850561
chr1	43300000	43550000	1	0,019722602	0,014216151
chr1	44850000	45100000	1	0,00645372	0,008426756
chr1	44900000	45150000	1	0,001329429	0,008426756
chr1	44950000	45200000	1	0,002162509	0,008585262
chr1	45000000	45250000	1	0,001150426	0,008491055
chr1	45050000	45300000	1	0,037091098	0,008290107
chr1	45450000	45700000	1	0,0232324	0,01447936
chr1	51850000	52100000	1	0,012064577	0,033538217
chr1	51900000	52150000	1	0,001373793	0,034439602
chr1	51950000	52200000	1	6,87E-05	0,033973101
chr1	52000000	52250000	1	0,000510163	0,03316103
chr1	52050000	52300000	1	0,006594534	0,032021666
chr1	52100000	52350000	1	0,034779841	0,030574133
chr1	53550000	53800000	1	0,013617665	0,017837138
chr1	54000000	54250000	1	0,003972537	0,010329685
chr1	54050000	54300000	1	0,007215042	0,009892807
chr1	54100000	54350000	1	0,004020424	0,009937556
chr1	85650000	85900000	1	0,014795275	0,029965653
chr1	85700000	85950000	1	0,001106864	0,031457784
chr1	85750000	86000000	1	0,015558049	0,029999992
chr1	86500000	86750000	1	0,041276032	0,008686897
chr1	93500000	93750000	1	0,006398158	0,022477045
chr1	94550000	94800000	1	0,006164718	0,02031218
chr1	94900000	95150000	1	0,02250307	0,015320513
chr1	94950000	95200000	1	0,02182381	0,014293648
chr1	95150000	95400000	1	0,02502953	0,014848098
chr1	109300000	109550000	1	0,022807918	0,014994247
chr1	109750000	110000000	1	0,014633055	0,008739748
chr1	110300000	110550000	1	0,012332907	0,020869165
chr1	110350000	110600000	1	0,000381656	0,020451588
chr1	110400000	110650000	1	0,00212121	0,019850783
chr1	110450000	110700000	1	0,0172101	0,019074349
chr1	112000000	112250000	1	0,048002292	0,006600589
chr1	112500000	112750000	1	0,031771288	0,01003458
chr1	113000000	113250000	1	0,009575249	0,016273822
chr1	113050000	113300000	1	0,010978556	0,01738337
chr1	150150000	150400000	1	0,007009883	0,005768491
chr1	150200000	150450000	1	0,001282986	0,006066839
chr1	150250000	150500000	1	0,020190797	0,006176434
chr1	150650000	150900000	1	0,03942072	0,011835074
chr1	151450000	151700000	1	0,028555344	0,010087084
chr1	155750000	156000000	1	0,011515191	0,008476208
chr1	156300000	156550000	1	0,006290565	0,003534638
chr1	156350000	156600000	1	0,02278509	0,003737641

chr1	156650000	156900000	1	0,047531701	0,004628268
chr1	173700000	173950000	1	0,008734386	0,016512114
chr1	180450000	180700000	1	0,005084096	0,049509656
chr1	183200000	183450000	1	0,029617779	0,032782747
chr1	183250000	183500000	1	0,010024407	0,03069516
chr1	186450000	186700000	1	0,010734006	0,050866626
chr1	199050000	199300000	1	0,015027043	0,021524545
chr1	200400000	200650000	1	0,00755811	0,023021318
chr1	200450000	200700000	1	6,30E-05	0,024442505
chr1	200500000	200750000	1	4,81E-05	0,024954212
chr1	200550000	200800000	1	2,31E-05	0,024887841
chr1	200600000	200850000	1	0,000133326	0,025173658
chr1	200650000	200900000	1	0,000572809	0,025428512
chr1	200700000	200950000	1	0,000421766	0,025392706
chr1	200750000	201000000	1	9,70E-05	0,024947495
chr1	200800000	201050000	1	0,001998451	0,024947495
chr1	200850000	201100000	1	0,03841752	0,024947495
chr1	203900000	204150000	1	0,006135594	0,017343704
chr1	203950000	204200000	1	0,004954766	0,017343704
chr1	205500000	205750000	1	0,020483868	0,012189097
chr1	205550000	205800000	1	0,002690244	0,012074315
chr1	205600000	205850000	1	0,000916664	0,011634011
chr1	205650000	205900000	1	0,034279101	0,010838629
chr1	206600000	206850000	1	0,011920485	0,018520333
chr1	206650000	206900000	1	0,000545212	0,019460691
chr1	206700000	206950000	1	0,006696801	0,020018611
chr1	207350000	207600000	1	0,012187342	0,003976231
chr1	207400000	207650000	1	0,007211957	0,003669425
chr1	210300000	210550000	1	0,008106964	0,02056218
chr1	212100000	212350000	1	0,048620285	0,02194937
chr1	212150000	212400000	1	0,007648599	0,02194937
chr1	212200000	212450000	1	0,00059465	0,02194937
chr1	212250000	212500000	1	0,005771749	0,02194937
chr1	218500000	218750000	1	0,030571964	0,038353155
chr1	218550000	218800000	1	0,009716982	0,039515196
chr1	224050000	224300000	1	0,02332427	0,014268278
chr1	232250000	232500000	1	0,014566876	0,017875492
chr1	232300000	232550000	1	0,043408845	0,016690643
chr2	22200000	22450000	1	0,04632509	0,019568283
chr2	24300000	24550000	1	0,013820723	0,020205723
chr2	24350000	24600000	1	0,038572663	0,018703602
chr2	25150000	25400000	1	0,01425154	0,004503287
chr2	25200000	25450000	1	0,016315837	0,004160461
chr2	38350000	38600000	1	0,016839366	0,016809844
chr2	38400000	38650000	1	0,001938391	0,016809844
chr2	38450000	38700000	1	0,001204228	0,016809844

chr2	38500000	38750000	1	0,035388189	0,017168229
chr2	38900000	39150000	1	0,028061972	0,010511045
chr2	44050000	44300000	1	0,012316071	0,025332
chr2	44100000	44350000	1	0,006372342	0,025848919
chr2	46850000	47100000	1	0,010642677	0,025791751
chr2	46900000	47150000	1	0,005692638	0,027174997
chr2	55250000	55500000	1	0,005240428	0,017260772
chr2	55300000	55550000	1	0,008496528	0,018344791
chr2	55350000	55600000	1	0,022653068	0,018514851
chr2	55400000	55650000	1	0,022269001	0,018135309
chr2	55450000	55700000	1	0,02570563	0,017571197
chr2	58150000	58400000	1	0,002380336	0,005724021
chr2	58200000	58450000	1	0,002277445	0,005994119
chr2	64900000	65150000	1	0,010804246	0,021036123
chr2	64950000	65200000	1	0,00032366	0,022959246
chr2	65000000	65250000	1	6,03E-06	0,023016675
chr2	65050000	65300000	1	1,04E-06	0,023093627
chr2	65100000	65350000	1	1,14E-06	0,023123365
chr2	65150000	65400000	1	5,02E-07	0,023005684
chr2	65200000	65450000	1	3,70E-05	0,022635288
chr2	65250000	65500000	1	0,001593485	0,021937756
chr2	65300000	65550000	1	0,019934732	0,020890884
chr2	66300000	66550000	1	0,013944795	0,026705288
chr2	66350000	66600000	1	0,000253487	0,02633967
chr2	66400000	66650000	1	0,000159212	0,026320005
chr2	66450000	66700000	1	0,000122249	0,026320005
chr2	66500000	66750000	1	0,001576484	0,026320005
chr2	67000000	67250000	1	0,022946675	0,048777813
chr2	67050000	67300000	1	0,004100824	0,051851346
chr2	67600000	67850000	1	0,029277006	0,017643978
chr2	67650000	67900000	1	0,024744372	0,018402147
chr2	67750000	68000000	1	0,026600519	0,018634665
chr2	67800000	68050000	1	0,005092501	0,017712456
chr2	69850000	70100000	1	0,021062537	0,006702854
chr2	77400000	77650000	1	0,00097352	0,052854717
chr2	77450000	77700000	1	0,003223115	0,051853938
chr2	77500000	77750000	1	0,028501759	0,049292556
chr2	88900000	89150000	1	0,042378284	0,026479446
chr2	89350000	89600000	1	0,034576902	0,01269114
chr2	89400000	89650000	1	0,000987477	0,012980688
chr2	89450000	89700000	1	0,002720773	0,013161413
chr2	89500000	89750000	1	0,002080504	0,013103683
chr2	89550000	89800000	1	0,000525085	0,012727442
chr2	89600000	89850000	1	0,015950998	0,012727442
chr2	112750000	113000000	1	0,014884543	0,019904906
chr2	112800000	113050000	1	0,003417533	0,021224629

chr2	112850000	113100000	1	0,043542257	0,021882158
chr2	119350000	119600000	1	0,033410151	0,033384926
chr2	144200000	144450000	1	0,019434114	0,04040141
chr2	159350000	159600000	1	0,038564493	0,004381708
chr2	160600000	160850000	1	0,006313183	0,01455895
chr2	171900000	172150000	1	0,026927612	0,019428615
chr2	184950000	185200000	1	0,032154593	0,036024884
chr2	185000000	185250000	1	0,002979515	0,037822631
chr2	185050000	185300000	1	0,001888487	0,038093833
chr2	190700000	190950000	1	0,011702308	0,030385097
chr2	190750000	191000000	1	0,001892556	0,030385097
chr2	190800000	191050000	1	0,02387135	0,030385097
chr2	203050000	203300000	1	0,017292728	0,002958333
chr2	203250000	203500000	1	0,008358019	0,002749714
chr2	203300000	203550000	1	0,033634872	0,003002754
chr2	208400000	208650000	1	0,049932498	0,024344944
chr2	219700000	219950000	1	0,027338251	0,014233536
chr2	228350000	228600000	1	0,035567921	0,008984106
chr2	232550000	232800000	1	0,027982301	0,00340604
chr2	233750000	234000000	1	0,011511037	0,020997577
chr2	233800000	234050000	1	0,035190481	0,020997577
chr2	238600000	238850000	1	0,012283861	0,013010223
chr2	238650000	238900000	1	0,038103558	0,013901107
chr3	9800000	10050000	1	0,007354821	0,006189297
chr3	9850000	10100000	1	0,002873306	0,006552971
chr3	12300000	12550000	1	0,002536246	0,00931304
chr3	12350000	12600000	1	0,006673095	0,009790274
chr3	12400000	12650000	1	0,043240047	0,009886323
chr3	15300000	15550000	1	0,019647388	0,01609824
chr3	15350000	15600000	1	0,009246943	0,017079347
chr3	24900000	25150000	1	0,043224714	0,037791702
chr3	24950000	25200000	1	0,011938776	0,035631153
chr3	32950000	33200000	1	0,021631904	0,024135259
chr3	33000000	33250000	1	0,004387013	0,025074365
chr3	33050000	33300000	1	0,000312518	0,025868972
chr3	33100000	33350000	1	0,000891029	0,026471059
chr3	33150000	33400000	1	0,017061148	0,024380543
chr3	47750000	48000000	1	0,004737125	0,00342746
chr3	47800000	48050000	1	0,003062225	0,003632181
chr3	47850000	48100000	1	0,01776204	0,003691017
chr3	49800000	50050000	1	0,013322416	0,005591706
chr3	49850000	50100000	1	0,000376515	0,005889758
chr3	49900000	50150000	1	0,000191207	0,006056992
chr3	49950000	50200000	1	0,002184341	0,006121289
chr3	50000000	50250000	1	0,008592077	0,005619301
chr3	50050000	50300000	1	0,019559493	0,00544365

chr3	50100000	50350000	1	0,032065818	0,005268551
chr3	50150000	50400000	1	0,037028098	0,005125615
chr3	50200000	50450000	1	0,023033285	0,005069806
chr3	50250000	50500000	1	0,004353092	0,005167346
chr3	50300000	50550000	1	0,005288574	0,005462727
chr3	52650000	52900000	1	0,026561116	0,010038659
chr3	60350000	60600000	1	0,043032437	0,067942317
chr3	60400000	60650000	1	0,005763396	0,064510312
chr3	60750000	61000000	1	0,005635656	0,050289913
chr3	60800000	61050000	1	0,000324292	0,047516403
chr3	60850000	61100000	1	0,00069941	0,046581366
chr3	98550000	98800000	1	0,02926135	0,035575481
chr3	101600000	101850000	1	0,030582679	0,039159536
chr3	101650000	101900000	1	0,046112372	0,036021219
chr3	104650000	104900000	1	0,025256256	0,029021199
chr3	104700000	104950000	1	0,016533994	0,03103735
chr3	105450000	105700000	1	0,024622503	0,017944386
chr3	107800000	108050000	1	0,009732715	0,026654398
chr3	124800000	125050000	1	0,025006435	0,019249426
chr3	124850000	125100000	1	0,004623508	0,018323567
chr3	127500000	127750000	1	0,005194883	0,022426812
chr3	127550000	127800000	1	0,008742791	0,021148625
chr3	132000000	132250000	1	0,024862524	0,008744746
chr3	133500000	133750000	1	0,044308419	0,015353588
chr3	133650000	133900000	1	0,034336538	0,015054199
chr3	133700000	133950000	1	0,033360647	0,016081185
chr3	141200000	141450000	1	0,027986749	0,015369008
chr3	177750000	178000000	1	0,042985646	0,027142324
chr3	177800000	178050000	1	0,004965866	0,025971386
chr3	183500000	183750000	1	0,027354221	0,017825858
chr3	183550000	183800000	1	0,007900028	0,019064946
chr3	185050000	185300000	1	0,018284135	0,020626766
chr3	191000000	191250000	1	0,012457634	0,007931681
chr3	191050000	191300000	1	0,004804838	0,008323104
chr3	191750000	192000000	1	0,047989233	0,018889375
chr3	191800000	192050000	1	0,046318406	0,020351676
chr3	196350000	196600000	1	0,048535644	0,007404744
chr3	197000000	197250000	1	0,034900524	0,009882963
chr3	197550000	197800000	1	0,009641683	0,011107671
chr4	2900000	3150000	1	0,017085244	0,016553687
chr4	2950000	3200000	1	0,028152346	0,015523788
chr4	66600000	66850000	1	0,00446977	0,017911114
chr4	66650000	66900000	1	0,022967006	0,017911114
chr4	76850000	77100000	1	0,00639846	0,017788506
chr4	83600000	83850000	1	0,011001834	0,013497043
chr4	83650000	83900000	1	0,047793772	0,014362797

chr4	83950000	84200000	1	0,029519551	0,010579489
chr4	84250000	84500000	1	0,02195496	0,012096866
chr4	100550000	100800000	1	0,015928095	0,042606377
chr4	100600000	100850000	1	0,002256126	0,045555421
chr4	100650000	100900000	1	0,000222458	0,045723755
chr4	100700000	100950000	1	0,005893603	0,043546915
chr4	103600000	103850000	1	0,037718176	0,010025465
chr4	114600000	114850000	1	0,005183298	0,013847468
chr4	114650000	114900000	1	0,008880913	0,013699762
chr4	120600000	120850000	1	0,003194525	0,061330957
chr4	120650000	120900000	1	0,010455522	0,058102953
chr4	124950000	125200000	1	0,025906136	0,038871706
chr4	129050000	129300000	1	0,017634822	0,01427954
chr4	140100000	140350000	1	0,048159514	0,015141975
chr4	152050000	152300000	1	0,044572731	0,025571137
chr4	152900000	153150000	1	0,015530539	0,035166331
chr4	174100000	174350000	1	0,047784266	0,019141228
chr4	175150000	175400000	1	0,025884561	0,016574246
chr4	175200000	175450000	1	0,027766359	0,015253339
chr4	177550000	177800000	1	0,013862367	0,040379704
chr4	177600000	177850000	1	0,040621019	0,03671328
chr5	32550000	32800000	1	0,011542962	0,006711666
chr5	55900000	56150000	1	0,003624477	0,018121255
chr5	55950000	56200000	1	0,00029796	0,018900754
chr5	56000000	56250000	1	0,001636406	0,019222874
chr5	56050000	56300000	1	0,01180603	0,019286542
chr5	56100000	56350000	1	0,027796533	0,019365091
chr5	56150000	56400000	1	0,017075751	0,019916485
chr5	56200000	56450000	1	0,003997737	0,020562525
chr5	62750000	63000000	1	0,024062943	0,004402375
chr5	62800000	63050000	1	0,01908748	0,004542623
chr5	62850000	63100000	1	0,022292503	0,004542623
chr5	62900000	63150000	1	0,0130858	0,004542623
chr5	62950000	63200000	1	0,002406908	0,004542623
chr5	63000000	63250000	1	9,88E-05	0,004542623
chr5	63050000	63300000	1	1,49E-05	0,004646952
chr5	63100000	63350000	1	0,004202103	0,004674922
chr5	91700000	91950000	1	0,005229298	0,004777537
chr5	95350000	95600000	1	0,007533959	0,0247301
chr5	102250000	102500000	1	0,000870519	0,016292916
chr5	102300000	102550000	1	0,000801879	0,015930763
chr5	102350000	102600000	1	0,012461281	0,015211577
chr5	111200000	111450000	1	0,007050306	0,022052262
chr5	111250000	111500000	1	0,007259464	0,022210159
chr5	123600000	123850000	1	0,011527882	0,022582672
chr5	123650000	123900000	1	0,000686554	0,023552003

chr5	123700000	123950000	1	0,000542702	0,024272961
chr5	123750000	124000000	1	0,002673786	0,024631464
chr5	123800000	124050000	1	0,002425254	0,024562684
chr5	123850000	124100000	1	0,00041241	0,024147205
chr5	123900000	124150000	1	0,000113963	0,023874769
chr5	123950000	124200000	1	0,000123581	0,023662259
chr5	124000000	124250000	1	0,003031912	0,022341131
chr5	126900000	127150000	1	0,007583647	0,032507286
chr5	127400000	127650000	1	0,009846723	0,022087323
chr5	131900000	132150000	1	0,028539469	0,002617466
chr5	132150000	132400000	1	0,022927821	0,002168914
chr5	137500000	137750000	1	0,008178948	0,012428853
chr5	137550000	137800000	1	0,012609889	0,013168212
chr5	139600000	139850000	1	0,041563649	0,015841578
chr5	139650000	139900000	1	0,003852825	0,015160127
chr5	142550000	142800000	1	0,031556207	0,017265148
chr5	142600000	142850000	1	0,00772041	0,018658456
chr5	179600000	179850000	1	0,025627686	0,015058811
chr6	9200000	9450000	1	0,025851447	0,023504654
chr6	9250000	9500000	1	0,03531835	0,02524565
chr6	11200000	11450000	1	0,03944196	0,03484604
chr6	11250000	11500000	1	0,015501363	0,035168492
chr6	11300000	11550000	1	0,002542148	0,034916939
chr6	11350000	11600000	1	0,000337672	0,034038276
chr6	11400000	11650000	1	0,006245706	0,032572033
chr6	17800000	18050000	1	0,043100359	0,008459229
chr6	22100000	22350000	1	0,041881216	0,027962852
chr6	31050000	31300000	1	0,016914059	0,01955715
chr6	31100000	31350000	1	0,031570637	0,01955715
chr6	32900000	33150000	1	0,036834772	0,016419352
chr6	34950000	35200000	1	0,024105835	0,014329849
chr6	35700000	35950000	1	0,048304828	0,016723802
chr6	35750000	36000000	1	0,004393812	0,015970518
chr6	41850000	42100000	1	0,049422124	0,006693237
chr6	42400000	42650000	1	0,010467807	0,01795877
chr6	47000000	47250000	1	0,031048159	0,030317082
chr6	112200000	112450000	1	0,011566692	0,029661635
chr6	113150000	113400000	1	0,032896792	0,022867023
chr6	113200000	113450000	1	0,005693644	0,024491852
chr6	140650000	140900000	1	0,017107422	0,050294063
chr6	140700000	140950000	1	0,004643172	0,04782735
chr6	156000000	156250000	1	0,040950285	0,019634993
chr6	158550000	158800000	1	0,019030645	0,008612261
chr6	158700000	158950000	1	0,02320664	0,008250048
chr6	158750000	159000000	1	0,009510118	0,008250048
chr6	158900000	159150000	1	0,027789418	0,008462155

chr6	158950000	159200000	1	0,014271967	0,008913416
chr7	5750000	6000000	1	0,038117116	0,014851957
chr7	6200000	6450000	1	0,014942865	0,00597675
chr7	15000000	15250000	1	0,046768716	0,074064675
chr7	18600000	18850000	1	0,033764185	0,009065621
chr7	22750000	23000000	1	0,04567921	0,013008363
chr7	22800000	23050000	1	0,004939127	0,013819133
chr7	22850000	23100000	1	0,00036243	0,014298753
chr7	22900000	23150000	1	0,009750237	0,014607499
chr7	40500000	40750000	1	0,035169433	0,029192082
chr7	40550000	40800000	1	0,000764604	0,031307021
chr7	40600000	40850000	1	0,000103202	0,032462713
chr7	40650000	40900000	1	0,000339551	0,032810795
chr7	40700000	40950000	1	0,0002443	0,032632798
chr7	40750000	41000000	1	7,07E-05	0,032242601
chr7	40800000	41050000	1	3,46E-05	0,031888782
chr7	40850000	41100000	1	7,43E-05	0,031675616
chr7	40900000	41150000	1	0,000116317	0,030722371
chr7	40950000	41200000	1	4,10E-05	0,031023226
chr7	41000000	41250000	1	0,000550838	0,032058672
chr7	41050000	41300000	1	0,022090485	0,03394207
chr7	41400000	41650000	1	0,007762395	0,045030667
chr7	41450000	41700000	1	0,002413547	0,043232658
chr7	41500000	41750000	1	0,041417526	0,040714066
chr7	43750000	44000000	1	0,001824825	0,001932069
chr7	65550000	65800000	1	0,009869806	0,025321315
chr7	65600000	65850000	1	0,009822749	0,025321315
chr7	66150000	66400000	1	0,016856401	0,01044852
chr7	66200000	66450000	1	0,000424229	0,010138353
chr7	73200000	73450000	1	0,038944996	0,01375373
chr7	75400000	75650000	1	0,013881488	0,020157095
chr7	78950000	79200000	1	0,033709689	0,058256663
chr7	79000000	79250000	1	0,001545698	0,0628695
chr7	79050000	79300000	1	0,014061482	0,059887585
chr7	79600000	79850000	1	0,004728975	0,036657896
chr7	79650000	79900000	1	0,000160186	0,034992371
chr7	79700000	79950000	1	0,007235331	0,034309579
chr7	83150000	83400000	1	0,004322602	0,03065187
chr7	83200000	83450000	1	3,63E-05	0,032170287
chr7	83250000	83500000	1	2,76E-06	0,032711223
chr7	83300000	83550000	1	5,31E-06	0,032767671
chr7	83350000	83600000	1	6,57E-06	0,032754503
chr7	83400000	83650000	1	0,00083085	0,032873694
chr7	83450000	83700000	1	0,026967978	0,033076156
chr7	99600000	99850000	1	0,025891661	0,003308586
chr7	99650000	99900000	1	0,002498862	0,003663025

chr7	99700000	99950000	1	0,001935696	0,003703217
chr7	99750000	100000000	1	0,025041713	0,003606842
chr7	100450000	100700000	1	0,016025586	0,019086364
chr7	100500000	100750000	1	0,002803054	0,019086364
chr7	100550000	100800000	1	0,000266011	0,019086364
chr7	100600000	100850000	1	0,000196616	0,019437903
chr7	100650000	100900000	1	0,007159239	0,019681955
chr7	100950000	101200000	1	0,035320168	0,016049093
chr7	101000000	101250000	1	0,001087875	0,015091044
chr7	101050000	101300000	1	1,78E-05	0,014505953
chr7	101100000	101350000	1	0,007031352	0,014008978
chr7	114750000	115000000	1	0,048190441	0,015350247
chr7	129000000	129250000	1	0,02244833	0,020575552
chr7	130200000	130450000	1	0,017556945	0,007030965
chr7	130250000	130500000	1	0,000507382	0,007030965
chr7	130300000	130550000	1	0,000338957	0,007248195
chr7	130350000	130600000	1	0,018873966	0,007308481
chr7	130950000	131200000	1	0,021210285	0,016749549
chr7	131000000	131250000	1	0,001195585	0,016463124
chr7	131050000	131300000	1	0,000848861	0,01601185
chr7	131100000	131350000	1	0,009421244	0,015463467
chr7	131150000	131400000	1	0,03937157	0,014808412
chr7	143350000	143600000	1	0,002770262	0,040356908
chr7	143400000	143650000	1	0,000140129	0,040356908
chr7	143450000	143700000	1	0,000177488	0,040356908
chr7	143500000	143750000	1	0,004568069	0,039936826
chr7	143750000	144000000	1	0,015016908	0,044655051
chr7	143800000	144050000	1	0,027769456	0,04722668
chrX	46200000	46450000	1	0,003531026	0,016830775
chrX	46250000	46500000	1	0,001756934	0,017806451
chrX	46300000	46550000	1	0,003112658	0,018012995
chrX	46350000	46600000	1	0,000715227	0,017750859
chrX	46400000	46650000	1	0,000556368	0,017340319
chrX	46450000	46700000	1	0,009155604	0,017032954
chrX	46500000	46750000	1	0,044578704	0,016974303
chrX	46700000	46950000	1	0,019184095	0,021152406
chrX	46750000	47000000	1	0,001807378	0,020654108
chrX	46800000	47050000	1	0,006439858	0,019568295
chrX	53050000	53300000	1	0,023729633	0,013813385
chrX	53100000	53350000	1	0,006059833	0,01483963
chrX	53150000	53400000	1	0,04629598	0,0153868
chrX	53300000	53550000	1	0,01828053	0,012980627
chrX	53350000	53600000	1	0,001711685	0,012939412
chrX	53400000	53650000	1	0,000294919	0,012699972
chrX	53450000	53700000	1	0,006118161	0,012080855
chrX	104100000	104350000	1	0,043291222	0,0110765

chrX	104650000	104900000	1	0,00592834	0,018531723
chrX	139950000	140200000	1	0,019362127	0,017865884
chrX	140150000	140400000	1	0,028019598	0,018416626
chrX	140600000	140850000	1	0,020765558	0,031546058
chrX	140650000	140900000	1	0,000831125	0,033309707
chrX	140700000	140950000	1	0,001385676	0,034371605
chr8	7750000	8000000	1	0,019901642	0,029826864
chr8	7800000	8050000	1	0,018255125	0,031900465
chr8	30000000	30250000	1	0,027168089	0,043101751
chr8	42300000	42550000	1	0,02763792	0,033107233
chr8	42350000	42600000	1	0,019682122	0,030748782
chr8	77300000	77550000	1	0,013510013	0,059091524
chr8	87050000	87300000	1	0,032776423	0,041429891
chr8	94600000	94850000	1	0,020508501	0,019813327
chr8	94650000	94900000	1	0,000397645	0,018213785
chr8	94700000	94950000	1	0,016903554	0,017818435
chr8	101550000	101800000	1	0,014436474	0,026418826
chr8	101600000	101850000	1	0,000966867	0,027700813
chr8	101650000	101900000	1	0,005877545	0,028623975
chr8	107950000	108200000	1	0,011272497	0,03224485
chr8	108000000	108250000	1	0,000280464	0,031652763
chr8	108050000	108300000	1	0,001754729	0,030923383
chr8	108100000	108350000	1	0,010026151	0,029888026
chr8	118800000	119050000	1	0,038560132	0,012923163
chr8	119050000	119300000	1	0,048709994	0,015111152
chr8	119100000	119350000	1	0,013721398	0,015331565
chr8	119150000	119400000	1	0,003913113	0,015490058
chr8	119200000	119450000	1	0,00109722	0,015584853
chr8	119250000	119500000	1	0,000272345	0,015653938
chr8	119300000	119550000	1	0,00011145	0,015653938
chr8	119350000	119600000	1	0,000561343	0,015653938
chr8	119400000	119650000	1	0,009347924	0,015653938
chr8	119850000	120100000	1	0,033151717	0,028928742
chr8	119900000	120150000	1	0,003116374	0,027704113
chr8	141950000	142200000	1	0,010632042	0,01276773
chr8	142000000	142250000	1	0,003307197	0,012379096
chr8	142050000	142300000	1	0,025656281	0,011869455
chr9	0	250000	1	5,06E-05	0,02119322
chr9	50000	300000	1	1,26E-05	0,02106212
chr9	100000	350000	1	7,27E-05	0,020873299
chr9	150000	400000	1	0,0002176	0,020742351
chr9	200000	450000	1	0,000131431	0,020773167
chr9	250000	500000	1	0,0103943	0,019550666
chr9	33400000	33650000	1	0,011230174	0,00728277
chr9	33450000	33700000	1	0,009093171	0,006847603
chr9	35600000	35850000	1	0,023851375	0,002207084

chr9	35650000	35900000	1	0,000883372	0,002413901
chr9	35700000	35950000	1	0,00093831	0,002469489
chr9	35750000	36000000	1	0,003049582	0,002438251
chr9	36050000	36300000	1	0,02332881	0,002774718
chr9	40600000	40850000	1	0,042723836	0,034368832
chr9	40650000	40900000	1	0,002388037	0,037038398
chr9	40700000	40950000	1	0,003000921	0,038225955
chr9	40750000	41000000	1	0,002640762	0,03773537
chr9	40800000	41050000	1	0,040275919	0,036609075
chr9	41450000	41700000	1	0,022034007	0,101772772
chr9	67000000	67250000	1	0,041149196	0,039279283
chr9	67400000	67650000	1	0,027354876	0,068049571
chr9	74950000	75200000	1	0,014938996	0,039552846
chr9	95450000	95700000	1	0,033433126	0,011747906
chr9	95700000	95950000	1	0,007860675	0,010622886
chr9	95750000	96000000	1	0,037903786	0,011159096
chr9	95850000	96100000	1	0,02224914	0,010328184
chr9	100050000	100300000	1	0,020402199	0,019670206
chr9	100100000	100350000	1	0,013271335	0,021061762
chr9	114450000	114700000	1	0,003981958	0,016334297
chr9	114500000	114750000	1	0,000379416	0,017176398
chr9	114550000	114800000	1	0,001653742	0,017511824
chr9	114600000	114850000	1	0,00132253	0,017459969
chr9	114650000	114900000	1	0,000233088	0,016987366
chr9	114700000	114950000	1	0,002206407	0,017627514
chr9	114750000	115000000	1	0,03156235	0,01853442
chr9	115050000	115300000	1	0,040779706	0,023384734
chr9	115100000	115350000	1	0,003625675	0,02262677
chr9	115150000	115400000	1	0,026331555	0,020942161
chr9	123750000	124000000	1	0,011639194	0,004620397
chr9	123800000	124050000	1	0,001294011	0,004448531
chr9	126800000	127050000	1	0,040559877	0,021386095
chr9	126850000	127100000	1	0,000581254	0,023205004
chr9	126900000	127150000	1	0,000725945	0,024051559
chr9	126950000	127200000	1	0,001791068	0,024169753
chr9	127000000	127250000	1	0,001880825	0,023880225
chr9	127050000	127300000	1	0,003587724	0,023478657
chr9	127100000	127350000	1	0,020606112	0,023139104
chr9	127600000	127850000	1	0,027802733	0,0097788
chr9	127650000	127900000	1	0,01521456	0,010331876
chr9	127850000	128100000	1	0,024991253	0,01068822
chr9	129550000	129800000	1	0,012146194	0,012354864
chr9	129600000	129850000	1	0,008371607	0,013216776
chr9	130600000	130850000	1	0,049399472	0,005276378
chr9	130650000	130900000	1	0,001865364	0,0057843
chr9	130700000	130950000	1	0,000957815	0,005703198

chr9	130750000	131000000	1	0,014763596	0,005459426
chr9	133000000	133250000	1	0,010207312	0,012695477
chr9	133050000	133300000	1	0,000341969	0,012695477
chr9	133100000	133350000	1	0,002738044	0,012695477
chr9	133150000	133400000	1	0,012941967	0,012715574
chr9	133200000	133450000	1	0,035613015	0,012554752
chr9	139600000	139850000	1	0,045083558	0,001929085
chr10	8600000	8850000	1	0,01775155	0,016425784
chr10	8650000	8900000	1	0,001110711	0,016285275
chr10	8700000	8950000	1	8,68E-05	0,016021323
chr10	8750000	9000000	1	0,000902755	0,015690526
chr10	8800000	9050000	1	0,004119344	0,015336845
chr10	8850000	9100000	1	0,008446972	0,014980698
chr10	8900000	9150000	1	0,012496494	0,014596002
chr10	8950000	9200000	1	0,020488331	0,01410001
chr10	10150000	10400000	1	0,047327947	0,018637171
chr10	16550000	16800000	1	0,018311185	0,004532525
chr10	28900000	29150000	1	0,027489581	0,018843282
chr10	28950000	29200000	1	0,006596231	0,019190816
chr10	29000000	29250000	1	0,004191432	0,019190816
chr10	29050000	29300000	1	0,003402774	0,019190816
chr10	29100000	29350000	1	0,000997677	0,019190816
chr10	29150000	29400000	1	0,002495202	0,019190816
chr10	45600000	45850000	1	0,009006713	0,00409796
chr10	45650000	45900000	1	0,045934285	0,004416209
chr10	63750000	64000000	1	0,014240199	0,021514565
chr10	63800000	64050000	1	0,035877342	0,020040643
chr10	70250000	70500000	1	0,002544862	0,009214636
chr10	70300000	70550000	1	2,04E-05	0,009676969
chr10	70350000	70600000	1	4,71E-05	0,009780519
chr10	70400000	70650000	1	0,000235401	0,00968489
chr10	70450000	70700000	1	0,000244924	0,009591659
chr10	70500000	70750000	1	0,004291681	0,009628505
chr10	73950000	74200000	1	0,006888045	0,005688699
chr10	75000000	75250000	1	0,014256486	0,013499865
chr10	88600000	88850000	1	0,005224054	0,018200685
chr10	88650000	88900000	1	2,21E-05	0,018329663
chr10	88700000	88950000	1	0,000351206	0,018902653
chr10	88750000	89000000	1	0,017018352	0,019983274
chr10	89050000	89300000	1	0,015906104	0,023116132
chr10	89100000	89350000	1	0,028896478	0,024564899
chr10	89500000	89750000	1	0,010747955	0,013089426
chr10	89550000	89800000	1	4,31E-05	0,012948154
chr10	89600000	89850000	1	0,000756772	0,012492408
chr10	89650000	89900000	1	0,024741839	0,011765026
chr10	89950000	90200000	1	0,036466375	0,009009252

chr10	90600000	90850000	1	0,002465589	0,035897457
chr10	90650000	90900000	1	0,006875265	0,037522693
chr10	90950000	91200000	1	0,029400168	0,031734901
chr10	91000000	91250000	1	0,008660844	0,027808874
chr10	91050000	91300000	1	0,011397835	0,026691288
chr10	94600000	94850000	1	0,00426814	0,013041215
chr10	95900000	96150000	1	0,040137334	0,010028882
chr10	102850000	103100000	1	0,019261165	0,009287986
chr10	102900000	103150000	1	0,007236767	0,008880129
chr10	102950000	103200000	1	0,040498862	0,008580498
chr10	103000000	103250000	1	0,042868462	0,008580498
chr10	103050000	103300000	1	0,008548905	0,008580498
chr10	103100000	103350000	1	0,025093077	0,008580498
chr10	103350000	103600000	1	0,021514314	0,010276147
chr10	103400000	103650000	1	0,016420357	0,010951335
chr10	104350000	104600000	1	0,007036813	0,018820979
chr10	104400000	104650000	1	0,00038375	0,018370035
chr10	104450000	104700000	1	0,005085613	0,018370035
chr10	104500000	104750000	1	0,0416211	0,018370035
chr10	104900000	105150000	1	0,020904227	0,01167414
chr10	104950000	105200000	1	0,005114011	0,011425888
chr10	105000000	105250000	1	0,010808019	0,010936192
chr10	105550000	105800000	1	0,040747525	0,007904708
chr10	116200000	116450000	1	0,007437729	0,031115773
chr10	116250000	116500000	1	0,017018212	0,029044878
chr10	117500000	117750000	1	0,040180522	0,025012692
chr10	121050000	121300000	1	0,033834249	0,006261186
chr11	8750000	9000000	1	0,039252146	0,015081839
chr11	8800000	9050000	1	0,009380908	0,016375715
chr11	10550000	10800000	1	0,005725132	0,009937564
chr11	10600000	10850000	1	0,007676926	0,010403719
chr11	12300000	12550000	1	0,025798088	0,02270653
chr11	12350000	12600000	1	0,00167364	0,024179686
chr11	12400000	12650000	1	0,028963297	0,02263156
chr11	14350000	14600000	1	0,029560052	0,029610928
chr11	45850000	46100000	1	0,049762941	0,013053135
chr11	45900000	46150000	1	0,00105264	0,014098862
chr11	45950000	46200000	1	8,79E-05	0,014645968
chr11	46000000	46250000	1	0,000571549	0,014822418
chr11	46050000	46300000	1	0,001199255	0,014772673
chr11	46100000	46350000	1	0,001567578	0,014615365
chr11	46150000	46400000	1	0,002344417	0,014424223
chr11	46200000	46450000	1	0,006137418	0,014229215
chr11	46250000	46500000	1	0,028242597	0,014035991
chr11	46800000	47050000	1	0,001499493	0,005026812
chr11	46850000	47100000	1	0,004694521	0,005026812

chr11	46900000	47150000	1	0,004329283	0,005026812
chr11	46950000	47200000	1	0,001338221	0,005144789
chr11	47000000	47250000	1	0,00078062	0,005266812
chr11	47050000	47300000	1	0,004738644	0,005345327
chr11	47100000	47350000	1	0,036926129	0,005384044
chr11	47350000	47600000	1	0,010957537	0,007006722
chr11	47400000	47650000	1	0,003226998	0,007006722
chr11	61550000	61800000	1	0,012064798	0,005480719
chr11	64400000	64650000	1	0,013871593	0,002136695
chr11	64500000	64750000	1	0,038340051	0,002232272
chr11	64550000	64800000	1	0,018286028	0,002145918
chr11	65050000	65300000	1	0,01183303	0,004990453
chr11	66950000	67200000	1	0,027834716	0,004592453
chr11	67000000	67250000	1	0,001871586	0,004522266
chr11	67050000	67300000	1	0,000845659	0,004382244
chr11	67100000	67350000	1	0,011701619	0,004211254
chr11	67150000	67400000	1	0,043137951	0,004062844
chr11	67250000	67500000	1	0,018449481	0,004001597
chr11	67300000	67550000	1	0,001653477	0,004096257
chr11	67350000	67600000	1	0,002006489	0,004096257
chr11	67400000	67650000	1	0,010783934	0,004096257
chr11	67450000	67700000	1	0,01222428	0,004096257
chr11	67500000	67750000	1	0,003225004	0,004096257
chr11	67550000	67800000	1	0,00021769	0,004222846
chr11	67600000	67850000	1	0,000241655	0,004326018
chr11	67650000	67900000	1	0,00051477	0,004366925
chr11	67700000	67950000	1	0,000159149	0,004340414
chr11	67750000	68000000	1	0,000288602	0,004275325
chr11	67800000	68050000	1	0,004171772	0,00421873
chr11	67850000	68100000	1	0,019423035	0,004208954
chr11	77450000	77700000	1	0,005896317	0,006744876
chr11	122800000	123050000	1	0,020850994	0,011919552
chr11	122850000	123100000	1	0,004368796	0,011287508
chr11	128100000	128350000	1	0,015748518	0,031407709
chr11	128150000	128400000	1	0,011584844	0,033711542
chr11	130000000	130250000	1	0,018762839	0,010380231
chr11	130050000	130300000	1	0,002727496	0,009963793
chr11	130100000	130350000	1	0,044010533	0,009190397
chr12	600000	850000	1	0,004742582	0,006441908
chr12	650000	900000	1	0,004035998	0,00647184
chr12	700000	950000	1	0,005117138	0,006277228
chr12	2700000	2950000	1	0,042811122	0,003248285
chr12	2750000	3000000	1	4,50E-05	0,00363248
chr12	2800000	3050000	1	0,00037349	0,003692112
chr12	2850000	3100000	1	0,000633252	0,003634104
chr12	2900000	3150000	1	0,010778204	0,00360603

chr12	12800000	13050000	1	0,046147702	0,005520832
chr12	14900000	15150000	1	0,047482933	0,025667413
chr12	14950000	15200000	1	0,00163204	0,028019494
chr12	15000000	15250000	1	0,00966507	0,026754569
chr12	27500000	27750000	1	0,03278037	0,014813993
chr12	39100000	39350000	1	0,041692198	0,041224427
chr12	39150000	39400000	1	0,001852483	0,040219712
chr12	39200000	39450000	1	0,005403068	0,038820113
chr12	43350000	43600000	1	0,047185537	0,031339292
chr12	43400000	43650000	1	0,024079335	0,028789893
chr12	49350000	49600000	1	0,035762337	0,006027673
chr12	49400000	49650000	1	0,002801386	0,006431318
chr12	49450000	49700000	1	0,027685025	0,006698479
chr12	49650000	49900000	1	0,049098433	0,0054758
chr12	49700000	49950000	1	0,008055265	0,005778208
chr12	50000000	50250000	1	0,039761653	0,007291423
chr12	50050000	50300000	1	0,002580156	0,007291423
chr12	50100000	50350000	1	0,001467115	0,007291423
chr12	50150000	50400000	1	0,020581963	0,007445055
chr12	50350000	50600000	1	0,012886615	0,006747893
chr12	50400000	50650000	1	0,000677434	0,006443694
chr12	50450000	50700000	1	0,000556088	0,006234493
chr12	50500000	50750000	1	0,000474553	0,006279624
chr12	50550000	50800000	1	0,012813923	0,00584119
chr12	53300000	53550000	1	0,011427625	0,006029401
chr12	53450000	53700000	1	0,030122583	0,006096375
chr12	53500000	53750000	1	0,015910793	0,005989962
chr12	56150000	56400000	1	0,033244617	0,006731298
chr12	56400000	56650000	1	0,048165436	0,005982237
chr12	56450000	56700000	1	0,002751934	0,005617787
chr12	56500000	56750000	1	0,000105815	0,005400905
chr12	56550000	56800000	1	0,000514416	0,005133944
chr12	56600000	56850000	1	0,005536875	0,004938192
chr12	56650000	56900000	1	0,019416904	0,00479464
chr12	56700000	56950000	1	0,019155442	0,004760673
chr12	56750000	57000000	1	0,004195226	0,004908373
chr12	56800000	57050000	1	0,019450478	0,005271936
chr12	57150000	57400000	1	0,014275775	0,007733551
chr12	58850000	59100000	1	0,038472946	0,020286775
chr12	58900000	59150000	1	0,005394383	0,01932397
chr12	60250000	60500000	1	0,003792148	0,041314046
chr12	60300000	60550000	1	0,048109482	0,03812675
chr12	64650000	64900000	1	0,029284777	0,017847261
chr12	64700000	64950000	1	0,005599844	0,019027638
chr12	65950000	66200000	1	0,000708995	0,029700175
chr12	66000000	66250000	1	0,000769446	0,029405416

chr12	66050000	66300000	1	0,000806514	0,029416205
chr12	66100000	66350000	1	0,000114067	0,029577574
chr12	66150000	66400000	1	2,10E-06	0,029561246
chr12	66200000	66450000	1	4,01E-05	0,028945315
chr12	66250000	66500000	1	0,008366416	0,027301261
chr12	74600000	74850000	1	0,014452906	0,014261063
chr12	74800000	75050000	1	0,028100898	0,012839824
chr12	74850000	75100000	1	0,001741988	0,012888073
chr12	74900000	75150000	1	2,24E-05	0,012780729
chr12	74950000	75200000	1	0,000501721	0,01237528
chr12	75000000	75250000	1	0,021557924	0,011633562
chr12	78400000	78650000	1	0,02891677	0,036242711
chr12	78450000	78700000	1	0,001079642	0,038532489
chr12	78500000	78750000	1	0,004054882	0,03990398
chr12	90600000	90850000	1	0,017141475	0,018161756
chr12	90650000	90900000	1	0,003811721	0,017356524
chr12	94000000	94250000	1	0,013241756	0,045477966
chr12	94050000	94300000	1	0,019782937	0,042522014
chr12	95650000	95900000	1	0,045204133	0,019817994
chr12	95950000	96200000	1	0,027448433	0,016523352
chr12	96000000	96250000	1	0,027358474	0,017254125
chr12	96050000	96300000	1	0,048668422	0,017278607
chr12	96100000	96350000	1	0,022139306	0,016021674
chr12	105250000	105500000	1	0,010123571	0,023627764
chr12	109800000	110050000	1	0,046234282	0,014020276
chr12	111850000	112100000	1	0,002704664	0,010937005
chr12	111900000	112150000	1	0,004202393	0,011472844
chr12	111950000	112200000	1	0,002080145	0,011461007
chr12	112000000	112250000	1	0,001201887	0,011168203
chr12	112050000	112300000	1	0,005741782	0,010880807
chr12	112100000	112350000	1	0,004270155	0,010809916
chr12	112150000	112400000	1	0,017117483	0,011014644
chr12	117250000	117500000	1	0,037421577	0,013358807
chr12	120750000	121000000	1	0,039488339	0,009595081
chr12	121100000	121350000	1	0,008147948	0,013269333
chr12	121150000	121400000	1	0,00022128	0,013100421
chr12	121200000	121450000	1	0,000449623	0,013100421
chr12	121250000	121500000	1	0,008523195	0,013100421
chr12	122950000	123200000	1	0,042301975	0,002823883
chr13	23900000	24150000	1	0,017391606	0,018789629
chr13	38100000	38350000	1	0,005544972	0,035702923
chr13	38150000	38400000	1	0,019302072	0,0335804
chr13	39300000	39550000	1	0,007474472	0,021451209
chr13	97650000	97900000	1	0,035423161	0,023383861
chr13	98150000	98400000	1	0,010882313	0,011585285
chr13	98200000	98450000	1	0,004150084	0,011585285

chr13	104450000	104700000	1	0,014751326	0,013242299
chr14	19650000	19900000	1	0,049222516	0,01550294
chr14	19700000	19950000	1	0,005920352	0,014227669
chr14	19750000	20000000	1	0,008613699	0,014554462
chr14	19800000	20050000	1	0,023586099	0,013234059
chr14	22900000	23150000	1	0,013763797	0,029546292
chr14	22950000	23200000	1	0,018398207	0,031143193
chr14	50550000	50800000	1	0,021329994	0,005141781
chr14	50600000	50850000	1	0,040506008	0,005141781
chr14	53150000	53400000	1	0,010294727	0,005855984
chr14	55200000	55450000	1	0,047517544	0,010800722
chr14	64450000	64700000	1	0,03535709	0,014706184
chr14	64500000	64750000	1	0,020877391	0,014706184
chr14	67950000	68200000	1	0,015581631	0,017304427
chr14	68000000	68250000	1	0,000511868	0,017284518
chr14	68050000	68300000	1	5,03E-05	0,017284518
chr14	68100000	68350000	1	9,84E-05	0,017284518
chr14	68150000	68400000	1	3,08E-05	0,017284518
chr14	68200000	68450000	1	3,95E-05	0,017116807
chr14	68250000	68500000	1	0,000126824	0,016967255
chr14	68300000	68550000	1	6,54E-05	0,016967332
chr14	68350000	68600000	1	0,000569529	0,017125489
chr14	68400000	68650000	1	0,011046624	0,01732834
chr14	68450000	68700000	1	0,049846696	0,017395718
chr14	68550000	68800000	1	0,028387352	0,015058119
chr14	68600000	68850000	1	0,002874101	0,015500607
chr14	68650000	68900000	1	0,005834039	0,016156648
chr14	68700000	68950000	1	0,040867046	0,016818943
chr14	68800000	69050000	1	0,046483841	0,016818943
chr14	68850000	69100000	1	0,011913475	0,016818943
chr14	68900000	69150000	1	0,001069688	0,016818943
chr14	68950000	69200000	1	2,26E-05	0,017320534
chr14	69000000	69250000	1	9,30E-06	0,017583679
chr14	69050000	69300000	1	0,000623025	0,017639665
chr14	69100000	69350000	1	0,009196995	0,017603042
chr14	69150000	69400000	1	0,045223163	0,017626921
chr14	69350000	69600000	1	0,018985232	0,019928774
chr14	69400000	69650000	1	0,002007538	0,021846816
chr14	69450000	69700000	1	0,000150136	0,021431648
chr14	69500000	69750000	1	0,001878523	0,020806903
chr14	69550000	69800000	1	0,015407866	0,020009486
chr14	73550000	73800000	1	0,020899366	0,018006722
chr14	73600000	73850000	1	0,009570703	0,019276993
chr14	74750000	75000000	1	0,009853535	0,023627483
chr14	74800000	75050000	1	0,001319198	0,023627483
chr14	74850000	75100000	1	0,029796881	0,023627483

chr14	77900000	78150000	1	0,029191522	0,018755167
chr14	84200000	84450000	1	0,028819201	0,030683428
chr14	84700000	84950000	1	0,003086282	0,013125307
chr14	84750000	85000000	1	0,000495908	0,013177439
chr14	84800000	85050000	1	0,000349539	0,013026378
chr14	84850000	85100000	1	0,000303124	0,013026378
chr14	84900000	85150000	1	0,00498029	0,013026378
chr14	84950000	85200000	1	0,028939679	0,013026378
chr14	85100000	85350000	1	0,044782962	0,012275947
chr14	85150000	85400000	1	0,007347487	0,011777994
chr14	100250000	100500000	1	0,010348491	0,01503821
chr15	34500000	34750000	1	0,004085224	0,016103139
chr15	34550000	34800000	1	0,000417649	0,016603051
chr15	34600000	34850000	1	0,009746685	0,015867777
chr15	36500000	36750000	1	0,013492955	0,032698286
chr15	36550000	36800000	1	0,001244959	0,034252076
chr15	36600000	36850000	1	0,014862275	0,035395361
chr15	39150000	39400000	1	0,025825126	0,030912394
chr15	39200000	39450000	1	0,004007661	0,033072143
chr15	40000000	40250000	1	0,031640679	0,02484448
chr15	40050000	40300000	1	0,019546104	0,026755604
chr15	41350000	41600000	1	0,019302072	0,011016283
chr15	42050000	42300000	1	0,034437719	0,021489582
chr15	42100000	42350000	1	0,002246679	0,021289785
chr15	42150000	42400000	1	0,00037001	0,020704872
chr15	42200000	42450000	1	0,011351869	0,019680191
chr15	42650000	42900000	1	0,013569247	0,011443754
chr15	42700000	42950000	1	0,045246197	0,012169522
chr15	43050000	43300000	1	0,027061271	0,014671614
chr15	43100000	43350000	1	0,007503476	0,015347225
chr15	43150000	43400000	1	0,004490672	0,015847853
chr15	43200000	43450000	1	0,009582783	0,01618355
chr15	43250000	43500000	1	0,031993833	0,016759893
chr15	43450000	43700000	1	0,045060409	0,018575558
chr15	43500000	43750000	1	0,005346493	0,019305636
chr15	43550000	43800000	1	0,005896564	0,020132373
chr15	44650000	44900000	1	0,036800435	0,002902939
chr15	54150000	54400000	1	0,00883101	0,020978825
chr15	58650000	58900000	1	0,048516481	0,038601044
chr15	59250000	59500000	1	0,001728598	0,014359014
chr15	59300000	59550000	1	0,004608304	0,014359014
chr15	59350000	59600000	1	0,02252852	0,014359014
chr15	59800000	60050000	1	0,010825314	0,02128993
chr15	63150000	63400000	1	0,04300168	0,039201973
chr15	63200000	63450000	1	0,012411606	0,04260019
chr15	64850000	65100000	1	0,03739215	0,010265519

chr15	67150000	67400000	1	0,047842378	0,004787254
chr15	67700000	67950000	1	0,013526202	0,019452159
chr15	67750000	68000000	1	0,001347508	0,019452159
chr15	67800000	68050000	1	0,014473853	0,019452159
chr15	74550000	74800000	1	0,001248071	0,012832844
chr15	74600000	74850000	1	0,002187467	0,013409662
chr15	74650000	74900000	1	0,006818995	0,013439043
chr15	74700000	74950000	1	0,021904187	0,013211609
chr15	76300000	76550000	1	0,028445834	0,016629352
chr15	76350000	76600000	1	0,007616239	0,015647467
chr15	78450000	78700000	1	0,006398271	0,008402438
chr15	89450000	89700000	1	0,015469721	0,02485184
chr15	89500000	89750000	1	0,019780306	0,026582009
chr15	89950000	90200000	1	0,006478815	0,015357135
chr15	90000000	90250000	1	0,015856963	0,014531181
chr15	90300000	90550000	1	0,00660748	0,017531703
chr15	90350000	90600000	1	0,013392941	0,018361853
chr15	101300000	101550000	1	0,015457687	0,015850155
chr15	101650000	101900000	1	0,036395958	0,012215983
chr16	3300000	3550000	1	0,021205637	0,017063855
chr16	3900000	4150000	1	0,012524277	0,005940281
chr16	3950000	4200000	1	0,004008662	0,00558526
chr16	4000000	4250000	1	0,034999471	0,005428399
chr16	4350000	4600000	1	0,006473962	0,007262584
chr16	4400000	4650000	1	0,029661879	0,006691313
chr16	11450000	11700000	1	0,007050547	0,004428495
chr16	11500000	11750000	1	0,018562682	0,004513888
chr16	15800000	16050000	1	0,029460161	0,011871138
chr16	18950000	19200000	1	0,004998973	0,013243535
chr16	19000000	19250000	1	0,018274389	0,013152911
chr16	19650000	19900000	1	0,004436964	0,024891282
chr16	19700000	19950000	1	0,028641186	0,023007874
chr16	24600000	24850000	1	0,010280572	0,027062068
chr16	29800000	30050000	1	0,021640369	0,005859085
chr16	29850000	30100000	1	0,000634876	0,005506976
chr16	29900000	30150000	1	0,001068222	0,005327625
chr16	29950000	30200000	1	0,000824254	0,005296027
chr16	30000000	30250000	1	0,002299091	0,005217099
chr16	30500000	30750000	1	0,005332772	0,001901321
chr16	30550000	30800000	1	0,01297985	0,00175464
chr16	61750000	62000000	1	0,025804493	0,049547406
chr16	68550000	68800000	1	0,028569696	0,025832426
chr16	74850000	75100000	1	0,012094578	0,020583491
chr16	84300000	84550000	1	0,021862579	0,017615325
chr16	84350000	84600000	1	0,011753936	0,01884136
chr16	84700000	84950000	1	0,003422971	0,01325806

chr16	84750000	85000000	1	0,000590844	0,012934746
chr16	84800000	85050000	1	0,009097349	0,012589148
chr17	200000	450000	1	0,031061791	0,009805689
chr17	250000	500000	1	0,032253386	0,009805689
chr17	2000000	2250000	1	0,005644408	0,020552926
chr17	4150000	4400000	1	0,01526581	0,020738531
chr17	15150000	15400000	1	0,031049667	0,018296345
chr17	15200000	15450000	1	0,011033827	0,019978478
chr17	19250000	19500000	1	0,02951649	0,008451187
chr17	26900000	27150000	1	0,017232829	0,007932369
chr17	26950000	27200000	1	0,013697243	0,008435907
chr17	28600000	28850000	1	0,042469216	0,022707648
chr17	28650000	28900000	1	0,008537323	0,02407457
chr17	28700000	28950000	1	0,00072007	0,02492483
chr17	28750000	29000000	1	0,007821446	0,025333779
chr17	30300000	30550000	1	0,01508163	0,016872709
chr17	35500000	35750000	1	0,006512121	0,020973256
chr17	35550000	35800000	1	0,000170236	0,022140734
chr17	35600000	35850000	1	0,001749373	0,022613997
chr17	35650000	35900000	1	0,011205121	0,02070371
chr17	35700000	35950000	1	0,04347578	0,019733991
chr17	40600000	40850000	1	0,0279215	0,001902488
chr17	40650000	40900000	1	0,023613901	0,001809406
chr17	40800000	41050000	1	0,023913487	0,001731975
chr17	40850000	41100000	1	0,001870738	0,001790818
chr17	40900000	41150000	1	0,018778092	0,001894139
chr17	41400000	41650000	1	0,017779822	0,003254393
chr17	42350000	42600000	1	0,029674416	0,005851082
chr17	42400000	42650000	1	0,001963391	0,006174192
chr17	42450000	42700000	1	6,07E-05	0,006400884
chr17	42500000	42750000	1	0,000149314	0,006518663
chr17	42550000	42800000	1	0,000195548	0,00653604
chr17	42600000	42850000	1	3,92E-05	0,006483722
chr17	42650000	42900000	1	6,34E-05	0,006406925
chr17	42700000	42950000	1	0,000670188	0,006350902
chr17	42750000	43000000	1	0,001705815	0,006346574
chr17	42800000	43050000	1	0,001284073	0,006401926
chr17	42850000	43100000	1	0,000251834	0,00650189
chr17	42900000	43150000	1	5,76E-05	0,006612574
chr17	42950000	43200000	1	0,001391108	0,006388395
chr17	43000000	43250000	1	0,016305841	0,006109264
chr17	44750000	45000000	1	0,018919582	0,011590426
chr17	44950000	45200000	1	0,014223465	0,010694219
chr17	45200000	45450000	1	0,029136242	0,011286533
chr17	45650000	45900000	1	0,008792104	0,005720043
chr17	45700000	45950000	1	0,019072421	0,005758097

chr17	45750000	46000000	1	0,00637089	0,005591836
chr17	45800000	46050000	1	0,015570898	0,005213293
chr17	61750000	62000000	1	0,02969748	0,009459983
chr17	62300000	62550000	1	0,027073928	0,026025051
chr17	65300000	65550000	1	0,028254101	0,015932784
chr17	73100000	73350000	1	0,019462801	0,003428864
chr17	73150000	73400000	1	0,000672095	0,003700255
chr17	73200000	73450000	1	0,006012764	0,003797445
chr17	73250000	73500000	1	0,022743248	0,003791917
chr17	73300000	73550000	1	0,048564843	0,003731025
chr17	73500000	73750000	1	0,038179353	0,002916533
chr17	73550000	73800000	1	0,005294242	0,002977444
chr17	73600000	73850000	1	0,005098546	0,0031391
chr17	73900000	74150000	1	0,048625719	0,0037813
chr18	9250000	9500000	1	0,009029338	0,014307286
chr18	12650000	12900000	1	0,013423842	0,028851992
chr18	12700000	12950000	1	0,036926129	0,026651569
chr18	18950000	19200000	1	0,009177692	0,014909696
chr18	19000000	19250000	1	0,001562152	0,014459067
chr18	19050000	19300000	1	0,002435415	0,014384583
chr18	19100000	19350000	1	0,001445447	0,013828231
chr18	19150000	19400000	1	0,034951351	0,012833785
chr18	20000000	20250000	1	0,030628322	0,028730664
chr18	20050000	20300000	1	0,001135243	0,030615122
chr18	20100000	20350000	1	0,004619438	0,031761868
chr18	43400000	43650000	1	0,035086186	0,014022047
chr18	55350000	55600000	1	0,042536806	0,028640988
chr18	68850000	69100000	1	0,020943944	0,012416873
chr18	68900000	69150000	1	5,78E-05	0,012427721
chr18	68950000	69200000	1	5,62E-07	0,012456849
chr18	69000000	69250000	1	7,28E-06	0,012300147
chr18	69050000	69300000	1	0,002373003	0,011749473
chr18	71300000	71550000	1	0,006302851	0,014541674
chr18	71350000	71600000	1	0,019992439	0,014541674
chr20	3250000	3500000	1	0,016445351	0,016180862
chr20	3300000	3550000	1	0,010219509	0,016180862
chr20	13850000	14100000	1	0,005067274	0,009574489
chr20	13900000	14150000	1	9,75E-05	0,009622595
chr20	13950000	14200000	1	4,58E-06	0,009575511
chr20	14000000	14250000	1	0,000248119	0,009292142
chr20	14050000	14300000	1	0,025092824	0,008649286
chr20	22600000	22850000	1	0,005929833	0,034516365
chr20	22650000	22900000	1	0,0259464	0,036473975
chr20	32800000	33050000	1	0,003068942	0,009784029
chr20	32850000	33100000	1	0,002515156	0,009384607
chr20	33800000	34050000	1	0,027708427	0,005190609

chr20	33850000	34100000	1	0,003910086	0,005541989
chr20	34150000	34400000	1	0,02853912	0,004727271
chr20	34850000	35100000	1	0,021932768	0,027786527
chr20	35350000	35600000	1	0,0142734	0,011806019
chr20	35600000	35850000	1	0,035002504	0,013734726
chr20	36750000	37000000	1	0,021839739	0,027931846
chr20	48650000	48900000	1	0,016708217	0,004276018
chr20	49100000	49350000	1	0,010846745	0,005771975
chr19	6250000	6500000	1	0,005490979	0,002998862
chr19	6300000	6550000	1	0,029829667	0,002843228
chr19	6400000	6650000	1	0,026113141	0,002897561
chr19	8200000	8450000	1	0,01251697	0,008084256
chr19	8250000	8500000	1	0,000185984	0,00870058
chr19	8300000	8550000	1	0,000175949	0,008759202
chr19	8350000	8600000	1	0,000511152	0,008985964
chr19	8400000	8650000	1	0,002181696	0,009097348
chr19	8450000	8700000	1	0,00137178	0,00887207
chr19	8500000	8750000	1	0,017782768	0,008192305
chr19	9950000	10200000	1	0,020567115	0,014769872
chr19	10500000	10750000	1	0,007571174	0,003723356
chr19	10550000	10800000	1	0,005681915	0,003697129
chr19	10600000	10850000	1	0,003935823	0,003673366
chr19	10650000	10900000	1	0,000362593	0,003613155
chr19	10700000	10950000	1	0,000414157	0,003608449
chr19	10750000	11000000	1	0,001426983	0,003608449
chr19	10800000	11050000	1	0,001032111	0,003608449
chr19	10850000	11100000	1	0,000157584	0,003608449
chr19	10900000	11150000	1	1,66E-05	0,003563254
chr19	10950000	11200000	1	0,000975313	0,003672828
chr19	11000000	11250000	1	0,033748637	0,003920699
chr19	18050000	18300000	1	0,041212935	0,007378854
chr19	39550000	39800000	1	0,04353378	0,010652959
chr19	45800000	46050000	1	0,014045207	0,005976711
chr19	45850000	46100000	1	0,004271789	0,006196535
chr19	45900000	46150000	1	0,001969751	0,006361403
chr19	45950000	46200000	1	0,003636849	0,006954578
chr19	46000000	46250000	1	0,026928244	0,007341722
chr19	46500000	46750000	1	0,048327504	0,01582329
chr19	46550000	46800000	1	0,002598287	0,01582329
chr19	46600000	46850000	1	0,018102895	0,01582329
chr19	47550000	47800000	1	0,042965492	0,004202697
chr19	47600000	47850000	1	0,023556346	0,004333586
chr19	47650000	47900000	1	0,026299425	0,004467784
chr19	47700000	47950000	1	0,025687641	0,004564097
chr19	47750000	48000000	1	0,011673008	0,004578424
chr19	47800000	48050000	1	0,001582537	0,004467603

chr19	47850000	48100000		1	0,011244298	0,00418277
chr19	49550000	49800000		1	0,028957718	0,015981112
chr19	54300000	54550000		1	0,045254873	0,015076774
chr22	30300000	30550000		1	0,034290539	0,027435684
chr22	36350000	36600000		1	0,033226618	0,018306533
chr22	39300000	39550000		1	0,036229898	0,01214895
chr22	39350000	39600000		1	0,044989045	0,013090695
chr22	41450000	41700000		1	0,007920173	0,008017088
chr22	44850000	45100000		1	0,018485084	0,008708927
chr22	44900000	45150000		1	0,000227689	0,008947258
chr22	44950000	45200000		1	0,028733413	0,009761104
chr22	45850000	46100000		1	0,045345934	0,005326161
chr22	46000000	46250000		1	0,010693866	0,005571038
chr22	46050000	46300000		1	0,002553924	0,005860229
chr22	46100000	46350000		1	0,022285492	0,006054473
chr22	46150000	46400000		1	0,046216277	0,005407212
chr22	46200000	46450000		1	0,015292344	0,005607909
chr22	46750000	47000000		1	0,022518009	0,014991937
chr21	34600000	34850000		1	0,02694921	0,017370956

Supplementary Table S3
List and Coordinates of PREP1 down-regulation induced early-to-late (EtoL) shifted genomic windows.

Chr	Start	End	Adj_Pvalue_LtoE	Adj_Pvalue_EtoL	Diff_S50
chr1	8100000	8350000	1	0,010046729	0,016668876
chr1	9450000	9700000	1	0,010668936	0,014763006
chr1	9800000	10050000	1	0,044046198	0,011653145
chr1	9850000	10100000	1	0,003207613	0,010992879
chr1	9900000	10150000	1	0,000108585	0,010579138
chr1	9950000	10200000	1	0,000327069	0,010014094
chr1	10000000	10250000	1	0,009027634	0,009543768
chr1	10850000	11100000	1	0,028269587	0,01293364
chr1	10900000	11150000	1	0,000985331	0,014332189
chr1	10950000	11200000	1	0,000138512	0,014018114
chr1	11000000	11250000	1	0,008116364	0,013326893
chr1	12100000	12350000	1	0,01686947	0,013277876
chr1	12150000	12400000	1	0,005639785	0,013058139
chr1	16100000	16350000	1	0,011598529	0,002972254
chr1	16150000	16400000	1	0,008526088	0,002807093
chr1	16200000	16450000	1	0,042771219	0,00271946
chr1	16250000	16500000	1	0,035218588	0,002903549
chr1	16300000	16550000	1	0,008779055	0,00287663
chr1	19450000	19700000	1	0,047325326	0,031490029
chr1	19500000	19750000	1	0,038638319	0,034497433
chr1	21000000	21250000	1	0,03729389	0,008389796
chr1	22050000	22300000	1	0,013236967	0,006117388
chr1	22100000	22350000	1	0,013654811	0,006123313
chr1	22150000	22400000	1	0,012812132	0,005717688
chr1	24600000	24850000	1	0,017094641	0,023845095
chr1	27250000	27500000	1	0,043594151	0,003931502
chr1	27300000	27550000	1	0,001173095	0,003931502
chr1	27350000	27600000	1	1,19E-05	0,003931502
chr1	27400000	27650000	1	0,002026305	0,003931502
chr1	27950000	28200000	1	0,019388358	0,013079842
chr1	28600000	28850000	1	0,014350039	0,003930439
chr1	32050000	32300000	1	0,021204414	0,003341968
chr1	32500000	32750000	1	0,007796403	0,005475981
chr1	32550000	32800000	1	0,000549082	0,005333545
chr1	32600000	32850000	1	0,008439846	0,005068764
chr1	35800000	36050000	1	0,002039133	0,004949127
chr1	35850000	36100000	1	0,014016497	0,005051808
chr1	36150000	36400000	1	0,036942673	0,006274001
chr1	38100000	38350000	1	0,020578054	0,011191839
chr1	40450000	40700000	1	0,019327041	0,027695418
chr1	40500000	40750000	1	0,012510837	0,02596203

chr1	41600000	41850000	1	0,003609384	0,014436388
chr1	41650000	41900000	1	0,005978473	0,013850561
chr1	43300000	43550000	1	0,019722602	0,014216151
chr1	44850000	45100000	1	0,00645372	0,008426756
chr1	44900000	45150000	1	0,001329429	0,008426756
chr1	44950000	45200000	1	0,002162509	0,008585262
chr1	45000000	45250000	1	0,001150426	0,008491055
chr1	45050000	45300000	1	0,037091098	0,008290107
chr1	45450000	45700000	1	0,0232324	0,01447936
chr1	51850000	52100000	1	0,012064577	0,033538217
chr1	51900000	52150000	1	0,001373793	0,034439602
chr1	51950000	52200000	1	6,87E-05	0,033973101
chr1	52000000	52250000	1	0,000510163	0,03316103
chr1	52050000	52300000	1	0,006594534	0,032021666
chr1	52100000	52350000	1	0,034779841	0,030574133
chr1	53550000	53800000	1	0,013617665	0,017837138
chr1	54000000	54250000	1	0,003972537	0,010329685
chr1	54050000	54300000	1	0,007215042	0,009892807
chr1	54100000	54350000	1	0,004020424	0,009937556
chr1	85650000	85900000	1	0,014795275	0,029965653
chr1	85700000	85950000	1	0,001106864	0,031457784
chr1	85750000	86000000	1	0,015558049	0,029999992
chr1	86500000	86750000	1	0,041276032	0,008686897
chr1	93500000	93750000	1	0,006398158	0,022477045
chr1	94550000	94800000	1	0,006164718	0,02031218
chr1	94900000	95150000	1	0,02250307	0,015320513
chr1	94950000	95200000	1	0,02182381	0,014293648
chr1	95150000	95400000	1	0,02502953	0,014848098
chr1	109300000	109550000	1	0,022807918	0,014994247
chr1	109750000	110000000	1	0,014633055	0,008739748
chr1	110300000	110550000	1	0,012332907	0,020869165
chr1	110350000	110600000	1	0,000381656	0,020451588
chr1	110400000	110650000	1	0,00212121	0,019850783
chr1	110450000	110700000	1	0,0172101	0,019074349
chr1	112000000	112250000	1	0,048002292	0,006600589
chr1	112500000	112750000	1	0,031771288	0,01003458
chr1	113000000	113250000	1	0,009575249	0,016273822
chr1	113050000	113300000	1	0,010978556	0,01738337
chr1	150150000	150400000	1	0,007009883	0,005768491
chr1	150200000	150450000	1	0,001282986	0,006066839
chr1	150250000	150500000	1	0,020190797	0,006176434
chr1	150650000	150900000	1	0,03942072	0,011835074
chr1	151450000	151700000	1	0,028555344	0,010087084
chr1	155750000	156000000	1	0,011515191	0,008476208
chr1	156300000	156550000	1	0,006290565	0,003534638
chr1	156350000	156600000	1	0,02278509	0,003737641

chr1	156650000	156900000	1	0,047531701	0,004628268
chr1	173700000	173950000	1	0,008734386	0,016512114
chr1	180450000	180700000	1	0,005084096	0,049509656
chr1	183200000	183450000	1	0,029617779	0,032782747
chr1	183250000	183500000	1	0,010024407	0,03069516
chr1	186450000	186700000	1	0,010734006	0,050866626
chr1	199050000	199300000	1	0,015027043	0,021524545
chr1	200400000	200650000	1	0,00755811	0,023021318
chr1	200450000	200700000	1	6,30E-05	0,024442505
chr1	200500000	200750000	1	4,81E-05	0,024954212
chr1	200550000	200800000	1	2,31E-05	0,024887841
chr1	200600000	200850000	1	0,000133326	0,025173658
chr1	200650000	200900000	1	0,000572809	0,025428512
chr1	200700000	200950000	1	0,000421766	0,025392706
chr1	200750000	201000000	1	9,70E-05	0,024947495
chr1	200800000	201050000	1	0,001998451	0,024947495
chr1	200850000	201100000	1	0,03841752	0,024947495
chr1	203900000	204150000	1	0,006135594	0,017343704
chr1	203950000	204200000	1	0,004954766	0,017343704
chr1	205500000	205750000	1	0,020483868	0,012189097
chr1	205550000	205800000	1	0,002690244	0,012074315
chr1	205600000	205850000	1	0,000916664	0,011634011
chr1	205650000	205900000	1	0,034279101	0,010838629
chr1	206600000	206850000	1	0,011920485	0,018520333
chr1	206650000	206900000	1	0,000545212	0,019460691
chr1	206700000	206950000	1	0,006696801	0,020018611
chr1	207350000	207600000	1	0,012187342	0,003976231
chr1	207400000	207650000	1	0,007211957	0,003669425
chr1	210300000	210550000	1	0,008106964	0,02056218
chr1	212100000	212350000	1	0,048620285	0,02194937
chr1	212150000	212400000	1	0,007648599	0,02194937
chr1	212200000	212450000	1	0,00059465	0,02194937
chr1	212250000	212500000	1	0,005771749	0,02194937
chr1	218500000	218750000	1	0,030571964	0,038353155
chr1	218550000	218800000	1	0,009716982	0,039515196
chr1	224050000	224300000	1	0,02332427	0,014268278
chr1	232250000	232500000	1	0,014566876	0,017875492
chr1	232300000	232550000	1	0,043408845	0,016690643
chr2	22200000	22450000	1	0,04632509	0,019568283
chr2	24300000	24550000	1	0,013820723	0,020205723
chr2	24350000	24600000	1	0,038572663	0,018703602
chr2	25150000	25400000	1	0,01425154	0,004503287
chr2	25200000	25450000	1	0,016315837	0,004160461
chr2	38350000	38600000	1	0,016839366	0,016809844
chr2	38400000	38650000	1	0,001938391	0,016809844
chr2	38450000	38700000	1	0,001204228	0,016809844

chr2	38500000	38750000		1	0,035388189	0,017168229
chr2	38900000	39150000		1	0,028061972	0,010511045
chr2	44050000	44300000		1	0,012316071	0,025332
chr2	44100000	44350000		1	0,006372342	0,025848919
chr2	46850000	47100000		1	0,010642677	0,025791751
chr2	46900000	47150000		1	0,005692638	0,027174997
chr2	55250000	55500000		1	0,005240428	0,017260772
chr2	55300000	55550000		1	0,008496528	0,018344791
chr2	55350000	55600000		1	0,022653068	0,018514851
chr2	55400000	55650000		1	0,022269001	0,018135309
chr2	55450000	55700000		1	0,02570563	0,017571197
chr2	58150000	58400000		1	0,002380336	0,005724021
chr2	58200000	58450000		1	0,002277445	0,005994119
chr2	64900000	65150000		1	0,010804246	0,021036123
chr2	64950000	65200000		1	0,00032366	0,022959246
chr2	65000000	65250000		1	6,03E-06	0,023016675
chr2	65050000	65300000		1	1,04E-06	0,023093627
chr2	65100000	65350000		1	1,14E-06	0,023123365
chr2	65150000	65400000		1	5,02E-07	0,023005684
chr2	65200000	65450000		1	3,70E-05	0,022635288
chr2	65250000	65500000		1	0,001593485	0,021937756
chr2	65300000	65550000		1	0,019934732	0,020890884
chr2	66300000	66550000		1	0,013944795	0,026705288
chr2	66350000	66600000		1	0,000253487	0,02633967
chr2	66400000	66650000		1	0,000159212	0,026320005
chr2	66450000	66700000		1	0,000122249	0,026320005
chr2	66500000	66750000		1	0,001576484	0,026320005
chr2	67000000	67250000		1	0,022946675	0,048777813
chr2	67050000	67300000		1	0,004100824	0,051851346
chr2	67600000	67850000		1	0,029277006	0,017643978
chr2	67650000	67900000		1	0,024744372	0,018402147
chr2	67750000	68000000		1	0,026600519	0,018634665
chr2	67800000	68050000		1	0,005092501	0,017712456
chr2	69850000	70100000		1	0,021062537	0,006702854
chr2	77400000	77650000		1	0,00097352	0,052854717
chr2	77450000	77700000		1	0,003223115	0,051853938
chr2	77500000	77750000		1	0,028501759	0,049292556
chr2	88900000	89150000		1	0,042378284	0,026479446
chr2	89350000	89600000		1	0,034576902	0,01269114
chr2	89400000	89650000		1	0,000987477	0,012980688
chr2	89450000	89700000		1	0,002720773	0,013161413
chr2	89500000	89750000		1	0,002080504	0,013103683
chr2	89550000	89800000		1	0,000525085	0,012727442
chr2	89600000	89850000		1	0,015950998	0,012727442
chr2	112750000	113000000		1	0,014884543	0,019904906
chr2	112800000	113050000		1	0,003417533	0,021224629

chr2	112850000	113100000	1	0,043542257	0,021882158
chr2	119350000	119600000	1	0,033410151	0,033384926
chr2	144200000	144450000	1	0,019434114	0,04040141
chr2	159350000	159600000	1	0,038564493	0,004381708
chr2	160600000	160850000	1	0,006313183	0,01455895
chr2	171900000	172150000	1	0,026927612	0,019428615
chr2	184950000	185200000	1	0,032154593	0,036024884
chr2	185000000	185250000	1	0,002979515	0,037822631
chr2	185050000	185300000	1	0,001888487	0,038093833
chr2	190700000	190950000	1	0,011702308	0,030385097
chr2	190750000	191000000	1	0,001892556	0,030385097
chr2	190800000	191050000	1	0,02387135	0,030385097
chr2	203050000	203300000	1	0,017292728	0,002958333
chr2	203250000	203500000	1	0,008358019	0,002749714
chr2	203300000	203550000	1	0,033634872	0,003002754
chr2	208400000	208650000	1	0,049932498	0,024344944
chr2	219700000	219950000	1	0,027338251	0,014233536
chr2	228350000	228600000	1	0,035567921	0,008984106
chr2	232550000	232800000	1	0,027982301	0,00340604
chr2	233750000	234000000	1	0,011511037	0,020997577
chr2	233800000	234050000	1	0,035190481	0,020997577
chr2	238600000	238850000	1	0,012283861	0,013010223
chr2	238650000	238900000	1	0,038103558	0,013901107
chr3	9800000	10050000	1	0,007354821	0,006189297
chr3	9850000	10100000	1	0,002873306	0,006552971
chr3	12300000	12550000	1	0,002536246	0,00931304
chr3	12350000	12600000	1	0,006673095	0,009790274
chr3	12400000	12650000	1	0,043240047	0,009886323
chr3	15300000	15550000	1	0,019647388	0,01609824
chr3	15350000	15600000	1	0,009246943	0,017079347
chr3	24900000	25150000	1	0,043224714	0,037791702
chr3	24950000	25200000	1	0,011938776	0,035631153
chr3	32950000	33200000	1	0,021631904	0,024135259
chr3	33000000	33250000	1	0,004387013	0,025074365
chr3	33050000	33300000	1	0,000312518	0,025868972
chr3	33100000	33350000	1	0,000891029	0,026471059
chr3	33150000	33400000	1	0,017061148	0,024380543
chr3	47750000	48000000	1	0,004737125	0,00342746
chr3	47800000	48050000	1	0,003062225	0,003632181
chr3	47850000	48100000	1	0,01776204	0,003691017
chr3	49800000	50050000	1	0,013322416	0,005591706
chr3	49850000	50100000	1	0,000376515	0,005889758
chr3	49900000	50150000	1	0,000191207	0,006056992
chr3	49950000	50200000	1	0,002184341	0,006121289
chr3	50000000	50250000	1	0,008592077	0,005619301
chr3	50050000	50300000	1	0,019559493	0,00544365

chr3	50100000	50350000		1	0,032065818	0,005268551
chr3	50150000	50400000		1	0,037028098	0,005125615
chr3	50200000	50450000		1	0,023033285	0,005069806
chr3	50250000	50500000		1	0,004353092	0,005167346
chr3	50300000	50550000		1	0,005288574	0,005462727
chr3	52650000	52900000		1	0,026561116	0,010038659
chr3	60350000	60600000		1	0,043032437	0,067942317
chr3	60400000	60650000		1	0,005763396	0,064510312
chr3	60750000	61000000		1	0,005635656	0,050289913
chr3	60800000	61050000		1	0,000324292	0,047516403
chr3	60850000	61100000		1	0,00069941	0,046581366
chr3	98550000	98800000		1	0,02926135	0,035575481
chr3	101600000	101850000		1	0,030582679	0,039159536
chr3	101650000	101900000		1	0,046112372	0,036021219
chr3	104650000	104900000		1	0,025256256	0,029021199
chr3	104700000	104950000		1	0,016533994	0,03103735
chr3	105450000	105700000		1	0,024622503	0,017944386
chr3	107800000	108050000		1	0,009732715	0,026654398
chr3	124800000	125050000		1	0,025006435	0,019249426
chr3	124850000	125100000		1	0,004623508	0,018323567
chr3	127500000	127750000		1	0,005194883	0,022426812
chr3	127550000	127800000		1	0,008742791	0,021148625
chr3	132000000	132250000		1	0,024862524	0,008744746
chr3	133500000	133750000		1	0,044308419	0,015353588
chr3	133650000	133900000		1	0,034336538	0,015054199
chr3	133700000	133950000		1	0,033360647	0,016081185
chr3	141200000	141450000		1	0,027986749	0,015369008
chr3	177750000	178000000		1	0,042985646	0,027142324
chr3	177800000	178050000		1	0,004965866	0,025971386
chr3	183500000	183750000		1	0,027354221	0,017825858
chr3	183550000	183800000		1	0,007900028	0,019064946
chr3	185050000	185300000		1	0,018284135	0,020626766
chr3	191000000	191250000		1	0,012457634	0,007931681
chr3	191050000	191300000		1	0,004804838	0,008323104
chr3	191750000	192000000		1	0,047989233	0,018889375
chr3	191800000	192050000		1	0,046318406	0,020351676
chr3	196350000	196600000		1	0,048535644	0,007404744
chr3	197000000	197250000		1	0,034900524	0,009882963
chr3	197550000	197800000		1	0,009641683	0,011107671
chr4	2900000	3150000		1	0,017085244	0,016553687
chr4	2950000	3200000		1	0,028152346	0,015523788
chr4	66600000	66850000		1	0,00446977	0,017911114
chr4	66650000	66900000		1	0,022967006	0,017911114
chr4	76850000	77100000		1	0,00639846	0,017788506
chr4	83600000	83850000		1	0,011001834	0,013497043
chr4	83650000	83900000		1	0,047793772	0,014362797

chr4	83950000	84200000	1	0,029519551	0,010579489
chr4	84250000	84500000	1	0,02195496	0,012096866
chr4	100550000	100800000	1	0,015928095	0,042606377
chr4	100600000	100850000	1	0,002256126	0,045555421
chr4	100650000	100900000	1	0,000222458	0,045723755
chr4	100700000	100950000	1	0,005893603	0,043546915
chr4	103600000	103850000	1	0,037718176	0,010025465
chr4	114600000	114850000	1	0,005183298	0,013847468
chr4	114650000	114900000	1	0,008880913	0,013699762
chr4	120600000	120850000	1	0,003194525	0,061330957
chr4	120650000	120900000	1	0,010455522	0,058102953
chr4	124950000	125200000	1	0,025906136	0,038871706
chr4	129050000	129300000	1	0,017634822	0,01427954
chr4	140100000	140350000	1	0,048159514	0,015141975
chr4	152050000	152300000	1	0,044572731	0,025571137
chr4	152900000	153150000	1	0,015530539	0,035166331
chr4	174100000	174350000	1	0,047784266	0,019141228
chr4	175150000	175400000	1	0,025884561	0,016574246
chr4	175200000	175450000	1	0,027766359	0,015253339
chr4	177550000	177800000	1	0,013862367	0,040379704
chr4	177600000	177850000	1	0,040621019	0,03671328
chr5	32550000	32800000	1	0,011542962	0,006711666
chr5	55900000	56150000	1	0,003624477	0,018121255
chr5	55950000	56200000	1	0,00029796	0,018900754
chr5	56000000	56250000	1	0,001636406	0,019222874
chr5	56050000	56300000	1	0,01180603	0,019286542
chr5	56100000	56350000	1	0,027796533	0,019365091
chr5	56150000	56400000	1	0,017075751	0,019916485
chr5	56200000	56450000	1	0,003997737	0,020562525
chr5	62750000	63000000	1	0,024062943	0,004402375
chr5	62800000	63050000	1	0,01908748	0,004542623
chr5	62850000	63100000	1	0,022292503	0,004542623
chr5	62900000	63150000	1	0,0130858	0,004542623
chr5	62950000	63200000	1	0,002406908	0,004542623
chr5	63000000	63250000	1	9,88E-05	0,004542623
chr5	63050000	63300000	1	1,49E-05	0,004646952
chr5	63100000	63350000	1	0,004202103	0,004674922
chr5	91700000	91950000	1	0,005229298	0,004777537
chr5	95350000	95600000	1	0,007533959	0,0247301
chr5	102250000	102500000	1	0,000870519	0,016292916
chr5	102300000	102550000	1	0,000801879	0,015930763
chr5	102350000	102600000	1	0,012461281	0,015211577
chr5	111200000	111450000	1	0,007050306	0,022052262
chr5	111250000	111500000	1	0,007259464	0,022210159
chr5	123600000	123850000	1	0,011527882	0,022582672
chr5	123650000	123900000	1	0,000686554	0,023552003

chr5	123700000	123950000		1	0,000542702	0,024272961
chr5	123750000	124000000		1	0,002673786	0,024631464
chr5	123800000	124050000		1	0,002425254	0,024562684
chr5	123850000	124100000		1	0,00041241	0,024147205
chr5	123900000	124150000		1	0,000113963	0,023874769
chr5	123950000	124200000		1	0,000123581	0,023662259
chr5	124000000	124250000		1	0,003031912	0,022341131
chr5	126900000	127150000		1	0,007583647	0,032507286
chr5	127400000	127650000		1	0,009846723	0,022087323
chr5	131900000	132150000		1	0,028539469	0,002617466
chr5	132150000	132400000		1	0,022927821	0,002168914
chr5	137500000	137750000		1	0,008178948	0,012428853
chr5	137550000	137800000		1	0,012609889	0,013168212
chr5	139600000	139850000		1	0,041563649	0,015841578
chr5	139650000	139900000		1	0,003852825	0,015160127
chr5	142550000	142800000		1	0,031556207	0,017265148
chr5	142600000	142850000		1	0,00772041	0,018658456
chr5	179600000	179850000		1	0,025627686	0,015058811
chr6	9200000	9450000		1	0,025851447	0,023504654
chr6	9250000	9500000		1	0,03531835	0,02524565
chr6	11200000	11450000		1	0,03944196	0,03484604
chr6	11250000	11500000		1	0,015501363	0,035168492
chr6	11300000	11550000		1	0,002542148	0,034916939
chr6	11350000	11600000		1	0,000337672	0,034038276
chr6	11400000	11650000		1	0,006245706	0,032572033
chr6	17800000	18050000		1	0,043100359	0,008459229
chr6	22100000	22350000		1	0,041881216	0,027962852
chr6	31050000	31300000		1	0,016914059	0,01955715
chr6	31100000	31350000		1	0,031570637	0,01955715
chr6	32900000	33150000		1	0,036834772	0,016419352
chr6	34950000	35200000		1	0,024105835	0,014329849
chr6	35700000	35950000		1	0,048304828	0,016723802
chr6	35750000	36000000		1	0,004393812	0,015970518
chr6	41850000	42100000		1	0,049422124	0,006693237
chr6	42400000	42650000		1	0,010467807	0,01795877
chr6	47000000	47250000		1	0,031048159	0,030317082
chr6	112200000	112450000		1	0,011566692	0,029661635
chr6	113150000	113400000		1	0,032896792	0,022867023
chr6	113200000	113450000		1	0,005693644	0,024491852
chr6	140650000	140900000		1	0,017107422	0,050294063
chr6	140700000	140950000		1	0,004643172	0,04782735
chr6	156000000	156250000		1	0,040950285	0,019634993
chr6	158550000	158800000		1	0,019030645	0,008612261
chr6	158700000	158950000		1	0,02320664	0,008250048
chr6	158750000	159000000		1	0,009510118	0,008250048
chr6	158900000	159150000		1	0,027789418	0,008462155

chr6	158950000	159200000	1	0,014271967	0,008913416
chr7	5750000	6000000	1	0,038117116	0,014851957
chr7	6200000	6450000	1	0,014942865	0,00597675
chr7	15000000	15250000	1	0,046768716	0,074064675
chr7	18600000	18850000	1	0,033764185	0,009065621
chr7	22750000	23000000	1	0,04567921	0,013008363
chr7	22800000	23050000	1	0,004939127	0,013819133
chr7	22850000	23100000	1	0,00036243	0,014298753
chr7	22900000	23150000	1	0,009750237	0,014607499
chr7	40500000	40750000	1	0,035169433	0,029192082
chr7	40550000	40800000	1	0,000764604	0,031307021
chr7	40600000	40850000	1	0,000103202	0,032462713
chr7	40650000	40900000	1	0,000339551	0,032810795
chr7	40700000	40950000	1	0,0002443	0,032632798
chr7	40750000	41000000	1	7,07E-05	0,032242601
chr7	40800000	41050000	1	3,46E-05	0,031888782
chr7	40850000	41100000	1	7,43E-05	0,031675616
chr7	40900000	41150000	1	0,000116317	0,030722371
chr7	40950000	41200000	1	4,10E-05	0,031023226
chr7	41000000	41250000	1	0,000550838	0,032058672
chr7	41050000	41300000	1	0,022090485	0,03394207
chr7	41400000	41650000	1	0,007762395	0,045030667
chr7	41450000	41700000	1	0,002413547	0,043232658
chr7	41500000	41750000	1	0,041417526	0,040714066
chr7	43750000	44000000	1	0,001824825	0,001932069
chr7	65550000	65800000	1	0,009869806	0,025321315
chr7	65600000	65850000	1	0,009822749	0,025321315
chr7	66150000	66400000	1	0,016856401	0,01044852
chr7	66200000	66450000	1	0,000424229	0,010138353
chr7	73200000	73450000	1	0,038944996	0,01375373
chr7	75400000	75650000	1	0,013881488	0,020157095
chr7	78950000	79200000	1	0,033709689	0,058256663
chr7	79000000	79250000	1	0,001545698	0,0628695
chr7	79050000	79300000	1	0,014061482	0,059887585
chr7	79600000	79850000	1	0,004728975	0,036657896
chr7	79650000	79900000	1	0,000160186	0,034992371
chr7	79700000	79950000	1	0,007235331	0,034309579
chr7	83150000	83400000	1	0,004322602	0,03065187
chr7	83200000	83450000	1	3,63E-05	0,032170287
chr7	83250000	83500000	1	2,76E-06	0,032711223
chr7	83300000	83550000	1	5,31E-06	0,032767671
chr7	83350000	83600000	1	6,57E-06	0,032754503
chr7	83400000	83650000	1	0,00083085	0,032873694
chr7	83450000	83700000	1	0,026967978	0,033076156
chr7	99600000	99850000	1	0,025891661	0,003308586
chr7	99650000	99900000	1	0,002498862	0,003663025

chr7	99700000	99950000		1	0,001935696	0,003703217
chr7	99750000	100000000		1	0,025041713	0,003606842
chr7	100450000	100700000		1	0,016025586	0,019086364
chr7	100500000	100750000		1	0,002803054	0,019086364
chr7	100550000	100800000		1	0,000266011	0,019086364
chr7	100600000	100850000		1	0,000196616	0,019437903
chr7	100650000	100900000		1	0,007159239	0,019681955
chr7	100950000	101200000		1	0,035320168	0,016049093
chr7	101000000	101250000		1	0,001087875	0,015091044
chr7	101050000	101300000		1	1,78E-05	0,014505953
chr7	101100000	101350000		1	0,007031352	0,014008978
chr7	114750000	115000000		1	0,048190441	0,015350247
chr7	129000000	129250000		1	0,02244833	0,020575552
chr7	130200000	130450000		1	0,017556945	0,007030965
chr7	130250000	130500000		1	0,000507382	0,007030965
chr7	130300000	130550000		1	0,000338957	0,007248195
chr7	130350000	130600000		1	0,018873966	0,007308481
chr7	130950000	131200000		1	0,021210285	0,016749549
chr7	131000000	131250000		1	0,001195585	0,016463124
chr7	131050000	131300000		1	0,000848861	0,01601185
chr7	131100000	131350000		1	0,009421244	0,015463467
chr7	131150000	131400000		1	0,03937157	0,014808412
chr7	143350000	143600000		1	0,002770262	0,040356908
chr7	143400000	143650000		1	0,000140129	0,040356908
chr7	143450000	143700000		1	0,000177488	0,040356908
chr7	143500000	143750000		1	0,004568069	0,039936826
chr7	143750000	144000000		1	0,015016908	0,044655051
chr7	143800000	144050000		1	0,027769456	0,04722668
chrX	46200000	46450000		1	0,003531026	0,016830775
chrX	46250000	46500000		1	0,001756934	0,017806451
chrX	46300000	46550000		1	0,003112658	0,018012995
chrX	46350000	46600000		1	0,000715227	0,017750859
chrX	46400000	46650000		1	0,000556368	0,017340319
chrX	46450000	46700000		1	0,009155604	0,017032954
chrX	46500000	46750000		1	0,044578704	0,016974303
chrX	46700000	46950000		1	0,019184095	0,021152406
chrX	46750000	47000000		1	0,001807378	0,020654108
chrX	46800000	47050000		1	0,006439858	0,019568295
chrX	53050000	53300000		1	0,023729633	0,013813385
chrX	53100000	53350000		1	0,006059833	0,01483963
chrX	53150000	53400000		1	0,04629598	0,0153868
chrX	53300000	53550000		1	0,01828053	0,012980627
chrX	53350000	53600000		1	0,001711685	0,012939412
chrX	53400000	53650000		1	0,000294919	0,012699972
chrX	53450000	53700000		1	0,006118161	0,012080855
chrX	104100000	104350000		1	0,043291222	0,0110765

chrX	104650000	104900000	1	0,00592834	0,018531723
chrX	139950000	140200000	1	0,019362127	0,017865884
chrX	140150000	140400000	1	0,028019598	0,018416626
chrX	140600000	140850000	1	0,020765558	0,031546058
chrX	140650000	140900000	1	0,000831125	0,033309707
chrX	140700000	140950000	1	0,001385676	0,034371605
chr8	7750000	8000000	1	0,019901642	0,029826864
chr8	7800000	8050000	1	0,018255125	0,031900465
chr8	30000000	30250000	1	0,027168089	0,043101751
chr8	42300000	42550000	1	0,02763792	0,033107233
chr8	42350000	42600000	1	0,019682122	0,030748782
chr8	77300000	77550000	1	0,013510013	0,059091524
chr8	87050000	87300000	1	0,032776423	0,041429891
chr8	94600000	94850000	1	0,020508501	0,019813327
chr8	94650000	94900000	1	0,000397645	0,018213785
chr8	94700000	94950000	1	0,016903554	0,017818435
chr8	101550000	101800000	1	0,014436474	0,026418826
chr8	101600000	101850000	1	0,000966867	0,027700813
chr8	101650000	101900000	1	0,005877545	0,028623975
chr8	107950000	108200000	1	0,011272497	0,03224485
chr8	108000000	108250000	1	0,000280464	0,031652763
chr8	108050000	108300000	1	0,001754729	0,030923383
chr8	108100000	108350000	1	0,010026151	0,029888026
chr8	118800000	119050000	1	0,038560132	0,012923163
chr8	119050000	119300000	1	0,048709994	0,015111152
chr8	119100000	119350000	1	0,013721398	0,015331565
chr8	119150000	119400000	1	0,003913113	0,015490058
chr8	119200000	119450000	1	0,00109722	0,015584853
chr8	119250000	119500000	1	0,000272345	0,015653938
chr8	119300000	119550000	1	0,00011145	0,015653938
chr8	119350000	119600000	1	0,000561343	0,015653938
chr8	119400000	119650000	1	0,009347924	0,015653938
chr8	119850000	120100000	1	0,033151717	0,028928742
chr8	119900000	120150000	1	0,003116374	0,027704113
chr8	141950000	142200000	1	0,010632042	0,01276773
chr8	142000000	142250000	1	0,003307197	0,012379096
chr8	142050000	142300000	1	0,025656281	0,011869455
chr9	0	250000	1	5,06E-05	0,02119322
chr9	50000	300000	1	1,26E-05	0,02106212
chr9	100000	350000	1	7,27E-05	0,020873299
chr9	150000	400000	1	0,0002176	0,020742351
chr9	200000	450000	1	0,000131431	0,020773167
chr9	250000	500000	1	0,0103943	0,019550666
chr9	33400000	33650000	1	0,011230174	0,00728277
chr9	33450000	33700000	1	0,009093171	0,006847603
chr9	35600000	35850000	1	0,023851375	0,002207084

chr9	35650000	35900000	1	0,000883372	0,002413901
chr9	35700000	35950000	1	0,00093831	0,002469489
chr9	35750000	36000000	1	0,003049582	0,002438251
chr9	36050000	36300000	1	0,02332881	0,002774718
chr9	40600000	40850000	1	0,042723836	0,034368832
chr9	40650000	40900000	1	0,002388037	0,037038398
chr9	40700000	40950000	1	0,003000921	0,038225955
chr9	40750000	41000000	1	0,002640762	0,03773537
chr9	40800000	41050000	1	0,040275919	0,036609075
chr9	41450000	41700000	1	0,022034007	0,101772772
chr9	67000000	67250000	1	0,041149196	0,039279283
chr9	67400000	67650000	1	0,027354876	0,068049571
chr9	74950000	75200000	1	0,014938996	0,039552846
chr9	95450000	95700000	1	0,033433126	0,011747906
chr9	95700000	95950000	1	0,007860675	0,010622886
chr9	95750000	96000000	1	0,037903786	0,011159096
chr9	95850000	96100000	1	0,02224914	0,010328184
chr9	100050000	100300000	1	0,020402199	0,019670206
chr9	100100000	100350000	1	0,013271335	0,021061762
chr9	114450000	114700000	1	0,003981958	0,016334297
chr9	114500000	114750000	1	0,000379416	0,017176398
chr9	114550000	114800000	1	0,001653742	0,017511824
chr9	114600000	114850000	1	0,00132253	0,017459969
chr9	114650000	114900000	1	0,000233088	0,016987366
chr9	114700000	114950000	1	0,002206407	0,017627514
chr9	114750000	115000000	1	0,03156235	0,01853442
chr9	115050000	115300000	1	0,040779706	0,023384734
chr9	115100000	115350000	1	0,003625675	0,02262677
chr9	115150000	115400000	1	0,026331555	0,020942161
chr9	123750000	124000000	1	0,011639194	0,004620397
chr9	123800000	124050000	1	0,001294011	0,004448531
chr9	126800000	127050000	1	0,040559877	0,021386095
chr9	126850000	127100000	1	0,000581254	0,023205004
chr9	126900000	127150000	1	0,000725945	0,024051559
chr9	126950000	127200000	1	0,001791068	0,024169753
chr9	127000000	127250000	1	0,001880825	0,023880225
chr9	127050000	127300000	1	0,003587724	0,023478657
chr9	127100000	127350000	1	0,020606112	0,023139104
chr9	127600000	127850000	1	0,027802733	0,0097788
chr9	127650000	127900000	1	0,01521456	0,010331876
chr9	127850000	128100000	1	0,024991253	0,01068822
chr9	129550000	129800000	1	0,012146194	0,012354864
chr9	129600000	129850000	1	0,008371607	0,013216776
chr9	130600000	130850000	1	0,049399472	0,005276378
chr9	130650000	130900000	1	0,001865364	0,0057843
chr9	130700000	130950000	1	0,000957815	0,005703198

chr9	130750000	131000000	1	0,014763596	0,005459426
chr9	133000000	133250000	1	0,010207312	0,012695477
chr9	133050000	133300000	1	0,000341969	0,012695477
chr9	133100000	133350000	1	0,002738044	0,012695477
chr9	133150000	133400000	1	0,012941967	0,012715574
chr9	133200000	133450000	1	0,035613015	0,012554752
chr9	139600000	139850000	1	0,045083558	0,001929085
chr10	8600000	8850000	1	0,01775155	0,016425784
chr10	8650000	8900000	1	0,001110711	0,016285275
chr10	8700000	8950000	1	8,68E-05	0,016021323
chr10	8750000	9000000	1	0,000902755	0,015690526
chr10	8800000	9050000	1	0,004119344	0,015336845
chr10	8850000	9100000	1	0,008446972	0,014980698
chr10	8900000	9150000	1	0,012496494	0,014596002
chr10	8950000	9200000	1	0,020488331	0,01410001
chr10	10150000	10400000	1	0,047327947	0,018637171
chr10	16550000	16800000	1	0,018311185	0,004532525
chr10	28900000	29150000	1	0,027489581	0,018843282
chr10	28950000	29200000	1	0,006596231	0,019190816
chr10	29000000	29250000	1	0,004191432	0,019190816
chr10	29050000	29300000	1	0,003402774	0,019190816
chr10	29100000	29350000	1	0,000997677	0,019190816
chr10	29150000	29400000	1	0,002495202	0,019190816
chr10	45600000	45850000	1	0,009006713	0,00409796
chr10	45650000	45900000	1	0,045934285	0,004416209
chr10	63750000	64000000	1	0,014240199	0,021514565
chr10	63800000	64050000	1	0,035877342	0,020040643
chr10	70250000	70500000	1	0,002544862	0,009214636
chr10	70300000	70550000	1	2,04E-05	0,009676969
chr10	70350000	70600000	1	4,71E-05	0,009780519
chr10	70400000	70650000	1	0,000235401	0,00968489
chr10	70450000	70700000	1	0,000244924	0,009591659
chr10	70500000	70750000	1	0,004291681	0,009628505
chr10	73950000	74200000	1	0,006888045	0,005688699
chr10	75000000	75250000	1	0,014256486	0,013499865
chr10	88600000	88850000	1	0,005224054	0,018200685
chr10	88650000	88900000	1	2,21E-05	0,018329663
chr10	88700000	88950000	1	0,000351206	0,018902653
chr10	88750000	89000000	1	0,017018352	0,019983274
chr10	89050000	89300000	1	0,015906104	0,023116132
chr10	89100000	89350000	1	0,028896478	0,024564899
chr10	89500000	89750000	1	0,010747955	0,013089426
chr10	89550000	89800000	1	4,31E-05	0,012948154
chr10	89600000	89850000	1	0,000756772	0,012492408
chr10	89650000	89900000	1	0,024741839	0,011765026
chr10	89950000	90200000	1	0,036466375	0,009009252

chr10	90600000	90850000	1	0,002465589	0,035897457
chr10	90650000	90900000	1	0,006875265	0,037522693
chr10	90950000	91200000	1	0,029400168	0,031734901
chr10	91000000	91250000	1	0,008660844	0,027808874
chr10	91050000	91300000	1	0,011397835	0,026691288
chr10	94600000	94850000	1	0,00426814	0,013041215
chr10	95900000	96150000	1	0,040137334	0,010028882
chr10	102850000	103100000	1	0,019261165	0,009287986
chr10	102900000	103150000	1	0,007236767	0,008880129
chr10	102950000	103200000	1	0,040498862	0,008580498
chr10	103000000	103250000	1	0,042868462	0,008580498
chr10	103050000	103300000	1	0,008548905	0,008580498
chr10	103100000	103350000	1	0,025093077	0,008580498
chr10	103350000	103600000	1	0,021514314	0,010276147
chr10	103400000	103650000	1	0,016420357	0,010951335
chr10	104350000	104600000	1	0,007036813	0,018820979
chr10	104400000	104650000	1	0,00038375	0,018370035
chr10	104450000	104700000	1	0,005085613	0,018370035
chr10	104500000	104750000	1	0,0416211	0,018370035
chr10	104900000	105150000	1	0,020904227	0,01167414
chr10	104950000	105200000	1	0,005114011	0,011425888
chr10	105000000	105250000	1	0,010808019	0,010936192
chr10	105550000	105800000	1	0,040747525	0,007904708
chr10	116200000	116450000	1	0,007437729	0,031115773
chr10	116250000	116500000	1	0,017018212	0,029044878
chr10	117500000	117750000	1	0,040180522	0,025012692
chr10	121050000	121300000	1	0,033834249	0,006261186
chr11	8750000	9000000	1	0,039252146	0,015081839
chr11	8800000	9050000	1	0,009380908	0,016375715
chr11	10550000	10800000	1	0,005725132	0,009937564
chr11	10600000	10850000	1	0,007676926	0,010403719
chr11	12300000	12550000	1	0,025798088	0,02270653
chr11	12350000	12600000	1	0,00167364	0,024179686
chr11	12400000	12650000	1	0,028963297	0,02263156
chr11	14350000	14600000	1	0,029560052	0,029610928
chr11	45850000	46100000	1	0,049762941	0,013053135
chr11	45900000	46150000	1	0,00105264	0,014098862
chr11	45950000	46200000	1	8,79E-05	0,014645968
chr11	46000000	46250000	1	0,000571549	0,014822418
chr11	46050000	46300000	1	0,001199255	0,014772673
chr11	46100000	46350000	1	0,001567578	0,014615365
chr11	46150000	46400000	1	0,002344417	0,014424223
chr11	46200000	46450000	1	0,006137418	0,014229215
chr11	46250000	46500000	1	0,028242597	0,014035991
chr11	46800000	47050000	1	0,001499493	0,005026812
chr11	46850000	47100000	1	0,004694521	0,005026812

chr11	46900000	47150000	1	0,004329283	0,005026812
chr11	46950000	47200000	1	0,001338221	0,005144789
chr11	47000000	47250000	1	0,00078062	0,005266812
chr11	47050000	47300000	1	0,004738644	0,005345327
chr11	47100000	47350000	1	0,036926129	0,005384044
chr11	47350000	47600000	1	0,010957537	0,007006722
chr11	47400000	47650000	1	0,003226998	0,007006722
chr11	61550000	61800000	1	0,012064798	0,005480719
chr11	64400000	64650000	1	0,013871593	0,002136695
chr11	64500000	64750000	1	0,038340051	0,002232272
chr11	64550000	64800000	1	0,018286028	0,002145918
chr11	65050000	65300000	1	0,01183303	0,004990453
chr11	66950000	67200000	1	0,027834716	0,004592453
chr11	67000000	67250000	1	0,001871586	0,004522266
chr11	67050000	67300000	1	0,000845659	0,004382244
chr11	67100000	67350000	1	0,011701619	0,004211254
chr11	67150000	67400000	1	0,043137951	0,004062844
chr11	67250000	67500000	1	0,018449481	0,004001597
chr11	67300000	67550000	1	0,001653477	0,004096257
chr11	67350000	67600000	1	0,002006489	0,004096257
chr11	67400000	67650000	1	0,010783934	0,004096257
chr11	67450000	67700000	1	0,01222428	0,004096257
chr11	67500000	67750000	1	0,003225004	0,004096257
chr11	67550000	67800000	1	0,00021769	0,004222846
chr11	67600000	67850000	1	0,000241655	0,004326018
chr11	67650000	67900000	1	0,00051477	0,004366925
chr11	67700000	67950000	1	0,000159149	0,004340414
chr11	67750000	68000000	1	0,000288602	0,004275325
chr11	67800000	68050000	1	0,004171772	0,00421873
chr11	67850000	68100000	1	0,019423035	0,004208954
chr11	77450000	77700000	1	0,005896317	0,006744876
chr11	122800000	123050000	1	0,020850994	0,011919552
chr11	122850000	123100000	1	0,004368796	0,011287508
chr11	128100000	128350000	1	0,015748518	0,031407709
chr11	128150000	128400000	1	0,011584844	0,033711542
chr11	130000000	130250000	1	0,018762839	0,010380231
chr11	130050000	130300000	1	0,002727496	0,009963793
chr11	130100000	130350000	1	0,044010533	0,009190397
chr12	600000	850000	1	0,004742582	0,006441908
chr12	650000	900000	1	0,004035998	0,00647184
chr12	700000	950000	1	0,005117138	0,006277228
chr12	2700000	2950000	1	0,042811122	0,003248285
chr12	2750000	3000000	1	4,50E-05	0,00363248
chr12	2800000	3050000	1	0,00037349	0,003692112
chr12	2850000	3100000	1	0,000633252	0,003634104
chr12	2900000	3150000	1	0,010778204	0,00360603

chr12	12800000	13050000	1	0,046147702	0,005520832
chr12	14900000	15150000	1	0,047482933	0,025667413
chr12	14950000	15200000	1	0,00163204	0,028019494
chr12	15000000	15250000	1	0,00966507	0,026754569
chr12	27500000	27750000	1	0,03278037	0,014813993
chr12	39100000	39350000	1	0,041692198	0,041224427
chr12	39150000	39400000	1	0,001852483	0,040219712
chr12	39200000	39450000	1	0,005403068	0,038820113
chr12	43350000	43600000	1	0,047185537	0,031339292
chr12	43400000	43650000	1	0,024079335	0,028789893
chr12	49350000	49600000	1	0,035762337	0,006027673
chr12	49400000	49650000	1	0,002801386	0,006431318
chr12	49450000	49700000	1	0,027685025	0,006698479
chr12	49650000	49900000	1	0,049098433	0,0054758
chr12	49700000	49950000	1	0,008055265	0,005778208
chr12	50000000	50250000	1	0,039761653	0,007291423
chr12	50050000	50300000	1	0,002580156	0,007291423
chr12	50100000	50350000	1	0,001467115	0,007291423
chr12	50150000	50400000	1	0,020581963	0,007445055
chr12	50350000	50600000	1	0,012886615	0,006747893
chr12	50400000	50650000	1	0,000677434	0,006443694
chr12	50450000	50700000	1	0,000556088	0,006234493
chr12	50500000	50750000	1	0,000474553	0,006279624
chr12	50550000	50800000	1	0,012813923	0,00584119
chr12	53300000	53550000	1	0,011427625	0,006029401
chr12	53450000	53700000	1	0,030122583	0,006096375
chr12	53500000	53750000	1	0,015910793	0,005989962
chr12	56150000	56400000	1	0,033244617	0,006731298
chr12	56400000	56650000	1	0,048165436	0,005982237
chr12	56450000	56700000	1	0,002751934	0,005617787
chr12	56500000	56750000	1	0,000105815	0,005400905
chr12	56550000	56800000	1	0,000514416	0,005133944
chr12	56600000	56850000	1	0,005536875	0,004938192
chr12	56650000	56900000	1	0,019416904	0,00479464
chr12	56700000	56950000	1	0,019155442	0,004760673
chr12	56750000	57000000	1	0,004195226	0,004908373
chr12	56800000	57050000	1	0,019450478	0,005271936
chr12	57150000	57400000	1	0,014275775	0,007733551
chr12	58850000	59100000	1	0,038472946	0,020286775
chr12	58900000	59150000	1	0,005394383	0,01932397
chr12	60250000	60500000	1	0,003792148	0,041314046
chr12	60300000	60550000	1	0,048109482	0,03812675
chr12	64650000	64900000	1	0,029284777	0,017847261
chr12	64700000	64950000	1	0,005599844	0,019027638
chr12	65950000	66200000	1	0,000708995	0,029700175
chr12	66000000	66250000	1	0,000769446	0,029405416

chr12	66050000	66300000	1	0,000806514	0,029416205
chr12	66100000	66350000	1	0,000114067	0,029577574
chr12	66150000	66400000	1	2,10E-06	0,029561246
chr12	66200000	66450000	1	4,01E-05	0,028945315
chr12	66250000	66500000	1	0,008366416	0,027301261
chr12	74600000	74850000	1	0,014452906	0,014261063
chr12	74800000	75050000	1	0,028100898	0,012839824
chr12	74850000	75100000	1	0,001741988	0,012888073
chr12	74900000	75150000	1	2,24E-05	0,012780729
chr12	74950000	75200000	1	0,000501721	0,01237528
chr12	75000000	75250000	1	0,021557924	0,011633562
chr12	78400000	78650000	1	0,02891677	0,036242711
chr12	78450000	78700000	1	0,001079642	0,038532489
chr12	78500000	78750000	1	0,004054882	0,03990398
chr12	90600000	90850000	1	0,017141475	0,018161756
chr12	90650000	90900000	1	0,003811721	0,017356524
chr12	94000000	94250000	1	0,013241756	0,045477966
chr12	94050000	94300000	1	0,019782937	0,042522014
chr12	95650000	95900000	1	0,045204133	0,019817994
chr12	95950000	96200000	1	0,027448433	0,016523352
chr12	96000000	96250000	1	0,027358474	0,017254125
chr12	96050000	96300000	1	0,048668422	0,017278607
chr12	96100000	96350000	1	0,022139306	0,016021674
chr12	105250000	105500000	1	0,010123571	0,023627764
chr12	109800000	110050000	1	0,046234282	0,014020276
chr12	111850000	112100000	1	0,002704664	0,010937005
chr12	111900000	112150000	1	0,004202393	0,011472844
chr12	111950000	112200000	1	0,002080145	0,011461007
chr12	112000000	112250000	1	0,001201887	0,011168203
chr12	112050000	112300000	1	0,005741782	0,010880807
chr12	112100000	112350000	1	0,004270155	0,010809916
chr12	112150000	112400000	1	0,017117483	0,011014644
chr12	117250000	117500000	1	0,037421577	0,013358807
chr12	120750000	121000000	1	0,039488339	0,009595081
chr12	121100000	121350000	1	0,008147948	0,013269333
chr12	121150000	121400000	1	0,00022128	0,013100421
chr12	121200000	121450000	1	0,000449623	0,013100421
chr12	121250000	121500000	1	0,008523195	0,013100421
chr12	122950000	123200000	1	0,042301975	0,002823883
chr13	23900000	24150000	1	0,017391606	0,018789629
chr13	38100000	38350000	1	0,005544972	0,035702923
chr13	38150000	38400000	1	0,019302072	0,0335804
chr13	39300000	39550000	1	0,007474472	0,021451209
chr13	97650000	97900000	1	0,035423161	0,023383861
chr13	98150000	98400000	1	0,010882313	0,011585285
chr13	98200000	98450000	1	0,004150084	0,011585285

chr13	104450000	104700000	1	0,014751326	0,013242299
chr14	19650000	19900000	1	0,049222516	0,01550294
chr14	19700000	19950000	1	0,005920352	0,014227669
chr14	19750000	20000000	1	0,008613699	0,014554462
chr14	19800000	20050000	1	0,023586099	0,013234059
chr14	22900000	23150000	1	0,013763797	0,029546292
chr14	22950000	23200000	1	0,018398207	0,031143193
chr14	50550000	50800000	1	0,021329994	0,005141781
chr14	50600000	50850000	1	0,040506008	0,005141781
chr14	53150000	53400000	1	0,010294727	0,005855984
chr14	55200000	55450000	1	0,047517544	0,010800722
chr14	64450000	64700000	1	0,03535709	0,014706184
chr14	64500000	64750000	1	0,020877391	0,014706184
chr14	67950000	68200000	1	0,015581631	0,017304427
chr14	68000000	68250000	1	0,000511868	0,017284518
chr14	68050000	68300000	1	5,03E-05	0,017284518
chr14	68100000	68350000	1	9,84E-05	0,017284518
chr14	68150000	68400000	1	3,08E-05	0,017284518
chr14	68200000	68450000	1	3,95E-05	0,017116807
chr14	68250000	68500000	1	0,000126824	0,016967255
chr14	68300000	68550000	1	6,54E-05	0,016967332
chr14	68350000	68600000	1	0,000569529	0,017125489
chr14	68400000	68650000	1	0,011046624	0,01732834
chr14	68450000	68700000	1	0,049846696	0,017395718
chr14	68550000	68800000	1	0,028387352	0,015058119
chr14	68600000	68850000	1	0,002874101	0,015500607
chr14	68650000	68900000	1	0,005834039	0,016156648
chr14	68700000	68950000	1	0,040867046	0,016818943
chr14	68800000	69050000	1	0,046483841	0,016818943
chr14	68850000	69100000	1	0,011913475	0,016818943
chr14	68900000	69150000	1	0,001069688	0,016818943
chr14	68950000	69200000	1	2,26E-05	0,017320534
chr14	69000000	69250000	1	9,30E-06	0,017583679
chr14	69050000	69300000	1	0,000623025	0,017639665
chr14	69100000	69350000	1	0,009196995	0,017603042
chr14	69150000	69400000	1	0,045223163	0,017626921
chr14	69350000	69600000	1	0,018985232	0,019928774
chr14	69400000	69650000	1	0,002007538	0,021846816
chr14	69450000	69700000	1	0,000150136	0,021431648
chr14	69500000	69750000	1	0,001878523	0,020806903
chr14	69550000	69800000	1	0,015407866	0,020009486
chr14	73550000	73800000	1	0,020899366	0,018006722
chr14	73600000	73850000	1	0,009570703	0,019276993
chr14	74750000	75000000	1	0,009853535	0,023627483
chr14	74800000	75050000	1	0,001319198	0,023627483
chr14	74850000	75100000	1	0,029796881	0,023627483

chr14	77900000	78150000	1	0,029191522	0,018755167
chr14	84200000	84450000	1	0,028819201	0,030683428
chr14	84700000	84950000	1	0,003086282	0,013125307
chr14	84750000	85000000	1	0,000495908	0,013177439
chr14	84800000	85050000	1	0,000349539	0,013026378
chr14	84850000	85100000	1	0,000303124	0,013026378
chr14	84900000	85150000	1	0,00498029	0,013026378
chr14	84950000	85200000	1	0,028939679	0,013026378
chr14	85100000	85350000	1	0,044782962	0,012275947
chr14	85150000	85400000	1	0,007347487	0,011777994
chr14	100250000	100500000	1	0,010348491	0,01503821
chr15	34500000	34750000	1	0,004085224	0,016103139
chr15	34550000	34800000	1	0,000417649	0,016603051
chr15	34600000	34850000	1	0,009746685	0,015867777
chr15	36500000	36750000	1	0,013492955	0,032698286
chr15	36550000	36800000	1	0,001244959	0,034252076
chr15	36600000	36850000	1	0,014862275	0,035395361
chr15	39150000	39400000	1	0,025825126	0,030912394
chr15	39200000	39450000	1	0,004007661	0,033072143
chr15	40000000	40250000	1	0,031640679	0,02484448
chr15	40050000	40300000	1	0,019546104	0,026755604
chr15	41350000	41600000	1	0,019302072	0,011016283
chr15	42050000	42300000	1	0,034437719	0,021489582
chr15	42100000	42350000	1	0,002246679	0,021289785
chr15	42150000	42400000	1	0,00037001	0,020704872
chr15	42200000	42450000	1	0,011351869	0,019680191
chr15	42650000	42900000	1	0,013569247	0,011443754
chr15	42700000	42950000	1	0,045246197	0,012169522
chr15	43050000	43300000	1	0,027061271	0,014671614
chr15	43100000	43350000	1	0,007503476	0,015347225
chr15	43150000	43400000	1	0,004490672	0,015847853
chr15	43200000	43450000	1	0,009582783	0,01618355
chr15	43250000	43500000	1	0,031993833	0,016759893
chr15	43450000	43700000	1	0,045060409	0,018575558
chr15	43500000	43750000	1	0,005346493	0,019305636
chr15	43550000	43800000	1	0,005896564	0,020132373
chr15	44650000	44900000	1	0,036800435	0,002902939
chr15	54150000	54400000	1	0,00883101	0,020978825
chr15	58650000	58900000	1	0,048516481	0,038601044
chr15	59250000	59500000	1	0,001728598	0,014359014
chr15	59300000	59550000	1	0,004608304	0,014359014
chr15	59350000	59600000	1	0,02252852	0,014359014
chr15	59800000	60050000	1	0,010825314	0,02128993
chr15	63150000	63400000	1	0,04300168	0,039201973
chr15	63200000	63450000	1	0,012411606	0,04260019
chr15	64850000	65100000	1	0,03739215	0,010265519

chr15	67150000	67400000	1	0,047842378	0,004787254
chr15	67700000	67950000	1	0,013526202	0,019452159
chr15	67750000	68000000	1	0,001347508	0,019452159
chr15	67800000	68050000	1	0,014473853	0,019452159
chr15	74550000	74800000	1	0,001248071	0,012832844
chr15	74600000	74850000	1	0,002187467	0,013409662
chr15	74650000	74900000	1	0,006818995	0,013439043
chr15	74700000	74950000	1	0,021904187	0,013211609
chr15	76300000	76550000	1	0,028445834	0,016629352
chr15	76350000	76600000	1	0,007616239	0,015647467
chr15	78450000	78700000	1	0,006398271	0,008402438
chr15	89450000	89700000	1	0,015469721	0,02485184
chr15	89500000	89750000	1	0,019780306	0,026582009
chr15	89950000	90200000	1	0,006478815	0,015357135
chr15	90000000	90250000	1	0,015856963	0,014531181
chr15	90300000	90550000	1	0,00660748	0,017531703
chr15	90350000	90600000	1	0,013392941	0,018361853
chr15	101300000	101550000	1	0,015457687	0,015850155
chr15	101650000	101900000	1	0,036395958	0,012215983
chr16	3300000	3550000	1	0,021205637	0,017063855
chr16	3900000	4150000	1	0,012524277	0,005940281
chr16	3950000	4200000	1	0,004008662	0,00558526
chr16	4000000	4250000	1	0,034999471	0,005428399
chr16	4350000	4600000	1	0,006473962	0,007262584
chr16	4400000	4650000	1	0,029661879	0,006691313
chr16	11450000	11700000	1	0,007050547	0,004428495
chr16	11500000	11750000	1	0,018562682	0,004513888
chr16	15800000	16050000	1	0,029460161	0,011871138
chr16	18950000	19200000	1	0,004998973	0,013243535
chr16	19000000	19250000	1	0,018274389	0,013152911
chr16	19650000	19900000	1	0,004436964	0,024891282
chr16	19700000	19950000	1	0,028641186	0,023007874
chr16	24600000	24850000	1	0,010280572	0,027062068
chr16	29800000	30050000	1	0,021640369	0,005859085
chr16	29850000	30100000	1	0,000634876	0,005506976
chr16	29900000	30150000	1	0,001068222	0,005327625
chr16	29950000	30200000	1	0,000824254	0,005296027
chr16	30000000	30250000	1	0,002299091	0,005217099
chr16	30500000	30750000	1	0,005332772	0,001901321
chr16	30550000	30800000	1	0,01297985	0,00175464
chr16	61750000	62000000	1	0,025804493	0,049547406
chr16	68550000	68800000	1	0,028569696	0,025832426
chr16	74850000	75100000	1	0,012094578	0,020583491
chr16	84300000	84550000	1	0,021862579	0,017615325
chr16	84350000	84600000	1	0,011753936	0,01884136
chr16	84700000	84950000	1	0,003422971	0,01325806

chr16	84750000	85000000	1	0,000590844	0,012934746
chr16	84800000	85050000	1	0,009097349	0,012589148
chr17	200000	450000	1	0,031061791	0,009805689
chr17	250000	500000	1	0,032253386	0,009805689
chr17	2000000	2250000	1	0,005644408	0,020552926
chr17	4150000	4400000	1	0,01526581	0,020738531
chr17	15150000	15400000	1	0,031049667	0,018296345
chr17	15200000	15450000	1	0,011033827	0,019978478
chr17	19250000	19500000	1	0,02951649	0,008451187
chr17	26900000	27150000	1	0,017232829	0,007932369
chr17	26950000	27200000	1	0,013697243	0,008435907
chr17	28600000	28850000	1	0,042469216	0,022707648
chr17	28650000	28900000	1	0,008537323	0,02407457
chr17	28700000	28950000	1	0,00072007	0,02492483
chr17	28750000	29000000	1	0,007821446	0,025333779
chr17	30300000	30550000	1	0,01508163	0,016872709
chr17	35500000	35750000	1	0,006512121	0,020973256
chr17	35550000	35800000	1	0,000170236	0,022140734
chr17	35600000	35850000	1	0,001749373	0,022613997
chr17	35650000	35900000	1	0,011205121	0,02070371
chr17	35700000	35950000	1	0,04347578	0,019733991
chr17	40600000	40850000	1	0,0279215	0,001902488
chr17	40650000	40900000	1	0,023613901	0,001809406
chr17	40800000	41050000	1	0,023913487	0,001731975
chr17	40850000	41100000	1	0,001870738	0,001790818
chr17	40900000	41150000	1	0,018778092	0,001894139
chr17	41400000	41650000	1	0,017779822	0,003254393
chr17	42350000	42600000	1	0,029674416	0,005851082
chr17	42400000	42650000	1	0,001963391	0,006174192
chr17	42450000	42700000	1	6,07E-05	0,006400884
chr17	42500000	42750000	1	0,000149314	0,006518663
chr17	42550000	42800000	1	0,000195548	0,00653604
chr17	42600000	42850000	1	3,92E-05	0,006483722
chr17	42650000	42900000	1	6,34E-05	0,006406925
chr17	42700000	42950000	1	0,000670188	0,006350902
chr17	42750000	43000000	1	0,001705815	0,006346574
chr17	42800000	43050000	1	0,001284073	0,006401926
chr17	42850000	43100000	1	0,000251834	0,00650189
chr17	42900000	43150000	1	5,76E-05	0,006612574
chr17	42950000	43200000	1	0,001391108	0,006388395
chr17	43000000	43250000	1	0,016305841	0,006109264
chr17	44750000	45000000	1	0,018919582	0,011590426
chr17	44950000	45200000	1	0,014223465	0,010694219
chr17	45200000	45450000	1	0,029136242	0,011286533
chr17	45650000	45900000	1	0,008792104	0,005720043
chr17	45700000	45950000	1	0,019072421	0,005758097

chr17	45750000	46000000	1	0,00637089	0,005591836
chr17	45800000	46050000	1	0,015570898	0,005213293
chr17	61750000	62000000	1	0,02969748	0,009459983
chr17	62300000	62550000	1	0,027073928	0,026025051
chr17	65300000	65550000	1	0,028254101	0,015932784
chr17	73100000	73350000	1	0,019462801	0,003428864
chr17	73150000	73400000	1	0,000672095	0,003700255
chr17	73200000	73450000	1	0,006012764	0,003797445
chr17	73250000	73500000	1	0,022743248	0,003791917
chr17	73300000	73550000	1	0,048564843	0,003731025
chr17	73500000	73750000	1	0,038179353	0,002916533
chr17	73550000	73800000	1	0,005294242	0,002977444
chr17	73600000	73850000	1	0,005098546	0,0031391
chr17	73900000	74150000	1	0,048625719	0,0037813
chr18	9250000	9500000	1	0,009029338	0,014307286
chr18	12650000	12900000	1	0,013423842	0,028851992
chr18	12700000	12950000	1	0,036926129	0,026651569
chr18	18950000	19200000	1	0,009177692	0,014909696
chr18	19000000	19250000	1	0,001562152	0,014459067
chr18	19050000	19300000	1	0,002435415	0,014384583
chr18	19100000	19350000	1	0,001445447	0,013828231
chr18	19150000	19400000	1	0,034951351	0,012833785
chr18	20000000	20250000	1	0,030628322	0,028730664
chr18	20050000	20300000	1	0,001135243	0,030615122
chr18	20100000	20350000	1	0,004619438	0,031761868
chr18	43400000	43650000	1	0,035086186	0,014022047
chr18	55350000	55600000	1	0,042536806	0,028640988
chr18	68850000	69100000	1	0,020943944	0,012416873
chr18	68900000	69150000	1	5,78E-05	0,012427721
chr18	68950000	69200000	1	5,62E-07	0,012456849
chr18	69000000	69250000	1	7,28E-06	0,012300147
chr18	69050000	69300000	1	0,002373003	0,011749473
chr18	71300000	71550000	1	0,006302851	0,014541674
chr18	71350000	71600000	1	0,019992439	0,014541674
chr20	3250000	3500000	1	0,016445351	0,016180862
chr20	3300000	3550000	1	0,010219509	0,016180862
chr20	13850000	14100000	1	0,005067274	0,009574489
chr20	13900000	14150000	1	9,75E-05	0,009622595
chr20	13950000	14200000	1	4,58E-06	0,009575511
chr20	14000000	14250000	1	0,000248119	0,009292142
chr20	14050000	14300000	1	0,025092824	0,008649286
chr20	22600000	22850000	1	0,005929833	0,034516365
chr20	22650000	22900000	1	0,0259464	0,036473975
chr20	32800000	33050000	1	0,003068942	0,009784029
chr20	32850000	33100000	1	0,002515156	0,009384607
chr20	33800000	34050000	1	0,027708427	0,005190609

chr20	33850000	34100000	1	0,003910086	0,005541989
chr20	34150000	34400000	1	0,02853912	0,004727271
chr20	34850000	35100000	1	0,021932768	0,027786527
chr20	35350000	35600000	1	0,0142734	0,011806019
chr20	35600000	35850000	1	0,035002504	0,013734726
chr20	36750000	37000000	1	0,021839739	0,027931846
chr20	48650000	48900000	1	0,016708217	0,004276018
chr20	49100000	49350000	1	0,010846745	0,005771975
chr19	6250000	6500000	1	0,005490979	0,002998862
chr19	6300000	6550000	1	0,029829667	0,002843228
chr19	6400000	6650000	1	0,026113141	0,002897561
chr19	8200000	8450000	1	0,01251697	0,008084256
chr19	8250000	8500000	1	0,000185984	0,00870058
chr19	8300000	8550000	1	0,000175949	0,008759202
chr19	8350000	8600000	1	0,000511152	0,008985964
chr19	8400000	8650000	1	0,002181696	0,009097348
chr19	8450000	8700000	1	0,00137178	0,00887207
chr19	8500000	8750000	1	0,017782768	0,008192305
chr19	9950000	10200000	1	0,020567115	0,014769872
chr19	10500000	10750000	1	0,007571174	0,003723356
chr19	10550000	10800000	1	0,005681915	0,003697129
chr19	10600000	10850000	1	0,003935823	0,003673366
chr19	10650000	10900000	1	0,000362593	0,003613155
chr19	10700000	10950000	1	0,000414157	0,003608449
chr19	10750000	11000000	1	0,001426983	0,003608449
chr19	10800000	11050000	1	0,001032111	0,003608449
chr19	10850000	11100000	1	0,000157584	0,003608449
chr19	10900000	11150000	1	1,66E-05	0,003563254
chr19	10950000	11200000	1	0,000975313	0,003672828
chr19	11000000	11250000	1	0,033748637	0,003920699
chr19	18050000	18300000	1	0,041212935	0,007378854
chr19	39550000	39800000	1	0,04353378	0,010652959
chr19	45800000	46050000	1	0,014045207	0,005976711
chr19	45850000	46100000	1	0,004271789	0,006196535
chr19	45900000	46150000	1	0,001969751	0,006361403
chr19	45950000	46200000	1	0,003636849	0,006954578
chr19	46000000	46250000	1	0,026928244	0,007341722
chr19	46500000	46750000	1	0,048327504	0,01582329
chr19	46550000	46800000	1	0,002598287	0,01582329
chr19	46600000	46850000	1	0,018102895	0,01582329
chr19	47550000	47800000	1	0,042965492	0,004202697
chr19	47600000	47850000	1	0,023556346	0,004333586
chr19	47650000	47900000	1	0,026299425	0,004467784
chr19	47700000	47950000	1	0,025687641	0,004564097
chr19	47750000	48000000	1	0,011673008	0,004578424
chr19	47800000	48050000	1	0,001582537	0,004467603

chr19	47850000	48100000	1	0,011244298	0,00418277
chr19	49550000	49800000	1	0,028957718	0,015981112
chr19	54300000	54550000	1	0,045254873	0,015076774
chr22	30300000	30550000	1	0,034290539	0,027435684
chr22	36350000	36600000	1	0,033226618	0,018306533
chr22	39300000	39550000	1	0,036229898	0,01214895
chr22	39350000	39600000	1	0,044989045	0,013090695
chr22	41450000	41700000	1	0,007920173	0,008017088
chr22	44850000	45100000	1	0,018485084	0,008708927
chr22	44900000	45150000	1	0,000227689	0,008947258
chr22	44950000	45200000	1	0,028733413	0,009761104
chr22	45850000	46100000	1	0,045345934	0,005326161
chr22	46000000	46250000	1	0,010693866	0,005571038
chr22	46050000	46300000	1	0,002553924	0,005860229
chr22	46100000	46350000	1	0,022285492	0,006054473
chr22	46150000	46400000	1	0,046216277	0,005407212
chr22	46200000	46450000	1	0,015292344	0,005607909
chr22	46750000	47000000	1	0,022518009	0,014991937
chr21	34600000	34850000	1	0,02694921	0,017370956

Table S4
Validation of Repli-seq by q-PCR analysis*.

**Probe Symbol**	**Primers**	**Chr**	**Primers' Genomic coordinates**	**Repli-seq Result**	**qPCR in replicate**
Chr2_1	CCATTGGATGCAGCACAAACA<br>GGTGAGTGCTCACATCTTCAC	2	154216022<br>154216104	LtE	LtE
Chr2_2	AGTCTAAGGTTTTCATTCTGGCA<br>AGGGAGAGTAACTGCCTGGT	2	154174096<br>154174184	LtE	LtE
Chr2_3	CCAAAGCCATGTGACTGCTTC<br>CCAGTGTGTCTGGAAGAGACC	2	154272746<br>154272819	LtE	LtE
Chr3-1	AGACTCAGCAGTAAGCACCG<br>CATTCATGAAGCGGGAACCG	3	54524327<br>54524423	LtE	LtE
Chr3_2	ATCCCACAAACAGCCCAACT<br>TCGCACATGTCAGAGGAACA	3	16923716<br>16923794	LtE	LtE
Chr4-1	TCCAAGACCACTCCCAGTGT<br>AGTCAGTGCCATGGTTGGTG	4	13268816<br>13268908	LtE	LtE
Chr4_2	AGCATAGTCTAAATCCTACAGAGAG<br>GTCCCTTTGATCTTTGTGGTGT	4	74703010<br>74703077	LtE	EtL
Chr5_2	TGGAGAGTCACCTCTGCAAATAC<br>GGCAAGTTTCCCTGCGAACA	5	25553179<br>25553264	LtE	LtE
Chr7_p4	CCTGGTACCTGGTATAGAGCTGTT<br>TTCTGGTGGGCTTTATGGAC	7	14838800<br>14838873	NE	NE
Chr7_p5	CAACATAACACCTGAAGTCCTAGC<br>TCTTTTTCAATTTTGATGCACTTT	7	15324826<br>15324900	NE	NE
7a_pos	TGTGGAAGGGGATTTTCTACA<br>GCCAGGCCACGTCATATAG	7	110060547<br>110060621	NE	NE
7b_pos	CCTGCAGAAAATCCCGTCT<br>ATCCTGGAGGGCATGAGTC	7	110241938<br>110242004	NE	NE
Chr10_p3	CCCAGAGAGGCTGATTTCAC<br>CAGAGAGGCTTTGAGAGCACA	10	11560213<br>11560277	NE	NE
Chr10_p4	CTGAGGCCGACAGAGAAGTT<br>GGAGGAGAAAGCTGACAGGA	10	12288594<br>12288655	NE	NE
Chr10_p5	GCCCTCCTGTGATTTCCTG<br>ACAGCCACTGATCTGGCTTC	10	11349302<br>11349366	NE	NE
Chr10_p6	CGCAAGCACACAAGAGGAT<br>CCTTGTAACATGACTGACAAAGAAC	10	12617922<br>12617997	NE	NE
Chr11_1	GGTGCTTTTCCCCATGAAA<br>AAAAGCAACAAATACGAATCCTCT	11	114014724<br>114014783	LtE	LtE
Chr11_2	CATGCTGCTGTAGCCATCC<br>TGGTGATGTCAGCATTAGCC	11	114169287<br>114169352	LtE	LtE
Chr11_4	GCTGTGCCTTTCTGATGTGA<br>TGTTTGCTCTTGTCATTGTTCTC	11	113019972<br>113020037	LtE	LtE
Chr10 p1	AGGCCTGGAATATGGACAAG<br>TTGGTGACAAGTTTAGAGAAAAGC	10	11858093<br>11858188	NE	NE
Chr10_2	ACCATTTCTCCCAGGATTTGT<br>CAGTTGTTGAGAATGTAAAGAACAGC	10	4986572<br>4986659	NE	NE
Chr10_p3	CCCAGAGAGGCTGATTTCAC<br>CAGAGAGGCTTTGAGAGCACA	10	11560213<br>11560277	NE	NE
Chr10_p4	CTGAGGCCGACAGAGAAGTT<br>GGAGGAGAAAGCTGACAGGA	10	12288594<br>12288655	NE	NE
Chr10_p5	GCCCTCCTGTGATTTCCTG<br>ACAGCCACTGATCTGGCTTC	10	11349302<br>11349366	NE	NE
Chr10_p6	CGCAAGCACACAAGAGGAT<br>CCTTGTAACATGACTGACAAAGAAC	10	12617922<br>12617997	NE	NE

Chr11_1	CCGGGTCTCTTCTTGGTGTG<br>TCATTGGGTATGCCACAGGC	11	131425341<br>131425457	LtE	LtE
Chr11-2	AGAGCTTGGGGTCAGGGTTA<br>AGTGTTGGAGGGTTCACACA	11	131477478<br>131477564	LtE	LtE
Chr12	GGGCTTCATGAGCCATACCA<br>ACCAGTAGTTCCCAGGACTGT	12	130174229<br>130174353	LtE	LtE
Chr13	CCCTTTTTGTGGCTGACTGC<br>ACCAACAGACAATAACGCTGC	13	76574440<br>76574536	LtE	NE
ChrX	TCCTACAGGACAGCAGGAAGA<br>TCTCCAAATTCACATTCATCGCT	X	31525569<br>31525646	LtE	LtE
G75/76	CATTCCGCTTTCCTTTTACA<br>TTTTCCTCCCTCACTCCTCT	1	89461665<br>89461724	LtE	LtE
G307/308	GACACCAAGGGCTGTTTTTC<br>TTTAAGACACATGTTTTCTCTACCTCA	1	89459900<br>89459981	LtE	LtE
G77/78	TTGCTTTTCTGCCATACCTC<br>CCTAGTGAGCCTTGCCATTA	1	88679907<br>88680022	LtE	LtE
G14/15	GAGCCAAGTCTCTTTTCAAG<br>GGATTCACAAGAAGGCAAGC	10	83986703<br>83986788	NE	NE
Early 1b	CAAATGATGAATTTGGAGAGG<br>AATGCGTAATGTTTACCCAC	19	3244014<br>3244113	NE	NE
MidLate 1a	GGTTTCATGTTACTAAGTGTC<br>GGGCTTCATCAGTAATGGAG	19	9487563<br>9487645	NE	LtE

* To validate the results of the Repli-seq experiments, the replication timing of 37 genomic regions has been assessed by qPCR analysis (see Methods). The probes coordinates are indicated (Hg19 human genome sequence). The column **Repli-seq Result** indicates whether in the Repli-seq experiment the genomic region was an LtE or EtL, or was not affected (NE). The column **qPCR in replicate** refers to the qPCR validation performed in a separate independent experiment. In the Primers column, the upper sequence indicates the forward, the lower the reverse primer. In the Primers' genomic coordinates, the upper number indicates the forward, the lower the reverse primer. The 3/37 regions showing a result different from that of Repli-seq are highlighted in yellow.

Supplementary Table S5
Genomic coordinates of valleys in control siLuc and in.siPREP1 HeLa cells.

COORDINATES OF SILUC CONTROL CELLS VALLEYS

**Chr**	**Start**	**End**	**S50 Value**
chr1	1350000	1600000	0,144990415
chr1	3400000	3650000	0,209091995
chr1	4600000	4850000	0,625913376
chr1	6350000	6600000	0,148302931
chr1	8050000	8300000	0,151459945
chr1	8900000	9150000	0,140667583
chr1	9900000	10150000	0,134948026
chr1	10900000	11150000	0,19914301
chr1	11850000	12100000	0,157432665
chr1	12350000	12600000	0,188951896
chr1	13450000	13700000	0,591115136
chr1	16300000	16550000	0,118423372
chr1	17300000	17550000	0,154980759
chr1	19800000	20050000	0,201709834
chr1	20900000	21150000	0,181555778
chr1	21500000	21750000	0,177341214
chr1	22050000	22300000	0,14352708
chr1	23750000	24000000	0,121698682
chr1	24850000	25100000	0,160500768
chr1	26850000	27100000	0,13267542
chr1	27800000	28050000	0,137211915

COORDINATES OF SIPREP1 CELLS VALLEYS

**Chr**	**Start**	**End**	**S50 Value**
chr1	0	150000	0,390663231
chr1	1400000	1650000	0,134581211
chr1	2000000	2250000	0,162841804
chr1	3400000	3650000	0,217751314
chr1	4550000	4800000	0,582505337
chr1	6350000	6600000	0,151438917
chr1	7300000	7550000	0,186469123
chr1	8050000	8300000	0,169175151
chr1	8900000	9150000	0,140459082
chr1	9900000	10150000	0,145296622
chr1	10900000	11150000	0,213550833
chr1	11850000	12100000	0,167335502
chr1	12400000	12650000	0,198060041
chr1	13950000	14200000	0,642688252
chr1	16250000	16500000	0,121421999
chr1	17300000	17550000	0,149845014
chr1	19800000	20050000	0,223464411
chr1	20850000	21100000	0,190106715
chr1	21450000	21700000	0,183410051
chr1	22050000	22300000	0,149148643
chr1	23800000	24050000	0,123644416

chr1	28650000	28900000	0,128440753
chr1	32100000	32350000	0,12206486
chr1	33050000	33300000	0,130719298
chr1	36500000	36750000	0,142086529
chr1	38100000	38350000	0,216414049
chr1	39450000	39700000	0,215018812
chr1	40350000	40600000	0,183854873
chr1	41250000	41500000	0,141603774
chr1	43850000	44100000	0,141501756
chr1	44450000	44700000	0,137433456
chr1	45050000	45300000	0,139129765
chr1	45900000	46150000	0,142587566
chr1	46600000	46850000	0,144390144
chr1	47650000	47900000	0,242487506
chr1	51850000	52100000	0,157920062
chr1	53100000	53350000	0,163456177
chr1	54250000	54500000	0,183825827
chr1	55300000	55550000	0,166714632
chr1	56850000	57100000	0,413544929
chr1	59050000	59300000	0,330002598
chr1	61950000	62200000	0,209904981
chr1	62750000	63000000	0,290006412
chr1	64000000	64250000	0,262179458
chr1	65350000	65600000	0,212245856
chr1	66650000	66900000	0,285233365
chr1	67950000	68200000	0,221670032
chr1	70700000	70950000	0,309676617

chr1	24850000	25100000	0,176201845
chr1	26800000	27050000	0,132402138
chr1	27700000	27950000	0,148065887
chr1	28650000	28900000	0,132114808
chr1	30100000	30350000	0,634681562
chr1	32050000	32300000	0,125196327
chr1	33050000	33300000	0,132923627
chr1	36500000	36750000	0,14605959
chr1	38100000	38350000	0,228202627
chr1	39450000	39700000	0,216437836
chr1	40200000	40450000	0,206469042
chr1	41250000	41500000	0,147218347
chr1	43850000	44100000	0,1400825
chr1	44450000	44700000	0,138554263
chr1	45050000	45300000	0,147298929
chr1	45900000	46150000	0,14643424
chr1	46650000	46900000	0,143048722
chr1	47700000	47950000	0,24703642
chr1	51850000	52100000	0,19165096
chr1	52350000	52600000	0,218836607
chr1	53100000	53350000	0,163661448
chr1	54250000	54500000	0,19282415
chr1	55300000	55550000	0,165221342
chr1	56900000	57150000	0,39178143
chr1	59100000	59350000	0,324849687
chr1	60000000	60250000	0,490272329
chr1	61500000	61750000	0,264237232

chr1	72600000	72850000	0,630862668
chr1	76100000	76350000	0,520909838
chr1	78200000	78450000	0,220725574
chr1	80150000	80400000	0,853927996
chr1	82050000	82300000	0,788152129
chr1	85650000	85900000	0,178181199
chr1	87200000	87450000	0,299275491
chr1	88150000	88400000	0,267841118
chr1	89050000	89300000	0,327655479
chr1	90250000	90500000	0,189884047
chr1	92150000	92400000	0,18572348
chr1	94000000	94250000	0,143127707
chr1	95000000	95250000	0,143399145
chr1	97300000	97550000	0,476484653
chr1	98350000	98600000	0,293548116
chr1	100350000	100600000	0,285232945
chr1	101500000	101750000	0,409756582
chr1	103300000	103550000	0,746249353
chr1	104100000	104350000	0,764497103
chr1	105500000	105750000	0,846812939
chr1	109600000	109850000	0,134415591
chr1	112000000	112250000	0,282635031
chr1	113300000	113550000	0,190474023
chr1	114300000	114550000	0,279888446
chr1	114950000	115200000	0,186482969
chr1	116650000	116900000	0,342547315
chr1	117550000	117800000	0,322775689

chr1	62000000	62250000	0,230038157
chr1	62700000	62950000	0,276252692
chr1	64000000	64250000	0,287128902
chr1	65350000	65600000	0,209211861
chr1	66650000	66900000	0,251082536
chr1	67950000	68200000	0,260149223
chr1	70700000	70950000	0,26957856
chr1	72650000	72900000	0,648242145
chr1	76100000	76350000	0,488919704
chr1	78200000	78450000	0,222716163
chr1	81900000	82150000	0,762560291
chr1	85650000	85900000	0,211010411
chr1	87200000	87450000	0,299400023
chr1	88200000	88450000	0,23942585
chr1	89050000	89300000	0,30701323
chr1	90250000	90500000	0,186646885
chr1	92150000	92400000	0,189149703
chr1	93250000	93500000	0,210534501
chr1	94000000	94250000	0,152239998
chr1	94950000	95200000	0,157621202
chr1	97300000	97550000	0,470441696
chr1	98350000	98600000	0,313439168
chr1	100350000	100600000	0,283355863
chr1	101450000	101700000	0,409300754
chr1	103300000	103550000	0,725498577
chr1	104100000	104350000	0,753282016
chr1	105250000	105500000	0,81195326

chr1	120450000	120700000	0,295023319
chr1	121050000	121300000	0,484484718
chr1	142650000	142900000	0,742379919
chr1	143900000	144150000	0,371499354
chr1	145400000	145650000	0,160528334
chr1	147500000	147750000	0,291616999
chr1	148550000	148800000	0,469729703
chr1	150350000	150600000	0,127094981
chr1	151100000	151350000	0,118672772
chr1	153650000	153900000	0,128209789
chr1	155050000	155300000	0,126701035
chr1	155900000	156150000	0,117382826
chr1	157850000	158100000	0,419523006
chr1	160000000	160250000	0,301767362
chr1	161050000	161300000	0,206068978
chr1	162250000	162500000	0,218477346
chr1	164450000	164700000	0,432610573
chr1	165600000	165850000	0,320630821
chr1	167800000	168050000	0,201177137
chr1	169300000	169550000	0,398063009
chr1	172200000	172450000	0,304695821
chr1	173850000	174100000	0,203692869
chr1	175150000	175400000	0,470401185
chr1	177100000	177350000	0,662921162
chr1	178500000	178750000	0,272445887
chr1	179000000	179250000	0,232586845
chr1	179950000	180200000	0,16193867

chr1	109200000	109450000	0,151781354
chr1	109650000	109900000	0,143109136
chr1	112000000	112250000	0,290100378
chr1	113300000	113550000	0,204574336
chr1	114300000	114550000	0,276433151
chr1	114950000	115200000	0,179764109
chr1	116650000	116900000	0,310950255
chr1	117600000	117850000	0,30474023
chr1	120400000	120650000	0,299857366
chr1	121050000	121300000	0,488444799
chr1	142650000	142900000	0,718361777
chr1	143800000	144050000	0,418847101
chr1	145350000	145600000	0,178441658
chr1	145950000	146200000	0,350908461
chr1	147000000	147250000	0,32439605
chr1	147550000	147800000	0,292372814
chr1	148600000	148850000	0,458098748
chr1	150350000	150600000	0,133909121
chr1	151050000	151300000	0,123812344
chr1	153650000	153900000	0,129423825
chr1	155100000	155350000	0,126124066
chr1	155950000	156200000	0,124640038
chr1	157850000	158100000	0,406319023
chr1	160000000	160250000	0,287949525
chr1	161050000	161300000	0,216807136
chr1	162050000	162300000	0,211045425
chr1	164450000	164700000	0,390713766

chr1	180850000	181100000	0,19183224
chr1	183000000	183250000	0,160463875
chr1	184900000	185150000	0,351041329
chr1	185250000	185500000	0,352421408
chr1	186450000	186700000	0,244128558
chr1	189050000	189300000	0,817867792
chr1	189650000	189900000	0,828849595
chr1	190650000	190900000	0,821807305
chr1	192900000	193150000	0,350962193
chr1	197250000	197500000	0,285379263
chr1	198050000	198300000	0,319630214
chr1	200550000	200800000	0,196621995
chr1	201350000	201600000	0,155845068
chr1	201850000	202100000	0,135136403
chr1	202750000	203000000	0,212118434
chr1	203550000	203800000	0,160008712
chr1	204250000	204500000	0,158466301
chr1	205200000	205450000	0,161888161
chr1	206050000	206300000	0,434667505
chr1	207200000	207450000	0,131694629
chr1	209800000	210050000	0,422489207
chr1	210300000	210550000	0,406788535
chr1	211750000	212000000	0,149215127
chr1	212500000	212750000	0,183675943
chr1	214600000	214850000	0,209918534
chr1	215550000	215800000	0,662981538
chr1	218500000	218750000	0,341884805

chr1	165600000	165850000	0,303530355
chr1	167900000	168150000	0,218803931
chr1	169300000	169550000	0,395404175
chr1	171600000	171850000	0,353722008
chr1	172250000	172500000	0,302181418
chr1	173850000	174100000	0,21822577
chr1	175200000	175450000	0,450698287
chr1	177100000	177350000	0,670600553
chr1	179000000	179250000	0,2405339
chr1	179900000	180150000	0,176115802
chr1	180900000	181150000	0,210802163
chr1	182950000	183200000	0,186198851
chr1	184950000	185200000	0,345349741
chr1	186450000	186700000	0,299471901
chr1	189100000	189350000	0,798731182
chr1	190300000	190550000	0,807609061
chr1	191000000	191250000	0,796852071
chr1	192900000	193150000	0,350132029
chr1	197250000	197500000	0,275598121
chr1	197850000	198100000	0,316265525
chr1	200550000	200800000	0,221069387
chr1	201350000	201600000	0,165311466
chr1	201850000	202100000	0,145675909
chr1	202750000	203000000	0,221313241
chr1	203550000	203800000	0,171920846
chr1	204250000	204500000	0,167407009
chr1	205200000	205450000	0,169012255

chr1	220700000	220950000	0,294149581
chr1	222950000	223200000	0,211863092
chr1	224200000	224450000	0,185620072
chr1	225950000	226200000	0,134742246
chr1	226850000	227100000	0,291689997
chr1	227450000	227700000	0,300043247
chr1	228300000	228550000	0,208859376
chr1	229500000	229750000	0,24469651
chr1	231450000	231700000	0,285042855
chr1	233400000	233650000	0,324913425
chr1	234650000	234900000	0,160519387
chr1	235050000	235300000	0,158196979
chr1	237800000	238050000	0,454434202
chr1	240000000	240250000	0,735944208
chr1	241800000	242050000	0,228051463
chr1	243100000	243350000	0,637082783
chr1	244950000	245200000	0,228998526
chr1	246800000	247050000	0,18060074
chr10	850000	1100000	0,182205928
chr10	1900000	2150000	0,389754225
chr10	3600000	3850000	0,253524623
chr10	4850000	5100000	0,330231515
chr10	5800000	6050000	0,200914053
chr10	6650000	6900000	0,290908553
chr10	7250000	7500000	0,273561254
chr10	11800000	12050000	0,128040641
chr10	13100000	13350000	0,265355582

chr1	206050000	206300000	0,439409937
chr1	207250000	207500000	0,136427504
chr1	209750000	210000000	0,424152747
chr1	210300000	210550000	0,42879298
chr1	211700000	211950000	0,157540807
chr1	212550000	212800000	0,199536021
chr1	214600000	214850000	0,208308523
chr1	215500000	215750000	0,615366911
chr1	218500000	218750000	0,384884852
chr1	220650000	220900000	0,287252392
chr1	222950000	223200000	0,196313677
chr1	224250000	224500000	0,197313646
chr1	226000000	226250000	0,138944598
chr1	227400000	227650000	0,290140547
chr1	228250000	228500000	0,206692241
chr1	229550000	229800000	0,249735411
chr1	231450000	231700000	0,281877623
chr1	233400000	233650000	0,304783091
chr1	234650000	234900000	0,146836798
chr1	237800000	238050000	0,484925034
chr1	239950000	240200000	0,716326812
chr1	241800000	242050000	0,223297347
chr1	243150000	243400000	0,595864806
chr1	244950000	245200000	0,219041178
chr1	246800000	247050000	0,18205999
chr1	248800000	249050000	0,549808996
chr1	249100000	249250621	0,547087211

chr10	14650000	14900000	0,226266077	chr10	850000	1100000	0,182133363
chr10	17700000	17950000	0,268557775	chr10	1900000	2150000	0,362962557
chr10	18250000	18500000	0,261606357	chr10	3650000	3900000	0,250258425
chr10	21550000	21800000	0,180487204	chr10	4900000	5150000	0,270073599
chr10	22450000	22700000	0,306151767	chr10	5750000	6000000	0,21457769
chr10	25000000	25250000	0,271627822	chr10	6700000	6950000	0,277625203
chr10	27200000	27450000	0,28645351	chr10	7250000	7500000	0,249058601
chr10	28650000	28900000	0,211407343	chr10	11800000	12050000	0,132160311
chr10	29250000	29500000	0,235225774	chr10	13100000	13350000	0,27437154
chr10	29950000	30200000	0,249756771	chr10	14650000	14900000	0,231245392
chr10	30600000	30850000	0,277793318	chr10	17750000	18000000	0,257259772
chr10	32250000	32500000	0,188386629	chr10	21550000	21800000	0,180479406
chr10	33150000	33400000	0,184873684	chr10	22450000	22700000	0,296830267
chr10	34050000	34300000	0,430605282	chr10	25000000	25250000	0,234879758
chr10	35300000	35550000	0,248004096	chr10	27250000	27500000	0,280888792
chr10	37000000	37250000	0,749511694	chr10	28650000	28900000	0,223301165
chr10	38200000	38450000	0,630573348	chr10	29300000	29550000	0,253679861
chr10	43750000	44000000	0,278153982	chr10	29950000	30200000	0,235538955
chr10	46000000	46250000	0,394262765	chr10	30600000	30850000	0,272127891
chr10	46950000	47200000	0,224479136	chr10	32250000	32500000	0,193608534
chr10	48300000	48550000	0,296266551	chr10	33150000	33400000	0,180864924
chr10	49250000	49500000	0,300988632	chr10	34200000	34450000	0,431787881
chr10	50650000	50900000	0,267719755	chr10	35300000	35550000	0,243749535
chr10	51550000	51800000	0,307667002	chr10	37000000	37250000	0,744597033
chr10	54100000	54350000	0,25002865	chr10	38150000	38400000	0,59071645
chr10	58000000	58250000	0,785255337	chr10	43750000	44000000	0,261568667
chr10	60100000	60350000	0,224761158	chr10	46050000	46300000	0,383084522

chr10	61500000	61750000	0,362264201
chr10	62550000	62800000	0,326303604
chr10	63500000	63750000	0,182505441
chr10	65300000	65550000	0,309293707
chr10	66950000	67200000	0,825178812
chr10	68400000	68650000	0,576234984
chr10	69900000	70150000	0,147868983
chr10	70700000	70950000	0,142417698
chr10	71950000	72200000	0,168258748
chr10	73850000	74100000	0,120625046
chr10	75450000	75700000	0,140293929
chr10	76750000	77000000	0,22811724
chr10	78200000	78450000	0,653836526
chr10	79600000	79850000	0,245622639
chr10	81000000	81250000	0,307971917
chr10	81850000	82100000	0,356924599
chr10	85350000	85600000	0,416483814
chr10	87200000	87450000	0,494001926
chr10	88400000	88650000	0,174073877
chr10	89400000	89650000	0,164361352
chr10	90950000	91200000	0,155992234
chr10	92650000	92900000	0,270857514
chr10	93350000	93600000	0,238129561
chr10	94250000	94500000	0,198278589
chr10	95100000	95350000	0,184859789
chr10	96000000	96250000	0,219312258
chr10	96950000	97200000	0,304207169

chr10	46800000	47050000	0,20055157
chr10	47550000	47800000	0,33300936
chr10	48500000	48750000	0,304665377
chr10	49300000	49550000	0,300549567
chr10	50700000	50950000	0,253767744
chr10	51800000	52050000	0,357308276
chr10	54100000	54350000	0,2437651
chr10	56800000	57050000	0,80251569
chr10	58000000	58250000	0,771341991
chr10	60050000	60300000	0,235036005
chr10	61550000	61800000	0,350465895
chr10	62550000	62800000	0,317317957
chr10	63450000	63700000	0,196816849
chr10	65300000	65550000	0,299125837
chr10	68400000	68650000	0,583865216
chr10	69800000	70050000	0,146124189
chr10	70750000	71000000	0,14782478
chr10	71900000	72150000	0,170331175
chr10	73800000	74050000	0,125957801
chr10	75500000	75750000	0,145089617
chr10	76750000	77000000	0,237291261
chr10	78200000	78450000	0,65378393
chr10	79600000	79850000	0,250426747
chr10	81000000	81250000	0,294058044
chr10	81850000	82100000	0,344463303
chr10	85350000	85600000	0,374891083
chr10	87250000	87500000	0,469842852

chr10	99150000	99400000	0,137157683
chr10	101550000	101800000	0,172687917
chr10	102650000	102900000	0,196337341
chr10	104000000	104250000	0,129618011
chr10	104850000	105100000	0,135192176
chr10	105600000	105850000	0,222058096
chr10	108550000	108800000	0,836752632
chr10	109800000	110050000	0,802797394
chr10	112200000	112450000	0,148585807
chr10	114550000	114800000	0,2670293
chr10	116200000	116450000	0,22230221
chr10	117600000	117850000	0,697967694
chr10	118600000	118850000	0,380887347
chr10	121100000	121350000	0,186284419
chr10	123800000	124050000	0,190378163
chr10	126300000	126550000	0,224900706
chr10	127250000	127500000	0,283606477
chr10	128300000	128550000	0,664558787
chr10	129750000	130000000	0,460882334
chr10	132300000	132550000	0,375792223
chr10	134100000	134350000	0,168181518
chr10	135000000	135250000	0,294498274
chr11	500000	750000	0,133430963
chr11	2850000	3100000	0,220804454
chr11	3700000	3950000	0,225297125
chr11	5550000	5800000	0,591647975
chr11	6400000	6650000	0,286785377

chr10	88350000	88600000	0,186218036
chr10	89400000	89650000	0,178458094
chr10	91000000	91250000	0,184861696
chr10	92650000	92900000	0,269546082
chr10	93300000	93550000	0,239734542
chr10	94250000	94500000	0,204647904
chr10	95100000	95350000	0,190756311
chr10	96000000	96250000	0,229875214
chr10	96950000	97200000	0,290881521
chr10	99150000	99400000	0,136425382
chr10	101550000	101800000	0,180408881
chr10	102650000	102900000	0,202959398
chr10	103950000	104200000	0,137285412
chr10	104850000	105100000	0,147543243
chr10	105650000	105900000	0,230913332
chr10	107400000	107650000	0,797021509
chr10	108450000	108700000	0,802247396
chr10	108950000	109200000	0,801934586
chr10	109800000	110050000	0,787215846
chr10	112300000	112550000	0,153262026
chr10	114550000	114800000	0,268080585
chr10	116150000	116400000	0,253907499
chr10	118600000	118850000	0,358083625
chr10	120900000	121150000	0,193839558
chr10	121150000	121400000	0,192993121
chr10	123800000	124050000	0,179172395
chr10	126350000	126600000	0,224461884

chr11	9350000	9600000	0,13498419
chr11	10300000	10550000	0,239331066
chr11	10750000	11000000	0,196265584
chr11	12400000	12650000	0,172373873
chr11	14300000	14550000	0,272368141
chr11	15900000	16150000	0,490495103
chr11	17050000	17300000	0,202285785
chr11	18250000	18500000	0,172166494
chr11	19550000	19800000	0,2658987
chr11	22750000	23000000	0,794386641
chr11	23850000	24100000	0,823103966
chr11	24650000	24900000	0,797051498
chr11	27750000	28000000	0,268232567
chr11	28600000	28850000	0,309788261
chr11	30200000	30450000	0,701723965
chr11	31850000	32100000	0,439594405
chr11	32900000	33150000	0,196165049
chr11	34400000	34650000	0,224683557
chr11	35000000	35250000	0,219554848
chr11	36150000	36400000	0,291304257
chr11	39100000	39350000	0,743029938
chr11	40000000	40250000	0,825324014
chr11	41050000	41300000	0,82982546
chr11	41300000	41550000	0,830091671
chr11	43400000	43650000	0,488024441
chr11	44450000	44700000	0,345892264
chr11	46700000	46950000	0,146575545

chr10	127250000	127500000	0,272947491
chr10	129750000	130000000	0,432191586
chr10	132350000	132600000	0,359499427
chr10	134100000	134350000	0,168126475
chr10	135000000	135250000	0,278588971
chr11	500000	750000	0,129354995
chr11	2850000	3100000	0,210166872
chr11	3700000	3950000	0,227362736
chr11	5550000	5800000	0,582419877
chr11	6400000	6650000	0,287322589
chr11	9350000	9600000	0,142111537
chr11	10250000	10500000	0,245225216
chr11	10750000	11000000	0,205049524
chr11	11950000	12200000	0,19535042
chr11	12400000	12650000	0,197432476
chr11	12900000	13150000	0,239234653
chr11	14300000	14550000	0,304492116
chr11	15900000	16150000	0,490728166
chr11	17050000	17300000	0,220699075
chr11	18250000	18500000	0,180714732
chr11	19550000	19800000	0,279863383
chr11	22000000	22250000	0,804048054
chr11	22750000	23000000	0,789136555
chr11	23600000	23850000	0,802648338
chr11	24650000	24900000	0,764671752
chr11	25500000	25750000	0,81045634
chr11	27700000	27950000	0,274489204

chr11	47250000	47500000	0,129361676
chr11	47600000	47850000	0,128710068
chr11	50300000	50550000	0,764117103
chr11	51200000	51450000	0,748362178
chr11	55100000	55350000	0,752861309
chr11	57150000	57400000	0,175689584
chr11	58200000	58450000	0,548045333
chr11	59250000	59500000	0,30425628
chr11	61600000	61850000	0,144118369
chr11	62250000	62500000	0,126964611
chr11	63600000	63850000	0,136074553
chr11	65050000	65300000	0,118493335
chr11	66750000	67000000	0,121370644
chr11	67900000	68150000	0,136308173
chr11	70000000	70250000	0,218690548
chr11	71750000	72000000	0,178102449
chr11	72900000	73150000	0,139950258
chr11	73300000	73550000	0,143626974
chr11	74200000	74450000	0,225962288
chr11	75050000	75300000	0,172865366
chr11	76350000	76600000	0,192258257
chr11	77650000	77900000	0,198119917
chr11	80200000	80450000	0,539773425
chr11	82650000	82900000	0,253517518
chr11	85800000	86050000	0,174352715
chr11	86250000	86500000	0,178621705
chr11	87950000	88200000	0,530657188

chr11	28600000	28850000	0,308238403
chr11	30150000	30400000	0,705770758
chr11	31800000	32050000	0,419482644
chr11	32900000	33150000	0,20169707
chr11	34400000	34650000	0,21388146
chr11	34950000	35200000	0,237754486
chr11	35750000	36000000	0,296544055
chr11	36050000	36300000	0,298010154
chr11	39100000	39350000	0,726097278
chr11	40850000	41100000	0,80460981
chr11	41750000	42000000	0,788951424
chr11	43350000	43600000	0,491314082
chr11	44450000	44700000	0,327949348
chr11	46750000	47000000	0,15175612
chr11	47250000	47500000	0,13576481
chr11	47650000	47900000	0,134225384
chr11	50200000	50450000	0,730392445
chr11	51200000	51450000	0,717091794
chr11	54850000	55100000	0,725073275
chr11	57150000	57400000	0,177365997
chr11	58200000	58450000	0,536933086
chr11	59250000	59500000	0,30812444
chr11	61600000	61850000	0,14972087
chr11	62300000	62550000	0,132010249
chr11	63650000	63900000	0,134904624
chr11	65050000	65300000	0,123902626
chr11	66700000	66950000	0,124566145

chr11	89850000	90100000	0,663920414
chr11	90750000	91000000	0,546131436
chr11	91850000	92100000	0,71500923
chr11	93350000	93600000	0,36323071
chr11	94350000	94600000	0,3116451
chr11	95650000	95900000	0,286934822
chr11	100400000	100650000	0,402394523
chr11	102100000	102350000	0,179688167
chr11	104900000	105150000	0,488579848
chr11	105750000	106000000	0,617512152
chr11	107750000	108000000	0,314517124
chr11	108250000	108500000	0,326885649
chr11	110050000	110300000	0,176345229
chr11	111750000	112000000	0,161783977
chr11	113000000	113250000	0,377783424
chr11	113550000	113800000	0,376601294
chr11	114050000	114300000	0,352168624
chr11	115200000	115450000	0,724853161
chr11	116850000	117100000	0,179121851
chr11	118550000	118800000	0,120602717
chr11	120050000	120300000	0,242167357
chr11	121200000	121450000	0,374626032
chr11	122350000	122600000	0,174039129
chr11	123000000	123250000	0,1870978
chr11	124600000	124850000	0,33136014
chr11	125900000	126150000	0,280530541
chr11	129050000	129300000	0,238848677

chr11	67900000	68150000	0,140802761
chr11	70000000	70250000	0,230139626
chr11	71750000	72000000	0,189531293
chr11	72900000	73150000	0,144766761
chr11	73400000	73650000	0,152305595
chr11	74200000	74450000	0,231967979
chr11	75050000	75300000	0,18260258
chr11	76350000	76600000	0,185092452
chr11	77650000	77900000	0,20272873
chr11	80200000	80450000	0,536243008
chr11	82700000	82950000	0,259132109
chr11	85850000	86100000	0,185721696
chr11	86250000	86500000	0,174760498
chr11	87950000	88200000	0,505652113
chr11	89900000	90150000	0,637227947
chr11	90700000	90950000	0,57699909
chr11	91750000	92000000	0,710503462
chr11	93350000	93600000	0,346647238
chr11	94350000	94600000	0,3340388
chr11	95700000	95950000	0,288324686
chr11	100400000	100650000	0,37988549
chr11	102100000	102350000	0,196134099
chr11	104900000	105150000	0,484993028
chr11	105750000	106000000	0,602351404
chr11	107750000	108000000	0,305746033
chr11	108200000	108450000	0,3073176
chr11	110050000	110300000	0,167216177

chr11	129900000	130150000	0,182195538
chr11	134000000	134250000	0,526409623
chr12	600000	850000	0,174346024
chr12	1550000	1800000	0,229223515
chr12	2950000	3200000	0,23941508
chr12	4250000	4500000	0,430371016
chr12	6600000	6850000	0,122986324
chr12	8050000	8300000	0,219965963
chr12	9100000	9350000	0,167483899
chr12	10300000	10550000	0,216299541
chr12	12800000	13050000	0,116153556
chr12	14900000	15150000	0,335451594
chr12	15750000	16000000	0,331501102
chr12	19900000	20150000	0,150744502
chr12	21500000	21750000	0,472439633
chr12	22600000	22850000	0,444013266
chr12	25350000	25600000	0,36653292
chr12	26150000	26400000	0,321312279
chr12	27000000	27250000	0,21986412
chr12	29800000	30050000	0,427679406
chr12	31550000	31800000	0,227899886
chr12	32600000	32850000	0,251860781
chr12	34150000	34400000	0,66780896
chr12	38800000	39050000	0,378199615
chr12	39650000	39900000	0,420436377
chr12	40400000	40650000	0,556894049
chr12	42600000	42850000	0,361066038

chr11	111750000	112000000	0,160724216
chr11	113000000	113250000	0,323114313
chr11	113550000	113800000	0,341943344
chr11	114050000	114300000	0,313123107
chr11	115150000	115400000	0,71678705
chr11	116900000	117150000	0,186618916
chr11	118500000	118750000	0,123254443
chr11	120000000	120250000	0,235968061
chr11	121150000	121400000	0,356217382
chr11	122300000	122550000	0,175004421
chr11	123000000	123250000	0,196644102
chr11	124600000	124850000	0,321689902
chr11	125900000	126150000	0,264703823
chr11	129050000	129300000	0,251513559
chr11	129850000	130100000	0,191517449
chr11	134050000	134300000	0,506115389
chr12	600000	850000	0,181147762
chr12	1600000	1850000	0,230266676
chr12	2950000	3200000	0,243153555
chr12	4100000	4350000	0,412046245
chr12	6600000	6850000	0,125670946
chr12	8000000	8250000	0,211160624
chr12	9150000	9400000	0,175757001
chr12	10250000	10500000	0,212257757
chr12	12800000	13050000	0,122375806
chr12	14900000	15150000	0,364276918
chr12	15800000	16050000	0,31762599

chr12	44050000	44300000	0,38229168
chr12	46700000	46950000	0,193079557
chr12	48150000	48400000	0,160906432
chr12	49500000	49750000	0,12991113
chr12	50500000	50750000	0,128709231
chr12	51350000	51600000	0,157522947
chr12	52450000	52700000	0,148850853
chr12	53500000	53750000	0,115705379
chr12	54450000	54700000	0,146852636
chr12	56550000	56800000	0,121027995
chr12	57550000	57800000	0,14723878
chr12	59200000	59450000	0,457594388
chr12	60200000	60450000	0,455055138
chr12	62900000	63150000	0,280957952
chr12	64850000	65100000	0,238819888
chr12	65800000	66050000	0,306218742
chr12	67600000	67850000	0,656865185
chr12	69250000	69500000	0,170668976
chr12	70950000	71200000	0,351713889
chr12	71950000	72200000	0,28931277
chr12	74750000	75000000	0,425384121
chr12	76200000	76450000	0,229750449
chr12	76900000	77150000	0,289020765
chr12	79000000	79250000	0,405583
chr12	80000000	80250000	0,2356392
chr12	82650000	82900000	0,717615578
chr12	84250000	84500000	0,807779465

chr12	19900000	20150000	0,149874471
chr12	21500000	21750000	0,460501035
chr12	22600000	22850000	0,412507763
chr12	25350000	25600000	0,360728869
chr12	26150000	26400000	0,320733801
chr12	27050000	27300000	0,243405849
chr12	29800000	30050000	0,425950714
chr12	31600000	31850000	0,228890045
chr12	32600000	32850000	0,260323461
chr12	34200000	34450000	0,628501217
chr12	38750000	39000000	0,400362967
chr12	39750000	40000000	0,417652114
chr12	40400000	40650000	0,539422636
chr12	42600000	42850000	0,34306607
chr12	44100000	44350000	0,358778462
chr12	46700000	46950000	0,195821373
chr12	48150000	48400000	0,159254488
chr12	49550000	49800000	0,136238351
chr12	50500000	50750000	0,135079653
chr12	51350000	51600000	0,156741413
chr12	52450000	52700000	0,151134734
chr12	53500000	53750000	0,121152286
chr12	54500000	54750000	0,153013744
chr12	56550000	56800000	0,126325044
chr12	57500000	57750000	0,153053141
chr12	59250000	59500000	0,468062333
chr12	60200000	60450000	0,497640534

chr12	85700000	85950000	0,551804539
chr12	86050000	86300000	0,546355359
chr12	87050000	87300000	0,551484335
chr12	88600000	88850000	0,26845917
chr12	90050000	90300000	0,305860302
chr12	91450000	91700000	0,380318589
chr12	92300000	92550000	0,362467831
chr12	93900000	94150000	0,206008579
chr12	95950000	96200000	0,150373127
chr12	98800000	99050000	0,205964528
chr12	100550000	100800000	0,336997207
chr12	102100000	102350000	0,273698774
chr12	104500000	104750000	0,15206231
chr12	105300000	105550000	0,207307063
chr12	106400000	106650000	0,294549274
chr12	107150000	107400000	0,317347996
chr12	107950000	108200000	0,521596349
chr12	109050000	109300000	0,206705632
chr12	110600000	110850000	0,125867802
chr12	112150000	112400000	0,143063305
chr12	113550000	113800000	0,22483154
chr12	115050000	115300000	0,572908532
chr12	117250000	117500000	0,206978048
chr12	118350000	118600000	0,295879929
chr12	120650000	120900000	0,143420065
chr12	122100000	122350000	0,132688032
chr12	123050000	123300000	0,137476258

chr12	62950000	63200000	0,2515212
chr12	64900000	65150000	0,255097254
chr12	65650000	65900000	0,327511494
chr12	67650000	67900000	0,620175199
chr12	69250000	69500000	0,16763722
chr12	70900000	71150000	0,338594071
chr12	71950000	72200000	0,292222734
chr12	74750000	75000000	0,438909432
chr12	76250000	76500000	0,241753431
chr12	76850000	77100000	0,304261506
chr12	79050000	79300000	0,417499778
chr12	80000000	80250000	0,245522127
chr12	82700000	82950000	0,690243452
chr12	84350000	84600000	0,793915803
chr12	85600000	85850000	0,543925573
chr12	87050000	87300000	0,561556278
chr12	88600000	88850000	0,309770239
chr12	90000000	90250000	0,30385146
chr12	91500000	91750000	0,379891791
chr12	92300000	92550000	0,35715069
chr12	93800000	94050000	0,245890242
chr12	95950000	96200000	0,165802259
chr12	96650000	96900000	0,236990606
chr12	98800000	99050000	0,212223996
chr12	100550000	100800000	0,343714916
chr12	102100000	102350000	0,285580802
chr12	104500000	104750000	0,158017103

chr12	123700000	123950000	0,139265696
chr12	125150000	125400000	0,139488194
chr12	127400000	127650000	0,778704565
chr12	129150000	129400000	0,7486723
chr12	130500000	130750000	0,375477776
chr12	131350000	131600000	0,382471809
chr12	132250000	132500000	0,221967126
chr12	133000000	133250000	0,312919157
chr13	20400000	20650000	0,232214575
chr13	21450000	21700000	0,169196465
chr13	24050000	24300000	0,311839803
chr13	27550000	27800000	0,177442948
chr13	28550000	28800000	0,353280428
chr13	30800000	31050000	0,194349065
chr13	32900000	33150000	0,275597399
chr13	34050000	34300000	0,229680904
chr13	36700000	36950000	0,441785362
chr13	37400000	37650000	0,385387689
chr13	38100000	38350000	0,437719861
chr13	41600000	41850000	0,237846397
chr13	42500000	42750000	0,252526142
chr13	43550000	43800000	0,283385494
chr13	44700000	44950000	0,195780774
chr13	45600000	45850000	0,186248209
chr13	47100000	47350000	0,37079928
chr13	49250000	49500000	0,220644048
chr13	49900000	50150000	0,198837071

chr12	105350000	105600000	0,231993419
chr12	106450000	106700000	0,296212803
chr12	107150000	107400000	0,299630048
chr12	107900000	108150000	0,513267145
chr12	109050000	109300000	0,206157573
chr12	110550000	110800000	0,128401492
chr12	112450000	112700000	0,15206513
chr12	113550000	113800000	0,235452238
chr12	115050000	115300000	0,544333776
chr12	117300000	117550000	0,221774267
chr12	118350000	118600000	0,292712678
chr12	120650000	120900000	0,152574727
chr12	122150000	122400000	0,132716889
chr12	122900000	123150000	0,140650256
chr12	123150000	123400000	0,140201528
chr12	123700000	123950000	0,142560625
chr12	125150000	125400000	0,140280159
chr12	127400000	127650000	0,782984885
chr12	129200000	129450000	0,713794511
chr12	130500000	130750000	0,33476232
chr12	131450000	131700000	0,349910624
chr12	132250000	132500000	0,209448037
chr12	133000000	133250000	0,297213756
chr13	20350000	20600000	0,233009119
chr13	21500000	21750000	0,180396194
chr13	24050000	24300000	0,329464857
chr13	24650000	24900000	0,329934674

chr13	52050000	52300000	0,302415878
chr13	52750000	53000000	0,31824852
chr13	55000000	55250000	0,785594411
chr13	55900000	56150000	0,62900926
chr13	57550000	57800000	0,825606239
chr13	60700000	60950000	0,392078294
chr13	64750000	65000000	0,592162947
chr13	66800000	67050000	0,591274455
chr13	67600000	67850000	0,500917981
chr13	71150000	71400000	0,818882682
chr13	73650000	73900000	0,156606591
chr13	76000000	76250000	0,287951123
chr13	77600000	77850000	0,386315374
chr13	79950000	80200000	0,209362861
chr13	83150000	83400000	0,609464246
chr13	83650000	83900000	0,576156137
chr13	84950000	85200000	0,622106998
chr13	85950000	86200000	0,515331695
chr13	87700000	87950000	0,611707651
chr13	88300000	88550000	0,629381631
chr13	89200000	89450000	0,681850511
chr13	90100000	90350000	0,632564832
chr13	91050000	91300000	0,596692296
chr13	93450000	93700000	0,796805058
chr13	95150000	95400000	0,517088974
chr13	96050000	96300000	0,338283017
chr13	97750000	98000000	0,319636101

chr13	24950000	25200000	0,32852168
chr13	27550000	27800000	0,170068567
chr13	28550000	28800000	0,324309701
chr13	30800000	31050000	0,195299902
chr13	32900000	33150000	0,289644677
chr13	34050000	34300000	0,232026647
chr13	36850000	37100000	0,43950215
chr13	37400000	37650000	0,36686914
chr13	38100000	38350000	0,474983261
chr13	40650000	40900000	0,291949242
chr13	41600000	41850000	0,239737369
chr13	42450000	42700000	0,263677183
chr13	43600000	43850000	0,278025922
chr13	44700000	44950000	0,177743791
chr13	45650000	45900000	0,204796644
chr13	47150000	47400000	0,359143189
chr13	49200000	49450000	0,218244744
chr13	49900000	50150000	0,198235749
chr13	52050000	52300000	0,288351363
chr13	52750000	53000000	0,303294301
chr13	55000000	55250000	0,779232928
chr13	55950000	56200000	0,63669811
chr13	57500000	57750000	0,813454046
chr13	60700000	60950000	0,378490851
chr13	64800000	65050000	0,603332186
chr13	66750000	67000000	0,57650134
chr13	67600000	67850000	0,480999492

chr13	98600000	98850000	0,26415578
chr13	99200000	99450000	0,270306453
chr13	99750000	100000000	0,274972329
chr13	100500000	100750000	0,293843599
chr13	102000000	102250000	0,333106369
chr13	103250000	103500000	0,423258233
chr13	106550000	106800000	0,42865046
chr13	109700000	109950000	0,250925369
chr13	111250000	111500000	0,222654267
chr13	113300000	113550000	0,198598764
chr13	113700000	113950000	0,197606323
chr13	114800000	115050000	0,364972604
chr14	19700000	19950000	0,587479754
chr14	20800000	21050000	0,299548638
chr14	21600000	21850000	0,194290995
chr14	23300000	23550000	0,139940258
chr14	24550000	24800000	0,183213884
chr14	27200000	27450000	0,678316958
chr14	28800000	29050000	0,512520988
chr14	31550000	31800000	0,165080778
chr14	32700000	32950000	0,274991803
chr14	35100000	35350000	0,13820781
chr14	35600000	35850000	0,135898588
chr14	37550000	37800000	0,336307432
chr14	38000000	38250000	0,34275623
chr14	38450000	38700000	0,341879882
chr14	39550000	39800000	0,211436355

chr13	73650000	73900000	0,163536321
chr13	76050000	76300000	0,249643023
chr13	77600000	77850000	0,385178386
chr13	79950000	80200000	0,210502348
chr13	83150000	83400000	0,582119403
chr13	83650000	83900000	0,556637193
chr13	84850000	85100000	0,617837905
chr13	85950000	86200000	0,559619047
chr13	87650000	87900000	0,586110498
chr13	88200000	88450000	0,608813611
chr13	89150000	89400000	0,663878571
chr13	90150000	90400000	0,640666817
chr13	91000000	91250000	0,598685872
chr13	93400000	93650000	0,775351667
chr13	95150000	95400000	0,514755375
chr13	96050000	96300000	0,31746563
chr13	97750000	98000000	0,342794802
chr13	98650000	98900000	0,278300385
chr13	99200000	99450000	0,278377634
chr13	99750000	100000000	0,273123467
chr13	100450000	100700000	0,291841455
chr13	102000000	102250000	0,344655122
chr13	103250000	103500000	0,4153506
chr13	106500000	106750000	0,419749273
chr13	109700000	109950000	0,2608093
chr13	111200000	111450000	0,221241666
chr13	113300000	113550000	0,194815783

chr14	41250000	41500000	0,804537553
chr14	41800000	42050000	0,812780763
chr14	42500000	42750000	0,807664063
chr14	45400000	45650000	0,365038012
chr14	47450000	47700000	0,846115305
chr14	48400000	48650000	0,837938852
chr14	50300000	50550000	0,144205215
chr14	50900000	51150000	0,148103242
chr14	52600000	52850000	0,236877854
chr14	53100000	53350000	0,198369816
chr14	55450000	55700000	0,143684965
chr14	56800000	57050000	0,651288424
chr14	57700000	57950000	0,586285796
chr14	58700000	58950000	0,293262493
chr14	59500000	59750000	0,270417331
chr14	60550000	60800000	0,357171954
chr14	61050000	61300000	0,319419457
chr14	61900000	62150000	0,217059165
chr14	63050000	63300000	0,401826973
chr14	64100000	64350000	0,153072592
chr14	64950000	65200000	0,128116308
chr14	67650000	67900000	0,19218897
chr14	68450000	68700000	0,19471448
chr14	69350000	69600000	0,165203121
chr14	71050000	71300000	0,266545653
chr14	74100000	74350000	0,12798623
chr14	75500000	75750000	0,136453316

chr13	114750000	115000000	0,359332815
chr14	19700000	19950000	0,601213903
chr14	20800000	21050000	0,293722975
chr14	21600000	21850000	0,196418185
chr14	23350000	23600000	0,148352472
chr14	24550000	24800000	0,188155464
chr14	27200000	27450000	0,682910398
chr14	28800000	29050000	0,495328315
chr14	31550000	31800000	0,17418291
chr14	32700000	32950000	0,279912487
chr14	35050000	35300000	0,148006155
chr14	35600000	35850000	0,139126792
chr14	36250000	36500000	0,195646361
chr14	37550000	37800000	0,32830514
chr14	38000000	38250000	0,337526567
chr14	38400000	38650000	0,338347153
chr14	39550000	39800000	0,211432015
chr14	41250000	41500000	0,79962353
chr14	41900000	42150000	0,796806616
chr14	43100000	43350000	0,833100881
chr14	45400000	45650000	0,35099521
chr14	47700000	47950000	0,831541535
chr14	50300000	50550000	0,152673459
chr14	50900000	51150000	0,156852639
chr14	52550000	52800000	0,261447842
chr14	53100000	53350000	0,203981327
chr14	55450000	55700000	0,151536076

chr14	77400000	77650000	0,179725747
chr14	77850000	78100000	0,208015818
chr14	79550000	79800000	0,294147819
chr14	80600000	80850000	0,316915145
chr14	81650000	81900000	0,353136036
chr14	84200000	84450000	0,572184504
chr14	85650000	85900000	0,578355728
chr14	89050000	89300000	0,338955129
chr14	90100000	90350000	0,29725817
chr14	90750000	91000000	0,287429177
chr14	91600000	91850000	0,281814923
chr14	92300000	92550000	0,284239888
chr14	93450000	93700000	0,288902302
chr14	94350000	94600000	0,442435312
chr14	95750000	96000000	0,406359904
chr14	96800000	97050000	0,350138464
chr14	100600000	100850000	0,142866815
chr14	102600000	102850000	0,128622018
chr14	103600000	103850000	0,119946178
chr14	105350000	105600000	0,134969191
chr14	107000000	107250000	0,625171553
chr15	20700000	20950000	0,567825653
chr15	21950000	22200000	0,742472805
chr15	22950000	23200000	0,250218877
chr15	25400000	25650000	0,685666754
chr15	28500000	28750000	0,421288391
chr15	30050000	30300000	0,312733431

chr14	57700000	57950000	0,5818568
chr14	58700000	58950000	0,301190369
chr14	59500000	59750000	0,294669909
chr14	60550000	60800000	0,338146365
chr14	61050000	61300000	0,316001492
chr14	61900000	62150000	0,220412388
chr14	63100000	63350000	0,390592334
chr14	64100000	64350000	0,157074949
chr14	64950000	65200000	0,131256631
chr14	67600000	67850000	0,202386939
chr14	68500000	68750000	0,210993176
chr14	69350000	69600000	0,186669648
chr14	70150000	70400000	0,221280704
chr14	71050000	71300000	0,276169812
chr14	74100000	74350000	0,1346648
chr14	75500000	75750000	0,138395612
chr14	77400000	77650000	0,179833771
chr14	79550000	79800000	0,290119981
chr14	80550000	80800000	0,309485139
chr14	81650000	81900000	0,321991235
chr14	84150000	84400000	0,604311736
chr14	85650000	85900000	0,60727523
chr14	89150000	89400000	0,322451339
chr14	90150000	90400000	0,291834329
chr14	90750000	91000000	0,265371773
chr14	91650000	91900000	0,269890061
chr14	92250000	92500000	0,269372379

chr15	31150000	31400000	0,312725134
chr15	32700000	32950000	0,358108146
chr15	34500000	34750000	0,184923232
chr15	36900000	37150000	0,4128335
chr15	39200000	39450000	0,281738877
chr15	40950000	41200000	0,122277887
chr15	41650000	41900000	0,133914429
chr15	42600000	42850000	0,185439947
chr15	43250000	43500000	0,187800293
chr15	43800000	44050000	0,156683505
chr15	44450000	44700000	0,221814764
chr15	45550000	45800000	0,26001836
chr15	48000000	48250000	0,37195428
chr15	48950000	49200000	0,221832229
chr15	50600000	50850000	0,154158026
chr15	52150000	52400000	0,147275033
chr15	55950000	56200000	0,219133039
chr15	57000000	57250000	0,338394999
chr15	57650000	57900000	0,30700694
chr15	59050000	59300000	0,171338408
chr15	59650000	59900000	0,159254611
chr15	60550000	60800000	0,19421373
chr15	63500000	63750000	0,16659245
chr15	64250000	64500000	0,197575163
chr15	65250000	65500000	0,141070967
chr15	67050000	67300000	0,125897813
chr15	68100000	68350000	0,181677721

chr14	93450000	93700000	0,281530599
chr14	94350000	94600000	0,435804419
chr14	95750000	96000000	0,394867998
chr14	96800000	97050000	0,335375419
chr14	100650000	100900000	0,143910111
chr14	102600000	102850000	0,129883883
chr14	103600000	103850000	0,125109419
chr14	105350000	105600000	0,128364374
chr14	106950000	107200000	0,614297734
chr15	20700000	20950000	0,589392208
chr15	22000000	22250000	0,735505834
chr15	22950000	23200000	0,249909641
chr15	25350000	25600000	0,642787284
chr15	28500000	28750000	0,411286536
chr15	30100000	30350000	0,318236784
chr15	31350000	31600000	0,30184193
chr15	32700000	32950000	0,342161326
chr15	34500000	34750000	0,202136137
chr15	36950000	37200000	0,434878484
chr15	39250000	39500000	0,315786909
chr15	40900000	41150000	0,125512089
chr15	41600000	41850000	0,142770956
chr15	42600000	42850000	0,196437359
chr15	43250000	43500000	0,204870699
chr15	43800000	44050000	0,170901125
chr15	44500000	44750000	0,225941828
chr15	45550000	45800000	0,245864803

chr15	69600000	69850000	0,261345775
chr15	70600000	70850000	0,203283358
chr15	72350000	72600000	0,198120848
chr15	74750000	75000000	0,141473377
chr15	75850000	76100000	0,146132864
chr15	77150000	77400000	0,287043347
chr15	78350000	78600000	0,21990515
chr15	78900000	79150000	0,197934568
chr15	80250000	80500000	0,378238155
chr15	80950000	81200000	0,38961702
chr15	83050000	83300000	0,243076605
chr15	85300000	85550000	0,147481421
chr15	85650000	85900000	0,149904126
chr15	88100000	88350000	0,508522216
chr15	90700000	90950000	0,12332317
chr15	92200000	92450000	0,603361898
chr15	93250000	93500000	0,182386499
chr15	94650000	94900000	0,329247428
chr15	96800000	97050000	0,667316487
chr15	99450000	99700000	0,192963151
chr15	101200000	101450000	0,186072446
chr16	450000	700000	0,129565238
chr16	1400000	1650000	0,170236519
chr16	2050000	2300000	0,132408336
chr16	2800000	3050000	0,127948914
chr16	4100000	4350000	0,123876809
chr16	6600000	6850000	0,849321235

chr15	48000000	48250000	0,319039444
chr15	49000000	49250000	0,249961753
chr15	50650000	50900000	0,157088767
chr15	52150000	52400000	0,147026048
chr15	56050000	56300000	0,229215537
chr15	57000000	57250000	0,322681383
chr15	57650000	57900000	0,302518124
chr15	59100000	59350000	0,188708563
chr15	59650000	59900000	0,178816094
chr15	60550000	60800000	0,198158542
chr15	63600000	63850000	0,187113123
chr15	64350000	64600000	0,215136833
chr15	65300000	65550000	0,144343726
chr15	67050000	67300000	0,131012002
chr15	68150000	68400000	0,190580448
chr15	69650000	69900000	0,241987895
chr15	70600000	70850000	0,202706935
chr15	72350000	72600000	0,195717527
chr15	74750000	75000000	0,153873386
chr15	75050000	75300000	0,152122878
chr15	75850000	76100000	0,155025813
chr15	77200000	77450000	0,282591092
chr15	78350000	78600000	0,228078693
chr15	78950000	79200000	0,193890186
chr15	80250000	80500000	0,367746177
chr15	80900000	81150000	0,386979547
chr15	82650000	82900000	0,261705088

chr16	8900000	9150000	0,233856022
chr16	10650000	10900000	0,244449022
chr16	11650000	11900000	0,13808229
chr16	14350000	14600000	0,166851984
chr16	14800000	15050000	0,193820666
chr16	15800000	16050000	0,167388409
chr16	19000000	19250000	0,167112906
chr16	21600000	21850000	0,226282477
chr16	22100000	22350000	0,175553093
chr16	23450000	23700000	0,399609915
chr16	24600000	24850000	0,183191762
chr16	27200000	27450000	0,403090437
chr16	28400000	28650000	0,140472572
chr16	29950000	30200000	0,126308484
chr16	30550000	30800000	0,127057783
chr16	32700000	32950000	0,71581951
chr16	33550000	33800000	0,705082626
chr16	34650000	34900000	0,776947999
chr16	46750000	47000000	0,23597518
chr16	48350000	48600000	0,323231331
chr16	50100000	50350000	0,253985817
chr16	53350000	53600000	0,4238192
chr16	54100000	54350000	0,558936066
chr16	54750000	55000000	0,680561993
chr16	55500000	55750000	0,618490662
chr16	56850000	57100000	0,132729159
chr16	57700000	57950000	0,149110447

chr15	83250000	83500000	0,308236101
chr15	85250000	85500000	0,151062316
chr15	85700000	85950000	0,153245794
chr15	88100000	88350000	0,545877364
chr15	90800000	91050000	0,125981596
chr15	93250000	93500000	0,173755364
chr15	94650000	94900000	0,35126281
chr15	96800000	97050000	0,623384585
chr15	99450000	99700000	0,194065289
chr15	101200000	101450000	0,202645609
chr16	450000	700000	0,12721111
chr16	1400000	1650000	0,170660006
chr16	2000000	2250000	0,135069549
chr16	2800000	3050000	0,131550986
chr16	4100000	4350000	0,129942195
chr16	8900000	9150000	0,224210558
chr16	10650000	10900000	0,230990187
chr16	11650000	11900000	0,14329721
chr16	14300000	14550000	0,181597356
chr16	15000000	15250000	0,234223791
chr16	15850000	16100000	0,180453268
chr16	19000000	19250000	0,17965466
chr16	21500000	21750000	0,222295804
chr16	22100000	22350000	0,180746831
chr16	23450000	23700000	0,389056861
chr16	24600000	24850000	0,212638615
chr16	27250000	27500000	0,378038161

chr16	58500000	58750000	0,224544123
chr16	60050000	60300000	0,808362565
chr16	61800000	62050000	0,465725698
chr16	64150000	64400000	0,863011291
chr16	66850000	67100000	0,13003924
chr16	67650000	67900000	0,127609863
chr16	68500000	68750000	0,187682087
chr16	69450000	69700000	0,122531776
chr16	70200000	70450000	0,134847382
chr16	71800000	72050000	0,198702446
chr16	73050000	73300000	0,288351723
chr16	75150000	75400000	0,166208965
chr16	77150000	77400000	0,657438534
chr16	77950000	78200000	0,703392037
chr16	78450000	78700000	0,638475306
chr16	80900000	81150000	0,165203763
chr16	81350000	81600000	0,153580833
chr16	84550000	84800000	0,15371409
chr16	85500000	85750000	0,19901116
chr16	86350000	86600000	0,348280616
chr16	87650000	87900000	0,150788581
chr16	88650000	88900000	0,165001574
chr16	89650000	89900000	0,145120091
chr17	0	150000	0,294869576
chr17	1400000	1650000	0,127437513
chr17	2400000	2650000	0,169561456
chr17	3700000	3950000	0,163326747

chr16	28500000	28750000	0,133774978
chr16	29950000	30200000	0,131791994
chr16	30550000	30800000	0,128957866
chr16	33050000	33300000	0,657507535
chr16	33550000	33800000	0,682694831
chr16	34700000	34950000	0,741999373
chr16	46750000	47000000	0,235752237
chr16	48350000	48600000	0,308357395
chr16	50100000	50350000	0,253789827
chr16	53350000	53600000	0,401572615
chr16	54050000	54300000	0,541145892
chr16	54850000	55100000	0,641956822
chr16	55500000	55750000	0,585192418
chr16	56850000	57100000	0,129264831
chr16	57750000	58000000	0,153825257
chr16	58500000	58750000	0,223227435
chr16	60000000	60250000	0,803969368
chr16	61800000	62050000	0,520880213
chr16	63200000	63450000	0,847172819
chr16	66900000	67150000	0,132575574
chr16	67550000	67800000	0,13018128
chr16	69450000	69700000	0,123404793
chr16	70200000	70450000	0,139909089
chr16	71800000	72050000	0,210802949
chr16	73000000	73250000	0,262445911
chr16	75150000	75400000	0,17804107
chr16	77150000	77400000	0,652222801

chr17	4550000	4800000	0,139517888
chr17	7250000	7500000	0,127440139
chr17	7950000	8200000	0,141775104
chr17	9550000	9800000	0,188743418
chr17	12350000	12600000	0,371146124
chr17	14050000	14300000	0,673585984
chr17	15400000	15650000	0,208084599
chr17	16200000	16450000	0,185043181
chr17	16900000	17150000	0,145459878
chr17	17800000	18050000	0,139410891
chr17	18900000	19150000	0,15038764
chr17	19550000	19800000	0,160646051
chr17	20900000	21150000	0,278907449
chr17	25650000	25900000	0,326172658
chr17	27050000	27300000	0,134716497
chr17	29150000	29400000	0,148507041
chr17	30400000	30650000	0,177097704
chr17	33950000	34200000	0,169602613
chr17	35650000	35900000	0,182730637
chr17	36700000	36950000	0,124983418
chr17	38200000	38450000	0,123174922
chr17	40050000	40300000	0,123321202
chr17	40550000	40800000	0,117750532
chr17	41300000	41550000	0,123756433
chr17	42900000	43150000	0,113809759
chr17	44350000	44600000	0,174330704
chr17	45550000	45800000	0,131653711

chr16	77950000	78200000	0,671097186
chr16	78500000	78750000	0,602267127
chr16	79100000	79350000	0,690949414
chr16	80850000	81100000	0,157570383
chr16	81350000	81600000	0,154390659
chr16	84600000	84850000	0,168494724
chr16	84950000	85200000	0,177855228
chr16	85500000	85750000	0,196613097
chr16	86500000	86750000	0,343832054
chr16	87650000	87900000	0,152631117
chr16	88650000	88900000	0,162319203
chr16	89650000	89900000	0,141637243
chr17	0	150000	0,30075671
chr17	1400000	1650000	0,131344325
chr17	2450000	2700000	0,173362213
chr17	3700000	3950000	0,164088769
chr17	4600000	4850000	0,145530365
chr17	7250000	7500000	0,131257961
chr17	7950000	8200000	0,150931928
chr17	9550000	9800000	0,178738993
chr17	12350000	12600000	0,384720187
chr17	14000000	14250000	0,633186302
chr17	15450000	15700000	0,224539576
chr17	16200000	16450000	0,18523868
chr17	16900000	17150000	0,145326678
chr17	17750000	18000000	0,136453173
chr17	18900000	19150000	0,154839403

chr17	47000000	47250000	0,120807447
chr17	48150000	48400000	0,146403655
chr17	48700000	48950000	0,127259051
chr17	53450000	53700000	0,395261701
chr17	54950000	55200000	0,181249106
chr17	55850000	56100000	0,189559325
chr17	56600000	56850000	0,214207858
chr17	57350000	57600000	0,222234055
chr17	58000000	58250000	0,160338618
chr17	60650000	60900000	0,164263621
chr17	61600000	61850000	0,187366848
chr17	62600000	62850000	0,155088248
chr17	64150000	64400000	0,284325317
chr17	65250000	65500000	0,206065966
chr17	65900000	66150000	0,167544113
chr17	67300000	67550000	0,263532467
chr17	69300000	69550000	0,622126225
chr17	71100000	71350000	0,294451773
chr17	73300000	73550000	0,11860148
chr17	75000000	75250000	0,168725394
chr17	76250000	76500000	0,140478214
chr17	78250000	78500000	0,140438344
chr17	79400000	79650000	0,120913409
chr17	80000000	80250000	0,122573086
chr18	650000	900000	0,306088517
chr18	2600000	2850000	0,240457008
chr18	3500000	3750000	0,163674893

chr17	19550000	19800000	0,166041386
chr17	20850000	21100000	0,272886941
chr17	25650000	25900000	0,3022644
chr17	27150000	27400000	0,141912658
chr17	27300000	27550000	0,141992102
chr17	28550000	28800000	0,203686113
chr17	29200000	29450000	0,155042172
chr17	30400000	30650000	0,194742139
chr17	33950000	34200000	0,171815046
chr17	35650000	35900000	0,204745907
chr17	36700000	36950000	0,129345984
chr17	38250000	38500000	0,127044593
chr17	40500000	40750000	0,119654937
chr17	41300000	41550000	0,127015704
chr17	42900000	43150000	0,120539591
chr17	44300000	44550000	0,174774467
chr17	45600000	45850000	0,136980656
chr17	47050000	47300000	0,124265087
chr17	48150000	48400000	0,145229036
chr17	48750000	49000000	0,128234449
chr17	53400000	53650000	0,395244521
chr17	54950000	55200000	0,188148781
chr17	55850000	56100000	0,196479518
chr17	56600000	56850000	0,220764633
chr17	57300000	57550000	0,230205747
chr17	57950000	58200000	0,159138288
chr17	60650000	60900000	0,164192966

chr18	5200000	5450000	0,711059516
chr18	6700000	6950000	0,175502053
chr18	8850000	9100000	0,13537726
chr18	9550000	9800000	0,142051184
chr18	11100000	11350000	0,23765383
chr18	11850000	12100000	0,159359012
chr18	12200000	12450000	0,159227666
chr18	14950000	15200000	0,746162482
chr18	19050000	19300000	0,200232044
chr18	20650000	20900000	0,153222328
chr18	23900000	24150000	0,357664236
chr18	25450000	25700000	0,668898368
chr18	26600000	26850000	0,765341783
chr18	28900000	29150000	0,382292161
chr18	29400000	29650000	0,349114861
chr18	32900000	33150000	0,22753628
chr18	33600000	33850000	0,266025529
chr18	34650000	34900000	0,512322055
chr18	36550000	36800000	0,709686429
chr18	37300000	37550000	0,681334298
chr18	39450000	39700000	0,718954499
chr18	42100000	42350000	0,445527046
chr18	43650000	43900000	0,185314938
chr18	45400000	45650000	0,197326249
chr18	46350000	46600000	0,163512167
chr18	48250000	48500000	0,267113979
chr18	51650000	51900000	0,364587249

chr17	61600000	61850000	0,195192252
chr17	62600000	62850000	0,169672776
chr17	64200000	64450000	0,299712496
chr17	65250000	65500000	0,222575976
chr17	66050000	66300000	0,169710346
chr17	67300000	67550000	0,249456691
chr17	69300000	69550000	0,586697048
chr17	71100000	71350000	0,287728682
chr17	73500000	73750000	0,121837686
chr17	74250000	74500000	0,138799642
chr17	75000000	75250000	0,167588098
chr17	76250000	76500000	0,13876953
chr17	78200000	78450000	0,142405198
chr17	79350000	79600000	0,121127679
chr17	80000000	80250000	0,122463257
chr18	650000	900000	0,293369281
chr18	2600000	2850000	0,240349354
chr18	3500000	3750000	0,152811832
chr18	5150000	5400000	0,692143473
chr18	6800000	7050000	0,185360955
chr18	8850000	9100000	0,142883463
chr18	9600000	9850000	0,148759673
chr18	11000000	11250000	0,242644972
chr18	11850000	12100000	0,154832779
chr18	14900000	15150000	0,730497799
chr18	19000000	19250000	0,214057181
chr18	20700000	20950000	0,158857164

chr18	52400000	52650000	0,395928165
chr18	55350000	55600000	0,194443907
chr18	56200000	56450000	0,19332366
chr18	57450000	57700000	0,397650134
chr18	60050000	60300000	0,240007282
chr18	60900000	61150000	0,252942637
chr18	65100000	65350000	0,485104538
chr18	66050000	66300000	0,603951067
chr18	67900000	68150000	0,32857877
chr18	70750000	71000000	0,370341194
chr18	71850000	72100000	0,376552272
chr18	72750000	73000000	0,579371381
chr18	74550000	74800000	0,408190514
chr18	77500000	77750000	0,319008404
chr19	750000	1000000	0,124220587
chr19	2050000	2300000	0,123533895
chr19	3250000	3500000	0,132275845
chr19	4150000	4400000	0,140024369
chr19	4650000	4900000	0,133694268
chr19	5650000	5900000	0,15103763
chr19	6400000	6650000	0,14766202
chr19	7500000	7750000	0,175924978
chr19	8300000	8550000	0,148333915
chr19	8950000	9200000	0,286018705
chr19	10550000	10800000	0,122747595
chr19	10900000	11150000	0,123521087
chr19	12900000	13150000	0,136785
chr18	23900000	24150000	0,339807359
chr18	25500000	25750000	0,680937765
chr18	26500000	26750000	0,760618672
chr18	28850000	29100000	0,361067675
chr18	29400000	29650000	0,338054397
chr18	32900000	33150000	0,226337361
chr18	33600000	33850000	0,269794758
chr18	36550000	36800000	0,681324133
chr18	37300000	37550000	0,655765286
chr18	39450000	39700000	0,69005478
chr18	42100000	42350000	0,430238371
chr18	43650000	43900000	0,195705448
chr18	45400000	45650000	0,182250867
chr18	46350000	46600000	0,167533113
chr18	48250000	48500000	0,27429238
chr18	51650000	51900000	0,328060449
chr18	52400000	52650000	0,367270463
chr18	55350000	55600000	0,226296469
chr18	56200000	56450000	0,193181324
chr18	57450000	57700000	0,395349874
chr18	60050000	60300000	0,249710032
chr18	60950000	61200000	0,227323544
chr18	62800000	63050000	0,821382158
chr18	65150000	65400000	0,454644085
chr18	67900000	68150000	0,331535036
chr18	70750000	71000000	0,371465812
chr18	71850000	72100000	0,381081843

chr19	14000000	14250000	0,142193624
chr19	14300000	14550000	0,148393935
chr19	15300000	15550000	0,297799671
chr19	16350000	16600000	0,1537347
chr19	17350000	17600000	0,145836994
chr19	18450000	18700000	0,121370862
chr19	21200000	21450000	0,399928319
chr19	23750000	24000000	0,647974331
chr19	27950000	28200000	0,723436285
chr19	30150000	30400000	0,410919623
chr19	33450000	33700000	0,204793935
chr19	34650000	34900000	0,221384746
chr19	35550000	35800000	0,267535533
chr19	36300000	36550000	0,191012223
chr19	38800000	39050000	0,155781749
chr19	39150000	39400000	0,149211568
chr19	39700000	39950000	0,183900047
chr19	40950000	41200000	0,141710279
chr19	41550000	41800000	0,191704316
chr19	42600000	42850000	0,174956267
chr19	43750000	44000000	0,133241398
chr19	45300000	45550000	0,133042693
chr19	45950000	46200000	0,118636895
chr19	47500000	47750000	0,120691161
chr19	49100000	49350000	0,122346919
chr19	50000000	50250000	0,132555051
chr19	51700000	51950000	0,379601184
chr18	72700000	72950000	0,56095468
chr18	74550000	74800000	0,38337537
chr18	77500000	77750000	0,301914715
chr19	900000	1150000	0,120875091
chr19	2100000	2350000	0,125747908
chr19	3250000	3500000	0,12922374
chr19	4700000	4950000	0,132881918
chr19	5600000	5850000	0,153058798
chr19	6400000	6650000	0,150809805
chr19	7500000	7750000	0,175264388
chr19	8300000	8550000	0,156990222
chr19	9000000	9250000	0,289833806
chr19	10500000	10750000	0,126230821
chr19	10900000	11150000	0,127027752
chr19	12900000	13150000	0,138628178
chr19	14000000	14250000	0,141404358
chr19	15300000	15550000	0,283956438
chr19	16400000	16650000	0,167100653
chr19	17350000	17600000	0,145271316
chr19	18450000	18700000	0,125825247
chr19	21200000	21450000	0,425964773
chr19	23750000	24000000	0,600722034
chr19	28000000	28250000	0,677782369
chr19	30150000	30400000	0,365956147
chr19	33450000	33700000	0,202289345
chr19	34650000	34900000	0,215041791
chr19	36300000	36550000	0,190163275

chr19	52600000	52850000	0,371734015
chr19	53150000	53400000	0,32455028
chr19	54300000	54550000	0,172550279
chr19	55800000	56050000	0,144336703
chr19	57750000	58000000	0,296651907
chr19	59000000	59128983	0,178338339
chr2	350000	600000	0,324499242
chr2	1600000	1850000	0,267865613
chr2	3450000	3700000	0,236074823
chr2	5550000	5800000	0,865597282
chr2	7000000	7250000	0,69970901
chr2	10250000	10500000	0,147092277
chr2	11350000	11600000	0,161023287
chr2	16300000	16550000	0,407295937
chr2	17850000	18100000	0,627097525
chr2	20450000	20700000	0,135408162
chr2	21600000	21850000	0,350726422
chr2	24250000	24500000	0,190053786
chr2	26100000	26350000	0,130828207
chr2	27450000	27700000	0,137259938
chr2	27800000	28050000	0,137447922
chr2	28550000	28800000	0,157071235
chr2	30400000	30650000	0,301345656
chr2	32250000	32500000	0,195514278
chr2	33000000	33250000	0,356653351
chr2	37350000	37600000	0,139142292
chr2	38850000	39100000	0,150910514

chr19	38700000	38950000	0,168040175
chr19	39200000	39450000	0,164224098
chr19	39750000	40000000	0,191026556
chr19	40950000	41200000	0,142451641
chr19	41550000	41800000	0,192397687
chr19	42600000	42850000	0,173639344
chr19	43850000	44100000	0,135419517
chr19	45300000	45550000	0,134316756
chr19	45950000	46200000	0,125290928
chr19	47500000	47750000	0,124568847
chr19	49000000	49250000	0,124599134
chr19	50000000	50250000	0,134713965
chr19	51750000	52000000	0,362412073
chr19	52600000	52850000	0,349515842
chr19	53150000	53400000	0,314492615
chr19	54300000	54550000	0,188410834
chr19	55800000	56050000	0,145907925
chr19	57750000	58000000	0,296971035
chr19	59000000	59128983	0,166332346
chr2	350000	600000	0,286150838
chr2	1600000	1850000	0,244492744
chr2	3450000	3700000	0,225685815
chr2	5600000	5850000	0,840055185
chr2	7000000	7250000	0,63133742
chr2	10250000	10500000	0,144416109
chr2	11350000	11600000	0,154268043
chr2	13800000	14050000	0,822206841

chr2	40450000	40700000	0,543983306
chr2	42150000	42400000	0,15039755
chr2	43200000	43450000	0,161684436
chr2	44250000	44500000	0,208120368
chr2	45750000	46000000	0,244799764
chr2	46500000	46750000	0,186712832
chr2	47250000	47500000	0,152607671
chr2	50650000	50900000	0,799661745
chr2	54200000	54450000	0,308735658
chr2	55950000	56200000	0,149293669
chr2	58550000	58800000	0,375184175
chr2	61150000	61400000	0,199486465
chr2	61850000	62100000	0,194727466
chr2	62700000	62950000	0,217385844
chr2	63850000	64100000	0,269263631
chr2	64950000	65200000	0,173183015
chr2	67500000	67750000	0,385710591
chr2	70100000	70350000	0,135910316
chr2	71150000	71400000	0,256589643
chr2	72200000	72450000	0,353607289
chr2	73100000	73350000	0,207974988
chr2	74450000	74700000	0,140881873
chr2	75600000	75850000	0,39363722
chr2	76400000	76650000	0,547079268
chr2	77400000	77650000	0,602763917
chr2	78000000	78250000	0,668272402
chr2	78600000	78850000	0,722762858

chr2	16250000	16500000	0,390346346
chr2	17850000	18100000	0,588862715
chr2	20450000	20700000	0,134079747
chr2	21600000	21850000	0,350884222
chr2	24250000	24500000	0,210640084
chr2	26150000	26400000	0,132492262
chr2	27500000	27750000	0,138853915
chr2	27850000	28100000	0,136466238
chr2	28550000	28800000	0,156775954
chr2	30400000	30650000	0,309435335
chr2	32300000	32550000	0,216751691
chr2	37350000	37600000	0,13724059
chr2	38850000	39100000	0,160388485
chr2	40450000	40700000	0,53300117
chr2	42150000	42400000	0,142562258
chr2	43200000	43450000	0,164002879
chr2	44300000	44550000	0,228332148
chr2	45800000	46050000	0,242111414
chr2	46400000	46650000	0,196631279
chr2	47300000	47550000	0,163914001
chr2	50650000	50900000	0,792400868
chr2	53750000	54000000	0,308523361
chr2	54200000	54450000	0,29417956
chr2	55950000	56200000	0,146380112
chr2	58550000	58800000	0,387351553
chr2	61200000	61450000	0,207246138
chr2	61850000	62100000	0,199977247

chr2	79900000	80150000	0,823060274
chr2	81600000	81850000	0,756116962
chr2	83450000	83700000	0,842737429
chr2	85600000	85850000	0,132247057
chr2	86650000	86900000	0,265861539
chr2	88050000	88300000	0,363539676
chr2	88800000	89050000	0,274969612
chr2	90000000	90250000	0,762678503
chr2	91800000	92050000	0,670314635
chr2	95850000	96100000	0,313466063
chr2	97150000	97400000	0,170362625
chr2	98100000	98350000	0,331608501
chr2	99600000	99850000	0,334250795
chr2	101800000	102050000	0,225478066
chr2	105900000	106150000	0,226200215
chr2	109250000	109500000	0,307817769
chr2	110800000	111050000	0,275724905
chr2	112350000	112600000	0,206027232
chr2	112950000	113200000	0,183834964
chr2	113900000	114150000	0,267344993
chr2	114400000	114650000	0,292723658
chr2	118750000	119000000	0,377587974
chr2	120800000	121050000	0,246362572
chr2	121550000	121800000	0,227711154
chr2	122300000	122550000	0,275501458
chr2	124950000	125200000	0,853045264
chr2	128350000	128600000	0,157420335

chr2	62650000	62900000	0,257186181
chr2	63900000	64150000	0,280695719
chr2	64950000	65200000	0,19606801
chr2	67500000	67750000	0,404201333
chr2	70100000	70350000	0,14095478
chr2	71200000	71450000	0,261921785
chr2	72250000	72500000	0,313908234
chr2	73100000	73350000	0,211301211
chr2	74400000	74650000	0,145108401
chr2	75600000	75850000	0,405452237
chr2	76400000	76650000	0,512667478
chr2	77450000	77700000	0,657826091
chr2	78050000	78300000	0,643428603
chr2	78650000	78900000	0,718040593
chr2	80000000	80250000	0,788827704
chr2	81600000	81850000	0,708765443
chr2	83300000	83550000	0,820579736
chr2	85600000	85850000	0,137315262
chr2	86700000	86950000	0,262367087
chr2	87800000	88050000	0,334227821
chr2	88800000	89050000	0,301157674
chr2	90200000	90450000	0,726643598
chr2	91800000	92050000	0,648796201
chr2	95850000	96100000	0,300975948
chr2	97200000	97450000	0,186026489
chr2	98150000	98400000	0,315722697
chr2	99500000	99750000	0,324952076

chr2	129800000	130050000	0,64075068
chr2	131600000	131850000	0,223525218
chr2	133050000	133300000	0,546120922
chr2	135750000	136000000	0,357280547
chr2	136450000	136700000	0,284940011
chr2	137950000	138200000	0,640432704
chr2	138650000	138900000	0,664309619
chr2	139250000	139500000	0,620007506
chr2	140800000	141050000	0,614688706
chr2	141850000	142100000	0,567487823
chr2	142200000	142450000	0,571077238
chr2	143450000	143700000	0,307098292
chr2	144300000	144550000	0,431336918
chr2	145050000	145300000	0,455401136
chr2	147200000	147450000	0,711367939
chr2	148500000	148750000	0,669552623
chr2	149250000	149500000	0,410842511
chr2	151200000	151450000	0,348263493
chr2	152900000	153150000	0,219843885
chr2	154100000	154350000	0,594777776
chr2	157150000	157400000	0,355150705
chr2	157850000	158100000	0,480257968
chr2	159750000	160000000	0,253986747
chr2	160900000	161150000	0,202116498
chr2	161850000	162100000	0,316498537
chr2	162850000	163100000	0,433693279
chr2	164250000	164500000	0,385958629
chr2	101850000	102100000	0,217865744
chr2	105900000	106150000	0,239491372
chr2	109250000	109500000	0,291409595
chr2	110750000	111000000	0,308169179
chr2	112250000	112500000	0,204009541
chr2	113050000	113300000	0,202456482
chr2	113850000	114100000	0,263628715
chr2	114350000	114600000	0,278485716
chr2	115950000	116200000	0,812918155
chr2	116850000	117100000	0,814205668
chr2	118750000	119000000	0,38621469
chr2	120850000	121100000	0,244171267
chr2	121550000	121800000	0,222564948
chr2	122300000	122550000	0,283354285
chr2	124900000	125150000	0,819585395
chr2	125600000	125850000	0,808420231
chr2	128300000	128550000	0,150158172
chr2	129800000	130050000	0,596869831
chr2	131550000	131800000	0,205944046
chr2	133100000	133350000	0,521085255
chr2	134800000	135050000	0,601213627
chr2	135750000	136000000	0,34725676
chr2	136500000	136750000	0,275742754
chr2	138050000	138300000	0,656810228
chr2	138700000	138950000	0,638585905
chr2	139250000	139500000	0,598102731
chr2	140800000	141050000	0,590625512

chr2	165600000	165850000	0,283587889
chr2	166750000	167000000	0,648238391
chr2	169150000	169400000	0,455992179
chr2	170400000	170650000	0,235264673
chr2	172050000	172300000	0,19589497
chr2	172750000	173000000	0,178549795
chr2	173750000	174000000	0,237031586
chr2	175300000	175550000	0,257985241
chr2	176800000	177050000	0,498784842
chr2	178000000	178250000	0,182131316
chr2	179150000	179400000	0,314642931
chr2	180000000	180250000	0,392131653
chr2	180600000	180850000	0,451943882
chr2	181550000	181800000	0,498993365
chr2	182600000	182850000	0,241113213
chr2	183750000	184000000	0,299048722
chr2	185050000	185300000	0,42492095
chr2	185850000	186100000	0,513427717
chr2	187300000	187550000	0,262977587
chr2	188300000	188550000	0,346850384
chr2	190400000	190650000	0,228849146
chr2	191550000	191800000	0,140449721
chr2	196300000	196550000	0,247687743
chr2	196950000	197200000	0,287173519
chr2	198200000	198450000	0,267311995
chr2	201600000	201850000	0,166978478
chr2	203050000	203300000	0,185688855

chr2	141750000	142000000	0,542688868
chr2	143450000	143700000	0,29404052
chr2	144400000	144650000	0,468395488
chr2	145050000	145300000	0,455708513
chr2	147150000	147400000	0,71075676
chr2	148550000	148800000	0,622096365
chr2	149250000	149500000	0,402472752
chr2	150550000	150800000	0,362058458
chr2	151150000	151400000	0,349682
chr2	152900000	153150000	0,234389373
chr2	154100000	154350000	0,501390235
chr2	157150000	157400000	0,319340666
chr2	159800000	160050000	0,248764751
chr2	160950000	161200000	0,20708429
chr2	161850000	162100000	0,300961076
chr2	162900000	163150000	0,483112692
chr2	164250000	164500000	0,383254704
chr2	165600000	165850000	0,278760279
chr2	166750000	167000000	0,599546893
chr2	169600000	169850000	0,438295109
chr2	170400000	170650000	0,245185725
chr2	172150000	172400000	0,212484585
chr2	172750000	173000000	0,180838063
chr2	173750000	174000000	0,241556826
chr2	175300000	175550000	0,261270264
chr2	176800000	177050000	0,465070216
chr2	178000000	178250000	0,177241158

chr2	204000000	204250000	0,209554611
chr2	205200000	205450000	0,304199942
chr2	206900000	207150000	0,253052021
chr2	208450000	208700000	0,212083932
chr2	210250000	210500000	0,466965954
chr2	211250000	211500000	0,190362955
chr2	213250000	213500000	0,553273324
chr2	216050000	216300000	0,538179921
chr2	216900000	217150000	0,348494956
chr2	219250000	219500000	0,147799933
chr2	220000000	220250000	0,1571698
chr2	223550000	223800000	0,250619186
chr2	224600000	224850000	0,243974323
chr2	225300000	225550000	0,353753772
chr2	226200000	226450000	0,281762841
chr2	227000000	227250000	0,289966392
chr2	227350000	227600000	0,286906639
chr2	228200000	228450000	0,205885181
chr2	230500000	230750000	0,236030304
chr2	231450000	231700000	0,229213092
chr2	232350000	232600000	0,141302284
chr2	233300000	233550000	0,142027937
chr2	234150000	234400000	0,16797382
chr2	236000000	236250000	0,214612692
chr2	237000000	237250000	0,343978858
chr2	238900000	239150000	0,189620445
chr2	239700000	239950000	0,319141675

chr2	179150000	179400000	0,299457563
chr2	180000000	180250000	0,384172174
chr2	180600000	180850000	0,447632956
chr2	181700000	181950000	0,529698699
chr2	182600000	182850000	0,229368746
chr2	183750000	184000000	0,282274751
chr2	185050000	185300000	0,465291376
chr2	185850000	186100000	0,535502454
chr2	187300000	187550000	0,283315116
chr2	188300000	188550000	0,330099129
chr2	190350000	190600000	0,248764395
chr2	191550000	191800000	0,145233724
chr2	192300000	192550000	0,226630612
chr2	196300000	196550000	0,238676244
chr2	196900000	197150000	0,296530379
chr2	198150000	198400000	0,258673539
chr2	201600000	201850000	0,177902765
chr2	203050000	203300000	0,18885422
chr2	204000000	204250000	0,206989925
chr2	205200000	205450000	0,315306337
chr2	206900000	207150000	0,253659986
chr2	208500000	208750000	0,236462383
chr2	210250000	210500000	0,43951084
chr2	211250000	211500000	0,179304756
chr2	213250000	213500000	0,536647868
chr2	216050000	216300000	0,516948024
chr2	216900000	217150000	0,340916223

chr2	240500000	240750000	0,311967594
chr2	241200000	241450000	0,249149818
chr2	242350000	242600000	0,195789099
chr20	300000	550000	0,242130031
chr20	1200000	1450000	0,291047268
chr20	3100000	3350000	0,181251914
chr20	3700000	3950000	0,153594644
chr20	4800000	5050000	0,284237046
chr20	5700000	5950000	0,331578621
chr20	6500000	6750000	0,451677478
chr20	7950000	8200000	0,738254794
chr20	8900000	9150000	0,720826141
chr20	10200000	10450000	0,280287539
chr20	11650000	11900000	0,271082609
chr20	13000000	13250000	0,557985616
chr20	13600000	13850000	0,478185664
chr20	15000000	15250000	0,554872981
chr20	16450000	16700000	0,591642236
chr20	17450000	17700000	0,230713645
chr20	19800000	20050000	0,348547908
chr20	21100000	21350000	0,379136957
chr20	22650000	22900000	0,366515373
chr20	25200000	25450000	0,251726727
chr20	30150000	30400000	0,167661746
chr20	30800000	31050000	0,16829453
chr20	32200000	32450000	0,139599116
chr20	32900000	33150000	0,137322967

chr2	219250000	219500000	0,148827804
chr2	220000000	220250000	0,165224581
chr2	223550000	223800000	0,234888892
chr2	224550000	224800000	0,239769677
chr2	225350000	225600000	0,330428689
chr2	226200000	226450000	0,27674105
chr2	226900000	227150000	0,266806233
chr2	227350000	227600000	0,276163565
chr2	228150000	228400000	0,214486874
chr2	230500000	230750000	0,203550136
chr2	231450000	231700000	0,223495802
chr2	232350000	232600000	0,146946872
chr2	233250000	233500000	0,143961695
chr2	234200000	234450000	0,174766182
chr2	236050000	236300000	0,218323154
chr2	236900000	237150000	0,294798685
chr2	238950000	239200000	0,196299681
chr2	239750000	240000000	0,289366862
chr2	240450000	240700000	0,256315315
chr2	241200000	241450000	0,237871096
chr2	242350000	242600000	0,198427097
chr20	300000	550000	0,226905576
chr20	1200000	1450000	0,298079664
chr20	3050000	3300000	0,192938673
chr20	3750000	4000000	0,157446734
chr20	4800000	5050000	0,280225835
chr20	5600000	5850000	0,321524532

chr20	34200000	34450000	0,119754473	chr20	6500000	6750000	0,466088275
chr20	35200000	35450000	0,141420502	chr20	7900000	8150000	0,733468366
chr20	36700000	36950000	0,179744417	chr20	10200000	10450000	0,258859868
chr20	39700000	39950000	0,2381861	chr20	11650000	11900000	0,232523258
chr20	42150000	42400000	0,260084959	chr20	13550000	13800000	0,479089109
chr20	43250000	43500000	0,144974613	chr20	14950000	15200000	0,534479962
chr20	44350000	44600000	0,204256842	chr20	16500000	16750000	0,565216338
chr20	45900000	46150000	0,188124225	chr20	17450000	17700000	0,218291466
chr20	47650000	47900000	0,179214178	chr20	19750000	20000000	0,328145014
chr20	48850000	49100000	0,114635274	chr20	21050000	21300000	0,356059857
chr20	50350000	50600000	0,164903783	chr20	22650000	22900000	0,40324967
chr20	52300000	52550000	0,193845194	chr20	25200000	25450000	0,242690015
chr20	55150000	55400000	0,362016553	chr20	30150000	30400000	0,172663034
chr20	56050000	56300000	0,372743326	chr20	30800000	31050000	0,170929258
chr20	56900000	57150000	0,352511158	chr20	32200000	32450000	0,144995639
chr20	57400000	57650000	0,338901862	chr20	32900000	33150000	0,146485865
chr20	58350000	58600000	0,600207129	chr20	34200000	34450000	0,124016225
chr20	61200000	61450000	0,162598339	chr20	35250000	35500000	0,153756422
chr20	62300000	62550000	0,144877885	chr20	36700000	36950000	0,20867502
chr21	9800000	10050000	0,708594146	chr20	39700000	39950000	0,240902366
chr21	10650000	10900000	0,724208329	chr20	42200000	42450000	0,257715261
chr21	14500000	14750000	0,717931758	chr20	43250000	43500000	0,143234401
chr21	17000000	17250000	0,320738294	chr20	44350000	44600000	0,210007568
chr21	17900000	18150000	0,391194468	chr20	45900000	46150000	0,181457345
chr21	18700000	18950000	0,273551233	chr20	47650000	47900000	0,184774089
chr21	21450000	21700000	0,847449199	chr20	48850000	49100000	0,119398986
chr21	22050000	22300000	0,854821475	chr20	50350000	50600000	0,160802489

chr21	23300000	23550000	0,852918835
chr21	23900000	24150000	0,851840893
chr21	24350000	24600000	0,850745314
chr21	27050000	27300000	0,551697996
chr21	30400000	30650000	0,178840853
chr21	33750000	34000000	0,17374274
chr21	34650000	34900000	0,171416194
chr21	36300000	36550000	0,37520602
chr21	37550000	37800000	0,207438651
chr21	38450000	38700000	0,203950108
chr21	40200000	40450000	0,189365361
chr21	43000000	43250000	0,18580523
chr21	44900000	45150000	0,122255891
chr21	46350000	46600000	0,143682611
chr21	47400000	47650000	0,311603543
chr21	48000000	48129895	0,323181112
chr22	16250000	16500000	0,699213857
chr22	18250000	18500000	0,159153401
chr22	19100000	19350000	0,17335924
chr22	19700000	19950000	0,135304626
chr22	21050000	21300000	0,185364347
chr22	21900000	22150000	0,152269492
chr22	24400000	24650000	0,129864268
chr22	26850000	27100000	0,364776906
chr22	28100000	28350000	0,265499546
chr22	29750000	30000000	0,137752131
chr22	30500000	30750000	0,139696656

chr20	52350000	52600000	0,215823527
chr20	55150000	55400000	0,345518318
chr20	56100000	56350000	0,346030908
chr20	57000000	57250000	0,332913107
chr20	57350000	57600000	0,320066952
chr20	58300000	58550000	0,570494722
chr20	61200000	61450000	0,155459867
chr20	62300000	62550000	0,145947894
chr21	9750000	10000000	0,674901725
chr21	10700000	10950000	0,679189332
chr21	14600000	14850000	0,720927514
chr21	17350000	17600000	0,286462357
chr21	18700000	18950000	0,276144031
chr21	20800000	21050000	0,818381718
chr21	22100000	22350000	0,834296123
chr21	23000000	23250000	0,831865219
chr21	23900000	24150000	0,830537598
chr21	27100000	27350000	0,526811833
chr21	30450000	30700000	0,170542757
chr21	33750000	34000000	0,175452902
chr21	34650000	34900000	0,189856228
chr21	37500000	37750000	0,216072109
chr21	38450000	38700000	0,206446612
chr21	40200000	40450000	0,180889577
chr21	43050000	43300000	0,186429862
chr21	44850000	45100000	0,122097248
chr21	46350000	46600000	0,14412974

chr22	31550000	31800000	0,149240935
chr22	32850000	33100000	0,360087228
chr22	35800000	36050000	0,168929999
chr22	36600000	36850000	0,158669238
chr22	37950000	38200000	0,12069452
chr22	39550000	39800000	0,156998047
chr22	41650000	41900000	0,120288194
chr22	42800000	43050000	0,135662156
chr22	44850000	45100000	0,21433977
chr22	46150000	46400000	0,119109116
chr22	48650000	48900000	0,580480259
chr22	50650000	50900000	0,150591429
chr3	250000	500000	0,765118923
chr3	2200000	2450000	0,846382304
chr3	3100000	3350000	0,707784874
chr3	4500000	4750000	0,223360623
chr3	4850000	5100000	0,20331385
chr3	6450000	6700000	0,663734667
chr3	8650000	8900000	0,347447273
chr3	9900000	10150000	0,140919325
chr3	11200000	11450000	0,269692909
chr3	11700000	11950000	0,267069519
chr3	12500000	12750000	0,166951662
chr3	13250000	13500000	0,26492354
chr3	14300000	14550000	0,319846562
chr3	15450000	15700000	0,201845023
chr3	16750000	17000000	0,233416227

chr21	47400000	47650000	0,297526729
chr21	48000000	48129895	0,309547472
chr22	16250000	16500000	0,689123135
chr22	18250000	18500000	0,162559705
chr22	19100000	19350000	0,184755851
chr22	19750000	20000000	0,132741811
chr22	21100000	21350000	0,189407758
chr22	21850000	22100000	0,146948245
chr22	24350000	24600000	0,130003493
chr22	26850000	27100000	0,365809793
chr22	28100000	28350000	0,264234934
chr22	29200000	29450000	0,176191546
chr22	29750000	30000000	0,140050432
chr22	30550000	30800000	0,158963353
chr22	31500000	31750000	0,151584867
chr22	35750000	36000000	0,16347978
chr22	36650000	36900000	0,170749875
chr22	38050000	38300000	0,123459828
chr22	39550000	39800000	0,165597918
chr22	41100000	41350000	0,1349843
chr22	41700000	41950000	0,127154144
chr22	42800000	43050000	0,138205896
chr22	44800000	45050000	0,223233866
chr22	46150000	46400000	0,124842214
chr22	48650000	48900000	0,552204803
chr22	50650000	50900000	0,14687238
chr3	250000	500000	0,730595564

chr3	17850000	18100000	0,327087148
chr3	19950000	20200000	0,439459836
chr3	22050000	22300000	0,783714757
chr3	23850000	24100000	0,277798637
chr3	25500000	25750000	0,423342551
chr3	27400000	27650000	0,267389488
chr3	29250000	29500000	0,487538239
chr3	30450000	30700000	0,299321302
chr3	31200000	31450000	0,371756227
chr3	33100000	33350000	0,192934688
chr3	37150000	37400000	0,211414954
chr3	38050000	38300000	0,233934243
chr3	39050000	39300000	0,335650585
chr3	40400000	40650000	0,412768854
chr3	41950000	42200000	0,307696977
chr3	42600000	42850000	0,265473412
chr3	43100000	43350000	0,299231802
chr3	44900000	45150000	0,220317929
chr3	45450000	45700000	0,250072614
chr3	48000000	48250000	0,133428199
chr3	48700000	48950000	0,13131545
chr3	49350000	49600000	0,126516777
chr3	50000000	50250000	0,121758452
chr3	52200000	52450000	0,129842658
chr3	53050000	53300000	0,142579437
chr3	53850000	54100000	0,292919778
chr3	55200000	55450000	0,241296828

chr3	1650000	1900000	0,812765205
chr3	3100000	3350000	0,676447172
chr3	4850000	5100000	0,190598164
chr3	6400000	6650000	0,621613541
chr3	8700000	8950000	0,33744559
chr3	9900000	10150000	0,147522144
chr3	11250000	11500000	0,287765051
chr3	11650000	11900000	0,271336126
chr3	12550000	12800000	0,175405905
chr3	13250000	13500000	0,260815485
chr3	14350000	14600000	0,298474005
chr3	15500000	15750000	0,216929145
chr3	16750000	17000000	0,212928018
chr3	17800000	18050000	0,326328988
chr3	19950000	20200000	0,422192672
chr3	22050000	22300000	0,757948082
chr3	23850000	24100000	0,292205031
chr3	25550000	25800000	0,424741098
chr3	27450000	27700000	0,264842682
chr3	30450000	30700000	0,29724921
chr3	31300000	31550000	0,345935602
chr3	32150000	32400000	0,268779277
chr3	33100000	33350000	0,21974314
chr3	37100000	37350000	0,227769265
chr3	38050000	38300000	0,251501432
chr3	39100000	39350000	0,32684941
chr3	40400000	40650000	0,411507709

chr3	57750000	58000000	0,136485836
chr3	60300000	60550000	0,626904342
chr3	60950000	61200000	0,640182389
chr3	62400000	62650000	0,804438264
chr3	63700000	63950000	0,701107186
chr3	65200000	65450000	0,662835295
chr3	65900000	66150000	0,426328795
chr3	66400000	66650000	0,465723178
chr3	67750000	68000000	0,610542366
chr3	69650000	69900000	0,218482171
chr3	71550000	71800000	0,380668357
chr3	72850000	73100000	0,312568194
chr3	75400000	75650000	0,700280314
chr3	77350000	77600000	0,753928283
chr3	79800000	80050000	0,473902599
chr3	81650000	81900000	0,503574788
chr3	87000000	87250000	0,263686581
chr3	87900000	88150000	0,470344159
chr3	90100000	90350000	0,811708203
chr3	93700000	93950000	0,479756708
chr3	95950000	96200000	0,833319944
chr3	97500000	97750000	0,461658624
chr3	98450000	98700000	0,405774953
chr3	99950000	100200000	0,304256869
chr3	101550000	101800000	0,210511931
chr3	104750000	105000000	0,460996015
chr3	105450000	105700000	0,518032827

chr3	42000000	42250000	0,306213289
chr3	42650000	42900000	0,264777537
chr3	44950000	45200000	0,241118545
chr3	45400000	45650000	0,259531449
chr3	48000000	48250000	0,136593969
chr3	48700000	48950000	0,131725223
chr3	49350000	49600000	0,12867209
chr3	50000000	50250000	0,127683261
chr3	52250000	52500000	0,133615936
chr3	53050000	53300000	0,145123052
chr3	53850000	54100000	0,273087706
chr3	55200000	55450000	0,248742465
chr3	57750000	58000000	0,140437095
chr3	59750000	60000000	0,657678175
chr3	61000000	61250000	0,684308725
chr3	63800000	64050000	0,678822755
chr3	65900000	66150000	0,41535771
chr3	66350000	66600000	0,458301351
chr3	67750000	68000000	0,594094547
chr3	69650000	69900000	0,220181022
chr3	71550000	71800000	0,372000328
chr3	72850000	73100000	0,318081471
chr3	75400000	75650000	0,673154485
chr3	77400000	77650000	0,735267463
chr3	79800000	80050000	0,49010624
chr3	81700000	81950000	0,482707718
chr3	83800000	84050000	0,820028162

chr3	106950000	107200000	0,328485232
chr3	108850000	109100000	0,524214174
chr3	111450000	111700000	0,204168695
chr3	112250000	112500000	0,320162369
chr3	113350000	113600000	0,249370962
chr3	114650000	114900000	0,377969185
chr3	115250000	115500000	0,456102026
chr3	118950000	119200000	0,321116398
chr3	119850000	120100000	0,198418787
chr3	121300000	121550000	0,37115234
chr3	122250000	122500000	0,251260666
chr3	123500000	123750000	0,269956525
chr3	124600000	124850000	0,161564688
chr3	126050000	126300000	0,329488147
chr3	127400000	127650000	0,213896084
chr3	128950000	129200000	0,143827067
chr3	130400000	130650000	0,506427717
chr3	130950000	131200000	0,51780508
chr3	132100000	132350000	0,424431713
chr3	133200000	133450000	0,234605469
chr3	133900000	134150000	0,160975363
chr3	136400000	136650000	0,196768449
chr3	137900000	138150000	0,215535841
chr3	138350000	138600000	0,239605907
chr3	141100000	141350000	0,194724461
chr3	146000000	146250000	0,611280278
chr3	149050000	149300000	0,16096783

chr3	87000000	87250000	0,249349643
chr3	87900000	88150000	0,44209381
chr3	90050000	90300000	0,781737365
chr3	93700000	93950000	0,455276221
chr3	97500000	97750000	0,454485593
chr3	98450000	98700000	0,438910953
chr3	99950000	100200000	0,296253425
chr3	101450000	101700000	0,246949923
chr3	104800000	105050000	0,491667709
chr3	106950000	107200000	0,295664696
chr3	108850000	109100000	0,520132943
chr3	111400000	111650000	0,206474256
chr3	112150000	112400000	0,337188269
chr3	113300000	113550000	0,254321412
chr3	114700000	114950000	0,354995683
chr3	118950000	119200000	0,306227409
chr3	119850000	120100000	0,199946772
chr3	121300000	121550000	0,3510088
chr3	122300000	122550000	0,245657614
chr3	123450000	123700000	0,247329529
chr3	124600000	124850000	0,176009241
chr3	126000000	126250000	0,305714057
chr3	127350000	127600000	0,234019513
chr3	128300000	128550000	0,193905812
chr3	128950000	129200000	0,14555454
chr3	130400000	130650000	0,511225025
chr3	130900000	131150000	0,520472875

chr3	149950000	150200000	0,158611787
chr3	151750000	152000000	0,297432917
chr3	152750000	153000000	0,398022788
chr3	153350000	153600000	0,489070725
chr3	155450000	155700000	0,317945451
chr3	156400000	156650000	0,229882756
chr3	158300000	158550000	0,427573085
chr3	159400000	159650000	0,266347542
chr3	160100000	160350000	0,288799961
chr3	162450000	162700000	0,750495301
chr3	163650000	163900000	0,835703551
chr3	164900000	165150000	0,82661878
chr3	165400000	165650000	0,814308944
chr3	167700000	167950000	0,337171802
chr3	168600000	168850000	0,325649859
chr3	169800000	170050000	0,19843411
chr3	171450000	171700000	0,198706881
chr3	172200000	172450000	0,167534805
chr3	174050000	174300000	0,581410435
chr3	177000000	177250000	0,284360547
chr3	179150000	179400000	0,282635919
chr3	180500000	180750000	0,659247747
chr3	181450000	181700000	0,660577732
chr3	182700000	182950000	0,251003071
chr3	183800000	184050000	0,187340145
chr3	185350000	185600000	0,177424681
chr3	186350000	186600000	0,250087161

chr3	132100000	132350000	0,432804938
chr3	133200000	133450000	0,26064064
chr3	133900000	134150000	0,172607106
chr3	136400000	136650000	0,189935659
chr3	137900000	138150000	0,234066707
chr3	138300000	138550000	0,248848888
chr3	141100000	141350000	0,210904949
chr3	146050000	146300000	0,606910849
chr3	147050000	147300000	0,801702974
chr3	149000000	149250000	0,174241449
chr3	149950000	150200000	0,169313213
chr3	151750000	152000000	0,315754091
chr3	152700000	152950000	0,383270297
chr3	153600000	153850000	0,486156857
chr3	155450000	155700000	0,314335124
chr3	156450000	156700000	0,237259156
chr3	158250000	158500000	0,418543325
chr3	159400000	159650000	0,279955912
chr3	160100000	160350000	0,295537193
chr3	162450000	162700000	0,745755483
chr3	163100000	163350000	0,799165773
chr3	163700000	163950000	0,809668178
chr3	165400000	165650000	0,795356501
chr3	167700000	167950000	0,330835107
chr3	168700000	168950000	0,308346571
chr3	169800000	170050000	0,215989036
chr3	171450000	171700000	0,225364903

chr3	187350000	187600000	0,341538279
chr3	187550000	187800000	0,341250893
chr3	188900000	189150000	0,299808397
chr3	189800000	190050000	0,225651685
chr3	190900000	191150000	0,538852685
chr3	191850000	192100000	0,431992692
chr3	192450000	192700000	0,482037024
chr3	194050000	194300000	0,162140488
chr3	195850000	196100000	0,13286706
chr3	197100000	197350000	0,16311266
chr4	850000	1100000	0,194730502
chr4	1850000	2100000	0,157424504
chr4	2400000	2650000	0,145519555
chr4	4100000	4350000	0,363744346
chr4	6700000	6950000	0,189578502
chr4	7950000	8200000	0,143665658
chr4	9950000	10200000	0,539493545
chr4	13600000	13850000	0,372683851
chr4	15650000	15900000	0,208663487
chr4	17500000	17750000	0,423519316
chr4	19250000	19500000	0,727630759
chr4	19800000	20050000	0,720362912
chr4	20500000	20750000	0,708757263
chr4	24150000	24400000	0,188853008
chr4	25100000	25350000	0,328240906
chr4	29950000	30200000	0,855022948
chr4	31100000	31350000	0,84057927

chr3	172200000	172450000	0,170103932
chr3	174100000	174350000	0,554480393
chr3	177000000	177250000	0,294352536
chr3	179100000	179350000	0,280009874
chr3	180500000	180750000	0,63302179
chr3	181450000	181700000	0,643310249
chr3	182750000	183000000	0,26497052
chr3	183850000	184100000	0,199267449
chr3	185400000	185650000	0,188528842
chr3	186350000	186600000	0,253676287
chr3	187450000	187700000	0,327814889
chr3	188900000	189150000	0,277793889
chr3	189750000	190000000	0,232918133
chr3	190900000	191150000	0,546070185
chr3	191900000	192150000	0,45152461
chr3	192450000	192700000	0,476772974
chr3	194050000	194300000	0,167628792
chr3	195800000	196050000	0,132656775
chr3	197150000	197400000	0,172375736
chr4	900000	1150000	0,190209025
chr4	1750000	2000000	0,160713573
chr4	2400000	2650000	0,150281195
chr4	6700000	6950000	0,189833054
chr4	7900000	8150000	0,14527411
chr4	9950000	10200000	0,574031456
chr4	11250000	11500000	0,811081092
chr4	13550000	13800000	0,317691862

chr4	34000000	34250000	0,571447212
chr4	36000000	36250000	0,443614035
chr4	37850000	38100000	0,298009516
chr4	39550000	39800000	0,136466114
chr4	40450000	40700000	0,179415348
chr4	41200000	41450000	0,164164498
chr4	44600000	44850000	0,71077541
chr4	45500000	45750000	0,564635972
chr4	48150000	48400000	0,234272482
chr4	53500000	53750000	0,450685632
chr4	54100000	54350000	0,442549977
chr4	54800000	55050000	0,46597011
chr4	56200000	56450000	0,250907986
chr4	56700000	56950000	0,276449134
chr4	57750000	58000000	0,276724359
chr4	60750000	61000000	0,842683436
chr4	61600000	61850000	0,846566755
chr4	62500000	62750000	0,848729906
chr4	66550000	66800000	0,508137269
chr4	67300000	67550000	0,403482261
chr4	68300000	68550000	0,424381567
chr4	69050000	69300000	0,400626955
chr4	71550000	71800000	0,343079869
chr4	74900000	75150000	0,300606181
chr4	76800000	77050000	0,226133551
chr4	77550000	77800000	0,210491186
chr4	78750000	79000000	0,260269899

chr4	15650000	15900000	0,199246589
chr4	17500000	17750000	0,410270682
chr4	19300000	19550000	0,703384358
chr4	19800000	20050000	0,703781032
chr4	20500000	20750000	0,68884688
chr4	24150000	24400000	0,17152066
chr4	25150000	25400000	0,305796261
chr4	29950000	30200000	0,834773363
chr4	30600000	30850000	0,836370787
chr4	31450000	31700000	0,80871661
chr4	34000000	34250000	0,581358862
chr4	36000000	36250000	0,440119727
chr4	37900000	38150000	0,286179878
chr4	39550000	39800000	0,141970297
chr4	40450000	40700000	0,174693973
chr4	41200000	41450000	0,165553917
chr4	44600000	44850000	0,686870383
chr4	45500000	45750000	0,562446753
chr4	48150000	48400000	0,254092347
chr4	53500000	53750000	0,444171999
chr4	54100000	54350000	0,433901299
chr4	54800000	55050000	0,482471642
chr4	56200000	56450000	0,248692997
chr4	56700000	56950000	0,277555972
chr4	57750000	58000000	0,257880191
chr4	59700000	59950000	0,802103813
chr4	60800000	61050000	0,809361626

chr4	79400000	79650000	0,226938845
chr4	80500000	80750000	0,539219319
chr4	82000000	82250000	0,681540198
chr4	83950000	84200000	0,134069626
chr4	85450000	85700000	0,551889902
chr4	88050000	88300000	0,252035578
chr4	89250000	89500000	0,263871575
chr4	89850000	90100000	0,323621258
chr4	90500000	90750000	0,329225336
chr4	91750000	92000000	0,585172895
chr4	93150000	93400000	0,608491411
chr4	94200000	94450000	0,518528638
chr4	95250000	95500000	0,230693374
chr4	96850000	97100000	0,721216681
chr4	97650000	97900000	0,746471971
chr4	99750000	100000000	0,382426496
chr4	100600000	100850000	0,342761263
chr4	102200000	102450000	0,429316504
chr4	103550000	103800000	0,227985562
chr4	106700000	106950000	0,216251845
chr4	108650000	108900000	0,360795287
chr4	109700000	109950000	0,284769358
chr4	113050000	113300000	0,322407006
chr4	114100000	114350000	0,429101899
chr4	114650000	114900000	0,422344157
chr4	117250000	117500000	0,840151423
chr4	119750000	120000000	0,329675083

chr4	61700000	61950000	0,825052694
chr4	62500000	62750000	0,816953925
chr4	63100000	63350000	0,829071011
chr4	66500000	66750000	0,526007681
chr4	67300000	67550000	0,410040109
chr4	68350000	68600000	0,40407182
chr4	69050000	69300000	0,400272206
chr4	71550000	71800000	0,331331562
chr4	74900000	75150000	0,266983286
chr4	76750000	77000000	0,244356458
chr4	77450000	77700000	0,193122203
chr4	78800000	79050000	0,258140264
chr4	79350000	79600000	0,225521383
chr4	80500000	80750000	0,537621656
chr4	82050000	82300000	0,684289056
chr4	83950000	84200000	0,143655201
chr4	85450000	85700000	0,532288181
chr4	88000000	88250000	0,245977902
chr4	89200000	89450000	0,256185714
chr4	89900000	90150000	0,303258054
chr4	90500000	90750000	0,280249627
chr4	91750000	92000000	0,567027394
chr4	93200000	93450000	0,59144399
chr4	94100000	94350000	0,538732373
chr4	95250000	95500000	0,244611626
chr4	96900000	97150000	0,705206034
chr4	97700000	97950000	0,729135109

chr4	120450000	120700000	0,347303932
chr4	122600000	122850000	0,323658626
chr4	123600000	123850000	0,186306726
chr4	124900000	125150000	0,465502497
chr4	126350000	126600000	0,520273608
chr4	129150000	129400000	0,192582554
chr4	133300000	133550000	0,747879756
chr4	134550000	134800000	0,684347994
chr4	134950000	135200000	0,685389699
chr4	136100000	136350000	0,740247322
chr4	137200000	137450000	0,716384121
chr4	139000000	139250000	0,480823739
chr4	140100000	140350000	0,192684335
chr4	141550000	141800000	0,307570625
chr4	144050000	144300000	0,374376048
chr4	146600000	146850000	0,360513958
chr4	148450000	148700000	0,290014573
chr4	150850000	151100000	0,251100889
chr4	152050000	152300000	0,213871722
chr4	152950000	153200000	0,273868635
chr4	154100000	154350000	0,269157495
chr4	155250000	155500000	0,733575173
chr4	157700000	157950000	0,518972386
chr4	159750000	160000000	0,241601011
chr4	162700000	162950000	0,501111502
chr4	164250000	164500000	0,599015195
chr4	166500000	166750000	0,292903711

chr4	99150000	99400000	0,462738069
chr4	99750000	100000000	0,384918498
chr4	100600000	100850000	0,389260308
chr4	102200000	102450000	0,426973799
chr4	103550000	103800000	0,238988967
chr4	106700000	106950000	0,20290712
chr4	108650000	108900000	0,346940163
chr4	109750000	110000000	0,248799949
chr4	113050000	113300000	0,305836762
chr4	114650000	114900000	0,437258116
chr4	116650000	116900000	0,825860545
chr4	117350000	117600000	0,827532394
chr4	119750000	120000000	0,317229539
chr4	122600000	122850000	0,320168072
chr4	123600000	123850000	0,196319177
chr4	126300000	126550000	0,542913908
chr4	129200000	129450000	0,205743147
chr4	133250000	133500000	0,734195955
chr4	134450000	134700000	0,665971139
chr4	135000000	135250000	0,663743832
chr4	139050000	139300000	0,469346374
chr4	140100000	140350000	0,209666063
chr4	141550000	141800000	0,304531821
chr4	143400000	143650000	0,379867085
chr4	144100000	144350000	0,361203015
chr4	146100000	146350000	0,376909888
chr4	146600000	146850000	0,361768132

chr4	169400000	169650000	0,144198143
chr4	174100000	174350000	0,2946668
chr4	175050000	175300000	0,398446287
chr4	177150000	177400000	0,409208989
chr4	178050000	178300000	0,475252449
chr4	184200000	184450000	0,149721739
chr4	185100000	185350000	0,220460965
chr4	186600000	186850000	0,19729144
chr4	187550000	187800000	0,357150964
chr4	188900000	189150000	0,360478699
chr4	190650000	190900000	0,693864189
chr5	0	150000	0,214933623
chr5	750000	1000000	0,262289554
chr5	1300000	1550000	0,33279409
chr5	3200000	3450000	0,821719365
chr5	5200000	5450000	0,424694228
chr5	6550000	6800000	0,403417944
chr5	7700000	7950000	0,74103126
chr5	10300000	10550000	0,158858153
chr5	12050000	12300000	0,786482145
chr5	13950000	14200000	0,249708101
chr5	14750000	15000000	0,249913875
chr5	17050000	17300000	0,230735165
chr5	21450000	21700000	0,706452658
chr5	23200000	23450000	0,872469301
chr5	24450000	24700000	0,862324011
chr5	25400000	25650000	0,849344714

chr4	148450000	148700000	0,297427499
chr4	150850000	151100000	0,268445732
chr4	152050000	152300000	0,242895534
chr4	153050000	153300000	0,309463786
chr4	154000000	154250000	0,268484602
chr4	155250000	155500000	0,718506902
chr4	157650000	157900000	0,566502011
chr4	159750000	160000000	0,250129241
chr4	162700000	162950000	0,531270798
chr4	164200000	164450000	0,576738575
chr4	166500000	166750000	0,268379602
chr4	169350000	169600000	0,144923411
chr4	174100000	174350000	0,316285981
chr4	175000000	175250000	0,412388201
chr4	177100000	177350000	0,43065612
chr4	178100000	178350000	0,458958244
chr4	179800000	180050000	0,824872996
chr4	184250000	184500000	0,151047203
chr4	185050000	185300000	0,240109789
chr4	186650000	186900000	0,176215844
chr4	187500000	187750000	0,326095098
chr4	188850000	189100000	0,326504909
chr4	190600000	190850000	0,667194797
chr5	0	150000	0,229809525
chr5	800000	1050000	0,257647698
chr5	3200000	3450000	0,796900405
chr5	5200000	5450000	0,426158706

chr5	27250000	27500000	0,705209034
chr5	28300000	28550000	0,694955286
chr5	29150000	29400000	0,690290315
chr5	30000000	30250000	0,531980885
chr5	32100000	32350000	0,199404554
chr5	34550000	34800000	0,299147822
chr5	36400000	36650000	0,188849096
chr5	37150000	37400000	0,222013395
chr5	38500000	38750000	0,170495755
chr5	40600000	40850000	0,350667999
chr5	41750000	42000000	0,550941565
chr5	43300000	43550000	0,294240182
chr5	45950000	46200000	0,795583072
chr5	49800000	50050000	0,401500598
chr5	52050000	52300000	0,284243996
chr5	53700000	53950000	0,336008889
chr5	54650000	54900000	0,231773583
chr5	55250000	55500000	0,267234843
chr5	56150000	56400000	0,239322481
chr5	57250000	57500000	0,294226654
chr5	58050000	58300000	0,249164228
chr5	59000000	59250000	0,243751746
chr5	60500000	60750000	0,321336537
chr5	61500000	61750000	0,359204551
chr5	65100000	65350000	0,161544859
chr5	66250000	66500000	0,365004954
chr5	68450000	68700000	0,219214237

chr5	6550000	6800000	0,422998094
chr5	7750000	8000000	0,71091297
chr5	10300000	10550000	0,155823429
chr5	12050000	12300000	0,757682141
chr5	12900000	13150000	0,707987169
chr5	14000000	14250000	0,297197019
chr5	14750000	15000000	0,26201424
chr5	17100000	17350000	0,231171954
chr5	19800000	20050000	0,851274668
chr5	21500000	21750000	0,663946947
chr5	24400000	24650000	0,836339572
chr5	25400000	25650000	0,832214115
chr5	27350000	27600000	0,676972178
chr5	28400000	28650000	0,674931208
chr5	29150000	29400000	0,655261977
chr5	30000000	30250000	0,559708772
chr5	32100000	32350000	0,216958148
chr5	34550000	34800000	0,301058892
chr5	36350000	36600000	0,188101869
chr5	37150000	37400000	0,234378105
chr5	38500000	38750000	0,15980467
chr5	40650000	40900000	0,334936445
chr5	41750000	42000000	0,551270539
chr5	43300000	43550000	0,289205438
chr5	45450000	45700000	0,763813949
chr5	45950000	46200000	0,763591101
chr5	49800000	50050000	0,383830377

chr5	69650000	69900000	0,294513088
chr5	70150000	70400000	0,296142541
chr5	72100000	72350000	0,277200324
chr5	72750000	73000000	0,248705395
chr5	74000000	74250000	0,319074685
chr5	76350000	76600000	0,269809997
chr5	77900000	78150000	0,274961486
chr5	79650000	79900000	0,302554095
chr5	80400000	80650000	0,359682099
chr5	80950000	81200000	0,382799582
chr5	82200000	82450000	0,592213251
chr5	83600000	83850000	0,644573715
chr5	86150000	86400000	0,406813865
chr5	87700000	87950000	0,658112636
chr5	90350000	90600000	0,222666987
chr5	92550000	92800000	0,346072516
chr5	93850000	94100000	0,193529066
chr5	95050000	95300000	0,172388762
chr5	95750000	96000000	0,183043551
chr5	98200000	98450000	0,376783539
chr5	99900000	100150000	0,718186926
chr5	100450000	100700000	0,701087648
chr5	102050000	102300000	0,388343256
chr5	103750000	104000000	0,616878393
chr5	106600000	106850000	0,455237339
chr5	107950000	108200000	0,375640488
chr5	108550000	108800000	0,404658533

chr5	52050000	52300000	0,275788723
chr5	53700000	53950000	0,3338374
chr5	54600000	54850000	0,242653296
chr5	56150000	56400000	0,260333292
chr5	57250000	57500000	0,283535573
chr5	58050000	58300000	0,235621203
chr5	59000000	59250000	0,225345701
chr5	60500000	60750000	0,292418453
chr5	61450000	61700000	0,34555374
chr5	65050000	65300000	0,163968565
chr5	66250000	66500000	0,368305529
chr5	68450000	68700000	0,234407575
chr5	69250000	69500000	0,280853626
chr5	70050000	70300000	0,275306391
chr5	72150000	72400000	0,279791485
chr5	72750000	73000000	0,248008457
chr5	74050000	74300000	0,307250969
chr5	76350000	76600000	0,27069656
chr5	77900000	78150000	0,265434294
chr5	79600000	79850000	0,285512893
chr5	80400000	80650000	0,338506191
chr5	80950000	81200000	0,363679924
chr5	82150000	82400000	0,599655219
chr5	83550000	83800000	0,661331488
chr5	86150000	86400000	0,384121005
chr5	87500000	87750000	0,642899372
chr5	90350000	90600000	0,234911932

chr5	112150000	112400000	0,25384597
chr5	113400000	113650000	0,61100582
chr5	114550000	114800000	0,400964514
chr5	116700000	116950000	0,74866401
chr5	118500000	118750000	0,271934534
chr5	119700000	119950000	0,754811175
chr5	121950000	122200000	0,202963821
chr5	123850000	124100000	0,435487126
chr5	125700000	125950000	0,207715942
chr5	127100000	127350000	0,383446878
chr5	127700000	127950000	0,444547484
chr5	129150000	129400000	0,759232296
chr5	130450000	130700000	0,26331405
chr5	131700000	131950000	0,15948355
chr5	133700000	133950000	0,128425796
chr5	135200000	135450000	0,302855776
chr5	137700000	137950000	0,154117338
chr5	138650000	138900000	0,151410056
chr5	139550000	139800000	0,165716579
chr5	141200000	141450000	0,242058681
chr5	142650000	142900000	0,206109001
chr5	145450000	145700000	0,233263906
chr5	147350000	147600000	0,195103315
chr5	148250000	148500000	0,20110349
chr5	149150000	149400000	0,160685676
chr5	149750000	150000000	0,141802731
chr5	150900000	151150000	0,355765415

chr5	92550000	92800000	0,327438516
chr5	93850000	94100000	0,178095622
chr5	95000000	95250000	0,1823325
chr5	95750000	96000000	0,192148306
chr5	98200000	98450000	0,340402771
chr5	99900000	100150000	0,680662702
chr5	100500000	100750000	0,67062514
chr5	102000000	102250000	0,399881173
chr5	103750000	104000000	0,600559298
chr5	106600000	106850000	0,446322031
chr5	107950000	108200000	0,356433279
chr5	108550000	108800000	0,38523125
chr5	111000000	111250000	0,497693961
chr5	112150000	112400000	0,250519828
chr5	114550000	114800000	0,396487376
chr5	116650000	116900000	0,72804415
chr5	118500000	118750000	0,262536275
chr5	121950000	122200000	0,184161746
chr5	123850000	124100000	0,458737377
chr5	125650000	125900000	0,220684599
chr5	127150000	127400000	0,41059441
chr5	129100000	129350000	0,741305762
chr5	130450000	130700000	0,266440555
chr5	131700000	131950000	0,161716219
chr5	133750000	134000000	0,130423821
chr5	135200000	135450000	0,315036037
chr5	137200000	137450000	0,196366215

chr5	153950000	154200000	0,232799039
chr5	157050000	157300000	0,447135772
chr5	158600000	158850000	0,251620595
chr5	159500000	159750000	0,238229227
chr5	162800000	163050000	0,472429331
chr5	163900000	164150000	0,855509451
chr5	165050000	165300000	0,860583372
chr5	167600000	167850000	0,192620097
chr5	169850000	170100000	0,339539126
chr5	170700000	170950000	0,260762988
chr5	172100000	172350000	0,143148763
chr5	173050000	173300000	0,310710292
chr5	175850000	176100000	0,263179339
chr5	176600000	176850000	0,150955029
chr5	177400000	177650000	0,238067921
chr5	179050000	179300000	0,181395365
chr5	180500000	180750000	0,320498518
chr6	1300000	1550000	0,241038421
chr6	2750000	3000000	0,154065596
chr6	3700000	3950000	0,18637875
chr6	4850000	5100000	0,359734502
chr6	5800000	6050000	0,548206819
chr6	6750000	7000000	0,213804545
chr6	7450000	7700000	0,16867477
chr6	10400000	10650000	0,179639129
chr6	11150000	11400000	0,16928942
chr6	12100000	12350000	0,304790672

chr5	137750000	138000000	0,165458812
chr5	138650000	138900000	0,155779119
chr5	139550000	139800000	0,180724132
chr5	141200000	141450000	0,248411648
chr5	142650000	142900000	0,225048748
chr5	145450000	145700000	0,23493926
chr5	147350000	147600000	0,177936453
chr5	148250000	148500000	0,199450855
chr5	149150000	149400000	0,154590137
chr5	149750000	150000000	0,146285616
chr5	150900000	151150000	0,34344862
chr5	153950000	154200000	0,244009605
chr5	157050000	157300000	0,45196678
chr5	158600000	158850000	0,233271658
chr5	159500000	159750000	0,233530312
chr5	162800000	163050000	0,452373624
chr5	164350000	164600000	0,842115459
chr5	165050000	165300000	0,845855097
chr5	167600000	167850000	0,196179911
chr5	170100000	170350000	0,336750116
chr5	170750000	171000000	0,257232122
chr5	172100000	172350000	0,143722455
chr5	173100000	173350000	0,29037521
chr5	175900000	176150000	0,263179808
chr5	176600000	176850000	0,154343556
chr5	177450000	177700000	0,235535187
chr5	179050000	179300000	0,184033169

chr6	13450000	13700000	0,257854214
chr6	14750000	15000000	0,391814466
chr6	16700000	16950000	0,327532732
chr6	17800000	18050000	0,219837146
chr6	20250000	20500000	0,357296899
chr6	21350000	21600000	0,385876086
chr6	24850000	25100000	0,206730451
chr6	26350000	26600000	0,311874118
chr6	27300000	27550000	0,361660449
chr6	28250000	28500000	0,319674007
chr6	28700000	28950000	0,318238924
chr6	29900000	30150000	0,252422429
chr6	30700000	30950000	0,131551634
chr6	31550000	31800000	0,12919856
chr6	33300000	33550000	0,154079515
chr6	34500000	34750000	0,147219601
chr6	35150000	35400000	0,159534432
chr6	36500000	36750000	0,135792324
chr6	36950000	37200000	0,133461834
chr6	38400000	38650000	0,379054472
chr6	40900000	41150000	0,329290624
chr6	41850000	42100000	0,148213767
chr6	42750000	43000000	0,140893278
chr6	43450000	43700000	0,154246127
chr6	43850000	44100000	0,155310826
chr6	45350000	45600000	0,423310058
chr6	47000000	47250000	0,270029887

chr5	180800000	180915260	0,306577569
chr6	1400000	1650000	0,252026242
chr6	2550000	2800000	0,15402515
chr6	3700000	3950000	0,208903315
chr6	4850000	5100000	0,331766011
chr6	5800000	6050000	0,529106084
chr6	6750000	7000000	0,19523859
chr6	7450000	7700000	0,171323744
chr6	10350000	10600000	0,169917843
chr6	11150000	11400000	0,201263306
chr6	12150000	12400000	0,292501498
chr6	13500000	13750000	0,267931795
chr6	14900000	15150000	0,38610618
chr6	16700000	16950000	0,31189532
chr6	17800000	18050000	0,229348516
chr6	20250000	20500000	0,324238033
chr6	21300000	21550000	0,374543435
chr6	25000000	25250000	0,225880373
chr6	26350000	26600000	0,312957659
chr6	27300000	27550000	0,340055046
chr6	28250000	28500000	0,298904673
chr6	28650000	28900000	0,3038729
chr6	29850000	30100000	0,260303543
chr6	30650000	30900000	0,141199959
chr6	31600000	31850000	0,135695618
chr6	33350000	33600000	0,154161875
chr6	34550000	34800000	0,153414501

chr6	48700000	48950000	0,549020242
chr6	49450000	49700000	0,443099713
chr6	52250000	52500000	0,312064331
chr6	53350000	53600000	0,244475638
chr6	54600000	54850000	0,425681558
chr6	56150000	56400000	0,308335506
chr6	56800000	57050000	0,229902492
chr6	58150000	58400000	0,641527242
chr6	62200000	62450000	0,821091168
chr6	64150000	64400000	0,228330139
chr6	68000000	68250000	0,842990879
chr6	68750000	69000000	0,839950404
chr6	71150000	71400000	0,306992308
chr6	72000000	72250000	0,308116916
chr6	74150000	74400000	0,162808288
chr6	76150000	76400000	0,250610914
chr6	77900000	78150000	0,688136242
chr6	80000000	80250000	0,284253793
chr6	82450000	82700000	0,343368043
chr6	83450000	83700000	0,451664666
chr6	84100000	84350000	0,399796817
chr6	86050000	86300000	0,231387338
chr6	88250000	88500000	0,330798572
chr6	89800000	90050000	0,228998844
chr6	90850000	91100000	0,400253551
chr6	92950000	93200000	0,590521203
chr6	95250000	95500000	0,812411963

chr6	35200000	35450000	0,170039422
chr6	36500000	36750000	0,135805641
chr6	36900000	37150000	0,13135053
chr6	38350000	38600000	0,364125474
chr6	40850000	41100000	0,304028014
chr6	41850000	42100000	0,154318025
chr6	42800000	43050000	0,149101139
chr6	43450000	43700000	0,152630281
chr6	43850000	44100000	0,154836706
chr6	45300000	45550000	0,410263306
chr6	47000000	47250000	0,303556238
chr6	48750000	49000000	0,54633335
chr6	49400000	49650000	0,443964996
chr6	52250000	52500000	0,29231023
chr6	53350000	53600000	0,24720021
chr6	54600000	54850000	0,4099953
chr6	56750000	57000000	0,24345288
chr6	58150000	58400000	0,628699819
chr6	62250000	62500000	0,784005209
chr6	64150000	64400000	0,210465656
chr6	67100000	67350000	0,819050841
chr6	67600000	67850000	0,819041366
chr6	68300000	68550000	0,826156454
chr6	71150000	71400000	0,315161015
chr6	72000000	72250000	0,313524549
chr6	74150000	74400000	0,171688091
chr6	75350000	75600000	0,328436891

chr6	95950000	96200000	0,761138355
chr6	97550000	97800000	0,559684424
chr6	98200000	98450000	0,643378449
chr6	99750000	100000000	0,3566988
chr6	100600000	100850000	0,466155781
chr6	101250000	101500000	0,530966125
chr6	101950000	102200000	0,636269214
chr6	105800000	106050000	0,304620322
chr6	107050000	107300000	0,200428432
chr6	108350000	108600000	0,261420598
chr6	109000000	109250000	0,327313891
chr6	109500000	109750000	0,333275256
chr6	111200000	111450000	0,234560956
chr6	112000000	112250000	0,203222506
chr6	113750000	114000000	0,373356374
chr6	116750000	117000000	0,324483354
chr6	117650000	117900000	0,28555464
chr6	118950000	119200000	0,397799291
chr6	121600000	121850000	0,497663492
chr6	122600000	122850000	0,632090364
chr6	125400000	125650000	0,300409004
chr6	126100000	126350000	0,191642292
chr6	127550000	127800000	0,425380861
chr6	128850000	129100000	0,405939981
chr6	129850000	130100000	0,316321306
chr6	131350000	131600000	0,459251146
chr6	134550000	134800000	0,147431476

chr6	76100000	76350000	0,26718062
chr6	77900000	78150000	0,693062733
chr6	80000000	80250000	0,262522809
chr6	82450000	82700000	0,328019423
chr6	83450000	83700000	0,447938909
chr6	84050000	84300000	0,369414045
chr6	86100000	86350000	0,253156513
chr6	88200000	88450000	0,308779503
chr6	89850000	90100000	0,244470666
chr6	92950000	93200000	0,601787994
chr6	95950000	96200000	0,748359052
chr6	97550000	97800000	0,540632153
chr6	99750000	100000000	0,356954457
chr6	100600000	100850000	0,488634056
chr6	101200000	101450000	0,521825127
chr6	101950000	102200000	0,628014538
chr6	105750000	106000000	0,312238275
chr6	107100000	107350000	0,211315282
chr6	108350000	108600000	0,267321872
chr6	109050000	109300000	0,311465244
chr6	111150000	111400000	0,25214182
chr6	111900000	112150000	0,223604898
chr6	113850000	114100000	0,368874111
chr6	116750000	117000000	0,317466205
chr6	117650000	117900000	0,270699979
chr6	118950000	119200000	0,404955824
chr6	121550000	121800000	0,472973306

chr6	135850000	136100000	0,232839499
chr6	136500000	136750000	0,20051295
chr6	138100000	138350000	0,220040188
chr6	139700000	139950000	0,311105868
chr6	142500000	142750000	0,284273325
chr6	144350000	144600000	0,223133596
chr6	145000000	145250000	0,37531635
chr6	145950000	146200000	0,427146762
chr6	147200000	147450000	0,402224746
chr6	150000000	150250000	0,218660062
chr6	151250000	151500000	0,209032618
chr6	152700000	152950000	0,26932531
chr6	154900000	155150000	0,249472453
chr6	155250000	155500000	0,242714356
chr6	157600000	157850000	0,265331878
chr6	158250000	158500000	0,195686182
chr6	159050000	159300000	0,168960001
chr6	160200000	160450000	0,300739781
chr6	162300000	162550000	0,76437017
chr6	163700000	163950000	0,454186162
chr6	166300000	166550000	0,232880412
chr6	168350000	168600000	0,519787746
chr6	169500000	169750000	0,382987401
chr6	169950000	170200000	0,353675861
chr7	850000	1100000	0,190192077
chr7	1400000	1650000	0,173897791
chr7	2350000	2600000	0,171605939

chr6	122600000	122850000	0,593190402
chr6	125450000	125700000	0,285664743
chr6	126150000	126400000	0,18335552
chr6	127600000	127850000	0,429570572
chr6	128850000	129100000	0,401265168
chr6	129850000	130100000	0,288005309
chr6	131400000	131650000	0,488253508
chr6	134550000	134800000	0,140989607
chr6	135900000	136150000	0,238852854
chr6	136450000	136700000	0,198953439
chr6	138050000	138300000	0,224062382
chr6	139700000	139950000	0,326582589
chr6	141550000	141800000	0,567801201
chr6	142500000	142750000	0,283166652
chr6	144350000	144600000	0,246826557
chr6	145950000	146200000	0,400403484
chr6	147200000	147450000	0,40233715
chr6	149950000	150200000	0,224869748
chr6	151250000	151500000	0,231415145
chr6	151900000	152150000	0,256202341
chr6	152600000	152850000	0,268885645
chr6	154850000	155100000	0,256752539
chr6	158200000	158450000	0,197931675
chr6	159100000	159350000	0,176743141
chr6	160250000	160500000	0,297235804
chr6	162200000	162450000	0,760081705
chr6	163700000	163950000	0,459817492

chr7	4650000	4900000	0,302626271
chr7	5500000	5750000	0,131554043
chr7	6150000	6400000	0,129901883
chr7	7800000	8050000	0,235280825
chr7	12100000	12350000	0,304187293
chr7	15000000	15250000	0,568377548
chr7	16800000	17050000	0,29729483
chr7	17250000	17500000	0,317504452
chr7	18350000	18600000	0,371982766
chr7	20300000	20550000	0,444356127
chr7	23250000	23500000	0,162697366
chr7	24900000	25150000	0,214309087
chr7	25800000	26050000	0,237848786
chr7	26050000	26300000	0,241222153
chr7	27050000	27300000	0,183572321
chr7	27550000	27800000	0,195650585
chr7	30250000	30500000	0,269183379
chr7	32850000	33100000	0,225124376
chr7	33650000	33900000	0,369808553
chr7	36250000	36500000	0,26625304
chr7	37750000	38000000	0,586389034
chr7	38100000	38350000	0,579269875
chr7	39700000	39950000	0,272268899
chr7	40900000	41150000	0,319089059
chr7	43850000	44100000	0,160325801
chr7	44650000	44900000	0,135652166
chr7	46000000	46250000	0,361174444

chr6	166250000	166500000	0,19451176
chr6	170000000	170250000	0,325626595
chr7	850000	1100000	0,190994907
chr7	1400000	1650000	0,175846011
chr7	2400000	2650000	0,17683914
chr7	4650000	4900000	0,288101527
chr7	5450000	5700000	0,13997332
chr7	6150000	6400000	0,136241526
chr7	7800000	8050000	0,251933399
chr7	12150000	12400000	0,310736207
chr7	15050000	15300000	0,649797542
chr7	16800000	17050000	0,290922598
chr7	18300000	18550000	0,381878629
chr7	20300000	20550000	0,438058545
chr7	23250000	23500000	0,164695264
chr7	24900000	25150000	0,220867332
chr7	25800000	26050000	0,218797493
chr7	27050000	27300000	0,187761988
chr7	27600000	27850000	0,206968182
chr7	30200000	30450000	0,269606271
chr7	32850000	33100000	0,224437173
chr7	33750000	34000000	0,378893496
chr7	36300000	36550000	0,255676215
chr7	38150000	38400000	0,561726557
chr7	39700000	39950000	0,293659549
chr7	40900000	41150000	0,350271374
chr7	43850000	44100000	0,162318597

chr7	47600000	47850000	0,160909065
chr7	51250000	51500000	0,219086625
chr7	54950000	55200000	0,161833568
chr7	55850000	56100000	0,343912933
chr7	61450000	61700000	0,750383245
chr7	62050000	62300000	0,757319148
chr7	64500000	64750000	0,389679301
chr7	65500000	65750000	0,193961766
chr7	66050000	66300000	0,157622342
chr7	69200000	69450000	0,832254927
chr7	72950000	73200000	0,14761281
chr7	73850000	74100000	0,119004707
chr7	74750000	75000000	0,176402911
chr7	75850000	76100000	0,14153122
chr7	76950000	77200000	0,296592793
chr7	79000000	79250000	0,456561308
chr7	80250000	80500000	0,227508614
chr7	82050000	82300000	0,456480141
chr7	83900000	84150000	0,395675344
chr7	85150000	85400000	0,485075263
chr7	87150000	87400000	0,321043952
chr7	90050000	90300000	0,30821746
chr7	90850000	91100000	0,460214251
chr7	92050000	92300000	0,213552991
chr7	92400000	92650000	0,226312644
chr7	93550000	93800000	0,330581438
chr7	95050000	95300000	0,274590569

chr7	44700000	44950000	0,136711211
chr7	45950000	46200000	0,364454594
chr7	47600000	47850000	0,164622506
chr7	51250000	51500000	0,207761545
chr7	54950000	55200000	0,163853354
chr7	55800000	56050000	0,332770774
chr7	61850000	62100000	0,712270705
chr7	64500000	64750000	0,386697531
chr7	65450000	65700000	0,21755803
chr7	66100000	66350000	0,168434372
chr7	69200000	69450000	0,804515053
chr7	70100000	70350000	0,817837413
chr7	72950000	73200000	0,155902501
chr7	73800000	74050000	0,123703364
chr7	74500000	74750000	0,136546374
chr7	75850000	76100000	0,144537979
chr7	76950000	77200000	0,286358128
chr7	79000000	79250000	0,522117479
chr7	80250000	80500000	0,212161231
chr7	82050000	82300000	0,445195529
chr7	84000000	84250000	0,397119269
chr7	85100000	85350000	0,543940596
chr7	87350000	87600000	0,360911196
chr7	90050000	90300000	0,322748388
chr7	90850000	91100000	0,451248262
chr7	92050000	92300000	0,20487033
chr7	93550000	93800000	0,317886184

chr7	96550000	96800000	0,62840224
chr7	97750000	98000000	0,203752414
chr7	98850000	99100000	0,191864539
chr7	99900000	100150000	0,128303636
chr7	101100000	101350000	0,159807275
chr7	102000000	102250000	0,167632369
chr7	105000000	105250000	0,188822588
chr7	106350000	106600000	0,358694102
chr7	107500000	107750000	0,231677739
chr7	110050000	110300000	0,503315848
chr7	111150000	111400000	0,280846019
chr7	111900000	112150000	0,388850385
chr7	113600000	113850000	0,499349788
chr7	114350000	114600000	0,417907962
chr7	116000000	116250000	0,124071824
chr7	116950000	117200000	0,172039657
chr7	120550000	120800000	0,369701113
chr7	123250000	123500000	0,459543448
chr7	124100000	124350000	0,380787588
chr7	125400000	125650000	0,511098238
chr7	128150000	128400000	0,141584574
chr7	129550000	129800000	0,125499991
chr7	130750000	131000000	0,159080595
chr7	132650000	132900000	0,43263933
chr7	134300000	134550000	0,311763182
chr7	135100000	135350000	0,312461405
chr7	137550000	137800000	0,42406815

chr7	95050000	95300000	0,255757161
chr7	96550000	96800000	0,588572533
chr7	97750000	98000000	0,199822965
chr7	98850000	99100000	0,192158464
chr7	99850000	100100000	0,13249474
chr7	101100000	101350000	0,17438698
chr7	101950000	102200000	0,161075502
chr7	105000000	105250000	0,200131651
chr7	106450000	106700000	0,361018357
chr7	107500000	107750000	0,229875801
chr7	110050000	110300000	0,528609413
chr7	111150000	111400000	0,281617855
chr7	111900000	112150000	0,366821145
chr7	113600000	113850000	0,473431251
chr7	114300000	114550000	0,411826432
chr7	116000000	116250000	0,1264356
chr7	116900000	117150000	0,161712901
chr7	120550000	120800000	0,381142184
chr7	123300000	123550000	0,441145173
chr7	124150000	124400000	0,348990102
chr7	125400000	125650000	0,499179339
chr7	128200000	128450000	0,143974272
chr7	129600000	129850000	0,127670726
chr7	130750000	131000000	0,172491144
chr7	132700000	132950000	0,441638957
chr7	134300000	134550000	0,320616375
chr7	135050000	135300000	0,303596355

chr7	138750000	139000000	0,195072769
chr7	140000000	140250000	0,26342844
chr7	141250000	141500000	0,508333953
chr7	142100000	142350000	0,44440382
chr7	142900000	143150000	0,397994628
chr7	143850000	144100000	0,424445682
chr7	145450000	145700000	0,832789199
chr7	148650000	148900000	0,182884527
chr7	149900000	150150000	0,323902518
chr7	151050000	151300000	0,143055513
chr7	152100000	152350000	0,268279684
chr7	153400000	153650000	0,792393265
chr7	155000000	155250000	0,376217636
chr7	156900000	157150000	0,210472081
chr7	158500000	158750000	0,241668735
chr8	0	150000	0,480379521
chr8	1700000	1950000	0,366100274
chr8	2750000	3000000	0,691291884
chr8	4550000	4800000	0,815214141
chr8	6450000	6700000	0,441600781
chr8	8250000	8500000	0,174053873
chr8	10400000	10650000	0,481576663
chr8	11600000	11850000	0,33179105
chr8	12900000	13150000	0,205523594
chr8	16450000	16700000	0,404408404
chr8	17350000	17600000	0,205483044
chr8	19050000	19300000	0,217079491

chr7	137550000	137800000	0,415280093
chr7	138750000	139000000	0,20576338
chr7	140050000	140300000	0,272110836
chr7	141300000	141550000	0,493959731
chr7	142100000	142350000	0,423979363
chr7	142850000	143100000	0,394662038
chr7	143900000	144150000	0,47085232
chr7	145500000	145750000	0,79292214
chr7	148650000	148900000	0,191139397
chr7	149900000	150150000	0,322327139
chr7	151050000	151300000	0,146243054
chr7	152100000	152350000	0,273632177
chr7	153650000	153900000	0,777634584
chr7	155000000	155250000	0,361567397
chr7	156900000	157150000	0,219609165
chr7	158550000	158800000	0,234677371
chr8	0	150000	0,456363515
chr8	1700000	1950000	0,341983028
chr8	2800000	3050000	0,680827241
chr8	4550000	4800000	0,821611516
chr8	6450000	6700000	0,436464399
chr8	8300000	8550000	0,177651213
chr8	10400000	10650000	0,463396831
chr8	11550000	11800000	0,329264742
chr8	12900000	13150000	0,205510588
chr8	16450000	16700000	0,383046436
chr8	17300000	17550000	0,209118908

chr8	22100000	22350000	0,127618474
chr8	22850000	23100000	0,143835921
chr8	24950000	25200000	0,245807416
chr8	26150000	26400000	0,265269252
chr8	27550000	27800000	0,147844549
chr8	28150000	28400000	0,178840043
chr8	29150000	29400000	0,224932042
chr8	29950000	30200000	0,186617793
chr8	30350000	30600000	0,195915281
chr8	32100000	32350000	0,362823191
chr8	33200000	33450000	0,318830022
chr8	35350000	35600000	0,78973742
chr8	36800000	37050000	0,31347737
chr8	37900000	38150000	0,181481656
chr8	38550000	38800000	0,181354394
chr8	39900000	40150000	0,233466469
chr8	41500000	41750000	0,281077446
chr8	42300000	42550000	0,21632254
chr8	48700000	48950000	0,287802209
chr8	52600000	52850000	0,463552989
chr8	53500000	53750000	0,565837021
chr8	54750000	55000000	0,260035458
chr8	56700000	56950000	0,348233018
chr8	57700000	57950000	0,63313521
chr8	59500000	59750000	0,403730062
chr8	60400000	60650000	0,536467145
chr8	61450000	61700000	0,331749539

chr8	19400000	19650000	0,22078343
chr8	22100000	22350000	0,129917559
chr8	22850000	23100000	0,147691215
chr8	24950000	25200000	0,23971811
chr8	26150000	26400000	0,271435446
chr8	27550000	27800000	0,144282406
chr8	28200000	28450000	0,18217914
chr8	29100000	29350000	0,220532527
chr8	29850000	30100000	0,229209349
chr8	30400000	30650000	0,210973121
chr8	32100000	32350000	0,371650756
chr8	33200000	33450000	0,305343672
chr8	36850000	37100000	0,275783056
chr8	37950000	38200000	0,188912912
chr8	38550000	38800000	0,177412963
chr8	39900000	40150000	0,217792124
chr8	41500000	41750000	0,269717976
chr8	42100000	42350000	0,247019938
chr8	47100000	47350000	0,683934441
chr8	48700000	48950000	0,276696192
chr8	52650000	52900000	0,456201383
chr8	53450000	53700000	0,569822198
chr8	54800000	55050000	0,257016363
chr8	56700000	56950000	0,343611555
chr8	57700000	57950000	0,648537871
chr8	59500000	59750000	0,39318614
chr8	61450000	61700000	0,316838812

chr8	62550000	62800000	0,280151822
chr8	63950000	64200000	0,397946197
chr8	65450000	65700000	0,759350068
chr8	67350000	67600000	0,162001924
chr8	70450000	70700000	0,412365482
chr8	71300000	71550000	0,333897489
chr8	72750000	73000000	0,624271053
chr8	74250000	74500000	0,179007047
chr8	75550000	75800000	0,323183448
chr8	77350000	77600000	0,480117559
chr8	79400000	79650000	0,642033398
chr8	81000000	81250000	0,230551015
chr8	81600000	81850000	0,289538212
chr8	82450000	82700000	0,465547369
chr8	84050000	84300000	0,847283807
chr8	86000000	86250000	0,539338443
chr8	87150000	87400000	0,476896261
chr8	89900000	90150000	0,399728332
chr8	90750000	91000000	0,321650554
chr8	91800000	92050000	0,472867009
chr8	92950000	93200000	0,783263719
chr8	94800000	95050000	0,293799377
chr8	95900000	96150000	0,236469542
chr8	97150000	97400000	0,597265828
chr8	98750000	99000000	0,179817152
chr8	99850000	100100000	0,244064686
chr8	101850000	102100000	0,142674126

chr8	62550000	62800000	0,259251233
chr8	63950000	64200000	0,407215792
chr8	67350000	67600000	0,171968378
chr8	70500000	70750000	0,421048682
chr8	71300000	71550000	0,336663407
chr8	72750000	73000000	0,637092412
chr8	74250000	74500000	0,178182134
chr8	75500000	75750000	0,266967663
chr8	77350000	77600000	0,543232647
chr8	79450000	79700000	0,606383839
chr8	81000000	81250000	0,238728243
chr8	81550000	81800000	0,29205375
chr8	82400000	82650000	0,447062138
chr8	83900000	84150000	0,835375896
chr8	84700000	84950000	0,830833315
chr8	86000000	86250000	0,506658993
chr8	87200000	87450000	0,518295287
chr8	89950000	90200000	0,392838165
chr8	90750000	91000000	0,314188904
chr8	91800000	92050000	0,465404249
chr8	94800000	95050000	0,310570585
chr8	95800000	96050000	0,248250277
chr8	97100000	97350000	0,540154915
chr8	98700000	98950000	0,192669935
chr8	99800000	100050000	0,247342742
chr8	102200000	102450000	0,161697768
chr8	103600000	103850000	0,151284577

chr8	103600000	103850000	0,153335353
chr8	105450000	105700000	0,710558738
chr8	107000000	107250000	0,618089518
chr8	107900000	108150000	0,436316557
chr8	109200000	109450000	0,669628318
chr8	110450000	110700000	0,335314571
chr8	112950000	113200000	0,856332791
chr8	113800000	114050000	0,852146893
chr8	114300000	114550000	0,848344008
chr8	115750000	116000000	0,801063723
chr8	117800000	118050000	0,221315313
chr8	119000000	119250000	0,343868929
chr8	119650000	119900000	0,355240204
chr8	120850000	121100000	0,500902701
chr8	122600000	122850000	0,418339347
chr8	124150000	124400000	0,185717843
chr8	125350000	125600000	0,369162572
chr8	126300000	126550000	0,239573549
chr8	128600000	128850000	0,178203097
chr8	130900000	131150000	0,226308403
chr8	132800000	133050000	0,642469882
chr8	134250000	134500000	0,23244175
chr8	135650000	135900000	0,479065626
chr8	137400000	137650000	0,670964289
chr8	138050000	138300000	0,705444474
chr8	141950000	142200000	0,152864292
chr8	144600000	144850000	0,141080581

chr8	105450000	105700000	0,687442388
chr8	107750000	108000000	0,464799277
chr8	109200000	109450000	0,631711535
chr8	110400000	110650000	0,325831629
chr8	115450000	115700000	0,769701349
chr8	117800000	118050000	0,203790361
chr8	119000000	119250000	0,357802064
chr8	119600000	119850000	0,376354456
chr8	120850000	121100000	0,49491681
chr8	122650000	122900000	0,370762397
chr8	124150000	124400000	0,186062502
chr8	125350000	125600000	0,355267469
chr8	126300000	126550000	0,257575014
chr8	128650000	128900000	0,187452006
chr8	130900000	131150000	0,226824669
chr8	132800000	133050000	0,61838176
chr8	134250000	134500000	0,234564322
chr8	135650000	135900000	0,452295733
chr8	137400000	137650000	0,646910206
chr8	138050000	138300000	0,685830706
chr8	141500000	141750000	0,180050313
chr8	141950000	142200000	0,166114111
chr8	144600000	144850000	0,14086401
chr8	145650000	145900000	0,17807129
chr9	200000	450000	0,230000961
chr9	2450000	2700000	0,473180948
chr9	3750000	4000000	0,385759583

chr8	145600000	145850000	0,176421458
chr9	200000	450000	0,20873424
chr9	2450000	2700000	0,459134588
chr9	3700000	3950000	0,38254302
chr9	4550000	4800000	0,227813325
chr9	5800000	6050000	0,207346759
chr9	9900000	10150000	0,832427558
chr9	10900000	11150000	0,755650303
chr9	12650000	12900000	0,514565766
chr9	14100000	14350000	0,425627588
chr9	15450000	15700000	0,322002622
chr9	16200000	16450000	0,3269586
chr9	16850000	17100000	0,316036587
chr9	17550000	17800000	0,353124209
chr9	19100000	19350000	0,23247575
chr9	20550000	20800000	0,341427876
chr9	21600000	21850000	0,159004031
chr9	23550000	23800000	0,842969775
chr9	27000000	27250000	0,301976936
chr9	29100000	29350000	0,527733193
chr9	33250000	33500000	0,121818106
chr9	33850000	34100000	0,131346808
chr9	35800000	36050000	0,12805597
chr9	37400000	37650000	0,212413422
chr9	38300000	38550000	0,322038745
chr9	39750000	40000000	0,477137896
chr9	41600000	41850000	0,44465572

chr9	4500000	4750000	0,233836214
chr9	5800000	6050000	0,205322542
chr9	9950000	10200000	0,821317781
chr9	10900000	11150000	0,7274227
chr9	12650000	12900000	0,509453958
chr9	14100000	14350000	0,41401426
chr9	15450000	15700000	0,303398099
chr9	16200000	16450000	0,319611938
chr9	16850000	17100000	0,303220398
chr9	17500000	17750000	0,331553615
chr9	19100000	19350000	0,237827006
chr9	20550000	20800000	0,321673757
chr9	21600000	21850000	0,146812836
chr9	23500000	23750000	0,816669121
chr9	27050000	27300000	0,308090683
chr9	29050000	29300000	0,496833444
chr9	33200000	33450000	0,127717231
chr9	33850000	34100000	0,135038268
chr9	35800000	36050000	0,130449601
chr9	37400000	37650000	0,216402267
chr9	38300000	38550000	0,325595542
chr9	39750000	40000000	0,459392938
chr9	40850000	41100000	0,687477835
chr9	41700000	41950000	0,533210826
chr9	42850000	43100000	0,624927686
chr9	43300000	43550000	0,541204665
chr9	44350000	44600000	0,691574701

chr9	42150000	42400000	0,559059023
chr9	43250000	43500000	0,530409394
chr9	44400000	44650000	0,669796768
chr9	44850000	45100000	0,674529078
chr9	45550000	45800000	0,492556862
chr9	46750000	47000000	0,625563584
chr9	65650000	65900000	0,601049262
chr9	66500000	66750000	0,577320452
chr9	67650000	67900000	0,677829912
chr9	68200000	68450000	0,746277101
chr9	69050000	69300000	0,581490298
chr9	69900000	70150000	0,596092457
chr9	70900000	71150000	0,526895782
chr9	71600000	71850000	0,363694777
chr9	73000000	73250000	0,238640159
chr9	74700000	74950000	0,174578198
chr9	77450000	77700000	0,423072225
chr9	78900000	79150000	0,390566736
chr9	79700000	79950000	0,436600139
chr9	81350000	81600000	0,36808829
chr9	84200000	84450000	0,512813924
chr9	86400000	86650000	0,286684055
chr9	88450000	88700000	0,254398939
chr9	89900000	90150000	0,214680625
chr9	90900000	91150000	0,292405613
chr9	91850000	92100000	0,409575367
chr9	93900000	94150000	0,309071136

chr9	44900000	45150000	0,670075338
chr9	45450000	45700000	0,514093609
chr9	46850000	47100000	0,547161775
chr9	65600000	65850000	0,597587592
chr9	66500000	66750000	0,582298701
chr9	67700000	67950000	0,717205657
chr9	68200000	68450000	0,725834505
chr9	69100000	69350000	0,571888284
chr9	69850000	70100000	0,606994621
chr9	70900000	71150000	0,532730713
chr9	71650000	71900000	0,360625479
chr9	73050000	73300000	0,224894147
chr9	74550000	74800000	0,195617114
chr9	76800000	77050000	0,527264528
chr9	77450000	77700000	0,423526195
chr9	78850000	79100000	0,366757557
chr9	79700000	79950000	0,435059068
chr9	81350000	81600000	0,362570319
chr9	84200000	84450000	0,548824118
chr9	86400000	86650000	0,276002252
chr9	88450000	88700000	0,245242416
chr9	89900000	90150000	0,210188258
chr9	90900000	91150000	0,290197548
chr9	91850000	92100000	0,392936724
chr9	93900000	94150000	0,295484787
chr9	94750000	95000000	0,228509025
chr9	95750000	96000000	0,224243694

chr9	94750000	95000000	0,227730369	chr9	97400000	97650000	0,157253947
chr9	95750000	96000000	0,214053581	chr9	99150000	99400000	0,218766485
chr9	97350000	97600000	0,16956324	chr9	99700000	99950000	0,235915293
chr9	97900000	98150000	0,232756552	chr9	100550000	100800000	0,166045218
chr9	99150000	99400000	0,192430718	chr9	101550000	101800000	0,257140158
chr9	100500000	100750000	0,158184857	chr9	103050000	103300000	0,315807552
chr9	101550000	101800000	0,300135945	chr9	104100000	104350000	0,502416743
chr9	103050000	103300000	0,327349697	chr9	106750000	107000000	0,541018797
chr9	104150000	104400000	0,479755773	chr9	107800000	108050000	0,295458268
chr9	106700000	106950000	0,536652963	chr9	110050000	110300000	0,342097083
chr9	107900000	108150000	0,34088302	chr9	111850000	112100000	0,238420093
chr9	110050000	110300000	0,36581857	chr9	113150000	113400000	0,255690143
chr9	111850000	112100000	0,238414227	chr9	114650000	114900000	0,158414715
chr9	113150000	113400000	0,266478354	chr9	116050000	116300000	0,2202882
chr9	114650000	114900000	0,141775706	chr9	117100000	117350000	0,256184233
chr9	116050000	116300000	0,227286455	chr9	118700000	118950000	0,340570158
chr9	117150000	117400000	0,246369631	chr9	123400000	123650000	0,180608362
chr9	118600000	118850000	0,361919981	chr9	124650000	124900000	0,295596289
chr9	121050000	121300000	0,81212746	chr9	125950000	126200000	0,253500357
chr9	123450000	123700000	0,177714801	chr9	127500000	127750000	0,153042828
chr9	124700000	124950000	0,316238148	chr9	130650000	130900000	0,12387917
chr9	125950000	126200000	0,256414558	chr9	132200000	132450000	0,125570347
chr9	127500000	127750000	0,142555733	chr9	134050000	134300000	0,14110514
chr9	130650000	130900000	0,1178961	chr9	135150000	135400000	0,3079166
chr9	132200000	132450000	0,127879714	chr9	136000000	136250000	0,240025918
chr9	134050000	134300000	0,140637076	chr9	136900000	137150000	0,212198199
chr9	135100000	135350000	0,31420719	chr9	139750000	140000000	0,12132007

chr9	136000000	136250000	0,238268439
chr9	136950000	137200000	0,210929021
chr9	139750000	140000000	0,119356005
chrX	150000	400000	0,911027119
chrX	2000000	2250000	0,929230275
chrX	2850000	3100000	0,402320883
chrX	3450000	3700000	0,412031005
chrX	6000000	6250000	0,773921032
chrX	8250000	8500000	0,311876718
chrX	9100000	9350000	0,389801952
chrX	10450000	10700000	0,43671092
chrX	13000000	13250000	0,262924365
chrX	13500000	13750000	0,246106198
chrX	15450000	15700000	0,306242684
chrX	16650000	16900000	0,24516402
chrX	18600000	18850000	0,286357459
chrX	19800000	20050000	0,282428976
chrX	21850000	22100000	0,321074701
chrX	23850000	24100000	0,137463555
chrX	26850000	27100000	0,551779605
chrX	27450000	27700000	0,634150366
chrX	28350000	28600000	0,524903271
chrX	29550000	29800000	0,695260271
chrX	30800000	31050000	0,41095248
chrX	31750000	32000000	0,604375053
chrX	35650000	35900000	0,77934741
chrX	37550000	37800000	0,410884926

chrX	2850000	3100000	0,384925664
chrX	3450000	3700000	0,3910452
chrX	6000000	6250000	0,739154055
chrX	8250000	8500000	0,318967984
chrX	9100000	9350000	0,365819155
chrX	10450000	10700000	0,440088314
chrX	13000000	13250000	0,258459273
chrX	13500000	13750000	0,237849581
chrX	15450000	15700000	0,310574643
chrX	16650000	16900000	0,238248362
chrX	18550000	18800000	0,296346655
chrX	19750000	20000000	0,287117814
chrX	21850000	22100000	0,307253921
chrX	23900000	24150000	0,138096378
chrX	26850000	27100000	0,553944072
chrX	28350000	28600000	0,516285875
chrX	29550000	29800000	0,69339912
chrX	30800000	31050000	0,405152411
chrX	31750000	32000000	0,55275982
chrX	35400000	35650000	0,754574906
chrX	37500000	37750000	0,416821821
chrX	38550000	38800000	0,496959945
chrX	39700000	39950000	0,184022313
chrX	41000000	41250000	0,190040533
chrX	43950000	44200000	0,3554857
chrX	44600000	44850000	0,349770218
chrX	45200000	45450000	0,308318865

chrX	38500000	38750000	0,508293569
chrX	39700000	39950000	0,19629474
chrX	40900000	41150000	0,184357488
chrX	44050000	44300000	0,380680504
chrX	44550000	44800000	0,373081703
chrX	45250000	45500000	0,302342399
chrX	46600000	46850000	0,187304503
chrX	48700000	48950000	0,166803799
chrX	51800000	52050000	0,72756548
chrX	53250000	53500000	0,201884837
chrX	57600000	57850000	0,424039937
chrX	62550000	62800000	0,678180251
chrX	64750000	65000000	0,410630043
chrX	67550000	67800000	0,256540364
chrX	69550000	69800000	0,366946048
chrX	70450000	70700000	0,202801429
chrX	71350000	71600000	0,281002989
chrX	73300000	73550000	0,347937543
chrX	75300000	75550000	0,717557214
chrX	77000000	77250000	0,291412043
chrX	79950000	80200000	0,652420918
chrX	81750000	82000000	0,851844861
chrX	84200000	84450000	0,736643873
chrX	85100000	85350000	0,724409217
chrX	87400000	87650000	0,857438346
chrX	88550000	88800000	0,838326277
chrX	91650000	91900000	0,614444476

chrX	46500000	46750000	0,205565358
chrX	48700000	48950000	0,168616896
chrX	51700000	51950000	0,727836691
chrX	53250000	53500000	0,215536228
chrX	54100000	54350000	0,281443218
chrX	57600000	57850000	0,373571228
chrX	62600000	62850000	0,64925624
chrX	64750000	65000000	0,401806653
chrX	67550000	67800000	0,253844054
chrX	69550000	69800000	0,350977716
chrX	70400000	70650000	0,21307903
chrX	71350000	71600000	0,277198433
chrX	73300000	73550000	0,331410284
chrX	75300000	75550000	0,688336207
chrX	77000000	77250000	0,273753153
chrX	79950000	80200000	0,612081163
chrX	81600000	81850000	0,827461496
chrX	82900000	83150000	0,832675775
chrX	84200000	84450000	0,718174677
chrX	85050000	85300000	0,69928824
chrX	87450000	87700000	0,843828864
chrX	88650000	88900000	0,824664858
chrX	91650000	91900000	0,634088777
chrX	94050000	94300000	0,829308371
chrX	95750000	96000000	0,600263418
chrX	96750000	97000000	0,628814029
chrX	100200000	100450000	0,268186288

chrX	93800000	94050000	0,851346914
chrX	95750000	96000000	0,62813243
chrX	96750000	97000000	0,63652166
chrX	98300000	98550000	0,835291382
chrX	100250000	100500000	0,276804869
chrX	102600000	102850000	0,168834385
chrX	104700000	104950000	0,669177602
chrX	106150000	106400000	0,360738234
chrX	106800000	107050000	0,287110419
chrX	109000000	109250000	0,330713868
chrX	110800000	111050000	0,675596512
chrX	112900000	113150000	0,652537772
chrX	114550000	114800000	0,302626411
chrX	117350000	117600000	0,21438231
chrX	118700000	118950000	0,183297263
chrX	119500000	119750000	0,340357775
chrX	120900000	121150000	0,70973325
chrX	122900000	123150000	0,224393857
chrX	124300000	124550000	0,803099925
chrX	125050000	125300000	0,824234468
chrX	125650000	125900000	0,837033852
chrX	126300000	126550000	0,826918231
chrX	126800000	127050000	0,827892596
chrX	129050000	129300000	0,174918798
chrX	131450000	131700000	0,250302389
chrX	133850000	134100000	0,196177384
chrX	135050000	135300000	0,240244818
chrX	102600000	102850000	0,173194508
chrX	104700000	104950000	0,689044729
chrX	106100000	106350000	0,326458419
chrX	106850000	107100000	0,265970195
chrX	109000000	109250000	0,31655507
chrX	110800000	111050000	0,657080188
chrX	112900000	113150000	0,66240721
chrX	114550000	114800000	0,294998245
chrX	117300000	117550000	0,219197453
chrX	118700000	118950000	0,193996535
chrX	119450000	119700000	0,311154638
chrX	120850000	121100000	0,723909324
chrX	122900000	123150000	0,219851196
chrX	129100000	129350000	0,176676232
chrX	131450000	131700000	0,256795357
chrX	133850000	134100000	0,193792716
chrX	135050000	135300000	0,266290391
chrX	135750000	136000000	0,350170589
chrX	137600000	137850000	0,784316294
chrX	138850000	139100000	0,53406002
chrX	142050000	142300000	0,46443947
chrX	144100000	144350000	0,811541053
chrX	145200000	145450000	0,828192927
chrX	146900000	147150000	0,701201156
chrX	149700000	149950000	0,275950914
chrX	151000000	151250000	0,310450788
chrX	152000000	152250000	0,284749383

chrX	135750000	136000000	0,366763818
chrX	138900000	139150000	0,578243504
chrX	140900000	141150000	0,661475268
chrX	142050000	142300000	0,506297834
chrX	144500000	144750000	0,841042346
chrX	146900000	147150000	0,728964832
chrX	149700000	149950000	0,286883044
chrX	151000000	151250000	0,32464884
chrX	151900000	152150000	0,315847989
chrX	153050000	153300000	0,149583008
chrX	153600000	153850000	0,151892633

chrX	153050000	153300000	0,144595014
chrX	153550000	153800000	0,14523108

Supplementary Table S6

List and coordinates of the Temporal Transition Regions* of PREP1 down-regulated HeLa cells.

	Start	End	Delta S50
chr1	5200000	5850000	0.24535939861
chr1	12850000	13350000	-0.2528660703
chr1	14500000	15250000	0.28397956876
chr1	18000000	18550000	-0.2360689436
chr1	29150000	29750000	-0.2501976977
chr1	29700000	30200000	-0.25831291299
chr1	30450000	31050000	0.24034579720
chr1	34050000	34600000	-0.2628823371
chr1	34550000	35050000	0.17321637133
chr1	35000000	35800000	0.30535136338
chr1	37050000	37450000	-0.2014136739
chr1	38550000	38950000	-0.1659366746
chr1	48500000	49700000	-0.3027886910
chr1	49650000	50650000	0.24612718524
chr1	55650000	56700000	-0.2145856152
chr1	57250000	58100000	-0.2719916162
chr1	58050000	58800000	0.27236558868
chr1	60750000	61250000	0.21010130372
chr1	68950000	69550000	-0.1857372636
chr1	71050000	71650000	-0.2572882562
chr1	76350000	77000000	-0.1919043329
chr1	76950000	77600000	0.19947289797
chr1	78750000	79900000	-0.4078606884
chr1	83050000	84300000	0.27652894126
chr1	86200000	86700000	-0.1705516074
chr1	90650000	91100000	-0.2254175170
chr1	95700000	96450000	-0.3233542345
chr1	99000000	99500000	-0.1505269261
chr1	99450000	99950000	0.15047181901
chr1	101850000	102600000	-0.2980421890
chr1	111050000	111500000	-0.1863144869
chr1	115250000	115950000	-0.15010003699
chr1	118500000	119100000	-0.2012588932
chr1	119050000	119750000	0.17444727068
chr1	121400000	141800000	-0.3683532179
chr1	143050000	143600000	0.21941892062
chr1	145800000	146450000	-0.1746644594
chr1	152200000	152800000	-0.3142112875
chr1	152750000	153200000	0.22456071737
chr1	157150000	157600000	-0.2135650880

chr1	158200000	158800000	-0.2343674789
chr1	158750000	159650000	0.24928255044
chr1	162800000	163750000	-0.3642754549
chr1	163700000	164300000	0.17177441041
chr1	165100000	165450000	0.14609071573
chr1	165950000	166500000	-0.2507629472
chr1	168350000	168850000	-0.1512399646
chr1	169900000	170750000	-0.1848286851
chr1	170700000	171250000	0.15915946191
chr1	174350000	174800000	-0.2183252184
chr1	177400000	177800000	0.14404780885
chr1	181300000	181750000	-0.2538217709
chr1	183600000	184400000	-0.2225671567
chr1	187000000	188550000	-0.3429070018
chr1	191050000	192400000	0.25834634427
chr1	193300000	194150000	-0.2519961112
chr1	205750000	206100000	-0.1466568830
chr1	207650000	208450000	-0.2906810696
chr1	208400000	208950000	-0.2080032184
chr1	210950000	211350000	0.19069775182
chr1	213350000	213900000	-0.2390241943
chr1	213850000	214350000	0.23752240435
chr1	215000000	215550000	-0.2925866696
chr1	218900000	219550000	-0.2352746668
chr1	219500000	219950000	0.18040868491
chr1	221650000	222200000	-0.1624127598
chr1	222150000	222650000	0.22992214997
chr1	224600000	225100000	-0.1882768527
chr1	236900000	237350000	-0.1817115952
chr1	238100000	239050000	-0.2993476125
chr1	240600000	241500000	0.30381887691
chr1	242300000	242850000	-0.2730656802
chr1	243600000	244350000	0.14001746879
chr1	246050000	246450000	0.15291275367
chr1	247550000	248300000	-0.3257811255
chr10	1200000	1650000	-0.1739850906
chr10	13600000	14050000	-0.1672759340
chr10	14000000	14400000	0.16073408380
chr10	15350000	15850000	-0.1443110448
chr10	19050000	20000000	-0.2607555961
chr10	19950000	21050000	0.23697076341
chr10	23800000	24250000	-0.1695561180
chr10	24200000	24650000	0.17169521111
chr10	25400000	26150000	-0.2487124138
chr10	26100000	26550000	0.15794478298
chr10	33500000	34100000	-0.1658088425

chr10	35900000	36750000	-0.3049582164
chr10	39100000	41650000	-0.1862703909
chr10	41600000	42400000	0.15077171645
chr10	44150000	44800000	-0.2910680589
chr10	50100000	50500000	0.14949307907
chr10	52600000	53200000	-0.2018715303
chr10	53150000	53850000	0.25154672221
chr10	54750000	56900000	-0.3514818922
chr10	58700000	59800000	0.33775700818
chr10	64100000	64650000	-0.1953661888
chr10	64600000	65200000	0.14860704374
chr10	65700000	66650000	-0.35284777
chr10	67400000	68300000	0.18062883054
chr10	68900000	69350000	0.22017620536
chr10	77200000	77950000	-0.2989142255
chr10	78700000	79400000	0.24838002112
chr10	79900000	80400000	-0.1666706616
chr10	82350000	83300000	-0.3324359551
chr10	87650000	88000000	0.18111999722
chr10	91600000	92100000	-0.2438737768
chr10	92050000	92450000	0.17280869498
chr10	100250000	100700000	-0.1608115781
chr10	100650000	101050000	0.16264598038
chr10	106250000	108000000	-0.3955294531
chr10	110150000	111500000	0.29416541077
chr10	112850000	113550000	-0.3297211586
chr10	113500000	113950000	0.15204145970
chr10	116750000	117450000	-0.3032163267
chr10	117850000	118350000	0.17248248641
chr10	119250000	119800000	-0.1698400100
chr10	119750000	120600000	0.30523082683
chr10	121750000	122250000	-0.2391429113
chr10	124250000	125150000	-0.2696915988
chr10	127650000	128350000	-0.2796208616
chr10	128900000	129650000	0.17428503928
chr10	130050000	130700000	-0.2103427972
chr11	4200000	5000000	-0.3296259218
chr11	5850000	6200000	0.14699534273
chr11	6850000	7250000	-0.1553921545
chr11	13400000	13900000	-0.1533051171
chr11	14600000	15400000	-0.3266217656
chr11	20600000	22050000	-0.2989657916
chr11	25800000	27100000	0.3538243451
chr11	29450000	30050000	-0.2163872099
chr11	32050000	32750000	0.14888132988
chr11	36650000	37600000	-0.3322909124

chr11	48250000	49500000	-0.4145759200
chr11	51550000	53950000	-0.1641224826
chr11	55850000	56500000	0.2240846223
chr11	57700000	58150000	-0.1991814307
chr11	58650000	59100000	0.1721857870
chr11	59600000	60100000	-0.2325755053
chr11	60050000	60450000	0.1643304067
chr11	79350000	80100000	0.1510855997
chr11	80550000	81550000	-0.2406727961
chr11	81500000	82400000	0.3341346362
chr11	83600000	84500000	-0.2148151158
chr11	84450000	85150000	0.2264817328
chr11	86850000	87450000	-0.2732551428
chr11	88250000	89100000	-0.2208719488
chr11	92350000	93100000	0.2050208104
chr11	100700000	101150000	-0.1531036319
chr11	101100000	101650000	0.1793407863
chr11	103100000	104150000	-0.2949891753
chr11	106550000	107300000	0.1931720376
chr11	108750000	109250000	-0.1823922447
chr11	109200000	109700000	0.2294583685
chr11	110400000	110850000	-0.2151674281
chr11	112200000	112700000	-0.1998235607
chr11	114450000	115050000	-0.2515014781
chr11	115750000	116400000	0.2818722585
chr11	119250000	119700000	-0.1439545446
chr11	123550000	124050000	-0.2515763525
chr11	124000000	124350000	0.1425551361
chr11	126450000	127400000	-0.3236890025
chr11	127350000	127900000	0.1589118271
chr11	131500000	132450000	-0.2435136212
chr12	1950000	2450000	-0.2238344848
chr12	2400000	2800000	0.1757476740
chr12	3350000	3800000	-0.2098100424
chr12	11500000	12300000	0.1447255330
chr12	13550000	14100000	-0.2985351558
chr12	16200000	17500000	-0.3489676428
chr12	22900000	23700000	-0.2692869248
chr12	28200000	28700000	-0.1641042807
chr12	33150000	33800000	-0.2870308480
chr12	34800000	37150000	-0.1935734184
chr12	41750000	42350000	0.1679262315
chr12	55000000	55500000	-0.2682540347
chr12	55450000	55900000	0.2124416373
chr12	60650000	61900000	-0.2075226987
chr12	61850000	62500000	0.2072297466

chr12	66600000	67250000	-0.1905604107
chr12	67950000	68550000	0.20921004711
chr12	72400000	73200000	-0.3127210064
chr12	73150000	74300000	0.1752178644
chr12	77450000	77950000	-0.2066860411
chr12	81200000	82450000	-0.2494700407
chr12	84400000	85400000	0.14805785594
chr12	97100000	97950000	-0.3308902943
chr12	97900000	98550000	0.26677309353
chr12	99200000	99750000	-0.2582507603
chr12	99700000	100350000	0.1666492540
chr12	103550000	104100000	0.2659192030
chr12	108400000	108800000	0.1648648094
chr12	114150000	114750000	-0.2471788617
chr12	115800000	116300000	0.16593132591
chr12	116250000	117000000	0.3033426695
chr12	118800000	119450000	-0.2548639334
chr12	119400000	120050000	0.21577844027
chr12	125750000	126900000	-0.4464338326
chr12	129700000	130350000	0.28140540011
chr13	19350000	20100000	0.26328444853
chr13	22200000	22900000	-0.2594061364
chr13	22850000	23800000	0.2312299654
chr13	46050000	46650000	-0.1644731398
chr13	50500000	51300000	-0.1530088648
chr13	53300000	54450000	-0.3764460651
chr13	56150000	57100000	-0.1735344445
chr13	58650000	60000000	0.2008121196
chr13	61050000	62200000	-0.3253152004
chr13	72250000	73300000	0.34712763843
chr13	75300000	75800000	0.21052393057
chr13	76450000	77000000	-0.2416417065
chr13	76950000	77400000	0.16824767871
chr13	78100000	78750000	-0.2202292925
chr13	78700000	79700000	0.3093900468
chr13	94300000	95000000	0.1622417599
chr13	96550000	97150000	-0.2133657844
chr13	97100000	97550000	0.18667451022
chr13	102500000	102900000	-0.1636013786
chr13	103550000	104450000	-0.2840897181
chr13	107200000	108000000	-0.1714551602
chr13	107950000	108750000	0.18411802617
chr13	112100000	112500000	-0.1718298401
chr13	112450000	113000000	0.31049689971
chr14	20200000	20600000	0.1728762197
chr14	22050000	22450000	-0.1564061389

chr14	25700000	26400000	-0.2468134903
chr14	30200000	30750000	0.17353713366
chr14	33150000	33800000	-0.2695212546
chr14	33750000	34300000	0.17644444394
chr14	40000000	41150000	-0.3870442253
chr14	44100000	45200000	0.29303216617
chr14	45800000	47350000	-0.3579516279
chr14	48700000	49750000	0.36359263160
chr14	56250000	56900000	-0.2502854266
chr14	58050000	58450000	0.15662789741
chr14	62250000	62800000	-0.1443379765
chr14	63350000	63900000	0.14007191855
chr14	72500000	72950000	0.16233777648
chr14	76000000	76900000	-0.1463528832
chr14	78450000	78900000	-0.2300416095
chr14	78850000	79250000	0.16086798316
chr14	82100000	82900000	-0.3127504540
chr14	87700000	88750000	0.28779619987
chr14	93800000	94200000	-0.1403453978
chr14	94650000	95150000	-0.1728346970
chr14	95100000	95550000	0.14855826936
chr14	97250000	98700000	-0.3325275938
chr14	98650000	99700000	0.31024681799
chr14	99650000	100300000	0.31823753958
chr14	106150000	106850000	-0.2906471080
chr15	19200000	20000000	0.14139551380
chr15	21250000	21650000	-0.1758478238
chr15	22200000	22700000	0.24832977965
chr15	23500000	24350000	-0.2956071336
chr15	26750000	28200000	0.19863395188
chr15	29400000	29850000	0.16148014966
chr15	33150000	33700000	-0.1830948960
chr15	33650000	34050000	0.16912675536
chr15	46050000	46900000	-0.2851682069
chr15	46850000	47500000	0.14848233159
chr15	53150000	54050000	-0.3065635349
chr15	54500000	55350000	0.26399491775
chr15	61000000	61650000	-0.3108459814
chr15	61600000	62150000	0.16009024636
chr15	73450000	74400000	0.18991269778
chr15	79350000	79900000	-0.1890776300
chr15	81950000	82400000	0.19669462210
chr15	83800000	84300000	-0.2144560501
chr15	84250000	84700000	0.19438950849
chr15	84650000	85450000	0.33481485750
chr15	86500000	87200000	-0.2687038559

chr15	87150000	87950000	0.148618629877
chr15	91700000	92250000	-0.2598451025
chr15	92500000	93050000	0.22680718390
chr15	93700000	94150000	-0.2680573355
chr15	94100000	94500000	0.17529072825
chr15	95050000	96050000	-0.3275277314
chr15	97100000	97900000	-0.1417970600
chr15	97850000	98800000	0.31648552770
chr15	100650000	101000000	0.16459747786
chr16	5250000	6600000	-0.4712775437
chr16	7350000	8600000	0.36241320463
chr16	9350000	9900000	-0.2782512926
chr16	9850000	10350000	0.20643113146
chr16	12250000	12850000	-0.2716976135
chr16	13300000	13850000	0.23122410663
chr16	16600000	17400000	-0.3122623271
chr16	17350000	18300000	0.23317853236
chr16	19950000	20350000	-0.1695202550
chr16	22550000	23000000	-0.1976392444
chr16	25350000	26250000	-0.3335424160
chr16	26200000	27050000	0.22228740633
chr16	31950000	32550000	-0.1757440784
chr16	45600000	46450000	0.34419004151
chr16	47200000	47600000	-0.1907593788
chr16	47550000	48150000	0.16529594494
chr16	48800000	49400000	-0.2130610858
chr16	49350000	49900000	0.22889708778
chr16	50850000	51700000	-0.2672233491
chr16	55750000	56200000	0.16056418765
chr16	58950000	59900000	-0.4117446521
chr16	60600000	61650000	0.26074283898
chr16	62150000	63000000	-0.2700028577
chr16	64600000	66150000	0.36111012923
chr16	70800000	71250000	-0.2035314796
chr16	72100000	72650000	-0.1754071991
chr16	75800000	76700000	-0.3094555413
chr16	79700000	80400000	0.25586779547
chr16	80350000	81050000	0.31556974295
chr16	83150000	83750000	0.24857973059
chr16	83700000	84200000	0.25081535820
chr17	5450000	5900000	-0.1770933118
chr17	11200000	11700000	0.15513094721
chr17	12850000	13500000	-0.1887400429
chr17	14400000	14900000	0.20374278186
chr17	21400000	21950000	-0.2732485498
chr17	22250000	24550000	-0.1513178137

chr17	24500000	25350000	0.33184916382
chr17	30900000	32050000	-0.4163937756
chr17	32000000	33150000	0.23794168609
chr17	49450000	51150000	-0.4264132260
chr17	63100000	63750000	-0.1484061389
chr17	69850000	70350000	0.15483150137
chr17	71450000	71850000	-0.1796659303
chr18	4100000	4850000	-0.3261120459
chr18	5500000	6300000	0.27107781074
chr18	7600000	7950000	-0.1539925617
chr18	7900000	8250000	0.14222429867
chr18	15400000	17800000	-0.1501339491
chr18	17750000	18550000	0.41304160869
chr18	22200000	22950000	-0.2947878225
chr18	22900000	23500000	0.16652689146
chr18	24350000	25000000	-0.2462904920
chr18	27500000	28500000	0.23606127000
chr18	30150000	31200000	-0.2563917191
chr18	31150000	32300000	0.26466601746
chr18	33950000	34650000	-0.1978958589
chr18	35000000	35950000	-0.2313509591
chr18	42650000	43150000	0.16641291513
chr18	48700000	49550000	-0.3163196262
chr18	50350000	51500000	0.30928146985
chr18	52750000	53550000	-0.2453507539
chr18	53500000	54100000	0.16099814870
chr18	56700000	57200000	-0.1778419394
chr18	57750000	58800000	-0.2821243046
chr18	58750000	59700000	0.27205478267
chr18	61700000	62850000	-0.3094752142
chr18	66850000	67750000	0.24843691053
chr18	68250000	69400000	-0.3489456813
chr18	69350000	70200000	0.21140946237
chr18	73650000	74300000	0.18282236493
chr18	75000000	75850000	-0.2307210112
chr18	75800000	76650000	0.14149717812
chr19	19900000	20600000	-0.2970906157
chr19	21550000	22750000	-0.2604215117
chr19	24550000	27000000	-0.1884807778
chr19	28900000	30000000	0.24533934807
chr19	31650000	32600000	0.25795128052
chr19	32550000	33100000	0.25346660053
chr19	36750000	37350000	-0.2276937674
chr19	54750000	55200000	-0.1494115679
chr19	55150000	55550000	0.14239146434
chr19	56800000	57250000	-0.1447867119

chr2	2000000	2600000	-0.3004339588
chr2	2550000	3200000	0.2633750224
chr2	3900000	4700000	-0.394802832
chr2	7700000	8550000	0.2101372279
chr2	12050000	13000000	-0.3263466607
chr2	16750000	17350000	-0.2352627956
chr2	19000000	19950000	0.3336646858
chr2	20900000	21350000	-0.1511103241
chr2	29500000	29950000	-0.1861875900
chr2	29900000	30250000	0.1460118963
chr2	30900000	31300000	-0.172653744
chr2	33850000	34850000	-0.3392768359
chr2	36250000	37050000	0.3268113419
chr2	40000000	40450000	-0.1783396930
chr2	41250000	41850000	0.2967412095
chr2	48900000	49800000	-0.3336835580
chr2	56350000	57450000	-0.4480152354
chr2	57400000	57950000	0.1494349236
chr2	59000000	59950000	-0.2786442871
chr2	59900000	60800000	0.3140985607
chr2	65950000	66650000	-0.161867157
chr2	74950000	75550000	-0.174687785
chr2	83700000	84600000	0.3153442342
chr2	84550000	85350000	0.3780288294
chr2	89300000	89800000	-0.296960698
chr2	90500000	91100000	-0.1502322778
chr2	91050000	91600000	0.1773759592
chr2	92250000	94600000	-0.2249090853
chr2	94550000	95400000	0.2778418745
chr2	100150000	100700000	-0.218352653
chr2	100650000	101550000	0.2717014979
chr2	102350000	103250000	-0.2862895952
chr2	104300000	105750000	0.4651114200
chr2	114800000	115950000	-0.404908072
chr2	116400000	118550000	0.2863686591
chr2	122700000	124050000	-0.4286511186
chr2	126700000	127700000	0.3454535762
chr2	129100000	129650000	-0.2435182667
chr2	130200000	130650000	0.1682206666
chr2	133350000	134200000	-0.1519249596
chr2	137200000	137700000	-0.2098454460
chr2	145400000	146800000	-0.210717852
chr2	153350000	154000000	-0.2781959773
chr2	163200000	163650000	-0.173704068
chr2	165950000	166500000	-0.2898860370
chr2	167750000	168950000	0.2224320087

chr2	177300000	177750000	0.19256587559
chr2	178300000	178800000	-0.14781943424
chr2	182000000	182450000	0.15273835735
chr2	186550000	187150000	0.17918433500
chr2	187600000	188000000	-0.15130669214
chr2	194650000	196150000	0.27231872396
chr2	198650000	199500000	-0.29746910314
chr2	199450000	200600000	0.23844462557
chr2	205500000	206150000	-0.1494563724
chr2	209300000	209800000	-0.2400130884
chr2	210550000	211100000	0.17782285100
chr2	217450000	218050000	-0.2067354237
chr2	218000000	218600000	0.17738259738
chr2	222700000	223350000	0.20350674960
chr2	228900000	229500000	-0.2820642165
chr2	229450000	230050000	0.17601845068
chr2	237300000	237700000	-0.1487852405
chr20	6850000	7650000	-0.2523181173
chr20	9150000	9950000	0.25652381946
chr20	10550000	11050000	-0.1634361400
chr20	11000000	11550000	0.16106410530
chr20	12000000	12700000	-0.2502676201
chr20	16850000	17250000	0.19316064556
chr20	18700000	19250000	-0.20999565624
chr20	19200000	19600000	0.15877543146
chr20	21550000	22050000	-0.2015691457
chr20	22000000	22450000	0.15782344463
chr20	23600000	24300000	-0.1561595094
chr20	24250000	24900000	0.25430739913
chr20	26200000	28700000	-0.30727283854
chr20	29400000	29900000	0.38078828842
chr20	37800000	38450000	-0.2456532084
chr20	38400000	39400000	0.34768087695
chr20	40350000	41250000	-0.3210949749
chr20	41200000	41900000	0.29225938421
chr20	44700000	45300000	-0.1814553036
chr20	45250000	45750000	0.15237045013
chr20	46300000	46800000	-0.20012994124
chr20	46750000	47250000	0.16794147806
chr20	50800000	51250000	-0.1958386278
chr20	53000000	53950000	-0.3997985767
chr20	53900000	54850000	0.28674081944
chr20	57800000	58300000	-0.1760490187
chr20	59300000	60400000	0.30001254513
chr20	60350000	61050000	0.31032828311
chr21	11100000	13600000	-0.1843744994

chr21	19100000	20950000	-0.4262969985
chr21	29100000	30150000	0.31509665554
chr21	30900000	32100000	-0.4313857823
chr21	32050000	32900000	0.22011577593
chr21	39050000	39500000	-0.2212109026
chr21	39450000	39850000	0.17571432704
chr21	40950000	41650000	-0.3012809086
chr21	41950000	42450000	0.16097623848
chr22	15300000	16050000	0.14649013894
chr22	16750000	17350000	0.27627313300
chr22	22300000	22900000	-0.1460679270
chr22	33400000	34450000	-0.2919153684
chr22	34400000	35450000	0.34009021119
chr22	47300000	48300000	-0.3547371505
chr22	49300000	50000000	0.31477115878
chr22	51100000	51304566	-0.2004784901
chr3	3500000	4150000	0.24760609177
chr3	4100000	4650000	0.25347537416
chr3	5350000	6000000	-0.3343029557
chr3	7700000	8350000	0.23704501308
chr3	10350000	10800000	-0.1759848346
chr3	18500000	19300000	-0.2453994175
chr3	19250000	19800000	0.16765120799
chr3	20300000	21500000	-0.2941828428
chr3	22400000	23050000	0.15870447098
chr3	25900000	26550000	-0.2176843697
chr3	26500000	27150000	0.25335857977
chr3	27800000	28650000	-0.2730560768
chr3	34000000	35050000	-0.3756055773
chr3	35900000	36800000	0.29636889469
chr3	39500000	39950000	-0.1956927821
chr3	40650000	41050000	-0.1423024034
chr3	58700000	59400000	-0.3036200294
chr3	66750000	67400000	-0.1458957253
chr3	68400000	68900000	0.19083223292
chr3	73300000	74750000	-0.3970150236
chr3	80150000	81000000	-0.1744922217
chr3	85500000	86650000	0.28897939857
chr3	88250000	89400000	-0.2706149698
chr3	92750000	93550000	0.30987140959
chr3	96250000	97350000	0.25045656642
chr3	102150000	103250000	-0.3228039095
chr3	103200000	104450000	0.18449377909
chr3	110100000	111150000	0.27893556323
chr3	113700000	114250000	-0.1504743675
chr3	115650000	116450000	-0.2652789150

chr3	117100000	118700000	0.36211575325
chr3	120350000	120850000	-0.1648710923
chr3	129800000	130200000	-0.1574282815
chr3	132650000	133000000	0.15402800808
chr3	134400000	135100000	-0.3546116911
chr3	135050000	135600000	0.22475816289
chr3	136850000	137300000	-0.2388998520
chr3	137250000	137600000	0.15666193754
chr3	139200000	140050000	-0.2480024528
chr3	140000000	140700000	0.25839228787
chr3	142850000	143550000	-0.2613224140
chr3	146400000	147200000	-0.1429143642
chr3	147700000	148500000	0.26361149510
chr3	153900000	154600000	-0.1882707078
chr3	154550000	155250000	0.23757347086
chr3	157250000	157950000	-0.1530025655
chr3	161150000	162100000	-0.1621081046
chr3	165800000	167300000	0.27638211785
chr3	172650000	173350000	-0.3430380199
chr3	175800000	176600000	0.23457385521
chr3	179550000	180150000	-0.2835769227
chr3	181850000	182300000	0.20016473061
chr3	190300000	190800000	-0.1825366864
chr3	197750000	198022430	-0.1421151929
chr4	0	500000	0.19048327088
chr4	4950000	5450000	-0.1463724378
chr4	5400000	6150000	0.17673081601
chr4	8550000	9500000	-0.3344403785
chr4	10250000	11100000	-0.2273389974
chr4	16350000	16950000	-0.2376524266
chr4	16900000	17350000	0.14527042824
chr4	18050000	19000000	-0.1926044237
chr4	26950000	28150000	-0.2860390006
chr4	35150000	35850000	0.15802164877
chr4	36350000	36900000	-0.1901116385
chr4	36850000	37500000	0.2048393437
chr4	41750000	42650000	-0.2690730764
chr4	42600000	43850000	-0.2957522039
chr4	45800000	46500000	-0.1714202790
chr4	46450000	47300000	0.25325523046
chr4	49050000	49500000	-0.2905635896
chr4	49650000	51950000	-0.1401006012
chr4	51900000	52650000	0.17703858723
chr4	55500000	55950000	0.18250115070
chr4	58200000	59900000	-0.3933524664
chr4	69400000	70450000	-0.2945766425

chr4	70400000	71350000	0.26873382138
chr4	79850000	80300000	-0.2106101652
chr4	82500000	83050000	0.23779230268
chr4	84650000	85150000	-0.2532446458
chr4	91100000	91550000	-0.1531067003
chr4	94650000	95100000	0.16756888629
chr4	96000000	96750000	-0.2324171008
chr4	98200000	98950000	0.19188844258
chr4	101000000	101550000	-0.1694783956
chr4	104150000	105200000	-0.3191644704
chr4	105150000	105900000	0.16364080980
chr4	107250000	107950000	-0.2802542377
chr4	107900000	108400000	0.16114754845
chr4	112250000	112800000	0.15276406274
chr4	115050000	116350000	-0.3276229524
chr4	121000000	121550000	-0.1849571231
chr4	121500000	122400000	0.20291239771
chr4	127350000	128300000	0.18571072962
chr4	130200000	131650000	-0.2876780280
chr4	144550000	145250000	-0.2108296692
chr4	145200000	145900000	0.17838154538
chr4	147150000	147750000	-0.1709051903
chr4	147700000	148250000	0.19980881086
chr4	148850000	149900000	-0.3674782788
chr4	149850000	150650000	0.32546899668
chr4	151150000	151500000	-0.1510042325
chr4	154600000	155250000	-0.2944836691
chr4	158450000	158900000	0.18432816722
chr4	160500000	161650000	-0.3002171129
chr4	161600000	162450000	0.15864874699
chr4	163050000	163650000	-0.1558149885
chr4	167000000	168150000	-0.2959079815
chr4	168100000	168950000	0.28250210355
chr4	170000000	171050000	-0.2930907843
chr4	171000000	172050000	-0.2713074268
chr4	174400000	174750000	-0.1569415703
chr4	175400000	176200000	-0.3068756963
chr4	176150000	176950000	0.25721155250
chr4	178450000	180100000	-0.2860064274
chr4	180800000	182800000	0.20756888228
chr5	1800000	2900000	-0.3508200090
chr5	4000000	5050000	0.31365476048
chr5	5500000	6000000	-0.2078020348
chr5	5950000	6450000	0.19164667839
chr5	6900000	7500000	-0.2540712609
chr5	9350000	10000000	0.31097123880

chr5	10800000	11850000	-0.4219264354
chr5	13000000	13750000	0.26715748566
chr5	15200000	15800000	-0.2929199500
chr5	15750000	16350000	0.21721054661
chr5	17500000	18200000	-0.2852268509
chr5	30300000	30900000	-0.1445027167
chr5	30850000	31400000	0.15904125901
chr5	35000000	35400000	-0.1592536212
chr5	37500000	38000000	-0.1504401532
chr5	37950000	38350000	0.14016573522
chr5	39650000	40050000	-0.1593752888
chr5	41000000	41450000	-0.2133275478
chr5	42350000	42750000	0.16769768019
chr5	43750000	44600000	-0.2998247583
chr5	48650000	49500000	0.27731962409
chr5	50150000	51000000	-0.2477466606
chr5	50950000	51750000	0.24340960019
chr5	59250000	59700000	-0.1588915676
chr5	62000000	63050000	-0.2541835116
chr5	63000000	64200000	0.28083766826
chr5	65450000	66150000	-0.1495719984
chr5	66650000	67200000	-0.1974429756
chr5	84950000	86000000	0.21561113176
chr5	86650000	87200000	-0.1653037068
chr5	88750000	89600000	0.19747914819
chr5	96600000	97350000	-0.2652353720
chr5	97300000	98000000	0.20752677473
chr5	98500000	99350000	-0.3115194735
chr5	101000000	101850000	0.19909290131
chr5	102750000	103350000	-0.1563957413
chr5	109000000	109750000	-0.2151347303
chr5	112900000	113300000	-0.1712906014
chr5	113950000	114350000	0.16058159947
chr5	117100000	118150000	0.26591173825
chr5	118950000	119700000	-0.3017073534
chr5	120400000	121250000	0.27458552966
chr5	124300000	124900000	-0.1654692197
chr5	124850000	125400000	0.23474795576
chr5	126050000	126700000	-0.2222627255
chr5	128100000	128950000	-0.2228513491
chr5	129500000	130250000	0.28767189598
chr5	135600000	136200000	-0.2170520819
chr5	136150000	136800000	0.22243575242
chr5	140150000	140600000	-0.1570947176
chr5	143650000	144200000	-0.2132413522
chr5	144150000	145150000	0.27834995715

chr5	146750000	147100000	0.15702054142
chr5	151350000	152350000	-0.3269314218
chr5	152300000	153300000	0.27324789668
chr5	154450000	155650000	-0.3981710891
chr5	155600000	156450000	0.18091391975
chr5	157400000	157900000	-0.1918856136
chr5	157850000	158350000	0.23934676879
chr5	159950000	161300000	-0.4386787154
chr5	161250000	162700000	0.26023567634
chr5	163150000	163900000	-0.2576069100
chr5	165750000	166850000	0.29133356986
chr5	168150000	168650000	-0.2279069082
chr6	5200000	5600000	-0.1704345736
chr6	6050000	6450000	0.14280917514
chr6	8200000	8800000	-0.2024774046
chr6	18550000	19200000	-0.2666695379
chr6	23550000	24200000	0.22854073127
chr6	39300000	39950000	-0.1571366213
chr6	39900000	40650000	0.22354081917
chr6	44450000	45000000	-0.2607671721
chr6	45700000	46150000	-0.1499049251
chr6	46100000	46550000	0.15969829737
chr6	47600000	48200000	-0.2256651807
chr6	49800000	51200000	-0.3153615955
chr6	51150000	51900000	0.27237589378
chr6	53750000	54400000	-0.1483383474
chr6	57250000	57800000	-0.3316712631
chr6	58700000	61150000	-0.2127198090
chr6	62550000	63850000	0.32441712506
chr6	64550000	65550000	-0.3792790264
chr6	69150000	70900000	0.36160819144
chr6	72350000	73100000	-0.1869614294
chr6	73050000	73900000	0.22811631891
chr6	74500000	75000000	-0.1559461004
chr6	76650000	77400000	-0.3226866830
chr6	86550000	87100000	-0.2742220250
chr6	87050000	87700000	0.20031998945
chr6	88600000	89000000	-0.1661898751
chr6	88950000	89500000	0.19102192778
chr6	91300000	92000000	-0.2037032487
chr6	98850000	99600000	0.24547907777
chr6	103950000	105250000	0.25575169297
chr6	114400000	115550000	-0.2832279398
chr6	115500000	116300000	0.22794004821
chr6	118000000	118500000	-0.2457195155
chr6	119350000	120350000	-0.2773328789

chr6	120300000	121450000	0.19834364741
chr6	123650000	125100000	0.32346599058
chr6	126500000	127100000	-0.2294581620
chr6	130300000	130850000	-0.2371966239
chr6	141900000	142350000	0.15688453695
chr6	146300000	146700000	-0.1542262080
chr6	147600000	148350000	-0.2375390146
chr6	148300000	149150000	0.21285807480
chr6	153450000	153950000	-0.1877151500
chr6	153900000	154450000	0.19624282198
chr6	154400000	155050000	0.25768468268
chr6	155650000	156450000	-0.2190066261
chr6	159450000	159850000	-0.1624998838
chr6	162800000	163550000	0.19540401691
chr6	164000000	164850000	-0.2417081612
chr6	164800000	165500000	0.22631214623
chr7	2950000	3700000	-0.3045245024
chr7	3650000	4400000	0.21405991657
chr7	6650000	7250000	-0.1480856169
chr7	8400000	9550000	-0.3721587797
chr7	9500000	10850000	0.18756304336
chr7	14300000	14900000	0.14409039720
chr7	15300000	15850000	-0.1418768670
chr7	15800000	16550000	0.26970487830
chr7	18750000	19750000	-0.2135854916
chr7	20600000	21200000	-0.2101259397
chr7	21400000	22450000	0.31016333769
chr7	23700000	24200000	-0.2120414996
chr7	28350000	28800000	-0.1620959637
chr7	28750000	29400000	0.17437250662
chr7	31000000	31800000	-0.2665992133
chr7	31750000	32450000	0.25460930095
chr7	34250000	34750000	-0.1719636747
chr7	36700000	37350000	-0.2618115752
chr7	45200000	45650000	-0.1638874962
chr7	48250000	49500000	-0.4111439872
chr7	49450000	50400000	0.21378660092
chr7	51700000	53000000	-0.4132880130
chr7	53450000	54600000	0.36607043869
chr7	56250000	56900000	-0.2648384344
chr7	66900000	68700000	-0.3792319235
chr7	71300000	72000000	0.28450492351
chr7	71950000	72600000	0.29005848380
chr7	77750000	78350000	-0.1813603229
chr7	85450000	86100000	-0.2157657958
chr7	86050000	86850000	0.26774722854

chr7	88050000	88950000	-0.3065727328
chr7	88900000	89750000	0.2558846620
chr7	96950000	97450000	0.22652554801
chr7	103150000	103650000	-0.1894725499
chr7	103600000	104350000	0.1931507312
chr7	108350000	109000000	-0.2176596184
chr7	108950000	109950000	0.1606186305
chr7	110600000	111000000	0.2081263132
chr7	112300000	113050000	-0.2185163591
chr7	115000000	115650000	0.2070683472
chr7	117950000	119250000	-0.3239105442
chr7	119200000	120350000	0.2866132490
chr7	121050000	122350000	-0.3302476804
chr7	122300000	123050000	0.2202548195
chr7	124550000	125050000	-0.1519508418
chr7	131550000	132000000	-0.2438854266
chr7	133050000	133450000	-0.1700725920
chr7	135850000	136700000	-0.1910828200
chr7	147000000	148150000	0.3455536778
chr7	152600000	153450000	-0.3436402054
chr7	153950000	154650000	0.2169060013
chr7	155600000	156050000	-0.1418401487
chr7	156000000	156400000	0.1733055608
chr7	157350000	157850000	-0.2457768546
chr7	157800000	158200000	0.1672624196
chr8	1000000	1500000	0.1619134916
chr8	2100000	2600000	-0.2050295293
chr8	5250000	6200000	0.2463254425
chr8	9100000	10000000	-0.2728685365
chr8	13550000	14900000	-0.3366390459
chr8	19450000	20200000	-0.2529016118
chr8	20150000	20950000	-0.2171825000
chr8	20900000	21650000	0.3037132478
chr8	24350000	24750000	0.1943338663
chr8	25250000	25650000	-0.1772831916
chr8	26450000	26850000	-0.1511461155
chr8	31050000	31550000	-0.2207590211
chr8	31500000	31900000	0.1452890145
chr8	35600000	36550000	0.2864768200
chr8	38900000	39450000	-0.1786573056
chr8	43100000	43600000	-0.2692324326
chr8	43800000	46100000	-0.1739511050
chr8	49900000	51050000	-0.2487154177
chr8	54050000	54500000	0.1822926147
chr8	55500000	56050000	-0.1975250785
chr8	56000000	56500000	0.1868636980

chr8	57050000	57500000	-0.2039711471
chr8	58650000	59300000	0.23889778621
chr8	64350000	65050000	-0.2621770483
chr8	65650000	66300000	0.20342871450
chr8	68400000	69200000	-0.2417588542
chr8	69150000	70200000	0.21553900262
chr8	71700000	72300000	-0.2746987636
chr8	73300000	73850000	0.21556247145
chr8	76400000	76900000	-0.1812722102
chr8	78050000	78900000	-0.1587191731
chr8	80050000	80550000	0.16441966325
chr8	82800000	83800000	-0.2966318273
chr8	87600000	88250000	-0.1876498931
chr8	91050000	91450000	-0.1510043485
chr8	92250000	92950000	-0.2150826977
chr8	93200000	94150000	0.21569661611
chr8	96400000	96900000	-0.2382942280
chr8	97800000	98400000	0.23555924028
chr8	100200000	100600000	-0.1798437711
chr8	104550000	105150000	-0.2419792962
chr8	108450000	108950000	-0.1534645273
chr8	109550000	110300000	0.24005661224
chr8	110850000	111750000	-0.3337759957
chr8	116000000	117500000	0.34762084562
chr8	121100000	121950000	-0.1496267602
chr8	121900000	122450000	0.14109531311
chr8	124500000	125050000	-0.1449140101
chr8	126750000	127250000	-0.2330543184
chr8	129650000	130200000	-0.1698098339
chr8	130150000	130650000	0.20431539330
chr8	131750000	132400000	-0.3099097615
chr8	133300000	133800000	0.18161274242
chr8	134650000	135250000	-0.2852748434
chr8	135950000	136900000	-0.2246831828
chr8	142500000	142950000	-0.2456645177
chr8	142900000	143500000	0.17615075905
chr9	950000	1600000	-0.2630236427
chr9	1550000	2150000	0.14033913981
chr9	6950000	7650000	-0.2924448716
chr9	11700000	12500000	0.20133979827
chr9	19550000	20000000	-0.2005294805
chr9	22400000	23450000	-0.4319061794
chr9	27700000	28750000	-0.1960868365
chr9	29350000	30550000	-0.2356839769
chr9	47200000	64750000	-0.2261069613
chr9	64700000	65450000	0.20262990589

chr9	72300000	72850000	0.19013675974
chr9	75900000	76450000	-0.2447869024
chr9	77800000	78300000	-0.1875110529
chr9	78250000	78750000	0.19046996584
chr9	82100000	83400000	-0.2468231783
chr9	83350000	84050000	0.15464872765
chr9	84550000	85200000	-0.1778161240
chr9	85150000	85950000	0.23131824584
chr9	86950000	87550000	-0.2235547642
chr9	87500000	88100000	0.21307529821
chr9	92250000	92650000	-0.2075411823
chr9	103400000	103850000	-0.2067578412
chr9	104450000	105550000	-0.2264668865
chr9	105500000	106600000	0.16623962839
chr9	108600000	109100000	-0.1802378450
chr9	117550000	118000000	-0.2175860140
chr9	119350000	120300000	-0.2630163177
chr9	121650000	123050000	0.35099986651
chr9	128150000	128900000	-0.1517815197
chr9	138100000	138700000	0.15468071680
chrX	2300000	2650000	0.25000504504
chrX	3850000	5150000	-0.3108784058
chrX	12100000	12700000	0.24447897928
chrX	14000000	14600000	-0.2764160573
chrX	14550000	15150000	0.18368642091
chrX	16150000	16500000	0.14267422662
chrX	17050000	17550000	-0.2420814136
chrX	20450000	21050000	-0.2358496369
chrX	21000000	21700000	0.23793840039
chrX	22250000	22900000	-0.2465387410
chrX	22850000	23450000	0.27658894699
chrX	25200000	26100000	-0.30873585310
chrX	26050000	26700000	0.16381858134
chrX	30000000	30600000	0.17804234555
chrX	31150000	31600000	-0.1646421854
chrX	38950000	39450000	0.17546443249
chrX	41450000	42800000	-0.4145531837
chrX	42750000	43600000	0.18062443513
chrX	49700000	51000000	-0.2650936703
chrX	52300000	52700000	0.21194822715
chrX	54800000	55650000	-0.2543104009
chrX	58500000	60950000	-0.2121151703
chrX	63900000	64600000	0.19567872934
chrX	65050000	65850000	-0.2648371854
chrX	66250000	67350000	0.28310377496
chrX	68500000	69000000	-0.1432743357

Supplementary Table S7: PREP1 ChIP-seq peaks in control HeLa cells.

peak	chr	start	end	pileup	SCORE (p)	Fold enrichment
1	chr1	856282	856655	18.00	13.13437	6.87792
2	chr1	875481	875724	21.00	22.47272	11.60387
3	chr1	935437	935759	47.00	64.23869	25.59357
4	chr1	1004195	1004441	24.00	26.99996	13.32998
5	chr1	1207989	1208172	17.00	15.02847	8.27595
6	chr1	1334669	1335014	128.00	225.15987	68.57209
7	chr1	2121089	2121370	29.00	32.49442	14.79214
8	chr1	3541261	3541556	48.00	65.98680	26.12677
9	chr1	3817919	3818119	13.00	11.52175	7.10893
10	chr1	6052461	6052841	165.00	293.39380	80.10943
11	chr1	6845165	6845536	108.00	167.30824	49.12040
12	chr1	7023394	7023692	40.00	41.11097	15.36156
13	chr1	7178083	7178269	28.00	17.16410	6.68511
14	chr1	7360753	7360947	16.00	12.31417	6.88616
15	chr1	7395400	7395618	23.00	15.26920	6.85029
16	chr1	7531098	7531355	13.00	9.84014	6.06806
17	chr1	7609407	7609621	23.00	19.14867	8.99213
18	chr1	7843853	7844149	62.00	82.07784	28.20401
19	chr1	8157502	8157775	32.00	24.84073	9.41915
20	chr1	8178270	8178553	42.00	42.64944	15.40204
21	chr1	8272055	8272324	46.00	41.74495	13.41516
22	chr1	8543039	8543245	12.00	9.97716	6.36384
23	chr1	8763544	8763873	55.00	63.10625	20.98164
24	chr1	8938653	8939048	225.00	385.90076	84.67592
25	chr1	9046363	9046563	18.00	17.28003	9.36836
26	chr1	9140624	9140880	23.00	21.43488	10.33659
27	chr1	9170673	9170995	42.00	43.92358	16.11090
28	chr1	9448927	9449232	58.00	76.19998	26.94478
29	chr1	9599172	9599404	17.00	17.20576	9.59759
30	chr1	10270314	10270679	159.00	294.29260	85.31190
31	chr1	10458796	10459175	118.00	203.65999	63.25642
32	chr1	11105712	11105947	17.00	17.20576	9.59759
33	chr1	11322516	11322950	169.00	279.11526	68.86160
34	chr1	11797334	11797666	36.00	42.47226	17.85572
35	chr1	11863235	11863423	13.00	8.20634	5.06794
36	chr1	11865777	11866069	25.00	25.00931	11.79486
37	chr1	12079309	12079626	57.00	56.32385	16.55487
38	chr1	12538395	12538710	19.00	18.11170	9.58381
39	chr1	12600139	12600566	85.00	129.79047	42.40414
40	chr1	12678161	12678473	34.00	39.81745	17.13342
41	chr1	12678884	12679059	16.00	11.45212	6.36943
42	chr1	14075760	14075948	14.00	13.33873	7.99799
43	chr1	15433209	15433423	14.00	13.33873	7.99799
44	chr1	15649580	15649852	35.00	34.26693	13.48820
45	chr1	16514460	16514655	12.00	9.35636	5.97708
46	chr1	16947594	16947818	20.00	15.72746	7.86812
47	chr1	17445917	17446198	34.00	38.62149	16.42488

48	chr1	17802198	17802573	71.00	108.32056	38.39035
49	chr1	19923196	19923394	13.00	12.10322	7.46479
50	chr1	20135918	20136127	13.00	12.10322	7.46479
51	chr1	20208675	20208852	15.00	10.44156	5.99476
52	chr1	21636845	21637060	28.00	27.98681	12.49005
53	chr1	21650773	21651007	28.00	31.20390	14.40338
54	chr1	21655954	21656129	20.00	15.72746	7.86812
55	chr1	21934292	21934759	55.00	77.51783	29.31007
56	chr1	21974355	21974730	25.00	21.51895	9.74148
57	chr1	22091144	22091491	32.00	30.30064	12.36418
58	chr1	22275259	22275434	11.00	9.53247	6.28073
59	chr1	22988831	22989102	27.00	31.46828	14.92958
60	chr1	23076974	23077236	28.00	25.58257	11.08736
61	chr1	23670703	23671106	173.00	326.23587	92.77669
62	chr1	23694708	23695095	108.00	182.83191	58.11873
63	chr1	23854846	23855085	23.00	14.25687	6.33151
64	chr1	24117398	24117625	15.00	12.67947	7.35640
65	chr1	24372837	24373127	12.00	7.57257	4.87074
66	chr1	24373751	24373948	27.00	23.95451	10.49082
67	chr1	24741996	24742427	254.00	403.97852	72.78436
68	chr1	25256684	25257007	81.00	127.78880	43.72235
69	chr1	25756998	25757495	198.00	384.46805	106.10667
70	chr1	26704923	26705276	18.00	16.23862	8.73573
71	chr1	27191675	27191936	22.00	24.10503	12.26359
72	chr1	27247974	27248245	32.00	39.21360	17.59558
73	chr1	27320006	27320280	36.00	45.64589	19.72838
74	chr1	27339273	27339541	30.00	22.61202	8.84830
75	chr1	27816626	27816910	43.00	54.33144	21.69514
76	chr1	27866982	27867190	19.00	14.62602	7.49344
77	chr1	27998541	27998825	34.00	42.30803	18.60483
78	chr1	28052217	28052509	43.00	57.33764	23.46077
79	chr1	28562476	28562686	24.00	13.49383	5.76302
80	chr1	28648881	28649143	19.00	17.47089	9.19550
81	chr1	28764497	28764738	18.00	16.23862	8.73573
82	chr1	29213390	29213715	73.00	91.77285	27.72574
83	chr1	29508441	29508725	31.00	29.00390	11.98951
84	chr1	30463902	30464211	22.00	24.10503	12.26359
85	chr1	31231660	31231922	30.00	36.07370	16.52918
86	chr1	31625289	31625489	14.00	13.33873	7.99799
87	chr1	31628368	31628552	12.00	10.89743	6.93159
88	chr1	32015201	32015448	31.00	23.71973	9.13372
89	chr1	32110386	32110799	202.00	357.78433	87.43036
90	chr1	32178226	32178414	10.00	7.87663	5.42379
91	chr1	32254164	32254424	25.00	27.36219	13.20229
92	chr1	32400345	32400571	13.00	12.10322	7.46479
93	chr1	32528079	32528254	8.00	6.39696	4.78410
94	chr1	32577572	32577766	12.00	10.89743	6.93159
95	chr1	32645563	32645739	13.00	12.10322	7.46479

96	chr1	33282982	33283309	55.00	78.45982	29.85916
97	chr1	33414528	33414793	33.00	31.61030	12.73885
98	chr1	33502337	33502773	256.00	523.83636	136.61261
99	chr1	33592885	33593231	48.00	65.98680	26.12677
100	chr1	33686531	33686794	14.00	7.15249	4.28143
101	chr1	33937923	33938299	97.00	156.35924	50.13639
102	chr1	35658639	35659023	169.00	219.30923	39.18857
103	chr1	36274518	36274799	33.00	34.90736	14.64351
104	chr1	36616359	36616683	43.00	56.30113	22.85366
105	chr1	37740926	37741178	14.00	13.33873	7.99799
106	chr1	38397403	38397748	105.00	176.25885	56.34606
107	chr1	38512619	38512813	17.00	17.20576	9.59759
108	chr1	38604316	38604516	18.00	13.54559	7.11877
109	chr1	39325388	39325682	76.00	117.98460	41.05635
110	chr1	39649604	39649828	12.00	7.57257	4.87074
111	chr1	40157024	40157327	53.00	74.85551	28.79276
112	chr1	40358385	40358736	10.00	8.48181	5.80194
113	chr1	40364697	40365004	54.00	61.58029	20.60697
114	chr1	41174413	41175080	72.00	110.24171	38.92355
115	chr1	41445431	41445730	50.00	69.50916	27.19317
116	chr1	41849148	41849487	51.00	57.04987	19.48295
117	chr1	41940236	41940824	22.00	17.98963	8.61746
118	chr1	42249002	42249177	13.00	11.18770	6.90300
119	chr1	42383665	42383917	20.00	21.28387	11.19719
120	chr1	43147838	43148176	127.00	165.65082	36.53490
121	chr1	43311978	43312350	177.00	323.09625	87.76671
122	chr1	43855408	43855606	17.00	15.02847	8.27595
123	chr1	44100414	44100694	33.00	40.70874	18.07326
124	chr1	44115877	44116211	111.00	158.09872	41.96328
125	chr1	44619791	44620077	32.00	37.58385	16.63271
126	chr1	44792088	44792357	10.00	7.40610	5.12677
127	chr1	44820806	44821187	152.00	278.51715	81.57951
128	chr1	44951307	44951517	17.00	13.40451	7.29123
129	chr1	45140154	45140543	218.00	404.18787	100.69077
130	chr1	45265572	45265831	23.00	25.47769	12.75760
131	chr1	45452075	45452449	65.00	78.76765	24.72836
132	chr1	45956824	45957201	105.00	169.22568	52.26557
133	chr1	46955242	46955606	89.00	143.49231	47.84099
134	chr1	47779712	47780195	91.00	127.36732	37.26628
135	chr1	48002312	48002527	14.00	13.33873	7.99799
136	chr1	49085339	49085658	46.00	62.03695	24.79009
137	chr1	52082861	52083368	42.00	50.26844	19.77033
138	chr1	52869252	52869532	20.00	15.72746	7.86812
139	chr1	53280300	53280498	15.00	12.76090	7.40642
140	chr1	53663105	53663365	27.00	26.51668	11.98356
141	chr1	54104970	54105276	68.00	71.80498	19.69459
142	chr1	54204843	54205094	31.00	29.55418	12.30341
143	chr1	54665598	54666014	250.00	463.86633	107.42403

144	chr1	54738709	54739068	103.00	125.64062	29.68460
145	chr1	54739555	54739821	38.00	18.88381	5.70050
146	chr1	54871980	54872302	51.00	57.04987	19.48295
147	chr1	55240859	55241165	48.00	52.59385	18.35894
148	chr1	55370525	55370818	24.00	22.71256	10.76729
149	chr1	55678488	55678663	15.00	11.92080	6.89106
150	chr1	56215974	56216293	40.00	42.14116	15.94381
151	chr1	57104006	57104227	18.00	16.14215	8.67713
152	chr1	57176463	57176638	13.00	9.42122	5.80920
153	chr1	59058279	59058504	24.00	16.27042	7.13572
154	chr1	59193100	59193304	14.00	9.45699	5.62008
155	chr1	59388265	59388608	18.00	14.36679	7.60580
156	chr1	60280294	60280604	30.00	33.42776	14.96020
157	chr1	60747184	60747359	16.00	13.03207	7.32175
158	chr1	61543554	61543922	113.00	151.60097	37.30113
159	chr1	61886616	61886791	9.00	7.13674	5.11596
160	chr1	61952462	61952734	22.00	17.98963	8.61746
161	chr1	61962183	61962410	20.00	16.00201	8.02878
162	chr1	62352952	62353460	21.00	16.36705	7.96391
163	chr1	62542125	62542325	16.00	14.01657	7.92319
164	chr1	62773738	62774024	15.00	7.96262	4.56686
165	chr1	64059825	64060202	54.00	74.61894	28.13777
166	chr1	64386045	64386435	62.00	73.99586	23.60435
167	chr1	64935692	64935965	40.00	34.24459	11.70258
168	chr1	66228100	66228286	14.00	11.54338	6.89663
169	chr1	66648184	66648417	21.00	10.91615	5.07146
170	chr1	66727869	66728147	22.00	17.98963	8.61746
171	chr1	70895078	70895253	13.00	8.50004	5.24541
172	chr1	78005610	78005858	26.00	24.83285	11.34169
173	chr1	85527789	85528139	87.00	100.27099	25.11774
174	chr1	85666572	85666898	58.00	69.46429	23.07533
175	chr1	85791651	85791826	14.00	10.03399	5.96948
176	chr1	86043571	86043820	25.00	20.94268	9.41189
177	chr1	87240000	87240446	18.00	14.14570	7.47397
178	chr1	87793936	87794253	74.00	101.59186	32.71576
179	chr1	87857929	87858136	21.00	18.35309	9.12874
180	chr1	88172539	88172716	15.00	10.58893	6.08300
181	chr1	88184128	88184423	43.00	32.80746	10.14292
182	chr1	88354164	88354454	15.00	11.44216	6.59887
183	chr1	88395630	88395857	14.00	9.45699	5.62008
184	chr1	88864150	88864433	35.00	39.54578	16.55191
185	chr1	89450967	89451158	23.00	15.26920	6.85029
186	chr1	90355809	90356023	14.00	11.46841	6.85037
187	chr1	90384778	90384959	18.00	12.53419	6.53070
188	chr1	92035060	92035248	15.00	10.64585	6.11716
189	chr1	92351622	92351874	20.00	12.37394	5.99401
190	chr1	94130124	94130424	35.00	28.27971	10.27544
191	chr1	94157563	94157757	15.00	11.06094	6.36745

192	chr1	94210222	94210404	16.00	12.80798	7.18540
193	chr1	94701300	94701538	29.00	27.78662	12.00916
194	chr1	94992993	94993259	41.00	46.61858	18.08904
195	chr1	95085116	95085302	20.00	12.37394	5.99401
196	chr1	95249169	95249443	32.00	35.65198	15.48631
197	chr1	98315511	98315805	29.00	30.20530	13.43048
198	chr1	98495998	98496290	43.00	45.34496	16.48558
199	chr1	100111560	100111840	23.00	15.26920	6.85029
200	chr1	109372077	109372353	30.00	23.39972	9.25386
201	chr1	109419307	109419493	12.00	9.35636	5.97708
202	chr1	109584594	109584799	14.00	12.80671	7.67394
203	chr1	109757438	109757613	18.00	8.51003	4.37990
204	chr1	109940525	109940885	150.00	243.01343	62.65636
205	chr1	109941990	109942207	15.00	11.24842	6.48109
206	chr1	110193265	110193469	19.00	12.97326	6.54406
207	chr1	110326010	110326336	57.00	65.88917	21.56913
208	chr1	110881310	110882130	174.00	306.30325	80.46066
209	chr1	111423629	111423862	24.00	25.02511	12.15080
210	chr1	111888753	111889008	22.00	14.28555	6.56486
211	chr1	112938605	112938999	197.00	292.46634	56.51492
212	chr1	113161179	113161517	44.00	46.77608	16.86025
213	chr1	113162139	113162466	22.00	17.48414	8.32590
214	chr1	113472685	113472860	13.00	8.50004	5.24541
215	chr1	113497919	113498187	13.00	10.87353	6.70867
216	chr1	113932798	113933006	20.00	16.23600	8.16634
217	chr1	114220525	114220726	30.00	26.01022	10.65535
218	chr1	114657176	114657395	24.00	16.29664	7.14934
219	chr1	115212627	115212971	71.00	76.16368	20.55088
220	chr1	115722200	115722514	42.00	42.79692	15.48349
221	chr1	116314444	116314657	26.00	22.12900	9.77388
222	chr1	116653107	116653282	29.00	15.56141	5.79990
223	chr1	116832955	116833307	99.00	90.16469	16.64725
224	chr1	116919092	116919305	14.00	8.90357	5.28933
225	chr1	117253935	117254116	21.00	14.41736	6.86839
226	chr1	117602482	117602903	21.00	13.32019	6.27944
227	chr1	117664299	117664974	205.00	307.72937	58.79835
228	chr1	144931817	144932125	62.00	52.26850	13.07414
229	chr1	145382693	145382887	22.00	17.98963	8.61746
230	chr1	145396972	145397177	17.00	12.48723	6.74410
231	chr1	145470135	145470382	24.00	16.27042	7.13572
232	chr1	145476813	145477198	164.00	272.46729	68.88537
233	chr1	147807133	147807338	17.00	17.15686	9.56820
234	chr1	149856167	149856465	38.00	42.14300	16.79697
235	chr1	150039290	150039506	24.00	18.22131	8.18007
236	chr1	150208402	150208826	168.00	201.58466	32.67279
237	chr1	150459572	150460059	276.00	414.32651	63.85432
238	chr1	150487882	150488234	84.00	108.28603	30.76963
239	chr1	150601475	150602372	256.00	376.25137	59.24390

240	chr1	150669580	150670094	206.00	345.73703	77.55714
241	chr1	150898530	150898842	60.00	47.64320	11.79314
242	chr1	151042859	151043070	19.00	11.44765	5.70858
243	chr1	151170310	151170529	23.00	12.61686	5.53250
244	chr1	151254284	151254887	273.00	483.89078	99.18682
245	chr1	151467230	151467495	29.00	25.78011	10.85987
246	chr1	151762931	151763306	211.00	379.61249	92.47653
247	chr1	151918980	151919185	18.00	10.54228	5.42315
248	chr1	151960970	151961223	44.00	39.25551	12.86882
249	chr1	152007183	152007456	28.00	22.72984	9.48888
250	chr1	152020522	152020738	28.00	21.17108	8.65684
251	chr1	153509184	153509421	23.00	12.61686	5.53250
252	chr1	153581699	153582018	52.00	58.55195	19.85763
253	chr1	153669791	153670060	16.00	11.45212	6.36943
254	chr1	153680976	153681327	35.00	43.66731	18.98815
255	chr1	153700168	153700513	122.00	204.04678	60.64778
256	chr1	153895415	153895869	242.00	326.80911	46.97922
257	chr1	153935972	153936503	134.00	189.05217	44.17239
258	chr1	153940089	153940496	231.00	422.07639	99.92041
259	chr1	154909349	154909982	50.00	46.93057	14.55687
260	chr1	154955500	154955970	294.00	530.27209	106.78873
261	chr1	154974520	154974811	74.00	97.18576	30.20045
262	chr1	155057708	155058107	148.00	231.41103	57.61310
263	chr1	155099291	155099466	16.00	12.59157	7.05403
264	chr1	155196759	155196984	24.00	13.80257	5.90851
265	chr1	155197216	155197611	218.00	332.81662	62.50892
266	chr1	155214352	155214653	102.00	129.61115	32.15655
267	chr1	155278260	155278531	42.00	31.68671	9.91240
268	chr1	155904141	155904507	89.00	118.86767	33.72050
269	chr1	156163680	156163944	35.00	32.26501	12.37396
270	chr1	156182544	156182924	114.00	169.90048	46.58285
271	chr1	156307941	156308359	209.00	352.03015	78.68116
272	chr1	156426982	156427269	25.00	21.51895	9.74148
273	chr1	156470465	156470802	138.00	215.81740	55.97149
274	chr1	156597330	156597571	19.00	16.50842	8.61383
275	chr1	156682518	156682728	25.00	20.94268	9.41189
276	chr1	159750380	159750718	15.00	11.50819	6.63908
277	chr1	159885943	159886438	16.00	10.55690	5.84326
278	chr1	160990901	160991288	85.00	97.18379	24.54688
279	chr1	161195449	161195885	165.00	232.87622	47.38120
280	chr1	161720040	161720301	30.00	27.72049	11.61484
281	chr1	162317143	162317418	18.00	10.54228	5.42315
282	chr1	162531063	162531325	35.00	33.45447	13.03185
283	chr1	163291606	163291821	19.00	16.69150	8.72420
284	chr1	164527933	164528313	187.00	306.32986	70.43837
285	chr1	164618136	164618345	19.00	11.44765	5.70858
286	chr1	165606638	165606866	17.00	7.75030	4.14938
287	chr1	167132239	167132414	16.00	12.38238	6.92737

288	chr1	167690962	167691312	208.00	352.85934	79.90543
289	chr1	168107464	168107733	21.00	16.84896	8.24279
290	chr1	168148426	168148855	28.00	20.43819	8.27744
291	chr1	172320964	172321169	16.00	11.08796	6.15393
292	chr1	172418258	172418456	18.00	11.00162	5.67172
293	chr1	173091641	173091945	14.00	12.71320	7.61671
294	chr1	173159790	173159974	13.00	11.18770	6.90300
295	chr1	175440262	175440463	13.00	11.18770	6.90300
296	chr1	177178533	177178823	35.00	41.35590	17.62295
297	chr1	177980633	177980864	18.00	17.28003	9.36836
298	chr1	178207821	178208085	18.00	17.62334	9.57641
299	chr1	179050793	179051054	12.00	7.57257	4.87074
300	chr1	180136790	180136974	18.00	10.54228	5.42315
301	chr1	180482800	180482975	18.00	13.13437	6.87792
302	chr1	181057713	181057896	18.00	13.54559	7.11877
303	chr1	181081752	181081934	13.00	10.29677	6.35108
304	chr1	181998714	181999072	92.00	145.13576	46.87400
305	chr1	183129091	183129328	21.00	16.84896	8.24279
306	chr1	184591415	184591652	25.00	25.27586	11.95416
307	chr1	184723954	184724251	26.00	27.06765	12.67062
308	chr1	185285253	185285634	126.00	208.61957	60.43188
309	chr1	192579911	192580096	18.00	13.54559	7.11877
310	chr1	197115697	197116091	96.00	145.69539	44.59819
311	chr1	197409812	197410100	41.00	42.51218	15.73623
312	chr1	197411215	197411424	19.00	11.44765	5.70858
313	chr1	198082656	198082904	24.00	23.93016	11.49438
314	chr1	198126663	198126969	80.00	107.87117	32.81053
315	chr1	198128096	198128479	182.00	298.65631	69.96505
316	chr1	198906500	198906714	18.00	18.23309	9.94449
317	chr1	199728711	199728988	17.00	12.48723	6.74410
318	chr1	200379081	200379435	155.00	268.90567	73.71609
319	chr1	201123489	201123840	125.00	196.67581	54.26712
320	chr1	201139639	201139837	21.00	10.91615	5.07146
321	chr1	201430249	201430593	34.00	38.05911	16.09213
322	chr1	201798135	201798355	19.00	18.80980	10.00636
323	chr1	202252505	202252736	21.00	16.84896	8.24279
324	chr1	202896042	202896256	18.00	13.54559	7.11877
325	chr1	204257326	204257690	22.00	22.16230	11.09950
326	chr1	204367784	204367970	19.00	17.47089	9.19550
327	chr1	205196835	205197150	105.00	176.55830	56.51913
328	chr1	205243563	205243738	12.00	10.28098	6.55228
329	chr1	205267590	205267892	22.00	20.17485	9.90590
330	chr1	205416603	205416900	42.00	42.94567	15.56581
331	chr1	205464666	205464941	29.00	30.20530	13.43048
332	chr1	205782194	205782545	116.00	190.91872	57.27458
333	chr1	206680620	206680888	37.00	39.15570	15.48472
334	chr1	207063599	207063951	69.00	86.18684	26.76259
335	chr1	207082738	207082922	13.00	11.10738	6.85337

336	chr1	207921462	207921637	11.00	7.75405	5.16830
337	chr1	207996007	207996218	18.00	13.54559	7.11877
338	chr1	208263774	208264127	85.00	111.96949	32.22181
339	chr1	208357792	208358039	21.00	16.84896	8.24279
340	chr1	209826548	209826745	17.00	12.71960	6.88181
341	chr1	210412653	210412963	30.00	32.07711	14.15743
342	chr1	211721954	211722140	15.00	12.92712	7.50851
343	chr1	212457976	212458809	120.00	186.81456	52.11366
344	chr1	212659388	212659596	28.00	20.43819	8.27744
345	chr1	214612735	214613034	25.00	25.27586	11.95416
346	chr1	214640147	214640372	9.00	7.32469	5.23394
347	chr1	219347106	219347298	16.00	15.89157	9.06439
348	chr1	220078415	220078631	15.00	13.93247	8.12449
349	chr1	220446103	220446278	12.00	7.57257	4.87074
350	chr1	220961685	220961860	16.00	13.03207	7.32175
351	chr1	220963306	220963580	18.00	16.33637	8.79512
352	chr1	221464418	221464607	15.00	12.67947	7.35640
353	chr1	221567702	221567968	12.00	7.57257	4.87074
354	chr1	221915298	221915688	138.00	184.66429	39.67461
355	chr1	222790971	222791225	39.00	46.69821	19.03366
356	chr1	223103531	223103723	19.00	15.98418	8.29885
357	chr1	224075873	224076190	55.00	71.43149	25.74741
358	chr1	224701498	224701750	19.00	16.50842	8.61383
359	chr1	226374451	226374782	13.00	12.10322	7.46479
360	chr1	226714475	226714802	50.00	55.55605	19.10828
361	chr1	226867913	226868209	33.00	36.58465	15.63236
362	chr1	227511697	227511882	16.00	13.84165	7.81618
363	chr1	227539224	227539405	11.00	8.94684	5.91686
364	chr1	227750976	227751347	134.00	214.64015	58.14334
365	chr1	228327492	228327839	49.00	52.92723	18.09978
366	chr1	228353069	228353303	16.00	15.28459	8.69713
367	chr1	228354163	228354442	29.00	26.45082	11.24017
368	chr1	228996885	228997158	28.00	25.19533	10.86549
369	chr1	229038817	229038997	15.00	12.67947	7.35640
370	chr1	229339283	229339592	52.00	67.87436	25.21960
371	chr1	230202427	230202634	34.00	32.14368	12.66985
372	chr1	231523770	231524041	22.00	17.98963	8.61746
373	chr1	232765861	232766180	55.00	74.27768	27.41347
374	chr1	233248690	233249031	37.00	46.64917	19.88898
375	chr1	233843214	233843448	21.00	13.32019	6.27944
376	chr1	234276771	234276946	14.00	10.20871	6.07602
377	chr1	234651514	234651722	21.00	13.32019	6.27944
378	chr1	234908132	234908405	28.00	26.13769	11.40764
379	chr1	235491533	235491732	11.00	9.37873	6.18563
380	chr1	235530537	235530818	38.00	31.82546	11.13173
381	chr1	235796420	235796595	20.00	8.40177	4.05993
382	chr1	236695053	236695240	18.00	13.54559	7.11877
383	chr1	236958423	236958715	32.00	33.49657	14.21282

384	chr1	244211515	244211834	57.00	79.94727	29.67256
385	chr1	245237664	245237887	19.00	17.47089	9.19550
386	chr1	246890372	246890597	16.00	11.67045	6.49949
387	chr1	247171291	247171696	221.00	439.25024	118.37025
388	chr1	247242009	247242359	98.00	155.22035	48.81407
389	chr1	247267611	247267954	134.00	229.24805	66.56464
390	chr1	247275629	247275931	17.00	9.65886	5.13772
391	chr1	247374094	247374439	80.00	89.55466	23.11974
392	chr10	180090	180395	17.00	17.20576	9.59759
393	chr10	557062	557310	15.00	13.36375	7.77651
394	chr10	1030381	1030634	21.00	16.84896	8.24279
395	chr10	1541545	1541770	14.00	12.71320	7.61671
396	chr10	3240073	3240248	12.00	10.52193	6.70110
397	chr10	3263048	3263223	11.00	9.72353	6.39839
398	chr10	3853451	3853707	17.00	9.65886	5.13772
399	chr10	3978212	3978411	12.00	10.89743	6.93159
400	chr10	4396385	4396592	19.00	18.57014	9.86143
401	chr10	5855318	5855678	144.00	260.65823	77.31390
402	chr10	5931884	5932287	213.00	420.07269	114.10466
403	chr10	6778964	6779242	27.00	29.57795	13.80600
404	chr10	6954412	6954696	25.00	21.51895	9.74148
405	chr10	7302526	7302821	24.00	24.19280	11.65174
406	chr10	7353164	7353728	37.00	36.97105	14.23754
407	chr10	7396631	7396911	29.00	29.34339	12.92074
408	chr10	8744965	8745177	15.00	14.60207	8.53119
409	chr10	12084824	12085057	23.00	25.54366	12.79678
410	chr10	13656872	13657132	19.00	18.57014	9.86143
411	chr10	16423235	16423478	24.00	20.32534	9.36681
412	chr10	18272975	18273212	16.00	14.96948	8.50540
413	chr10	18448716	18448916	11.00	9.72353	6.39839
414	chr10	21463337	21463543	18.00	18.10711	9.86863
415	chr10	21484183	21484396	18.00	13.54559	7.11877
416	chr10	21583038	21583295	20.00	15.72746	7.86812
417	chr10	21807147	21807449	27.00	23.95451	10.49082
418	chr10	22292691	22292905	14.00	13.33873	7.99799
419	chr10	22519407	22519628	14.00	13.29834	7.97350
420	chr10	22716065	22716342	22.00	17.98963	8.61746
421	chr10	22718187	22718423	16.00	14.38123	8.14624
422	chr10	24753033	24753322	57.00	56.32385	16.55487
423	chr10	25178546	25178878	41.00	53.94481	22.39437
424	chr10	25463420	25463820	94.00	153.87737	50.65394
425	chr10	26901395	26901708	13.00	12.10322	7.46479
426	chr10	27168810	27169038	20.00	12.37394	5.99401
427	chr10	28491216	28491391	16.00	13.03207	7.32175
428	chr10	28491859	28492137	31.00	29.43344	12.23433
429	chr10	28543640	28543824	16.00	14.76748	8.38221
430	chr10	30101023	30101280	27.00	19.37298	7.99201
431	chr10	30508853	30509145	30.00	36.07370	16.52918

432	chr10	30823464	30823669	14.00	13.33873	7.99799
433	chr10	31422925	31423100	13.00	12.10322	7.46479
434	chr10	31435927	31436151	13.00	8.50004	5.24541
435	chr10	32229919	32230167	20.00	10.09393	4.84094
436	chr10	32438281	32438456	16.00	14.76748	8.38221
437	chr10	33248182	33248357	14.00	9.45699	5.62008
438	chr10	33553012	33553265	28.00	29.40866	13.33348
439	chr10	42863387	42863767	157.00	289.77155	84.24550
440	chr10	43779895	43780091	13.00	12.10322	7.46479
441	chr10	45474125	45474517	144.00	260.24768	77.07716
442	chr10	48376704	48376879	13.00	11.18770	6.90300
443	chr10	50787766	50787966	18.00	16.23862	8.73573
444	chr10	52178103	52178460	60.00	87.60279	32.52516
445	chr10	52364633	52364875	16.00	15.72948	8.96663
446	chr10	61438611	61438908	20.00	21.28387	11.19719
447	chr10	61661222	61661499	20.00	21.28387	11.19719
448	chr10	62578024	62578211	12.00	10.68938	6.80412
449	chr10	63044066	63044253	12.00	10.89743	6.93159
450	chr10	63393065	63393324	18.00	17.98321	9.79391
451	chr10	63395911	63396111	15.00	14.55884	8.50506
452	chr10	63521117	63521386	25.00	25.27586	11.95416
453	chr10	65228816	65228991	12.00	10.89743	6.93159
454	chr10	68386944	68387227	37.00	47.17789	20.19953
455	chr10	69913361	69913631	27.00	31.46828	14.92958
456	chr10	71211055	71211401	109.00	178.00037	54.63350
457	chr10	72137950	72138245	51.00	57.04987	19.48295
458	chr10	73489081	73489401	48.00	53.26324	18.73381
459	chr10	73519110	73519366	28.00	31.38161	14.50922
460	chr10	73527048	73527223	14.00	12.18211	7.29048
461	chr10	73528624	73528959	49.00	45.62112	14.27144
462	chr10	73620138	73620331	18.00	13.54559	7.11877
463	chr10	73631151	73631346	14.00	13.33873	7.99799
464	chr10	73829008	73829183	12.00	10.28098	6.55228
465	chr10	73858589	73858915	44.00	39.20615	12.84430
466	chr10	74004003	74004261	24.00	23.93016	11.49438
467	chr10	74087709	74087965	19.00	19.90304	10.66399
468	chr10	74425697	74425872	12.00	10.89743	6.93159
469	chr10	74450146	74450512	51.00	71.28306	27.72637
470	chr10	74451561	74451992	124.00	216.51817	66.44582
471	chr10	74877804	74877979	15.00	12.67947	7.35640
472	chr10	75361220	75361395	12.00	10.89743	6.93159
473	chr10	76585784	76586455	236.00	469.86609	123.09811
474	chr10	77054520	77054963	53.00	60.06214	20.23230
475	chr10	77055102	77055285	11.00	9.72353	6.39839
476	chr10	77160812	77161013	15.00	14.60207	8.53119
477	chr10	77162274	77162501	16.00	15.89157	9.06439
478	chr10	77228810	77229098	18.00	13.54559	7.11877
479	chr10	79789159	79789576	184.00	351.67859	98.64188

480	chr10	80166716	80167094	130.00	193.50551	49.08206
481	chr10	80733348	80733534	20.00	15.72746	7.86812
482	chr10	88407546	88407839	30.00	27.72049	11.61484
483	chr10	89483321	89483543	13.00	8.50004	5.24541
484	chr10	90146098	90146291	18.00	13.79201	7.26413
485	chr10	90233860	90234052	20.00	20.39366	10.66339
486	chr10	90976875	90977088	13.00	12.06567	7.44193
487	chr10	92681049	92681227	20.00	15.26912	7.60191
488	chr10	92728503	92728811	45.00	52.72791	19.81181
489	chr10	93349090	93349428	66.00	68.93024	19.12374
490	chr10	93358286	93358582	30.00	30.71417	13.35143
491	chr10	93376868	93377065	14.00	7.15249	4.28143
492	chr10	94147140	94147315	16.00	15.89157	9.06439
493	chr10	94334624	94334865	15.00	14.60207	8.53119
494	chr10	95222705	95223015	32.00	30.30064	12.36418
495	chr10	95230717	95231015	24.00	26.09272	12.78990
496	chr10	96161924	96162270	44.00	46.77608	16.86025
497	chr10	96995710	96996011	32.00	39.12203	17.54170
498	chr10	99052323	99052661	86.00	137.47931	46.24629
499	chr10	99241079	99241318	29.00	26.85215	11.46968
500	chr10	99258437	99258765	47.00	60.95343	23.66743
501	chr10	99334385	99334830	18.00	16.23862	8.73573
502	chr10	99399965	99400277	46.00	59.28403	23.17436
503	chr10	99446928	99447160	31.00	23.71973	9.13372
504	chr10	99448052	99448227	20.00	8.40177	4.05993
505	chr10	100029335	100029628	24.00	13.49383	5.76302
506	chr10	100163793	100163981	12.00	9.35636	5.97708
507	chr10	101190365	101190643	27.00	23.33103	10.13588
508	chr10	101380153	101380554	135.00	203.02882	50.95542
509	chr10	101418963	101419381	85.00	132.52682	43.99725
510	chr10	101754106	101754285	12.00	10.86272	6.91037
511	chr10	102505122	102505329	16.00	15.89157	9.06439
512	chr10	102672486	102672789	53.00	67.79759	24.66133
513	chr10	102790782	102791219	224.00	383.76910	84.30125
514	chr10	102802426	102802792	161.00	278.25226	74.48358
515	chr10	103127445	103127868	23.00	25.54366	12.79678
516	chr10	104004885	104005600	137.00	206.86060	51.70476
517	chr10	104006040	104006380	11.00	9.72353	6.39839
518	chr10	104261717	104262230	265.00	476.39066	101.69783
519	chr10	104262798	104262998	18.00	18.54332	10.13079
520	chr10	104263422	104263832	170.00	315.25949	88.81763
521	chr10	104503374	104503757	155.00	265.45590	71.72493
522	chr10	104677810	104678198	116.00	199.73347	62.38433
523	chr10	104944152	104944327	12.00	10.89743	6.93159
524	chr10	105001538	105001718	17.00	9.65886	5.13772
525	chr10	105128371	105128546	16.00	11.45212	6.36943
526	chr10	105300539	105300806	17.00	17.15686	9.56820
527	chr10	105881681	105882031	22.00	22.84051	11.50731

528	chr10	106090326	106090501	14.00	9.45699	5.62008
529	chr10	112064741	112064920	12.00	10.05135	6.40993
530	chr10	112170079	112170369	51.00	64.49141	23.74795
531	chr10	112389507	112389714	18.00	13.54559	7.11877
532	chr10	112534786	112534970	14.00	13.09665	7.85091
533	chr10	112625844	112626127	32.00	35.29685	15.27574
534	chr10	112679030	112679205	16.00	13.03207	7.32175
535	chr10	114501770	114502082	55.00	68.62959	24.11872
536	chr10	114709880	114710203	34.00	42.40530	18.66198
537	chr10	114760786	114760961	15.00	14.13432	8.24751
538	chr10	115707602	115707810	14.00	13.29834	7.97350
539	chr10	118707341	118707610	31.00	29.00390	11.98951
540	chr10	119221233	119221584	64.00	77.17039	24.35369
541	chr10	121631653	121631882	19.00	19.90304	10.66399
542	chr10	124913634	124913980	95.00	138.93106	41.34638
543	chr10	125770340	125770748	24.00	26.99996	13.32998
544	chr10	125853021	125853278	26.00	29.96288	14.39638
545	chr10	127227613	127227788	11.00	9.69164	6.37880
546	chr10	127407951	127408210	20.00	21.28387	11.19719
547	chr10	127936168	127936435	30.00	27.72049	11.61484
548	chr10	130008415	130008613	17.00	17.03367	9.49408
549	chr10	131909305	131909639	68.00	102.59337	36.79076
550	chr10	132416280	132416580	25.00	28.40159	13.82073
551	chr10	132435394	132435646	33.00	25.97464	9.70458
552	chr10	132734602	132734784	14.00	11.61937	6.94352
553	chr10	132735190	132735543	23.00	22.98148	11.26277
554	chr10	134302726	134303024	28.00	31.93140	14.83628
555	chr10	134350830	134351134	21.00	22.68483	11.73039
556	chr10	134775736	134775911	14.00	10.83560	6.46037
557	chr10	134822811	134822986	12.00	5.61615	3.71058
558	chr11	535521	535843	73.00	112.16877	39.45675
559	chr11	842718	842992	36.00	45.64589	19.72838
560	chr11	3400322	3400701	157.00	198.99757	36.42232
561	chr11	3818680	3819070	171.00	312.95584	86.69170
562	chr11	4115838	4116120	34.00	27.12109	9.99001
563	chr11	6411560	6411743	14.00	11.54338	6.89663
564	chr11	7625890	7626144	26.00	22.72887	10.11615
565	chr11	8090286	8090530	28.00	28.67591	12.89799
566	chr11	9482128	9482659	166.00	298.61081	82.34293
567	chr11	9587619	9587883	16.00	11.45212	6.36943
568	chr11	10350435	10350661	26.00	25.95772	12.00848
569	chr11	10830374	10830679	47.00	53.70549	19.44328
570	chr11	10879476	10879858	209.00	339.86456	72.18142
571	chr11	10921157	10921771	31.00	29.00390	11.98951
572	chr11	11643433	11643776	89.00	134.86502	42.82574
573	chr11	12031174	12031381	18.00	10.54228	5.42315
574	chr11	12088489	12088721	14.00	13.09665	7.85091
575	chr11	12989649	12989998	50.00	55.55605	19.10828

576	chr11	13139828	13140040	14.00	11.46841	6.85037
577	chr11	13461690	13462109	146.00	254.91139	72.48150
578	chr11	14913743	14914102	45.00	48.21673	17.23492
579	chr11	16301909	16302171	22.00	24.10503	12.26359
580	chr11	16634783	16634977	13.00	10.43506	6.43685
581	chr11	16946383	16946577	21.00	16.36705	7.96391
582	chr11	17705372	17705547	14.00	9.97699	5.93479
583	chr11	18001397	18001699	58.00	45.31947	11.40648
584	chr11	19138491	19138814	60.00	87.60279	32.52516
585	chr11	20134106	20134307	15.00	14.60207	8.53119
586	chr11	22647307	22647642	66.00	98.80634	35.72436
587	chr11	27466347	27466528	12.00	10.89743	6.93159
588	chr11	27486164	27486346	13.00	12.10322	7.46479
589	chr11	27494346	27494565	17.00	17.20576	9.59759
590	chr11	28070942	28071266	42.00	55.63624	22.92757
591	chr11	32197821	32198147	43.00	45.34496	16.48558
592	chr11	34272674	34273065	72.00	90.12595	27.35107
593	chr11	34676443	34676655	15.00	14.60207	8.53119
594	chr11	34912461	34912662	16.00	15.89157	9.06439
595	chr11	34939034	34939211	21.00	22.68483	11.73039
596	chr11	35104000	35104257	25.00	17.28854	7.42115
597	chr11	43702057	43702342	15.00	14.60207	8.53119
598	chr11	44921527	44921884	85.00	129.79047	42.40414
599	chr11	45168619	45168919	105.00	167.94145	51.51937
600	chr11	45743746	45743933	19.00	11.44765	5.70858
601	chr11	45868460	45868726	10.00	5.81477	4.12139
602	chr11	46264684	46265000	45.00	60.64092	24.45206
603	chr11	46575440	46575712	16.00	11.45212	6.36943
604	chr11	46922807	46923012	15.00	14.34297	8.37430
605	chr11	47198275	47198519	23.00	19.14867	8.99213
606	chr11	47879113	47879392	19.00	7.69525	3.86660
607	chr11	47935537	47935825	22.00	24.04191	12.22603
608	chr11	47945817	47946160	17.00	17.20576	9.59759
609	chr11	56700338	56700609	18.00	18.54332	10.13079
610	chr11	57126125	57126423	44.00	55.43476	21.87143
611	chr11	57434783	57435184	116.00	147.04666	33.39518
612	chr11	57479237	57479506	28.00	17.16410	6.68511
613	chr11	57479698	57480073	113.00	151.60097	37.30113
614	chr11	58346410	58346588	17.00	15.60178	8.62543
615	chr11	58668248	58668540	18.00	13.54559	7.11877
616	chr11	58874172	58874703	24.00	26.99996	13.32998
617	chr11	58926071	58926279	20.00	12.37394	5.99401
618	chr11	58938974	58939291	78.00	100.09328	29.59910
619	chr11	59383181	59384016	230.00	444.91742	113.89949
620	chr11	59436740	59437064	19.00	18.57014	9.86143
621	chr11	61129740	61129971	12.00	10.35998	6.60115
622	chr11	61427013	61427200	13.00	11.52175	7.10893
623	chr11	61792908	61793275	24.00	22.71256	10.76729

624	chr11	61813232	61813419	16.00	15.07286	8.56837
625	chr11	61891192	61891533	95.00	153.63123	49.86253
626	chr11	62264674	62264849	13.00	10.72323	6.61555
627	chr11	62521592	62521984	103.00	143.59521	38.96591
628	chr11	62599027	62599477	209.00	401.28217	106.63390
629	chr11	62657462	62657770	55.00	63.10625	20.98164
630	chr11	62688726	62688929	18.00	18.54332	10.13079
631	chr11	63366777	63367069	28.00	20.43819	8.27744
632	chr11	63389769	63390001	17.00	12.48723	6.74410
633	chr11	63685272	63685521	22.00	11.75739	5.30198
634	chr11	63741717	63742043	81.00	117.43875	37.70157
635	chr11	63953035	63953753	219.00	433.81979	116.94465
636	chr11	64008848	64009161	46.00	62.49952	25.06037
637	chr11	64052052	64052387	56.00	76.36037	28.10507
638	chr11	64102559	64102921	40.00	34.24459	11.70258
639	chr11	64490589	64490912	67.00	100.69668	36.25756
640	chr11	64546085	64546525	41.00	42.51218	15.73623
641	chr11	64684740	64685016	26.00	28.14226	13.31293
642	chr11	64902473	64902663	11.00	8.94684	5.91686
643	chr11	65101040	65101253	15.00	14.60207	8.53119
644	chr11	65150386	65150598	22.00	18.29233	8.79342
645	chr11	65236371	65236546	15.00	7.96262	4.56686
646	chr11	65261385	65261604	20.00	17.07015	8.66101
647	chr11	65314045	65314375	18.00	16.74095	9.04099
648	chr11	65382761	65383208	217.00	363.78351	78.91506
649	chr11	65479791	65480077	34.00	42.40530	18.66198
650	chr11	65627844	65628158	46.00	49.66670	17.60959
651	chr11	66056571	66056996	144.00	220.36937	54.32747
652	chr11	66247647	66247986	103.00	172.10246	55.28292
653	chr11	66313382	66313743	109.00	183.83693	58.01935
654	chr11	66383845	66384044	20.00	18.72424	9.65528
655	chr11	66405839	66406243	147.00	257.06964	72.97457
656	chr11	66510925	66511176	15.00	14.13432	8.24751
657	chr11	66703896	66704082	12.00	10.05135	6.40993
658	chr11	66725793	66726205	114.00	195.16017	61.13016
659	chr11	66737722	66737945	20.00	15.72746	7.86812
660	chr11	67069590	67069972	91.00	106.50380	26.25946
661	chr11	67146934	67147219	49.00	65.89687	25.57979
662	chr11	67195804	67196086	34.00	38.05911	16.09213
663	chr11	67250798	67251113	44.00	58.92307	23.92050
664	chr11	67275546	67275907	92.00	124.09187	34.84451
665	chr11	67797912	67798227	44.00	46.77608	16.86025
666	chr11	68227706	68228175	148.00	184.02126	34.34763
667	chr11	68611305	68611690	88.00	117.13580	33.34583
668	chr11	68778540	68778844	72.00	85.49232	24.84083
669	chr11	69294698	69294883	18.00	9.05851	4.65195
670	chr11	69454041	69454242	23.00	19.14867	8.99213
671	chr11	69490197	69490413	17.00	14.49926	7.95370

672	chr11	71010479	71010664	16.00	10.06962	5.56245
673	chr11	71159377	71159795	100.00	135.87404	36.56157
674	chr11	71659612	71659855	23.00	15.26920	6.85029
675	chr11	71823232	71823690	170.00	267.76819	61.90126
676	chr11	71834454	71834630	22.00	11.75739	5.30198
677	chr11	72433453	72433788	45.00	27.68572	7.65773
678	chr11	72523714	72523944	23.00	17.13496	7.85287
679	chr11	72960354	72960709	30.00	16.43444	5.99323
680	chr11	73020435	73020635	15.00	7.96262	4.56686
681	chr11	75525796	75526342	204.00	305.81403	58.51292
682	chr11	77705768	77706170	100.00	138.22391	37.84189
683	chr11	78798251	78798475	20.00	14.47265	7.14599
684	chr11	79794673	79794891	30.00	27.72049	11.61484
685	chr11	82414866	82415165	24.00	21.62983	10.12671
686	chr11	82867559	82868013	208.00	287.73645	48.17889
687	chr11	83437478	83437687	22.00	14.28555	6.56486
688	chr11	85339419	85339760	49.00	64.31889	24.65357
689	chr11	85956359	85956645	16.00	15.84551	9.03663
690	chr11	86195352	86195645	27.00	28.01568	12.87371
691	chr11	86430822	86431152	17.00	17.20576	9.59759
692	chr11	90896730	90896984	25.00	28.47325	13.86318
693	chr11	93394498	93394937	15.00	14.55884	8.50506
694	chr11	95523195	95523822	98.00	132.36182	35.83757
695	chr11	95770894	95771159	17.00	17.20576	9.59759
696	chr11	101881855	101882092	19.00	18.93242	10.08043
697	chr11	102123819	102124140	27.00	23.95451	10.49082
698	chr11	102139237	102139585	37.00	36.97105	14.23754
699	chr11	105892839	105893484	241.00	478.66385	123.80620
700	chr11	105947918	105948294	169.00	295.46317	78.16179
701	chr11	107798735	107799104	145.00	262.88034	77.84711
702	chr11	107992868	107993106	25.00	14.38764	5.99355
703	chr11	110064929	110065267	25.00	27.69739	13.40219
704	chr11	111475113	111475382	26.00	29.70071	14.24111
705	chr11	111565559	111565874	33.00	40.80316	18.12878
706	chr11	111990541	111990877	85.00	124.86620	39.54067
707	chr11	113272496	113272748	22.00	17.98963	8.61746
708	chr11	113752673	113752866	18.00	18.49157	10.09976
709	chr11	114165807	114166058	16.00	15.84551	9.03663
710	chr11	114224955	114225206	19.00	19.84845	10.63133
711	chr11	117014791	117015195	100.00	138.22391	37.84189
712	chr11	117015804	117016046	23.00	23.93211	11.83371
713	chr11	117016289	117016465	20.00	19.88133	10.35450
714	chr11	117198351	117198600	20.00	21.28387	11.19719
715	chr11	117778035	117778337	19.00	18.45299	9.79053
716	chr11	117856980	117857218	29.00	30.81632	13.79326
717	chr11	118123105	118123280	10.00	8.58401	5.86519
718	chr11	118482529	118482814	13.00	12.10322	7.46479
719	chr11	118799187	118799588	191.00	354.68332	94.66971

720	chr11	118992100	118992450	123.00	206.12819	61.14085
721	chr11	119066434	119066737	57.00	56.32385	16.55487
722	chr11	120151293	120151605	34.00	42.40530	18.66198
723	chr11	122310146	122310357	13.00	12.06567	7.44193
724	chr11	122513202	122513377	16.00	14.76748	8.38221
725	chr11	122932821	122933003	15.00	13.54753	7.88914
726	chr11	123381890	123382098	17.00	15.90723	8.81147
727	chr11	123389790	123389965	12.00	10.89743	6.93159
728	chr11	124981574	124982010	143.00	258.43909	76.78071
729	chr11	124994062	124994394	98.00	162.06445	52.78674
730	chr11	125034540	125034832	39.00	50.59282	21.32797
731	chr11	125462357	125462879	216.00	427.24933	115.70426
732	chr11	125931961	125932225	25.00	21.51895	9.74148
733	chr11	126243274	126243533	25.00	28.47325	13.86318
734	chr11	128377328	128377618	29.00	32.49442	14.79214
735	chr11	128392570	128392803	24.00	22.71256	10.76729
736	chr11	129716889	129717155	20.00	21.22644	11.16290
737	chr11	129872748	129872935	23.00	19.46545	9.17574
738	chr11	130260217	130260644	64.00	77.17039	24.35369
739	chr11	130655335	130655627	28.00	27.59230	12.25744
740	chr11	131292508	131292744	21.00	16.84896	8.24279
741	chr11	132948588	132948826	19.00	17.47089	9.19550
742	chr11	133804642	133804956	44.00	59.04880	23.99397
743	chr12	680088	680767	105.00	128.89023	30.25546
744	chr12	798804	799036	21.00	22.68483	11.73039
745	chr12	1280889	1281173	32.00	24.84073	9.41915
746	chr12	1459613	1459818	10.00	8.58401	5.86519
747	chr12	2921504	2921933	235.00	407.32153	88.42264
748	chr12	5613051	5613271	19.00	18.57014	9.86143
749	chr12	6480290	6480621	47.00	63.43336	25.12292
750	chr12	6579747	6580180	77.00	119.93452	41.58955
751	chr12	6663348	6663600	26.00	28.14226	13.31293
752	chr12	6717629	6717824	13.00	12.10322	7.46479
753	chr12	6832880	6833249	80.00	125.81718	43.18915
754	chr12	6960362	6961326	151.00	242.37666	61.57037
755	chr12	7052581	7052920	99.00	136.44197	37.46722
756	chr12	7282876	7283093	16.00	15.89157	9.06439
757	chr12	8179131	8179490	103.00	137.62228	35.74699
758	chr12	8643908	8644119	12.00	10.89743	6.93159
759	chr12	9292648	9292890	24.00	26.99996	13.32998
760	chr12	10321704	10321946	21.00	22.62456	11.69446
761	chr12	10367427	10367764	61.00	89.27890	32.95713
762	chr12	12487374	12487571	23.00	15.26920	6.85029
763	chr12	12602792	12602979	15.00	12.67947	7.35640
764	chr12	12870086	12870460	111.00	168.76094	47.93423
765	chr12	12878247	12878563	77.00	108.86735	35.15038
766	chr12	13043734	13043949	17.00	12.48723	6.74410
767	chr12	13324837	13325164	25.00	25.14172	11.87397

768	chr12	13347774	13347987	17.00	17.15686	9.56820
769	chr12	13349441	13349885	15.00	14.60207	8.53119
770	chr12	14927063	14927427	211.00	356.23590	79.43050
771	chr12	14956428	14956818	144.00	260.24768	77.07716
772	chr12	16034908	16035096	11.00	9.72353	6.39839
773	chr12	20152100	20152352	13.00	12.06567	7.44193
774	chr12	21679054	21679261	10.00	8.58401	5.86519
775	chr12	25348079	25348255	14.00	9.45699	5.62008
776	chr12	25486862	25487043	11.00	9.72353	6.39839
777	chr12	25981084	25981262	13.00	11.69699	7.21657
778	chr12	27676790	27677198	178.00	256.78885	51.09177
779	chr12	27707964	27708161	19.00	14.62602	7.49344
780	chr12	28343170	28343508	79.00	123.85088	42.65595
781	chr12	31812171	31812350	20.00	15.72746	7.86812
782	chr12	31881354	31881581	16.00	8.79855	4.85229
783	chr12	41085978	41086304	51.00	71.28306	27.72637
784	chr12	46654332	46654641	22.00	17.98963	8.61746
785	chr12	46767637	46767816	21.00	15.66308	7.56186
786	chr12	46781085	46781327	31.00	27.23532	10.99907
787	chr12	46849553	46849814	30.00	27.72049	11.61484
788	chr12	47057228	47057431	15.00	12.67947	7.35640
789	chr12	47415146	47415373	20.00	15.72746	7.86812
790	chr12	48218924	48219099	16.00	12.31417	6.88616
791	chr12	48580067	48580242	19.00	14.88650	7.64645
792	chr12	49075972	49076363	107.00	168.23941	50.33553
793	chr12	49183307	49183494	13.00	12.10322	7.46479
794	chr12	49259429	49259805	92.00	108.07386	26.54489
795	chr12	49389208	49389462	28.00	25.19533	10.86549
796	chr12	49504516	49504984	140.00	234.67487	65.26905
797	chr12	49974257	49974535	24.00	23.93016	11.49438
798	chr12	50101285	50101639	103.00	157.05676	46.55901
799	chr12	50135028	50135340	49.00	48.26919	15.60998
800	chr12	50236456	50236808	82.00	82.57882	19.13324
801	chr12	50288262	50288449	19.00	14.62602	7.49344
802	chr12	50505643	50505967	46.00	49.66670	17.60959
803	chr12	51167533	51167804	50.00	69.36634	27.10990
804	chr12	51566879	51567089	17.00	14.16795	7.75245
805	chr12	51632779	51633209	215.00	398.55618	99.98663
806	chr12	52146382	52146658	43.00	45.34496	16.48558
807	chr12	52355677	52355852	13.00	10.64968	6.56995
808	chr12	52539008	52539281	23.00	21.65690	10.46904
809	chr12	52679179	52679380	24.00	16.27042	7.13572
810	chr12	53363555	53363954	38.00	44.49220	18.17672
811	chr12	53364541	53364716	14.00	12.09821	7.23880
812	chr12	53473080	53473448	187.00	358.66742	100.24148
813	chr12	53661918	53662166	22.00	14.85416	6.86577
814	chr12	53715071	53715301	23.00	15.26920	6.85029
815	chr12	53764580	53764806	23.00	20.39780	9.72164

816	chr12	53764989	53765263	22.00	14.28555	6.56486
817	chr12	53773119	53774026	238.00	342.55954	55.09452
818	chr12	53835251	53835597	87.00	98.24874	24.15130
819	chr12	54019649	54020086	207.00	368.74777	89.58382
820	chr12	54090049	54090374	61.00	70.99155	22.44373
821	chr12	54164106	54164410	25.00	24.24871	11.34146
822	chr12	54391696	54391986	35.00	34.26693	13.48820
823	chr12	54393348	54393523	17.00	12.48723	6.74410
824	chr12	54673283	54673777	209.00	315.41168	59.94006
825	chr12	54674073	54674569	111.00	125.10165	25.81835
826	chr12	54718574	54718927	125.00	196.67581	54.26712
827	chr12	54752874	54753310	176.00	253.08261	50.52091
828	chr12	54826005	54826267	19.00	17.36905	9.13383
829	chr12	56123323	56123706	100.00	108.53744	23.28262
830	chr12	56211483	56211892	130.00	170.79718	37.39118
831	chr12	56223296	56223698	167.00	236.52724	47.95205
832	chr12	56321725	56321919	14.00	11.39443	6.80473
833	chr12	56367239	56367909	270.00	376.93457	52.39246
834	chr12	56390687	56391107	103.00	113.00587	23.97418
835	chr12	56400663	56400935	15.00	14.55884	8.50506
836	chr12	56553018	56553289	19.00	17.16931	9.01292
837	chr12	56615077	56615375	38.00	42.14300	16.79697
838	chr12	56694170	56694393	30.00	27.72049	11.61484
839	chr12	56753864	56754197	36.00	45.64589	19.72838
840	chr12	57039657	57040181	233.00	403.02243	87.67329
841	chr12	57119070	57119333	38.00	31.82546	11.13173
842	chr12	57472455	57473008	141.00	222.35866	57.51969
843	chr12	57848876	57849138	28.00	31.56204	14.61662
844	chr12	58008048	58008333	27.00	29.57795	13.80600
845	chr12	58087493	58087954	64.00	90.57361	32.04964
846	chr12	58138556	58138940	111.00	188.82468	59.53546
847	chr12	58329848	58330186	38.00	38.34014	14.61222
848	chr12	64237425	64237712	35.00	44.01965	19.19518
849	chr12	65127711	65127970	14.00	12.62120	7.56032
850	chr12	65564217	65564461	21.00	21.21263	10.84757
851	chr12	65996734	65996978	29.00	28.03230	12.15205
852	chr12	67662785	67663185	168.00	303.03995	83.32906
853	chr12	68114114	68114289	11.00	6.67667	4.49607
854	chr12	68726048	68726344	46.00	48.59421	17.01380
855	chr12	69401871	69402189	47.00	58.23227	22.06921
856	chr12	71554565	71554803	24.00	26.99996	13.32998
857	chr12	72080067	72080254	11.00	9.72353	6.39839
858	chr12	74796320	74796618	21.00	16.84896	8.24279
859	chr12	74931076	74931378	33.00	40.80316	18.12878
860	chr12	75784717	75785032	42.00	48.13118	18.51973
861	chr12	76175507	76175749	25.00	21.51895	9.74148
862	chr12	76347421	76347596	19.00	11.44765	5.70858
863	chr12	76358019	76358263	26.00	18.32292	7.70658

864	chr12	76742121	76742480	127.00	200.64453	55.12850
865	chr12	79509584	79509760	12.00	10.89743	6.93159
866	chr12	80326867	80327128	19.00	19.90304	10.66399
867	chr12	83044500	83044675	16.00	11.45212	6.36943
868	chr12	85573765	85573946	10.00	8.58401	5.86519
869	chr12	86228361	86228539	13.00	12.10322	7.46479
870	chr12	87054609	87054784	17.00	15.02847	8.27595
871	chr12	90341449	90341664	18.00	16.23862	8.73573
872	chr12	90485259	90485451	13.00	12.10322	7.46479
873	chr12	91721689	91721934	19.00	14.62602	7.49344
874	chr12	92011694	92011894	13.00	12.10322	7.46479
875	chr12	92279805	92280021	15.00	14.13432	8.24751
876	chr12	92465027	92465282	19.00	19.90304	10.66399
877	chr12	92538536	92538780	23.00	25.47769	12.75760
878	chr12	92539691	92539999	24.00	26.99996	13.32998
879	chr12	93323069	93323451	64.00	95.04503	34.65796
880	chr12	94280408	94280713	29.00	21.51802	8.56287
881	chr12	95009308	95009483	12.00	9.35636	5.97708
882	chr12	95015598	95015815	25.00	17.28854	7.42115
883	chr12	95335493	95335702	14.00	7.15249	4.28143
884	chr12	95348007	95348220	35.00	31.16833	11.77930
885	chr12	95397346	95397633	34.00	42.40530	18.66198
886	chr12	95611276	95611560	43.00	54.87289	22.01398
887	chr12	95998869	95999098	18.00	13.54559	7.11877
888	chr12	96040811	96040987	12.00	10.44029	6.65075
889	chr12	96043158	96043482	41.00	35.46992	11.98801
890	chr12	98869084	98869301	22.00	19.18340	9.31657
891	chr12	100660498	100660913	28.00	20.43819	8.27744
892	chr12	100967587	100967955	55.00	78.45982	29.85916
893	chr12	103273037	103273224	12.00	10.89743	6.93159
894	chr12	104613847	104614173	44.00	46.77608	16.86025
895	chr12	104680592	104680864	24.00	26.09272	12.78990
896	chr12	104802388	104802563	12.00	7.57257	4.87074
897	chr12	105839497	105839706	15.00	13.93247	8.12449
898	chr12	105947981	105948299	37.00	47.28369	20.26158
899	chr12	105968155	105968402	21.00	21.47741	11.00699
900	chr12	106468548	106468990	56.00	77.06541	28.51812
901	chr12	106639857	106640032	12.00	10.05135	6.40993
902	chr12	107350504	107350790	15.00	10.44156	5.99476
903	chr12	108908688	108909080	86.00	137.72466	46.38834
904	chr12	109085377	109085567	19.00	18.57014	9.86143
905	chr12	109900176	109900446	27.00	25.42518	11.34191
906	chr12	110540210	110540415	18.00	18.49157	10.09976
907	chr12	112563033	112563445	190.00	312.49881	71.56239
908	chr12	113658728	113659325	112.00	140.39027	32.25346
909	chr12	115352197	115352588	26.00	22.72887	10.11615
910	chr12	116844438	116844707	12.00	10.89743	6.93159
911	chr12	117578143	117578371	13.00	11.60865	7.16234

912	chr12	117593131	117593489	58.00	67.72969	22.10566
913	chr12	117666165	117666394	15.00	14.60207	8.53119
914	chr12	118406620	118406934	67.00	97.29065	34.27346
915	chr12	119617623	119617860	15.00	10.44156	5.99476
916	chr12	120315019	120315365	110.00	167.36401	47.80675
917	chr12	120638838	120639273	208.00	407.55939	111.09741
918	chr12	120739896	120740184	29.00	34.52419	15.99598
919	chr12	121342298	121342687	91.00	147.78462	49.05434
920	chr12	121837624	121838039	198.00	378.35138	102.57834
921	chr12	121907396	121907637	20.00	21.28387	11.19719
922	chr12	122028074	122028396	57.00	82.09486	30.92556
923	chr12	122237392	122237605	19.00	19.57577	10.46788
924	chr12	122429898	122430107	14.00	13.33873	7.99799
925	chr12	123717184	123717590	198.00	383.90372	105.78175
926	chr12	123868413	123868798	138.00	240.37837	70.05899
927	chr12	125124925	125125115	23.00	19.14867	8.99213
928	chr12	125197480	125197655	12.00	10.89743	6.93159
929	chr12	129308468	129308775	91.00	147.78462	49.05434
930	chr12	130612829	130613074	20.00	18.72424	9.65528
931	chr12	132221330	132221536	21.00	13.32019	6.27944
932	chr12	132221810	132222078	20.00	18.51119	9.52662
933	chr12	133263865	133264111	32.00	39.21360	17.59558
934	chr12	133336800	133337027	12.00	7.57257	4.87074
935	chr13	20207567	20207906	118.00	170.99268	44.58599
936	chr13	20437709	20438101	77.00	119.93452	41.58955
937	chr13	20532577	20532919	88.00	141.73415	47.45474
938	chr13	21063401	21063730	35.00	43.91954	19.13640
939	chr13	21347941	21348155	16.00	15.89157	9.06439
940	chr13	26795828	26796168	92.00	149.81091	49.58754
941	chr13	33854082	33854404	12.00	7.57257	4.87074
942	chr13	34271297	34271605	51.00	57.04987	19.48295
943	chr13	35097054	35097310	17.00	17.20576	9.59759
944	chr13	36738154	36738365	14.00	13.33873	7.99799
945	chr13	39260949	39261177	14.00	13.33873	7.99799
946	chr13	40176394	40176603	15.00	14.60207	8.53119
947	chr13	41240713	41240996	24.00	26.99996	13.32998
948	chr13	44716035	44716363	95.00	155.64645	51.03039
949	chr13	44884532	44884827	26.00	29.96288	14.39638
950	chr13	45794210	45794409	10.00	8.58401	5.86519
951	chr13	46038716	46038891	11.00	9.72353	6.39839
952	chr13	47863883	47864117	20.00	21.28387	11.19719
953	chr13	52586124	52586464	74.00	114.10165	39.98995
954	chr13	60737981	60738381	202.00	368.10483	93.33437
955	chr13	73302393	73302694	22.00	17.98963	8.61746
956	chr13	73632893	73633240	115.00	186.13286	55.19762
957	chr13	73881855	73882137	27.00	28.01568	12.87371
958	chr13	74706364	74706640	33.00	40.70874	18.07326
959	chr13	74864635	74864962	66.00	54.78001	12.95312

960	chr13	76278178	76278364	13.00	11.18770	6.90300
961	chr13	76299347	76299555	14.00	13.33873	7.99799
962	chr13	76334402	76334689	41.00	46.61858	18.08904
963	chr13	80033960	80034386	10.00	8.58401	5.86519
964	chr13	80065843	80066222	11.00	9.72353	6.39839
965	chr13	89356867	89357066	16.00	11.45212	6.36943
966	chr13	91999778	91999972	18.00	18.54332	10.13079
967	chr13	95253911	95254113	15.00	14.60207	8.53119
968	chr13	97874275	97874632	104.00	139.01955	35.90943
969	chr13	101327107	101327381	55.00	78.45982	29.85916
970	chr13	103109812	103110009	12.00	10.89743	6.93159
971	chr13	106466937	106467246	19.00	11.44765	5.70858
972	chr13	106469333	106469590	12.00	10.89743	6.93159
973	chr13	106622141	106622413	22.00	22.56316	11.34064
974	chr13	107187975	107188272	49.00	54.07066	18.73361
975	chr13	108170790	108171041	18.00	13.54559	7.11877
976	chr13	109615050	109615370	40.00	52.14924	21.79423
977	chr13	110438883	110439307	33.00	40.80316	18.12878
978	chr13	110651010	110651210	17.00	12.48723	6.74410
979	chr13	110747267	110747549	44.00	39.20615	12.84430
980	chr13	111217614	111217835	17.00	16.01209	8.87529
981	chr13	111267601	111267974	131.00	195.40366	49.45673
982	chr13	111365343	111365797	162.00	258.04126	62.31859
983	chr13	111367649	111367916	32.00	38.22182	17.01048
984	chr13	111522600	111522944	104.00	142.94951	38.00955
985	chr13	111617808	111618057	36.00	29.45019	10.56087
986	chr13	112236458	112236795	70.00	74.70468	20.26545
987	chr13	113312523	113312740	16.00	13.84165	7.81618
988	chr13	113709942	113710167	17.00	17.20576	9.59759
989	chr13	113807160	113807415	26.00	29.15619	13.91766
990	chr13	114147710	114147885	9.00	7.48180	5.33199
991	chr14	20811379	20811794	29.00	32.49442	14.79214
992	chr14	21077613	21077917	31.00	37.63698	17.06238
993	chr14	21482951	21483192	21.00	10.91615	5.07146
994	chr14	21572110	21572446	38.00	46.99860	19.65684
995	chr14	21735865	21736067	15.00	12.67947	7.35640
996	chr14	21737519	21738131	257.00	526.30292	137.14418
997	chr14	21776831	21777200	48.00	65.98680	26.12677
998	chr14	21905274	21905688	197.00	368.35794	97.62814
999	chr14	22801307	22801485	14.00	13.33873	7.99799
1000	chr14	22802447	22802780	24.00	26.99996	13.32998
1001	chr14	23058461	23058654	18.00	17.28003	9.36836
1002	chr14	23083179	23083560	99.00	146.42021	43.06914
1003	chr14	23340692	23341104	88.00	141.73415	47.45474
1004	chr14	23398601	23398776	13.00	8.67630	5.35252
1005	chr14	23476267	23476645	138.00	208.78119	52.07943
1006	chr14	23790158	23790743	242.00	379.99652	69.35921
1007	chr14	23938445	23938961	125.00	190.97365	51.03860

1008	chr14	24583595	24584492	74.00	80.57626	21.40717
1009	chr14	24610891	24611205	60.00	74.14967	24.70916
1010	chr14	24740185	24740556	79.00	123.05749	42.19589
1011	chr14	25476302	25476614	38.00	46.27534	19.22978
1012	chr14	28851859	28852078	10.00	8.58401	5.86519
1013	chr14	28852214	28852401	12.00	10.89743	6.93159
1014	chr14	31698987	31699380	68.00	71.80498	19.69459
1015	chr14	32979129	32979392	15.00	14.60207	8.53119
1016	chr14	34407498	34407814	41.00	50.08941	20.12600
1017	chr14	34420267	34420495	24.00	20.32534	9.36681
1018	chr14	35344536	35344743	21.00	22.68483	11.73039
1019	chr14	36789772	36790118	84.00	124.39368	39.88898
1020	chr14	36989273	36989597	48.00	65.98680	26.12677
1021	chr14	37130748	37131197	210.00	354.13199	79.05583
1022	chr14	37304424	37304599	10.00	8.58401	5.86519
1023	chr14	39309082	39309372	33.00	40.80316	18.12878
1024	chr14	39644230	39644598	153.00	270.07983	75.93300
1025	chr14	45603631	45603975	79.00	122.49884	41.87152
1026	chr14	50087156	50087572	210.00	354.13199	79.05583
1027	chr14	50417085	50417343	8.00	6.42036	4.79879
1028	chr14	50779424	50779613	16.00	15.89157	9.06439
1029	chr14	51009706	51009981	31.00	29.00390	11.98951
1030	chr14	51561795	51562143	100.00	138.22391	37.84189
1031	chr14	52456055	52456417	92.00	138.05537	42.75909
1032	chr14	53019205	53019638	111.00	138.73566	31.96803
1033	chr14	53418429	53418683	28.00	17.16410	6.68511
1034	chr14	53925139	53925383	8.00	6.00547	4.53619
1035	chr14	54391556	54391742	16.00	14.57150	8.26254
1036	chr14	54465750	54465987	13.00	12.10322	7.46479
1037	chr14	54863396	54863625	23.00	22.60143	11.03461
1038	chr14	54976345	54976704	31.00	35.46775	15.77829
1039	chr14	55589363	55589597	16.00	11.45212	6.36943
1040	chr14	55594274	55594466	16.00	8.79855	4.85229
1041	chr14	55658228	55658591	70.00	86.84992	26.60172
1042	chr14	55915118	55915312	20.00	15.72746	7.86812
1043	chr14	56219689	56219893	9.00	7.13674	5.11596
1044	chr14	58764831	58765099	19.00	19.90304	10.66399
1045	chr14	59365649	59365848	14.00	13.33873	7.99799
1046	chr14	60558359	60558541	14.00	10.20871	6.07602
1047	chr14	60714612	60714964	104.00	145.39403	39.34058
1048	chr14	61116104	61116414	40.00	52.26358	21.86117
1049	chr14	61190992	61191327	42.00	48.13118	18.51973
1050	chr14	62036525	62036749	17.00	15.60178	8.62543
1051	chr14	62228804	62229116	35.00	44.01965	19.19518
1052	chr14	64010000	64010351	61.00	88.40855	32.45043
1053	chr14	64108816	64109368	129.00	191.61064	48.70739
1054	chr14	64357700	64358057	25.00	22.87882	10.53177
1055	chr14	64385198	64385373	16.00	14.76748	8.38221

1056	chr14	64805414	64805734	103.00	172.39622	55.45273
1057	chr14	64854683	64855029	67.00	81.98164	25.47771
1058	chr14	64888063	64888254	13.00	12.10322	7.46479
1059	chr14	64971849	64972179	114.00	187.52452	56.70321
1060	chr14	65006921	65007505	186.00	332.56866	85.97797
1061	chr14	65878351	65878696	103.00	149.28288	42.12710
1062	chr14	67441379	67441666	28.00	25.19533	10.86549
1063	chr14	67708810	67709056	17.00	17.15686	9.56820
1064	chr14	67826532	67826709	15.00	14.34297	8.37430
1065	chr14	68161514	68161724	19.00	16.50842	8.61383
1066	chr14	68438847	68439119	10.00	8.55496	5.84723
1067	chr14	68658456	68658665	10.00	8.58401	5.86519
1068	chr14	69259734	69260063	78.00	101.18623	30.20349
1069	chr14	69329774	69329985	15.00	14.60207	8.53119
1070	chr14	69445984	69446409	104.00	171.26086	54.12425
1071	chr14	69926849	69927079	20.00	20.79970	10.90743
1072	chr14	70345864	70346237	201.00	381.34192	101.81235
1073	chr14	70348950	70349136	15.00	12.67947	7.35640
1074	chr14	71276378	71276745	62.00	73.99586	23.60435
1075	chr14	73392899	73393265	131.00	232.04996	70.38232
1076	chr14	73925192	73925726	233.00	468.24945	124.76865
1077	chr14	73957487	73957662	13.00	11.60865	7.16234
1078	chr14	74190583	74190896	34.00	32.93251	13.11353
1079	chr14	74351570	74351858	29.00	34.44115	15.94700
1080	chr14	75355084	75355268	11.00	9.72353	6.39839
1081	chr14	75469223	75469698	55.00	73.94379	27.21778
1082	chr14	75530581	75531118	172.00	323.93753	92.24349
1083	chr14	75721700	75721893	18.00	13.54559	7.11877
1084	chr14	75919142	75919396	22.00	21.90452	10.94436
1085	chr14	76127152	76127504	100.00	120.79749	28.82832
1086	chr14	77423109	77423436	37.00	46.91131	20.04305
1087	chr14	77499487	77499749	17.00	17.20576	9.59759
1088	chr14	77512934	77513109	10.00	8.58401	5.86519
1089	chr14	78227389	78227622	24.00	20.32534	9.36681
1090	chr14	79483182	79483475	14.00	13.33873	7.99799
1091	chr14	81866570	81866834	16.00	15.84551	9.03663
1092	chr14	82000191	82000386	10.00	8.58401	5.86519
1093	chr14	89882359	89882783	170.00	242.02304	48.80834
1094	chr14	90420957	90421351	136.00	225.61270	62.98920
1095	chr14	90920681	90920982	32.00	39.21360	17.59558
1096	chr14	91526966	91527600	90.00	145.76303	48.52114
1097	chr14	91882312	91882530	17.00	12.48723	6.74410
1098	chr14	92505681	92505971	40.00	52.26358	21.86117
1099	chr14	93616915	93617126	11.00	6.67667	4.49607
1100	chr14	94004817	94005104	39.00	45.60815	18.39101
1101	chr14	94492484	94492845	128.00	225.52483	68.78271
1102	chr14	95927366	95927614	13.00	12.10322	7.46479
1103	chr14	96000936	96001327	155.00	241.89040	58.44886

1104	chr14	99034968	99035145	12.00	10.86272	6.91037
1105	chr14	99947533	99947780	21.00	19.99769	10.11506
1106	chr14	100224820	100225072	15.00	13.54753	7.88914
1107	chr14	100489205	100489470	21.00	22.68483	11.73039
1108	chr14	100770262	100770477	27.00	23.95451	10.49082
1109	chr14	100862637	100862865	17.00	7.75030	4.14938
1110	chr14	100891162	100891387	28.00	25.58257	11.08736
1111	chr14	100906455	100906731	17.00	12.48723	6.74410
1112	chr14	100912904	100913248	67.00	99.08344	35.31929
1113	chr14	102430526	102430738	27.00	19.37298	7.99201
1114	chr14	102783686	102783913	17.00	15.60178	8.62543
1115	chr14	102991085	102991443	19.00	14.19124	7.23991
1116	chr14	103058668	103058915	24.00	26.99996	13.32998
1117	chr14	103060929	103061136	22.00	22.56316	11.34064
1118	chr14	103588391	103588665	21.00	16.84896	8.24279
1119	chr14	103653285	103653519	22.00	24.10503	12.26359
1120	chr14	103665116	103665313	18.00	16.23862	8.73573
1121	chr14	103752599	103752885	19.00	19.57577	10.46788
1122	chr14	103796315	103796583	25.00	24.00721	11.19798
1123	chr14	103800285	103800618	85.00	128.75090	41.79874
1124	chr14	104181718	104182214	105.00	171.22319	53.42628
1125	chr14	104346419	104346623	13.00	10.43506	6.43685
1126	chr14	104478396	104478588	19.00	11.44765	5.70858
1127	chr14	105148114	105148448	62.00	73.99586	23.60435
1128	chr14	105171822	105171998	13.00	12.10322	7.46479
1129	chr14	105557919	105558139	16.00	15.89157	9.06439
1130	chr14	105634996	105635188	18.00	18.54332	10.13079
1131	chr14	107287554	107287789	22.00	24.10503	12.26359
1132	chr15	22977296	22977626	31.00	37.63698	17.06238
1133	chr15	33486861	33487204	92.00	148.88719	49.05273
1134	chr15	34563762	34563960	16.00	11.45212	6.36943
1135	chr15	35598100	35598435	41.00	51.07812	20.70900
1136	chr15	36639678	36639995	28.00	32.90870	15.41543
1137	chr15	36665843	36666034	12.00	10.89743	6.93159
1138	chr15	37070833	37071071	21.00	22.62456	11.69446
1139	chr15	37089459	37089666	12.00	10.05135	6.40993
1140	chr15	37391966	37392332	89.00	143.74619	47.98794
1141	chr15	37393784	37394112	58.00	79.87083	29.09121
1142	chr15	40074663	40074946	17.00	17.15686	9.56820
1143	chr15	40697733	40698028	38.00	44.07580	17.93123
1144	chr15	40722690	40722928	15.00	14.60207	8.53119
1145	chr15	41150328	41150503	13.00	8.50004	5.24541
1146	chr15	41221310	41221656	107.00	180.73669	57.58553
1147	chr15	41245449	41245624	14.00	9.45699	5.62008
1148	chr15	41575881	41576278	197.00	381.54993	105.25018
1149	chr15	42220284	42220474	12.00	10.05135	6.40993
1150	chr15	42222447	42222768	24.00	20.32534	9.36681
1151	chr15	42264755	42265086	33.00	22.07791	7.83771

1152	chr15	42783759	42783961	13.00	8.50004	5.24541
1153	chr15	43212938	43213332	157.00	289.77155	84.24550
1154	chr15	43785218	43785641	210.00	354.13199	79.05583
1155	chr15	44487391	44487841	237.00	340.70459	54.86400
1156	chr15	45410433	45410629	11.00	9.72353	6.39839
1157	chr15	45571273	45571696	160.00	290.53610	82.36694
1158	chr15	45694276	45694704	231.00	456.26175	119.58882
1159	chr15	47488368	47488543	14.00	10.83560	6.46037
1160	chr15	48112942	48113265	45.00	48.21673	17.23492
1161	chr15	48298117	48298396	16.00	11.45212	6.36943
1162	chr15	49103131	49103670	125.00	219.02989	67.18312
1163	chr15	49256019	49256279	14.00	12.80671	7.67394
1164	chr15	49438321	49438550	16.00	15.84551	9.03663
1165	chr15	49913020	49913218	18.00	17.28003	9.36836
1166	chr15	51178408	51178619	14.00	13.29834	7.97350
1167	chr15	51673406	51673646	12.00	10.89743	6.93159
1168	chr15	51717011	51717384	59.00	51.93626	13.83126
1169	chr15	52018627	52018910	14.00	13.33873	7.99799
1170	chr15	52121299	52121588	31.00	29.00390	11.98951
1171	chr15	52138699	52138994	34.00	38.05911	16.09213
1172	chr15	57179345	57179548	18.00	16.23862	8.73573
1173	chr15	58624234	58624564	36.00	29.45019	10.56087
1174	chr15	58820404	58820701	14.00	9.45699	5.62008
1175	chr15	59397047	59397336	19.00	19.90304	10.66399
1176	chr15	59537889	59538072	16.00	11.45212	6.36943
1177	chr15	59949641	59949967	92.00	133.36130	40.05430
1178	chr15	60120489	60120787	23.00	19.14867	8.99213
1179	chr15	60178056	60178373	38.00	44.07580	17.93123
1180	chr15	60385909	60386199	37.00	47.17789	20.19953
1181	chr15	60663346	60663651	18.00	13.54559	7.11877
1182	chr15	62896086	62896313	19.00	9.29158	4.61042
1183	chr15	63343523	63343757	14.00	12.71320	7.61671
1184	chr15	63795315	63795655	111.00	151.65845	38.49676
1185	chr15	64673614	64673994	91.00	144.97163	47.42315
1186	chr15	65186314	65186496	16.00	15.28459	8.69713
1187	chr15	65341709	65341912	16.00	15.07286	8.56837
1188	chr15	66125280	66125469	17.00	14.16795	7.75245
1189	chr15	67058344	67058529	17.00	12.09955	6.51592
1190	chr15	67135325	67135596	17.00	9.65886	5.13772
1191	chr15	67303561	67303829	21.00	16.84896	8.24279
1192	chr15	67341820	67342231	45.00	48.21673	17.23492
1193	chr15	67348632	67348859	15.00	14.60207	8.53119
1194	chr15	67417715	67417962	24.00	16.27042	7.13572
1195	chr15	67440873	67441071	13.00	11.97110	7.38428
1196	chr15	68291279	68291500	15.00	13.36375	7.77651
1197	chr15	69113075	69113464	145.00	262.88034	77.84711
1198	chr15	69436175	69436356	14.00	12.26727	7.34289
1199	chr15	69452602	69453009	116.00	199.40269	62.19329

1200	chr15	70100109	70100341	14.00	7.15249	4.28143
1201	chr15	70391915	70392327	237.00	411.62796	89.17198
1202	chr15	71571558	71571942	21.00	22.62456	11.69446
1203	chr15	72523768	72524099	36.00	43.58135	18.51176
1204	chr15	74833204	74833459	21.00	22.68483	11.73039
1205	chr15	75134869	75135220	113.00	193.36678	60.78473
1206	chr15	75165249	75165630	150.00	272.52982	79.64475
1207	chr15	75491111	75491315	13.00	11.02827	6.80445
1208	chr15	75743959	75744750	104.00	171.26086	54.12425
1209	chr15	75748097	75748325	18.00	13.54559	7.11877
1210	chr15	76304583	76304913	24.00	26.93115	13.28916
1211	chr15	78505047	78505329	27.00	19.37298	7.99201
1212	chr15	78650505	78650807	14.00	11.54338	6.89663
1213	chr15	78799473	78799782	107.00	161.60327	46.51468
1214	chr15	79141101	79141384	27.00	31.46828	14.92958
1215	chr15	79462644	79462837	11.00	8.94684	5.91686
1216	chr15	80364710	80364969	31.00	29.00390	11.98951
1217	chr15	81616437	81616803	76.00	108.27338	35.40269
1218	chr15	83478048	83478419	106.00	149.00392	40.08992
1219	chr15	85174393	85174825	264.00	470.45416	99.28813
1220	chr15	85523780	85523988	23.00	15.26920	6.85029
1221	chr15	85535794	85536047	15.00	6.30094	3.68834
1222	chr15	85923531	85923815	23.00	25.54366	12.79678
1223	chr15	85987126	85987320	21.00	15.66308	7.56186
1224	chr15	86338052	86338327	102.00	156.19621	46.72579
1225	chr15	89533742	89533936	16.00	8.79855	4.85229
1226	chr15	89631298	89631780	253.00	401.97052	72.49893
1227	chr15	89721104	89721279	18.00	13.54559	7.11877
1228	chr15	90548427	90548662	24.00	13.49383	5.76302
1229	chr15	90895160	90895611	205.00	401.02475	109.83907
1230	chr15	91103779	91103978	23.00	14.75946	6.58672
1231	chr15	91135452	91135737	21.00	13.32019	6.27944
1232	chr15	91537639	91538039	172.00	275.80307	64.81829
1233	chr15	91653240	91653415	13.00	11.10738	6.85337
1234	chr15	91822802	91822987	16.00	13.92852	7.86932
1235	chr15	93425274	93425859	238.00	413.78391	89.54665
1236	chr15	93443343	93443734	103.00	141.17438	37.64755
1237	chr15	93448324	93448502	22.00	11.75739	5.30198
1238	chr15	98503526	98503701	12.00	10.05135	6.40993
1239	chr15	99195008	99195211	15.00	12.67947	7.35640
1240	chr15	99395526	99395939	246.00	432.64172	93.38493
1241	chr15	99440260	99440477	26.00	15.29767	6.22407
1242	chr15	99789647	99789992	134.00	210.29303	55.67795
1243	chr15	101262106	101262335	24.00	20.32534	9.36681
1244	chr15	102070717	102071081	86.00	122.34853	37.47015
1245	chr15	102181483	102181658	14.00	9.54176	5.67115
1246	chr16	103490	103813	41.00	53.94481	22.39437
1247	chr16	104553	104787	11.00	9.53247	6.28073

1248	chr16	420441	420767	114.00	195.48526	61.31793
1249	chr16	639360	640084	173.00	326.23587	92.77669
1250	chr16	810660	810886	13.00	12.10322	7.46479
1251	chr16	857886	858136	22.00	17.98963	8.61746
1252	chr16	1728044	1728367	62.00	91.31023	33.59156
1253	chr16	1756036	1756334	53.00	74.85551	28.79276
1254	chr16	1832518	1833108	178.00	287.94882	67.06632
1255	chr16	2205314	2205766	141.00	247.76808	72.10482
1256	chr16	2262539	2262872	67.00	85.67113	27.54464
1257	chr16	2273325	2273664	85.00	135.72733	45.85514
1258	chr16	2571417	2571605	13.00	12.10322	7.46479
1259	chr16	2932688	2932960	25.00	21.51895	9.74148
1260	chr16	2955383	2955576	10.00	8.58401	5.86519
1261	chr16	3029880	3030141	18.00	18.49157	10.09976
1262	chr16	3053880	3054201	40.00	52.26358	21.86117
1263	chr16	3284982	3285332	75.00	95.08407	28.47509
1264	chr16	3314420	3314837	157.00	289.77155	84.24550
1265	chr16	3450924	3451298	121.00	200.48892	59.29589
1266	chr16	3989455	3989724	40.00	52.26358	21.86117
1267	chr16	4094788	4095039	11.00	6.67667	4.49607
1268	chr16	4357860	4358074	24.00	21.62983	10.12671
1269	chr16	4666179	4666425	47.00	51.12580	17.98426
1270	chr16	4784297	4784565	48.00	64.83015	25.45066
1271	chr16	4852827	4853196	117.00	201.86313	62.91753
1272	chr16	4962102	4962327	13.00	11.18770	6.90300
1273	chr16	8964641	8964980	47.00	64.10442	25.51520
1274	chr16	9085933	9086109	13.00	12.10322	7.46479
1275	chr16	9185174	9185456	92.00	141.70955	44.88058
1276	chr16	10837870	10838279	121.00	188.77977	52.54435
1277	chr16	11439090	11439381	62.00	82.07784	28.20401
1278	chr16	11734409	11734759	54.00	61.58029	20.60697
1279	chr16	11810307	11810553	14.00	12.01554	7.18786
1280	chr16	11961399	11961593	17.00	15.90723	8.81147
1281	chr16	12043436	12043611	12.00	10.89743	6.93159
1282	chr16	14579213	14579452	17.00	15.80392	8.74857
1283	chr16	14580521	14580728	23.00	18.61959	8.68790
1284	chr16	15521644	15521867	21.00	22.68483	11.73039
1285	chr16	15684891	15685175	14.00	7.15249	4.28143
1286	chr16	15982489	15982668	16.00	14.76748	8.38221
1287	chr16	16115812	16116060	25.00	21.51895	9.74148
1288	chr16	16267979	16268154	11.00	9.72353	6.39839
1289	chr16	16984986	16985219	18.00	18.54332	10.13079
1290	chr16	19010108	19010327	10.00	8.58401	5.86519
1291	chr16	19078604	19079423	176.00	283.89041	66.31698
1292	chr16	19191248	19191504	28.00	31.02884	14.29907
1293	chr16	19386898	19387191	27.00	29.57795	13.80600
1294	chr16	22385836	22386232	200.00	389.18781	107.17307
1295	chr16	22681936	22682240	62.00	64.92486	18.80624

1296	chr16	23521939	23522162	15.00	14.60207	8.53119
1297	chr16	23568824	23569143	78.00	121.88997	42.12275
1298	chr16	23689868	23690197	40.00	29.47528	9.45136
1299	chr16	25122819	25123134	82.00	106.84836	31.09779
1300	chr16	27095275	27095580	37.00	47.17789	20.19953
1301	chr16	27279983	27280333	134.00	208.52621	54.68421
1302	chr16	28222287	28222510	13.00	12.10322	7.46479
1303	chr16	28857713	28857908	18.00	18.49157	10.09976
1304	chr16	28961953	28962396	196.00	366.07330	97.13506
1305	chr16	29776849	29777185	31.00	29.00390	11.98951
1306	chr16	29816023	29816238	23.00	23.93211	11.83371
1307	chr16	29822385	29822717	38.00	48.93276	20.79478
1308	chr16	30032443	30032882	237.00	411.62796	89.17198
1309	chr16	30047920	30048215	33.00	31.61030	12.73885
1310	chr16	30381977	30382656	213.00	419.46561	113.75525
1311	chr16	30457067	30457544	209.00	395.94119	103.54500
1312	chr16	30579554	30579800	22.00	14.28555	6.56486
1313	chr16	30773392	30773583	15.00	12.84344	7.45712
1314	chr16	30906211	30906413	22.00	11.75739	5.30198
1315	chr16	30934012	30934297	26.00	24.14413	10.93684
1316	chr16	30959470	30959819	105.00	173.31308	54.63972
1317	chr16	31009577	31009912	63.00	76.46091	24.46865
1318	chr16	31106038	31106329	21.00	22.68483	11.73039
1319	chr16	31227243	31227488	17.00	16.67645	9.27844
1320	chr16	31415927	31416259	65.00	96.92242	35.19116
1321	chr16	31711624	31711959	32.00	39.21360	17.59558
1322	chr16	46655237	46655412	9.00	7.48180	5.33199
1323	chr16	46723056	46723552	116.00	199.73347	62.38433
1324	chr16	46782116	46782518	122.00	204.04678	60.64778
1325	chr16	46865287	46865619	54.00	52.25098	15.69859
1326	chr16	53133820	53134049	12.00	8.75023	5.59899
1327	chr16	53469240	53469630	104.00	145.39403	39.34058
1328	chr16	53537129	53537680	253.00	446.33670	95.16673
1329	chr16	53803867	53804053	16.00	11.45212	6.36943
1330	chr16	54320590	54320829	12.00	10.89743	6.93159
1331	chr16	54964601	54964784	12.00	10.89743	6.93159
1332	chr16	56485191	56485464	14.00	12.35372	7.39607
1333	chr16	56725929	56726174	46.00	32.02672	9.08652
1334	chr16	56965851	56966113	25.00	28.47325	13.86318
1335	chr16	57125287	57125553	28.00	31.02884	14.29907
1336	chr16	57219986	57220229	12.00	10.89743	6.93159
1337	chr16	57569884	57570122	20.00	21.28387	11.19719
1338	chr16	57850352	57850527	14.00	10.83560	6.46037
1339	chr16	57852432	57852827	23.00	23.93211	11.83371
1340	chr16	57927760	57928063	42.00	51.93481	20.75124
1341	chr16	57944586	57944988	22.00	22.70079	11.42337
1342	chr16	58163416	58163728	42.00	36.70545	12.27344
1343	chr16	66542228	66542510	29.00	26.85215	11.46968

1344	chr16	66914161	66914547	206.00	345.73703	77.55714
1345	chr16	67217402	67217765	110.00	186.72050	59.00389
1346	chr16	67926830	67927214	115.00	197.60750	61.85113
1347	chr16	67969540	67969865	71.00	103.36067	35.50114
1348	chr16	68271347	68271631	37.00	42.55443	17.47146
1349	chr16	68292333	68292533	17.00	15.02847	8.27595
1350	chr16	68301625	68301858	16.00	11.45212	6.36943
1351	chr16	68367976	68368277	9.00	7.48180	5.33199
1352	chr16	69213622	69213841	14.00	9.45699	5.62008
1353	chr16	69432003	69432291	33.00	22.07791	7.83771
1354	chr16	69458172	69458609	35.00	28.27971	10.27544
1355	chr16	69511258	69511475	11.00	8.94684	5.91686
1356	chr16	69950454	69950629	14.00	12.90175	7.73204
1357	chr16	70323353	70323680	175.00	330.34113	93.55572
1358	chr16	70414713	70414905	14.00	12.99838	7.79102
1359	chr16	70415242	70415601	39.00	50.59282	21.32797
1360	chr16	72126687	72127029	63.00	75.57976	23.97902
1361	chr16	72133228	72133456	21.00	16.84896	8.24279
1362	chr16	72698695	72699114	187.00	358.13440	99.93452
1363	chr16	72961673	72961928	22.00	17.98963	8.61746
1364	chr16	73129364	73129598	23.00	21.43488	10.33659
1365	chr16	73650192	73650396	20.00	10.09393	4.84094
1366	chr16	74700740	74701151	97.00	132.89111	36.71788
1367	chr16	75410581	75410757	11.00	8.94684	5.91686
1368	chr16	75498497	75498762	35.00	44.01965	19.19518
1369	chr16	75551113	75551438	64.00	86.86694	29.88539
1370	chr16	77224305	77224814	96.00	131.12227	36.34320
1371	chr16	79634610	79634836	18.00	18.54332	10.13079
1372	chr16	80966617	80966951	67.00	100.50546	36.14653
1373	chr16	80971933	80972249	51.00	48.24844	14.84230
1374	chr16	81030278	81030512	19.00	19.84845	10.63133
1375	chr16	81481776	81481999	14.00	12.62120	7.56032
1376	chr16	84189930	84190220	14.00	13.33873	7.99799
1377	chr16	84224508	84224829	36.00	45.64589	19.72838
1378	chr16	84314724	84315046	49.00	67.60371	26.57833
1379	chr16	84538115	84538506	191.00	314.55969	71.93706
1380	chr16	84619506	84619809	30.00	27.72049	11.61484
1381	chr16	84734245	84734480	26.00	18.32292	7.70658
1382	chr16	85061184	85061511	65.00	92.38122	32.54271
1383	chr16	85207934	85208251	35.00	44.01965	19.19518
1384	chr16	85593338	85593526	12.00	10.52193	6.70110
1385	chr16	85767343	85767631	33.00	19.13387	6.57322
1386	chr16	86376981	86377163	13.00	11.78681	7.27162
1387	chr16	86609465	86609751	32.00	36.97464	16.27136
1388	chr16	86774985	86775286	32.00	30.30064	12.36418
1389	chr16	87421858	87422432	84.00	85.38596	19.59428
1390	chr16	87526640	87526943	44.00	46.77608	16.86025
1391	chr16	87615786	87616041	23.00	15.26920	6.85029

1392	chr16	87887796	87887993	17.00	12.48723	6.74410
1393	chr16	87888221	87888479	13.00	9.77812	6.02968
1394	chr16	88837335	88837523	8.00	6.11255	4.60436
1395	chr16	88839918	88840174	16.00	13.03207	7.32175
1396	chr16	89237588	89238328	82.00	129.76570	44.25555
1397	chr16	89556974	89557499	142.00	256.22293	76.24751
1398	chr16	89558769	89559062	42.00	52.69979	21.20207
1399	chr16	89939408	89939583	14.00	13.29834	7.97350
1400	chr16	90143684	90144063	92.00	149.81091	49.58754
1401	chr17	618042	618255	19.00	19.05697	10.15561
1402	chr17	1572713	1572888	11.00	9.72353	6.39839
1403	chr17	1588058	1588452	60.00	83.41049	30.07736
1404	chr17	1618395	1618717	13.00	11.18770	6.90300
1405	chr17	1619524	1619937	79.00	101.77402	29.97378
1406	chr17	1929435	1929610	11.00	6.67667	4.49607
1407	chr17	2263006	2263181	18.00	11.98297	6.21686
1408	chr17	2310293	2310543	25.00	22.87882	10.53177
1409	chr17	2786920	2787221	47.00	33.08840	9.27985
1410	chr17	3539438	3539785	94.00	127.59798	35.59386
1411	chr17	3796666	3796860	16.00	11.45212	6.36943
1412	chr17	4047252	4047579	17.00	17.20576	9.59759
1413	chr17	4870434	4870984	178.00	327.57785	89.55689
1414	chr17	5389578	5389962	13.00	11.18770	6.90300
1415	chr17	5662097	5662310	22.00	22.56316	11.34064
1416	chr17	6410020	6410333	50.00	55.55605	19.10828
1417	chr17	6555515	6555844	39.00	33.02968	11.41716
1418	chr17	6910492	6910795	24.00	26.58739	13.08485
1419	chr17	7359736	7359942	16.00	11.45212	6.36943
1420	chr17	7475746	7476187	233.00	362.17792	66.79036
1421	chr17	7789312	7789559	27.00	29.57795	13.80600
1422	chr17	7835138	7835445	43.00	52.79736	20.79172
1423	chr17	8021778	8021983	20.00	10.09393	4.84094
1424	chr17	8059827	8060146	48.00	65.98680	26.12677
1425	chr17	8093480	8093731	27.00	16.22333	6.45459
1426	chr17	8125684	8126078	98.00	147.46980	44.32059
1427	chr17	8191577	8191927	31.00	35.66154	15.89338
1428	chr17	8533950	8534528	168.00	314.76944	90.11069
1429	chr17	9304962	9305212	24.00	20.32534	9.36681
1430	chr17	9666992	9667218	23.00	19.14867	8.99213
1431	chr17	12294915	12295092	15.00	13.01197	7.56063
1432	chr17	12426182	12426447	18.00	18.10711	9.86863
1433	chr17	12452825	12453216	25.00	21.86390	9.94039
1434	chr17	12569044	12569302	26.00	29.96288	14.39638
1435	chr17	12921263	12921624	72.00	107.53007	37.34650
1436	chr17	13972654	13972892	12.00	10.44029	6.65075
1437	chr17	14204141	14204512	148.00	228.15480	55.82616
1438	chr17	15192853	15193086	24.00	25.17084	12.23816
1439	chr17	15848217	15848473	23.00	23.37735	11.50057

1440	chr17	16341861	16342180	63.00	88.77263	31.55657
1441	chr17	16945490	16945743	55.00	69.16361	24.42776
1442	chr17	17183875	17184236	85.00	135.72733	45.85514
1443	chr17	17349664	17350009	32.00	39.21360	17.59558
1444	chr17	17350156	17350331	10.00	8.58401	5.86519
1445	chr17	17566634	17566958	83.00	113.12780	34.02573
1446	chr17	17875594	17876271	226.00	348.42245	64.79236
1447	chr17	17942375	17942680	32.00	36.39178	15.92537
1448	chr17	18086476	18086900	227.00	390.16986	85.42526
1449	chr17	18128668	18128884	21.00	22.68483	11.73039
1450	chr17	18163047	18163281	20.00	19.63655	10.20667
1451	chr17	18163634	18164092	160.00	288.42978	81.14746
1452	chr17	19280193	19280372	16.00	14.10583	7.97780
1453	chr17	19552061	19552236	13.00	8.50004	5.24541
1454	chr17	19654105	19654280	13.00	11.18770	6.90300
1455	chr17	20946608	20946846	33.00	40.80316	18.12878
1456	chr17	21002645	21002935	50.00	40.91375	11.75657
1457	chr17	21030130	21030382	28.00	23.60167	9.96791
1458	chr17	21187656	21188064	101.00	122.40765	29.11374
1459	chr17	25592470	25592665	14.00	13.19662	7.91173
1460	chr17	25659522	25659883	49.00	54.07066	18.73361
1461	chr17	25856176	25856500	46.00	60.15939	23.68901
1462	chr17	26368076	26368817	245.00	497.51547	131.16704
1463	chr17	26663357	26663556	15.00	14.13432	8.24751
1464	chr17	27139409	27139781	164.00	305.64233	87.97790
1465	chr17	27434973	27435169	17.00	15.02847	8.27595
1466	chr17	27621018	27621391	115.00	177.04268	49.96021
1467	chr17	27907482	27907675	25.00	10.50281	4.32828
1468	chr17	28258239	28258448	21.00	9.12806	4.25326
1469	chr17	29151665	29151968	45.00	58.77430	23.35790
1470	chr17	33569827	33570206	224.00	405.33243	96.29643
1471	chr17	33570790	33571008	18.00	8.51003	4.37990
1472	chr17	33584460	33584645	17.00	14.16795	7.75245
1473	chr17	35404897	35405204	21.00	21.75046	11.17117
1474	chr17	36598772	36598954	16.00	13.03207	7.32175
1475	chr17	36829066	36829241	18.00	13.54559	7.11877
1476	chr17	36908730	36909141	231.00	422.07639	99.92041
1477	chr17	37225654	37225872	17.00	7.75030	4.14938
1478	chr17	37321118	37321419	30.00	27.02617	11.22187
1479	chr17	37558320	37558522	19.00	18.11170	9.58381
1480	chr17	38085759	38085946	19.00	15.65382	8.10137
1481	chr17	38255365	38255598	28.00	27.72231	12.33401
1482	chr17	38375989	38376184	15.00	13.93247	8.12449
1483	chr17	38574148	38574629	228.00	352.34317	65.36321
1484	chr17	38984140	38984521	16.00	13.03207	7.32175
1485	chr17	39006709	39006893	12.00	10.12670	6.45669
1486	chr17	39010650	39010880	22.00	17.98963	8.61746
1487	chr17	39845509	39845854	143.00	230.83266	60.86454

1488	chr17	39942908	39943451	248.00	487.55914	122.77478
1489	chr17	39966200	39966414	22.00	17.98963	8.61746
1490	chr17	40086674	40086973	17.00	12.48723	6.74410
1491	chr17	40176970	40177261	41.00	42.51218	15.73623
1492	chr17	40306889	40307266	201.00	300.08069	57.65663
1493	chr17	40404804	40405060	12.00	9.04351	5.78186
1494	chr17	40418870	40419269	19.00	11.44765	5.70858
1495	chr17	40540350	40540829	126.00	201.14066	56.11774
1496	chr17	40761445	40761760	46.00	41.74495	13.41516
1497	chr17	40819497	40819843	92.00	121.93128	33.66560
1498	chr17	41322963	41323327	156.00	287.51511	83.71230
1499	chr17	41633638	41633943	47.00	57.73001	21.77512
1500	chr17	41748365	41748783	45.00	48.21673	17.23492
1501	chr17	41977960	41978264	39.00	33.02968	11.41716
1502	chr17	43038132	43038315	18.00	13.54559	7.11877
1503	chr17	43138801	43139140	112.00	140.39027	32.25346
1504	chr17	43238898	43239257	127.00	172.62759	39.96154
1505	chr17	43248251	43248527	19.00	14.62602	7.49344
1506	chr17	43697521	43697703	17.00	17.20576	9.59759
1507	chr17	45177598	45177815	13.00	12.10322	7.46479
1508	chr17	45921733	45921909	18.00	11.47393	5.93179
1509	chr17	45972977	45973734	295.00	570.71844	128.29622
1510	chr17	46024314	46024634	55.00	63.10625	20.98164
1511	chr17	46100699	46101009	64.00	77.17039	24.35369
1512	chr17	46102408	46102620	18.00	12.53419	6.53070
1513	chr17	46125367	46125787	206.00	309.64682	59.08378
1514	chr17	46184750	46185016	21.00	17.13758	8.41110
1515	chr17	46680104	46680285	21.00	13.32019	6.27944
1516	chr17	46703773	46704044	39.00	43.25486	17.01243
1517	chr17	47207232	47207447	21.00	19.23886	9.65850
1518	chr17	47268439	47268629	23.00	19.14867	8.99213
1519	chr17	47270226	47270646	36.00	19.36777	6.15948
1520	chr17	47271148	47271518	75.00	72.92091	17.51960
1521	chr17	47427041	47427254	15.00	12.44159	7.21033
1522	chr17	47785470	47785805	66.00	91.09685	31.22668
1523	chr17	47841520	47841774	33.00	22.07791	7.83771
1524	chr17	47866313	47866769	134.00	229.24805	66.56464
1525	chr17	48133064	48133280	19.00	18.80980	10.00636
1526	chr17	48138539	48138831	31.00	36.46677	16.37107
1527	chr17	48501134	48501393	23.00	25.47769	12.75760
1528	chr17	48520790	48521038	30.00	27.02617	11.22187
1529	chr17	48619804	48619983	14.00	13.19662	7.91173
1530	chr17	49199854	49200053	14.00	13.29834	7.97350
1531	chr17	54237421	54237675	15.00	14.60207	8.53119
1532	chr17	54517821	54518011	15.00	13.54753	7.88914
1533	chr17	55370665	55370860	11.00	9.72353	6.39839
1534	chr17	55372493	55372668	13.00	8.50004	5.24541
1535	chr17	55740430	55740639	13.00	8.67630	5.35252

1536	chr17	56084990	56085291	24.00	20.32534	9.36681
1537	chr17	57232441	57232782	59.00	70.11027	22.93936
1538	chr17	57408808	57409168	106.00	172.57747	53.53401
1539	chr17	57784769	57784944	14.00	12.35372	7.39607
1540	chr17	57833755	57834004	15.00	14.23777	8.31042
1541	chr17	57923132	57923330	17.00	13.55040	7.37903
1542	chr17	58679274	58679685	147.00	267.33347	78.91351
1543	chr17	58754551	58755372	183.00	341.25385	93.43160
1544	chr17	58979289	58979522	14.00	13.33873	7.99799
1545	chr17	60747634	60747894	11.00	9.72353	6.39839
1546	chr17	60856675	60856923	22.00	17.98963	8.61746
1547	chr17	61627396	61627915	197.00	376.02563	102.06287
1548	chr17	61699175	61699896	172.00	319.80054	89.85643
1549	chr17	66097685	66097894	20.00	21.28387	11.19719
1550	chr17	66470446	66470710	29.00	26.45082	11.24017
1551	chr17	66818247	66818509	26.00	18.32292	7.70658
1552	chr17	67410730	67410905	12.00	10.35998	6.60115
1553	chr17	68087967	68088160	14.00	12.35372	7.39607
1554	chr17	68133527	68133838	42.00	52.69979	21.20207
1555	chr17	69436781	69436963	21.00	16.84896	8.24279
1556	chr17	70461779	70462010	18.00	13.54559	7.11877
1557	chr17	71358933	71359114	11.00	9.72353	6.39839
1558	chr17	72745949	72746124	11.00	9.69164	6.37880
1559	chr17	73043083	73043376	36.00	45.64589	19.72838
1560	chr17	73150541	73150915	131.00	202.64563	53.46901
1561	chr17	73285349	73285716	168.00	267.75577	63.31960
1562	chr17	73389783	73390234	185.00	339.95041	91.05190
1563	chr17	73511547	73511851	61.00	84.07985	29.92039
1564	chr17	73975181	73975509	65.00	67.50246	18.83831
1565	chr17	74025654	74025861	13.00	11.18770	6.90300
1566	chr17	74609225	74609448	29.00	26.45082	11.24017
1567	chr17	74667628	74668126	230.00	409.32559	93.57076
1568	chr17	74868281	74868557	21.00	22.68483	11.73039
1569	chr17	75283015	75283212	19.00	15.65382	8.10137
1570	chr17	76165496	76165817	49.00	67.74368	26.65997
1571	chr17	76588467	76588716	20.00	19.88133	10.35450
1572	chr17	76930317	76930534	19.00	15.65382	8.10137
1573	chr17	77771051	77771374	58.00	83.34073	31.11947
1574	chr17	77805411	77805593	11.00	9.72353	6.39839
1575	chr17	78244657	78244990	43.00	54.33144	21.69514
1576	chr17	78765767	78766013	28.00	28.81880	12.98280
1577	chr17	78806185	78806411	28.00	24.54824	10.49788
1578	chr17	79008567	79008838	38.00	46.51284	19.37006
1579	chr17	79196704	79197036	126.00	212.39325	62.62007
1580	chr17	79212798	79213237	199.00	331.12686	74.93444
1581	chr17	79316896	79317214	44.00	59.04880	23.99397
1582	chr17	79339620	79339889	22.00	18.29233	8.79342
1583	chr17	79367755	79368113	163.00	303.36707	87.44469

1584	chr17	79368910	79369312	103.00	172.39622	55.45273
1585	chr17	79633440	79633841	205.00	307.72937	58.79835
1586	chr17	80056700	80056886	10.00	8.58401	5.86519
1587	chr17	80215328	80215589	37.00	30.63221	10.84630
1588	chr17	80415889	80416100	23.00	19.14867	8.99213
1589	chr17	80477387	80477630	38.00	48.93276	20.79478
1590	chr17	80656542	80656857	105.00	176.55830	56.51913
1591	chr17	81009634	81009857	15.00	14.23777	8.31042
1592	chr18	158242	158517	29.00	34.52419	15.99598
1593	chr18	3089581	3089761	11.00	9.72353	6.39839
1594	chr18	3448049	3448382	33.00	38.91012	17.01081
1595	chr18	3451363	3451631	24.00	26.58739	13.08485
1596	chr18	3623902	3624261	81.00	125.83909	42.59091
1597	chr18	3983688	3983914	10.00	8.58401	5.86519
1598	chr18	7038572	7038773	18.00	15.41328	8.23523
1599	chr18	7048179	7048366	18.00	13.54559	7.11877
1600	chr18	7059975	7060154	15.00	10.44156	5.99476
1601	chr18	7878316	7879022	58.00	67.72969	22.10566
1602	chr18	8177402	8177613	15.00	13.73699	8.00508
1603	chr18	8843104	8843311	12.00	10.44029	6.65075
1604	chr18	8844804	8845086	35.00	43.41929	18.84219
1605	chr18	9017363	9017681	44.00	46.77608	16.86025
1606	chr18	10852460	10852688	14.00	12.44150	7.45002
1607	chr18	11120046	11120262	11.00	6.67667	4.49607
1608	chr18	11149512	11149719	20.00	19.05461	9.85493
1609	chr18	11176395	11176625	15.00	14.55884	8.50506
1610	chr18	11808051	11808226	16.00	13.84165	7.81618
1611	chr18	11850010	11850663	33.00	40.80316	18.12878
1612	chr18	11947308	11947665	106.00	171.24306	52.75864
1613	chr18	12946724	12946938	16.00	15.89157	9.06439
1614	chr18	18895559	18895819	22.00	22.56316	11.34064
1615	chr18	19804191	19804488	38.00	48.93276	20.79478
1616	chr18	20686572	20686861	15.00	10.44156	5.99476
1617	chr18	21572541	21572870	58.00	73.55672	25.41080
1618	chr18	22411949	22412147	15.00	14.34297	8.37430
1619	chr18	24057897	24058102	22.00	24.10503	12.26359
1620	chr18	28786838	28787179	42.00	55.51620	22.85736
1621	chr18	29523936	29524113	18.00	18.54332	10.13079
1622	chr18	29673535	29673898	42.00	43.92358	16.11090
1623	chr18	30111312	30111549	17.00	16.56128	9.20873
1624	chr18	42117351	42117556	14.00	12.44150	7.45002
1625	chr18	43170602	43170840	17.00	16.01209	8.87529
1626	chr18	45419456	45419748	32.00	30.30064	12.36418
1627	chr18	45457256	45457686	40.00	52.26358	21.86117
1628	chr18	47807958	47808212	21.00	21.21263	10.84757
1629	chr18	48405252	48405575	79.00	123.85088	42.65595
1630	chr18	48724148	48724494	94.00	153.87737	50.65394
1631	chr18	51749716	51749891	11.00	9.72353	6.39839

1632	chr18	52495679	52495854	10.00	8.55496	5.84723
1633	chr18	55102366	55102700	62.00	91.31023	33.59156
1634	chr18	55712822	55713032	15.00	14.60207	8.53119
1635	chr18	56206351	56206573	19.00	19.84845	10.63133
1636	chr18	59854216	59854539	52.00	73.06522	28.25957
1637	chr18	60193424	60193819	42.00	48.13118	18.51973
1638	chr18	60742431	60742640	14.00	13.33873	7.99799
1639	chr18	65265110	65265330	20.00	21.28387	11.19719
1640	chr18	67873225	67873550	91.00	147.78462	49.05434
1641	chr18	71959099	71959586	157.00	289.77155	84.24550
1642	chr18	72163303	72163617	29.00	34.52419	15.99598
1643	chr18	72922493	72922833	55.00	78.45982	29.85916
1644	chr18	74204824	74205193	106.00	149.00392	40.08992
1645	chr18	74783655	74783971	47.00	64.23869	25.59357
1646	chr19	556048	556294	16.00	15.89157	9.06439
1647	chr19	633535	633865	38.00	48.28117	20.41237
1648	chr19	752649	752904	29.00	33.82398	15.58204
1649	chr19	797122	797376	28.00	25.19533	10.86549
1650	chr19	1039790	1040232	191.00	358.31671	96.77213
1651	chr19	1040898	1041299	235.00	456.70331	116.36485
1652	chr19	1113468	1113703	22.00	22.56316	11.34064
1653	chr19	1260831	1261174	103.00	172.39622	55.45273
1654	chr19	1275338	1275638	55.00	75.30885	28.01780
1655	chr19	1479152	1479445	63.00	93.17426	34.12476
1656	chr19	1652740	1653179	222.00	401.94574	96.04419
1657	chr19	1790869	1791092	20.00	21.28387	11.19719
1658	chr19	2215507	2215776	15.00	14.60207	8.53119
1659	chr19	2375539	2375714	11.00	9.69164	6.37880
1660	chr19	2532906	2533197	25.00	26.41649	12.63683
1661	chr19	2588486	2588683	12.00	8.75023	5.59899
1662	chr19	2602561	2602736	14.00	9.45699	5.62008
1663	chr19	2648616	2648818	16.00	15.89157	9.06439
1664	chr19	2739977	2740335	163.00	303.36707	87.44469
1665	chr19	2841104	2841570	163.00	303.36707	87.44469
1666	chr19	2866860	2867303	43.00	45.34496	16.48558
1667	chr19	2900622	2900956	18.00	18.54332	10.13079
1668	chr19	2944885	2945290	138.00	208.78119	52.07943
1669	chr19	2950636	2950969	59.00	75.21384	25.84149
1670	chr19	2977120	2977472	148.00	269.56445	79.44670
1671	chr19	3031052	3031235	16.00	14.76748	8.38221
1672	chr19	3036124	3036464	116.00	147.04666	33.39518
1673	chr19	3047730	3047913	22.00	18.29233	8.79342
1674	chr19	3094224	3094447	23.00	25.54366	12.79678
1675	chr19	3274988	3275201	22.00	24.04191	12.22603
1676	chr19	3494123	3494411	26.00	29.96288	14.39638
1677	chr19	3573809	3574127	37.00	47.28369	20.26158
1678	chr19	3700554	3700825	40.00	52.26358	21.86117
1679	chr19	3971066	3971451	115.00	197.27957	61.66172

1680	chr19	4065891	4066150	49.00	67.74368	26.65997
1681	chr19	4086530	4086705	10.00	8.58401	5.86519
1682	chr19	4153367	4153734	111.00	189.14120	59.71833
1683	chr19	4182634	4182842	12.00	7.57257	4.87074
1684	chr19	4246775	4247184	247.00	433.26822	92.91870
1685	chr19	4402223	4402589	109.00	154.44899	41.21394
1686	chr19	4471599	4472301	157.00	289.77155	84.24550
1687	chr19	4474620	4474941	62.00	91.31023	33.59156
1688	chr19	4724425	4724638	17.00	17.20576	9.59759
1689	chr19	4840936	4841369	11.00	8.94684	5.91686
1690	chr19	4867556	4867952	150.00	263.56183	74.45378
1691	chr19	4911959	4912169	15.00	14.03257	8.18553
1692	chr19	4968503	4969040	224.00	344.50937	64.22150
1693	chr19	5171729	5171965	13.00	12.10322	7.46479
1694	chr19	5559556	5559757	14.00	12.80671	7.67394
1695	chr19	5575651	5575830	12.00	7.57257	4.87074
1696	chr19	5826719	5826925	13.00	12.10322	7.46479
1697	chr19	5904089	5904293	18.00	13.79201	7.26413
1698	chr19	5904501	5904704	12.00	5.61615	3.71058
1699	chr19	6110550	6110930	136.00	242.99040	73.04831
1700	chr19	6274162	6274488	75.00	106.45683	34.94292
1701	chr19	6279899	6280207	104.00	174.47520	55.98593
1702	chr19	6372411	6372826	191.00	354.68332	94.66971
1703	chr19	6424779	6425728	275.00	570.98175	146.71237
1704	chr19	6460564	6460790	9.00	7.48180	5.33199
1705	chr19	6515979	6516665	32.00	30.30064	12.36418
1706	chr19	6601748	6601937	13.00	12.10322	7.46479
1707	chr19	6692999	6693282	25.00	28.47325	13.86318
1708	chr19	6737554	6737998	180.00	319.39288	83.21931
1709	chr19	6753584	6753898	31.00	37.63698	17.06238
1710	chr19	6761167	6761389	14.00	13.33873	7.99799
1711	chr19	6800985	6801209	18.00	13.54559	7.11877
1712	chr19	6828758	6829101	29.00	29.34339	12.92074
1713	chr19	6829321	6829744	75.00	96.13466	29.05652
1714	chr19	6863508	6863750	21.00	22.68483	11.73039
1715	chr19	7438844	7439109	20.00	21.28387	11.19719
1716	chr19	7670501	7670873	62.00	91.31023	33.59156
1717	chr19	7694312	7694747	243.00	449.15164	105.08871
1718	chr19	7739043	7739570	134.00	221.48244	62.06965
1719	chr19	7894480	7895169	235.00	473.10898	125.83505
1720	chr19	8008577	8008906	50.00	66.01459	25.14664
1721	chr19	8070518	8070774	19.00	19.90304	10.66399
1722	chr19	8115541	8115838	21.00	13.32019	6.27944
1723	chr19	8372758	8373103	55.00	46.96013	12.90918
1724	chr19	8408020	8408383	174.00	306.30325	80.46066
1725	chr19	8426850	8427200	85.00	135.48483	45.71473
1726	chr19	8454803	8455385	237.00	411.62796	89.17198
1727	chr19	8477996	8478255	48.00	65.98680	26.12677

1728	chr19	8578615	8578917	27.00	31.46828	14.92958
1729	chr19	8590303	8590697	83.00	131.74779	44.78875
1730	chr19	9121121	9121400	27.00	29.57795	13.80600
1731	chr19	9129353	9129584	16.00	11.45212	6.36943
1732	chr19	9945762	9945981	15.00	14.44997	8.43918
1733	chr19	10530704	10531068	92.00	149.81091	49.58754
1734	chr19	10644409	10644649	20.00	12.37394	5.99401
1735	chr19	10694157	10694377	22.00	19.18340	9.31657
1736	chr19	10828101	10828860	268.00	527.57080	128.00137
1737	chr19	10946452	10946683	16.00	13.84165	7.81618
1738	chr19	10946908	10947104	25.00	25.27586	11.95416
1739	chr19	11266353	11266858	168.00	314.29056	89.83475
1740	chr19	11456945	11457278	41.00	35.46992	11.98801
1741	chr19	11484339	11484575	26.00	22.72887	10.11615
1742	chr19	11485047	11485927	137.00	235.62215	68.04385
1743	chr19	11545843	11546152	35.00	37.76629	15.50489
1744	chr19	11708037	11708327	44.00	59.04880	23.99397
1745	chr19	11750365	11750710	80.00	115.59489	37.24179
1746	chr19	11805664	11805950	28.00	32.98889	15.46278
1747	chr19	11849657	11850067	99.00	136.44197	37.46722
1748	chr19	11877617	11877984	74.00	113.89050	39.86749
1749	chr19	11924803	11925222	132.00	204.60258	53.87408
1750	chr19	12035692	12035996	89.00	143.74619	47.98794
1751	chr19	12163725	12164028	49.00	45.62112	14.27144
1752	chr19	12175221	12175612	44.00	59.04880	23.99397
1753	chr19	12202848	12203152	53.00	74.85551	28.79276
1754	chr19	12251122	12251437	51.00	57.04987	19.48295
1755	chr19	12267495	12267774	46.00	48.59421	17.01380
1756	chr19	12273590	12273905	33.00	31.61030	12.73885
1757	chr19	12405566	12405951	123.00	214.71704	66.11672
1758	chr19	12444367	12444780	129.00	218.68896	64.09928
1759	chr19	12476390	12476777	54.00	76.65376	29.32596
1760	chr19	12511969	12512159	26.00	22.72887	10.11615
1761	chr19	12551731	12552056	56.00	80.27357	30.39236
1762	chr19	12595540	12595879	48.00	52.59385	18.35894
1763	chr19	12662208	12662723	231.00	462.73901	123.32345
1764	chr19	12721503	12721792	33.00	38.49447	16.76443
1765	chr19	12814019	12814304	27.00	31.46828	14.92958
1766	chr19	12847700	12848041	53.00	60.06214	20.23230
1767	chr19	12979593	12979898	61.00	73.27170	23.70400
1768	chr19	13049151	13049468	69.00	73.25175	19.98002
1769	chr19	13273673	13273977	48.00	49.83319	16.84233
1770	chr19	13274778	13275136	32.00	18.22108	6.37989
1771	chr19	13781774	13781962	17.00	14.16795	7.75245
1772	chr19	13858449	13858726	31.00	32.86217	14.23227
1773	chr19	13905855	13906280	251.00	491.65170	122.48003
1774	chr19	13944320	13944737	148.00	239.62799	62.20557
1775	chr19	13957423	13957697	30.00	22.61202	8.84830

1776	chr19	14062848	14063387	261.00	432.49738	81.79628
1777	chr19	14117050	14117656	290.00	511.33392	100.02283
1778	chr19	14202738	14202935	21.00	20.70605	10.54219
1779	chr19	14228477	14228933	132.00	202.91422	52.92940
1780	chr19	14247682	14248124	86.00	98.72491	24.83231
1781	chr19	14248279	14248722	53.00	60.06214	20.23230
1782	chr19	14530027	14530477	147.00	200.50247	42.24347
1783	chr19	14607201	14607388	20.00	15.26912	7.60191
1784	chr19	14639877	14640312	214.00	362.55981	80.55452
1785	chr19	15218713	15218888	15.00	10.44156	5.99476
1786	chr19	15490152	15490526	123.00	191.54985	52.73853
1787	chr19	15529694	15530140	274.00	362.78714	45.77993
1788	chr19	15562936	15563279	84.00	118.31512	36.37865
1789	chr19	15609968	15610143	14.00	9.45699	5.62008
1790	chr19	15640919	15641126	18.00	14.67274	7.78897
1791	chr19	15947841	15948151	51.00	48.24844	14.84230
1792	chr19	16222405	16222855	184.00	323.32822	82.28030
1793	chr19	16291647	16291867	19.00	16.69150	8.72420
1794	chr19	16695408	16695668	22.00	17.48414	8.32590
1795	chr19	16830262	16830438	14.00	5.61393	3.45781
1796	chr19	16882322	16882644	47.00	43.02813	13.70059
1797	chr19	17400326	17400713	86.00	119.94786	36.10002
1798	chr19	17414094	17414450	202.00	332.61218	73.48512
1799	chr19	17445570	17445796	31.00	30.43339	12.80976
1800	chr19	17580833	17581203	69.00	92.83223	30.53470
1801	chr19	17727777	17728200	42.00	36.70545	12.27344
1802	chr19	17765920	17766221	35.00	24.13375	8.29876
1803	chr19	17858245	17858514	51.00	65.89905	24.57203
1804	chr19	17883057	17883312	44.00	39.20615	12.84430
1805	chr19	17970437	17970814	219.00	307.63477	50.71462
1806	chr19	18175630	18175892	26.00	22.72887	10.11615
1807	chr19	18264859	18265149	39.00	41.85611	16.20273
1808	chr19	18314787	18315343	263.00	468.25336	98.91346
1809	chr19	18392443	18392626	13.00	11.97110	7.38428
1810	chr19	18404251	18404488	19.00	14.62602	7.49344
1811	chr19	18414233	18414586	83.00	108.55029	31.47246
1812	chr19	18433778	18434106	85.00	120.53011	37.03946
1813	chr19	18633060	18633440	48.00	52.59385	18.35894
1814	chr19	18654314	18654578	60.00	87.43150	32.42556
1815	chr19	19030074	19030485	182.00	274.26019	57.13251
1816	chr19	19143169	19143542	120.00	167.74377	41.59025
1817	chr19	19145213	19145418	19.00	18.80980	10.00636
1818	chr19	19174238	19174863	224.00	344.50937	64.22150
1819	chr19	19249142	19249538	160.00	223.79462	45.95405
1820	chr19	19303878	19304073	21.00	16.84896	8.24279
1821	chr19	19496207	19496736	266.00	428.20535	76.20951
1822	chr19	19779290	19779711	142.00	256.22293	76.24751
1823	chr19	19843818	19844187	146.00	224.25620	55.07681

1824	chr19	19976383	19976728	97.00	160.01103	52.25354
1825	chr19	20748557	20748752	13.00	12.10322	7.46479
1826	chr19	20844319	20844590	26.00	29.96288	14.39638
1827	chr19	20958834	20959151	25.00	28.47325	13.86318
1828	chr19	21105762	21106106	30.00	36.07370	16.52918
1829	chr19	21203138	21203517	76.00	96.74823	28.84976
1830	chr19	21264714	21265055	63.00	93.17426	34.12476
1831	chr19	21324511	21324829	44.00	59.04880	23.99397
1832	chr19	21512204	21512379	12.00	10.89743	6.93159
1833	chr19	21541498	21541788	19.00	19.90304	10.66399
1834	chr19	21579604	21580015	134.00	238.60458	71.98191
1835	chr19	21688171	21688502	82.00	124.03819	40.92493
1836	chr19	21751982	21752318	88.00	76.10496	14.81605
1837	chr19	21950328	21950683	64.00	95.04503	34.65796
1838	chr19	22235031	22235281	18.00	18.54332	10.13079
1839	chr19	22605185	22605412	16.00	15.89157	9.06439
1840	chr19	23433131	23433551	186.00	356.33548	99.70828
1841	chr19	23578248	23578600	70.00	101.51529	35.00807
1842	chr19	23869940	23870231	34.00	42.40530	18.66198
1843	chr19	23941701	23941976	21.00	22.68483	11.73039
1844	chr19	24097352	24097731	124.00	208.21309	61.63393
1845	chr19	24269715	24270006	29.00	26.45082	11.24017
1846	chr19	29768149	29768366	20.00	21.28387	11.19719
1847	chr19	31557449	31557624	8.00	6.42036	4.79879
1848	chr19	32896416	32896719	85.00	133.09958	44.33034
1849	chr19	33165982	33166354	136.00	242.99040	73.04831
1850	chr19	33462863	33463240	96.00	131.12227	36.34320
1851	chr19	33864385	33864706	80.00	125.81718	43.18915
1852	chr19	35264089	35264294	13.00	10.43506	6.43685
1853	chr19	35804527	35804724	14.00	13.29834	7.97350
1854	chr19	36087398	36087579	14.00	12.35372	7.39607
1855	chr19	36134576	36134919	44.00	59.04880	23.99397
1856	chr19	36138919	36139277	68.00	97.84283	34.02193
1857	chr19	36193134	36193917	209.00	315.41168	59.94006
1858	chr19	36239187	36239624	184.00	342.33267	93.24398
1859	chr19	36248704	36249123	120.00	174.70982	45.33533
1860	chr19	36542629	36542856	25.00	26.72232	12.81986
1861	chr19	36545335	36545568	15.00	10.44156	5.99476
1862	chr19	36822577	36822943	73.00	112.16877	39.45675
1863	chr19	37742257	37742897	140.00	220.37328	57.11462
1864	chr19	37808500	37808845	101.00	168.25076	54.38633
1865	chr19	38146912	38147087	16.00	14.76748	8.38221
1866	chr19	38791453	38791628	9.00	7.07618	5.07780
1867	chr19	38795641	38795917	25.00	28.22109	13.71367
1868	chr19	38806728	38806935	25.00	21.51895	9.74148
1869	chr19	38826356	38826538	13.00	12.10322	7.46479
1870	chr19	38865018	38865467	24.00	23.93016	11.49438
1871	chr19	39034643	39034917	25.00	24.00721	11.19798

1872	chr19	39062418	39062656	25.00	21.51895	9.74148
1873	chr19	39086749	39086964	11.00	9.72353	6.39839
1874	chr19	39140508	39140709	12.00	10.89743	6.93159
1875	chr19	39174250	39174460	13.00	10.43506	6.43685
1876	chr19	39340491	39341478	176.00	333.14569	94.37629
1877	chr19	39421380	39421655	29.00	30.81632	13.79326
1878	chr19	39832833	39833034	16.00	15.89157	9.06439
1879	chr19	39881726	39882056	66.00	84.00542	27.13957
1880	chr19	40283820	40284178	30.00	36.07370	16.52918
1881	chr19	40561720	40561948	13.00	12.10322	7.46479
1882	chr19	40596413	40596615	16.00	14.76748	8.38221
1883	chr19	40971794	40971975	19.00	11.44765	5.70858
1884	chr19	41082475	41083150	218.00	371.01984	82.05321
1885	chr19	41152058	41152297	19.00	14.62602	7.49344
1886	chr19	41220913	41221271	166.00	310.20068	89.04430
1887	chr19	41222133	41222444	32.00	39.21360	17.59558
1888	chr19	41256528	41256754	15.00	14.23777	8.31042
1889	chr19	41283799	41284033	15.00	10.44156	5.99476
1890	chr19	41284456	41284758	34.00	40.02687	17.25750
1891	chr19	41468844	41469021	17.00	9.65886	5.13772
1892	chr19	41769813	41770076	18.00	17.39272	9.43670
1893	chr19	41869993	41870259	33.00	34.90736	14.64351
1894	chr19	42498334	42498633	30.00	36.07370	16.52918
1895	chr19	42746823	42747052	20.00	19.51683	10.13433
1896	chr19	42772844	42773501	248.00	473.18268	114.48403
1897	chr19	42901006	42901457	189.00	253.96552	43.79899
1898	chr19	43942657	43942832	13.00	9.77812	6.02968
1899	chr19	43979459	43979679	22.00	11.75739	5.30198
1900	chr19	45244556	45245005	19.00	19.05697	10.15561
1901	chr19	45256602	45256881	26.00	29.15619	13.91766
1902	chr19	45409696	45409931	29.00	34.44115	15.94700
1903	chr19	45681743	45682327	53.00	65.38303	23.25734
1904	chr19	45808747	45808958	18.00	18.54332	10.13079
1905	chr19	45873739	45874144	241.00	487.73132	129.03424
1906	chr19	45926828	45927197	55.00	71.43149	25.74741
1907	chr19	46087386	46087924	190.00	312.49881	71.56239
1908	chr19	46119203	46119486	27.00	31.00451	14.65503
1909	chr19	46144031	46144312	22.00	20.07057	9.84364
1910	chr19	46180584	46180961	49.00	64.01712	24.47631
1911	chr19	46220580	46221109	65.00	78.76765	24.72836
1912	chr19	46273920	46274198	32.00	37.17467	16.39005
1913	chr19	46282829	46283118	28.00	29.40866	13.33348
1914	chr19	46296016	46296310	60.00	87.60279	32.52516
1915	chr19	46498284	46498818	243.00	395.40170	76.17669
1916	chr19	46532213	46532549	42.00	55.63624	22.92757
1917	chr19	46850644	46850926	25.00	28.47325	13.86318
1918	chr19	47220188	47220549	91.00	146.22754	48.15226
1919	chr19	47221450	47221625	13.00	9.77812	6.02968

1920	chr19	47287111	47287286	9.00	7.48180	5.33199
1921	chr19	47367172	47367361	16.00	14.96948	8.50540
1922	chr19	47551765	47552193	253.00	516.44690	135.01791
1923	chr19	47787600	47787782	20.00	15.26912	7.60191
1924	chr19	47818639	47818833	14.00	13.33873	7.99799
1925	chr19	47931430	47931736	13.00	12.10322	7.46479
1926	chr19	48111838	48112160	37.00	47.28369	20.26158
1927	chr19	48638674	48638920	23.00	25.54366	12.79678
1928	chr19	48673690	48673954	32.00	39.21360	17.59558
1929	chr19	48835895	48836557	86.00	118.43068	35.24094
1930	chr19	48972175	48972656	216.00	328.93475	61.93806
1931	chr19	49061747	49061931	14.00	13.33873	7.99799
1932	chr19	49087476	49087790	58.00	67.72969	22.10566
1933	chr19	49140208	49140392	15.00	14.55884	8.50506
1934	chr19	49375314	49375658	59.00	85.75960	31.99196
1935	chr19	49496612	49496954	82.00	120.64671	38.95042
1936	chr19	49622422	49622647	19.00	19.90304	10.66399
1937	chr19	49955041	49955334	50.00	68.65258	26.69310
1938	chr19	49999356	49999749	173.00	326.23587	92.77669
1939	chr19	50179909	50180573	182.00	333.20346	89.58331
1940	chr19	50269938	50270265	115.00	197.27957	61.66172
1941	chr19	50320665	50320996	81.00	124.73872	41.95086
1942	chr19	50372452	50372627	16.00	11.45212	6.36943
1943	chr19	50384875	50385227	53.00	60.06214	20.23230
1944	chr19	50433046	50433420	107.00	150.81496	40.46460
1945	chr19	50449816	50450018	12.00	10.89743	6.93159
1946	chr19	51161415	51161802	55.00	78.30276	29.76773
1947	chr19	51308115	51308471	101.00	168.25076	54.38633
1948	chr19	51842838	51843122	22.00	17.98963	8.61746
1949	chr19	51897413	51897778	116.00	167.29005	43.83665
1950	chr19	52429925	52430289	126.00	212.39325	62.62007
1951	chr19	52692861	52693225	23.00	25.54366	12.79678
1952	chr19	54481458	54481762	35.00	44.01965	19.19518
1953	chr19	54640667	54641552	130.00	229.87161	69.84911
1954	chr19	54663483	54663921	50.00	46.93057	14.55687
1955	chr19	54974870	54975481	41.00	42.51218	15.73623
1956	chr19	55582374	55582656	27.00	31.46828	14.92958
1957	chr19	55591967	55592319	95.00	155.64645	51.03039
1958	chr19	55791748	55792049	47.00	64.10442	25.51520
1959	chr19	55850040	55850243	15.00	13.54753	7.88914
1960	chr19	55850441	55851342	239.00	482.84998	127.96785
1961	chr19	55919426	55919838	108.00	182.83191	58.11873
1962	chr19	55952118	55952307	15.00	10.44156	5.99476
1963	chr19	55973034	55973630	159.00	294.29260	85.31190
1964	chr19	56826501	56826676	12.00	10.89743	6.93159
1965	chr19	57922288	57922613	82.00	129.76570	44.25555
1966	chr19	57946509	57946707	16.00	11.45212	6.36943
1967	chr19	58144287	58144640	132.00	217.36501	61.15010

1968	chr19	58257931	58258274	124.00	216.51817	66.44582
1969	chr19	58326185	58326532	66.00	80.37143	25.10304
1970	chr19	58330836	58331193	135.00	240.79591	72.51511
1971	chr19	58360962	58361230	39.00	50.59282	21.32797
1972	chr19	58427957	58428205	47.00	64.23869	25.59357
1973	chr19	58609595	58609878	24.00	26.99996	13.32998
1974	chr19	58892444	58892847	134.00	201.11771	50.58075
1975	chr19	59084100	59084439	62.00	91.31023	33.59156
1976	chr19	59084641	59084906	30.00	35.98782	16.47856
1977	chr2	437828	438272	89.00	103.37780	25.68860
1978	chr2	443851	444059	22.00	16.06620	7.52567
1979	chr2	1800181	1800362	14.00	9.45699	5.62008
1980	chr2	3487061	3487323	12.00	9.83210	6.27363
1981	chr2	3618889	3619144	39.00	28.38507	9.22084
1982	chr2	8782847	8783023	18.00	10.54228	5.42315
1983	chr2	8818870	8819047	15.00	13.54753	7.88914
1984	chr2	8821941	8822270	165.00	297.43109	82.44692
1985	chr2	9144029	9144242	20.00	19.75804	10.28005
1986	chr2	9562924	9563249	63.00	75.57976	23.97902
1987	chr2	10037638	10037972	46.00	56.37351	21.46449
1988	chr2	10548506	10548778	44.00	45.75088	16.28981
1989	chr2	10638034	10638325	28.00	28.53476	12.81429
1990	chr2	10952855	10953201	119.00	187.80013	53.37100
1991	chr2	11530603	11530844	36.00	25.17959	8.52928
1992	chr2	11722855	11723100	15.00	12.92712	7.50851
1993	chr2	12003879	12004152	33.00	36.23242	15.42405
1994	chr2	16607026	16607404	12.00	9.04351	5.78186
1995	chr2	20251364	20251729	92.00	142.26035	45.20096
1996	chr2	20252637	20252920	35.00	34.26693	13.48820
1997	chr2	20379858	20380169	47.00	53.10804	19.10234
1998	chr2	20551036	20551275	22.00	22.42758	11.25910
1999	chr2	20643138	20643335	17.00	12.48723	6.74410
2000	chr2	20646497	20646861	139.00	217.19962	56.04272
2001	chr2	23491847	23492076	15.00	10.44156	5.99476
2002	chr2	24270255	24270536	27.00	28.76973	13.32356
2003	chr2	24346045	24346380	56.00	64.63990	21.35632
2004	chr2	25194403	25194959	160.00	248.01561	58.28131
2005	chr2	25735789	25736010	20.00	15.72746	7.86812
2006	chr2	25829887	25830078	15.00	12.67947	7.35640
2007	chr2	26220621	26220927	47.00	62.15504	24.37346
2008	chr2	26565766	26566100	48.00	59.33968	22.22877
2009	chr2	26568642	26568998	101.00	140.01013	38.21656
2010	chr2	27008513	27009023	248.00	487.55914	122.77478
2011	chr2	27543979	27544188	20.00	16.81057	8.50643
2012	chr2	27545811	27546056	28.00	23.60167	9.96791
2013	chr2	28152780	28153492	77.00	114.55594	38.45957
2014	chr2	28215940	28216197	30.00	32.23818	14.25303
2015	chr2	28308151	28308405	16.00	12.59157	7.05403

2016	chr2	28600965	28601147	21.00	13.32019	6.27944
2017	chr2	28819540	28819766	28.00	14.70246	5.60657
2018	chr2	28885047	28885364	49.00	54.07066	18.73361
2019	chr2	29245840	29246131	43.00	45.34496	16.48558
2020	chr2	29256345	29256520	20.00	15.72746	7.86812
2021	chr2	30369464	30369950	52.00	69.11088	25.94489
2022	chr2	30497678	30498008	50.00	36.32626	9.85984
2023	chr2	33523253	33523428	10.00	8.06847	5.54424
2024	chr2	33682999	33683178	16.00	11.08796	6.15393
2025	chr2	36595027	36595202	12.00	8.21418	5.26589
2026	chr2	36959588	36959856	23.00	18.61959	8.68790
2027	chr2	37553512	37553860	70.00	86.84992	26.60172
2028	chr2	37603822	37604172	40.00	43.30234	16.60780
2029	chr2	37605579	37605860	18.00	17.28003	9.36836
2030	chr2	38830100	38830441	144.00	195.19603	41.38719
2031	chr2	40590750	40590999	14.00	12.09821	7.23880
2032	chr2	40662109	40662457	91.00	129.80171	38.64577
2033	chr2	42158371	42158701	56.00	54.95864	16.26945
2034	chr2	42190740	42191178	34.00	27.12109	9.99001
2035	chr2	42217006	42217341	91.00	79.88412	15.31547
2036	chr2	42220260	42220584	31.00	29.00390	11.98951
2037	chr2	42311497	42311706	18.00	12.53419	6.53070
2038	chr2	43269038	43269493	24.00	20.65621	9.55807
2039	chr2	43385421	43385596	17.00	10.09093	5.37321
2040	chr2	43690441	43690699	45.00	48.21673	17.23492
2041	chr2	43864171	43864545	201.00	335.29053	75.68378
2042	chr2	44175511	44175689	19.00	16.50842	8.61383
2043	chr2	44353511	44354000	64.00	77.17039	24.35369
2044	chr2	44395021	44396045	236.00	409.47382	88.79731
2045	chr2	44460751	44461001	15.00	13.01197	7.56063
2046	chr2	45225145	45225445	30.00	33.25076	14.85491
2047	chr2	45236522	45237061	164.00	282.92999	74.85199
2048	chr2	45241637	45241821	15.00	13.09803	7.61346
2049	chr2	45400788	45401012	12.00	5.61615	3.71058
2050	chr2	46526698	46526976	41.00	44.75902	17.01287
2051	chr2	46926089	46926305	32.00	35.47324	15.38030
2052	chr2	47259546	47259850	64.00	77.08248	24.30506
2053	chr2	47630117	47630359	38.00	27.30554	8.99032
2054	chr2	53255090	53255265	17.00	12.48723	6.74410
2055	chr2	53429249	53429538	34.00	36.33072	15.07420
2056	chr2	53700565	53700776	12.00	8.75023	5.59899
2057	chr2	53704049	53704293	19.00	18.33757	9.72064
2058	chr2	54784802	54784997	21.00	15.66308	7.56186
2059	chr2	54798211	54798429	19.00	16.50842	8.61383
2060	chr2	54956014	54956216	24.00	18.22131	8.18007
2061	chr2	55277602	55277978	128.00	189.71912	48.33271
2062	chr2	55746484	55746796	25.00	26.72232	12.81986
2063	chr2	55961940	55962200	24.00	26.99996	13.32998

2064	chr2	56006668	56007163	18.00	13.79201	7.26413
2065	chr2	56070492	56070768	21.00	16.84896	8.24279
2066	chr2	56185379	56185610	13.00	9.77812	6.02968
2067	chr2	56219557	56219758	13.00	10.03086	6.18621
2068	chr2	61765356	61765961	207.00	311.56635	59.36921
2069	chr2	61794249	61794434	14.00	11.54338	6.89663
2070	chr2	61935316	61935491	19.00	13.55649	6.87442
2071	chr2	62081546	62081989	126.00	163.94209	36.24947
2072	chr2	62932274	62932897	236.00	368.10107	67.64664
2073	chr2	63271108	63271333	17.00	12.48723	6.74410
2074	chr2	63277785	63278134	119.00	172.84944	44.96066
2075	chr2	63446244	63446638	66.00	84.00542	27.13957
2076	chr2	63815463	63815809	44.00	52.04960	19.88424
2077	chr2	63950713	63950921	18.00	18.49157	10.09976
2078	chr2	64067841	64068285	225.00	385.90076	84.67592
2079	chr2	64245228	64245513	17.00	9.65886	5.13772
2080	chr2	64978022	64978686	137.00	242.84496	72.22839
2081	chr2	65058357	65058624	24.00	24.32677	11.73205
2082	chr2	65357418	65357630	13.00	12.10322	7.46479
2083	chr2	65454624	65455065	175.00	330.34113	93.55572
2084	chr2	65593878	65594053	16.00	11.45212	6.36943
2085	chr2	65664013	65664188	13.00	11.18770	6.90300
2086	chr2	65681172	65681376	9.00	7.48180	5.33199
2087	chr2	65890110	65890285	18.00	10.54228	5.42315
2088	chr2	66662460	66662757	31.00	35.46775	15.77829
2089	chr2	67691005	67691315	51.00	33.66012	8.65657
2090	chr2	67788780	67789017	18.00	18.54332	10.13079
2091	chr2	68443671	68443851	12.00	10.89743	6.93159
2092	chr2	69056566	69056895	61.00	72.41879	23.22968
2093	chr2	69198649	69198975	12.00	9.97716	6.36384
2094	chr2	69481152	69481479	24.00	16.27042	7.13572
2095	chr2	69614298	69614618	50.00	69.50916	27.19317
2096	chr2	69870237	69870465	15.00	10.44156	5.99476
2097	chr2	69971084	69971290	17.00	12.48723	6.74410
2098	chr2	70006489	70006767	15.00	12.67947	7.35640
2099	chr2	70141698	70142090	17.00	15.02847	8.27595
2100	chr2	70360176	70360400	16.00	13.84165	7.81618
2101	chr2	70370659	70370964	30.00	36.07370	16.52918
2102	chr2	71454163	71454829	79.00	101.77402	29.97378
2103	chr2	71970028	71970382	37.00	36.11132	13.75584
2104	chr2	72342620	72342894	39.00	39.72020	14.98689
2105	chr2	73298318	73298731	193.00	359.23260	95.65585
2106	chr2	73460297	73460637	70.00	106.40540	37.85715
2107	chr2	74055893	74056166	48.00	62.33617	23.98679
2108	chr2	74425422	74425805	71.00	108.32056	38.39035
2109	chr2	74685181	74685613	230.00	396.58771	86.54928
2110	chr2	74881098	74881353	32.00	35.12276	15.17258
2111	chr2	75819243	75819419	14.00	11.54338	6.89663

2112	chr2	85147295	85147540	21.00	22.68483	11.73039
2113	chr2	85685078	85685270	15.00	13.54753	7.88914
2114	chr2	85969680	85969855	15.00	12.06501	6.97936
2115	chr2	86162934	86163194	12.00	7.57257	4.87074
2116	chr2	88316408	88316667	27.00	31.46828	14.92958
2117	chr2	88926811	88927561	238.00	372.05899	68.21750
2118	chr2	88959058	88959345	44.00	46.77608	16.86025
2119	chr2	96814294	96814497	16.00	15.89157	9.06439
2120	chr2	96874492	96874798	73.00	112.16877	39.45675
2121	chr2	97222684	97222872	16.00	11.08796	6.15393
2122	chr2	97405809	97406054	18.00	13.54559	7.11877
2123	chr2	97454165	97454388	37.00	26.23695	8.75980
2124	chr2	98279860	98280197	93.00	145.34668	46.34871
2125	chr2	98280441	98280725	54.00	72.21973	26.73175
2126	chr2	98310061	98310330	21.00	22.68483	11.73039
2127	chr2	99485839	99486069	29.00	31.96617	14.47761
2128	chr2	101956527	101956857	20.00	21.22644	11.16290
2129	chr2	101976717	101976950	24.00	26.75781	13.18622
2130	chr2	101991716	101991937	17.00	13.40451	7.29123
2131	chr2	102500433	102500703	24.00	26.99996	13.32998
2132	chr2	106127838	106128075	23.00	19.14867	8.99213
2133	chr2	109582314	109582568	19.00	15.65382	8.10137
2134	chr2	110461457	110461710	19.00	16.24178	8.45341
2135	chr2	110859167	110859471	29.00	34.52419	15.99598
2136	chr2	110863598	110863882	39.00	43.62424	17.22766
2137	chr2	112456370	112456680	56.00	57.99500	17.79537
2138	chr2	113032884	113033250	202.00	393.91620	108.23947
2139	chr2	113034626	113034822	13.00	9.77812	6.02968
2140	chr2	113099774	113099960	18.00	15.86018	8.50597
2141	chr2	113341956	113342178	20.00	17.80660	9.10210
2142	chr2	113404525	113404843	36.00	32.42414	12.10651
2143	chr2	113626855	113627114	21.00	10.91615	5.07146
2144	chr2	118845699	118846068	33.00	30.84510	12.30785
2145	chr2	120301544	120301889	47.00	51.12580	17.98426
2146	chr2	121003019	121003408	66.00	65.89429	17.67545
2147	chr2	121352466	121352669	22.00	17.48414	8.32590
2148	chr2	121427376	121427551	14.00	11.46841	6.85037
2149	chr2	121669532	121669709	15.00	11.71088	6.76273
2150	chr2	121672708	121672883	18.00	13.13437	6.87792
2151	chr2	121693281	121693462	15.00	10.20576	5.85418
2152	chr2	121832769	121832978	22.00	17.98963	8.61746
2153	chr2	122392150	122392463	26.00	22.72887	10.11615
2154	chr2	127415337	127415563	25.00	12.21501	5.02658
2155	chr2	128267751	128268012	21.00	16.84896	8.24279
2156	chr2	128284847	128285155	69.00	81.23402	24.06047
2157	chr2	128617603	128617885	11.00	7.75405	5.16830
2158	chr2	128690159	128690530	98.00	157.08472	49.89813
2159	chr2	130998608	130998985	43.00	45.34496	16.48558

2160	chr2	131148705	131149089	41.00	42.51218	15.73623
2161	chr2	131565482	131565711	13.00	11.60865	7.16234
2162	chr2	136288211	136288494	31.00	34.90310	15.44278
2163	chr2	136288641	136289138	222.00	340.60400	63.65064
2164	chr2	143701885	143702133	14.00	12.71320	7.61671
2165	chr2	144857793	144858010	18.00	7.00940	3.67327
2166	chr2	150268001	150268298	30.00	36.07370	16.52918
2167	chr2	150851037	150851357	51.00	48.24844	14.84230
2168	chr2	151044623	151044900	20.00	20.52682	10.74351
2169	chr2	151383398	151383598	11.00	9.72353	6.39839
2170	chr2	153573875	153574253	120.00	199.89453	59.66164
2171	chr2	154336135	154336431	17.00	17.20576	9.59759
2172	chr2	157056184	157056451	19.00	19.44286	10.38803
2173	chr2	157292772	157293019	26.00	22.72887	10.11615
2174	chr2	158103372	158103647	28.00	27.59230	12.25744
2175	chr2	159810932	159811240	57.00	44.16847	11.21315
2176	chr2	160890298	160890622	62.00	55.74403	14.52282
2177	chr2	160896069	160896270	14.00	12.09821	7.23880
2178	chr2	161085061	161085316	16.00	13.03207	7.32175
2179	chr2	161110281	161110456	15.00	11.64254	6.72100
2180	chr2	161113495	161113857	92.00	124.09187	34.84451
2181	chr2	161225839	161226133	19.00	17.36905	9.13383
2182	chr2	161991479	161991762	31.00	29.00390	11.98951
2183	chr2	169312545	169312831	43.00	54.87289	22.01398
2184	chr2	170685037	170685281	20.00	21.28387	11.19719
2185	chr2	171243328	171243515	12.00	10.05135	6.40993
2186	chr2	171571637	171571885	27.00	31.46828	14.92958
2187	chr2	172903543	172903869	50.00	46.93057	14.55687
2188	chr2	173557695	173557936	8.00	6.42036	4.79879
2189	chr2	174455070	174455308	17.00	12.48723	6.74410
2190	chr2	174829915	174830367	197.00	368.35794	97.62814
2191	chr2	175471184	175471491	16.00	15.07286	8.56837
2192	chr2	176033065	176033240	11.00	8.94684	5.91686
2193	chr2	178076924	178077266	27.00	29.57795	13.80600
2194	chr2	183716868	183717108	18.00	10.54228	5.42315
2195	chr2	189168367	189168595	13.00	12.10322	7.46479
2196	chr2	190244037	190244314	28.00	32.98889	15.46278
2197	chr2	190417796	190417971	12.00	10.89743	6.93159
2198	chr2	190648978	190649297	101.00	168.25076	54.38633
2199	chr2	191399722	191400040	31.00	37.63698	17.06238
2200	chr2	191745276	191745596	50.00	69.36634	27.10990
2201	chr2	196941573	196941846	15.00	14.60207	8.53119
2202	chr2	198669076	198669436	113.00	193.36678	60.78473
2203	chr2	200603598	200603810	13.00	12.10322	7.46479
2204	chr2	201527625	201527811	13.00	12.10322	7.46479
2205	chr2	201828286	201828689	191.00	314.55969	71.93706
2206	chr2	201983221	201983473	15.00	14.60207	8.53119
2207	chr2	203050642	203050922	10.00	8.58401	5.86519

2208	chr2	206613496	206613711	15.00	13.64153	7.94669
2209	chr2	206809348	206809674	65.00	78.76765	24.72836
2210	chr2	208299536	208299732	10.00	8.58401	5.86519
2211	chr2	208497588	208497820	16.00	11.45212	6.36943
2212	chr2	211418260	211418435	11.00	9.72353	6.39839
2213	chr2	211423964	211424207	13.00	12.10322	7.46479
2214	chr2	212022658	212022907	25.00	26.72232	12.81986
2215	chr2	213092569	213092783	12.00	10.89743	6.93159
2216	chr2	213951445	213951669	15.00	14.60207	8.53119
2217	chr2	216103546	216103726	18.00	13.54559	7.11877
2218	chr2	217557792	217558141	90.00	145.76303	48.52114
2219	chr2	219134745	219135134	186.00	353.15506	97.87469
2220	chr2	219151854	219152210	60.00	70.84866	22.85500
2221	chr2	219188715	219188890	14.00	11.54338	6.89663
2222	chr2	219252717	219252899	24.00	11.41743	4.83325
2223	chr2	219824072	219824443	146.00	265.10544	78.38030
2224	chr2	220094152	220094579	172.00	311.92889	85.30135
2225	chr2	220408604	220408895	23.00	23.93211	11.83371
2226	chr2	220462461	220462659	19.00	11.44765	5.70858
2227	chr2	220572715	220572911	16.00	15.89157	9.06439
2228	chr2	220929461	220929739	34.00	40.45509	17.51112
2229	chr2	221089931	221090184	20.00	21.28387	11.19719
2230	chr2	224588762	224588989	19.00	18.57014	9.86143
2231	chr2	224661981	224662166	21.00	17.13758	8.41110
2232	chr2	224679803	224680111	9.00	7.26095	5.19401
2233	chr2	226983959	226984231	19.00	18.80980	10.00636
2234	chr2	227019456	227019734	36.00	30.40245	11.04494
2235	chr2	227050474	227050816	15.00	13.36375	7.77651
2236	chr2	227521152	227521436	26.00	18.32292	7.70658
2237	chr2	227656295	227656520	27.00	28.01568	12.87371
2238	chr2	230273125	230273310	11.00	6.67667	4.49607
2239	chr2	230320063	230320256	15.00	14.55884	8.50506
2240	chr2	232545112	232545303	14.00	13.09665	7.85091
2241	chr2	232571590	232571974	137.00	166.03798	31.81190
2242	chr2	232572918	232573232	27.00	30.09214	14.11260
2243	chr2	232645880	232646294	174.00	275.74097	63.34925
2244	chr2	233789963	233790149	15.00	14.34297	8.37430
2245	chr2	233816445	233816690	14.00	13.33873	7.99799
2246	chr2	233853133	233853415	37.00	47.28369	20.26158
2247	chr2	233894054	233894411	49.00	58.98667	21.53457
2248	chr2	234159858	234160034	13.00	10.72323	6.61555
2249	chr2	234295379	234295597	28.00	25.58257	11.08736
2250	chr2	234475265	234475565	51.00	57.04987	19.48295
2251	chr2	238571345	238571601	20.00	15.72746	7.86812
2252	chr2	238599416	238599607	12.00	10.89743	6.93159
2253	chr2	238600329	238600601	27.00	23.95451	10.49082
2254	chr2	239050621	239050846	20.00	12.37394	5.99401
2255	chr2	239197376	239197762	196.00	379.75699	105.04028

2256	chr2	239593625	239593833	18.00	13.79201	7.26413
2257	chr2	240196893	240197226	38.00	45.35899	18.68843
2258	chr2	240322573	240323274	96.00	157.96201	51.72034
2259	chr2	240610239	240610428	13.00	12.10322	7.46479
2260	chr2	241327052	241327352	56.00	76.36037	28.10507
2261	chr2	241351799	241352024	28.00	25.19533	10.86549
2262	chr2	241497304	241497619	38.00	48.93276	20.79478
2263	chr2	242088777	242089104	80.00	120.22924	39.93878
2264	chr20	442418	442615	15.00	14.60207	8.53119
2265	chr20	2083196	2083371	12.00	10.89743	6.93159
2266	chr20	2489764	2489985	20.00	21.28387	11.19719
2267	chr20	3027364	3027650	30.00	27.72049	11.61484
2268	chr20	3869633	3870081	146.00	265.10544	78.38030
2269	chr20	5093549	5093853	36.00	45.64589	19.72838
2270	chr20	5676601	5676870	35.00	43.91954	19.13640
2271	chr20	6145250	6145520	16.00	15.89157	9.06439
2272	chr20	6571611	6571887	21.00	22.68483	11.73039
2273	chr20	9870324	9870522	14.00	13.33873	7.99799
2274	chr20	10333099	10333306	15.00	10.44156	5.99476
2275	chr20	10414968	10415317	36.00	45.64589	19.72838
2276	chr20	13766823	13767005	13.00	12.10322	7.46479
2277	chr20	13975901	13976250	104.00	174.47520	55.98593
2278	chr20	17662811	17663138	94.00	153.87737	50.65394
2279	chr20	21182993	21183239	15.00	14.60207	8.53119
2280	chr20	22666852	22667027	13.00	12.10322	7.46479
2281	chr20	23331176	23331560	100.00	166.18437	53.85313
2282	chr20	24975566	24975805	18.00	18.54332	10.13079
2283	chr20	30181416	30181659	20.00	15.72746	7.86812
2284	chr20	30311327	30311989	185.00	269.83624	53.08977
2285	chr20	30410520	30410764	19.00	14.62602	7.49344
2286	chr20	30448158	30448333	17.00	12.09955	6.51592
2287	chr20	30591262	30591437	15.00	12.67947	7.35640
2288	chr20	31687632	31688056	72.00	109.00928	38.20777
2289	chr20	32307957	32308349	195.00	322.82559	73.43575
2290	chr20	32414307	32414489	15.00	12.21280	7.06995
2291	chr20	32803869	32804048	24.00	16.27042	7.13572
2292	chr20	33103976	33104344	169.00	254.52715	55.62449
2293	chr20	33463869	33464194	20.00	19.63655	10.20667
2294	chr20	34035188	34035395	16.00	14.38123	8.14624
2295	chr20	34330243	34330654	225.00	427.74960	108.29706
2296	chr20	34345078	34345259	15.00	10.44156	5.99476
2297	chr20	34360071	34360412	98.00	140.50027	40.34176
2298	chr20	34605620	34605952	48.00	58.35112	21.65173
2299	chr20	35510354	35510529	15.00	7.96262	4.56686
2300	chr20	35807199	35807590	231.00	398.73074	86.92395
2301	chr20	36802266	36802503	29.00	28.03230	12.15205
2302	chr20	36812636	36812937	44.00	46.77608	16.86025
2303	chr20	36998080	36998382	25.00	25.14172	11.87397

2304	chr20	39765353	39765729	80.00	124.44805	42.39492
2305	chr20	39778376	39778625	38.00	27.30554	8.99032
2306	chr20	39945776	39946009	18.00	17.05967	9.23460
2307	chr20	40104740	40105156	53.00	58.82412	19.54777
2308	chr20	40246930	40247314	254.00	498.79340	123.93813
2309	chr20	42817548	42817754	14.00	11.93405	7.13762
2310	chr20	43461489	43461724	23.00	19.46545	9.17574
2311	chr20	44407846	44408021	10.00	7.13828	4.95710
2312	chr20	44440855	44441260	172.00	275.80307	64.81829
2313	chr20	44485898	44486367	295.00	487.69955	84.48695
2314	chr20	44489368	44489655	29.00	29.34339	12.92074
2315	chr20	44509618	44510058	210.00	291.33603	48.63993
2316	chr20	44622219	44622447	20.00	16.81057	8.50643
2317	chr20	44827885	44828060	15.00	13.73699	8.00508
2318	chr20	45035143	45035560	223.00	431.30347	112.07120
2319	chr20	45360542	45360763	20.00	15.72746	7.86812
2320	chr20	45810051	45810356	30.00	33.97421	15.28521
2321	chr20	45986298	45986557	36.00	21.94582	7.15321
2322	chr20	46069968	46070241	16.00	8.79855	4.85229
2323	chr20	46093068	46093631	90.00	129.67125	39.19292
2324	chr20	47663194	47663474	56.00	74.04485	26.74908
2325	chr20	48430205	48430461	29.00	18.11946	6.91563
2326	chr20	48808897	48809085	18.00	13.26099	6.95184
2327	chr20	48929265	48929440	16.00	10.30820	5.69940
2328	chr20	48948273	48948559	36.00	33.55295	12.71768
2329	chr20	48949213	48949483	39.00	31.26531	10.55251
2330	chr20	49039696	49039895	15.00	13.54753	7.88914
2331	chr20	49113417	49113592	11.00	9.23007	6.09337
2332	chr20	49178466	49178689	24.00	21.62983	10.12671
2333	chr20	49359782	49359988	13.00	12.06567	7.44193
2334	chr20	49406872	49407100	12.00	8.21418	5.26589
2335	chr20	49598012	49598187	19.00	14.62602	7.49344
2336	chr20	50413814	50414012	18.00	15.41328	8.23523
2337	chr20	50418909	50419271	72.00	95.03972	30.10734
2338	chr20	50471693	50471938	16.00	15.17788	8.63227
2339	chr20	50516326	50516550	23.00	21.32582	10.27162
2340	chr20	50535450	50535659	18.00	13.54559	7.11877
2341	chr20	50549876	50550051	10.00	7.29675	5.05753
2342	chr20	50555221	50555446	14.00	13.29834	7.97350
2343	chr20	50656738	50657043	19.00	19.90304	10.66399
2344	chr20	50721759	50722082	83.00	83.97982	19.36376
2345	chr20	51563393	51563601	15.00	13.54753	7.88914
2346	chr20	51590713	51590988	25.00	25.41176	12.03543
2347	chr20	51691451	51691821	86.00	118.06103	35.03252
2348	chr20	51698520	51698698	16.00	13.84165	7.81618
2349	chr20	52516161	52516438	37.00	32.61494	11.86358
2350	chr20	52550973	52551148	20.00	13.98452	6.87126
2351	chr20	52679265	52679569	57.00	56.32385	16.55487

2352	chr20	52683998	52684354	65.00	67.50246	18.83831
2353	chr20	52739943	52740644	35.00	24.13375	8.29876
2354	chr20	54823133	54823362	38.00	31.82546	11.13173
2355	chr20	55616011	55616353	39.00	39.72020	14.98689
2356	chr20	56172274	56172603	71.00	88.48494	26.97640
2357	chr20	56273180	56273450	40.00	30.77538	10.03841
2358	chr20	56292902	56293134	30.00	25.07416	10.14329
2359	chr20	56293460	56293814	121.00	155.45006	34.82232
2360	chr20	56596575	56596925	56.00	48.19299	13.13970
2361	chr20	57425825	57426231	189.00	287.76190	59.31791
2362	chr20	57607258	57607581	81.00	111.78385	34.44514
2363	chr20	58569701	58570038	54.00	64.08788	22.01678
2364	chr20	58786238	58786463	30.00	22.61202	8.84830
2365	chr20	59325266	59325586	47.00	55.61530	20.54324
2366	chr20	60813150	60813685	211.00	359.20724	81.05241
2367	chr20	60877753	60878093	116.00	174.20370	47.67663
2368	chr20	61334977	61335168	20.00	10.09393	4.84094
2369	chr20	61556019	61556253	33.00	22.07791	7.83771
2370	chr20	61643272	61643550	31.00	29.00390	11.98951
2371	chr20	62466283	62466628	120.00	194.25854	56.39444
2372	chr20	62526198	62526420	19.00	15.90023	8.24859
2373	chr20	62697370	62697592	20.00	15.72746	7.86812
2374	chr20	62726865	62727118	20.00	12.37394	5.99401
2375	chr21	17101552	17101837	16.00	15.89157	9.06439
2376	chr21	17566673	17566920	28.00	25.19533	10.86549
2377	chr21	17791550	17791813	20.00	21.28387	11.19719
2378	chr21	17960763	17960994	9.00	7.48180	5.33199
2379	chr21	28215191	28215486	21.00	16.84896	8.24279
2380	chr21	30257194	30257610	147.00	267.33347	78.91351
2381	chr21	30365164	30365533	90.00	125.57040	36.86121
2382	chr21	30397041	30397481	144.00	250.60376	71.49535
2383	chr21	30541187	30541394	13.00	12.10322	7.46479
2384	chr21	30604903	30605191	14.00	11.54338	6.89663
2385	chr21	33835755	33835993	10.00	8.58401	5.86519
2386	chr21	33867675	33867852	16.00	14.76748	8.38221
2387	chr21	33997109	33997402	12.00	10.05135	6.40993
2388	chr21	34100652	34100852	18.00	18.54332	10.13079
2389	chr21	34494423	34494757	61.00	81.65821	28.50606
2390	chr21	34576238	34576507	11.00	6.67667	4.49607
2391	chr21	35013884	35014771	122.00	178.44127	46.08468
2392	chr21	37470266	37470514	21.00	22.68483	11.73039
2393	chr21	37692210	37692568	21.00	22.68483	11.73039
2394	chr21	38160446	38160649	15.00	13.01197	7.56063
2395	chr21	38711421	38711683	19.00	19.84845	10.63133
2396	chr21	38738853	38739225	68.00	102.59337	36.79076
2397	chr21	38919406	38919590	16.00	8.79855	4.85229
2398	chr21	40282313	40282488	20.00	10.09393	4.84094
2399	chr21	43050553	43050883	30.00	29.35740	12.55712

2400	chr21	43098523	43098801	38.00	34.97009	12.76091
2401	chr21	43099283	43099476	20.00	15.72746	7.86812
2402	chr21	43203794	43204006	14.00	12.35372	7.39607
2403	chr21	43206682	43206885	22.00	17.98963	8.61746
2404	chr21	43648296	43648570	37.00	36.97105	14.23754
2405	chr21	43779123	43779334	19.00	18.57014	9.86143
2406	chr21	43883974	43884350	139.00	210.70490	52.45411
2407	chr21	44394556	44394921	186.00	349.33652	95.66843
2408	chr21	44842536	44842863	46.00	49.66670	17.60959
2409	chr21	44866442	44866647	17.00	14.84749	8.16567
2410	chr21	44921833	44922099	27.00	23.95451	10.49082
2411	chr21	45006121	45006300	12.00	5.61615	3.71058
2412	chr21	45066560	45066801	24.00	20.32534	9.36681
2413	chr21	45132431	45132619	15.00	10.44156	5.99476
2414	chr21	46294534	46294751	15.00	13.93247	8.12449
2415	chr21	46471103	46471306	17.00	12.48723	6.74410
2416	chr21	46947596	46947853	29.00	34.52419	15.99598
2417	chr21	47393856	47394094	19.00	14.62602	7.49344
2418	chr21	47604221	47604811	131.00	232.04996	70.38232
2419	chr22	18484524	18484783	25.00	26.72232	12.81986
2420	chr22	19259040	19259232	21.00	22.62456	11.69446
2421	chr22	19435132	19435513	130.00	223.26727	66.02682
2422	chr22	19718520	19718758	18.00	18.54332	10.13079
2423	chr22	19794012	19794362	17.00	17.20576	9.59759
2424	chr22	19862330	19862544	25.00	19.32458	8.50728
2425	chr22	19868638	19868944	49.00	45.62112	14.27144
2426	chr22	20004314	20004655	129.00	227.69656	69.31592
2427	chr22	20008295	20008648	76.00	117.76775	40.93063
2428	chr22	21399976	21400313	99.00	164.12225	53.31993
2429	chr22	23412144	23412485	87.00	137.63301	45.70732
2430	chr22	23487464	23487691	17.00	12.48723	6.74410
2431	chr22	23516092	23516333	30.00	27.72049	11.61484
2432	chr22	24384199	24384478	20.00	21.28387	11.19719
2433	chr22	24951101	24951353	17.00	12.48723	6.74410
2434	chr22	25079914	25080110	12.00	7.57257	4.87074
2435	chr22	25202047	25202222	12.00	10.05135	6.40993
2436	chr22	25631857	25632224	150.00	273.60745	80.26656
2437	chr22	27054819	27055092	49.00	31.63393	8.32362
2438	chr22	28114305	28114546	19.00	17.47089	9.19550
2439	chr22	29137742	29137998	21.00	19.99769	10.11506
2440	chr22	29468693	29468937	32.00	39.21360	17.59558
2441	chr22	29853918	29854104	13.00	12.10322	7.46479
2442	chr22	29999090	29999529	221.00	377.38568	83.17722
2443	chr22	30018119	30018372	29.00	31.45995	14.17617
2444	chr22	30129412	30129649	20.00	21.28387	11.19719
2445	chr22	30158294	30158496	12.00	10.89743	6.93159
2446	chr22	30303782	30304052	29.00	34.44115	15.94700
2447	chr22	30592514	30592689	16.00	13.03207	7.32175

2448	chr22	30631704	30631965	30.00	27.72049	11.61484
2449	chr22	30723043	30723314	59.00	81.63708	29.58428
2450	chr22	31226078	31226307	15.00	13.18532	7.66705
2451	chr22	31475391	31475608	13.00	12.10322	7.46479
2452	chr22	32954830	32955115	30.00	34.35212	15.50985
2453	chr22	32980837	32981166	42.00	27.87303	8.31320
2454	chr22	35695966	35696334	103.00	170.63409	54.43298
2455	chr22	35874028	35874296	14.00	13.33873	7.99799
2456	chr22	36232066	36232241	15.00	10.44156	5.99476
2457	chr22	36236456	36236708	21.00	21.89025	11.25511
2458	chr22	36316716	36316920	11.00	9.72353	6.39839
2459	chr22	36616443	36616618	13.00	12.10322	7.46479
2460	chr22	36783993	36784560	175.00	281.86487	65.94231
2461	chr22	36877555	36877791	28.00	27.98681	12.49005
2462	chr22	36925282	36925517	19.00	19.90304	10.66399
2463	chr22	37172095	37172679	196.00	344.69876	84.84621
2464	chr22	37374124	37374301	14.00	13.33873	7.99799
2465	chr22	37582276	37582687	189.00	277.34338	54.23149
2466	chr22	37997555	37997949	98.00	134.66437	37.09255
2467	chr22	38054233	38054676	225.00	448.88614	120.50305
2468	chr22	38142063	38142308	32.00	39.21360	17.59558
2469	chr22	38149882	38150096	13.00	11.18770	6.90300
2470	chr22	38240282	38240714	200.00	389.18781	107.17307
2471	chr22	39101263	39101850	249.00	489.94507	123.26785
2472	chr22	39151910	39152201	78.00	117.42355	39.52511
2473	chr22	39190057	39190407	141.00	189.91646	40.53090
2474	chr22	39916035	39916265	11.00	9.72353	6.39839
2475	chr22	39928762	39929120	72.00	77.62866	20.83631
2476	chr22	40440082	40440886	205.00	400.44046	109.50272
2477	chr22	40573730	40574009	28.00	29.40866	13.33348
2478	chr22	41032572	41033094	224.00	446.47427	119.96986
2479	chr22	41107813	41108013	14.00	13.33873	7.99799
2480	chr22	41156272	41156569	30.00	34.16175	15.39671
2481	chr22	41347456	41347817	124.00	216.87173	66.64992
2482	chr22	41763101	41763352	25.00	28.40159	13.82073
2483	chr22	41864676	41864851	25.00	14.38764	5.99355
2484	chr22	42017123	42017376	25.00	28.47325	13.86318
2485	chr22	42109103	42109342	13.00	12.10322	7.46479
2486	chr22	42342757	42342949	26.00	11.23803	4.49476
2487	chr22	42475804	42476214	125.00	135.00281	24.35960
2488	chr22	43411136	43411529	139.00	210.70490	52.45411
2489	chr22	45641910	45642434	45.00	60.76950	24.52717
2490	chr22	45981325	45981513	16.00	14.76748	8.38221
2491	chr22	46038332	46038676	14.00	10.20871	6.07602
2492	chr22	46154670	46154952	22.00	20.07057	9.84364
2493	chr22	46924151	46924374	22.00	21.29036	10.57483
2494	chr22	47589086	47589319	15.00	14.60207	8.53119
2495	chr22	50327849	50328030	13.00	8.50004	5.24541

2496	chr22	50364462	50364683	20.00	21.28387	11.19719
2497	chr22	50639129	50639464	46.00	62.49952	25.06037
2498	chr22	50682842	50683162	90.00	120.60435	34.09517
2499	chr22	50738698	50738985	23.00	25.14815	12.56146
2500	chr22	50946966	50947172	19.00	16.50842	8.61383
2501	chr3	4575498	4575733	16.00	13.03207	7.32175
2502	chr3	5038375	5038590	14.00	12.80671	7.67394
2503	chr3	5059905	5060212	16.00	13.84165	7.81618
2504	chr3	9438601	9439329	106.00	178.34319	56.87762
2505	chr3	9690958	9691148	15.00	14.60207	8.53119
2506	chr3	9885535	9885962	198.00	329.04825	74.55977
2507	chr3	10018745	10018940	18.00	17.28003	9.36836
2508	chr3	10052811	10053079	13.00	12.10322	7.46479
2509	chr3	10233882	10234116	18.00	10.54228	5.42315
2510	chr3	10392819	10392994	12.00	10.05135	6.40993
2511	chr3	11641951	11642197	26.00	22.12900	9.77388
2512	chr3	11759296	11759559	26.00	22.72887	10.11615
2513	chr3	12391786	12392007	28.00	24.54824	10.49788
2514	chr3	12395435	12395638	17.00	14.16795	7.75245
2515	chr3	12447295	12447519	15.00	12.44159	7.21033
2516	chr3	12486695	12486882	22.00	11.75739	5.30198
2517	chr3	13521472	13521814	90.00	104.93843	25.97403
2518	chr3	14473755	14474028	30.00	32.56670	14.44816
2519	chr3	14989705	14990096	180.00	323.39954	85.52958
2520	chr3	15140540	15140729	15.00	11.31229	6.51988
2521	chr3	15248314	15248550	19.00	17.16931	9.01292
2522	chr3	15642892	15643332	199.00	296.26920	57.08578
2523	chr3	15672144	15672467	80.00	107.52744	32.61648
2524	chr3	15687670	15687965	32.00	18.22108	6.37989
2525	chr3	16306497	16306737	18.00	15.32712	8.18314
2526	chr3	16938949	16939328	103.00	143.59521	38.96591
2527	chr3	16939573	16939748	13.00	8.50004	5.24541
2528	chr3	17771994	17772169	15.00	7.96262	4.56686
2529	chr3	18015011	18015204	22.00	8.39626	3.82887
2530	chr3	20096039	20096364	41.00	30.57590	9.68188
2531	chr3	24406775	24406969	17.00	12.09955	6.51592
2532	chr3	25469615	25469853	19.00	9.29158	4.61042
2533	chr3	25706274	25706668	159.00	277.51480	75.60625
2534	chr3	27525981	27526239	36.00	43.12796	18.24364
2535	chr3	27563058	27563296	28.00	20.43819	8.27744
2536	chr3	27565668	27565843	20.00	10.09393	4.84094
2537	chr3	27799521	27799696	9.00	7.07618	5.07780
2538	chr3	29725294	29725555	51.00	57.04987	19.48295
2539	chr3	30647782	30648138	101.00	122.40765	29.11374
2540	chr3	32010588	32010859	17.00	12.48723	6.74410
2541	chr3	33481956	33482634	184.00	351.67859	98.64188
2542	chr3	33523721	33523896	9.00	7.48180	5.33199
2543	chr3	37034649	37035352	206.00	403.39847	110.37227

2544	chr3	38178463	38178746	28.00	31.02884	14.29907
2545	chr3	38206459	38206850	113.00	163.35114	43.58478
2546	chr3	38538064	38538239	9.00	7.45558	5.31567
2547	chr3	39329937	39330167	27.00	26.64490	12.05936
2548	chr3	42543861	42544220	115.00	189.57698	57.19628
2549	chr3	43663688	43664004	49.00	54.07066	18.73361
2550	chr3	45120883	45121107	19.00	16.50842	8.61383
2551	chr3	46920527	46920721	14.00	13.19662	7.91173
2552	chr3	48229823	48230163	76.00	96.74823	28.84976
2553	chr3	48481418	48481615	14.00	13.33873	7.99799
2554	chr3	48594163	48594481	27.00	31.00451	14.65503
2555	chr3	49044608	49044934	42.00	43.92358	16.11090
2556	chr3	49625034	49625255	19.00	19.90304	10.66399
2557	chr3	49711596	49711831	17.00	17.20576	9.59759
2558	chr3	49823894	49824328	240.00	485.28973	128.50105
2559	chr3	50201605	50201826	18.00	15.32712	8.18314
2560	chr3	50337090	50337375	26.00	25.31819	11.62867
2561	chr3	50605099	50605433	116.00	167.29005	43.83665
2562	chr3	51381914	51382116	17.00	12.48723	6.74410
2563	chr3	52091268	52091443	11.00	8.30943	5.51730
2564	chr3	52254575	52254815	23.00	25.54366	12.79678
2565	chr3	52489280	52489564	50.00	55.55605	19.10828
2566	chr3	53164360	53164653	54.00	51.00921	15.09456
2567	chr3	53838851	53839033	14.00	9.45699	5.62008
2568	chr3	53926052	53926309	15.00	12.92712	7.50851
2569	chr3	54390489	54390680	14.00	9.45699	5.62008
2570	chr3	55203656	55203996	18.00	13.54559	7.11877
2571	chr3	55403516	55403948	36.00	35.61321	13.86287
2572	chr3	55654905	55655086	19.00	17.47089	9.19550
2573	chr3	55920001	55920204	14.00	12.99838	7.79102
2574	chr3	56166320	56166546	24.00	26.09272	12.78990
2575	chr3	56501170	56501458	57.00	77.41483	28.18985
2576	chr3	56717215	56717390	12.00	10.89743	6.93159
2577	chr3	57261515	57261859	85.00	135.72733	45.85514
2578	chr3	57344368	57344567	10.00	8.55496	5.84723
2579	chr3	57937252	57937521	24.00	26.93115	13.28916
2580	chr3	58449520	58449753	20.00	16.81057	8.50643
2581	chr3	61236978	61237390	109.00	184.93109	58.65193
2582	chr3	66024819	66025138	60.00	87.60279	32.52516
2583	chr3	67568648	67569056	11.00	9.72353	6.39839
2584	chr3	69131181	69131432	21.00	19.99769	10.11506
2585	chr3	69249616	69249872	36.00	35.61321	13.86287
2586	chr3	69281025	69281373	47.00	55.83940	20.67319
2587	chr3	69819350	69819526	10.00	8.48181	5.80194
2588	chr3	70873989	70874292	31.00	36.88874	16.62084
2589	chr3	71627456	71627658	14.00	11.54338	6.89663
2590	chr3	72388279	72388595	25.00	28.47325	13.86318
2591	chr3	72710381	72710558	17.00	12.48723	6.74410

2592	chr3	72772270	72772526	42.00	44.50830	16.43987
2593	chr3	80032437	80032619	13.00	12.10322	7.46479
2594	chr3	81501675	81501869	18.00	18.54332	10.13079
2595	chr3	88108072	88108466	201.00	391.55093	107.70627
2596	chr3	94973719	94973957	31.00	29.00390	11.98951
2597	chr3	100211101	100211561	169.00	305.25836	83.82214
2598	chr3	101231515	101231715	13.00	12.10322	7.46479
2599	chr3	101497764	101498159	94.00	153.87737	50.65394
2600	chr3	105587782	105588618	79.00	101.77402	29.97378
2601	chr3	107241529	107241962	237.00	477.97583	126.90145
2602	chr3	111395424	111395733	36.00	39.38707	16.03703
2603	chr3	111454197	111454464	19.00	19.71095	10.54897
2604	chr3	111458170	111458353	21.00	10.91615	5.07146
2605	chr3	111697549	111697833	41.00	43.08278	16.05755
2606	chr3	111804955	111805163	15.00	14.60207	8.53119
2607	chr3	113415572	113415968	147.00	262.79150	76.28942
2608	chr3	114866011	114867010	138.00	247.38885	74.11471
2609	chr3	115377235	115377410	10.00	8.55496	5.84723
2610	chr3	115503344	115503519	9.00	7.48180	5.33199
2611	chr3	119396198	119396373	15.00	7.96262	4.56686
2612	chr3	119864359	119864678	42.00	43.92358	16.11090
2613	chr3	120613129	120613304	14.00	12.35372	7.39607
2614	chr3	120626629	120626894	22.00	24.10503	12.26359
2615	chr3	121468576	121468762	14.00	11.54338	6.89663
2616	chr3	121720165	121720342	15.00	13.09803	7.61346
2617	chr3	122134801	122135067	37.00	38.99604	15.39259
2618	chr3	122694363	122694609	19.00	18.80980	10.00636
2619	chr3	122696727	122696937	12.00	10.68938	6.80412
2620	chr3	122920440	122920773	55.00	63.10625	20.98164
2621	chr3	123304511	123304686	12.00	10.60494	6.75222
2622	chr3	123488268	123488523	11.00	9.72353	6.39839
2623	chr3	123595854	123596070	14.00	13.29834	7.97350
2624	chr3	127842529	127842945	182.00	264.23029	52.23349
2625	chr3	132136666	132136939	32.00	36.97464	16.27136
2626	chr3	133207938	133208182	19.00	19.90304	10.66399
2627	chr3	133291888	133292303	49.00	67.60371	26.57833
2628	chr3	133812885	133813094	12.00	10.86272	6.91037
2629	chr3	134018084	134018315	22.00	23.57287	11.94623
2630	chr3	134030638	134031268	25.00	27.69739	13.40219
2631	chr3	136580733	136581127	133.00	236.41647	71.44872
2632	chr3	138048756	138048990	14.00	13.09665	7.85091
2633	chr3	138109209	138109384	8.00	6.42036	4.79879
2634	chr3	138187835	138188050	16.00	15.61544	8.89770
2635	chr3	139108348	139108655	18.00	18.49157	10.09976
2636	chr3	139254008	139254183	12.00	7.57257	4.87074
2637	chr3	140661399	140661659	19.00	19.90304	10.66399
2638	chr3	140996400	140996637	11.00	9.72353	6.39839
2639	chr3	142297696	142297894	14.00	13.33873	7.99799

2640	chr3	142837756	142838026	34.00	40.02687	17.25750
2641	chr3	142838520	142838716	13.00	12.10322	7.46479
2642	chr3	148804121	148804559	196.00	324.89761	73.81042
2643	chr3	149099943	149100261	26.00	22.72887	10.11615
2644	chr3	149530727	149531106	123.00	206.12819	61.14085
2645	chr3	150026310	150026784	26.00	26.63790	12.41393
2646	chr3	150037811	150038112	32.00	24.84073	9.41915
2647	chr3	150062863	150063179	37.00	45.16198	19.01208
2648	chr3	150264431	150264994	24.00	26.93115	13.28916
2649	chr3	150944037	150944226	14.00	11.54338	6.89663
2650	chr3	151036177	151036437	18.00	18.54332	10.13079
2651	chr3	151987296	151987643	25.00	28.22109	13.71367
2652	chr3	153453036	153453252	19.00	11.44765	5.70858
2653	chr3	156443611	156443797	19.00	11.44765	5.70858
2654	chr3	157335859	157336034	21.00	16.84896	8.24279
2655	chr3	157827447	157827696	16.00	15.89157	9.06439
2656	chr3	158362436	158362769	57.00	82.09486	30.92556
2657	chr3	158440772	158441162	21.00	20.95565	10.69270
2658	chr3	159368531	159368841	29.00	26.45082	11.24017
2659	chr3	159542095	159542537	19.00	19.57577	10.46788
2660	chr3	167804438	167804668	16.00	15.17788	8.63227
2661	chr3	167809878	167810162	16.00	14.66876	8.32195
2662	chr3	167813640	167813975	73.00	102.84084	34.02337
2663	chr3	169945813	169946074	15.00	14.60207	8.53119
2664	chr3	171177022	171177390	60.00	87.60279	32.52516
2665	chr3	171178151	171178399	23.00	19.14867	8.99213
2666	chr3	171529270	171529592	36.00	35.61321	13.86287
2667	chr3	171814768	171815040	36.00	39.21373	15.93558
2668	chr3	171858560	171858803	11.00	8.13511	5.40775
2669	chr3	172259934	172260199	23.00	19.14867	8.99213
2670	chr3	172281216	172281449	13.00	12.06567	7.44193
2671	chr3	177448581	177448782	11.00	9.61136	6.32938
2672	chr3	177541800	177542034	15.00	14.60207	8.53119
2673	chr3	179170066	179170285	14.00	11.54338	6.89663
2674	chr3	179280432	179280745	20.00	15.72746	7.86812
2675	chr3	181429647	181429822	13.00	12.10322	7.46479
2676	chr3	181569000	181569275	21.00	16.84896	8.24279
2677	chr3	184037192	184037394	13.00	12.10322	7.46479
2678	chr3	184279156	184279678	86.00	136.86108	45.88803
2679	chr3	185053635	185053829	18.00	10.54228	5.42315
2680	chr3	185270194	185270378	10.00	8.58401	5.86519
2681	chr3	185815494	185815669	19.00	11.44765	5.70858
2682	chr3	185826836	185827193	156.00	243.86388	58.82354
2683	chr3	186475095	186475302	14.00	13.33873	7.99799
2684	chr3	186501772	186502094	42.00	43.92358	16.11090
2685	chr3	186945703	186945882	19.00	16.50842	8.61383
2686	chr3	189073812	189074036	20.00	21.28387	11.19719
2687	chr3	189491070	189491279	15.00	14.60207	8.53119

2688	chr3	189655326	189655551	16.00	11.67045	6.49949
2689	chr3	193830369	193830590	19.00	18.68905	9.93336
2690	chr3	193853599	193853885	26.00	25.31819	11.62867
2691	chr3	194014178	194014413	20.00	17.71077	9.04452
2692	chr3	194041571	194041967	59.00	69.28559	22.48033
2693	chr3	194053893	194054132	21.00	16.84896	8.24279
2694	chr3	194085580	194085935	17.00	15.02847	8.27595
2695	chr3	194489779	194490280	44.00	59.04880	23.99397
2696	chr3	195163658	195164084	113.00	193.36678	60.78473
2697	chr3	196014532	196014744	25.00	21.51895	9.74148
2698	chr3	196758266	196758575	30.00	27.72049	11.61484
2699	chr3	197676644	197676880	19.00	19.84845	10.63133
2700	chr4	52882	53266	171.00	222.73088	39.64961
2701	chr4	124059	124437	140.00	146.79681	23.47262
2702	chr4	206191	206428	23.00	25.54366	12.79678
2703	chr4	467882	468286	186.00	356.33548	99.70828
2704	chr4	603282	603616	13.00	12.10322	7.46479
2705	chr4	680945	681261	41.00	35.46992	11.98801
2706	chr4	699278	699471	16.00	15.89157	9.06439
2707	chr4	776026	776394	48.00	65.98680	26.12677
2708	chr4	1004575	1004774	19.00	19.90304	10.66399
2709	chr4	1037418	1037672	24.00	25.31873	12.32679
2710	chr4	1075555	1075814	19.00	18.57014	9.86143
2711	chr4	1779616	1779854	14.00	13.33873	7.99799
2712	chr4	2263736	2264096	99.00	163.83989	53.15666
2713	chr4	2470571	2470893	69.00	104.49630	37.32396
2714	chr4	3076119	3076487	74.00	114.10165	39.98995
2715	chr4	6271219	6271654	113.00	193.36678	60.78473
2716	chr4	6711613	6711818	17.00	12.48723	6.74410
2717	chr4	6785222	6785670	29.00	33.82398	15.58204
2718	chr4	6836667	6836914	26.00	28.14226	13.31293
2719	chr4	7069693	7070065	172.00	304.81235	81.18581
2720	chr4	7104709	7104957	23.00	25.47769	12.75760
2721	chr4	8412434	8412690	23.00	19.14867	8.99213
2722	chr4	8596145	8596361	11.00	9.72353	6.39839
2723	chr4	8601549	8601803	18.00	10.54228	5.42315
2724	chr4	13546548	13546919	111.00	181.38985	55.22400
2725	chr4	14728638	14728813	9.00	7.48180	5.33199
2726	chr4	14890568	14890743	10.00	8.26989	5.67016
2727	chr4	15915401	15915624	17.00	12.48723	6.74410
2728	chr4	16122461	16122744	30.00	33.78941	15.17532
2729	chr4	18023339	18023666	56.00	80.11367	30.29930
2730	chr4	23335851	23336072	15.00	10.44156	5.99476
2731	chr4	23891535	23891907	91.00	131.51392	39.62361
2732	chr4	24262684	24263013	54.00	66.74472	23.53913
2733	chr4	25236340	25236595	48.00	57.40865	21.10388
2734	chr4	26068710	26068965	19.00	14.62602	7.49344
2735	chr4	36056651	36056865	18.00	10.54228	5.42315

2736	chr4	38277107	38277308	12.00	10.89743	6.93159
2737	chr4	38676360	38676583	18.00	13.13437	6.87792
2738	chr4	38677181	38677390	17.00	12.71960	6.88181
2739	chr4	40517998	40518226	14.00	13.33873	7.99799
2740	chr4	40911407	40911582	13.00	6.36973	3.99600
2741	chr4	41321674	41321922	17.00	17.15686	9.56820
2742	chr4	41408005	41408501	13.00	12.10322	7.46479
2743	chr4	42154252	42154496	23.00	25.54366	12.79678
2744	chr4	47376557	47376855	39.00	39.72020	14.98689
2745	chr4	48130182	48130362	16.00	11.45212	6.36943
2746	chr4	48700480	48700666	10.00	8.58401	5.86519
2747	chr4	48782193	48782747	229.00	457.90009	122.26031
2748	chr4	52708870	52709394	189.00	363.33817	101.30788
2749	chr4	53588407	53588767	108.00	152.62999	40.83927
2750	chr4	54948305	54948638	50.00	69.50916	27.19317
2751	chr4	55364767	55365078	34.00	42.06298	18.46070
2752	chr4	55434495	55434670	12.00	10.86272	6.91037
2753	chr4	56813567	56813753	15.00	12.76090	7.40642
2754	chr4	57688085	57688329	16.00	15.89157	9.06439
2755	chr4	74176921	74177192	27.00	31.46828	14.92958
2756	chr4	74486086	74486269	11.00	9.69164	6.37880
2757	chr4	77507229	77507465	36.00	35.61321	13.86287
2758	chr4	78741237	78741574	48.00	65.98680	26.12677
2759	chr4	79487127	79487477	71.00	95.97116	31.20719
2760	chr4	80572731	80572906	11.00	6.67667	4.49607
2761	chr4	83205903	83206269	69.00	104.49630	37.32396
2762	chr4	83821649	83821963	19.00	14.62602	7.49344
2763	chr4	83996880	83997083	17.00	12.71960	6.88181
2764	chr4	84074126	84074361	13.00	8.50004	5.24541
2765	chr4	84456583	84456850	19.00	18.57014	9.86143
2766	chr4	86416834	86417009	10.00	8.58401	5.86519
2767	chr4	87515248	87515530	40.00	49.46666	20.21593
2768	chr4	87928186	87928446	18.00	17.16903	9.30100
2769	chr4	88226085	88226304	18.00	18.36122	10.02152
2770	chr4	88343792	88344019	18.00	16.23862	8.73573
2771	chr4	89066246	89066504	20.00	15.72746	7.86812
2772	chr4	89205546	89205999	170.00	316.44141	89.50039
2773	chr4	89513419	89513752	73.00	112.16877	39.45675
2774	chr4	90674557	90675186	64.00	95.04503	34.65796
2775	chr4	94238822	94239030	14.00	13.33873	7.99799
2776	chr4	94580682	94580859	13.00	12.06567	7.44193
2777	chr4	95428999	95429182	16.00	14.76748	8.38221
2778	chr4	95438224	95438403	17.00	12.48723	6.74410
2779	chr4	100484773	100484989	29.00	15.56141	5.79990
2780	chr4	100644768	100645019	19.00	19.90304	10.66399
2781	chr4	102268693	102269065	136.00	242.99040	73.04831
2782	chr4	103350814	103351043	15.00	14.44997	8.43918
2783	chr4	106554483	106554702	11.00	8.94684	5.91686

2784	chr4	106629859	106630063	14.00	13.29834	7.97350
2785	chr4	106631778	106632058	22.00	17.98963	8.61746
2786	chr4	106635299	106635597	28.00	20.43819	8.27744
2787	chr4	109863154	109863492	27.00	30.09214	14.11260
2788	chr4	109876253	109876457	16.00	14.76748	8.38221
2789	chr4	109998750	109999068	9.00	6.84365	4.93071
2790	chr4	110650599	110650837	18.00	18.23309	9.94449
2791	chr4	110910178	110910407	12.00	7.57257	4.87074
2792	chr4	111544187	111544560	134.00	230.93491	67.54291
2793	chr4	111590895	111591075	15.00	12.67947	7.35640
2794	chr4	119910609	119910840	21.00	18.93321	9.47521
2795	chr4	123653560	123653974	144.00	177.43980	33.42554
2796	chr4	123699109	123699284	11.00	9.69164	6.37880
2797	chr4	123795477	123795753	30.00	19.08895	7.14615
2798	chr4	124317712	124318068	112.00	191.25208	60.25153
2799	chr4	124318456	124318802	107.00	132.15613	30.82632
2800	chr4	124509368	124509602	15.00	14.60207	8.53119
2801	chr4	126235843	126236133	33.00	40.80316	18.12878
2802	chr4	128983434	128983651	17.00	17.20576	9.59759
2803	chr4	129308089	129308400	27.00	26.64490	12.05936
2804	chr4	129314491	129314700	14.00	13.29834	7.97350
2805	chr4	129474591	129474831	12.00	10.86272	6.91037
2806	chr4	129475122	129475348	20.00	21.22644	11.16290
2807	chr4	137241354	137241529	12.00	8.75023	5.59899
2808	chr4	139163417	139163640	31.00	17.32114	6.18656
2809	chr4	140004036	140004440	136.00	204.94313	51.33009
2810	chr4	140477544	140477804	23.00	25.54366	12.79678
2811	chr4	140586889	140587064	9.00	7.32469	5.23394
2812	chr4	141281070	141281283	14.00	12.35372	7.39607
2813	chr4	141648007	141648207	18.00	18.54332	10.13079
2814	chr4	141662076	141662368	28.00	25.19533	10.86549
2815	chr4	141686057	141686288	17.00	17.20576	9.59759
2816	chr4	142228444	142228634	21.00	16.84896	8.24279
2817	chr4	142774594	142774825	24.00	22.71256	10.76729
2818	chr4	142820751	142820955	10.00	8.55496	5.84723
2819	chr4	143017203	143017548	20.00	18.72424	9.65528
2820	chr4	143738661	143738868	12.00	10.89743	6.93159
2821	chr4	144109566	144109752	18.00	13.54559	7.11877
2822	chr4	146102117	146102472	23.00	19.14867	8.99213
2823	chr4	149363623	149364028	205.00	401.02475	109.83907
2824	chr4	153891638	153891913	38.00	23.87852	7.53988
2825	chr4	154155151	154155341	16.00	13.84165	7.81618
2826	chr4	159131394	159131620	15.00	14.60207	8.53119
2827	chr4	159221253	159221502	18.00	17.28003	9.36836
2828	chr4	159968028	159968299	30.00	32.23818	14.25303
2829	chr4	160024049	160024352	91.00	145.59456	47.78492
2830	chr4	160219624	160219799	12.00	10.89743	6.93159
2831	chr4	163085030	163085373	102.00	163.19814	50.78635

2832	chr4	163086108	163086382	17.00	17.20576	9.59759
2833	chr4	166248579	166248865	36.00	35.61321	13.86287
2834	chr4	166427146	166427415	43.00	37.95094	12.55887
2835	chr4	166888667	166888917	19.00	19.90304	10.66399
2836	chr4	166892942	166893257	25.00	21.51895	9.74148
2837	chr4	169564688	169564955	24.00	25.02511	12.15080
2838	chr4	169577433	169577735	56.00	64.63990	21.35632
2839	chr4	169991970	169992265	41.00	48.92794	19.44185
2840	chr4	170541345	170541608	34.00	32.93251	13.11353
2841	chr4	174173596	174173819	17.00	12.48723	6.74410
2842	chr4	175204192	175204609	149.00	266.19089	76.73938
2843	chr4	183896948	183897180	14.00	13.33873	7.99799
2844	chr4	184329920	184330244	53.00	60.80211	20.64542
2845	chr4	184509408	184509616	15.00	10.44156	5.99476
2846	chr4	184531331	184531561	21.00	21.75046	11.17117
2847	chr4	184580226	184580591	194.00	367.75491	99.76119
2848	chr4	184797681	184797939	11.00	9.72353	6.39839
2849	chr4	185394172	185394352	18.00	18.10711	9.86863
2850	chr4	186130503	186130825	42.00	54.62375	22.33426
2851	chr4	186835807	186836059	12.00	9.62240	6.14301
2852	chr4	187546087	187546351	18.00	13.54559	7.11877
2853	chr4	187686841	187687135	19.00	17.47089	9.19550
2854	chr4	189031283	189031500	16.00	15.84551	9.03663
2855	chr5	82240	82508	37.00	36.97105	14.23754
2856	chr5	891397	891624	22.00	14.28555	6.56486
2857	chr5	912478	912653	17.00	9.65886	5.13772
2858	chr5	1176841	1177206	125.00	186.31485	48.44468
2859	chr5	5351678	5351853	17.00	9.29016	4.94004
2860	chr5	5422279	5422650	126.00	136.48633	24.55293
2861	chr5	9348787	9349193	36.00	31.37751	11.55138
2862	chr5	10316043	10316287	34.00	23.09975	8.06824
2863	chr5	10441349	10441538	35.00	25.26263	8.81421
2864	chr5	10485227	10485588	103.00	125.64062	29.68460
2865	chr5	10504225	10504606	20.00	10.09393	4.84094
2866	chr5	10507650	10507996	97.00	122.71574	31.31347
2867	chr5	14145303	14145686	154.00	212.98561	44.24147
2868	chr5	14264803	14265136	105.00	91.32027	15.49368
2869	chr5	14580375	14581085	80.00	105.53518	31.49875
2870	chr5	14581653	14582016	134.00	204.60371	52.49792
2871	chr5	14666549	14666758	23.00	14.75946	6.58672
2872	chr5	14908425	14908600	16.00	9.44969	5.21180
2873	chr5	16465806	16466141	88.00	82.79391	17.20638
2874	chr5	16502987	16503199	32.00	18.22108	6.37989
2875	chr5	16550482	16550666	19.00	11.44765	5.70858
2876	chr5	16964921	16965270	69.00	73.25175	19.98002
2877	chr5	17070136	17070478	68.00	84.28503	26.23296
2878	chr5	17326806	17326993	20.00	10.09393	4.84094
2879	chr5	27241871	27242134	30.00	24.20696	9.67819

2880	chr5	29264324	29264545	18.00	14.51812	7.69630
2881	chr5	31532117	31532487	200.00	298.17386	57.37120
2882	chr5	31639032	31639295	35.00	24.13375	8.29876
2883	chr5	32313117	32313549	233.00	395.85840	83.81575
2884	chr5	33442566	33442808	29.00	23.22017	9.45267
2885	chr5	34598383	34598699	37.00	28.96195	10.02488
2886	chr5	34684525	34684719	27.00	16.22333	6.45459
2887	chr5	34691682	34691894	25.00	18.60677	8.11719
2888	chr5	34929209	34929480	37.00	30.63221	10.84630
2889	chr5	35858032	35858389	31.00	23.71973	9.13372
2890	chr5	35877097	35877371	33.00	28.69264	11.12490
2891	chr5	35938701	35938992	37.00	26.23695	8.75980
2892	chr5	36151191	36151458	24.00	16.27042	7.13572
2893	chr5	36450772	36451001	18.00	10.56158	5.43350
2894	chr5	36725158	36725426	43.00	34.21035	10.77293
2895	chr5	37249847	37250108	31.00	17.32114	6.18656
2896	chr5	37272668	37272894	26.00	18.32292	7.70658
2897	chr5	37812532	37812802	43.00	41.52236	14.39693
2898	chr5	38445383	38445783	153.00	147.39465	20.06253
2899	chr5	38634900	38635219	36.00	34.03033	12.97978
2900	chr5	38808370	38808547	20.00	13.04234	6.35230
2901	chr5	39092623	39092906	24.00	15.73713	6.86117
2902	chr5	39102511	39102830	58.00	50.68118	13.60074
2903	chr5	39428873	39429048	19.00	13.49598	6.83989
2904	chr5	40676847	40677038	23.00	17.13496	7.85287
2905	chr5	40678187	40678367	22.00	15.14085	7.01965
2906	chr5	41904259	41904445	19.00	11.44765	5.70858
2907	chr5	43313634	43314046	97.00	115.99284	27.97203
2908	chr5	43432542	43432907	87.00	89.63459	20.28585
2909	chr5	43602860	43603164	68.00	71.80498	19.69459
2910	chr5	52079248	52079489	28.00	32.98889	15.46278
2911	chr5	55008147	55008322	12.00	10.89743	6.93159
2912	chr5	55290831	55291143	50.00	69.50916	27.19317
2913	chr5	55419833	55420031	16.00	15.84551	9.03663
2914	chr5	55839968	55840166	11.00	8.94684	5.91686
2915	chr5	55933321	55933564	17.00	17.15686	9.56820
2916	chr5	56790710	56790891	14.00	12.35372	7.39607
2917	chr5	57487280	57487520	11.00	9.69164	6.37880
2918	chr5	58944754	58944994	15.00	14.60207	8.53119
2919	chr5	59041225	59041580	22.00	24.04191	12.22603
2920	chr5	59087558	59087739	12.00	10.77529	6.85683
2921	chr5	59995376	59995600	15.00	14.60207	8.53119
2922	chr5	60704542	60704879	37.00	47.28369	20.26158
2923	chr5	65891994	65892309	98.00	162.06445	52.78674
2924	chr5	71893205	71893380	19.00	14.62602	7.49344
2925	chr5	75997170	75997345	11.00	9.72353	6.39839
2926	chr5	77934210	77934445	13.00	10.43506	6.43685
2927	chr5	78075619	78075975	60.00	83.41049	30.07736

2928	chr5	78081411	78081586	12.00	10.89743	6.93159
2929	chr5	81046820	81047182	51.00	71.28306	27.72637
2930	chr5	81147417	81147613	18.00	13.54559	7.11877
2931	chr5	81591039	81591300	22.00	24.10503	12.26359
2932	chr5	82373157	82373395	19.00	14.62602	7.49344
2933	chr5	86205233	86205423	14.00	13.33873	7.99799
2934	chr5	89705534	89705793	19.00	19.90304	10.66399
2935	chr5	90278977	90279249	21.00	22.68483	11.73039
2936	chr5	90447832	90448083	15.00	14.60207	8.53119
2937	chr5	90675767	90676071	76.00	117.98460	41.05635
2938	chr5	92695492	92695835	66.00	94.19536	33.03579
2939	chr5	92936751	92936926	10.00	8.40994	5.75733
2940	chr5	93805773	93805979	19.00	9.29158	4.61042
2941	chr5	93807309	93807650	38.00	48.93276	20.79478
2942	chr5	94033863	94034297	67.00	100.69668	36.25756
2943	chr5	94082473	94082722	15.00	14.13432	8.24751
2944	chr5	94466688	94466863	16.00	13.03207	7.32175
2945	chr5	94890351	94890643	43.00	54.60016	21.85340
2946	chr5	95744278	95744489	16.00	13.03207	7.32175
2947	chr5	95828287	95828641	26.00	22.72887	10.11615
2948	chr5	96143959	96144365	208.00	408.15222	111.43867
2949	chr5	98109052	98109459	107.00	179.66258	56.96445
2950	chr5	98265238	98265579	49.00	67.74368	26.65997
2951	chr5	106878190	106878455	26.00	22.12900	9.77388
2952	chr5	108084488	108084763	46.00	51.99404	18.92561
2953	chr5	108745652	108746019	75.00	102.63219	32.73255
2954	chr5	109024482	109024681	25.00	17.28854	7.42115
2955	chr5	110702887	110703100	17.00	13.40451	7.29123
2956	chr5	111564360	111564644	13.00	6.36973	3.99600
2957	chr5	111674870	111675058	20.00	15.41028	7.68361
2958	chr5	112196706	112197085	243.00	351.86127	56.24712
2959	chr5	112294481	112294727	24.00	18.22131	8.18007
2960	chr5	118660152	118660413	36.00	33.55295	12.71768
2961	chr5	118664579	118664754	15.00	10.82008	6.22198
2962	chr5	121668687	121669049	26.00	22.72887	10.11615
2963	chr5	121829024	121829370	28.00	20.43819	8.27744
2964	chr5	121835397	121835572	19.00	9.29158	4.61042
2965	chr5	122091394	122091677	14.00	9.64725	5.73484
2966	chr5	123149634	123150056	19.00	12.97326	6.54406
2967	chr5	123155457	123155683	18.00	13.54559	7.11877
2968	chr5	123998007	123998350	75.00	82.05872	21.69259
2969	chr5	124070876	124071064	18.00	14.99246	7.98119
2970	chr5	124511806	124511984	17.00	15.21434	8.38926
2971	chr5	125703938	125704199	36.00	25.17959	8.52928
2972	chr5	125758759	125759190	81.00	91.07008	23.40517
2973	chr5	126113900	126114187	41.00	38.87061	13.74252
2974	chr5	126366179	126366539	119.00	191.57719	55.54813
2975	chr5	126853943	126854279	43.00	45.94378	16.82220

2976	chr5	127170063	127170244	15.00	10.44156	5.99476
2977	chr5	127211254	127211501	21.00	10.91615	5.07146
2978	chr5	131601318	131601637	48.00	38.55299	11.29553
2979	chr5	132299186	132299438	23.00	19.14867	8.99213
2980	chr5	133560603	133560851	30.00	22.61202	8.84830
2981	chr5	133841887	133842173	41.00	43.08278	16.05755
2982	chr5	133890010	133890310	16.00	14.76748	8.38221
2983	chr5	134360904	134361269	68.00	90.43758	29.71771
2984	chr5	134370445	134370638	20.00	17.24802	8.76723
2985	chr5	136646229	136646404	12.00	9.10444	5.81988
2986	chr5	136897364	136897546	19.00	15.65382	8.10137
2987	chr5	136996063	136996238	21.00	9.12806	4.25326
2988	chr5	137090233	137090597	103.00	143.59521	38.96591
2989	chr5	137114777	137115045	23.00	24.67397	12.27830
2990	chr5	137151347	137151662	32.00	18.22108	6.37989
2991	chr5	137549027	137549205	15.00	11.92080	6.89106
2992	chr5	137618846	137619070	17.00	16.01209	8.87529
2993	chr5	137690713	137691032	48.00	44.32025	13.98602
2994	chr5	139123194	139123382	17.00	13.92968	7.60807
2995	chr5	139726345	139726622	45.00	51.07658	18.85752
2996	chr5	139780504	139781527	280.00	505.88535	105.28288
2997	chr5	139927071	139927322	28.00	26.72140	11.74698
2998	chr5	140700193	140700590	186.00	306.89639	71.49434
2999	chr5	141075299	141075513	17.00	14.00817	7.65559
3000	chr5	141093600	141093778	19.00	11.44765	5.70858
3001	chr5	142812407	142812717	21.00	17.13758	8.41110
3002	chr5	142874794	142875017	20.00	10.09393	4.84094
3003	chr5	143016673	143016909	18.00	13.54559	7.11877
3004	chr5	143301086	143301309	26.00	18.32292	7.70658
3005	chr5	145316284	145316471	11.00	6.67667	4.49607
3006	chr5	145582521	145583318	271.00	551.58441	139.15407
3007	chr5	145826447	145827009	191.00	343.61230	88.27684
3008	chr5	145879308	145879535	18.00	17.50714	9.50604
3009	chr5	146098266	146098671	104.00	161.18639	48.27640
3010	chr5	146102084	146102385	45.00	48.21673	17.23492
3011	chr5	147391355	147391555	17.00	15.60178	8.62543
3012	chr5	147470236	147470626	36.00	35.61321	13.86287
3013	chr5	148188431	148188715	39.00	28.38507	9.22084
3014	chr5	148292663	148292966	10.00	5.81477	4.12139
3015	chr5	148293691	148293882	19.00	18.45299	9.79053
3016	chr5	148345413	148345643	27.00	25.42518	11.34191
3017	chr5	148737256	148737663	83.00	108.55029	31.47246
3018	chr5	148866380	148866597	15.00	12.76090	7.40642
3019	chr5	149317338	149317559	16.00	11.08796	6.15393
3020	chr5	149317962	149318172	21.00	13.32019	6.27944
3021	chr5	149340061	149340494	230.00	434.11203	107.66212
3022	chr5	149894396	149894571	14.00	9.45699	5.62008
3023	chr5	150010379	150010616	19.00	14.62602	7.49344

3024	chr5	150138016	150138498	211.00	356.23590	79.43050
3025	chr5	150460465	150460825	123.00	187.11015	50.22846
3026	chr5	150827433	150827694	36.00	37.58275	14.98752
3027	chr5	151150827	151151594	262.00	511.76215	124.27778
3028	chr5	153825224	153825632	184.00	335.47986	89.27859
3029	chr5	156476618	156476883	29.00	26.45082	11.24017
3030	chr5	156569728	156570163	199.00	374.17722	99.33364
3031	chr5	156570693	156571019	35.00	41.79023	17.88005
3032	chr5	158768959	158769248	21.00	18.07733	8.96484
3033	chr5	159008988	159009286	33.00	16.79232	5.66006
3034	chr5	159139620	159139949	66.00	70.70631	20.00029
3035	chr5	159326351	159326584	26.00	18.32292	7.70658
3036	chr5	159333930	159334154	18.00	13.54559	7.11877
3037	chr5	159363093	159363273	13.00	8.38884	5.17806
3038	chr5	159546411	159546999	233.00	426.57062	100.78179
3039	chr5	159780346	159780609	24.00	23.93016	11.49438
3040	chr5	162864248	162864710	187.00	306.32986	70.43837
3041	chr5	167372606	167372912	47.00	53.70549	19.44328
3042	chr5	167532250	167532504	21.00	16.84896	8.24279
3043	chr5	168039241	168039417	14.00	9.45699	5.62008
3044	chr5	169095337	169095537	12.00	10.28098	6.55228
3045	chr5	170737281	170737456	14.00	11.24935	6.71524
3046	chr5	171431993	171432179	16.00	13.18599	7.41557
3047	chr5	171529995	171530269	21.00	20.46342	10.39586
3048	chr5	171847507	171847933	176.00	283.64713	66.18454
3049	chr5	172329366	172329558	20.00	12.37394	5.99401
3050	chr5	172410565	172410801	21.00	19.55588	9.84902
3051	chr5	172483979	172484355	206.00	358.94608	84.85883
3052	chr5	172662174	172662387	23.00	17.84635	8.24931
3053	chr5	173193425	173193723	35.00	37.27223	15.21614
3054	chr5	173285881	173286061	15.00	10.44156	5.99476
3055	chr5	176057453	176057732	26.00	22.72887	10.11615
3056	chr5	176778694	176779099	213.00	323.12698	61.08178
3057	chr5	176853152	176853537	129.00	191.61064	48.70739
3058	chr5	176884618	176884871	28.00	30.03004	13.70363
3059	chr5	177631264	177631638	200.00	371.58078	96.99998
3060	chr5	177715959	177716244	15.00	10.44156	5.99476
3061	chr5	178214061	178214376	27.00	31.39094	14.88386
3062	chr5	179125661	179126008	165.00	296.40009	81.84985
3063	chr5	179159408	179159946	193.00	359.23260	95.65585
3064	chr5	179718988	179719257	43.00	52.55379	20.64841
3065	chr5	179780753	179780964	15.00	14.55884	8.50506
3066	chr5	179920515	179920781	24.00	23.42492	11.19207
3067	chr6	1079723	1080006	26.00	22.72887	10.11615
3068	chr6	1111259	1111485	14.00	11.54338	6.89663
3069	chr6	1397202	1397441	19.00	18.93242	10.08043
3070	chr6	2987096	2987274	11.00	9.72353	6.39839
3071	chr6	3612813	3613109	19.00	19.90304	10.66399

3072	chr6	4180993	4181207	17.00	12.48723	6.74410
3073	chr6	6685598	6685926	86.00	98.72491	24.83231
3074	chr6	6694607	6694782	20.00	12.37394	5.99401
3075	chr6	6712859	6713102	27.00	26.64490	12.05936
3076	chr6	6882106	6882344	20.00	15.26912	7.60191
3077	chr6	7141251	7141461	25.00	26.11938	12.45895
3078	chr6	7267873	7268064	17.00	12.48723	6.74410
3079	chr6	7480245	7480442	23.00	19.14867	8.99213
3080	chr6	7540818	7541020	20.00	16.00201	8.02878
3081	chr6	7698630	7698842	10.00	7.81468	5.38479
3082	chr6	7726087	7726418	81.00	105.15163	30.72312
3083	chr6	7728788	7728964	12.00	10.05135	6.40993
3084	chr6	7729181	7729424	21.00	21.47741	11.00699
3085	chr6	8000340	8000616	21.00	16.84896	8.24279
3086	chr6	9516457	9516724	19.00	14.62602	7.49344
3087	chr6	10441457	10441769	31.00	29.00390	11.98951
3088	chr6	10694779	10695055	20.00	21.28387	11.19719
3089	chr6	11230951	11231180	23.00	20.39780	9.72164
3090	chr6	11435185	11435447	18.00	16.84567	9.10462
3091	chr6	11766944	11767164	13.00	11.43624	7.05630
3092	chr6	11805505	11805706	11.00	9.72353	6.39839
3093	chr6	13913326	13913555	17.00	16.01209	8.87529
3094	chr6	14271283	14271518	21.00	13.32019	6.27944
3095	chr6	15245491	15245862	135.00	235.71503	69.57703
3096	chr6	15949837	15950102	23.00	19.14867	8.99213
3097	chr6	16277624	16277881	25.00	28.47325	13.86318
3098	chr6	16757044	16757257	14.00	13.33873	7.99799
3099	chr6	17393718	17393977	33.00	40.80316	18.12878
3100	chr6	17865540	17865753	17.00	16.01209	8.87529
3101	chr6	18387431	18387793	74.00	113.89050	39.86749
3102	chr6	20280012	20280226	12.00	10.89743	6.93159
3103	chr6	25174215	25174457	20.00	19.39882	10.06300
3104	chr6	25263902	25264162	19.00	19.90304	10.66399
3105	chr6	26016641	26016947	45.00	40.47089	13.12973
3106	chr6	26055605	26055841	19.00	19.90304	10.66399
3107	chr6	26195555	26195866	52.00	71.81245	27.52827
3108	chr6	26597057	26597317	32.00	24.84073	9.41915
3109	chr6	27219533	27219914	77.00	119.93452	41.58955
3110	chr6	27342988	27343237	20.00	21.28387	11.19719
3111	chr6	27585665	27585977	54.00	76.49955	29.23616
3112	chr6	33567246	33567457	12.00	10.89743	6.93159
3113	chr6	33636840	33637143	22.00	24.10503	12.26359
3114	chr6	33894245	33894509	23.00	25.54366	12.79678
3115	chr6	34204542	34204735	18.00	16.43544	8.85533
3116	chr6	35300008	35300221	15.00	14.60207	8.53119
3117	chr6	35656625	35657011	91.00	147.78462	49.05434
3118	chr6	35722733	35722926	24.00	20.32534	9.36681
3119	chr6	36164911	36165298	133.00	236.41647	71.44872

3120	chr6	36679498	36679790	20.00	21.28387	11.19719
3121	chr6	37190513	37190810	33.00	31.61030	12.73885
3122	chr6	37210320	37210564	11.00	8.94684	5.91686
3123	chr6	40132255	40132495	23.00	15.26920	6.85029
3124	chr6	41286605	41286904	20.00	21.22644	11.16290
3125	chr6	41902937	41903136	15.00	13.36375	7.77651
3126	chr6	42018518	42018775	14.00	11.54338	6.89663
3127	chr6	42981551	42981906	107.00	150.81496	40.46460
3128	chr6	43027109	43027423	38.00	38.34014	14.61222
3129	chr6	43142347	43142665	29.00	28.03230	12.15205
3130	chr6	43655560	43655738	21.00	13.32019	6.27944
3131	chr6	43738314	43738490	12.00	10.60494	6.75222
3132	chr6	44213822	44214159	59.00	82.75965	30.24129
3133	chr6	45413515	45413837	23.00	24.52095	12.18673
3134	chr6	46620466	46620658	21.00	21.21263	10.84757
3135	chr6	46724315	46724554	20.00	21.22644	11.16290
3136	chr6	47002854	47003053	19.00	17.47089	9.19550
3137	chr6	49508943	49509199	32.00	39.21360	17.59558
3138	chr6	49524169	49524512	64.00	95.04503	34.65796
3139	chr6	49754956	49755194	12.00	10.86272	6.91037
3140	chr6	52149587	52149959	93.00	144.77391	46.01547
3141	chr6	52226617	52226805	21.00	10.91615	5.07146
3142	chr6	52369087	52369373	33.00	34.90736	14.64351
3143	chr6	52528952	52529308	166.00	297.58835	81.75089
3144	chr6	53918749	53918968	20.00	19.88133	10.35450
3145	chr6	56246757	56247037	19.00	14.62602	7.49344
3146	chr6	64197129	64197452	20.00	21.22644	11.16290
3147	chr6	64271128	64271365	17.00	13.40451	7.29123
3148	chr6	64276073	64276338	43.00	32.80746	10.14292
3149	chr6	72113913	72114173	22.00	24.04191	12.22603
3150	chr6	72114727	72114915	20.00	15.72746	7.86812
3151	chr6	72130564	72130755	14.00	12.71320	7.61671
3152	chr6	73497459	73497641	12.00	10.05135	6.40993
3153	chr6	74390017	74390321	12.00	10.89743	6.93159
3154	chr6	75459587	75459898	47.00	60.95343	23.66743
3155	chr6	79713990	79714267	18.00	18.10711	9.86863
3156	chr6	79943521	79943867	64.00	75.67220	23.52972
3157	chr6	80173373	80173902	39.00	50.59282	21.32797
3158	chr6	82707493	82707699	13.00	12.06567	7.44193
3159	chr6	82957407	82957695	32.00	24.84073	9.41915
3160	chr6	84174598	84174909	50.00	36.32626	9.85984
3161	chr6	84903250	84903436	14.00	13.33873	7.99799
3162	chr6	85361867	85362185	46.00	60.15939	23.68901
3163	chr6	85592837	85593114	22.00	24.04191	12.22603
3164	chr6	85776114	85776404	34.00	32.93251	13.11353
3165	chr6	85823903	85824421	30.00	29.35740	12.55712
3166	chr6	86070871	86071055	20.00	15.72746	7.86812
3167	chr6	86168693	86168895	18.00	14.51812	7.69630

3168	chr6	86182368	86182602	22.00	21.29036	10.57483
3169	chr6	86303675	86303925	20.00	20.52682	10.74351
3170	chr6	88410511	88410695	17.00	12.48723	6.74410
3171	chr6	89745436	89745789	25.00	25.27586	11.95416
3172	chr6	89856543	89856840	14.00	7.15249	4.28143
3173	chr6	90007729	90007968	35.00	34.26693	13.48820
3174	chr6	90529462	90529710	26.00	29.88839	14.35230
3175	chr6	90928309	90928508	23.00	15.26920	6.85029
3176	chr6	91762797	91762974	16.00	15.84551	9.03663
3177	chr6	96969571	96969746	11.00	9.72353	6.39839
3178	chr6	97842225	97842400	15.00	14.55884	8.50506
3179	chr6	99282175	99282437	10.00	8.58401	5.86519
3180	chr6	100676714	100677034	62.00	91.13325	33.48869
3181	chr6	101528550	101528807	15.00	10.44156	5.99476
3182	chr6	106040294	106040508	14.00	11.24935	6.71524
3183	chr6	107349365	107349583	12.00	10.86272	6.91037
3184	chr6	108879606	108880001	152.00	278.51715	81.57951
3185	chr6	109058109	109058434	46.00	56.62083	21.60944
3186	chr6	109330680	109331122	157.00	289.32397	83.98752
3187	chr6	110114971	110115316	27.00	31.46828	14.92958
3188	chr6	110217631	110217806	10.00	8.58401	5.86519
3189	chr6	110725832	110726097	16.00	11.45212	6.36943
3190	chr6	111137024	111137266	19.00	14.88650	7.64645
3191	chr6	111195718	111196062	171.00	299.79166	79.08134
3192	chr6	112133914	112134145	12.00	10.44029	6.65075
3193	chr6	112194524	112194973	153.00	211.19379	43.95605
3194	chr6	112340298	112340578	14.00	11.54338	6.89663
3195	chr6	112525330	112525539	16.00	14.86771	8.44336
3196	chr6	112552831	112553060	15.00	13.54753	7.88914
3197	chr6	113754743	113754918	14.00	10.20871	6.07602
3198	chr6	117002676	117002856	18.00	18.54332	10.13079
3199	chr6	117761852	117762199	76.00	110.84270	36.89767
3200	chr6	117923672	117923873	18.00	11.47393	5.93179
3201	chr6	119670839	119671090	28.00	32.98889	15.46278
3202	chr6	122720549	122720744	13.00	12.10322	7.46479
3203	chr6	126071410	126071770	19.00	19.90304	10.66399
3204	chr6	126143253	126143428	15.00	11.92080	6.89106
3205	chr6	127687476	127687651	15.00	11.24842	6.48109
3206	chr6	129046947	129047125	15.00	12.84344	7.45712
3207	chr6	129639502	129639704	10.00	5.81477	4.12139
3208	chr6	132275423	132275646	21.00	21.75046	11.17117
3209	chr6	132275925	132276125	16.00	13.84165	7.81618
3210	chr6	132834142	132834583	179.00	339.56732	95.68198
3211	chr6	133361197	133361420	15.00	14.23777	8.31042
3212	chr6	133911361	133911693	46.00	57.12513	21.90528
3213	chr6	133930735	133930957	18.00	16.84567	9.10462
3214	chr6	134609951	134610212	18.00	18.49157	10.09976
3215	chr6	134614784	134615024	12.00	7.57257	4.87074

3216	chr6	134789737	134789979	19.00	17.47089	9.19550
3217	chr6	134793149	134793324	10.00	8.55496	5.84723
3218	chr6	136571392	136571720	47.00	43.02813	13.70059
3219	chr6	136724325	136724563	18.00	13.54559	7.11877
3220	chr6	138050254	138050502	28.00	27.98681	12.49005
3221	chr6	138188758	138189024	16.00	11.45212	6.36943
3222	chr6	139094664	139094905	18.00	18.49157	10.09976
3223	chr6	139728954	139729158	14.00	12.99838	7.79102
3224	chr6	139977364	139977570	17.00	16.79353	9.34922
3225	chr6	140681644	140681826	12.00	10.89743	6.93159
3226	chr6	140784409	140784621	15.00	14.34297	8.37430
3227	chr6	142330877	142331061	18.00	13.54559	7.11877
3228	chr6	142697203	142697507	16.00	15.89157	9.06439
3229	chr6	143206509	143206822	48.00	62.63177	24.16050
3230	chr6	143381268	143381565	13.00	12.10322	7.46479
3231	chr6	143994772	143995028	16.00	8.79855	4.85229
3232	chr6	144600717	144600974	25.00	22.87882	10.53177
3233	chr6	144605932	144606191	18.00	18.54332	10.13079
3234	chr6	144608206	144608471	24.00	25.93308	12.69451
3235	chr6	145653203	145653395	16.00	15.89157	9.06439
3236	chr6	147312302	147312543	10.00	8.58401	5.86519
3237	chr6	147523518	147523864	87.00	124.17191	37.90084
3238	chr6	149397330	149397510	20.00	14.59977	7.21814
3239	chr6	149657251	149657463	13.00	8.50004	5.24541
3240	chr6	149775409	149775585	10.00	8.13450	5.58558
3241	chr6	150071247	150071564	20.00	20.93955	10.99128
3242	chr6	150210436	150210712	17.00	13.40451	7.29123
3243	chr6	150284901	150285106	13.00	12.10322	7.46479
3244	chr6	150657451	150657626	12.00	10.89743	6.93159
3245	chr6	150739718	150740037	44.00	58.92307	23.92050
3246	chr6	150903279	150903600	17.00	17.20576	9.59759
3247	chr6	151390090	151390266	16.00	8.79855	4.85229
3248	chr6	151403147	151403440	19.00	14.62602	7.49344
3249	chr6	152085012	152085414	141.00	238.33131	66.63806
3250	chr6	152086670	152086950	29.00	31.29580	14.07846
3251	chr6	154731771	154731994	22.00	19.18340	9.31657
3252	chr6	155055238	155055553	20.00	15.72746	7.86812
3253	chr6	155522842	155523134	18.00	17.28003	9.36836
3254	chr6	157153299	157153602	51.00	62.16832	22.39595
3255	chr6	157274777	157274952	12.00	10.86272	6.91037
3256	chr6	157427689	157427887	22.00	18.29233	8.79342
3257	chr6	159274625	159274802	15.00	13.27388	7.72139
3258	chr6	159527723	159528117	91.00	138.70396	43.77742
3259	chr6	160211243	160211640	163.00	257.75443	61.44624
3260	chr6	160675754	160676015	27.00	29.57795	13.80600
3261	chr6	162479342	162479522	17.00	12.48723	6.74410
3262	chr6	165987135	165987310	10.00	8.58401	5.86519
3263	chr6	166397214	166397476	32.00	30.30064	12.36418

3264	chr6	166419652	166419920	41.00	46.61858	18.08904
3265	chr6	169612993	169613236	31.00	37.63698	17.06238
3266	chr6	170747603	170747877	43.00	42.87618	15.12373
3267	chr6	170862952	170863223	28.00	29.40866	13.33348
3268	chr7	475153	475468	39.00	47.86564	19.72286
3269	chr7	554539	554754	18.00	14.51812	7.69630
3270	chr7	916445	916693	16.00	13.42407	7.56089
3271	chr7	1177785	1178201	208.00	400.32587	106.92354
3272	chr7	1714925	1715167	13.00	12.10322	7.46479
3273	chr7	1782479	1782675	12.00	10.05135	6.40993
3274	chr7	1808470	1808822	54.00	61.58029	20.60697
3275	chr7	2883936	2884358	70.00	86.84992	26.60172
3276	chr7	4652810	4653093	36.00	29.45019	10.56087
3277	chr7	4815064	4815364	66.00	94.19536	33.03579
3278	chr7	5463545	5463801	76.00	108.27338	35.40269
3279	chr7	5465865	5466165	33.00	30.84510	12.30785
3280	chr7	5469609	5469840	21.00	16.84896	8.24279
3281	chr7	5570192	5570472	41.00	42.51218	15.73623
3282	chr7	5594074	5594278	13.00	11.10738	6.85337
3283	chr7	5609724	5609960	16.00	15.89157	9.06439
3284	chr7	7139204	7139379	16.00	11.08796	6.15393
3285	chr7	8008098	8008492	136.00	226.35478	63.41745
3286	chr7	11012585	11012979	152.00	259.09494	70.34561
3287	chr7	12202125	12202578	12.00	10.89743	6.93159
3288	chr7	12726936	12727305	145.00	252.75609	71.98843
3289	chr7	17639453	17639719	30.00	27.72049	11.61484
3290	chr7	20392420	20392677	19.00	14.88650	7.64645
3291	chr7	20447528	20447945	41.00	30.57590	9.68188
3292	chr7	20554072	20554303	16.00	11.45212	6.36943
3293	chr7	21467554	21467816	35.00	38.10524	15.70356
3294	chr7	22616745	22617072	20.00	15.72746	7.86812
3295	chr7	22893422	22893597	13.00	11.69699	7.21657
3296	chr7	23053543	23054078	169.00	279.11526	68.86160
3297	chr7	23312889	23313147	33.00	31.61030	12.73885
3298	chr7	23509981	23510333	55.00	76.38741	28.64937
3299	chr7	24241849	24242043	17.00	16.11854	8.94003
3300	chr7	24330023	24330198	14.00	10.83560	6.46037
3301	chr7	24612534	24612736	13.00	12.10322	7.46479
3302	chr7	24613613	24613854	18.00	16.23862	8.73573
3303	chr7	24781289	24781468	20.00	14.59977	7.21814
3304	chr7	24860600	24860775	14.00	9.45699	5.62008
3305	chr7	24861135	24861339	17.00	15.80392	8.74857
3306	chr7	25019729	25020108	121.00	194.30525	55.71634
3307	chr7	25792735	25793244	27.00	30.44836	14.32468
3308	chr7	25886294	25886469	15.00	10.64585	6.11716
3309	chr7	25894677	25895083	189.00	329.53247	81.83138
3310	chr7	25896586	25897030	77.00	106.16860	33.59393
3311	chr7	25933938	25934204	32.00	30.30064	12.36418

3312	chr7	26525023	26525337	27.00	25.31033	11.27483
3313	chr7	26693199	26693535	102.00	143.23245	39.37923
3314	chr7	27138558	27138744	14.00	11.69641	6.99105
3315	chr7	27150288	27150665	13.00	11.35209	7.00445
3316	chr7	27153054	27153247	21.00	19.88509	10.04721
3317	chr7	27154771	27154952	14.00	10.20871	6.07602
3318	chr7	27156275	27156552	30.00	29.22960	12.48285
3319	chr7	27182189	27182479	29.00	30.20530	13.43048
3320	chr7	27183256	27183443	17.00	14.16795	7.75245
3321	chr7	27190487	27190814	52.00	65.86428	24.04337
3322	chr7	27229022	27229238	23.00	19.14867	8.99213
3323	chr7	27702381	27702842	240.00	482.88208	127.11513
3324	chr7	29662234	29662511	22.00	17.98963	8.61746
3325	chr7	30066156	30066636	215.00	367.69965	82.58170
3326	chr7	30323870	30324194	62.00	80.22781	27.13356
3327	chr7	33534858	33535102	25.00	21.86390	9.94039
3328	chr7	33762593	33762824	17.00	15.90723	8.81147
3329	chr7	33927805	33928039	30.00	22.61202	8.84830
3330	chr7	36087880	36088141	25.00	23.77130	11.05808
3331	chr7	38217539	38217906	154.00	212.98561	44.24147
3332	chr7	40938868	40939043	13.00	9.77812	6.02968
3333	chr7	41228435	41228671	29.00	26.45082	11.24017
3334	chr7	41442919	41443135	14.00	13.09665	7.85091
3335	chr7	41956187	41956425	20.00	12.37394	5.99401
3336	chr7	42030528	42030768	21.00	19.55588	9.84902
3337	chr7	42276771	42277102	70.00	96.34446	31.99586
3338	chr7	43687577	43687757	16.00	12.52106	7.01130
3339	chr7	44163126	44163375	35.00	38.10524	15.70356
3340	chr7	44646273	44646609	97.00	138.24571	39.69669
3341	chr7	44679810	44680011	14.00	10.20871	6.07602
3342	chr7	44835996	44836382	222.00	414.50113	103.22694
3343	chr7	44887715	44888174	219.00	367.97418	79.63905
3344	chr7	45927895	45928087	17.00	15.02847	8.27595
3345	chr7	46792441	46792655	20.00	19.88133	10.35450
3346	chr7	50602539	50602790	26.00	18.32292	7.70658
3347	chr7	51418141	51418319	14.00	9.45699	5.62008
3348	chr7	54422212	54422534	36.00	43.12796	18.24364
3349	chr7	54898710	54899005	12.00	10.77529	6.85683
3350	chr7	54899569	54899845	17.00	16.56128	9.20873
3351	chr7	54905802	54906141	50.00	61.30541	22.39016
3352	chr7	55432965	55433305	20.00	12.37394	5.99401
3353	chr7	56174071	56174538	163.00	257.75443	61.44624
3354	chr7	63774121	63774398	22.00	24.10503	12.26359
3355	chr7	64023416	64023752	66.00	98.80634	35.72436
3356	chr7	64034849	64035089	19.00	11.44765	5.70858
3357	chr7	64126233	64126504	51.00	71.28306	27.72637
3358	chr7	64254511	64254867	111.00	189.14120	59.71833
3359	chr7	64363370	64363595	28.00	32.90870	15.41543

3360	chr7	64467014	64467364	92.00	149.81091	49.58754
3361	chr7	64838445	64838837	103.00	167.83553	52.80917
3362	chr7	66093633	66093962	69.00	96.43652	32.62489
3363	chr7	66461408	66461667	25.00	28.47325	13.86318
3364	chr7	72298932	72299224	33.00	40.80316	18.12878
3365	chr7	72936538	72936895	131.00	197.24510	50.46659
3366	chr7	73017778	73018061	25.00	17.28854	7.42115
3367	chr7	73148992	73149255	17.00	12.48723	6.74410
3368	chr7	73242565	73242748	20.00	18.61702	9.59052
3369	chr7	73305601	73305776	13.00	11.02827	6.80445
3370	chr7	73684360	73684535	12.00	10.89743	6.93159
3371	chr7	73727065	73727328	18.00	18.10711	9.86863
3372	chr7	73892487	73892717	14.00	11.10797	6.62808
3373	chr7	74050014	74050266	24.00	20.32534	9.36681
3374	chr7	75368195	75368655	98.00	151.72240	46.78137
3375	chr7	75923943	75924297	67.00	89.04348	29.47346
3376	chr7	75944784	75944992	18.00	13.52142	7.10456
3377	chr7	76052577	76052965	151.00	253.78482	68.04778
3378	chr7	77280886	77281139	22.00	14.28555	6.56486
3379	chr7	77325458	77325830	232.00	373.01288	72.74249
3380	chr7	77427626	77428195	241.00	423.65768	92.52209
3381	chr7	77649915	77650110	21.00	20.11179	10.18382
3382	chr7	78912556	78912783	16.00	10.06962	5.56245
3383	chr7	79907183	79907445	18.00	14.75134	7.83616
3384	chr7	80253209	80253422	22.00	17.98963	8.61746
3385	chr7	80351606	80351807	18.00	13.13437	6.87792
3386	chr7	80460399	80460574	14.00	9.54176	5.67115
3387	chr7	80642472	80642698	20.00	12.37394	5.99401
3388	chr7	83097577	83097904	22.00	22.56316	11.34064
3389	chr7	84041693	84041906	23.00	22.60143	11.03461
3390	chr7	84159134	84159309	12.00	10.68938	6.80412
3391	chr7	84305228	84305453	20.00	15.26912	7.60191
3392	chr7	84654764	84654996	30.00	22.61202	8.84830
3393	chr7	86974775	86975048	30.00	27.02617	11.22187
3394	chr7	90208499	90208761	17.00	13.47705	7.33487
3395	chr7	91686680	91686876	17.00	15.12078	8.33222
3396	chr7	92381889	92382104	20.00	17.52246	8.93153
3397	chr7	92546279	92546538	44.00	39.20615	12.84430
3398	chr7	93366379	93366584	15.00	11.92080	6.89106
3399	chr7	93669542	93669774	24.00	21.73283	10.18731
3400	chr7	95063628	95063819	21.00	10.91615	5.07146
3401	chr7	95225975	95226150	9.00	5.86707	4.30691
3402	chr7	97735937	97736359	204.00	367.22049	91.17547
3403	chr7	97761248	97761605	70.00	83.74308	24.90715
3404	chr7	97763457	97763671	24.00	13.49383	5.76302
3405	chr7	97793552	97793890	33.00	29.72506	11.68652
3406	chr7	97910323	97910513	23.00	21.11144	10.14409
3407	chr7	97987157	97987376	15.00	6.30094	3.68834

3408	chr7	98189294	98189619	54.00	61.58029	20.60697
3409	chr7	99070233	99070560	48.00	62.63177	24.16050
3410	chr7	99156035	99156307	31.00	30.69624	12.96218
3411	chr7	99679252	99679525	34.00	36.33072	15.07420
3412	chr7	99698953	99699343	176.00	288.47397	68.83061
3413	chr7	99746226	99746621	149.00	185.67392	34.57815
3414	chr7	99797923	99798140	15.00	14.23777	8.31042
3415	chr7	100136380	100136686	45.00	35.07793	10.60397
3416	chr7	100183690	100184429	299.00	496.00516	85.62866
3417	chr7	100209690	100210000	52.00	43.30815	12.21761
3418	chr7	101132249	101132461	21.00	18.26016	9.07344
3419	chr7	101345621	101346092	18.00	13.54559	7.11877
3420	chr7	101465374	101465661	49.00	53.27969	18.29432
3421	chr7	101526124	101526448	28.00	23.07031	9.67486
3422	chr7	101740974	101741284	24.00	18.22131	8.18007
3423	chr7	101794211	101794627	17.00	12.48723	6.74410
3424	chr7	101929442	101929741	15.00	14.34297	8.37430
3425	chr7	102033916	102034255	97.00	132.89111	36.71788
3426	chr7	102789337	102789565	27.00	16.22333	6.45459
3427	chr7	105070332	105070508	15.00	10.64585	6.11716
3428	chr7	105470891	105471241	22.00	13.32011	6.06769
3429	chr7	105705413	105705676	38.00	32.83260	11.64196
3430	chr7	105712575	105712786	28.00	19.81061	7.95895
3431	chr7	106175971	106176293	8.00	6.42036	4.79879
3432	chr7	106209135	106209490	89.00	120.11595	34.40904
3433	chr7	106358059	106358379	43.00	45.34496	16.48558
3434	chr7	106809023	106809556	265.00	426.17737	75.92408
3435	chr7	107204672	107204997	60.00	53.19851	14.06178
3436	chr7	107589621	107589876	15.00	5.08643	3.09328
3437	chr7	107593332	107593520	19.00	13.99491	7.12625
3438	chr7	107687590	107687783	12.00	7.73484	4.97019
3439	chr7	108166535	108167268	68.00	80.12167	23.95865
3440	chr7	110753745	110753961	17.00	9.65886	5.13772
3441	chr7	111180816	111181006	15.00	10.44156	5.99476
3442	chr7	112579795	112580113	47.00	43.02813	13.70059
3443	chr7	113882934	113883263	53.00	57.00955	18.56094
3444	chr7	114170311	114170563	26.00	25.31819	11.62867
3445	chr7	115858117	115858333	18.00	7.00940	3.67327
3446	chr7	116273479	116273768	39.00	38.81320	14.47983
3447	chr7	116442443	116442676	22.00	11.75739	5.30198
3448	chr7	116442855	116443031	18.00	12.53419	6.53070
3449	chr7	116445310	116445532	17.00	9.65886	5.13772
3450	chr7	116450399	116450602	16.00	9.62007	5.30739
3451	chr7	116502732	116502978	24.00	23.93016	11.49438
3452	chr7	117024861	117025072	18.00	16.23862	8.73573
3453	chr7	117052395	117053060	38.00	38.34014	14.61222
3454	chr7	117636298	117636473	13.00	10.43506	6.43685
3455	chr7	120591045	120591382	82.00	92.59078	23.69060

3456	chr7	123178380	123178572	14.00	12.62120	7.56032
3457	chr7	123283899	123284164	41.00	36.55940	12.53749
3458	chr7	123438475	123438820	62.00	73.99586	23.60435
3459	chr7	124099341	124099573	24.00	20.32534	9.36681
3460	chr7	124206924	124207207	22.00	14.28555	6.56486
3461	chr7	124341886	124342282	67.00	91.06698	30.64392
3462	chr7	128001828	128002088	22.00	17.98963	8.61746
3463	chr7	128379227	128379521	22.00	18.81602	9.10001
3464	chr7	128784539	128784745	23.00	15.26920	6.85029
3465	chr7	128864331	128864527	21.00	17.13758	8.41110
3466	chr7	129078926	129079159	21.00	13.32019	6.27944
3467	chr7	129304779	129305111	27.00	31.39094	14.88386
3468	chr7	129464798	129465025	23.00	21.00607	10.08151
3469	chr7	129526134	129526331	21.00	18.07733	8.96484
3470	chr7	129536531	129536737	21.00	17.13758	8.41110
3471	chr7	129554726	129554901	15.00	12.67947	7.35640
3472	chr7	129598410	129598748	49.00	45.62112	14.27144
3473	chr7	129915639	129915862	21.00	13.86146	6.56726
3474	chr7	129932761	129933045	47.00	50.02967	17.37579
3475	chr7	129996050	129996355	25.00	19.32458	8.50728
3476	chr7	130013681	130014002	46.00	50.85356	18.27706
3477	chr7	130353454	130353795	77.00	106.91727	34.02439
3478	chr7	130538437	130538680	35.00	33.45447	13.03185
3479	chr7	130588828	130589119	48.00	46.91286	15.29778
3480	chr7	131271038	131271227	15.00	10.10091	5.79193
3481	chr7	131769712	131769979	40.00	37.55991	13.41532
3482	chr7	132458273	132458448	19.00	9.29158	4.61042
3483	chr7	132766820	132767152	69.00	58.43648	13.53311
3484	chr7	134331204	134331695	245.00	359.02292	58.13976
3485	chr7	134464068	134464320	30.00	19.48203	7.32656
3486	chr7	134758917	134759092	13.00	11.78681	7.27162
3487	chr7	134856398	134856676	37.00	36.97105	14.23754
3488	chr7	135538359	135538627	34.00	30.98894	12.03024
3489	chr7	137655338	137655537	24.00	13.49383	5.76302
3490	chr7	137686876	137687283	221.00	333.46143	60.92715
3491	chr7	138144990	138145172	16.00	12.66289	7.09728
3492	chr7	138575856	138576120	28.00	23.97067	10.17334
3493	chr7	138803168	138803351	18.00	13.54559	7.11877
3494	chr7	139045082	139045432	133.00	146.96486	25.90624
3495	chr7	139875016	139875446	151.00	162.94199	25.30381
3496	chr7	139877384	139877668	51.00	45.91910	13.71826
3497	chr7	139930869	139931073	22.00	14.28555	6.56486
3498	chr7	140397088	140397411	26.00	22.31380	9.87893
3499	chr7	140510935	140511260	27.00	26.90588	12.21388
3500	chr7	140927328	140927562	20.00	18.83289	9.72092
3501	chr7	147939350	147939668	27.00	28.92681	13.41733
3502	chr7	148638035	148638250	15.00	6.30094	3.68834
3503	chr7	148663857	148664086	18.00	14.51812	7.69630

3504	chr7	148725495	148725782	19.00	16.78471	8.78046
3505	chr7	148936512	148936727	24.00	17.53336	7.80499
3506	chr7	149570816	149571139	92.00	124.09187	34.84451
3507	chr7	150076181	150076521	82.00	108.97562	32.27650
3508	chr7	150521520	150521842	69.00	85.22098	26.22705
3509	chr7	150725223	150725649	124.00	171.02725	40.90036
3510	chr7	150725785	150725960	17.00	6.34524	3.47994
3511	chr7	150758067	150758299	18.00	16.23862	8.73573
3512	chr7	150810701	150810970	37.00	26.23695	8.75980
3513	chr7	150929358	150929734	174.00	279.84180	65.56763
3514	chr7	151217881	151218086	16.00	14.01657	7.92319
3515	chr7	151442534	151442778	27.00	27.87077	12.78736
3516	chr7	152011207	152011409	17.00	15.02847	8.27595
3517	chr7	155021976	155022333	30.00	27.72049	11.61484
3518	chr7	156803352	156803538	15.00	10.70334	6.15171
3519	chr7	158209036	158209348	63.00	87.62508	30.88556
3520	chr7	158225506	158225777	34.00	27.12109	9.99001
3521	chr7	158750681	158750866	17.00	11.06474	5.91809
3522	chr7	158898731	158898938	20.00	16.72589	8.45613
3523	chr8	681138	681458	72.00	110.24171	38.92355
3524	chr8	1711904	1712112	16.00	15.89157	9.06439
3525	chr8	1771886	1772164	43.00	55.71640	22.51022
3526	chr8	2965647	2965837	22.00	14.28555	6.56486
3527	chr8	8187935	8188168	32.00	30.30064	12.36418
3528	chr8	8458434	8458706	26.00	29.96288	14.39638
3529	chr8	8896378	8896602	12.00	10.35998	6.60115
3530	chr8	8937458	8937673	15.00	11.92080	6.89106
3531	chr8	10510525	10510726	14.00	9.45699	5.62008
3532	chr8	10657249	10657598	30.00	22.61202	8.84830
3533	chr8	12979536	12979801	30.00	28.13592	11.85200
3534	chr8	17323793	17324160	13.00	6.36973	3.99600
3535	chr8	18925000	18925230	21.00	13.32019	6.27944
3536	chr8	19243475	19243727	34.00	32.93251	13.11353
3537	chr8	19394622	19394859	13.00	11.18770	6.90300
3538	chr8	19466223	19466398	13.00	8.50004	5.24541
3539	chr8	22226766	22226977	19.00	14.62602	7.49344
3540	chr8	22926423	22926617	13.00	12.10322	7.46479
3541	chr8	22951727	22951970	19.00	14.62602	7.49344
3542	chr8	23183617	23183834	15.00	10.44156	5.99476
3543	chr8	23315157	23315465	35.00	42.46661	18.28010
3544	chr8	25159332	25159528	16.00	15.28459	8.69713
3545	chr8	25315847	25316287	176.00	325.35556	89.87714
3546	chr8	26240717	26241045	111.00	189.14120	59.71833
3547	chr8	26315534	26315762	17.00	16.33643	9.07239
3548	chr8	27168743	27169072	84.00	124.86993	40.16582
3549	chr8	27474746	27474941	15.00	10.44156	5.99476
3550	chr8	27729925	27730134	10.00	8.33931	5.71341
3551	chr8	27803011	27803242	14.00	12.44150	7.45002

3552	chr8	27874350	27874560	15.00	14.60207	8.53119
3553	chr8	28347748	28347923	14.00	9.45699	5.62008
3554	chr8	28560666	28560935	24.00	16.27042	7.13572
3555	chr8	29317198	29317541	82.00	115.10522	35.74739
3556	chr8	29594882	29595057	12.00	10.05135	6.40993
3557	chr8	29710906	29711088	14.00	10.83560	6.46037
3558	chr8	29891046	29891242	22.00	17.98963	8.61746
3559	chr8	30584493	30584668	13.00	12.06567	7.44193
3560	chr8	32185656	32185898	18.00	18.10711	9.86863
3561	chr8	36736514	36736727	17.00	16.56128	9.20873
3562	chr8	36737229	36738253	33.00	31.61030	12.73885
3563	chr8	36843389	36843673	32.00	30.30064	12.36418
3564	chr8	36930867	36931361	36.00	29.45019	10.56087
3565	chr8	37107277	37107485	20.00	19.88133	10.35450
3566	chr8	37160163	37160573	34.00	38.24411	16.20154
3567	chr8	37250582	37250969	13.00	11.87816	7.32752
3568	chr8	37278496	37278800	50.00	56.25368	19.49846
3569	chr8	37378769	37378970	18.00	18.54332	10.13079
3570	chr8	37456035	37456379	64.00	78.06566	24.85097
3571	chr8	37495451	37495789	15.00	12.67947	7.35640
3572	chr8	38243754	38244127	112.00	153.43071	38.84048
3573	chr8	38385600	38385845	16.00	14.10583	7.97780
3574	chr8	38854140	38854470	45.00	60.76950	24.52717
3575	chr8	41009060	41009277	19.00	14.62602	7.49344
3576	chr8	42010274	42010620	42.00	55.63624	22.92757
3577	chr8	49701344	49701596	22.00	22.56316	11.34064
3578	chr8	52628621	52628826	15.00	12.67947	7.35640
3579	chr8	53379030	53379260	22.00	17.98963	8.61746
3580	chr8	57123949	57124408	31.00	36.67606	16.49501
3581	chr8	60609751	60610000	19.00	19.84845	10.63133
3582	chr8	61566124	61566381	20.00	10.09393	4.84094
3583	chr8	61619810	61620056	15.00	14.60207	8.53119
3584	chr8	62627076	62627430	87.00	139.72694	46.92154
3585	chr8	64144478	64144659	12.00	10.89743	6.93159
3586	chr8	66556774	66557112	84.00	131.71320	44.14911
3587	chr8	66934440	66934677	20.00	17.71077	9.04452
3588	chr8	67368392	67368687	29.00	21.51802	8.56287
3589	chr8	67429693	67429917	20.00	19.39882	10.06300
3590	chr8	71314240	71314675	234.00	364.15048	67.07578
3591	chr8	74541187	74541362	10.00	8.06847	5.54424
3592	chr8	75232659	75232861	19.00	18.80980	10.00636
3593	chr8	75262467	75262724	23.00	25.47769	12.75760
3594	chr8	75541866	75542046	12.00	8.75023	5.59899
3595	chr8	77912438	77913145	68.00	102.59337	36.79076
3596	chr8	79578063	79578340	28.00	25.19533	10.86549
3597	chr8	80680020	80680319	33.00	40.80316	18.12878
3598	chr8	80996431	80996775	59.00	69.28559	22.48033
3599	chr8	89952646	89952871	11.00	9.72353	6.39839

3600	chr8	89972448	89972623	12.00	10.89743	6.93159
3601	chr8	90898223	90898471	28.00	25.19533	10.86549
3602	chr8	93978126	93978622	40.00	52.26358	21.86117
3603	chr8	95003882	95004166	13.00	12.10322	7.46479
3604	chr8	95071650	95071857	16.00	15.50331	8.82982
3605	chr8	95254447	95254638	16.00	12.31417	6.88616
3606	chr8	95369863	95370116	21.00	22.68483	11.73039
3607	chr8	95449498	95449833	65.00	67.50246	18.83831
3608	chr8	97159297	97159653	28.00	14.70246	5.60657
3609	chr8	97505648	97505979	50.00	69.50916	27.19317
3610	chr8	98775853	98776031	11.00	9.72353	6.39839
3611	chr8	101322644	101322945	51.00	70.77036	27.42733
3612	chr8	101859800	101859986	15.00	12.67947	7.35640
3613	chr8	101965013	101965340	63.00	92.54128	33.75671
3614	chr8	102063941	102064219	24.00	16.27042	7.13572
3615	chr8	102300344	102300552	21.00	16.84896	8.24279
3616	chr8	103549046	103549338	38.00	42.14300	16.79697
3617	chr8	103698040	103698265	17.00	16.01209	8.87529
3618	chr8	103875098	103875478	170.00	319.34842	91.17709
3619	chr8	107738149	107738487	64.00	95.04503	34.65796
3620	chr8	110547999	110548206	12.00	10.68938	6.80412
3621	chr8	117879202	117879412	19.00	19.57577	10.46788
3622	chr8	117959940	117960130	14.00	13.33873	7.99799
3623	chr8	118532662	118533038	156.00	287.51511	83.71230
3624	chr8	119044053	119044280	14.00	12.35372	7.39607
3625	chr8	119890644	119890937	37.00	30.63221	10.84630
3626	chr8	120212912	120213257	35.00	41.57148	17.75057
3627	chr8	122824086	122824299	23.00	15.26920	6.85029
3628	chr8	124054602	124054878	33.00	34.90736	14.64351
3629	chr8	124179434	124179609	14.00	12.44150	7.45002
3630	chr8	125486782	125487154	46.00	56.87135	21.75635
3631	chr8	126537607	126537877	27.00	16.22333	6.45459
3632	chr8	127837120	127837375	26.00	18.32292	7.70658
3633	chr8	128234892	128235170	97.00	30.65102	4.11352
3634	chr8	128235330	128235523	81.00	19.56473	3.25390
3635	chr8	128342829	128343011	14.00	11.77451	7.03923
3636	chr8	128961856	128962120	21.00	22.68483	11.73039
3637	chr8	129188931	129189391	23.00	22.85309	11.18567
3638	chr8	129335284	129335478	13.00	11.97110	7.38428
3639	chr8	130975160	130975367	23.00	19.14867	8.99213
3640	chr8	134586940	134587168	15.00	14.55884	8.50506
3641	chr8	141474365	141474632	25.00	24.00721	11.19798
3642	chr8	142348790	142348990	24.00	20.32534	9.36681
3643	chr8	144450747	144451090	130.00	229.87161	69.84911
3644	chr8	144512882	144513218	85.00	135.72733	45.85514
3645	chr8	145070043	145070260	13.00	11.10738	6.85337
3646	chr8	145070392	145070734	76.00	111.74635	37.42445
3647	chr8	145149616	145149899	25.00	28.47325	13.86318

3648	chr8	145159041	145159516	268.00	554.30798	143.43063
3649	chr8	145702968	145703226	27.00	31.46828	14.92958
3650	chr9	1083661	1083901	35.00	28.27971	10.27544
3651	chr9	2014982	2015366	123.00	192.72081	53.40574
3652	chr9	2087760	2087972	15.00	13.18532	7.66705
3653	chr9	2466099	2466427	58.00	70.67104	23.75768
3654	chr9	2466740	2466963	23.00	15.26920	6.85029
3655	chr9	3525875	3526245	62.00	81.44658	27.83793
3656	chr9	3777253	3777436	20.00	15.72746	7.86812
3657	chr9	4677495	4677753	31.00	32.86217	14.23227
3658	chr9	5879567	5879788	23.00	10.63663	4.63992
3659	chr9	6080182	6080644	29.00	34.52419	15.99598
3660	chr9	6152748	6153080	54.00	61.58029	20.60697
3661	chr9	14027196	14027477	24.00	18.96478	8.59303
3662	chr9	14030359	14030675	70.00	74.70468	20.26545
3663	chr9	14251333	14251547	14.00	11.24935	6.71524
3664	chr9	16203438	16203786	83.00	76.19734	16.23973
3665	chr9	16728090	16728343	31.00	30.69624	12.96218
3666	chr9	17894578	17894837	36.00	36.11326	14.14594
3667	chr9	19380215	19380390	11.00	8.13511	5.40775
3668	chr9	21111012	21111217	14.00	11.24935	6.71524
3669	chr9	21478975	21479166	14.00	9.45699	5.62008
3670	chr9	21988543	21988794	26.00	22.72887	10.11615
3671	chr9	22124748	22124945	18.00	5.84465	3.16298
3672	chr9	26947172	26947352	13.00	12.10322	7.46479
3673	chr9	27433521	27433795	11.00	6.67667	4.49607
3674	chr9	27573707	27573929	12.00	10.89743	6.93159
3675	chr9	28927805	28927986	11.00	9.72353	6.39839
3676	chr9	29044951	29045259	36.00	45.64589	19.72838
3677	chr9	33076695	33076876	16.00	15.72948	8.96663
3678	chr9	33225508	33225802	28.00	25.19533	10.86549
3679	chr9	34329196	34329571	19.00	18.57014	9.86143
3680	chr9	34620457	34620754	25.00	21.51895	9.74148
3681	chr9	34651886	34652442	80.00	120.22924	39.93878
3682	chr9	34989286	34989818	230.00	396.58771	86.54928
3683	chr9	35072489	35072957	35.00	43.41929	18.84219
3684	chr9	35511208	35511396	15.00	13.18532	7.66705
3685	chr9	35690096	35690348	26.00	27.21479	12.75856
3686	chr9	35936066	35936241	12.00	10.89743	6.93159
3687	chr9	37027644	37027923	32.00	38.22182	17.01048
3688	chr9	38641981	38642230	34.00	42.06298	18.46070
3689	chr9	73028828	73029182	26.00	29.96288	14.39638
3690	chr9	73033861	73034209	47.00	64.10442	25.51520
3691	chr9	74526154	74526498	40.00	52.26358	21.86117
3692	chr9	81486775	81487064	25.00	28.47325	13.86318
3693	chr9	82186204	82186775	82.00	129.53175	44.12003
3694	chr9	84301945	84302223	26.00	28.46988	13.50858
3695	chr9	84304691	84305070	157.00	245.84010	59.19821

3696	chr9	85781572	85781747	13.00	12.10322	7.46479
3697	chr9	86595053	86595462	172.00	245.69958	49.37920
3698	chr9	86876040	86876306	26.00	29.88839	14.35230
3699	chr9	88358203	88358443	15.00	14.60207	8.53119
3700	chr9	88897386	88897685	28.00	32.98889	15.46278
3701	chr9	89109509	89109793	20.00	16.81057	8.50643
3702	chr9	89987623	89987871	24.00	26.75781	13.18622
3703	chr9	89990506	89990681	9.00	7.38955	5.27449
3704	chr9	90408403	90408578	12.00	7.57257	4.87074
3705	chr9	91795289	91795478	21.00	13.32019	6.27944
3706	chr9	92276906	92277288	169.00	240.18855	48.52291
3707	chr9	94496710	94497002	43.00	45.34496	16.48558
3708	chr9	96215791	96216164	126.00	221.19147	67.71632
3709	chr9	96928142	96928594	67.00	100.50546	36.14653
3710	chr9	96928901	96929195	51.00	70.05431	27.00887
3711	chr9	97630404	97630607	16.00	14.47567	8.20398
3712	chr9	97681787	97682023	20.00	10.09393	4.84094
3713	chr9	97713209	97713496	47.00	43.02813	13.70059
3714	chr9	97810754	97810929	13.00	12.10322	7.46479
3715	chr9	98078634	98078919	42.00	52.69979	21.20207
3716	chr9	98271481	98271760	25.00	27.03737	13.00826
3717	chr9	99180569	99180919	89.00	143.74619	47.98794
3718	chr9	99380583	99380796	19.00	14.62602	7.49344
3719	chr9	99540319	99540653	49.00	67.74368	26.65997
3720	chr9	100564516	100564747	18.00	16.23862	8.73573
3721	chr9	101018338	101018560	12.00	9.35636	5.97708
3722	chr9	102582394	102582603	21.00	22.32345	11.51467
3723	chr9	104295574	104295996	157.00	245.84010	59.19821
3724	chr9	107690420	107690684	28.00	27.98681	12.49005
3725	chr9	107826867	107827085	24.00	16.27042	7.13572
3726	chr9	110006263	110006438	14.00	12.35372	7.39607
3727	chr9	110267126	110267340	19.00	11.44765	5.70858
3728	chr9	110811565	110811868	16.00	14.57150	8.26254
3729	chr9	111236196	111236462	26.00	29.88839	14.35230
3730	chr9	111240705	111241038	48.00	44.32025	13.98602
3731	chr9	111241391	111241633	22.00	17.98963	8.61746
3732	chr9	111305685	111305962	30.00	22.61202	8.84830
3733	chr9	111696362	111696623	17.00	16.33643	9.07239
3734	chr9	111880295	111880491	9.00	6.95804	5.00318
3735	chr9	112259784	112260047	24.00	26.41979	12.98503
3736	chr9	113318453	113318628	15.00	11.92080	6.89106
3737	chr9	115143078	115143293	20.00	19.88133	10.35450
3738	chr9	115480057	115480616	109.00	184.93109	58.65193
3739	chr9	117160647	117160866	16.00	15.89157	9.06439
3740	chr9	117314314	117314508	16.00	14.10583	7.97780
3741	chr9	117374002	117374250	24.00	26.99996	13.32998
3742	chr9	119449325	119449705	106.00	178.64548	57.05233
3743	chr9	123476671	123477011	84.00	129.46600	42.84183

3744	chr9	123883861	123884214	92.00	124.09187	34.84451
3745	chr9	124052124	124052351	21.00	22.17667	11.42683
3746	chr9	125811929	125812217	14.00	13.33873	7.99799
3747	chr9	126770497	126770720	18.00	17.50714	9.50604
3748	chr9	127021293	127021567	39.00	45.60815	18.39101
3749	chr9	127420448	127420838	67.00	81.98164	25.47771
3750	chr9	127421730	127422011	31.00	35.85825	16.01017
3751	chr9	127905733	127906116	140.00	251.79976	75.18111
3752	chr9	128003642	128004086	209.00	339.86456	72.18142
3753	chr9	128172471	128172806	114.00	129.70221	26.50991
3754	chr9	128508841	128509101	32.00	37.58385	16.63271
3755	chr9	129676859	129677216	158.00	280.02600	77.83469
3756	chr9	130007445	130007791	105.00	128.89023	30.25546
3757	chr9	130159044	130159335	22.00	21.65384	10.79349
3758	chr9	130487156	130487335	13.00	10.57714	6.52498
3759	chr9	130533589	130533909	58.00	66.37342	21.35775
3760	chr9	130564842	130565133	13.00	10.95033	6.75622
3761	chr9	130592957	130593248	34.00	39.20697	16.77167
3762	chr9	130681797	130682047	21.00	19.99769	10.11506
3763	chr9	130829516	130830394	258.00	412.02750	73.92608
3764	chr9	130955161	130955801	107.00	161.60327	46.51468
3765	chr9	131219049	131219313	30.00	27.72049	11.61484
3766	chr9	131320991	131321218	18.00	11.00162	5.67172
3767	chr9	131798502	131798870	98.00	117.59004	28.25746
3768	chr9	131940438	131940813	231.00	447.27087	114.39256
3769	chr9	131965527	131965758	24.00	23.93016	11.49438
3770	chr9	131988181	131988356	11.00	7.96760	5.30246
3771	chr9	132258146	132258489	81.00	115.72643	36.70999
3772	chr9	132332906	132333167	17.00	17.20576	9.59759
3773	chr9	132597573	132597902	95.00	139.39059	41.60959
3774	chr9	132646466	132646728	39.00	24.86141	7.73320
3775	chr9	133565344	133565677	53.00	60.06214	20.23230
3776	chr9	133822784	133823085	65.00	78.76765	24.72836
3777	chr9	134242228	134242435	16.00	11.45212	6.36943
3778	chr9	134248448	134249132	249.00	497.95947	127.89896
3779	chr9	134884649	134884840	13.00	11.69699	7.21657
3780	chr9	135448354	135448587	33.00	31.61030	12.73885
3781	chr9	136762926	136763151	19.00	17.47089	9.19550
3782	chr9	137533442	137533726	49.00	58.98668	21.53457
3783	chr9	137828285	137828460	19.00	11.44765	5.70858
3784	chr9	138843855	138844167	46.00	41.74495	13.41516
3785	chr9	139000279	139000470	16.00	11.45212	6.36943
3786	chr9	139686036	139686216	13.00	11.78681	7.27162
3787	chr9	139695530	139695863	41.00	51.07812	20.70900
3788	chr9	139729852	139730030	17.00	15.02847	8.27595
3789	chr9	139780625	139781026	203.00	382.11133	100.58656
3790	chr9	140117966	140118249	27.00	23.95451	10.49082
3791	chr9	140135352	140135830	221.00	435.47171	116.19348

3792	chr9	140147122	140147297	11.00	8.87876	5.87432
3793	chrX	3732910	3733222	33.00	40.80316	18.12878
3794	chrX	7895247	7895560	57.00	82.09486	30.92556
3795	chrX	8035889	8036163	23.00	19.14867	8.99213
3796	chrX	8222049	8222255	12.00	10.89743	6.93159
3797	chrX	9132619	9132850	11.00	9.72353	6.39839
3798	chrX	11129195	11129490	44.00	54.91177	21.56357
3799	chrX	11777482	11777816	78.00	98.26390	28.59766
3800	chrX	12965317	12965614	28.00	24.54824	10.49788
3801	chrX	13416330	13416517	19.00	14.62602	7.49344
3802	chrX	13670603	13670782	15.00	10.44156	5.99476
3803	chrX	14057607	14057784	25.00	21.51895	9.74148
3804	chrX	15511376	15511656	26.00	24.95212	11.41210
3805	chrX	17755357	17755695	30.00	33.97421	15.28521
3806	chrX	20285127	20285302	11.00	9.72353	6.39839
3807	chrX	23799657	23799897	33.00	33.41350	13.77232
3808	chrX	23949507	23949777	29.00	32.85976	15.00953
3809	chrX	24100110	24100307	13.00	10.43506	6.43685
3810	chrX	24164377	24164712	45.00	52.72791	19.81181
3811	chrX	31217364	31217630	11.00	6.67667	4.49607
3812	chrX	31227641	31227863	12.00	10.05135	6.40993
3813	chrX	39590164	39590396	18.00	16.95189	9.16915
3814	chrX	39667889	39668217	41.00	46.61858	18.08904
3815	chrX	39767450	39767643	15.00	13.73699	8.00508
3816	chrX	39788493	39788796	28.00	27.98681	12.49005
3817	chrX	39872429	39872790	56.00	80.11367	30.29930
3818	chrX	40333352	40333617	28.00	31.74527	14.72563
3819	chrX	40439861	40440275	141.00	189.91646	40.53090
3820	chrX	40882033	40882261	17.00	15.90723	8.81147
3821	chrX	41241106	41241313	18.00	16.23862	8.73573
3822	chrX	43931383	43931782	84.00	110.25735	31.84714
3823	chrX	44732048	44732386	147.00	267.33347	78.91351
3824	chrX	45305365	45305576	16.00	15.84551	9.03663
3825	chrX	45339541	45339776	12.00	9.83210	6.27363
3826	chrX	46184695	46185104	18.00	13.54559	7.11877
3827	chrX	46404524	46404772	23.00	12.61686	5.53250
3828	chrX	46988417	46989188	267.00	491.85834	108.55829
3829	chrX	47004427	47004665	20.00	19.88133	10.35450
3830	chrX	47077192	47077536	90.00	139.47723	44.86950
3831	chrX	47221067	47221414	74.00	80.57626	21.40717
3832	chrX	47221546	47221922	124.00	208.21309	61.63393
3833	chrX	47415526	47415858	63.00	88.77263	31.55657
3834	chrX	47420311	47420697	206.00	309.88397	59.19658
3835	chrX	47441478	47441732	33.00	25.97464	9.70458
3836	chrX	47483139	47483406	24.00	26.99996	13.32998
3837	chrX	47509992	47510760	47.00	37.38565	11.06501
3838	chrX	47518040	47518788	81.00	105.15163	30.72312
3839	chrX	47931129	47931452	43.00	32.80746	10.14292

3840	chrX	48554743	48555042	44.00	55.97284	22.18821
3841	chrX	48755000	48755280	57.00	69.31563	23.49396
3842	chrX	48815552	48815924	154.00	267.64554	73.75544
3843	chrX	48958024	48958372	88.00	117.13580	33.34583
3844	chrX	49028871	49029153	22.00	14.28555	6.56486
3845	chrX	49031124	49031339	19.00	19.57577	10.46788
3846	chrX	49047282	49048031	153.00	192.31326	35.50023
3847	chrX	49126040	49126265	19.00	19.90304	10.66399
3848	chrX	52004880	52005219	60.00	87.60279	32.52516
3849	chrX	52949861	52950200	77.00	114.55594	38.45957
3850	chrX	53024479	53024807	94.00	127.59798	35.59386
3851	chrX	53111187	53111460	23.00	25.54366	12.79678
3852	chrX	53122993	53123269	27.00	31.46828	14.92958
3853	chrX	54556379	54556726	71.00	92.39642	29.16491
3854	chrX	54665709	54665928	11.00	8.94684	5.91686
3855	chrX	55187503	55187845	49.00	61.48180	22.98876
3856	chrX	62974904	62975253	83.00	108.55029	31.47246
3857	chrX	63422805	63422980	9.00	7.48180	5.33199
3858	chrX	63425505	63425887	173.00	323.27759	91.07057
3859	chrX	68721290	68721591	37.00	44.69602	18.73671
3860	chrX	68776209	68776599	126.00	220.83221	67.50896
3861	chrX	68835498	68835990	89.00	143.74619	47.98794
3862	chrX	70288369	70288710	57.00	82.09486	30.92556
3863	chrX	70401825	70402212	166.00	296.58032	81.16732
3864	chrX	70474444	70474771	61.00	61.85760	17.69659
3865	chrX	70503238	70503630	130.00	225.85451	67.52644
3866	chrX	71401073	71401320	12.00	5.61615	3.71058
3867	chrX	72434629	72434950	35.00	41.57148	17.75057
3868	chrX	73640540	73640759	16.00	15.89157	9.06439
3869	chrX	73755686	73756000	78.00	101.18623	30.20349
3870	chrX	73756315	73756564	28.00	31.02884	14.29907
3871	chrX	74493634	74494073	220.00	436.21912	117.47621
3872	chrX	77040492	77040686	13.00	12.10322	7.46479
3873	chrX	77359364	77359789	141.00	244.16473	70.01614
3874	chrX	80020615	80020875	15.00	14.60207	8.53119
3875	chrX	80065463	80065638	13.00	12.10322	7.46479
3876	chrX	80377034	80377478	213.00	360.44980	80.17985
3877	chrX	84258698	84258898	19.00	19.90304	10.66399
3878	chrX	96549134	96549309	12.00	10.89743	6.93159
3879	chrX	102510064	102510328	18.00	17.28003	9.36836
3880	chrX	102611166	102611425	20.00	15.72746	7.86812
3881	chrX	106990849	106991096	16.00	15.89157	9.06439
3882	chrX	107334721	107334977	31.00	37.63698	17.06238
3883	chrX	107681544	107681875	66.00	98.61796	35.61496
3884	chrX	109129477	109129725	15.00	13.54753	7.88914
3885	chrX	110690628	110690932	23.00	25.54366	12.79678
3886	chrX	114795216	114795555	80.00	125.58893	43.05689
3887	chrX	114838038	114838244	15.00	14.60207	8.53119

3888	chrX	115415640	115415815	12.00	10.89743	6.93159
3889	chrX	117103322	117103555	14.00	13.33873	7.99799
3890	chrX	117208081	117208379	15.00	14.60207	8.53119
3891	chrX	117420066	117420386	15.00	10.44156	5.99476
3892	chrX	118740369	118740571	11.00	9.72353	6.39839
3893	chrX	118926040	118926226	14.00	12.35372	7.39607
3894	chrX	122993789	122993964	13.00	12.10322	7.46479
3895	chrX	128977772	128978145	73.00	91.77285	27.72574
3896	chrX	129307395	129307703	9.00	7.48180	5.33199
3897	chrX	131371266	131371441	12.00	10.89743	6.93159
3898	chrX	131601989	131602225	15.00	10.44156	5.99476
3899	chrX	132363501	132363695	15.00	14.60207	8.53119
3900	chrX	133500015	133500291	22.00	17.98963	8.61746
3901	chrX	133682518	133682702	13.00	12.10322	7.46479
3902	chrX	135282674	135282914	16.00	15.89157	9.06439
3903	chrX	135962840	135963224	173.00	326.23587	92.77669
3904	chrX	152735903	152736206	38.00	48.93276	20.79478
3905	chrX	153059379	153059716	57.00	82.09486	30.92556
3906	chrX	153234792	153234967	10.00	8.58401	5.86519
3907	chrX	153363242	153363592	51.00	71.28306	27.72637
3908	chrX	153656716	153657073	167.00	312.48376	89.57749
3909	chrX	154444402	154444786	62.00	86.97838	31.06350

Supplementary Table S8. List of affected genes in the LtoE siPREP1 HeLa cells.

ENSG00000101276	SLC52A3	0,0438278
ENSG00000232241	NA	0,0929155
ENSG00000186462	NAP1L2	0,1089938
ENSG00000168961	LGALS9	0,1251065
ENSG00000166033	HTRA1	0,1286722
ENSG00000181790	ADGRB1	0,1701479
ENSG00000221887	HMSD	0,1738285
ENSG00000235325	NA	0,1859678
ENSG00000254080	NA	0,1901936
ENSG00000221994	ZNF630	0,2053958
ENSG00000261114	NA	0,2139052
ENSG00000260086	NA	0,2160221
ENSG00000138798	EGF	0,2162736
ENSG00000213579	NA	0,2166716
ENSG00000103316	CRYM	0,2330343
ENSG00000204514	ZNF814	0,2378891
ENSG00000271851	NA	0,2431109
ENSG00000179603	GRM8	0,249258
ENSG00000204311	DFNB59	0,260374
ENSG00000134323	MYCN	0,2766963
ENSG00000250441	NA	0,2785424
ENSG00000273419	NA	0,3095534
ENSG00000213625	LEPROT	0,3098326
ENSG00000189423	NA	0,3230666
ENSG00000184486	POU3F2	0,3291197
ENSG00000173852	DPY19L1	0,3328429
ENSG00000132122	SPATA6	0,3386435
ENSG00000100228	RAB36	0,3400089
ENSG00000166750	SLFN5	0,3432601
ENSG00000232460	NA	0,3510878
ENSG00000171798	KNDC1	0,3548553
ENSG00000165424	ZCCHC24	0,3696279
ENSG00000188833	ENTPD8	0,3715659
ENSG00000169918	OTUD7A	0,3726082
ENSG00000104427	ZC2HC1A	0,3876275
ENSG00000223652	NA	0,3903172
ENSG00000157388	CACNA1D	0,393657
ENSG00000219891	NA	0,3937996
ENSG00000103647	CORO2B	0,3954614
ENSG00000147234	FRMPD3	0,3991128
ENSG00000172197	MBOAT1	0,3997589
ENSG00000213967	ZNF726	0,4003068
ENSG00000070915	SLC12A3	0,4053199
ENSG00000146243	IRAK1BP1	0,4084059
ENSG00000117114	ADGRL2	0,4093055
ENSG00000272750	NA	0,4109849
ENSG00000243244	STON1	0,4182753
ENSG00000176092	AIM1L	0,4187449
ENSG00000235590	GNAS-AS1	0,4315528
ENSG00000183496	MEX3B	0,435349
ENSG00000248697	NA	0,4395038

ENSG00000147100	SLC16A2	0,4406456
ENSG00000113161	HMGCR	0,4442872
ENSG00000065534	MYLK	0,4447039
ENSG00000230202	NA	0,4512674
ENSG00000153823	PID1	0,4555846
ENSG00000205403	CFI	0,4575255
ENSG00000163629	PTPN13	0,4584953
ENSG00000163053	SLC16A14	0,4636708
ENSG00000127928	GNGT1	0,4674781
ENSG00000011677	GABRA3	0,4681431
ENSG00000128266	GNAZ	0,4683146
ENSG00000185630	PBX1	0,4704902
ENSG00000226237	GAS1RR	0,4744409
ENSG00000140553	UNC45A	0,4766978
ENSG00000177054	ZDHHC13	0,4777299
ENSG00000253982	LOC101927	0,4778537
ENSG00000165868	HSPA12A	0,479083
ENSG00000225377	NA	0,4831008
ENSG00000099810	MTAP	0,4876366
ENSG00000183091	NEB	0,4884973
ENSG00000116704	SLC35D1	0,4891932
ENSG00000156011	PSD3	0,4899239
ENSG00000092871	RFFL	0,4917476
ENSG00000267156	NA	0,4929594
ENSG00000139304	PTPRQ	0,4959852
ENSG00000106018	VIPR2	0,5087571
ENSG00000110660	SLC35F2	0,5110414
ENSG00000111110	PPM1H	0,5169196
ENSG00000111450	STX2	0,517535
ENSG00000236813	NA	0,5175665
ENSG00000180537	RNF182	0,5220171
ENSG00000033170	FUT8	0,522328
ENSG00000177683	THAP5	0,5233776
ENSG00000137726	FXYD6	0,5271697
ENSG00000137726	FXYD6-FXYI	0,5271697
ENSG00000260766	NA	0,5286402
ENSG00000074181	NOTCH3	0,530051
ENSG00000047634	SCML1	0,5302361
ENSG00000270804	NA	0,5347722
ENSG00000164983	TMEM65	0,5354286
ENSG00000031081	ARHGAP31	0,5370942
ENSG00000090612	ZNF268	0,5397425
ENSG00000181467	RAP2B	0,5410102
ENSG00000269416	LINC01224	0,5437811
ENSG00000182310	SPACA6	0,5439547
ENSG00000168264	IRF2BP2	0,5443713
ENSG00000169435	RASSF6	0,5448448
ENSG00000167555	ZNF528	0,5484721
ENSG00000072840	EVC	0,5500042
ENSG00000112149	CD83	0,5512015
ENSG00000135842	FAM129A	0,5559238
ENSG00000145687	SSBP2	0,5561053

ENSG00000197766	CFD	0,5595513
ENSG00000137198	GMPR	0,5630424
ENSG00000107771	CCSER2	0,5669786
ENSG00000184867	ARMCX2	0,5677362
ENSG00000006638	TBXA2R	0,5684171
ENSG00000228106	DISP1	0,5694953
ENSG00000228106	LOC102724	0,5694953
ENSG00000239407	NA	0,5726067
ENSG00000137460	FHDC1	0,5741941
ENSG00000182518	FAM104B	0,574539
ENSG00000205221	VIT	0,5774016
ENSG00000109618	SEPSECS	0,5782472
ENSG00000120832	MTERF2	0,5806444
ENSG00000181444	ZNF467	0,5806805
ENSG00000128536	CDHR3	0,5810373
ENSG00000198780	FAM169A	0,5847377
ENSG00000178966	RMI1	0,5857775
ENSG00000156475	PPP2R2B	0,5858186
ENSG00000186806	VSIG10L	0,5861306
ENSG00000152359	POC5	0,5868339
ENSG00000226278	NA	0,58685
ENSG00000154309	DISP1	0,5870812
ENSG00000151575	TEX9	0,5873427
ENSG00000163923	RPL39L	0,5875864
ENSG00000127990	SGCE	0,5884621
ENSG00000163412	EIF4E3	0,5911618
ENSG00000143061	IGSF3	0,5925942
ENSG00000160305	DIP2A	0,5953548
ENSG00000079156	OSBPL6	0,5978267
ENSG00000153879	CEBPG	0,5990043
ENSG00000105289	TJP3	0,601559
ENSG00000165650	PDZD8	0,6017204
ENSG00000138376	BARD1	0,6033245
ENSG00000064961	HMG20B	0,6058784
ENSG00000145919	BOD1	0,6076877
ENSG00000064309	CDON	0,613355
ENSG00000137338	PGBD1	0,616109
ENSG00000164695	CHMP4C	0,6185814
ENSG00000224113	NA	0,6225613
ENSG00000153234	NR4A2	0,6244772
ENSG00000140263	SORD	0,6275494
ENSG00000120549	KIAA1217	0,6278389
ENSG00000169621	PROKR1	0,6283764
ENSG00000169621	APLF	0,6283764
ENSG00000163634	THOC7	0,6292142
ENSG00000157570	TSPAN18	0,6321989
ENSG00000185015	CA13	0,632431
ENSG00000184349	EFNA5	0,6326643
ENSG00000118418	HMGN3	0,6346926
ENSG00000145781	COMMD10	0,6362906
ENSG00000186487	MYT1L	0,6373305
ENSG00000261786	NA	0,63734

ENSG00000108272	NA	0,637859
ENSG00000121644	DESI2	0,6384647
ENSG00000133678	TMEM254	0,6390968
ENSG00000005156	LIG3	0,6405725
ENSG00000182372	CLN8	0,6418267
ENSG00000105875	WDR91	0,6445109
ENSG00000109458	GAB1	0,6461393
ENSG00000213853	EMP2	0,6474994
ENSG00000183117	CSMD1	0,6480043
ENSG00000105258	POLR2I	0,6489455
ENSG00000128710	HOXD10	0,6501035
ENSG00000233588	NA	0,6523015
ENSG00000187210	GCNT1	0,6529271
ENSG00000184588	PDE4B	0,6581178
ENSG00000231831	NA	0,6583271
ENSG00000172845	SP3	0,6598952
ENSG00000159882	ZNF230	0,6602452
ENSG00000085117	CD82	0,6605331
ENSG00000198081	ZBTB14	0,6607532
ENSG00000133111	RFXAP	0,6623279
ENSG00000099624	ATP5D	0,6632855
ENSG00000152495	CAMK4	0,6633766
ENSG00000261578	NA	0,6661461
ENSG00000204789	NA	0,6669483
ENSG00000183137	CEP57L1	0,6670925
ENSG00000112276	BVES	0,6676744
ENSG00000175745	NR2F1	0,6678771
ENSG00000204934	ATP6V0E2-/	0,6679598
ENSG00000185305	ARL15	0,6681318
ENSG00000196139	AKR1C3	0,6694245
ENSG00000145675	PIK3R1	0,6696791
ENSG00000146282	RARS2	0,6699408
ENSG00000165591	FAAH2	1,5005924
ENSG00000179454	KLHL28	1,5181176
ENSG00000213096	ZNF254	1,524943
ENSG00000221930	NA	1,5358811
ENSG00000196204	RNF216P1	1,5371674
ENSG00000260482	NA	1,5386802
ENSG00000222041	LINC00152	1,5416494
ENSG00000222041	MIR4435-2I	1,5416494
ENSG00000204291	COL15A1	1,5441484
ENSG00000113845	TIMMDC1	1,5457498
ENSG00000235105	NA	1,5793723
ENSG00000128590	DNAJB9	1,5838922
ENSG00000196357	ZNF565	1,5930955
ENSG00000163884	KLF15	1,5995544
ENSG00000081692	JMJD4	1,6072229
ENSG00000185875	THNSL1	1,6155021
ENSG00000241293	NA	1,6188077
ENSG00000132640	BTBD3	1,6239453
ENSG00000154822	PLCL2	1,6367521
ENSG00000250903	NA	1,6390274

ENSG00000205213	LGR4	1,6413461
ENSG00000200534	SNORA33	1,6429633
ENSG00000124596	OARD1	1,6438478
ENSG00000172965	MIR4435-2I	1,6498169
ENSG00000125510	OPRL1	1,6705445
ENSG00000153094	BCL2L11	1,6718452
ENSG00000178172	SPINK6	1,6833564
ENSG00000117226	GBP3	1,6848387
ENSG00000184508	HDDC3	1,6881179
ENSG00000237036	NA	1,7085542
ENSG00000227403	LOC100996	1,7135873
ENSG00000121578	B4GALT4	1,7212635
ENSG00000247095	MIR210HG	1,7441985
ENSG00000143641	GALNT2	1,7456308
ENSG00000153395	LPCAT1	1,7497296
ENSG00000180425	C11orf71	1,7529814
ENSG00000215386	MIR99AHG	1,7572254
ENSG00000145244	CORIN	1,7842713
ENSG00000270362	HMGN3-AS	1,7860917
ENSG00000144158	NA	1,7861478
ENSG00000185127	C6orf120	1,7920676
ENSG00000239523	MYLK-AS1	1,8020626
ENSG00000003436	TFPI	1,8025117
ENSG00000203813	NA	1,8200177
ENSG00000270574	NA	1,825022
ENSG00000213683	NA	1,8786603
ENSG00000171316	CHD7	1,8857149
ENSG00000247796	LOC257396	1,910671
ENSG00000221500	SNORD100	2,0098632
ENSG00000109452	INPP4B	2,0503047
ENSG00000175003	SLC22A1	2,0738168
ENSG00000235008	NA	2,0774238
ENSG00000006756	ARSD	2,0870478
ENSG00000166741	NNMT	2,1634273
ENSG00000154438	ASZ1	2,1931308
ENSG00000134363	FST	2,2284028
ENSG00000260329	NA	2,2329698
ENSG00000091831	ESR1	2,2718147
ENSG00000198879	SFMBT2	2,3052378
ENSG00000124882	EREG	2,3071511
ENSG00000247317	NA	2,358686
ENSG00000182585	EPGN	2,4783992
ENSG00000215417	MIR17HG	2,4823601
ENSG00000260806	NA	2,4906486
ENSG00000064692	SNCAIP	2,6050378
ENSG00000112539	C6orf118	2,6254895
ENSG00000124602	UNC5CL	2,7620373
ENSG00000263013	NA	3,3360892
ENSG00000117228	GBP1	3,9815694
ENSG00000174473	GALNTL6	4,5122275
ENSG00000187601	MAGEH1	4,6351169
ENSG00000254143	NA	4,9180498

ENSG00000205090	TMEM240	15,481776
ENSG00000188107	EYS	17,507905

Supplementary Table S9. Genes present in EtoL shifts and affected by PREP1 DR.

ENSG00000C	NA	0,0984661
ENSG00000C	JMJD7	0,1082326
ENSG00000C	LOC101928	0,112379
ENSG00000C	MIR5094	0,127
ENSG00000C	OASL	0,1305462
ENSG00000C	AQP3	0,1508852
ENSG00000C	TMEM170E	0,2131953
ENSG00000C	NA	0,2219743
ENSG00000C	GAL3ST4	0,2290462
ENSG00000C	CTSK	0,2366875
ENSG00000C	NA	0,2414576
ENSG00000C	ZMYND10	0,2514804
ENSG00000C	HPGD	0,2778139
ENSG00000C	NPPC	0,2788736
ENSG00000C	PPIP5K2	0,2799182
ENSG00000C	COL5A3	0,2831592
ENSG00000C	NA	0,2993402
ENSG00000C	PRRT2	0,3006171
ENSG00000C	NA	0,3073036
ENSG00000C	PRKCG	0,3233246
ENSG00000C	NA	0,3363886
ENSG00000C	NA	0,3384936
ENSG00000C	SLC41A1	0,3478163
ENSG00000C	WNK4	0,3494346
ENSG00000C	SDF2	0,3532074
ENSG00000C	PAK2	0,378888
ENSG00000C	SH3BP5	0,3904551
ENSG00000C	NPR3	0,3971836
ENSG00000C	SLC38A3	0,3973477
ENSG00000C	LEAP2	0,4029093
ENSG00000C	CRABP2	0,4101766
ENSG00000C	TRPM4	0,4104231
ENSG00000C	NA	0,4206618
ENSG00000C	GLIPR2	0,422429
ENSG00000C	NA	0,4275629
ENSG00000C	CORO1A	0,4354306
ENSG00000C	ADGRB2	0,4403759
ENSG00000C	FOPNL	0,4421117
ENSG00000C	RAB14	0,4480033
ENSG00000C	LITAF	0,4554907
ENSG00000C	HP1BP3	0,4581529
ENSG00000C	FZD5	0,4583075
ENSG00000C	FOSB	0,4616491
ENSG00000C	CDIPT	0,4628674
ENSG00000C	PNPO	0,4703031
ENSG00000C	MAPK9	0,4726634
ENSG00000C	TCIRG1	0,4762534
ENSG00000C	SLC44A2	0,4775751
ENSG00000C	LIPH	0,478995
ENSG00000C	EFR3B	0,4798401
ENSG00000C	PDE4A	0,4845403

ENSG00000(	SEMA3F	0,4864139
ENSG00000(	ANKRD36BI	0,4887679
ENSG00000(	DGKA	0,4931392
ENSG00000(	NA	0,4963519
ENSG00000(	PLCE1	0,4987429
ENSG00000(	PPP2R5A	0,5025025
ENSG00000(	FREM2	0,5081766
ENSG00000(	DNM1	0,5104586
ENSG00000(	GIPR	0,5123275
ENSG00000(	FAM126A	0,5159616
ENSG00000(	APOBEC3C	0,5181344
ENSG00000(	ARPIN	0,5186053
ENSG00000(	CYTH3	0,5208648
ENSG00000(	CELSR2	0,5226154
ENSG00000(	APOBEC3B	0,5237258
ENSG00000(	MINPP1	0,5246431
ENSG00000(	GALNT16	0,5256077
ENSG00000(	MYO15B	0,531562
ENSG00000(	AGO1	0,5323549
ENSG00000(	FAM46B	0,5349168
ENSG00000(	IFNAR1	0,5375496
ENSG00000(	CD70	0,5395897
ENSG00000(	TWSG1	0,5410694
ENSG00000(	EML2	0,547658
ENSG00000(	CEP44	0,5548821
ENSG00000(	MUC3A	0,5560309
ENSG00000(	CASKIN2	0,5607612
ENSG00000(	ZSWIM5	0,5642079
ENSG00000(	HEXIM1	0,5691415
ENSG00000(	KIF3A	0,5722744
ENSG00000(	MANEAL	0,5723685
ENSG00000(	MAP6D1	0,5769586
ENSG00000(	STOX1	0,5788619
ENSG00000(	RPIA	0,5804256
ENSG00000(	NA	0,584151
ENSG00000(	PPFIBP1	0,5861695
ENSG00000(	MAMDC4	0,5877736
ENSG00000(	OLFM2	0,5884109
ENSG00000(	CPD	0,6012171
ENSG00000(	RP2	0,6016299
ENSG00000(	NUCKS1	0,6026374
ENSG00000(	PILRB	0,6057468
ENSG00000(	SLC41A2	0,6058895
ENSG00000(	RARG	0,6059274
ENSG00000(	NA	0,6078551
ENSG00000(	UNC13D	0,6088406
ENSG00000(	MAP4K2	0,6116356
ENSG00000(	ADAM11	0,6150761
ENSG00000(	HRK	0,6177493
ENSG00000(	TUBB4A	0,6180137
ENSG00000(	BSDC1	0,6180372
ENSG00000(	CBWD1	0,6188918

ENSG00000	BMPR1A	0,6192041
ENSG00000	IGFBP6	0,6197054
ENSG00000	KIAA0100	0,6207758
ENSG00000	MED20	0,6208774
ENSG00000	CREB1	0,622376
ENSG00000	PROCA1	0,6246642
ENSG00000	KIF18B	0,6291383
ENSG00000	SMIM19	0,6319317
ENSG00000	DDB2	0,6322913
ENSG00000	CD3EAP	0,6334239
ENSG00000	TUBA1A	0,6343425
ENSG00000	AAMDC	0,637495
ENSG00000	KCNH3	0,639164
ENSG00000	C1QL4	0,6394057
ENSG00000	CSAD	0,6413754
ENSG00000	CRABP1	0,6443971
ENSG00000	ARSJ	0,6449339
ENSG00000	CHP1	0,6453533
ENSG00000	SLC47A1	0,6454881
ENSG00000	GATS	0,645788
ENSG00000	ZBTB44	0,6465466
ENSG00000	UBL7-AS1	0,646801
ENSG00000	KIF13A	0,6481289
ENSG00000	RTKN2	0,6520625
ENSG00000	ATG4D	0,6543299
ENSG00000	SAP30	0,6545669
ENSG00000	FBXW4	0,6586549
ENSG00000	EML2-AS1	0,6593325
ENSG00000	RTN2	0,6611598
ENSG00000	SUOX	0,6621586
ENSG00000	FANCL	0,6627876
ENSG00000	PHF21A	0,6630224
ENSG00000	RYK	0,6633897
ENSG00000	AP1M2	0,6658627
ENSG00000	MAST3	0,6659501
ENSG00000	DDX59	0,6681756
ENSG00000	ACADS	0,6682904
ENSG00000	LCMT2	0,6687361
ENSG00000	SLC25A45	1,5001087
ENSG00000	CLDN15	1,5029652
ENSG00000	RPS6KB2	1,510105
ENSG00000	SH2D2A	1,5150649
ENSG00000	GALNT7	1,5170958
ENSG00000	EAF1	1,5176519
ENSG00000	MMP24	1,5184255
ENSG00000	GPR176	1,5194098
ENSG00000	ZC3H6	1,5199557
ENSG00000	ABCC5	1,5245887
ENSG00000	GTF2H5	1,5308047
ENSG00000	TPM1	1,5359357
ENSG00000	ARID3B	1,5368906
ENSG00000	NA	1,5457736

ENSG00000C	CR2	1,5882008
ENSG00000C	NEDD9	1,5904879
ENSG00000C	TMED5	1,5947709
ENSG00000C	YIPF2	1,5986076
ENSG00000C	ZFYVE26	1,6089902
ENSG00000C	CSF1	1,612845
ENSG00000C	TRAF2	1,6255551
ENSG00000C	TET1	1,6304925
ENSG00000C	PAM16	1,6305775
ENSG00000C	POLD4	1,6452238
ENSG00000C	NTN4	1,645334
ENSG00000C	APOLD1	1,6687731
ENSG00000C	SPRYD3	1,6774367
ENSG00000C	CD320	1,6996147
ENSG00000C	SNORD4A	1,7131322
ENSG00000C	RBPMS2	1,7145825
ENSG00000C	SEMA7A	1,7305157
ENSG00000C	FBXO8	1,7328337
ENSG00000C	TUSC2	1,7355991
ENSG00000C	ARID5B	1,7451309
ENSG00000C	INA	1,7516968
ENSG00000C	FAM222B	1,7569445
ENSG00000C	SPAG4	1,7583003
ENSG00000C	HSD17B6	1,7685678
ENSG00000C	KLLN	1,7685848
ENSG00000C	NOP14-AS1	1,7743162
ENSG00000C	NA	1,778826
ENSG00000C	C1orf52	1,7911916
ENSG00000C	MKLN1-AS	1,819593
ENSG00000C	HN1	1,8211959
ENSG00000C	ATAD1	1,8237128
ENSG00000C	STAMBPL1	1,8270886
ENSG00000C	NA	1,8376192
ENSG00000C	ILF3-AS1	1,8442238
ENSG00000C	ARHGEF39	1,8456609
ENSG00000C	SP2	1,8524387
ENSG00000C	LRRC46	1,8645979
ENSG00000C	C12orf60	1,9084061
ENSG00000C	PLK3	1,9135803
ENSG00000C	TGM2	1,9219728
ENSG00000C	UGCG	1,9286022
ENSG00000C	CYP11A1	1,9475932
ENSG00000C	IFIT2	1,9486824
ENSG00000C	TMEM234	1,9559146
ENSG00000C	DDAH1	1,9811324
ENSG00000C	AOC2	2,0208283
ENSG00000C	AOC3	2,0214683
ENSG00000C	IL6	2,0295862
ENSG00000C	NA	2,0331103
ENSG00000C	TMC7	2,0463434
ENSG00000C	GREB1L	2,0527527
ENSG00000C	YRDC	2,0760223

ENSG00000(	VEGFC	2,0799765
ENSG00000(	SLC25A41	2,1040608
ENSG00000(	ACTA2	2,1200123
ENSG00000(	NA	2,1479262
ENSG00000(	ZBTB37	2,1759057
ENSG00000(	ISM2	2,2432292
ENSG00000(	NA	2,3549931
ENSG00000(	RPL13AP20	2,5348355
ENSG00000(	F3	2,5573179
ENSG00000(	RNF112	2,5618533
ENSG00000(	NA	2,5657359
ENSG00000(	ANGPTL4	2,5803008
ENSG00000(	SERTAD4-A	2,6865992
ENSG00000(	ST14	2,8233254
ENSG00000(	ZCWPW1	2,8718532
ENSG00000(	TSACC	2,9009895
ENSG00000(	LOC541472	2,9379126
ENSG00000(	CD72	3,0907364
ENSG00000(	TNFRSF21	3,2040536
ENSG00000(	NA	3,3419689
ENSG00000(	NA	3,4687559
ENSG00000(	NA	3,6089906
ENSG00000(	ETV4	3,6922456
ENSG00000(	HMGA2	3,8470356
ENSG00000(	NA	3,9645362
ENSG00000(	NA	4,1322597
ENSG00000(	NA	4,6428463
ENSG00000(	NA	4,648017
ENSG00000(	ARHGDIB	4,7478939
ENSG00000(	NA	5,2699699
ENSG00000(	APOL3	7,9589422
ENSG00000(	NA	19,908715

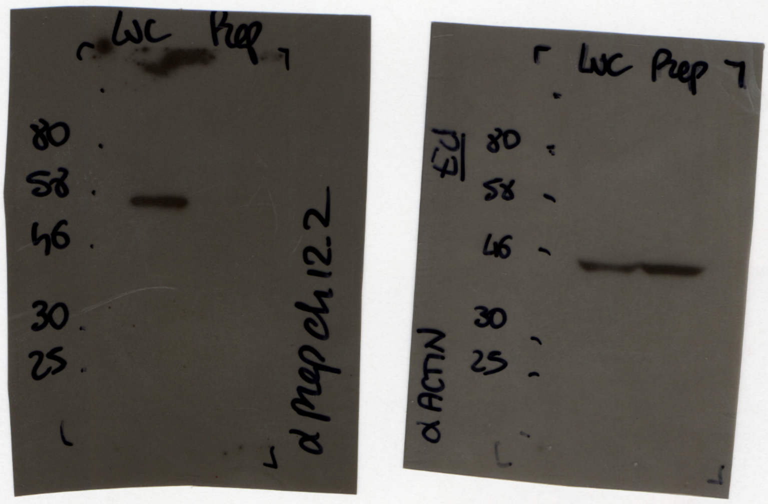

WC Prep
80
58
46
30
25
WC Prep
80
58
46
30
25
α ACTN

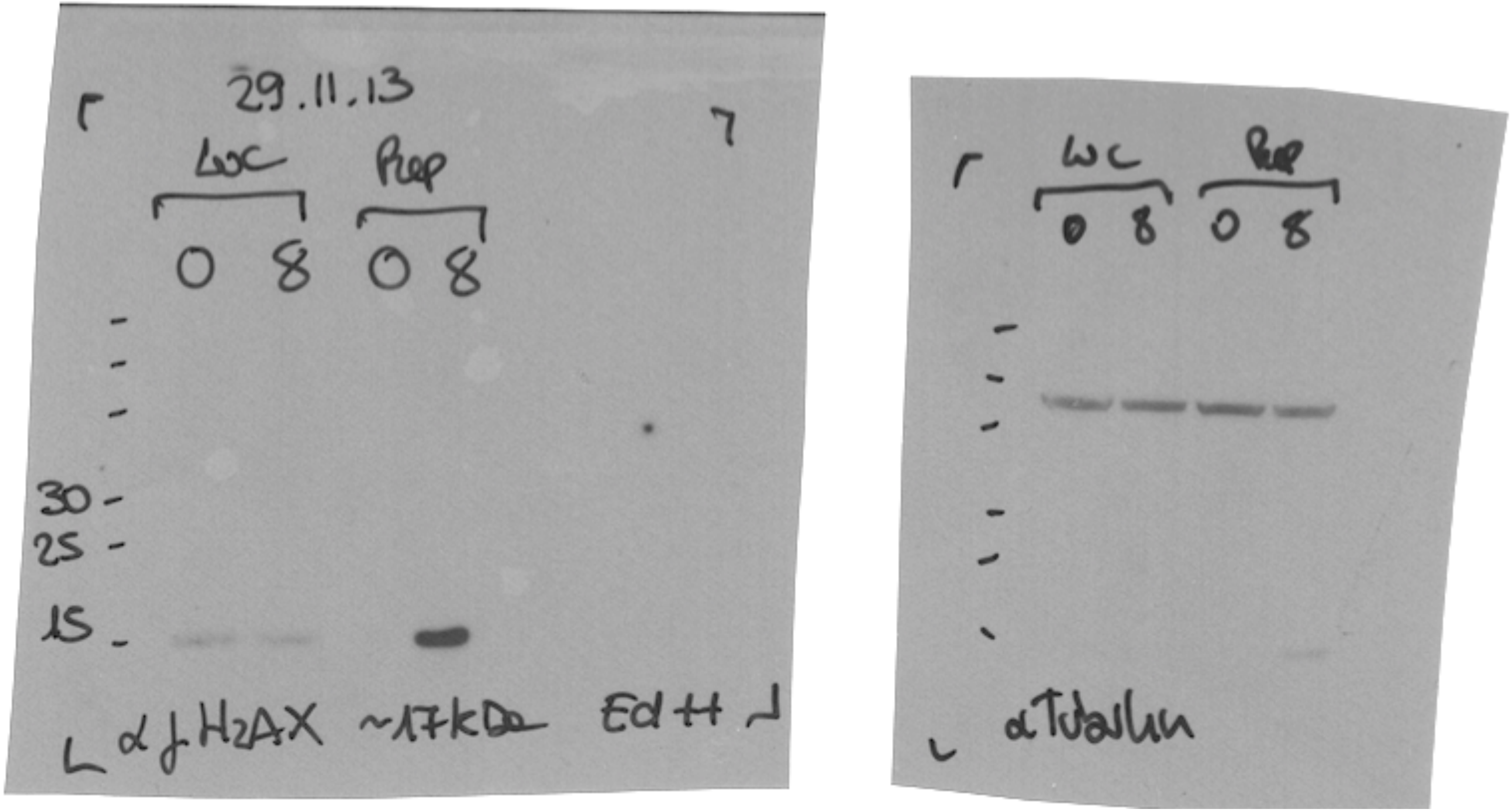

Full length blot of siLuc and siPrep cells treated with a double-thymidne block, incubated for 0 or 8 hrs in complete medium, lysed, electrophoresed (11% Acrylamide gel), blotted and treated with gH2Ax (left) or α-Tubulin antibody. This blot refers to Figure 2G.

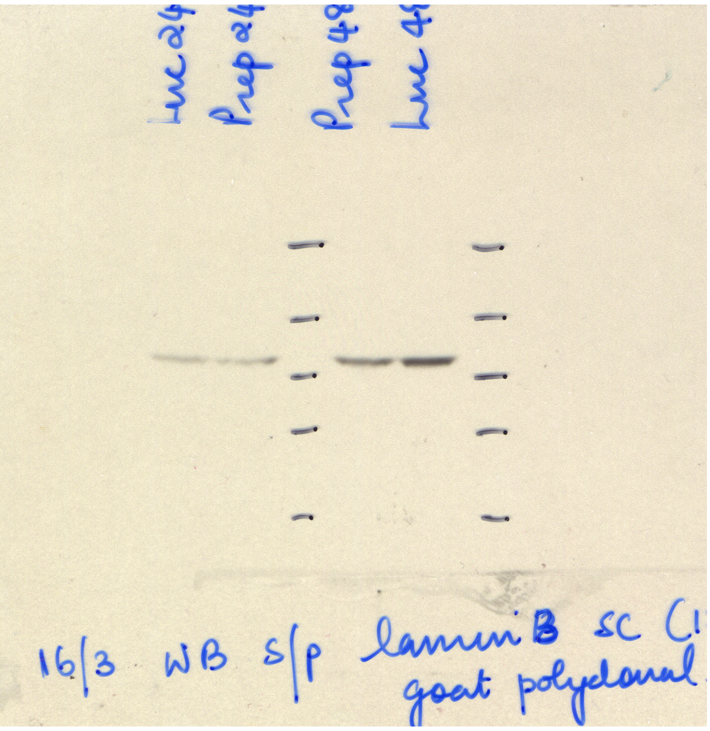
Luc 24
Prep 24
Prep 48
Luc 48
16/3 WB S/P laminB sc (1
goat polyclonal

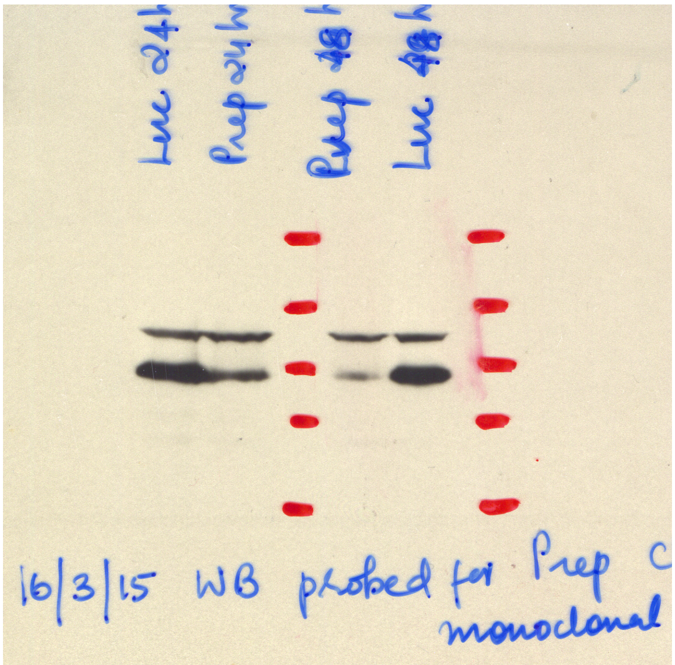
Luc 24h
Prep 24h
Prep 48h
Luc 48h
16/3/15 WB probed for Prep C
monoclonal

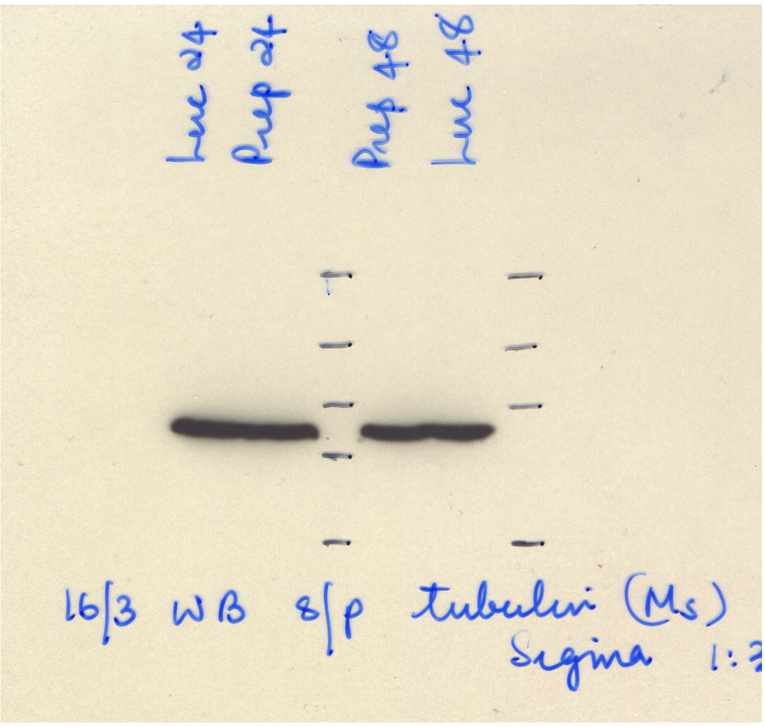
Luc 24
Prep 24
Prep 48
Luc 48
16/3 WB 8/p tubulin (Ms)
Sigma
